# Pan-genome insights into the evolutionary dynamics and functional diversification of genes in monkeypox virus

**DOI:** 10.7717/peerj.21846

**Published:** 2026-10-26

**Authors:** Yan Liu, Yinggen Tang, Yunhai Xu, Xizi Long, Zhaozhong Zhu

**Affiliations:** 1 School of Public Health, University of South China, Hengyang, China; 2 Hunan Provincial Key Laboratory for Geochemical Processes and Resource Environmental Effects, Changsha, China; 3 Hunan Provincial Key Laboratory of Medical Virology, Bioinformatics Center, College of Biology, Hunan University, Changsha, China

**Keywords:** Monkeypox virus, Gene number variation, Evolution

## Abstract

Monkeypox virus (MPXV) continues to emerge as a global health threat. While genetically diverse, its genomic structure and evolution remain undercharacterized. This study analyzed 157 non-redundant MPXV genomes (subtypes Ia, Ib, IIa, IIb), identifying 183 core and 222 non-core genes *via* pan-genome analysis—indicative of an open pan-genome. Over half (55.8%) of MPXV genes show gene number variation. Non-core genes exhibit a higher ratio of insertions/deletions compared to core genes. Additionally, selection pressure analysis indicated that non-core genes are more prone to positive selection. Furthermore, interaction analysis revealed that core viral proteins target immune-related human pathways, while non-core proteins engage with viral infection-associated proteins. These results deepen our understanding of MPXV evolution and inform future research directions.

## Introduction

Monkeypox virus (MPXV), a double-stranded DNA virus belonging to the *Orthopoxvirus* genus within the *Poxviridae* family, was first identified in 1958 in monkey colonies in Denmark (Parker & Buller, 2013; Karagoz et al., 2023). MPXV infection typically causes fever, intense headache, swollen lymph nodes (lymphadenopathy), muscle aches (myalgia), back pain, and profound fatigue (asthenia) within 5 to 21 days of exposure (Lum et al., 2022). The first human case emerged in 1970 in the Democratic Republic of the Congo, with subsequent endemic outbreaks in Central and West Africa (Sun et al., 2024). Since the first non-African case in the United States of America in 2003, MPXV has spread globally, causing sporadic outbreaks in Asia, Europe, and the Americas (Reynolds et al., 2007). A major surge began in May 2022, with Europe and the Americas most affected (Gong et al., 2022; Luna et al., 2022; Zinnah et al., 2024). On July 23, 2022, the World Health Organization (WHO) declared the outbreak a Public Health Emergency of International Concern (PHEIC) (Wei et al., 2022). Although the dataset spans multiple genotypes and decades, certain lineages (*e.g.*, clade Ib) remain underrepresented, which may limit the resolution of genotype-specific evolutionary inferences (Sahay et al., 2026). By September 2023, cases had been reported in 114 countries, totaling over 89,000 infections and 157 deaths (Bruno & Buccoliero, 2023).

The pan-genome encompasses all genes across strains of a bacterial species, moving beyond single reference genomes (Medini et al., 2005). In virology, this approach enables broad genomic comparisons, distinguishing core from accessory genes to reveal viral diversity and evolution. For instance, pan-genome analyses of influenza viruses have linked variable accessory genes to antigenic drift and immune evasion (Zhang et al., 2020), while studies of African swine fever virus (ASFV) highlight accessory genes involved in replication and host interaction (Wang et al., 2020). These insights illustrate the power of pan-genome in understanding viral pathogenesis and guiding antiviral strategies. Within this evolutionary context, poxviruses utilize ‘genomic accordion’ mechanisms, driven by recombination and repeat-associated instability, to rapidly adapt through transient gene copy number variations (Monzón et al., 2024). This structural plasticity represents a critical evolutionary strategy that enables large DNA viruses to refine their genomes under selective pressure.

MPXV exhibits substantial genetic diversity, necessitating comprehensive genomic analysis to support effective epidemic response (Luna et al., 2023). Recent studies have identified polymorphic repeat regions and microsatellite-associated variability in MPXV genomes, which are thought to contribute to genome plasticity and rapid evolution (Agarwal et al., 2024; Monzón et al., 2024). These repeat elements can act as mutational hotspots, facilitating insertions, deletions, and replication slippage events that may alter gene function or regulation (Saeed, Wang & Wang, 2016). Such mechanisms are particularly relevant in large DNA viruses, where structural variation can drive host adaptation, immune evasion, and phenotypic diversity (Elde et al., 2012).

Yu et al. (2024) show that MPXV terminal regions are hotspots for genetic variation—including SNPs, gene gain/loss, and recombination—driving viral evolution. Analysis of 404 genomes identified clade-specific microsatellite patterns (I/IIa/IIb), 26 conserved diagnostic loci, and nine mutational hotspot genes, including OPG153 with pathogenicity-linked “(ATC)n” repeats (Agarwal et al., 2024). Unlike traditional reference-based methods, pan-genome analysis captures both core and accessory variation, improving insights into MPXV evolution (Andriyanov et al., 2024). However, most MPXV studies rely on limited genomes and focus on phylogeny or mutations, leaving genome-wide variation and selection poorly understood (Isidro et al., 2022; Masirika et al., 2025). Here, we apply an integrated pan-genome framework to distinguish core/non-core genes and quantify their evolutionary dynamics across populations.

Due to its increasing geographic spread and potential for significant public health consequences, MPXV has garnered widespread attention. In this study, 157 MPXV genomes from NCBI and GISAID database were analyzed using pan-genomic approaches, revealing 183 core and 222 non-core genes. The MPXV genome exhibits an open configuration, with phylogenetic analysis showing that over 55.8% of genes display gene number variation. Non-core genes show a higher ratio of insertions/deletions than core genes, highlighting distinct evolutionary patterns. Non-core genes also display stronger positive selection than core genes. Notably, human proteins interacting with core viral proteins show greater enrichment in immune-related pathways than those linked to non-core proteins. These results provide valuable insights into MPXV evolution and inform the development of targeted countermeasures.

## Methods

### Collection of data resources and phylogenetic analysis

A total of 9,048 and 11,322 MPXV genomes were retrieved from the GISAID (Shu & McCauley, 2017) and NCBI GenBank (https://www.ncbi.nlm.nih.gov/nucleotide/) databases (Sayers et al., 2024), respectively. Redundant MPXV genome sequences were removed at the national level using CD-HIT (v4.8.1) (Li & Godzik, 2006), with the default threshold parameter set to 0.99. Only sequences evaluated by NextClade (Aksamentov et al., 2021) as having a mediocre or good overall quality control status were retained; those with excessive ambiguous bases or large missing regions were excluded. After filtering, 157 high-quality, non-redundant genomes remained (Table S1). Sequences were aligned with MAFFT (v7.505) (Katoh & Standley, 2013), and a maximum-likelihood phylogenetic tree was built using IQ-TREE (v2.1.4) (Minh et al., 2020) with ModelFinder-selected parameters (Kalyaanamoorthy et al., 2017) and 1,000 bootstrap replicates. The tree was visualized using iTOL (v7) (Letunic & Bork, 2007).

### Identification of core and non-core genes

Core genes were defined as those present in all MPXV genomes, while non-core genes appeared only in some strains. Non-core genes were further classified as dispensable (found in ≥2 genomes) or unique (found in only one genome). Open reading frames (ORF) were predicted using ORFfinder (v0.4.3) (Sayers et al., 2011), which also provided nucleotide and protein sequences. Genes shorter than 180 bp were excluded from further analysis. Gene clustering was conducted using OrthoFinder (v2.5.5) (Emms & Kelly, 2019), resulting in 405 orthogroups, including 278 multi-gene and 127 singleton clusters (Table S2). Protein annotation was performed using BLAST (v2.12.0+) (Camacho et al., 2009) against MPXV proteins from the NCBI RefSeq database (retrieved December 3, 2024), and functional classifications were derived from Hernaez’s work (Van Vliet et al., 2009; Moss, 2013; Molteni et al., 2023; Hernaez & Alcamí, 2024) (Table S3).

### Pan-genome inference

To estimate the size of the MPXV pan-genome, we performed random resampling of 1 to 157 strains (in increments of 1), with 1,000 replicates at each sampling level. For each sample size, the mean total gene count was calculated and used to fit a power regression model, characterizing the pan-genome dynamics of MPXV. The pan-genome size was fitted using the Heaps’ law-based power-law model, *y* = A_pan_ × *x*^Bpan^ + C_pan_, where *y* represents pan-genome size, *x* represents the number of genomes, and A_pan_, B_pan_, and C_pan_ are the fitted parameters. Here, B_pan_ represents the parameter used to characterize pan-genome openness. A value of 0 <  B_pan_ < 1 indicates an open pan-genome, whereas B_pan_ < 0 or B_pan_ > 1 indicates a closed pan-genome. The fitted model showed a high goodness-of-fit (R^2^ > 0.99), and the estimated *B*_pan_ value was 0.681 (95%CI [0.664–0.697]), supporting an open pan-genome structure of MPXV.

### Ancestral state reconstruction of genes of MPXV in the phylogenetic tree

Ancestral state reconstruction of MPXV genes was conducted using Brito’s method (Brito & Pinney, 2020; Zhu et al., 2021). A gene count matrix for 157 MPXV genomes, along with the corresponding phylogenetic tree, was input into the Trace Ancestral Character function in Mesquite (v3.81) (Maddison & Maddison, 2023). Ancestral gene counts at internal nodes were inferred using linear parsimony, providing a parsimonious model of gene repertoire evolution in MPXV.

### Gene variation analysis

To analyze variation patterns in core and non-core MPXV genes, point mutations and indels were quantified across 278 gene groups. Genomes were first aligned using MAFFT, and mutations were identified *via* custom Python scripts. Point mutations are genetic variations characterized by the substitution of a single nucleotide base within a DNA sequence. Insertions and deletions (Indels) refer to the addition or removal of one or more nucleotides in a genomic sequence, which can lead to significant changes in gene length and function. To evaluate variation across orthologous gene groups, point mutations and indels were identified from multiple-sequence alignments (MSAs). For each orthologous group, the total number of mutated nucleotide positions (V) was calculated by summing all single-nucleotide substitutions and the total number of altered nucleotides within indels (*e.g.*, a deletion of three consecutive nucleotides was counted as three mutations). To enable unbiased comparisons across genes of different sizes and sample coverages, the mutation ratio (R) was normalized by both gene length and sequence count using the formula: R = V/(L × N), where L represents the alignment length in base pairs (excluding gap-only positions) and N represents the total number of sequences analyzed in the orthologous group.

### Calculate selection pressure

Termination codons were first removed from the MAFFT-aligned nucleotide sequences. A phylogenetic tree was then constructed using IQ-TREE. Based on the phylogenetic tree and the cleaned sequence alignments, the ratio of nonsynonymous to synonymous substitutions (dN/dS, or *ω*) was estimated using the Single-Likelihood Ancestor Counting (SLAC) method (Kosakovsky Pond & Frost, 2005), as implemented in HyPhy v2.5.59 (Pond, Frost & Muse, 2005). The SLAC method uses codon sequence alignments and the corresponding phylogenetic relationships to reconstruct ancestral sequences under a maximum-likelihood framework and combines this information with substitution counting to estimate synonymous and nonsynonymous substitution rates and their corresponding *ω* ratio (dN/dS). Following the classical dN/dS framework, orthologous genes were classified according to their gene-wide *ω* values. Specifically, genes with *ω* > 1 were considered to exhibit evolutionary patterns consistent with positive selection, genes with *ω* = 1 were considered to exhibit neutral evolutionary patterns, and genes with *ω* < 1 were considered to exhibit evolutionary patterns consistent with purifying selection (Yang et al., 2000). Genes for which dN/dS could not be reliably estimated were excluded from the selection classification.

### Detection of homologous recombination events

Given that recombination can affect the accuracy of selection pressure analysis, we conducted a recombination analysis on MPXV genomes in this study to rule out this interference. Recombination analysis of aligned MPXV genomes was performed using RDP5 (Martin et al., 2021), implementing nine built-in algorithms (RDP, GENECONV, Bootscan, Maxchi, Chimaera, SiScan, PhylPro, LARD, and 3Seq) under default settings. Signals with *p* < 0.05 were recorded. For each detected event, RDP identified the recombinant strain, potential major/minor parental lineages, crossover regions, and supporting methods. To ensure reliability, only events supported by at least three methods were retained for subsequent analyses (Table S4).

### Functional enrichment analysis

The protein–protein interaction (PPI) data between MPXV and humans were downloaded from the P-HIPSTer database (http://phipster.org/) (Lasso et al., 2019) and the dataset from Zhu et al.’s research. Gene Ontology (GO) and Kyoto Encyclopedia of Genes and Genomes (KEGG) pathway enrichment analyses were performed on human genes using the *enrichGO()* and *enrichKEGG()* functions in the “clusterProfiler” package (Xu et al., 2024). Enrichments with adjusted *p*-values below 0.05 were considered statistically significant.

### Statistical analysis

All statistical analyses were conducted using R (v4.5.0; R Core Team, 2025). The Wilcoxon rank-sum and signed-rank tests, both implemented *via* the *wilcox.test()* function, were used for independent and paired sample comparisons, respectively. Spearman correlation coefficients were computed with the *cor.test()* function using the “spearman” method. A chi-square test was conducted using the *chisq.test()* function in R, comparing the proportions of genes under positive selection between core and non-core gene groups.

## Results

### Identification of MPXV genotypes

A total of 157 MPXV genomes were obtained from the NCBI nucleotide and GISAID databases, representing over 37 countries, primarily in Africa, and Americas, with samples spanning from 1968 to 2024 (Fig. 1A). Most sequences were collected in 2022. Genome sizes ranged from 184,351 to 199,469 bp, averaging 196,532 bp, with isolate COP-58 (AY753185.1) being the largest and isolate 22MPX0036V (PQ221506.1) the smallest. The dataset included four genotypes: Ia (22), Ib (2), IIa (5), and IIb (128), with IIb being the most prevalent and Ib the least (Fig. 1B). This diverse dataset supports robust evolutionary analysis.

**Figure 1:**
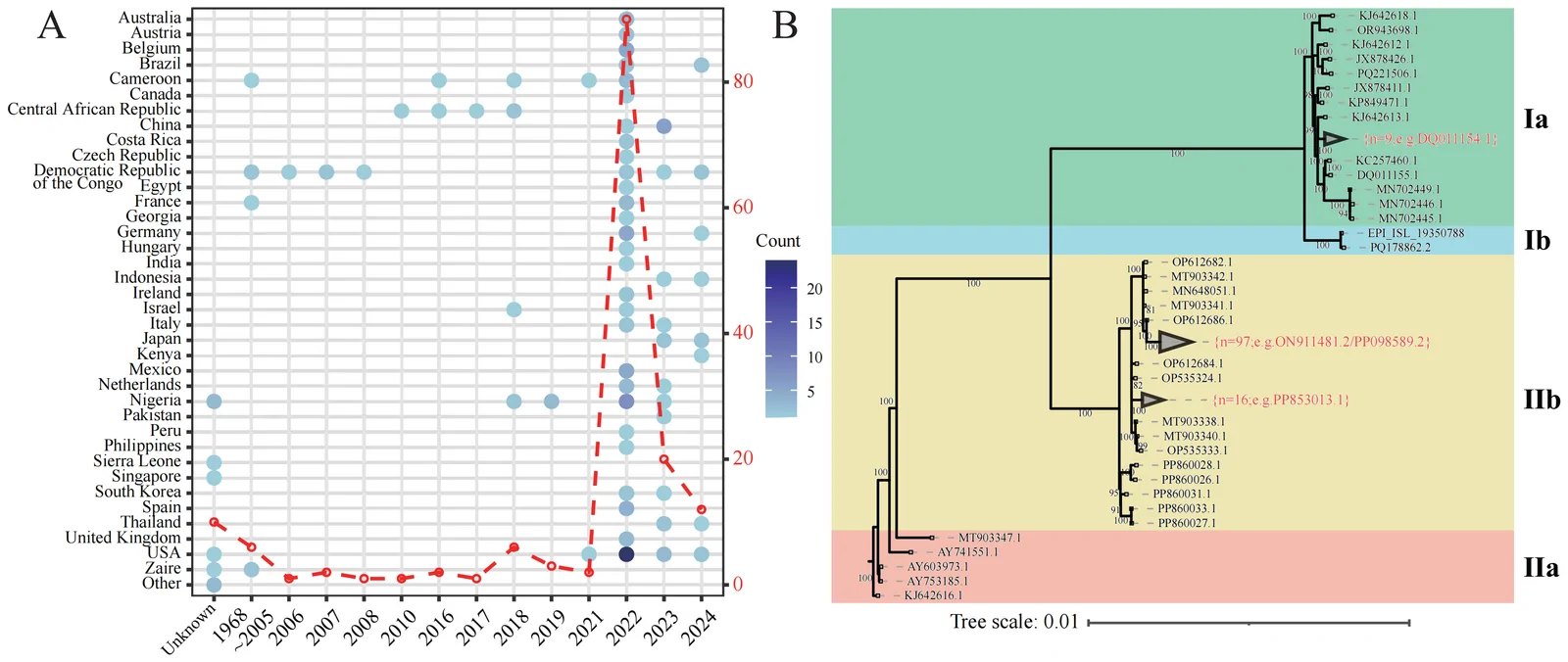
Overview of MPXV genome dataset. (A) The number of MPXV genomes collected over time and across different countries. Color gradients indicate the numerical values. (B) Classification of MPXV genomes by genotype, highlighting the major lineages included in the study. Genotypes are color-coded as indicated.

### Pan-genomic analysis of MPXV genes

A total of 183 core and 222 non-core gene clusters were identified in MPXV, including 95 dispensable and 127 unique genes among the non-core gene (Fig. 2A). The core genes primarily encode proteins involved in immune evasion or adaptation proteins and structural proteins, while the non-core genes mainly encode proteins related to immune evasion or adaptation proteins and unknown proteins (Fig. S1). A power-law regression model (*y* = 5.27xˆ0.681+238.19) accurately described the increasing number of non-core gene clusters as more genomes were analyzed, indicating MPXV’s adaptability through pan-genome expansion (Fig. 2B). Conversely, an exponential model (*y* = 36.26eˆ(−0.018x)+182.78) captured the declining trend in core gene clusters with additional genomes, reflecting the genome’s dynamic and diverse nature (Fig. 2B).

**Figure 2:**
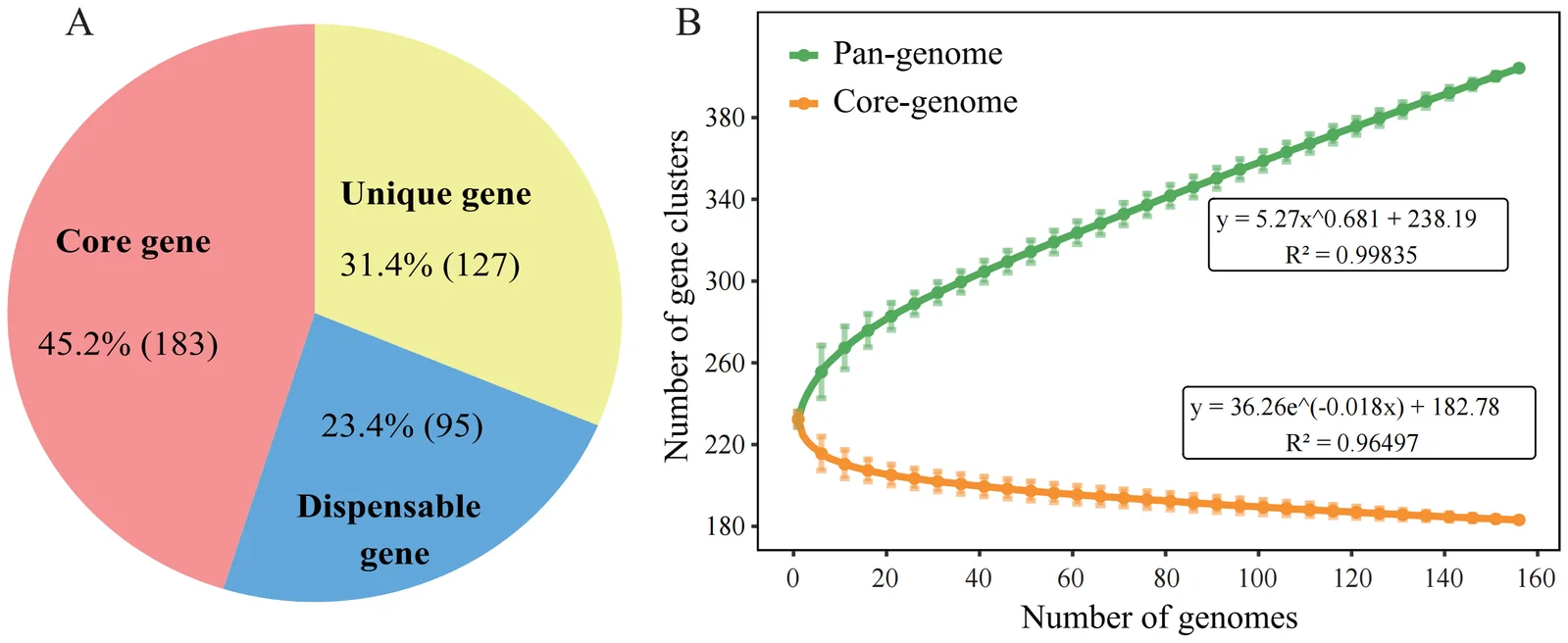
Overview of MPXV pan-genome architecture. (A) The pie chart illustrates the proportions of core (pink), dispensable (blue), and strain-unique genes (yellow). (B) Pan-genome curve of MPXV based on protein-coding genes, showing the accumulation of total, core, and non-core genes as additional genomes are analyzed. The core genes are indicated by the orange line, while non-core genes are shown in green.

To assess genotype-specific patterns, we analyzed the pan-genomes of MPXV genotypes I and II (Fig. S2). Genotype I contained 214 core and 67 non-core gene clusters (including 30 unique genes), while genotype II had 198 core and 148 non-core clusters (with 101 unique genes). In both genotypes, the power-law regression parameter Bpan (the exponent of *x*) >0.5 (Fig. S2), indicating an open pan-genome structure. As more genomes were analyzed, core gene cluster curves showed a converging trend, suggesting that gene content within each genotype is relatively conserved during evolution.

### Dynamics of MPXV gene evolution

To further explore the evolutionary dynamics of MPXV genes, we analyzed dynamic changes in the number of genes across genomes from 1968 to 2024 (Fig. 3). While most MPXV genomes contain only a single copy of each gene, approximately 4.5% of the genome contains duplicated genes. Ancestral gene states were reconstructed on the phylogenetic tree, revealing two major types of gene dynamics: gene gains/losses (GLs) and copy number variations (CNVs) (Fig. 3). In total, 336 GLs (228 gains, 108 losses) and 53 CNVs (11 increases, 42 decreases) were identified. Notably, 24.3% of genes experienced multiple changes, with 11 genes undergoing more than 5 events. For instance, gene OPG015 (OG0000003) showed 9 CNVs and 1 GLs. Most (98.2%) number variations occurred within non-core gene clusters. Branch-specific analysis revealed that 74.1% of CNVs and 64.2% of GLs occurred in terminal branches, highlighting recent and lineage-specific changes. Besides, genes exhibiting number changes during evolution are primarily involved in immune evasion, as well as in biological functions that remain to be fully elucidated (Fig. S3).

**Figure 3:**
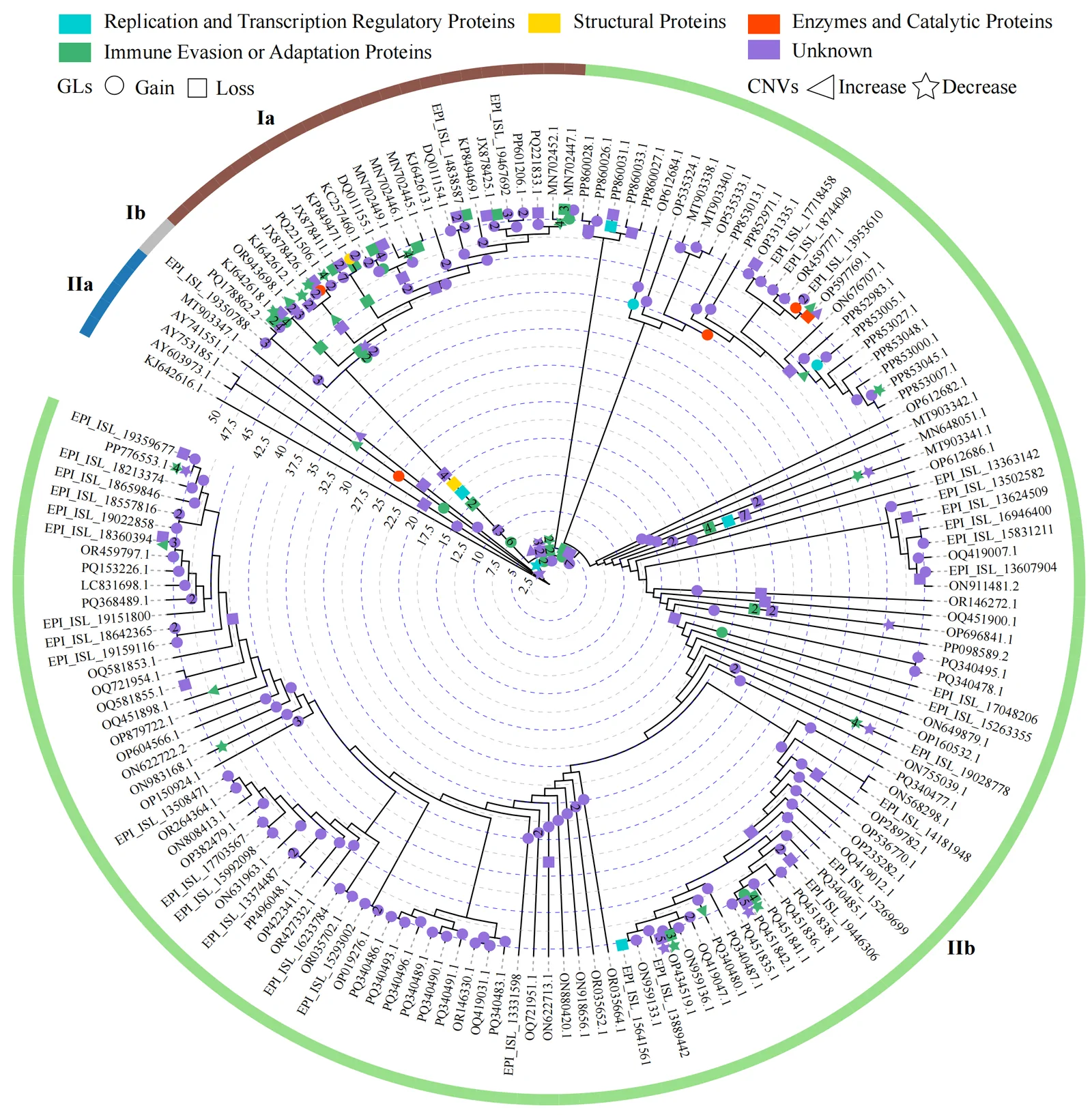
Gene number variation across the MPXV phylogeny. GLs and CNVs events in MPXV were mapped onto the branches of the phylogenetic tree using colored shape. Functional categories of the affected genes are indicated by colors, as defined in the legend (directly above). Values above symbols indicate repeated events of the same type and function at each respective node. The phylogenetic tree was constructed based on full-genome sequences using IQ-TRE E (see ‘Materials and Methods’). MPXV genotypes are labeled using bold numbers adjacent to corresponding branches.

Certain gene cluster dynamics exhibited genotype-specific patterns. For example, loss of the Unknown (OG0000260) gene was exclusive to genotype Ia, loss of the KM543_gp176 (OG0000263) to genotype IIa, and loss of the OPG174 (OG0000255) to genotype IIb (Fig. 3). Genotype-based analysis showed that 52.3% of CNVs and 3.8% of GLs occurred among viruses within the same genotype. Notably, Clade IIb viruses exhibited one CNV and 116 GLs. Some strains underwent extensive gene changes, such as strain MPXV-M5320_M15_Bayelsa which experienced two CNVs and 14 GLs (Table S5).

### Comparative variation patterns in core *vs.* accessory genes of MPXV

MPXV gene sequences exhibit extensive variation, primarily through point mutations and insertions/deletions (indels). Comparative analysis revealed that non-core genes contain significantly more indels than core genes (average: 0.038 *vs.* 0.006, *p* < 0.01) (Fig. 4A). Although the median indel ratio is zero for both groups, the higher indel ratio in non-core genes arises from a right-skewed distribution and the presence of extreme positive outliers (Q3:0.028 *vs.* 0.002). Despite this, both gene categories primarily undergo variation *via* point mutations (Figs. 4B & 4C), indicating that point mutations are the dominant mechanism of genetic change in MPXV. Furthermore, a significant positive correlation between point mutations and indels in both core and non-core genes suggests that these two types of genetic variation may accumulate concurrently during viral evolution (Fig. 4D). Notably, this association is especially pronounced in immune evasion–related core genes (Fig. S4), suggesting that these genes may experience relatively greater genetic variation during viral evolution.

**Figure 4:**
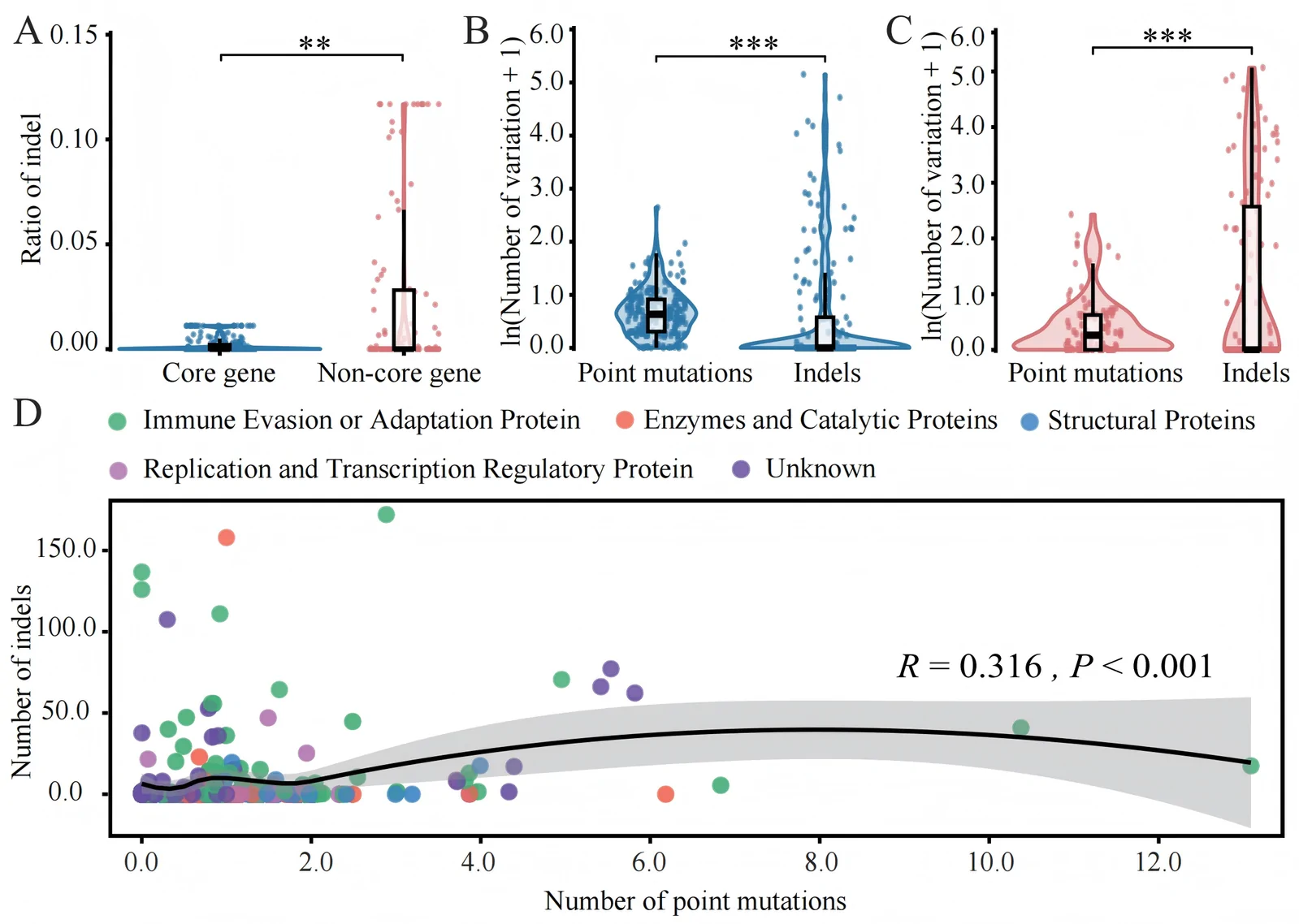
Variation patterns in MPXV genes. (A) Indel ratio comparison in core *vs.* non-core genes. Statistical tests include all raw data; the plot displays the lower 90% of the distribution to improve visualization. (B) Numbers of point mutations and indels in core gene sets. (C) Numbers of point mutations and indels in non-core gene sets. (D) Correlation between the numbers of point mutations and indels across all MPXV genes. Statistical significance was assessed using the Wilcoxon rank-sum test in (A), paired Wilcoxon signed-rank tests in (B) and (C), and Spearman’s rank correlation test in (D). ^∗^* p* < 0.05, ^∗∗^*p* < 0.01, ^∗∗∗^*p* < 0.001.

### Positive-selection signals in non-core genes of MPXV

Selection pressures on 278 MPXV genes were estimated using the HyPhy tool based on dN/dS. A dN/dS ratio >1 indicates positive selection, <1 purifying selection, and = 1 neutral evolution. The majority of genes showed dN/dS ratios below 1, consistent with predominant purifying selection. Among core genes, 42 showed dN/dS ratios >1 and 141 showed dN/dS ratios <1, with none showing neutral evolution (Fig. 5A). Similarly, among non-core genes, 39 showed dN/dS ratios >1 and 56 showed dN/dS ratios <1, with no neutral cases observed (Fig. 5A). In this dataset, the proportion of non-core genes showing dN/dS ratios >1 was higher than that of core genes (41% *vs.* 23%, *p* < 0.01; Fig. 5A), suggesting a higher frequency of positive-selection signals among non-core genes. These signals were observed particularly among genes associated with immune evasion or genes of unknown function.

**Figure 5:**
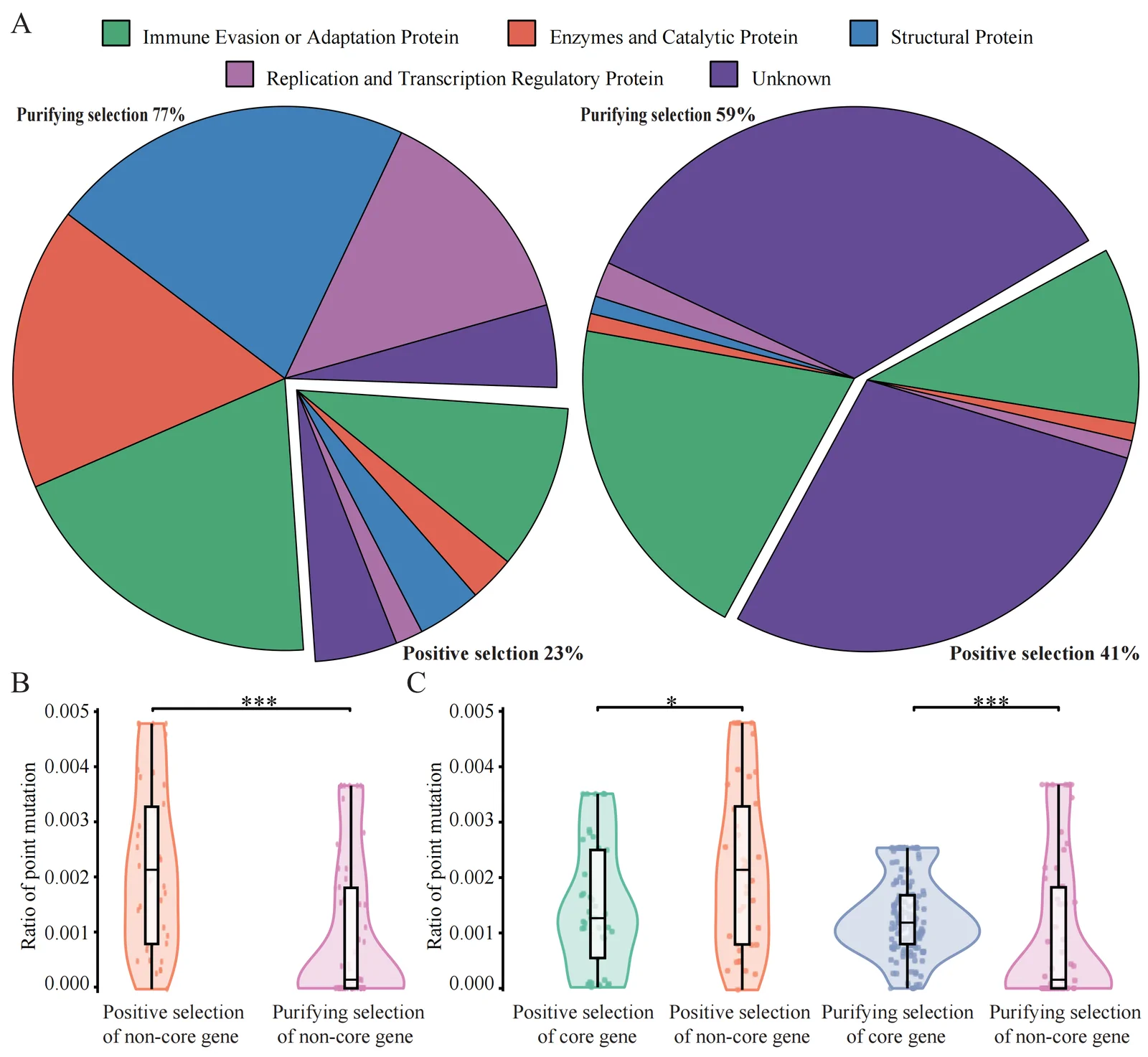
Selective pressure and its relationship with genomic variation in MPXV genes. (A) Ratio of core and non-core genes under positive or purifying selection across different functional categories. (B) Point mutation ratios of non-core genes under different selective pressures (positive *vs.* purifying selection). (C) Comparison of point mutation ratios between core and non-core genes under positive and purifying selection. (B) and (C) illustrate the lower 90% of data points for visualization; however, all statistical tests were conducted using the full raw dataset. Significance was assessed using the Chi-square test (A) and Wilcoxon rank-sum tests (B, C). ^∗^ *p* < 0.05, ^∗∗^ *p* < 0.01, ^∗∗∗^ *p* < 0.001.

To investigate evolutionary dynamics, point mutations in MPXV core and non-core genes showing dN/dS patterns consistent with positive or purifying selection were analyzed. Specifically, non-core genes showing positive-selection signals had higher point mutation ratios than those showing purifying-selection signals (median 0.0022 *vs.* 0.0002; *p* < 0.001; Fig. 5B). Among genes showing positive-selection signals, non-core genes exhibited a higher point mutation ratio than core genes (median: 0.0022 *vs.* 0.0013, *p* < 0.05; Fig. 5C). In contrast, among genes showing purifying-selection signals, core genes showed a higher point mutation ratio than non-core genes (median: 0.0012 *vs.* 0.0002, *p* < 0.001; Fig. 5C). These observations may reflect differences in the evolutionary dynamics of core and non-core genes.

### Functional analysis of human proteins interacting with core and non-core MPXV proteins

To distinguish the functional roles of MPXV core *vs.* non-core genes, we performed GO enrichment and KEGG analyses of human proteins interacting with each group.

**Figure 6:**
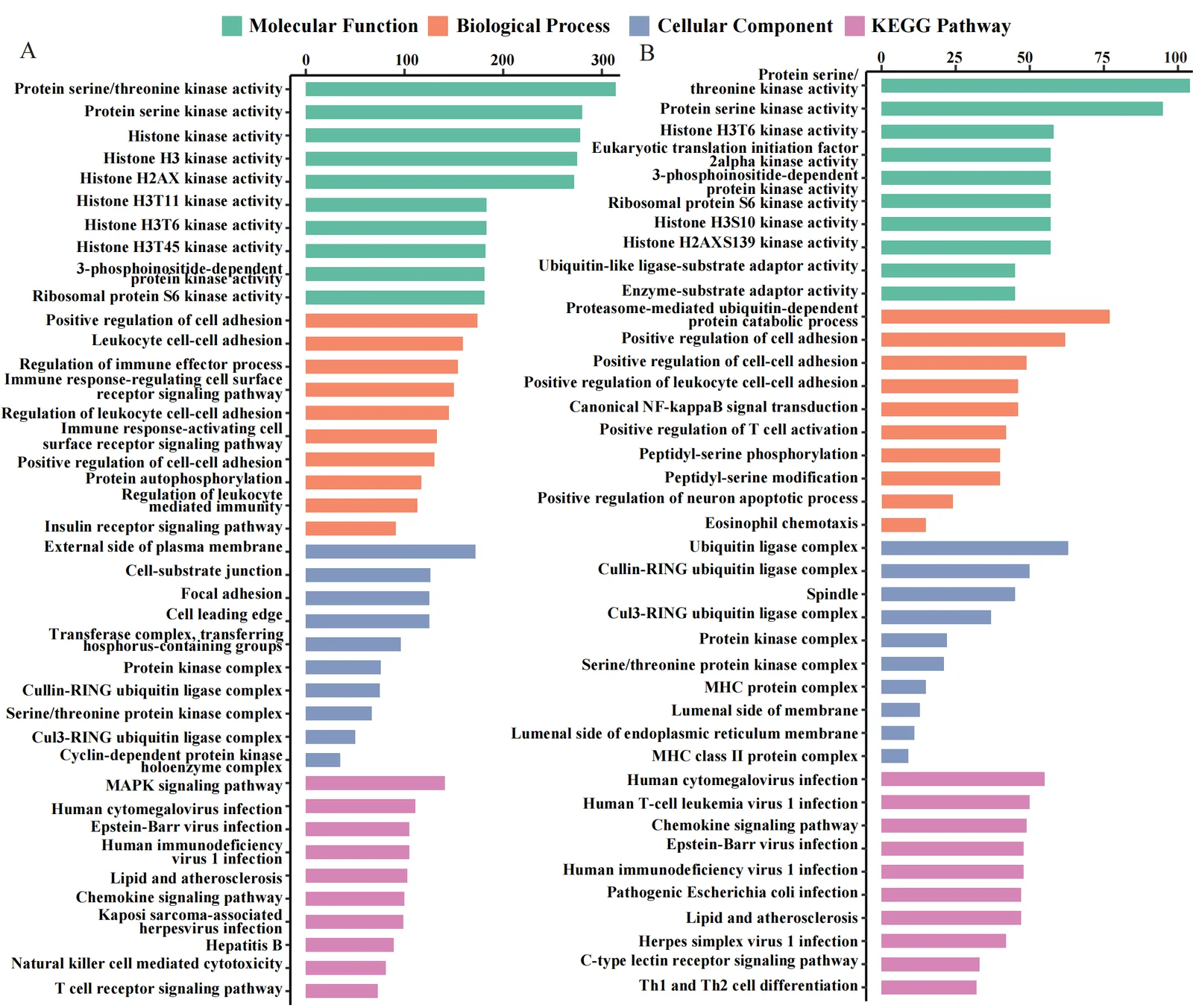
Functional analysis of the human proteins interacting with MPXV proteins. (A–B) The top 10 enriched GO terms and KEGG pathways for human proteins interacting with MPXV proteins encoded by (A) core genes and (B) non-core genes. Enrichments span the domains of biological processes, cellular components, molecular functions, and KEGG pathways.

Notably, the proteins encoded by core genes interact with a substantially larger number of human proteins than do those encoded by non-core genes (Fig. S5). For core genes (Fig. 6A), human interactors were mainly involved in immune functions—like activating leukocytes and regulating immune responses through surface receptors. In the GO molecular function category, the most enriched terms were protein serine/threonine kinase activity and protein serine kinase activity, along with multiple histone kinase activities (*e.g.*, H3 and H2AX). This suggests that MPXV may target host phosphorylation processes and epigenetic regulation. Enrichment of 3-phosphoinositide-dependent protein kinase and ribosomal S6 kinase activity further indicates potential modulation of signaling and protein translation. In the biological process category, enriched terms were mainly related to immune responses and cell adhesion, including positive regulation of cell adhesion, leukocyte cell–cell adhesion, and immune receptor signaling pathways. These findings imply that MPXV may interfere with immune cell communication and signaling. Processes such as protein autophosphorylation also suggest alterations in intracellular signaling. For cellular component, enriched terms included plasma membrane, focal adhesion, and protein kinase complexes, indicating that MPXV-host interactions are closely linked to membrane-associated structures and signaling complexes. The enrichment of Cullin-RING ubiquitin ligase complexes highlights the role of ubiquitination. KEGG analysis revealed significant enrichment in immune and viral infection pathways, including MAPK signaling, chemokine signaling, and pathways associated with viruses such as cytomegalovirus and Epstein–Barr virus.

The functional profile of human proteins interacting with non-core MPXV proteins revealed a strong focus on immune regulation and viral response (Fig. 6B). Key biological processes included T cell activation, leukocyte adhesion, and signaling pathways like NF-*κ*B and apoptosis control. These proteins were linked to cellular structures involved in degradation and signaling, such as ubiquitin ligase and kinase complexes, as well as immune-related MHC components. Their molecular roles involved a variety of kinases—especially serine/threonine-specific and histone kinases—and ubiquitin-related adaptors. KEGG analysis showed a clear emphasis on viral infection pathways (EBV, HIV-1, CMV, HTLV-1, HSV), immune signaling mechanisms, and inflammation-related pathways such as atherosclerosis.

## Discussion

Despite its utility in virology,pan-genome analysis has seen limited application in MPXV research (Labrie et al., 2013; Hick et al., 2017). In this study, we applied a pan-genomic framework to 157 MPXV genomes, identifying 183 core and 222 non-core genes, and systematically examined their differences in evolutionary dynamics, mutational patterns, and functional roles. Our findings revealed that, non-core genes are more frequently gained or lost, exhibit a higher ratio of indels, and show stronger signals of positive selection than core genes. These results suggest that MPXV preserves essential viral functions through a highly conserved core genome, while exploiting the evolutionary plasticity of non-core genes to adapt to changing environments and potentially broaden its host range. Our findings underscore the distinct evolutionary roles of core and non-core genes in MPXV. Furthermore, the presence of an open pan-genome indicates ongoing genetic diversification and suggests a continued potential for the emergence of novel genetic variants. Collectively, these insights enhance our understanding of MPXV evolution and provide a valuable foundation for the development of future antiviral and vaccine strategies.

Pan-genome analysis of MPXV revealed distinct core and non-core gene clusters, indicating that the MPXV genome is in an “open” state, continuing to acquire new genes as additional strains are sequenced. Core genes primarily support viral particle assembly, genome protection, and replication, whereas non-core genes are mainly associated with immune evasion, host adaptation, and cross-species transmission. Identifying core genes highlights the fundamental components of the viral replication cycle, providing a basis for targeted antiviral development (Diab et al., 2018; Andersen et al., 2020). Meanwhile, systematic characterization of non-core genes uncovers adaptive hotspots, immune-evasion epitopes, and potential zoonotic determinants, offering valuable insights for the design of broad-spectrum antivirals and next-generation vaccines (Geraghty, Aliota & Bonnac, 2021). Furthermore, while examining the genome, we also found many genes with unknown functions and several that are unique to specific genotypes (Figs. S1 and S6). These genotype-specific genes may explain why some versions of the virus spread more easily or cause more severe illness than others (Schwartz, 2024; Young et al., 2024).

The open pangenome characteristics exhibited by MPXV are closely linked to its repeat-mediated genomic plasticity. Mechanisms such as short tandem repeat (STR) instability, homologous recombination, and the ‘genomic accordion’ model can drive rapid gene gain/loss and copy number variation (Desingu, Nagarajan & Sundaresan, 2023; Monzón et al., 2024). Although repeat elements were not explicitly analyzed in this study, the observed enrichment of structural variation in non-core regions is consistent with these processes (Monzón et al., 2024). Such dynamics, particularly in terminal genomic regions, may facilitate rapid adaptation and contribute to the evolutionary flexibility of MPXV.

Consistent with patterns observed in other giant viruses (Desnues et al., 2012), MPXV exhibits extensive gene copy number variation, leading to expansion and contraction of its genome throughout evolution. Such extensive gene copy number variation likely enhances viral adaptability by enabling rapid functional diversification and reversible genome expansion, allowing MPXV to respond efficiently to fluctuating host environments and immune pressures while maintaining overall genomic stability (Monzón et al., 2024). These structural fluctuations may contribute to phenotypic heterogeneity, including changes in virulence and host interaction (Desingu, Nagarajan & Sundaresan, 2023). The present study aligns with findings in large DNA viruses and underscores the evolutionary plasticity of the MPXV genome (Kejnovsky & Jedlicka, 2022). Such variability may hinder the efficacy of universal vaccines and antivirals (Vemula et al., 2017). Elucidating the mechanistic and functional implications of these gene-level dynamics is essential for advancing targeted therapeutic development.

MPXV exhibits distinct mutation patterns: non-core genes accumulate more indels. Indels in non-core genes promote rapid adaptation—particularly in host interaction and immune evasion—whereas point mutations in core genes fine-tune essential viral functions (Wang et al., 2025; Rodrigues et al., 2022). Notably, a strong association between indels and point mutations across both gene types suggests synergistic effects that enhance genetic diversity and evolutionary potential. These patterns have key public health implications: variability and positive selection in non-core genes may influence transmissibility and host range, while conserved core genes remain promising targets for antivirals and vaccines (Alam et al., 2020). The open pan-genome further indicates ongoing diversification, emphasizing the need for continuous genomic surveillance (Otieno et al., 2025).

This study has four main limitations. First, limited availability of genotype Ib genomes restricted comparative analyses, although the dataset spans 1968–2024 and diverse geographic regions. Second, many newly identified genes—particularly non-core—lack experimental validation and functional annotation. Third, despite potential recombination bias in dN/dS estimates, the low recombination rate suggests minimal impact on positive selection results. Fourth, repeat elements (*e.g.*, STRs) were not explicitly analyzed, despite their role in genome plasticity. Future integration of pan-genome, repeat, and recombination analyses would improve understanding of MPXV evolution. Although incomplete functional characterization limits insight into virus–host interactions, our findings still provide valuable evolutionary and pathogenic clues.

## Conclusions

This study shows that MPXV has an open pan-genome with substantial gene variation, reflecting its evolutionary flexibility. Core and non-core genes display distinct mutation patterns and selective pressures, with core genes primarily targeting host immune pathways and non-core genes linked to infection-related proteins. These findings advance understanding of MPXV evolution and host interactions, informing surveillance and therapeutic strategies.

## Supplemental Information

10.7717/peerj.21846/supp-1 Supplemental Information 1 Supplemental figures and tables

10.7717/peerj.21846/supp-2 Supplemental Information 2 Raw data

## Additional Information and Declarations

### Data Availability

The following information was supplied regarding data availability: The raw data is available in the Supplemental Files. The raw sequence data are available at NCBI, GISAID, and P-HIPSTer; the complete lists of accessions are available in Tables S1 and S2.

## References

[ref-1] Agarwal P, Shukla N, Bhatia A, Mahfooz S, Narayan J (2024). Comparative genome analysis reveals driving forces behind monkeypox virus evolution and sheds light on the role of ATC trinucleotide motif. Virus Evolution.

[ref-2] Aksamentov I, Roemer C, Hodcroft E, Neher R (2021). Nextclade: clade assignment, mutation calling and quality control for viral genomes. Journal of Open Source Software.

[ref-3] Alam I, Kamau AA, Kulmanov M, Jaremko Ł, Arold ST, Pain A, Gojobori T, Duarte CM (2020). Functional pangenome analysis shows key features of E protein are preserved in SARS and SARS-CoV-2. Frontiers in Cellular and Infection Microbiology.

[ref-4] Andersen PI, Ianevski A, Lysvand H, Vitkauskiene A, Oksenych V, Bjørås M, Telling K, Lutsar I, Dumpis U, Irie Y, Tenson T, Kantele A, Kainov DE (2020). Discovery and development of safe-in-man broad-spectrum antiviral agents. International Journal of Infectious Diseases.

[ref-5] Andriyanov P, Zhurilov P, Menshikova A, Tutrina A, Yashin I, Kashina D (2024). Large-scale genomic analysis of elizabethkingia anophelis. BMC Genomics.

[ref-6] Brito AF, Pinney JW (2020). The evolution of protein domain repertoires: shedding light on the origins of the Herpesviridae family. Virus Evolution.

[ref-7] Bruno G, Buccoliero GB (2023). Antivirals against monkeypox (mpox) in humans: an updated narrative review. Life.

[ref-8] Camacho C, Coulouris G, Avagyan V, Ma N, Papadopoulos J, Bealer K, Madden TL (2009). BLAST+: architecture and applications. BMC Bioinformatics.

[ref-9] Desingu PA, Nagarajan K, Sundaresan NR (2023). Unique tandem repeats in the inverted terminal repeat regions of monkeypox viruses. Microbiology Spectrum.

[ref-10] Desnues C, La Scola B, Yutin N, Fournous G, Robert C, Azza S, Jardot P, Monteil S, Campocasso A, Koonin EV, Raoult D (2012). Provirophages and transpovirons as the diverse mobilome of giant viruses. Proceedings of the National Academy of Sciences of the United States of America.

[ref-11] Diab A, Foca A, Zoulim F, Durantel D, Andrisani O (2018). The diverse functions of the hepatitis B core/capsid protein (HBc) in the viral life cycle: implications for the development of HBc-targeting antivirals. Antiviral Research.

[ref-12] Elde NC, Child SJ, Eickbush MT, Kitzman JO, Rogers KS, Shendure J, Geballe AP, Malik HS (2012). Poxviruses deploy genomic accordions to adapt rapidly against host antiviral defenses. Cell.

[ref-13] Emms DM, Kelly S (2019). OrthoFinder: phylogenetic orthology inference for comparative genomics. Genome Biology.

[ref-14] Geraghty R, Aliota M, Bonnac L (2021). Broad-spectrum antiviral strategies and nucleoside analogues. Viruses.

[ref-15] Gong Q, Wang C, Chuai X, Chiu S (2022). Monkeypox virus: a re-emergent threat to humans. Virologica Sinica.

[ref-16] Hernaez B, Alcamí A (2024). Poxvirus immune evasion. Annual Review of Immunology.

[ref-17] Hick PM, Subramaniam K, Thompson PM, Waltzek TB, Becker JA, Whittington RJ (2017). Molecular epidemiology of epizootic haematopoietic necrosis virus (EHNV). Virology.

[ref-18] Isidro J, Borges V, Pinto M, Sobral D, Santos JD, Nunes A, Mixão V, Ferreira R, Santos D, Duarte S, Vieira L, Borrego MJ, Núncio S, De Carvalho IL, Pelerito A, Cordeiro R, Gomes JP (2022). Phylogenomic characterization and signs of microevolution in the 2022 multi-country outbreak of monkeypox virus. Nature Medicine.

[ref-19] Kalyaanamoorthy S, Minh BQ, Wong TKF, Von Haeseler A, Jermiin LS (2017). ModelFinder: fast model selection for accurate phylogenetic estimates. Nature Methods.

[ref-20] Karagoz A, Tombuloglu H, Alsaeed M, Tombuloglu G, Al Rubaish AA, Mahmoud A, Smajlović S, Ćordić S, Rabaan AA, Alsuhaimi E (2023). Monkeypox (mpox) virus: classification, origin, transmission, genome organization, antiviral drugs, and molecular diagnosis. Journal of Infection and Public Health.

[ref-21] Katoh K, Standley DM (2013). MAFFT multiple sequence alignment software version 7: improvements in performance and usability. Molecular Biology and Evolution.

[ref-22] Kejnovsky E, Jedlicka P (2022). Nucleic acids movement and its relation to genome dynamics of repetitive DNA: is cellular and intercellular movement of DNA and RNA molecules related to the evolutionary dynamic genome components?. BioEssays.

[ref-23] Kosakovsky Pond SL, Frost SDW (2005). Not so different after all: a comparison of methods for detecting amino acid sites under selection. Molecular Biology and Evolution.

[ref-24] Labrie SJ, Frois-Moniz K, Osburne MS, Kelly L, Roggensack SE, Sullivan MB, Gearin G, Zeng Q, Fitzgerald M, Henn MR, Chisholm SW (2013). Genomes of marine cyanopodoviruses reveal multiple origins of diversity. Environmental Microbiology.

[ref-25] Lasso G, Mayer SV, Winkelmann ER, Chu T, Elliot O, Patino-Galindo JA, Park K, Rabadan R, Honig B, Shapira SD (2019). A structure-informed atlas of human-virus interactions. Cell.

[ref-26] Letunic I, Bork P (2007). Interactive tree of life (iTOL): an online tool for phylogenetic tree display and annotation. Bioinformatics.

[ref-27] Li W, Godzik A (2006). Cd-hit: a fast program for clustering and comparing large sets of protein or nucleotide sequences. Bioinformatics.

[ref-28] Lum F-M, Torres-Ruesta A, Tay MZ, Lin RTP, Lye DC, Rénia L, Ng LFP (2022). Monkeypox: disease epidemiology, host immunity and clinical interventions. Nature Reviews Immunology.

[ref-29] Luna N, Muñoz M, Bonilla-Aldana DK, Patiño LH, Kasminskaya Y, Paniz-Mondolfi A, Ramírez JD (2023). Monkeypox virus (MPXV) genomics: a mutational and phylogenomic analyses of B.1 lineages. Travel Medicine and Infectious Disease.

[ref-30] Luna N, Ramírez AL, Muñoz M, Ballesteros N, Patiño LH, Castañeda SA, Bonilla-Aldana DK, Paniz-Mondolfi A, Ramírez JD (2022). Phylogenomic analysis of the monkeypox virus (MPXV) 2022 outbreak: emergence of a novel viral lineage?. Travel Medicine and Infectious Disease.

[ref-31] Maddison WP, Maddison DR (2023). https://www.mesquiteproject.org.

[ref-32] Martin DP, Varsani A, Roumagnac P, Botha G, Maslamoney S, Schwab T, Kelz Z, Kumar V, B Murrell (2021). RDP5: a computer program for analyzing recombination in, and removing signals of recombination from, nucleotide sequence datasets. Virus Evolution.

[ref-33] Masirika LM, Udahemuka JC, Schuele L, Nieuwenhuijse DF, Ndishimye P, Boter M, Mbiribindi JB, Kacita C, Lang T, Gortázar C, Musabyimana JP, Otani S, Aarestrup FM, Siangoli FB, Oude Munnink BB, Koopmans M (2025). Epidemiological and genomic evolution of the ongoing outbreak of clade ib mpox virus in the eastern democratic Republic of the Congo. Nature Medicine.

[ref-34] Medini D, Donati C, Tettelin H, Masignani V, Rappuoli R (2005). The microbial pan-genome. Current Opinion in Genetics & Development.

[ref-35] Minh BQ, Schmidt HA, Chernomor O, Schrempf D, Woodhams MD, Von Haeseler A, Lanfear R (2020). IQ-TREE 2: new models and efficient methods for phylogenetic inference in the genomic era. Molecular Biology and Evolution.

[ref-36] Molteni C, Forni D, Cagliani R, Arrigoni F, Pozzoli U, De Gioia L, Sironi M (2023). Selective events at individual sites underlie the evolution of monkeypox virus clades. Virus Evolution.

[ref-37] Monzón S, Varona S, Negredo A, Vidal-Freire S, Patiño Galindo JA, Ferressini-Gerpe N, Zaballos A, Orviz E, Ayerdi O, Muñoz Gómez A, Delgado-Iribarren A, Estrada V, García C, Molero F, Sánchez-Mora P, Torres M, Vázquez A, Galán J-C, Torres I, CausseDel Río M, Merino-Diaz L, López M, Galar A, Cardeñoso L, Gutiérrez A, Loras C, Escribano I, Alvarez-Argüelles ME, Del Río L, Simón M, Meléndez MA, Camacho J, Herrero L, Jiménez P, Navarro-Rico ML, Jado I, Giannetti E, Kuhn JH, Sanchez-Lockhart M, Di Paola N, Kugelman JR, Guerra S, García-Sastre A, Cuesta I, Sánchez-Seco MP, Palacios G (2024). Monkeypox virus genomic accordion strategies. Nature Communications.

[ref-38] Moss B (2013). Poxvirus DNA replication. Cold Spring Harbor Perspectives in Biology.

[ref-39] Otieno JR, Ruis C, Onoja AB, Kuppalli K, Hoxha A, Nitsche A, Brinkmann A, Michel J, Mbala-Kingebeni P, Mukadi-Bamuleka D, Osman MM, Hussein H, Raja MA, Fotsing R, Herring BL, Keita M, Rico JM, Gresh L, Barakat A, Katawera V, Nahapetyan K, Naidoo D, Floto RA, Cunningham J, Van Kerkhove MD, Lewis RF, Subissi L (2025). Global genomic surveillance of monkeypox virus. Nature Medicine.

[ref-40] Parker S, Buller RM (2013). A review of experimental and natural infections of animals with monkeypox virus between 1958 and 2012. Future Virology.

[ref-41] Pond SLK, Frost SDW, Muse SV (2005). HyPhy: hypothesis testing using phylogenies. Bioinformatics.

[ref-42] R Core Team (2025). R: A language and environment for statistical computing. https://www.R-project.org/.

[ref-43] Reynolds MG, Davidson WB, Curns AT, Conover CS, Huhn G, Davis JP, Wegner M, Croft DR, Newman A, Obiesie NN, Hansen GR, Hays PL, Pontones P, Beard B, Teclaw R, Howell JF, Braden Z, Holman RC, Karem KL, Damon IK (2007). Spectrum of infection and risk factors for human monkeypox, United States, 2003. Emerging Infectious Diseases.

[ref-44] Rodrigues RAL, Queiroz VF, Ghosh J, Dunigan DD, Van Etten JL (2022). Functional genomic analyses reveal an open pan-genome for the chloroviruses and a potential for genetic innovation in new isolates. Journal of Virology.

[ref-45] Saeed AF, Wang R, Wang S (2016). Microsatellites in pursuit of microbial genome evolution. Frontiers in Microbiology.

[ref-46] Sahay RR, Shete AM, Khurana J, Jain R, Chenayil S, Yadav S, Majumdar T, Rani S, Vijay N, Patil DY, Kannan Sabarinath PS, Aravind J, Gavari S, Gawande P, Pandita P, Singh S, Anitha PM, Pulloor NK, Kizhakkekarammel P, Manjusree S, Siba S, Balakrishnan A, Raji TK, Gupta N, Yadav PD (2026). The evolving landscape of Mpox: clinical and genomic insights into Clade Ib emergence in India. Virology.

[ref-47] Sayers EW, Barrett T, Benson DA, Bolton E, Bryant SH, Canese K, Chetvernin V, Church DM, Di Cuccio M, Federhen S, Feolo M, Fingerman IM, Geer LY, Helmberg W, Kapustin Y, Landsman D, Lipman DJ, Lu Z, Madden TL, Madej T, Maglott DR, Marchler-Bauer A, Miller V, Mizrachi I, Ostell J, Panchenko A, Phan L, Pruitt KD, Schuler GD, Sequeira E, Sherry ST, Shumway M, Sirotkin K, Slotta D, Souvorov A, Starchenko G, Tatusova TA, Wagner L, Wang Y, Wilbur WJ, Yaschenko E, Ye J (2011). Database resources of the national center for biotechnology information. Nucleic Acids Research.

[ref-48] Sayers EW, Cavanaugh M, Clark K, Pruitt KD, Sherry ST, Yankie L, Karsch-Mizrachi I (2024). GenBank 2024 update. Nucleic Acids Research.

[ref-49] Schwartz DA (2024). High rates of miscarriage and stillbirth among pregnant women with clade I mpox (monkeypox) are confirmed during 2023–2024 DR congo outbreak in South Kivu province. Viruses.

[ref-50] Shu Y, McCauley J (2017). GISAID: global initiative on sharing all influenza data –from vision to reality. Eurosurveillance.

[ref-51] Sun Y, Nie W, Tian D, Ye Q (2024). Human monkeypox virus: epidemiologic review and research progress in diagnosis and treatment. Journal of Clinical Virology.

[ref-52] Van Vliet K, Mohamed MR, Zhang L, Villa NY, Werden SJ, Liu J, McFadden G (2009). Poxvirus proteomics and virus-host protein interactions. Microbiology and Molecular Biology Reviews.

[ref-53] Vemula SV, Sayedahmed EE, Sambhara S, Mittal SK (2017). Vaccine approaches conferring cross-protection against influenza viruses. Expert Review of Vaccines.

[ref-54] Wang L, Luo Y, Zhao Y, Gao GF, Bi Y, Qiu H (2020). Comparative genomic analysis reveals an ‘open’ pan-genome of african swine fever virus. Transboundary and Emerging Diseases.

[ref-55] Wang W, Liu Q, Zhao Q, Zhu Z, Wen W, Zhang Z, Li X (2025). Homologous recombination and evolutionary arms race drive the adaptive evolution of african swine fever virus. Veterinary Research.

[ref-56] Wei F, Peng Z, Jin Z, Wang J, Xu X, Zhang X, Xu J, Ren Z, Bai Y, Wang X, Lu B, Wang Z, Xu J, Huang S (2022). Study and prediction of the 2022 global monkeypox epidemic. Journal of Biosafety and Biosecurity.

[ref-57] Xu S, Hu E, Cai Y, Xie Z, Luo X, Zhan L, Tang W, Wang Q, Liu B, Wang R, Xie W, Wu T, Xie L, Yu G (2024). Using clusterProfiler to characterize multiomics data. Nature Protocols.

[ref-58] Yang Z, Nielsen R, Goldman N, Pedersen A-MK (2000). Codon-substitution models for heterogeneous selection pressure at amino acid sites. Genetics.

[ref-59] Young B, Seifert SN, Lawson C, Koehler H (2024). Exploring the genomic basis of mpox virus-host transmission and pathogenesis. mSphere.

[ref-60] Yu Z, Zou X, Deng Z, Zhao M, Gu C, Fu L, Xiao W, He M, He L, Yang Q, Liang S, Wen C, Lü M (2024). Genome analysis of the mpox (formerly monkeypox) virus and characterization of core/variable regions. Genomics.

[ref-61] Zhang J, Ye H, Li H, Ma K, Qiu W, Chen Y, Qiu Z, Li B, Jia W, Liang Z, Liao M, Qi W (2020). Evolution and antigenic drift of influenza a (H7N9) viruses, China, 2017–2019. Emerging Infectious Diseases.

[ref-62] Zhu Z, Chen H, Liu L, Cao Y, Jiang T, Zou Y, Peng Y (2021). Classification and characterization of multigene family proteins of african swine fever viruses. Briefings in Bioinformatics.

[ref-63] Zhu Z, Sun Q, Xu Y, Niu Y, Yang F, Feng S (2025). Novel drug targets for monkeypox: from viral to host proteins. Infectious Medicine.

[ref-64] Zinnah MA, Uddin MB, Hasan T, Das S, Khatun F, Hasan MH, Udonsom R, Rahman MM, Ashour HM (2024). The re-emergence of mpox: old illness, modern challenges. Biomedicines.

