# Supplementary material for: Pan-genome insights into the evolutionary dynamics and functional diversification of genes in monkeypox virus
Source: PeerJ. 2026 Oct 26;14:e21846. doi: 10.7717/peerj.21846 (PMC13718445; doi:10.7717/peerj.21846)
Supplement: Supplementary file 1 [file peerj-14-21846-s001.docx]

**Supplementary Materials for**

**Pan-genome insights into the evolutionary dynamics and functional diversification of genes in monkeypox virus**

**Fig.S1.** Distribution of the number of MPXV genes categorized by major functional classes.Pie chart: Proportion of all MPXV genes by functional category. Bar chart: Number of each functional category within core, dispensable, and unique genes. The combined visualization highlights that while essential functions—such as replication and structural integrity—are predominantly localized within the core genome, the dispensable and unique gene sets are largely composed of proteins with unknown functions or those involved in immune evasion.

**Fig.S2**. Pan-genomic analysis of monkeypox virus subtypes. A pie chart depicting the core and non-core (dispensable and strain-unique) MPXV of genotype (A)I and (C)II gene. The diagram depicts the core (pink), dispensable (blue), and strain-unique (yellow) gene number. The pan-genome analysis of MPXV of genotype (B)I and (D II based on protein-coding genes.

**Fig.S3**. Functional distribution of MPXV genes with number changes (copy number variations or gain/loss events). Different colors represent different functional categories of the genes.

**Fig.S4.** The relationship among the mutation patterns of evasion-related genes in monkeypox virus. The relationship between point mutations and insertions/deletions in immune evasion-related genes within (A) core and (B) non-core genes of monkeypox virus.

**Fig.S5.** Distribution of the number of human proteins interacting with proteins encoded by core and non-core genes. The red area represents human proteins that interact exclusively with core virus proteins (n = 1,864). The blue area represents human proteins that interact exclusively with non-core virus proteins (n = 90). The overlapping purple region indicates the subset of human proteins that interact with both core and non-core virus proteins (n = 653).

**Fig.S6.** Distribution of gene cluster numbers among various MPXV genotypes. The intersection of the four ellipses represents the number of gene clusters shared by genotypes IIa, Ia, Ib, and IIb. While the majority of clusters (210) are universal to all genotypes, lineage IIb exhibits the highest number of unique gene clusters (109). This variation in cluster distribution suggests a complex evolutionary history characterized by both high conservation of core functions and lineage-specific gene gain or loss.

**Table S1.** The MPXV strains used in this study. The table provides comprehensive details for each isolate, including its accession number, clade designation, Qc.overallStatus, country of origin, and collection date.

| **Accession number** | **clade** | **Qc.overallStatus** | **Country** | **Collection date** |
| --- | --- | --- | --- | --- |
| OP597769.1 | IIb | good | Egypt | 2022-09-26 |
| PP496048.1 | IIb | good | Philippines | 2022-08-16 |
| EPI_ISL_19359677 | IIb | good | Brazil | 2024-02-27 |
| EPI_ISL_13374487 | IIb | good | Hungary | 2022-06-01 |
| EPI_ISL_19446306 | IIb | good | Brazil | 2024-09-11 |
| EPI_ISL_17048206 | IIb | good | Brazil | 2022-08-02 |
| EPI_ISL_13508471 | IIb | good | Brazil | 2022-06-24 |
| OP160532.1 | IIb | good | Netherlands | 2022-06 |
| PQ178862.2 | Ib | mediocre | Kenya | 2024-07-25 |
| EPI_ISL_15269699 | IIb | good | Netherlands | 2022-05-24 |
| EPI_ISL_18659846 | IIb | good | Netherlands | 2023-11-07 |
| EPI_ISL_15641561 | IIb | good | Netherlands | 2022-06-10 |
| OR146272.1 | IIb | good | Ireland | 2022-07-11 |
| OR146330.1 | IIb | good | Ireland | 2022-07-12 |
| KJ642612.1 | Ia | mediocre | Zaire | 1986 |
| MN702445.1 | Ia | mediocre | Central African Republic | 2017-02-06 |
| DQ011155.1 | Ia | mediocre | Zaire | Unknown |
| KJ642613.1 | Ia | mediocre | Zaire | 1970 |
| MN702446.1 | Ia | mediocre | Central African Republic | 2018-04-14 |
| MN702447.1 | Ia | mediocre | Central African Republic | 2018-03-20 |
| OP382479.1 | IIb | good | France | 2022-07-01 |
| ON622722.2 | IIb | good | France | 2022-05-22 |
| ON755039.1 | IIb | good | France | 2022-05-19 |
| MN702449.1 | Ia | mediocre | Central African Republic | 2016-01-02 |
| OQ721951.1 | IIb | good | China | 2023-03 |
| OQ721954.1 | IIb | good | China | 2023-03 |
| OQ581853.1 | IIb | good | China | 2023-02 |
| OQ581855.1 | IIb | good | China | 2023-03 |
| ON918656.1 | IIb | good | China | 2022-06 |
| AY741551.1 | IIa | mediocre | Sierra Leone | Unknown |
| MN702452.1 | Ia | mediocre | Central African Republic | 2010-08-10 |
| KJ642616.1 | IIa | mediocre | France | 1968 |
| EPI_ISL_18213374 | IIb | good | China | 2023-06-24 |
| EPI_ISL_18360394 | IIb | mediocre | China | 2023-09-22 |
| PQ451835.1 | IIb | good | USA | 2023-10 |
| PQ451836.1 | IIb | good | USA | 2024-01 |
| PQ451841.1 | IIb | good | USA | 2024-01 |
| PQ451838.1 | IIb | good | USA | 2023-12 |
| PQ340477.1 | IIb | good | USA | 2022-10 |
| PQ340480.1 | IIb | good | USA | 2022-10 |
| PQ340478.1 | IIb | good | USA | 2022-10 |
| PQ340485.1 | IIb | good | USA | 2022-10 |
| PQ340486.1 | IIb | good | USA | 2022-10 |
| PQ340483.1 | IIb | good | USA | 2022-10 |
| PQ340487.1 | IIb | good | USA | 2022-10 |
| PQ340489.1 | IIb | good | USA | 2022-10 |
| PQ340490.1 | IIb | good | USA | 2022-10 |
| PQ340491.1 | IIb | good | USA | 2022-10 |
| PQ340493.1 | IIb | good | USA | 2022-10 |
| PQ340496.1 | IIb | good | USA | 2022-10 |
| PQ340495.1 | IIb | good | USA | 2022-10 |
| OR427332.1 | IIb | good | USA | 2022-09 |
| OQ419047.1 | IIb | good | USA | 2022-08 |
| OQ419007.1 | IIb | good | USA | 2022-07 |
| OQ419012.1 | IIb | good | USA | 2022-09 |
| OP879722.1 | IIb | good | USA | 2022-07-26 |
| OP604566.1 | IIb | good | USA | 2022-08-28 |
| OQ419031.1 | IIb | good | USA | 2022-07 |
| OP150924.1 | IIb | good | USA | 2022-06 |
| ON959133.1 | IIb | good | USA | 2022-05 |
| ON959136.1 | IIb | good | USA | 2022-06 |
| ON676707.1 | IIb | good | USA | 2021-07 |
| MT903347.1 | IIa | mediocre | USA | Unknown |
| PQ221506.1 | Ia | mediocre | Democratic Republic of the Congo | 2022-02-05 |
| PQ221833.1 | Ia | mediocre | Democratic Republic of the Congo | 2024-02-15 |
| OP434519.1 | IIb | good | USA | 2022-07 |
| PP601206.1 | Ia | mediocre | Democratic Republic of the Congo | 2024-01-20 |
| KP849469.1 | Ia | mediocre | Democratic Republic of the Congo | 2008 |
| KP849471.1 | Ia | mediocre | Democratic Republic of the Congo | 1985 |
| JX878411.1 | Ia | mediocre | Democratic Republic of the Congo | 2006-11-30 |
| JX878425.1 | Ia | mediocre | Democratic Republic of the Congo | 2007-04-20 |
| JX878426.1 | Ia | mediocre | Democratic Republic of the Congo | 2007-05-27 |
| LC831698.1 | IIb | good | Japan | 2024-03-03 |
| EPI_ISL_18557816 | IIb | good | Japan | 2023-09-22 |
| EPI_ISL_19467692 | Ia | mediocre | Democratic Republic of the Congo | 2023 |
| EPI_ISL_19151800 | IIb | good | Japan | 2024-01-31 |
| KC257460.1 | Ia | mediocre | Democratic Republic of the Congo | 1985 |
| EPI_ISL_15293002 | IIb | good | Spain | 2022 |
| EPI_ISL_16233784 | IIb | good | Spain | 2022-08-17 |
| EPI_ISL_14838587 | Ia | mediocre | Democratic Republic of the Congo | 2022 |
| MT903342.1 | IIb | good | Singapore | Unknown |
| EPI_ISL_19022858 | IIb | good | Japan | 2023-05-08 |
| EPI_ISL_13331598 | IIb | good | Georgia | 2022-06-04 |
| EPI_ISL_16946400 | IIb | good | Costa Rica | 2022-10-18 |
| EPI_ISL_13363142 | IIb | good | Spain | 2022-06-10 |
| EPI_ISL_15831211 | IIb | good | Costa Rica | 2022-10-31 |
| EPI_ISL_14181948 | IIb | good | Spain | 2022 |
| EPI_ISL_19028778 | IIb | good | Canada | 2022-09 |
| EPI_ISL_13953610 | IIb | good | India | 2022-07-13 |
| ON911481.2 | IIb | good | Mexico | 2022-06-28 |
| EPI_ISL_17703567 | IIb | good | Mexico | 2022-08-31 |
| EPI_ISL_15992098 | IIb | good | Mexico | 2022-08-11 |
| EPI_ISL_13624509 | IIb | good | Mexico | 2022-05-27 |
| EPI_ISL_13607904 | IIb | good | Mexico | 2022-06-28 |
| OP289782.1 | IIb | good | Peru | 2022-06-25 |
| ON649879.1 | IIb | good | Israel | 2022-05-20 |
| OR035664.1 | IIb | good | Belgium | 2022-06-13 |
| MN648051.1 | IIb | good | Israel | 2018-10-04 |
| OR035652.1 | IIb | good | Belgium | 2022-08-02 |
| OR035702.1 | IIb | good | Belgium | 2022-05-30 |
| ON880420.1 | IIb | good | Belgium | 2022-05-27 |
| ON622713.1 | IIb | good | Belgium | 2022-05-22 |
| PP852983.1 | IIb | good | Nigeria | 2022-10-05 |
| PQ451842.1 | IIb | good | USA | 2023-11 |
| PP852971.1 | IIb | good | Nigeria | 2023-05-31 |
| PP853000.1 | IIb | good | Nigeria | 2022-10-24 |
| PP853005.1 | IIb | good | Nigeria | 2022-10-01 |
| PP853007.1 | IIb | good | Nigeria | 2022-10-24 |
| PP853013.1 | IIb | good | Nigeria | 2022-09-27 |
| PP853027.1 | IIb | good | Nigeria | 2022-10-24 |
| PP853045.1 | IIb | good | Nigeria | 2022-10-24 |
| OP535324.1 | IIb | good | Nigeria | 2018-02 |
| OP535333.1 | IIb | good | Nigeria | 2018-01 |
| PP853048.1 | IIb | good | Nigeria | 2022-09-12 |
| OP612682.1 | IIb | good | Nigeria | 2019-04-20 |
| OP612684.1 | IIb | mediocre | Nigeria | 2019-05 |
| OP612686.1 | IIb | good | Nigeria | 2019-06-26 |
| MT903338.1 | IIb | good | Nigeria | Unknown |
| MT903340.1 | IIb | good | Nigeria | Unknown |
| PP776553.1 | IIb | good | Germany | 2024-04 |
| OQ451898.1 | IIb | good | Germany | 2022-09 |
| OQ451900.1 | IIb | good | Germany | 2022-10 |
| ON568298.1 | IIb | good | Germany | 2022-05-19 |
| EPI_ISL_13889442 | IIb | good | Germany | 2022-05-25 |
| EPI_ISL_17718458 | IIb | good | Pakistan | 2023-05-20 |
| OP696841.1 | IIb | good | Germany | 2022-07 |
| PQ368489.1 | IIb | good | Thailand | 2023-07-03 |
| PQ153226.1 | IIb | good | Thailand | 2023-09 |
| OP235282.1 | IIb | good | Australia | 2022 |
| ON631963.1 | IIb | good | Australia | 2022-05 |
| OR264364.1 | IIb | good | Australia | 2022-08-01 |
| ON983168.1 | IIb | good | Czech Republic | 2022-04-28 |
| PP098589.2 | IIb | good | Italy | 2023-05 |
| EPI_ISL_15263355 | IIb | good | Italy | 2022-08-08 |
| EPI_ISL_13502582 | IIb | good | Italy | 2022-06-13 |
| EPI_ISL_19350788 | Ib | mediocre | Thailand | 2024-08-16 |
| EPI_ISL_18642365 | IIb | good | Indonesia | 2023-11-16 |
| AY753185.1 | IIa | mediocre | Unknown | Unknown |
| EPI_ISL_19159116 | IIb | good | Indonesia | 2024-01-22 |
| AY603973.1 | IIa | mediocre | Unknown | Unknown |
| DQ011154.1 | Ia | mediocre | Unknown | Unknown |
| OP422341.1 | IIb | good | Austria | 2022-08-01 |
| OP019276.1 | IIb | good | Austria | 2022-06-21 |
| PP860026.1 | IIb | good | Cameroon | 2018-05-10 |
| PP860028.1 | IIb | good | Cameroon | 2022-10-27 |
| PP860027.1 | IIb | good | Cameroon | 2022-10-13 |
| PP860031.1 | IIb | good | Cameroon | 2021-07-22 |
| PP860033.1 | IIb | mediocre | Cameroon | 2022 |
| OR459797.1 | IIb | good | South Korea | 2023-04-22 |
| OR459777.1 | IIb | good | South Korea | 2022-11-22 |
| EPI_ISL_18744049 | IIb | good | South Korea | 2022-12-19 |
| OR943698.1 | Ia | mediocre | Cameroon | 2016-08 |
| OP536770.1 | IIb | good | United Kingdom | 2022-08 |
| MT903341.1 | IIb | good | Nigeria | Unknown |
| OP331335.1 | IIb | good | United Kingdom | 2022-06-03 |
| ON808413.1 | IIb | good | United Kingdom | 2022-05-15 |
| KJ642618.1 | Ia | mediocre | Cameroon | 1990 |

**Table S2**. **Grouping of MPXV genes based on sequence homology.**

| **Grouping of MPXV genes** | Genes assigned to each group |
| --- | --- |
| OG0000000 | AY603973.1:194647-196419;AY603973.1:4547-2775;AY741551.1:194208-195980;AY741551.1:4547-2775;AY753185.1:194915-196687;AY753185.1:4553-2781;DQ011154.1:192456-194219;DQ011154.1:4738-2975;DQ011155.1:192316-194079;DQ011155.1:4650-2887;EPI_ISL_13331598:192800-194572;EPI_ISL_13331598:4628-2856;EPI_ISL_13363142:192559-194331;EPI_ISL_13363142:4621-2849;EPI_ISL_13374487:192614-194386;EPI_ISL_13374487:4636-2864;EPI_ISL_13502582:192608-194380;EPI_ISL_13502582:4621-2849;EPI_ISL_13508471:192659-194431;EPI_ISL_13508471:4713-2941;EPI_ISL_13607904:192971-194743;EPI_ISL_13607904:4612-2840;EPI_ISL_13624509:192393-194165;EPI_ISL_13624509:4620-2848;EPI_ISL_13889442:192700-194472;EPI_ISL_13889442:4731-2959;EPI_ISL_13953610:192507-194279;EPI_ISL_13953610:4612-2840;EPI_ISL_14181948:192641-194413;EPI_ISL_14181948:4757-2985;EPI_ISL_14838587:192215-193978;EPI_ISL_14838587:4635-2872;EPI_ISL_15263355:192303-194075;EPI_ISL_15263355:4609-2837;EPI_ISL_15269699:192527-194299;EPI_ISL_15269699:4581-2809;EPI_ISL_15293002:193250-195022;EPI_ISL_15293002:4726-2954;EPI_ISL_15641561:191940-193712;EPI_ISL_15641561:4570-2798;EPI_ISL_15831211:192579-194351;EPI_ISL_15831211:4619-2847;EPI_ISL_15992098:192558-194330;EPI_ISL_15992098:4634-2862;EPI_ISL_16233784:192579-194351;EPI_ISL_16233784:4630-2858;EPI_ISL_16946400:192512-194284;EPI_ISL_16946400:4619-2847;EPI_ISL_17048206:192184-193956;EPI_ISL_17048206:4269-2497;EPI_ISL_17703567:192580-194352;EPI_ISL_17703567:4634-2862;EPI_ISL_17718458:192557-194329;EPI_ISL_17718458:4612-2840;EPI_ISL_18213374:192585-194357;EPI_ISL_18213374:4609-2837;EPI_ISL_18360394:191254-193026;EPI_ISL_18360394:4270-2498;EPI_ISL_18557816:192532-194304;EPI_ISL_18557816:4610-2838;EPI_ISL_18642365:192591-194363;EPI_ISL_18642365:4612-2840;EPI_ISL_18659846:192861-194633;EPI_ISL_18659846:4582-2810;EPI_ISL_18744049:192586-194358;EPI_ISL_18744049:4614-2842;EPI_ISL_19022858:192530-194302;EPI_ISL_19022858:4609-2837;EPI_ISL_19028778:4526-2754;EPI_ISL_19151800:191439-193211;EPI_ISL_19151800:4610-2838;EPI_ISL_19159116:192569-194341;EPI_ISL_19159116:4612-2840;EPI_ISL_19350788:190890-192653;EPI_ISL_19350788:4644-2881;EPI_ISL_19359677:192584-194356;EPI_ISL_19359677:4609-2837;EPI_ISL_19446306:192568-194340;EPI_ISL_19446306:4604-2832;EPI_ISL_19467692:192559-194322;EPI_ISL_19467692:4794-3031;JX878411.1:193298-195061;JX878411.1:5579-3816;JX878425.1:192128-193891;JX878425.1:5018-3255;JX878426.1:192163-193926;JX878426.1:4583-2820;KC257460.1:192070-193833;KC257460.1:4416-2653;KJ642612.1:190265-192028;KJ642612.1:2922-1159;KJ642613.1:192020-193783;KJ642613.1:4421-2658;KJ642616.1:194331-196103;KJ642616.1:4409-2637;KJ642618.1:189778-191541;KJ642618.1:4479-2821;KP849469.1:192604-194367;KP849469.1:4842-3079;KP849471.1:192423-194186;KP849471.1:4824-3061;LC831698.1:192567-194339;LC831698.1:4610-2838;MN648051.1:193007-194779;MN648051.1:4409-2637;MN702445.1:185663-187426;MN702446.1:4471-2708;MN702447.1:185224-186987;MN702449.1:185710-187473;MN702452.1:4490-2727;MT903338.1:193147-194919;MT903338.1:4411-2639;MT903340.1:192596-194368;MT903340.1:4612-2840;MT903341.1:180593-182365;MT903341.1:3158-2943;MT903342.1:192697-194469;MT903342.1:4611-2839;MT903347.1:194052-195824;MT903347.1:4727-2955;ON568298.1:192642-194414;ON568298.1:4758-2986;ON622713.1:193301-195073;ON622713.1:4715-2943;ON622722.2:192504-194276;ON622722.2:4615-2843;ON631963.1:192807-194579;ON631963.1:4635-2863;ON649879.1:192307-194079;ON649879.1:4445-2673;ON676707.1:192599-194371;ON676707.1:4665-2893;ON755039.1:192318-194090;ON755039.1:3853-2081;ON808413.1:192515-194287;ON808413.1:4615-2843;ON880420.1:193255-195027;ON880420.1:4714-2942;ON911481.2:192800-194572;ON911481.2:4608-2836;ON918656.1:192592-194364;ON918656.1:4647-2875;ON959133.1:193162-194934;ON959133.1:4608-2836;ON959136.1:193184-194956;ON959136.1:4609-2837;ON983168.1:191728-193500;ON983168.1:3183-1411;OP019276.1:192583-194355;OP019276.1:4609-2837;OP150924.1:193155-194927;OP150924.1:4622-2850;OP160532.1:192433-194205;OP160532.1:4575-2803;OP235282.1:192583-194355;OP235282.1:4609-2837;OP289782.1:192587-194359;OP289782.1:4612-2840;OP331335.1:192579-194351;OP331335.1:4629-2857;OP382479.1:192560-194332;OP382479.1:4616-2844;OP422341.1:192575-194347;OP422341.1:4629-2857;OP434519.1:185684-187456;OP434519.1:4608-2836;OP535324.1:192623-194395;OP535324.1:4610-2838;OP535333.1:192678-194450;OP535333.1:4719-2947;OP536770.1:192586-194358;OP536770.1:4611-2839;OP597769.1:192567-194339;OP597769.1:4633-2861;OP604566.1:192592-194364;OP604566.1:4612-2840;OP612682.1:192636-194408;OP612682.1:4410-2638;OP612684.1:193101-194873;OP612684.1:4234-2462;OP612686.1:192722-194494;OP612686.1:4410-2638;OP696841.1:189806-191578;OP696841.1:4609-2837;OP879722.1:191746-193518;OP879722.1:3835-2063;OQ419007.1:191803-193575;OQ419007.1:3845-2073;OQ419012.1:191777-193549;OQ419012.1:3837-2065;OQ419031.1:191781-193553;OQ419031.1:3842-2070;OQ419047.1:191783-193555;OQ419047.1:3845-2073;OQ451898.1:191617-193389;OQ451898.1:4610-2838;OQ451900.1:192531-194303;OQ451900.1:4610-2838;OQ581853.1:192529-194301;OQ581853.1:4636-2864;OQ581855.1:192638-194410;OQ581855.1:4717-2945;OQ721951.1:192607-194379;OQ721951.1:4634-2862;OQ721954.1:192539-194311;OQ721954.1:4607-2835;OR035652.1:192919-194691;OR035652.1:4559-2787;OR035664.1:192865-194637;OR035664.1:4559-2787;OR035702.1:192882-194654;OR035702.1:4559-2787;OR146272.1:192521-194293;OR146272.1:4575-2803;OR146330.1:192599-194371;OR146330.1:4620-2848;OR264364.1:192576-194348;OR264364.1:4630-2858;OR427332.1:192576-194348;OR427332.1:4630-2858;OR459777.1:192562-194334;OR459777.1:4614-2842;OR459797.1:192574-194346;OR459797.1:4629-2857;OR943698.1:192126-193889;OR943698.1:4601-2838;PP098589.2:192585-194357;PP098589.2:4611-2839;PP496048.1:192503-194275;PP496048.1:4615-2843;PP601206.1:192170-193933;PP601206.1:4635-2872;PP776553.1:4560-2788;PP852971.1:192574-194346;PP852971.1:4609-2837;PP852983.1:192516-194288;PP852983.1:4614-2842;PP853000.1:192465-194237;PP853000.1:4621-2849;PP853005.1:192539-194311;PP853005.1:4616-2844;PP853007.1:192490-194262;PP853007.1:4621-2849;PP853013.1:192519-194291;PP853013.1:4622-2850;PP853027.1:192512-194284;PP853027.1:4621-2849;PP853045.1:192277-194049;PP853045.1:4625-2853;PP853048.1:192456-194228;PP853048.1:4621-2849;PP860026.1:192491-194263;PP860026.1:4618-2846;PP860027.1:192455-194227;PP860027.1:4617-2845;PP860028.1:192460-194232;PP860028.1:4617-2845;PP860031.1:192453-194225;PP860031.1:4630-2858;PP860033.1:192463-194235;PP860033.1:4617-2845;PQ153226.1:192566-194338;PQ153226.1:4609-2837;PQ178862.2:190655-192418;PQ178862.2:4665-2902;PQ221833.1:192147-193910;PQ221833.1:4634-2871;PQ340477.1:192672-194444;PQ340477.1:4576-2804;PQ340478.1:192565-194337;PQ340478.1:4577-2805;PQ340480.1:192735-194507;PQ340480.1:4608-2836;PQ340483.1:192707-194479;PQ340483.1:4576-2804;PQ340485.1:192730-194502;PQ340485.1:4578-2806;PQ340486.1:192805-194577;PQ340486.1:4577-2805;PQ340487.1:192881-194653;PQ340487.1:4570-2798;PQ340489.1:192781-194553;PQ340489.1:4577-2805;PQ340490.1:192591-194363;PQ340490.1:4576-2804;PQ340491.1:192898-194670;PQ340491.1:4570-2798;PQ340493.1:192685-194457;PQ340493.1:4578-2806;PQ340495.1:192778-194550;PQ340495.1:4576-2804;PQ340496.1:192876-194648;PQ340496.1:4609-2837;PQ368489.1:192599-194371;PQ368489.1:4612-2840;PQ451835.1:192888-194660;PQ451835.1:4572-2800;PQ451836.1:192538-194310;PQ451836.1:4632-2860;PQ451838.1:192823-194595;PQ451838.1:4603-2831;PQ451841.1:192702-194474;PQ451841.1:4595-2823;PQ451842.1:181255-183027;PQ451842.1:4595-2823 |
| OG0000001 | AY603973.1:196507-197556;AY603973.1:2687-1638;AY741551.1:196070-197119;AY741551.1:2685-1636;AY753185.1:196775-197824;AY753185.1:2693-1644;DQ011154.1:194309-195355;DQ011154.1:2885-1839;DQ011155.1:194169-195215;DQ011155.1:2797-1751;EPI_ISL_13331598:194662-195711;EPI_ISL_13331598:2766-1717;EPI_ISL_13363142:194421-195470;EPI_ISL_13363142:2759-1710;EPI_ISL_13374487:194476-195525;EPI_ISL_13374487:2774-1725;EPI_ISL_13502582:194470-195519;EPI_ISL_13502582:2759-1710;EPI_ISL_13508471:194521-195570;EPI_ISL_13508471:2851-1802;EPI_ISL_13607904:194833-195882;EPI_ISL_13607904:2750-1701;EPI_ISL_13624509:194255-195304;EPI_ISL_13624509:2758-1709;EPI_ISL_13889442:194562-195611;EPI_ISL_13889442:2869-1820;EPI_ISL_13953610:194369-195418;EPI_ISL_13953610:2750-1701;EPI_ISL_14181948:194503-195552;EPI_ISL_14181948:2895-1846;EPI_ISL_14838587:194068-195114;EPI_ISL_14838587:2782-1736;EPI_ISL_15263355:194165-195214;EPI_ISL_15263355:2747-1698;EPI_ISL_15269699:194389-195438;EPI_ISL_15269699:2719-1670;EPI_ISL_15293002:195112-196161;EPI_ISL_15293002:2864-1815;EPI_ISL_15641561:193802-194851;EPI_ISL_15641561:2708-1659;EPI_ISL_15831211:194441-195490;EPI_ISL_15831211:2757-1708;EPI_ISL_15992098:194420-195469;EPI_ISL_15992098:2772-1723;EPI_ISL_16233784:194441-195490;EPI_ISL_16233784:2768-1719;EPI_ISL_16946400:194374-195423;EPI_ISL_16946400:2757-1708;EPI_ISL_17048206:194046-195095;EPI_ISL_17048206:2407-1358;EPI_ISL_17703567:194442-195491;EPI_ISL_17703567:2772-1723;EPI_ISL_17718458:194419-195468;EPI_ISL_17718458:2750-1701;EPI_ISL_18213374:194447-195496;EPI_ISL_18213374:2747-1698;EPI_ISL_18360394:193116-194165;EPI_ISL_18360394:2408-1359;EPI_ISL_18557816:194394-195443;EPI_ISL_18557816:2748-1699;EPI_ISL_18642365:194453-195502;EPI_ISL_18642365:2750-1701;EPI_ISL_18659846:194723-195772;EPI_ISL_18659846:2720-1671;EPI_ISL_18744049:194448-195497;EPI_ISL_18744049:2752-1703;EPI_ISL_19022858:194392-195441;EPI_ISL_19022858:2747-1698;EPI_ISL_19028778:2664-1615;EPI_ISL_19151800:193301-194350;EPI_ISL_19151800:2748-1699;EPI_ISL_19159116:194431-195480;EPI_ISL_19159116:2750-1701;EPI_ISL_19350788:192743-193789;EPI_ISL_19350788:2791-1745;EPI_ISL_19359677:194446-195495;EPI_ISL_19359677:2747-1698;EPI_ISL_19446306:194430-195479;EPI_ISL_19446306:2742-1693;EPI_ISL_19467692:194412-195458;EPI_ISL_19467692:2941-1895;JX878411.1:195151-196197;JX878411.1:3726-2680;JX878425.1:193981-195027;JX878425.1:3165-2119;JX878426.1:194016-195062;JX878426.1:2730-1684;KC257460.1:193923-194969;KC257460.1:2563-1517;KJ642612.1:192118-193164;KJ642612.1:1069-23;KJ642613.1:193873-194919;KJ642613.1:2568-1522;KJ642616.1:196191-197240;KJ642616.1:2549-1500;KJ642618.1:191631-192677;KJ642618.1:2731-1685;KP849469.1:194457-195503;KP849469.1:2989-1943;KP849471.1:194276-195322;KP849471.1:2971-1925;LC831698.1:194429-195478;LC831698.1:2748-1699;MN648051.1:194869-195918;MN648051.1:2547-1498;MN702445.1:187516-188562;MN702446.1:2618-1572;MN702447.1:187077-188123;MN702449.1:187563-188609;MN702452.1:2637-1591;MT903338.1:195009-196058;MT903338.1:2549-1500;MT903340.1:194458-195507;MT903340.1:2750-1701;MT903341.1:182455-183504;MT903341.1:2853-1804;MT903342.1:194559-195608;MT903342.1:2749-1700;MT903347.1:195914-196963;MT903347.1:2865-1816;ON568298.1:194504-195553;ON568298.1:2896-1847;ON622713.1:195163-196212;ON622713.1:2853-1804;ON622722.2:194366-195415;ON622722.2:2753-1704;ON631963.1:194669-195718;ON631963.1:2773-1724;ON649879.1:194169-195218;ON649879.1:2583-1534;ON676707.1:194461-195510;ON676707.1:2803-1754;ON755039.1:194180-195229;ON755039.1:1991-942;ON808413.1:194377-195426;ON808413.1:2753-1704;ON880420.1:195117-196166;ON880420.1:2852-1803;ON911481.2:194662-195711;ON911481.2:2746-1697;ON918656.1:194454-195503;ON918656.1:2785-1736;ON959133.1:195024-196073;ON959133.1:2746-1697;ON959136.1:195046-196095;ON959136.1:2747-1698;ON983168.1:193590-194639;ON983168.1:1321-272;OP019276.1:194445-195494;OP019276.1:2747-1698;OP150924.1:195017-196066;OP150924.1:2760-1711;OP160532.1:194295-195344;OP160532.1:2713-1664;OP235282.1:194445-195494;OP235282.1:2747-1698;OP289782.1:194449-195498;OP289782.1:2750-1701;OP331335.1:194441-195490;OP331335.1:2767-1718;OP382479.1:194422-195471;OP382479.1:2754-1705;OP422341.1:194437-195486;OP422341.1:2767-1718;OP434519.1:187546-188595;OP434519.1:2746-1697;OP535324.1:194485-195534;OP535324.1:2748-1699;OP535333.1:194540-195589;OP535333.1:2857-1808;OP536770.1:194448-195497;OP536770.1:2749-1700;OP597769.1:194429-195478;OP597769.1:2771-1722;OP604566.1:194454-195503;OP604566.1:2750-1701;OP612682.1:194498-195547;OP612682.1:2548-1499;OP612684.1:194963-196012;OP612684.1:2372-1323;OP612686.1:194584-195633;OP612686.1:2548-1499;OP696841.1:191668-192717;OP696841.1:2747-1698;OP879722.1:193608-194657;OP879722.1:1973-924;OQ419007.1:193665-194714;OQ419007.1:1983-934;OQ419012.1:193639-194688;OQ419012.1:1975-926;OQ419031.1:193643-194692;OQ419031.1:1980-931;OQ419047.1:193645-194694;OQ419047.1:1983-934;OQ451898.1:193479-194528;OQ451898.1:2748-1699;OQ451900.1:194393-195442;OQ451900.1:2748-1699;OQ581853.1:194391-195440;OQ581853.1:2774-1725;OQ581855.1:194500-195549;OQ581855.1:2855-1806;OQ721951.1:194469-195518;OQ721951.1:2772-1723;OQ721954.1:194401-195450;OQ721954.1:2745-1696;OR035652.1:194781-195830;OR035652.1:2697-1648;OR035664.1:194727-195776;OR035664.1:2697-1648;OR035702.1:194744-195793;OR035702.1:2697-1648;OR146272.1:194383-195432;OR146272.1:2713-1664;OR146330.1:194461-195510;OR146330.1:2758-1709;OR264364.1:194438-195487;OR264364.1:2768-1719;OR427332.1:194438-195487;OR427332.1:2768-1719;OR459777.1:194424-195473;OR459777.1:2752-1703;OR459797.1:194436-195485;OR459797.1:2767-1718;OR943698.1:193979-195025;OR943698.1:2748-1702;PP098589.2:194447-195496;PP098589.2:2749-1700;PP496048.1:194365-195414;PP496048.1:2753-1704;PP601206.1:194023-195069;PP601206.1:2782-1736;PP776553.1:2698-1649;PP852971.1:194436-195485;PP852971.1:2747-1698;PP852983.1:194378-195427;PP852983.1:2752-1703;PP853000.1:194327-195376;PP853000.1:2759-1710;PP853005.1:194401-195450;PP853005.1:2754-1705;PP853007.1:194352-195401;PP853007.1:2759-1710;PP853013.1:194381-195430;PP853013.1:2760-1711;PP853027.1:194374-195423;PP853027.1:2759-1710;PP853045.1:194139-195188;PP853045.1:2763-1714;PP853048.1:194318-195367;PP853048.1:2759-1710;PP860026.1:194353-195402;PP860026.1:2756-1707;PP860027.1:194317-195366;PP860027.1:2755-1706;PP860028.1:194322-195371;PP860028.1:2755-1706;PP860031.1:194315-195364;PP860031.1:2768-1719;PP860033.1:194325-195374;PP860033.1:2755-1706;PQ153226.1:194428-195477;PQ153226.1:2747-1698;PQ178862.2:192508-193554;PQ178862.2:2812-1766;PQ221833.1:194000-195046;PQ221833.1:2781-1735;PQ340477.1:194534-195583;PQ340477.1:2714-1665;PQ340478.1:194427-195476;PQ340478.1:2715-1666;PQ340480.1:194597-195646;PQ340480.1:2746-1697;PQ340483.1:194569-195618;PQ340483.1:2714-1665;PQ340485.1:194592-195641;PQ340485.1:2716-1667;PQ340486.1:194667-195716;PQ340486.1:2715-1666;PQ340487.1:194743-195792;PQ340487.1:2708-1659;PQ340489.1:194643-195692;PQ340489.1:2715-1666;PQ340490.1:194453-195502;PQ340490.1:2714-1665;PQ340491.1:194760-195809;PQ340491.1:2708-1659;PQ340493.1:194547-195596;PQ340493.1:2716-1667;PQ340495.1:194640-195689;PQ340495.1:2714-1665;PQ340496.1:194738-195787;PQ340496.1:2747-1698;PQ368489.1:194461-195510;PQ368489.1:2750-1701;PQ451835.1:194750-195799;PQ451835.1:2710-1661;PQ451836.1:194400-195449;PQ451836.1:2770-1721;PQ451838.1:194685-195734;PQ451838.1:2741-1692;PQ451841.1:194564-195613;PQ451841.1:2733-1684;PQ451842.1:183117-184166;PQ451842.1:2733-1684 |
| OG0000002 | AY603973.1:197683-198423;AY603973.1:1511-771;AY741551.1:197246-197986;AY741551.1:1509-769;AY753185.1:197951-198691;AY753185.1:1517-777;DQ011154.1:195483-196241;DQ011154.1:1711-953;DQ011155.1:195343-196083;DQ011155.1:1623-883;EPI_ISL_13331598:195838-196578;EPI_ISL_13331598:1590-850;EPI_ISL_13363142:195597-196337;EPI_ISL_13363142:1583-843;EPI_ISL_13374487:195652-196392;EPI_ISL_13374487:1598-858;EPI_ISL_13502582:195646-196386;EPI_ISL_13502582:1583-843;EPI_ISL_13508471:195697-196437;EPI_ISL_13508471:1675-935;EPI_ISL_13607904:196009-196749;EPI_ISL_13607904:1574-834;EPI_ISL_13624509:195431-196171;EPI_ISL_13624509:1582-842;EPI_ISL_13889442:195738-196478;EPI_ISL_13889442:1693-953;EPI_ISL_13953610:195545-196285;EPI_ISL_13953610:1574-834;EPI_ISL_14181948:195679-196419;EPI_ISL_14181948:1719-979;EPI_ISL_14838587:195242-195982;EPI_ISL_14838587:1608-868;EPI_ISL_15263355:195341-196081;EPI_ISL_15263355:1571-831;EPI_ISL_15269699:195565-196305;EPI_ISL_15269699:1543-803;EPI_ISL_15293002:196288-197028;EPI_ISL_15293002:1688-948;EPI_ISL_15641561:194978-195718;EPI_ISL_15641561:1532-792;EPI_ISL_15831211:195617-196357;EPI_ISL_15831211:1581-841;EPI_ISL_15992098:195596-196336;EPI_ISL_15992098:1596-856;EPI_ISL_16233784:195617-196357;EPI_ISL_16233784:1592-852;EPI_ISL_16946400:195550-196290;EPI_ISL_16946400:1581-841;EPI_ISL_17048206:195222-195962;EPI_ISL_17048206:1231-491;EPI_ISL_17703567:195618-196358;EPI_ISL_17703567:1596-856;EPI_ISL_17718458:195595-196335;EPI_ISL_17718458:1574-834;EPI_ISL_18213374:195623-196363;EPI_ISL_18213374:1571-831;EPI_ISL_18360394:194292-195032;EPI_ISL_18360394:1232-492;EPI_ISL_18557816:195570-196310;EPI_ISL_18557816:1572-832;EPI_ISL_18642365:195629-196369;EPI_ISL_18642365:1574-834;EPI_ISL_18659846:195899-196639;EPI_ISL_18659846:1544-804;EPI_ISL_18744049:195624-196364;EPI_ISL_18744049:1576-836;EPI_ISL_19022858:195568-196308;EPI_ISL_19022858:1571-831;EPI_ISL_19028778:1488-748;EPI_ISL_19151800:194477-195217;EPI_ISL_19151800:1572-832;EPI_ISL_19159116:195607-196347;EPI_ISL_19159116:1574-834;EPI_ISL_19350788:193917-194657;EPI_ISL_19350788:1617-877;EPI_ISL_19359677:195622-196362;EPI_ISL_19359677:1571-831;EPI_ISL_19446306:195606-196346;EPI_ISL_19446306:1566-826;EPI_ISL_19467692:195586-196344;EPI_ISL_19467692:1767-1009;JX878411.1:196325-197083;JX878411.1:2552-1794;JX878425.1:195155-195913;JX878425.1:1991-1233;JX878426.1:195191-195931;JX878426.1:1555-815;KC257460.1:195097-195855;KC257460.1:1389-631;KJ642612.1:193293-194033;KJ642613.1:195047-195805;KJ642613.1:1394-636;KJ642616.1:197367-198107;KJ642616.1:1373-633;KJ642618.1:192806-193546;KJ642618.1:1556-816;KP849469.1:195631-196389;KP849469.1:1815-1057;KP849471.1:195450-196190;KP849471.1:1797-1057;LC831698.1:195605-196345;LC831698.1:1572-832;MN648051.1:196045-196785;MN648051.1:1371-631;MN702445.1:188689-189447;MN702446.1:1445-705;MN702447.1:188251-189009;MN702449.1:188736-189494;MN702452.1:1463-705;MT903338.1:196185-196925;MT903338.1:1373-633;MT903340.1:195634-196374;MT903340.1:1574-834;MT903341.1:183631-184371;MT903341.1:1677-937;MT903342.1:195735-196475;MT903342.1:1573-833;MT903347.1:197090-197830;MT903347.1:1689-949;ON568298.1:195680-196420;ON568298.1:1720-980;ON622713.1:196339-197079;ON622713.1:1677-937;ON622722.2:195542-196282;ON622722.2:1577-837;ON631963.1:195845-196585;ON631963.1:1597-857;ON649879.1:195345-196085;ON649879.1:1407-667;ON676707.1:195637-196377;ON676707.1:1627-887;ON755039.1:195356-196096;ON755039.1:815-75;ON808413.1:195553-196293;ON808413.1:1577-837;ON880420.1:196293-197033;ON880420.1:1676-936;ON911481.2:195838-196578;ON911481.2:1570-830;ON918656.1:195630-196370;ON918656.1:1609-869;ON959133.1:196200-196940;ON959133.1:1570-830;ON959136.1:196222-196962;ON959136.1:1571-831;ON983168.1:194766-194915;OP019276.1:195621-196361;OP019276.1:1571-831;OP150924.1:196193-196933;OP150924.1:1584-844;OP160532.1:195471-196211;OP160532.1:1537-797;OP235282.1:195621-196361;OP235282.1:1571-831;OP289782.1:195625-196365;OP289782.1:1574-834;OP331335.1:195617-196357;OP331335.1:1591-851;OP382479.1:195598-196338;OP382479.1:1578-838;OP422341.1:195613-196353;OP422341.1:1591-851;OP434519.1:188722-189462;OP434519.1:1570-830;OP535324.1:195661-196401;OP535324.1:1572-832;OP535333.1:195716-196456;OP535333.1:1681-941;OP536770.1:195624-196364;OP536770.1:1573-833;OP597769.1:195605-196345;OP597769.1:1595-855;OP604566.1:195630-196370;OP604566.1:1574-834;OP612682.1:195674-196414;OP612682.1:1372-632;OP612684.1:196139-196879;OP612684.1:1196-456;OP612686.1:195760-196500;OP612686.1:1372-632;OP696841.1:192844-193584;OP696841.1:1571-831;OP879722.1:194784-195524;OP879722.1:797-57;OQ419007.1:194841-195581;OQ419007.1:807-67;OQ419012.1:194815-195555;OQ419012.1:799-59;OQ419031.1:194819-195559;OQ419031.1:804-64;OQ419047.1:194821-195561;OQ419047.1:807-67;OQ451898.1:194655-195395;OQ451898.1:1572-832;OQ451900.1:195569-196309;OQ451900.1:1572-832;OQ581853.1:195567-196307;OQ581853.1:1598-858;OQ581855.1:195676-196416;OQ581855.1:1679-939;OQ721951.1:195645-196385;OQ721951.1:1596-856;OQ721954.1:195577-196317;OQ721954.1:1569-829;OR035652.1:195957-196697;OR035652.1:1521-781;OR035664.1:195903-196643;OR035664.1:1521-781;OR035702.1:195920-196660;OR035702.1:1521-781;OR146272.1:195559-196299;OR146272.1:1537-797;OR146330.1:195637-196377;OR146330.1:1582-842;OR264364.1:195614-196354;OR264364.1:1592-852;OR427332.1:195614-196354;OR427332.1:1592-852;OR459777.1:195600-196340;OR459777.1:1576-836;OR459797.1:195612-196352;OR459797.1:1591-851;OR943698.1:195153-195893;OR943698.1:1574-834;PP098589.2:195623-196363;PP098589.2:1573-833;PP496048.1:195541-196281;PP496048.1:1577-837;PP601206.1:195197-195937;PP601206.1:1608-868;PP776553.1:1522-782;PP852971.1:195612-196352;PP852971.1:1571-831;PP852983.1:195554-196294;PP852983.1:1576-836;PP853000.1:195503-196243;PP853000.1:1583-843;PP853005.1:195577-196317;PP853005.1:1578-838;PP853007.1:195528-196268;PP853007.1:1583-843;PP853013.1:195557-196297;PP853013.1:1584-844;PP853027.1:195550-196290;PP853027.1:1583-843;PP853045.1:195315-196055;PP853045.1:1587-847;PP853048.1:195494-196234;PP853048.1:1583-843;PP860026.1:195530-196270;PP860026.1:1579-839;PP860027.1:195494-196234;PP860027.1:1578-838;PP860028.1:195499-196239;PP860028.1:1578-838;PP860031.1:195492-196232;PP860031.1:1591-851;PP860033.1:195502-196242;PP860033.1:1578-838;PQ153226.1:195604-196344;PQ153226.1:1571-831;PQ178862.2:193682-194422;PQ178862.2:1638-898;PQ221833.1:195173-195913;PQ221833.1:1608-868;PQ340477.1:195710-196450;PQ340477.1:1538-798;PQ340478.1:195603-196343;PQ340478.1:1539-799;PQ340480.1:195773-196513;PQ340480.1:1570-830;PQ340483.1:195745-196485;PQ340483.1:1538-798;PQ340485.1:195768-196508;PQ340485.1:1540-800;PQ340486.1:195843-196583;PQ340486.1:1539-799;PQ340487.1:195919-196659;PQ340487.1:1532-792;PQ340489.1:195819-196559;PQ340489.1:1539-799;PQ340490.1:195629-196369;PQ340490.1:1538-798;PQ340491.1:195936-196676;PQ340491.1:1532-792;PQ340493.1:195723-196463;PQ340493.1:1540-800;PQ340495.1:195816-196556;PQ340495.1:1538-798;PQ340496.1:195914-196654;PQ340496.1:1571-831;PQ368489.1:195637-196377;PQ368489.1:1574-834;PQ451835.1:195926-196666;PQ451835.1:1534-794;PQ451836.1:195576-196316;PQ451836.1:1594-854;PQ451838.1:195861-196601;PQ451838.1:1565-825;PQ451841.1:195740-196480;PQ451841.1:1557-817;PQ451842.1:184293-185033;PQ451842.1:1557-817 |
| OG0000003 | AY603973.1:193011-194324;AY603973.1:6183-4870;AY741551.1:192748-194061;AY741551.1:6007-4694;AY753185.1:193183-194496;AY753185.1:6285-4972;DQ011154.1:190986-192299;DQ011154.1:6208-4895;DQ011155.1:190846-192159;DQ011155.1:6120-4807;EPI_ISL_13331598:191257-192570;EPI_ISL_13331598:6171-4858;EPI_ISL_13363142:191016-192329;EPI_ISL_13363142:6164-4851;EPI_ISL_13374487:191071-192384;EPI_ISL_13374487:6179-4866;EPI_ISL_13502582:191065-192378;EPI_ISL_13502582:6164-4851;EPI_ISL_13508471:191116-192429;EPI_ISL_13508471:6256-4943;EPI_ISL_13607904:191308-192621;EPI_ISL_13607904:6155-4842;EPI_ISL_13624509:190898-192211;EPI_ISL_13624509:6099-4786;EPI_ISL_13889442:191157-192470;EPI_ISL_13889442:6274-4961;EPI_ISL_13953610:190964-192277;EPI_ISL_13953610:6155-4842;EPI_ISL_14181948:191098-192411;EPI_ISL_14181948:6300-4987;EPI_ISL_14838587:190745-192058;EPI_ISL_14838587:6105-4792;EPI_ISL_15263355:190760-192073;EPI_ISL_15263355:6152-4839;EPI_ISL_15269699:190984-192297;EPI_ISL_15269699:6124-4811;EPI_ISL_15293002:191579-192892;EPI_ISL_15293002:6397-5084;EPI_ISL_15641561:190397-191710;EPI_ISL_15641561:6113-4800;EPI_ISL_15831211:191036-192349;EPI_ISL_15831211:6162-4849;EPI_ISL_15992098:191015-192328;EPI_ISL_15992098:6177-4864;EPI_ISL_16233784:191036-192349;EPI_ISL_16233784:6173-4860;EPI_ISL_16946400:190969-192282;EPI_ISL_16946400:6162-4849;EPI_ISL_17048206:190641-191954;EPI_ISL_17048206:5812-4499;EPI_ISL_17703567:191037-192350;EPI_ISL_17703567:6177-4864;EPI_ISL_17718458:191014-192327;EPI_ISL_17718458:6155-4842;EPI_ISL_18213374:191042-192355;EPI_ISL_18213374:6152-4839;EPI_ISL_18360394:189711-191024;EPI_ISL_18360394:5813-4500;EPI_ISL_18557816:190989-192302;EPI_ISL_18557816:6153-4840;EPI_ISL_18642365:191048-192361;EPI_ISL_18642365:6155-4842;EPI_ISL_18659846:191318-192631;EPI_ISL_18659846:6125-4812;EPI_ISL_18744049:191043-192356;EPI_ISL_18744049:6157-4844;EPI_ISL_19022858:190987-192300;EPI_ISL_19022858:6152-4839;EPI_ISL_19028778:6069-4756;EPI_ISL_19151800:189896-191209;EPI_ISL_19151800:6153-4840;EPI_ISL_19159116:191026-192339;EPI_ISL_19159116:6155-4842;EPI_ISL_19350788:189420-190733;EPI_ISL_19350788:6114-4801;EPI_ISL_19359677:191041-192354;EPI_ISL_19359677:6152-4839;EPI_ISL_19446306:191028-192341;EPI_ISL_19446306:6147-4834;EPI_ISL_19467692:191009-192322;EPI_ISL_19467692:6344-5031;JX878411.1:191828-193141;JX878411.1:7049-5736;JX878425.1:190658-191971;JX878425.1:6488-5175;JX878426.1:190693-192006;JX878426.1:6053-4740;KC257460.1:190600-191913;KC257460.1:5886-4573;KJ642612.1:188795-190108;KJ642612.1:4392-3079;KJ642613.1:190550-191863;KJ642613.1:5891-4578;KJ642616.1:192743-194056;KJ642616.1:5997-4684;KJ642618.1:188308-189621;KP849469.1:191134-192447;KP849469.1:6312-4999;KP849471.1:190953-192266;KP849471.1:6294-4981;LC831698.1:191024-192337;LC831698.1:6153-4840;MN648051.1:191231-192544;MN648051.1:6374-5061;MN702445.1:184193-185506;MN702446.1:5941-4628;MN702447.1:183754-185067;MN702449.1:184240-185553;MN702452.1:5960-4647;MT903338.1:191236-192549;MT903338.1:6318-5005;MT903340.1:191053-192366;MT903340.1:6155-4842;MT903341.1:178922-180235;MT903342.1:191138-192451;MT903342.1:6170-4857;MT903347.1:192510-193823;MT903347.1:6269-4956;ON568298.1:191099-192412;ON568298.1:6301-4988;ON622713.1:191630-192943;ON622713.1:6386-5073;ON622722.2:190961-192274;ON622722.2:6158-4845;ON631963.1:191264-192577;ON631963.1:6178-4865;ON649879.1:190764-192077;ON649879.1:5988-4675;ON676707.1:191056-192369;ON676707.1:6208-4895;ON755039.1:190647-191960;ON755039.1:5818-4505;ON808413.1:190972-192285;ON808413.1:6158-4845;ON880420.1:191584-192897;ON880420.1:6385-5072;ON911481.2:191239-192552;ON911481.2:6175-4862;ON918656.1:191049-192362;ON918656.1:6190-4877;ON959133.1:191491-192804;ON959133.1:6279-4966;ON959136.1:191513-192826;ON959136.1:6280-4967;ON983168.1:190057-191370;ON983168.1:4854-3541;OP019276.1:191040-192353;OP019276.1:6152-4839;OP150924.1:191484-192797;OP150924.1:6293-4980;OP160532.1:190890-192203;OP160532.1:6118-4805;OP235282.1:191040-192353;OP235282.1:6152-4839;OP289782.1:191044-192357;OP289782.1:6155-4842;OP331335.1:191036-192349;OP331335.1:6172-4859;OP382479.1:191017-192330;OP382479.1:6159-4846;OP422341.1:191032-192345;OP422341.1:6172-4859;OP434519.1:6151-4838;OP535324.1:191080-192393;OP535324.1:6153-4840;OP535333.1:191135-192448;OP535333.1:6262-4949;OP536770.1:191043-192356;OP536770.1:6154-4841;OP597769.1:191024-192337;OP597769.1:6176-4863;OP604566.1:191049-192362;OP604566.1:6155-4842;OP612682.1:190950-192263;OP612682.1:6081-4768;OP612684.1:191190-192503;OP612684.1:6145-4832;OP612686.1:191003-192316;OP612686.1:6145-4832;OP696841.1:188263-189576;OP696841.1:5849-4839;OP879722.1:190203-191516;OP879722.1:5378-4065;OQ419007.1:190260-191573;OQ419007.1:5388-4075;OQ419012.1:190234-191547;OQ419012.1:5380-4067;OQ419031.1:190238-191551;OQ419031.1:5385-4072;OQ419047.1:190240-191553;OQ419047.1:5388-4075;OQ451898.1:190074-191387;OQ451898.1:6153-4840;OQ451900.1:190988-192301;OQ451900.1:6153-4840;OQ581853.1:190986-192299;OQ581853.1:6179-4866;OQ581855.1:191095-192408;OQ581855.1:6260-4947;OQ721951.1:191064-192377;OQ721951.1:6193-4880;OQ721954.1:190996-192309;OQ721954.1:6150-4837;OR035652.1:191376-192689;OR035652.1:6102-4789;OR035664.1:191322-192635;OR035664.1:6102-4789;OR035702.1:191339-192652;OR035702.1:6102-4789;OR146272.1:190978-192291;OR146272.1:6118-4805;OR146330.1:191056-192369;OR146330.1:6163-4850;OR264364.1:191033-192346;OR264364.1:6173-4860;OR427332.1:191033-192346;OR427332.1:6173-4860;OR459777.1:191019-192332;OR459777.1:6157-4844;OR459797.1:191031-192344;OR459797.1:6172-4859;OR943698.1:190656-191969;OR943698.1:6071-4758;PP098589.2:191042-192355;PP098589.2:6154-4841;PP496048.1:190960-192273;PP496048.1:6158-4845;PP601206.1:190700-192013;PP601206.1:6105-4792;PP776553.1:6103-4790;PP852971.1:191031-192344;PP852971.1:6152-4839;PP852983.1:191037-192350;PP852983.1:6157-4844;PP853000.1:190922-192235;PP853000.1:6116-4803;PP853005.1:190996-192309;PP853005.1:6127-4814;PP853007.1:190979-192292;PP853007.1:6164-4851;PP853013.1:190976-192289;PP853013.1:6133-4820;PP853027.1:191017-192330;PP853027.1:6164-4851;PP853045.1:190782-192095;PP853045.1:6168-4855;PP853048.1:190977-192290;PP853048.1:6164-4851;PP860026.1:190980-192293;PP860026.1:6161-4848;PP860027.1:190960-192273;PP860027.1:6160-4847;PP860028.1:190917-192230;PP860028.1:6128-4815;PP860031.1:190958-192271;PP860031.1:6173-4860;PP860033.1:191255-192265;PP860033.1:5857-4847;PQ153226.1:191023-192336;PQ153226.1:6152-4839;PQ178862.2:189185-190498;PQ178862.2:6135-4822;PQ221833.1:190677-191990;PQ221833.1:6104-4791;PQ340477.1:191113-192426;PQ340477.1:6135-4822;PQ340478.1:191022-192335;PQ340478.1:6120-4807;PQ340480.1:191144-191707;PQ340480.1:6199-5636;PQ340483.1:191100-192413;PQ340483.1:6183-4870;PQ340485.1:191139-192452;PQ340485.1:6169-4856;PQ340486.1:191198-192511;PQ340486.1:6184-4871;PQ340487.1:191242-192555;PQ340487.1:6209-4896;PQ340489.1:191174-192487;PQ340489.1:6184-4871;PQ340490.1:191032-192345;PQ340490.1:6135-4822;PQ340491.1:191275-192588;PQ340491.1:6193-4880;PQ340493.1:191094-192407;PQ340493.1:6169-4856;PQ340495.1:191155-192468;PQ340495.1:6199-4886;PQ340496.1:191253-192566;PQ340496.1:6232-4919;PQ368489.1:191056-192369;PQ368489.1:6155-4842;PQ451835.1:191217-192530;PQ451835.1:6243-4930;PQ451836.1:190995-192308;PQ451836.1:6175-4862;PQ451838.1:191200-192513;PQ451838.1:6226-4913;PQ451841.1:191111-192424;PQ451841.1:6186-4873;PQ451842.1:6138-4825 |
| OG0000004 | EPI_ISL_13331598:6152-6328;EPI_ISL_13331598:191276-191100;EPI_ISL_13363142:6145-6321;EPI_ISL_13363142:191035-190859;EPI_ISL_13374487:6160-6336;EPI_ISL_13374487:191090-190914;EPI_ISL_13502582:6145-6321;EPI_ISL_13502582:191084-190908;EPI_ISL_13508471:6237-6413;EPI_ISL_13508471:191135-190959;EPI_ISL_13607904:6136-6312;EPI_ISL_13607904:191327-191151;EPI_ISL_13624509:6080-6256;EPI_ISL_13624509:190917-190741;EPI_ISL_13889442:6255-6431;EPI_ISL_13889442:191176-191000;EPI_ISL_13953610:6136-6312;EPI_ISL_13953610:190983-190807;EPI_ISL_14181948:6281-6457;EPI_ISL_14181948:191117-190941;EPI_ISL_15263355:6133-6309;EPI_ISL_15263355:190779-190603;EPI_ISL_15269699:6105-6281;EPI_ISL_15269699:191003-190827;EPI_ISL_15293002:6378-6554;EPI_ISL_15293002:191598-191422;EPI_ISL_15641561:6094-6270;EPI_ISL_15641561:190416-190240;EPI_ISL_15831211:6143-6319;EPI_ISL_15831211:191055-190879;EPI_ISL_15992098:6158-6334;EPI_ISL_15992098:191034-190858;EPI_ISL_16233784:6154-6330;EPI_ISL_16233784:191055-190879;EPI_ISL_16946400:6143-6319;EPI_ISL_16946400:190988-190812;EPI_ISL_17048206:5793-5969;EPI_ISL_17048206:190660-190484;EPI_ISL_17703567:6158-6334;EPI_ISL_17703567:191056-190880;EPI_ISL_17718458:6136-6312;EPI_ISL_17718458:191033-190857;EPI_ISL_18213374:6133-6309;EPI_ISL_18213374:191061-190885;EPI_ISL_18360394:5794-5970;EPI_ISL_18360394:189730-189554;EPI_ISL_18557816:6134-6310;EPI_ISL_18557816:191008-190832;EPI_ISL_18642365:6136-6312;EPI_ISL_18642365:191067-190891;EPI_ISL_18659846:6106-6282;EPI_ISL_18659846:191337-191161;EPI_ISL_18744049:6138-6314;EPI_ISL_18744049:191062-190886;EPI_ISL_19022858:6133-6309;EPI_ISL_19022858:191006-190830;EPI_ISL_19028778:6050-6226;EPI_ISL_19151800:6134-6310;EPI_ISL_19151800:189915-189739;EPI_ISL_19159116:6136-6312;EPI_ISL_19159116:191045-190869;EPI_ISL_19350788:6095-6274;EPI_ISL_19350788:189439-189260;EPI_ISL_19359677:6133-6309;EPI_ISL_19359677:191060-190884;EPI_ISL_19446306:6128-6304;EPI_ISL_19446306:191047-190871;LC831698.1:6134-6310;LC831698.1:191043-190867;MN648051.1:6355-6531;MN648051.1:191250-191074;MN702445.1:184212-184033;MN702446.1:5922-6101;MN702449.1:184259-184080;MT903338.1:6299-6475;MT903338.1:191255-191079;MT903340.1:6136-6312;MT903340.1:191072-190896;MT903341.1:178941-178765;MT903342.1:6151-6327;MT903342.1:191157-190981;ON568298.1:6282-6458;ON568298.1:191118-190942;ON622713.1:6367-6543;ON622713.1:191649-191473;ON622722.2:6139-6315;ON622722.2:190980-190804;ON631963.1:6159-6335;ON631963.1:191283-191107;ON649879.1:5969-6145;ON649879.1:190783-190607;ON676707.1:6189-6365;ON676707.1:191075-190899;ON755039.1:5799-5975;ON755039.1:190666-190490;ON808413.1:6139-6315;ON808413.1:190991-190815;ON880420.1:6366-6542;ON880420.1:191603-191427;ON911481.2:6156-6332;ON911481.2:191258-191082;ON918656.1:6171-6347;ON918656.1:191068-190892;ON959133.1:6260-6436;ON959133.1:191510-191334;ON959136.1:6261-6437;ON959136.1:191532-191356;ON983168.1:4835-5011;ON983168.1:190076-189900;OP019276.1:6133-6309;OP019276.1:191059-190883;OP150924.1:6274-6450;OP150924.1:191503-191327;OP160532.1:6099-6275;OP160532.1:190909-190733;OP235282.1:6133-6309;OP235282.1:191059-190883;OP289782.1:6136-6312;OP289782.1:191063-190887;OP331335.1:6153-6329;OP331335.1:191055-190879;OP382479.1:6140-6316;OP382479.1:191036-190860;OP422341.1:6153-6329;OP422341.1:191051-190875;OP434519.1:6132-6308;OP535324.1:6134-6310;OP535324.1:191099-190923;OP535333.1:6243-6419;OP535333.1:191154-190978;OP536770.1:6135-6311;OP536770.1:191062-190886;OP597769.1:6157-6333;OP597769.1:191043-190867;OP604566.1:6136-6312;OP604566.1:191068-190892;OP612682.1:6062-6238;OP612682.1:190969-190793;OP612684.1:6126-6302;OP612684.1:191209-191033;OP612686.1:6126-6302;OP612686.1:191022-190846;OP696841.1:188282-188106;OP879722.1:5359-5535;OP879722.1:190222-190046;OQ419007.1:5369-5545;OQ419007.1:190279-190103;OQ419012.1:5361-5537;OQ419012.1:190253-190077;OQ419031.1:5366-5542;OQ419031.1:190257-190081;OQ419047.1:5369-5545;OQ419047.1:190259-190083;OQ451898.1:6134-6310;OQ451898.1:190093-189917;OQ451900.1:6134-6310;OQ451900.1:191007-190831;OQ581853.1:6160-6336;OQ581853.1:191005-190829;OQ581855.1:6241-6417;OQ581855.1:191114-190938;OQ721951.1:6174-6350;OQ721951.1:191083-190907;OQ721954.1:6131-6307;OQ721954.1:191015-190839;OR035652.1:6083-6259;OR035652.1:191395-191219;OR035664.1:6083-6259;OR035664.1:191341-191165;OR035702.1:6083-6259;OR035702.1:191358-191182;OR146272.1:6099-6275;OR146272.1:190997-190821;OR146330.1:6144-6320;OR146330.1:191075-190899;OR264364.1:6154-6330;OR264364.1:191052-190876;OR427332.1:6154-6330;OR427332.1:191052-190876;OR459777.1:6138-6314;OR459777.1:191038-190862;OR459797.1:6153-6329;OR459797.1:191050-190874;PP098589.2:6135-6311;PP098589.2:191061-190885;PP496048.1:6139-6315;PP496048.1:190979-190803;PP776553.1:6084-6260;PP852971.1:6133-6309;PP852971.1:191050-190874;PP852983.1:6138-6314;PP852983.1:191056-190880;PP853000.1:6097-6273;PP853000.1:190941-190765;PP853005.1:6108-6284;PP853005.1:191015-190839;PP853007.1:6145-6321;PP853007.1:190998-190822;PP853013.1:6114-6290;PP853013.1:190995-190819;PP853027.1:6145-6321;PP853027.1:191036-190860;PP853045.1:6149-6325;PP853045.1:190801-190625;PP853048.1:6145-6321;PP853048.1:190996-190820;PP860026.1:6142-6318;PP860026.1:190999-190823;PP860027.1:6141-6317;PP860027.1:190979-190803;PP860028.1:6109-6285;PP860028.1:190936-190760;PP860031.1:6154-6330;PP860031.1:190977-190801;PP860033.1:6140-6316;PP860033.1:190972-190796;PQ153226.1:6133-6309;PQ153226.1:191042-190866;PQ178862.2:6116-6295;PQ178862.2:189204-189025;PQ340477.1:6116-6292;PQ340477.1:191132-190956;PQ340478.1:6101-6277;PQ340478.1:191041-190865;PQ340480.1:6180-6356;PQ340480.1:191163-190987;PQ340483.1:6164-6340;PQ340483.1:191119-190943;PQ340485.1:6150-6326;PQ340485.1:191158-190982;PQ340486.1:6165-6341;PQ340486.1:191217-191041;PQ340487.1:6190-6366;PQ340487.1:191261-191085;PQ340489.1:6165-6341;PQ340489.1:191193-191017;PQ340490.1:6116-6292;PQ340490.1:191051-190875;PQ340491.1:6174-6350;PQ340491.1:191294-191118;PQ340493.1:6150-6326;PQ340493.1:191113-190937;PQ340495.1:6180-6356;PQ340495.1:191174-190998;PQ340496.1:6213-6389;PQ340496.1:191272-191096;PQ368489.1:6136-6312;PQ368489.1:191075-190899;PQ451835.1:6224-6400;PQ451835.1:191236-191060;PQ451836.1:6156-6332;PQ451836.1:191014-190838;PQ451838.1:6207-6383;PQ451838.1:191219-191043;PQ451841.1:6167-6343;PQ451841.1:191130-190954;PQ451842.1:6119-6295 |
| OG0000005 | AY603973.1:182488-183060;AY741551.1:182225-182797;AY753185.1:182661-183233;DQ011154.1:180452-181024;DQ011155.1:180310-180882;EPI_ISL_13331598:180721-181293;EPI_ISL_13363142:180480-181052;EPI_ISL_13374487:180535-181107;EPI_ISL_13502582:180529-181101;EPI_ISL_13508471:180580-181152;EPI_ISL_13607904:180772-181344;EPI_ISL_13624509:180362-180934;EPI_ISL_13889442:180621-181193;EPI_ISL_13953610:180428-181000;EPI_ISL_14181948:180562-181134;EPI_ISL_14838587:180211-180783;EPI_ISL_15263355:180224-180796;EPI_ISL_15269699:180448-181020;EPI_ISL_15293002:181043-181615;EPI_ISL_15641561:179861-180433;EPI_ISL_15831211:180500-181072;EPI_ISL_15992098:180479-181051;EPI_ISL_16233784:180500-181072;EPI_ISL_16946400:180433-181005;EPI_ISL_17048206:180105-180677;EPI_ISL_17703567:180501-181073;EPI_ISL_17718458:180478-181050;EPI_ISL_18213374:180506-181078;EPI_ISL_18360394:179175-179747;EPI_ISL_18557816:180453-181025;EPI_ISL_18642365:180512-181084;EPI_ISL_18659846:180782-181354;EPI_ISL_18744049:180507-181079;EPI_ISL_19022858:180451-181023;EPI_ISL_19028778:180421-180993;EPI_ISL_19151800:179360-179932;EPI_ISL_19159116:180490-181062;EPI_ISL_19350788:178905-179477;EPI_ISL_19359677:180505-181077;EPI_ISL_19446306:180492-181064;EPI_ISL_19467692:180475-181047;JX878411.1:181296-181868;JX878425.1:180745-181317;JX878426.1:180158-180730;KC257460.1:180099-180671;KJ642612.1:178262-178834;KJ642613.1:180016-180588;KJ642616.1:182220-182792;KJ642618.1:177778-178350;KP849469.1:180602-181174;KP849471.1:180417-180989;LC831698.1:180488-181060;MN648051.1:180695-181267;MN702445.1:173658-174230;MN702446.1:180015-180587;MN702447.1:173220-173792;MN702449.1:173705-174277;MN702452.1:179901-180473;MT903338.1:180700-181272;MT903340.1:180517-181089;MT903341.1:172062-172634;MT903342.1:180602-181174;MT903347.1:181986-182558;ON568298.1:180563-181135;ON622713.1:181094-181666;ON622722.2:180425-180997;ON631963.1:180728-181300;ON649879.1:180228-180800;ON676707.1:180520-181092;ON755039.1:180111-180683;ON808413.1:180436-181008;ON880420.1:181048-181620;ON911481.2:180703-181275;ON918656.1:180513-181085;ON959133.1:180955-181527;ON959136.1:180977-181549;ON983168.1:179521-180093;OP019276.1:180504-181076;OP150924.1:180948-181520;OP160532.1:180354-180926;OP235282.1:180504-181076;OP289782.1:180508-181080;OP331335.1:180500-181072;OP382479.1:180481-181053;OP422341.1:180496-181068;OP434519.1:180486-181058;OP535324.1:180544-181116;OP535333.1:180599-181171;OP536770.1:180507-181079;OP597769.1:180489-181061;OP604566.1:180513-181085;OP612682.1:180414-180986;OP612684.1:180654-181226;OP612686.1:180467-181039;OP696841.1:177727-178299;OP879722.1:179667-180239;OQ419007.1:179724-180296;OQ419012.1:179698-180270;OQ419031.1:179702-180274;OQ419047.1:179704-180276;OQ451898.1:179538-180110;OQ451900.1:180452-181024;OQ581853.1:180450-181022;OQ581855.1:180559-181131;OQ721951.1:180528-181100;OQ721954.1:180460-181032;OR035652.1:180840-181412;OR035664.1:180786-181358;OR035702.1:180803-181375;OR146272.1:180442-181014;OR146330.1:180521-181093;OR264364.1:180497-181069;OR427332.1:180497-181069;OR459777.1:180483-181055;OR459797.1:180495-181067;OR943698.1:180119-180691;PP098589.2:180506-181078;PP496048.1:180424-180996;PP601206.1:180166-180738;PP776553.1:180376-180948;PP852971.1:180495-181067;PP852983.1:180501-180671;PP852983.1:180831-181073;PP853000.1:180386-180556;PP853000.1:180716-180958;PP853005.1:180460-180630;PP853005.1:180790-181032;PP853007.1:180443-180613;PP853007.1:180773-181015;PP853013.1:180440-181012;PP853027.1:180481-180651;PP853027.1:180811-181053;PP853045.1:180246-180818;PP853048.1:180441-180611;PP853048.1:180771-181013;PP860026.1:180444-181016;PP860027.1:180425-180997;PP860028.1:180380-180952;PP860031.1:180421-180993;PP860033.1:180418-180990;PQ153226.1:180487-181059;PQ178862.2:178670-179242;PQ221506.1:174024-174596;PQ221833.1:180143-180715;PQ340477.1:180577-181149;PQ340478.1:180486-181058;PQ340480.1:180608-181180;PQ340483.1:180564-181136;PQ340485.1:180603-181175;PQ340486.1:180662-181234;PQ340487.1:180706-181278;PQ340489.1:180638-181210;PQ340490.1:180496-181068;PQ340491.1:180739-181311;PQ340493.1:180558-181130;PQ340495.1:180619-181191;PQ340496.1:180717-181289;PQ368489.1:180520-181092;PQ451835.1:180681-181253;PQ451836.1:180459-181031;PQ451838.1:180664-181236;PQ451841.1:180575-181147;PQ451842.1:180457-180630 |
| OG0000006 | AY603973.1:192366-192896;AY603973.1:6828-6298;AY741551.1:192103-192633;AY741551.1:6652-6122;AY753185.1:192538-193068;AY753185.1:6930-6400;DQ011154.1:190340-190870;DQ011155.1:190200-190730;EPI_ISL_13331598:190613-191143;EPI_ISL_13363142:190372-190902;EPI_ISL_13374487:190427-190957;EPI_ISL_13502582:190421-190951;EPI_ISL_13508471:190472-191002;EPI_ISL_13607904:190664-191194;EPI_ISL_13624509:190254-190784;EPI_ISL_13889442:190513-191043;EPI_ISL_13953610:190320-190850;EPI_ISL_14181948:190454-190984;EPI_ISL_14838587:190099-190629;EPI_ISL_15263355:190116-190646;EPI_ISL_15269699:190340-190870;EPI_ISL_15293002:190935-191465;EPI_ISL_15641561:189753-190283;EPI_ISL_15831211:190392-190922;EPI_ISL_15992098:190371-190901;EPI_ISL_16233784:190392-190922;EPI_ISL_16946400:190325-190855;EPI_ISL_17048206:189997-190527;EPI_ISL_17703567:190393-190923;EPI_ISL_17718458:190370-190900;EPI_ISL_18213374:190398-190928;EPI_ISL_18360394:189067-189597;EPI_ISL_18557816:190345-190875;EPI_ISL_18642365:190404-190934;EPI_ISL_18659846:190674-191204;EPI_ISL_18744049:190399-190929;EPI_ISL_19022858:190343-190873;EPI_ISL_19028778:190313-190690;EPI_ISL_19151800:189252-189782;EPI_ISL_19159116:190382-190912;EPI_ISL_19350788:188773-189303;EPI_ISL_19359677:190397-190927;EPI_ISL_19446306:190384-190914;EPI_ISL_19467692:190363-190893;JX878411.1:191182-191712;JX878425.1:190162-190542;JX878426.1:190047-190577;KC257460.1:189954-190484;KJ642612.1:188149-188679;KJ642613.1:189904-190434;KJ642616.1:192098-192628;KJ642616.1:6642-6112;KJ642618.1:187662-188192;KP849469.1:190489-191019;KP849471.1:190307-190837;LC831698.1:190380-190910;MN648051.1:190587-191117;MN702445.1:183546-184076;MN702446.1:189902-190354;MN702447.1:183108-183638;MN702449.1:183593-184123;MN702452.1:189789-190241;MT903338.1:190592-191122;MT903340.1:190409-190939;MT903341.1:178278-178808;MT903342.1:190494-191024;MT903347.1:191865-192395;MT903347.1:6914-6384;ON568298.1:190455-190985;ON622713.1:190986-191516;ON622722.2:190317-190847;ON631963.1:190620-191150;ON649879.1:190120-190650;ON676707.1:190412-190942;ON755039.1:190003-190533;ON808413.1:190328-190858;ON880420.1:190940-191470;ON911481.2:190595-191125;ON918656.1:190405-190935;ON959133.1:190847-191377;ON959136.1:190869-191399;ON983168.1:189413-189943;OP019276.1:190396-190926;OP150924.1:190840-191370;OP160532.1:190246-190776;OP235282.1:190396-190926;OP289782.1:190400-190930;OP331335.1:190392-190922;OP382479.1:190373-190903;OP422341.1:190388-190918;OP535324.1:190436-190966;OP535333.1:190491-191021;OP536770.1:190399-190929;OP597769.1:190572-190910;OP597769.1:190381-190596;OP604566.1:190405-190935;OP612682.1:190306-190836;OP612684.1:190546-191076;OP612686.1:190359-190889;OP696841.1:187619-188149;OP879722.1:189559-190089;OQ419007.1:189616-190146;OQ419012.1:189590-190120;OQ419031.1:189594-190124;OQ419047.1:189596-190126;OQ451898.1:189430-189960;OQ451900.1:190344-190874;OQ581853.1:190342-190872;OQ581855.1:190451-190981;OQ721951.1:190420-190950;OQ721954.1:190352-190882;OR035652.1:190732-191262;OR035664.1:190678-191208;OR035702.1:190695-191225;OR146272.1:190334-190864;OR146330.1:190412-190942;OR264364.1:190389-190919;OR427332.1:190389-190919;OR459777.1:190375-190905;OR459797.1:190387-190917;OR943698.1:190010-190540;PP098589.2:190398-190928;PP496048.1:190316-190846;PP601206.1:190054-190584;PP776553.1:190268-190615;PP852971.1:190387-190917;PP852983.1:190393-190923;PP853000.1:190278-190808;PP853005.1:190352-190882;PP853007.1:190335-190865;PP853013.1:190332-190862;PP853027.1:190373-190903;PP853045.1:190138-190668;PP853048.1:190333-190863;PP860026.1:190336-190866;PP860027.1:190316-190846;PP860028.1:190273-190803;PP860031.1:190314-190844;PP860033.1:190309-190839;PQ153226.1:190379-190909;PQ178862.2:188538-189068;PQ221506.1:183910-184350;PQ221833.1:190031-190561;PQ340477.1:190469-190999;PQ340478.1:190378-190908;PQ340480.1:190500-191030;PQ340483.1:190456-190986;PQ340485.1:190495-191025;PQ340486.1:190554-191084;PQ340487.1:190598-191128;PQ340489.1:190530-191060;PQ340490.1:190388-190918;PQ340491.1:190631-191161;PQ340493.1:190450-190980;PQ340495.1:190511-191041;PQ340496.1:190609-191139;PQ368489.1:190412-190942;PQ451835.1:190573-191103;PQ451836.1:190351-190881;PQ451838.1:190556-191086;PQ451841.1:190467-190997 |
| OG0000007 | AY603973.1:170082-170300;AY603973.1:170389-170745;AY741551.1:170116-170334;AY741551.1:170423-170779;AY753185.1:170652-171008;AY753185.1:170345-170563;DQ011154.1:168006-168671;DQ011155.1:167948-168613;EPI_ISL_13331598:168224-168889;EPI_ISL_13363142:168212-168877;EPI_ISL_13374487:168245-168910;EPI_ISL_13502582:168240-168905;EPI_ISL_13508471:168306-168971;EPI_ISL_13607904:168211-168876;EPI_ISL_13624509:168146-168811;EPI_ISL_13889442:168339-169004;EPI_ISL_13953610:168139-168804;EPI_ISL_14181948:168369-169034;EPI_ISL_14838587:167960-168625;EPI_ISL_15263355:168205-168870;EPI_ISL_15269699:168174-168839;EPI_ISL_15293002:168443-169108;EPI_ISL_15831211:168214-168879;EPI_ISL_15992098:168254-168919;EPI_ISL_16233784:168226-168891;EPI_ISL_16946400:168216-168881;EPI_ISL_17048206:167880-168545;EPI_ISL_17703567:168227-168892;EPI_ISL_17718458:168207-168872;EPI_ISL_18213374:168218-168883;EPI_ISL_18360394:167525-168190;EPI_ISL_18557816:168221-168886;EPI_ISL_18642365:168216-168881;EPI_ISL_18659846:168175-168840;EPI_ISL_18744049:168224-168889;EPI_ISL_19022858:168181-168846;EPI_ISL_19028778:168133-168798;EPI_ISL_19151800:168217-168540;EPI_ISL_19159116:168202-168867;EPI_ISL_19350788:166592-167257;EPI_ISL_19359677:168217-168882;EPI_ISL_19446306:168204-168869;EPI_ISL_19467692:168147-168812;JX878411.1:168848-169513;JX878425.1:168335-169000;JX878426.1:167908-168573;KC257460.1:167730-168395;KJ642612.1:166079-166744;KJ642613.1:167614-168279;KJ642616.1:169801-170019;KJ642616.1:170108-170464;KJ642618.1:165545-166210;KP849469.1:168164-168829;KP849471.1:168099-168764;LC831698.1:168200-168865;MN648051.1:168443-169108;MN702445.1:161482-162147;MN702446.1:167646-168311;MN702447.1:161016-161681;MN702449.1:161520-162185;MN702452.1:167704-168369;MT903338.1:168376-169041;MT903340.1:168229-168894;MT903341.1:159770-160435;MT903342.1:168266-168931;MT903347.1:170151-170306;ON568298.1:168370-169035;ON622713.1:168434-169099;ON622722.2:168222-168887;ON631963.1:168231-168896;ON649879.1:168037-168702;ON676707.1:168268-168933;ON755039.1:167859-168524;ON808413.1:168196-168861;ON880420.1:168432-169097;ON911481.2:168217-168882;ON918656.1:168239-168904;ON959133.1:168328-168993;ON959136.1:168329-168994;ON983168.1:166905-167570;OP019276.1:168216-168881;OP150924.1:168342-169007;OP160532.1:168165-168830;OP235282.1:168216-168881;OP289782.1:168220-168885;OP331335.1:168225-168890;OP382479.1:168209-168874;OP422341.1:168222-168887;OP434519.1:168198-168863;OP535324.1:168229-168894;OP535333.1:168336-169001;OP536770.1:168219-168884;OP597769.1:168253-168918;OP604566.1:168219-168884;OP612682.1:168154-168819;OP612684.1:168205-168870;OP612686.1:168207-168872;OP696841.1:165495-166160;OP879722.1:167442-168107;OQ419007.1:167436-168101;OQ419012.1:167410-168075;OQ419031.1:167414-168079;OQ419047.1:167416-168081;OQ451898.1:167306-167971;OQ451900.1:168220-168885;OQ581853.1:168228-168893;OQ581855.1:168327-168992;OQ721951.1:168246-168911;OQ721954.1:168219-168884;OR035652.1:168148-168813;OR035664.1:168152-168817;OR035702.1:168149-168814;OR146272.1:168168-168833;OR146330.1:168229-168894;OR264364.1:168223-168888;OR427332.1:168223-168888;OR459777.1:168212-168877;OR459797.1:168221-168886;OR943698.1:167854-168519;PP098589.2:168218-168883;PP496048.1:168222-168887;PP601206.1:167907-168572;PP776553.1:168142-168807;PP852971.1:168206-168871;PP852983.1:168227-168892;PP853000.1:168178-168843;PP853005.1:168186-168851;PP853007.1:168229-168894;PP853013.1:168201-168866;PP853027.1:168243-168908;PP853045.1:168194-168859;PP853048.1:168219-168884;PP860026.1:168226-168891;PP860027.1:168236-168901;PP860028.1:168175-168840;PP860031.1:168222-168872;PP860033.1:168219-168884;PQ153226.1:168199-168864;PQ178862.2:166571-167236;PQ221506.1:161741-162406;PQ221833.1:167888-168553;PQ340477.1:168186-168851;PQ340478.1:168168-168833;PQ340480.1:168246-168911;PQ340483.1:168232-168897;PQ340485.1:168215-168880;PQ340486.1:168233-168898;PQ340487.1:168257-168922;PQ340489.1:168232-168897;PQ340490.1:168184-168849;PQ340491.1:168239-168904;PQ340493.1:168223-168888;PQ340495.1:168249-168914;PQ340496.1:168284-168949;PQ368489.1:168232-168897;PQ451835.1:168289-168954;PQ451836.1:168227-168892;PQ451838.1:168277-168942;PQ451841.1:168240-168905;PQ451842.1:168186-168851 |
| OG0000008 | AY603973.1:191156-191617;AY603973.1:8038-7577;AY741551.1:190893-191354;AY741551.1:7862-7401;AY753185.1:191328-191789;AY753185.1:8140-7679;DQ011154.1:189131-189592;DQ011155.1:188990-189451;EPI_ISL_13331598:189402-189863;EPI_ISL_13363142:189161-189622;EPI_ISL_13374487:189216-189677;EPI_ISL_13502582:189210-189671;EPI_ISL_13508471:189261-189722;EPI_ISL_13607904:189453-189914;EPI_ISL_13624509:189043-189504;EPI_ISL_13889442:189302-189763;EPI_ISL_13953610:189109-189570;EPI_ISL_14181948:189243-189704;EPI_ISL_14838587:188890-189351;EPI_ISL_15263355:188905-189366;EPI_ISL_15269699:189129-189590;EPI_ISL_15293002:189724-190185;EPI_ISL_15641561:188542-189003;EPI_ISL_15831211:189181-189642;EPI_ISL_15992098:189160-189621;EPI_ISL_16233784:189181-189642;EPI_ISL_16946400:189114-189575;EPI_ISL_17048206:188786-189247;EPI_ISL_17703567:189182-189643;EPI_ISL_17718458:189159-189620;EPI_ISL_18213374:189187-189648;EPI_ISL_18360394:187856-188317;EPI_ISL_18557816:189134-189595;EPI_ISL_18642365:189193-189654;EPI_ISL_18659846:189463-189924;EPI_ISL_18744049:189188-189649;EPI_ISL_19022858:189132-189593;EPI_ISL_19028778:189102-189563;EPI_ISL_19151800:188041-188502;EPI_ISL_19159116:189171-189632;EPI_ISL_19350788:187564-188025;EPI_ISL_19359677:189186-189647;EPI_ISL_19446306:189173-189634;EPI_ISL_19467692:189154-189615;JX878411.1:189974-190435;JX878425.1:189424-189885;JX878426.1:188838-189299;KC257460.1:188745-189206;KJ642612.1:186940-187401;KJ642613.1:188695-189156;KJ642616.1:190888-191349;KJ642616.1:7852-7391;KJ642618.1:186453-186914;KP849469.1:189280-189741;KP849471.1:189098-189559;LC831698.1:189169-189630;MN648051.1:189376-189837;MN702445.1:182337-182798;MN702446.1:188693-189154;MN702447.1:181899-182360;MN702449.1:182384-182845;MN702452.1:188580-189041;MT903338.1:189381-189842;MT903340.1:189198-189659;MT903341.1:177067-177528;MT903342.1:189283-189744;MT903347.1:190655-191116;MT903347.1:8124-7663;ON568298.1:189244-189705;ON622713.1:189775-190236;ON622722.2:189106-189567;ON631963.1:189409-189870;ON649879.1:188909-189370;ON676707.1:189201-189662;ON755039.1:188792-189253;ON808413.1:189117-189578;ON880420.1:189729-190190;ON911481.2:189384-189845;ON918656.1:189194-189655;ON959133.1:189636-190097;ON959136.1:189658-190119;ON983168.1:188202-188663;OP019276.1:189185-189646;OP150924.1:189629-190090;OP160532.1:189035-189496;OP235282.1:189185-189646;OP289782.1:189189-189650;OP331335.1:189181-189642;OP382479.1:189162-189623;OP422341.1:189177-189638;OP535324.1:189225-189686;OP535333.1:189280-189741;OP536770.1:189188-189649;OP597769.1:189170-189631;OP604566.1:189194-189655;OP612682.1:189095-189556;OP612684.1:189335-189796;OP612686.1:189148-189609;OP696841.1:186408-186869;OP879722.1:188348-188809;OQ419007.1:188405-188866;OQ419012.1:188379-188840;OQ419031.1:188383-188844;OQ419047.1:188385-188846;OQ451898.1:188219-188680;OQ451900.1:189133-189594;OQ581853.1:189131-189592;OQ581855.1:189240-189701;OQ721951.1:189209-189670;OQ721954.1:189141-189602;OR035652.1:189521-189982;OR035664.1:189467-189928;OR035702.1:189484-189945;OR146272.1:189123-189584;OR146330.1:189201-189662;OR264364.1:189178-189639;OR427332.1:189178-189639;OR459777.1:189164-189625;OR459797.1:189176-189637;OR943698.1:188801-189262;PP098589.2:189187-189648;PP496048.1:189105-189566;PP601206.1:188845-189306;PP776553.1:189057-189518;PP852971.1:189176-189637;PP852983.1:189182-189643;PP853000.1:189067-189528;PP853005.1:189141-189602;PP853007.1:189124-189585;PP853013.1:189121-189582;PP853027.1:189162-189623;PP853045.1:188927-189388;PP853048.1:189122-189583;PP860026.1:189125-189586;PP860027.1:189105-189566;PP860028.1:189062-189523;PP860031.1:189103-189564;PP860033.1:189098-189559;PQ153226.1:189168-189629;PQ178862.2:187329-187790;PQ221506.1:182701-183162;PQ221833.1:188822-189283;PQ340477.1:189258-189719;PQ340478.1:189167-189628;PQ340480.1:189289-189750;PQ340483.1:189245-189706;PQ340485.1:189284-189745;PQ340486.1:189343-189804;PQ340487.1:189387-189848;PQ340489.1:189319-189780;PQ340490.1:189177-189638;PQ340491.1:189420-189881;PQ340493.1:189239-189700;PQ340495.1:189300-189761;PQ340496.1:189398-189859;PQ368489.1:189201-189662;PQ451835.1:189362-189823;PQ451836.1:189140-189601;PQ451838.1:189345-189806;PQ451841.1:189256-189717 |
| OG0000009 | AY603973.1:190587-190952;AY603973.1:8607-8242;AY741551.1:190324-190575;AY741551.1:8431-8180;AY753185.1:190759-191124;AY753185.1:8709-8344;DQ011154.1:188609-188926;DQ011155.1:188468-188785;EPI_ISL_13331598:188833-189198;EPI_ISL_13363142:188592-188957;EPI_ISL_13374487:188647-189012;EPI_ISL_13502582:188641-189006;EPI_ISL_13508471:188692-189057;EPI_ISL_13607904:188884-189249;EPI_ISL_13624509:188474-188839;EPI_ISL_13889442:188733-189098;EPI_ISL_13953610:188540-188905;EPI_ISL_14181948:188674-189039;EPI_ISL_14838587:188368-188685;EPI_ISL_15263355:188336-188701;EPI_ISL_15269699:188560-188925;EPI_ISL_15293002:189155-189520;EPI_ISL_15641561:187973-188338;EPI_ISL_15831211:188612-188977;EPI_ISL_15992098:188591-188956;EPI_ISL_16233784:188612-188977;EPI_ISL_16946400:188545-188910;EPI_ISL_17048206:188217-188582;EPI_ISL_17703567:188613-188978;EPI_ISL_17718458:188590-188955;EPI_ISL_18213374:188618-188983;EPI_ISL_18360394:187287-187652;EPI_ISL_18557816:188565-188930;EPI_ISL_18642365:188624-188989;EPI_ISL_18659846:188894-189259;EPI_ISL_18744049:188619-188984;EPI_ISL_19022858:188563-188928;EPI_ISL_19028778:188533-188898;EPI_ISL_19151800:187472-187837;EPI_ISL_19159116:188602-188967;EPI_ISL_19350788:187042-187359;EPI_ISL_19359677:188617-188982;EPI_ISL_19446306:188604-188969;EPI_ISL_19467692:188632-188949;JX878411.1:189452-189769;JX878425.1:188902-189219;JX878426.1:188316-188633;KC257460.1:188223-188540;KJ642612.1:186418-186735;KJ642613.1:188173-188490;KJ642616.1:190319-190684;KJ642616.1:8424-8056;KJ642618.1:185931-186248;KP849469.1:188759-189076;KP849471.1:188576-188893;LC831698.1:188600-188965;MN648051.1:188807-189172;MN702445.1:181815-182132;MN702446.1:188171-188488;MN702447.1:181377-181694;MN702449.1:181862-182179;MN702452.1:188058-188375;MT903338.1:188812-189177;MT903340.1:188629-188994;MT903342.1:188714-189079;MT903347.1:190085-190450;MT903347.1:8694-8329;ON568298.1:188675-189040;ON622713.1:189206-189571;ON622722.2:188537-188902;ON631963.1:188840-189205;ON649879.1:188340-188705;ON676707.1:188632-188997;ON755039.1:188223-188588;ON808413.1:188548-188913;ON880420.1:189160-189525;ON911481.2:188815-189180;ON918656.1:188625-188990;ON959133.1:189067-189432;ON959136.1:189089-189454;ON983168.1:187633-187998;OP019276.1:188616-188981;OP150924.1:189060-189425;OP160532.1:188466-188831;OP235282.1:188616-188981;OP289782.1:188620-188985;OP331335.1:188612-188977;OP382479.1:188593-188958;OP422341.1:188608-188973;OP535324.1:188656-189021;OP535333.1:188711-189076;OP536770.1:188619-188984;OP597769.1:188601-188966;OP604566.1:188625-188990;OP612682.1:188526-188891;OP612684.1:188766-189131;OP612686.1:188579-188944;OP696841.1:185839-186204;OP879722.1:187779-188144;OQ419007.1:187836-188201;OQ419012.1:187810-188175;OQ419031.1:187814-188179;OQ419047.1:187816-188181;OQ451898.1:187650-188015;OQ451900.1:188564-188929;OQ581853.1:188562-188927;OQ581855.1:188671-189036;OQ721951.1:188640-189005;OQ721954.1:188572-188937;OR035652.1:188952-189317;OR035664.1:188898-189263;OR035702.1:188915-189280;OR146272.1:188554-188919;OR146330.1:188632-188997;OR264364.1:188609-188974;OR427332.1:188609-188974;OR459777.1:188595-188960;OR459797.1:188607-188972;OR943698.1:188279-188596;PP098589.2:188618-188983;PP496048.1:188536-188901;PP601206.1:188323-188640;PP776553.1:188488-188853;PP852971.1:188607-188972;PP852983.1:188613-188978;PP853000.1:188498-188863;PP853005.1:188572-188937;PP853007.1:188555-188920;PP853013.1:188552-188917;PP853027.1:188593-188958;PP853045.1:188358-188723;PP853048.1:188553-188918;PP860026.1:188556-188921;PP860027.1:188536-188901;PP860028.1:188493-188858;PP860031.1:188534-188899;PP860033.1:188529-188894;PQ153226.1:188599-188964;PQ178862.2:186807-187124;PQ221506.1:182179-182496;PQ221833.1:188300-188617;PQ340477.1:188689-189054;PQ340478.1:188598-188963;PQ340480.1:188720-189085;PQ340483.1:188676-189041;PQ340485.1:188715-189080;PQ340486.1:188774-189139;PQ340487.1:188818-189183;PQ340489.1:188750-189115;PQ340490.1:188608-188973;PQ340491.1:188851-189216;PQ340493.1:188670-189035;PQ340495.1:188731-189096;PQ340496.1:188829-189194;PQ368489.1:188632-188997;PQ451835.1:188793-189158;PQ451836.1:188571-188936;PQ451838.1:188776-189141;PQ451841.1:188687-189052 |
| OG0000010 | AY603973.1:180853-182316;AY741551.1:180860-182053;AY753185.1:181116-182489;DQ011154.1:179200-180273;DQ011155.1:179058-180131;EPI_ISL_13331598:179070-180542;EPI_ISL_13363142:179108-180301;EPI_ISL_13374487:179271-180356;EPI_ISL_13502582:179157-180350;EPI_ISL_13508471:179154-180401;EPI_ISL_13607904:179520-180593;EPI_ISL_13624509:178990-180183;EPI_ISL_13889442:179249-180442;EPI_ISL_13953610:178984-180249;EPI_ISL_14181948:179217-180383;EPI_ISL_14838587:178959-180032;EPI_ISL_15263355:178972-180045;EPI_ISL_15269699:179022-180269;EPI_ISL_15293002:179293-180864;EPI_ISL_15641561:178516-179682;EPI_ISL_15831211:179128-180321;EPI_ISL_15992098:179134-180300;EPI_ISL_16233784:179074-180321;EPI_ISL_16946400:179052-180254;EPI_ISL_17048206:178760-179926;EPI_ISL_17703567:179075-180322;EPI_ISL_17718458:179034-180299;EPI_ISL_18213374:179062-180327;EPI_ISL_18360394:177975-178241;EPI_ISL_18360394:178376-178996;EPI_ISL_18557816:179081-180274;EPI_ISL_18642365:179068-180333;EPI_ISL_18659846:179023-180603;EPI_ISL_18744049:179063-180328;EPI_ISL_19022858:179025-180272;EPI_ISL_19028778:178977-180242;EPI_ISL_19151800:177988-179181;EPI_ISL_19159116:179046-180311;EPI_ISL_19350788:177524-178726;EPI_ISL_19359677:179061-180326;EPI_ISL_19446306:179048-180313;EPI_ISL_19467692:179013-180296;JX878411.1:180044-181117;JX878425.1:179493-180566;JX878426.1:178906-179979;KC257460.1:178847-179920;KJ642612.1:177010-178083;KJ642613.1:178764-179837;KJ642616.1:180558-182048;KJ642618.1:176433-177599;KP849469.1:179350-180423;KP849471.1:178934-180238;LC831698.1:179044-180309;MN648051.1:179443-180516;MN702445.1:172304-173479;MN702446.1:178763-179836;MN702447.1:171968-173041;MN702449.1:172342-173526;MN702452.1:178649-179722;MT903338.1:179256-180521;MT903340.1:179073-180338;MT903341.1:170636-171883;MT903342.1:179176-180423;MT903347.1:180424-181815;ON568298.1:179218-180384;ON622713.1:179290-180915;ON622722.2:179173-180246;ON631963.1:179077-180549;ON649879.1:178883-180049;ON676707.1:179148-180341;ON755039.1:178859-179932;ON808413.1:179046-180257;ON880420.1:179280-180869;ON911481.2:179070-180524;ON918656.1:179087-180334;ON959133.1:179178-180776;ON959136.1:179173-180798;ON983168.1:177753-179342;OP019276.1:179060-180325;OP150924.1:179696-180769;OP160532.1:179009-180175;OP235282.1:179060-180325;OP289782.1:179064-180329;OP331335.1:179074-180321;OP382479.1:179055-180302;OP422341.1:179070-180317;OP434519.1:179042-180307;OP535324.1:179292-180365;OP535333.1:179182-180420;OP536770.1:179063-180328;OP597769.1:179143-179934;OP597769.1:179957-180310;OP604566.1:179069-180334;OP612682.1:178970-180235;OP612684.1:179021-180475;OP612686.1:179023-180288;OP696841.1:176355-177548;OP879722.1:178322-179488;OQ419007.1:178280-179545;OQ419012.1:178254-179519;OQ419031.1:178258-179523;OQ419047.1:178260-179525;OQ451898.1:178166-179359;OQ451900.1:179080-180273;OQ581853.1:179078-180271;OQ581855.1:179187-180380;OQ721951.1:179066-180349;OQ721954.1:179208-180281;OR035652.1:179588-180661;OR035664.1:179000-180607;OR035702.1:178999-180624;OR146272.1:179016-180263;OR146330.1:179149-180342;OR264364.1:179071-180318;OR427332.1:179071-180318;OR459777.1:179039-180304;OR459797.1:179069-180316;OR943698.1:178531-179940;PP098589.2:179062-180327;PP496048.1:179031-180245;PP601206.1:178914-179987;PP776553.1:179004-180197;PP852971.1:179051-180316;PP852983.1:179075-180322;PP853000.1:179014-180207;PP853005.1:179034-180281;PP853007.1:179071-180264;PP853013.1:179068-180261;PP853027.1:179109-180302;PP853045.1:178874-180067;PP853048.1:179069-180262;PP860026.1:179072-180265;PP860027.1:179053-180246;PP860028.1:179008-180201;PP860031.1:179049-180242;PP860033.1:179046-180239;PQ153226.1:179043-180308;PQ178862.2:177418-178491;PQ221506.1:172772-173845;PQ221833.1:178696-179964;PQ340477.1:179034-180398;PQ340478.1:179024-180307;PQ340480.1:179092-180429;PQ340483.1:179084-180385;PQ340485.1:179069-180424;PQ340486.1:179083-180483;PQ340487.1:179109-180527;PQ340489.1:179086-180459;PQ340490.1:179034-180317;PQ340491.1:179097-180560;PQ340493.1:179069-180379;PQ340495.1:179103-180440;PQ340496.1:179138-180538;PQ368489.1:179076-180341;PQ451835.1:179147-180502;PQ451836.1:179087-180280;PQ451838.1:179139-180485;PQ451841.1:179104-180396;PQ451842.1:179040-180278 |
| OG0000011 | AY603973.1:8237-8434;AY603973.1:190957-190760;AY741551.1:8061-8258;AY741551.1:190694-190497;AY753185.1:8339-8536;AY753185.1:191129-190932;DQ011154.1:188931-188734;DQ011155.1:188790-188593;EPI_ISL_13331598:189203-189006;EPI_ISL_13363142:188962-188765;EPI_ISL_13374487:189017-188820;EPI_ISL_13502582:189011-188814;EPI_ISL_13508471:189062-188865;EPI_ISL_13607904:189254-189057;EPI_ISL_13624509:188844-188647;EPI_ISL_13889442:189103-188906;EPI_ISL_13953610:188910-188713;EPI_ISL_14181948:189044-188847;EPI_ISL_14838587:188690-188493;EPI_ISL_15263355:188706-188509;EPI_ISL_15269699:188930-188733;EPI_ISL_15293002:189525-189328;EPI_ISL_15641561:188343-188146;EPI_ISL_15831211:188982-188785;EPI_ISL_15992098:188961-188764;EPI_ISL_16233784:188982-188785;EPI_ISL_16946400:188915-188718;EPI_ISL_17048206:188587-188390;EPI_ISL_17703567:188983-188786;EPI_ISL_17718458:188960-188763;EPI_ISL_18213374:188988-188791;EPI_ISL_18360394:187657-187460;EPI_ISL_18557816:188935-188738;EPI_ISL_18642365:188994-188797;EPI_ISL_18659846:189264-189067;EPI_ISL_18744049:188989-188792;EPI_ISL_19022858:188933-188736;EPI_ISL_19028778:188903-188706;EPI_ISL_19151800:187842-187645;EPI_ISL_19159116:188972-188775;EPI_ISL_19350788:187364-187167;EPI_ISL_19359677:188987-188790;EPI_ISL_19446306:188974-188777;EPI_ISL_19467692:188954-188757;JX878411.1:189774-189577;JX878425.1:189224-189027;JX878426.1:188638-188441;KC257460.1:188545-188348;KJ642612.1:186740-186543;KJ642613.1:188495-188298;KJ642616.1:8051-8248;KJ642616.1:190689-190492;KJ642618.1:186253-186056;KP849469.1:189081-188884;KP849471.1:188898-188701;LC831698.1:188970-188773;MN648051.1:189177-188980;MN702445.1:182137-181940;MN702446.1:188493-188296;MN702447.1:181699-181502;MN702449.1:182184-181987;MN702452.1:188380-188183;MT903338.1:189182-188985;MT903340.1:188999-188802;MT903342.1:189084-188887;MT903347.1:8312-8521;MT903347.1:190467-190258;ON568298.1:189045-188848;ON622713.1:189576-189379;ON622722.2:188907-188710;ON631963.1:189210-189013;ON649879.1:188710-188513;ON676707.1:189002-188805;ON755039.1:188593-188396;ON808413.1:188918-188721;ON880420.1:189530-189333;ON911481.2:189185-188988;ON918656.1:188995-188798;ON959133.1:189437-189240;ON959136.1:189459-189262;ON983168.1:188003-187806;OP019276.1:188986-188789;OP150924.1:189430-189233;OP160532.1:188836-188639;OP235282.1:188986-188789;OP289782.1:188990-188793;OP331335.1:188982-188785;OP382479.1:188963-188766;OP422341.1:188978-188781;OP535324.1:189026-188829;OP535333.1:189081-188884;OP536770.1:188989-188792;OP597769.1:188971-188774;OP604566.1:188995-188798;OP612682.1:188896-188699;OP612684.1:189136-188939;OP612686.1:188949-188752;OP696841.1:186209-186012;OP879722.1:188149-187952;OQ419007.1:188206-188009;OQ419012.1:188180-187983;OQ419031.1:188184-187987;OQ419047.1:188186-187989;OQ451898.1:188020-187823;OQ451900.1:188934-188737;OQ581853.1:188932-188735;OQ581855.1:189041-188844;OQ721951.1:189010-188813;OQ721954.1:188942-188745;OR035652.1:189322-189125;OR035664.1:189268-189071;OR035702.1:189285-189088;OR146272.1:188924-188727;OR146330.1:189002-188805;OR264364.1:188979-188782;OR427332.1:188979-188782;OR459777.1:188965-188768;OR459797.1:188977-188780;OR943698.1:188601-188404;PP098589.2:188988-188791;PP496048.1:188906-188709;PP601206.1:188645-188448;PP776553.1:188858-188661;PP852971.1:188977-188780;PP852983.1:188983-188786;PP853000.1:188868-188671;PP853005.1:188942-188745;PP853007.1:188925-188728;PP853013.1:188922-188725;PP853027.1:188963-188766;PP853045.1:188728-188531;PP853048.1:188923-188726;PP860026.1:188926-188729;PP860027.1:188906-188709;PP860028.1:188863-188666;PP860031.1:188904-188707;PP860033.1:188899-188702;PQ153226.1:188969-188772;PQ178862.2:187129-186932;PQ221506.1:182501-182304;PQ221833.1:188622-188425;PQ340477.1:189059-188862;PQ340478.1:188968-188771;PQ340480.1:189090-188893;PQ340483.1:189046-188849;PQ340485.1:189085-188888;PQ340486.1:189144-188947;PQ340487.1:189188-188991;PQ340489.1:189120-188923;PQ340490.1:188978-188781;PQ340491.1:189221-189024;PQ340493.1:189040-188843;PQ340495.1:189101-188904;PQ340496.1:189199-189002;PQ368489.1:189002-188805;PQ451835.1:189163-188966;PQ451836.1:188941-188744;PQ451838.1:189146-188949;PQ451841.1:189057-188860 |
| OG0000012 | AY603973.1:14598-12616;AY741551.1:14433-12451;AY753185.1:14844-12862;DQ011154.1:13088-11106;DQ011155.1:13068-11086;EPI_ISL_13331598:12915-10933;EPI_ISL_13363142:12908-10926;EPI_ISL_13374487:12923-10941;EPI_ISL_13502582:12908-10926;EPI_ISL_13508471:13000-11018;EPI_ISL_13607904:12899-10917;EPI_ISL_13624509:12843-10861;EPI_ISL_13889442:13018-11036;EPI_ISL_13953610:12899-10917;EPI_ISL_14181948:13044-11062;EPI_ISL_14838587:12942-10960;EPI_ISL_15263355:12896-10914;EPI_ISL_15269699:12868-10886;EPI_ISL_15293002:13141-11159;EPI_ISL_15641561:12857-10875;EPI_ISL_15831211:12906-10924;EPI_ISL_15992098:12921-10939;EPI_ISL_16233784:12917-10935;EPI_ISL_16946400:12906-10924;EPI_ISL_17048206:12556-10574;EPI_ISL_17703567:12921-10939;EPI_ISL_17718458:12899-10917;EPI_ISL_18213374:12896-10914;EPI_ISL_18360394:12557-10575;EPI_ISL_18557816:12897-10915;EPI_ISL_18642365:12899-10917;EPI_ISL_18659846:12869-10887;EPI_ISL_18744049:12901-10919;EPI_ISL_19022858:12896-10914;EPI_ISL_19028778:12813-10831;EPI_ISL_19151800:12897-10915;EPI_ISL_19159116:12899-10917;EPI_ISL_19350788:12954-10972;EPI_ISL_19359677:12896-10914;EPI_ISL_19446306:12891-10909;EPI_ISL_19467692:13220-11238;JX878411.1:13933-11951;JX878425.1:13448-11466;JX878426.1:12890-10908;KC257460.1:12869-10887;KJ642612.1:11175-9193;KJ642613.1:12751-10769;KJ642616.1:14415-12433;KJ642618.1:10347-8368;KP849469.1:13258-11276;KP849471.1:13178-11196;LC831698.1:12897-10915;MN648051.1:13118-11136;MN702445.1:6598-4616;MN702446.1:12744-10762;MN702447.1:6110-4128;MN702449.1:6606-4624;MN702452.1:12787-10805;MT903338.1:13062-11080;MT903340.1:12899-10917;MT903341.1:4432-3227;MT903342.1:12914-10932;MT903347.1:14769-12787;ON568298.1:13045-11063;ON622713.1:13130-11148;ON622722.2:12902-10920;ON631963.1:12922-10940;ON649879.1:12732-10750;ON676707.1:12952-10970;ON755039.1:12562-10580;ON808413.1:12902-10920;ON880420.1:13129-11147;ON911481.2:12919-10937;ON918656.1:12934-10952;ON959133.1:13023-11041;ON959136.1:13024-11042;ON983168.1:11598-9616;OP019276.1:12896-10914;OP150924.1:13037-11055;OP160532.1:12862-10880;OP235282.1:12896-10914;OP289782.1:12899-10917;OP331335.1:12916-10934;OP382479.1:12903-10921;OP422341.1:12916-10934;OP434519.1:12895-10913;OP535324.1:12897-10915;OP535333.1:13006-11024;OP536770.1:12898-10916;OP597769.1:12920-10938;OP604566.1:12899-10917;OP612682.1:12825-10843;OP612684.1:12889-10907;OP612686.1:12889-10907;OP696841.1:10200-8218;OP879722.1:12122-10140;OQ419007.1:12132-10150;OQ419012.1:12124-10142;OQ419031.1:12129-10147;OQ419047.1:12132-10150;OQ451898.1:11984-11307;OQ451898.1:11286-10915;OQ451900.1:12897-10915;OQ581853.1:12923-10941;OQ581855.1:13004-11022;OQ721951.1:12937-10955;OQ721954.1:12894-10912;OR035652.1:12846-10864;OR035664.1:12846-10864;OR035702.1:12846-10864;OR146272.1:12862-10880;OR146330.1:12907-10925;OR264364.1:12917-10935;OR427332.1:12917-10935;OR459777.1:12901-10919;OR459797.1:12916-10934;OR943698.1:12925-11747;OR943698.1:11769-10975;PP098589.2:12898-10916;PP496048.1:12902-10920;PP601206.1:12940-10958;PP776553.1:12847-10865;PP852971.1:12896-10914;PP852983.1:12901-10919;PP853000.1:12863-10881;PP853005.1:12871-10889;PP853007.1:12903-10921;PP853013.1:12877-10895;PP853027.1:12908-10926;PP853045.1:12907-10925;PP853048.1:12908-10926;PP860026.1:12903-10921;PP860027.1:12902-10920;PP860028.1:12870-10888;PP860031.1:12909-10927;PP860033.1:12901-10919;PQ153226.1:12896-10914;PQ178862.2:12931-10949;PQ221506.1:6761-4779;PQ221833.1:12932-10950;PQ340477.1:12879-10897;PQ340478.1:12864-10882;PQ340480.1:12943-10961;PQ340483.1:12927-10945;PQ340485.1:12913-10931;PQ340486.1:12928-10946;PQ340487.1:12953-10971;PQ340489.1:12928-10946;PQ340490.1:12879-10897;PQ340491.1:12937-10955;PQ340493.1:12913-10931;PQ340495.1:12943-10961;PQ340496.1:12976-10994;PQ368489.1:12899-10917;PQ451835.1:12987-11005;PQ451836.1:12919-10937;PQ451838.1:12970-10988;PQ451841.1:12930-10948;PQ451842.1:12882-10900 |
| OG0000013 | AY603973.1:20508-19558;AY741551.1:20375-19425;AY741551.1:9812-9630;AY753185.1:20751-19801;DQ011154.1:19020-18073;DQ011155.1:19001-18054;EPI_ISL_13331598:18843-17893;EPI_ISL_13363142:18836-17886;EPI_ISL_13374487:18851-17901;EPI_ISL_13502582:18836-17886;EPI_ISL_13508471:18928-17978;EPI_ISL_13607904:18827-17877;EPI_ISL_13624509:18771-17821;EPI_ISL_13889442:18946-17996;EPI_ISL_13953610:18827-17877;EPI_ISL_14181948:18972-18022;EPI_ISL_14838587:18874-17927;EPI_ISL_15263355:18827-17877;EPI_ISL_15269699:18796-17846;EPI_ISL_15293002:19069-18119;EPI_ISL_15641561:18785-17835;EPI_ISL_15831211:18834-17884;EPI_ISL_15992098:18849-17899;EPI_ISL_16233784:18845-17895;EPI_ISL_16946400:18834-17884;EPI_ISL_17048206:18484-17534;EPI_ISL_17703567:18849-17899;EPI_ISL_17718458:18827-17877;EPI_ISL_18213374:18824-17874;EPI_ISL_18360394:18485-17535;EPI_ISL_18557816:18825-17875;EPI_ISL_18642365:18827-17877;EPI_ISL_18659846:18797-17847;EPI_ISL_18744049:18829-17879;EPI_ISL_19022858:18824-17874;EPI_ISL_19028778:18741-17791;EPI_ISL_19151800:18825-17875;EPI_ISL_19159116:18827-17877;EPI_ISL_19350788:18893-17946;EPI_ISL_19359677:18824-17874;EPI_ISL_19446306:18819-17869;EPI_ISL_19467692:19152-18205;JX878411.1:19866-18919;JX878425.1:19380-18433;JX878426.1:18823-17876;KC257460.1:18800-17853;KJ642612.1:17108-16161;KJ642613.1:18689-17742;KJ642616.1:20325-19375;KJ642618.1:16279-15332;KP849469.1:19190-18243;KP849471.1:19111-18164;LC831698.1:18825-17875;MN648051.1:19047-18097;MN702445.1:12531-11584;MN702446.1:18677-17730;MN702447.1:12042-11095;MN702449.1:12539-11592;MN702452.1:18719-17772;MT903338.1:18990-18040;MT903340.1:18827-17877;MT903341.1:10360-9410;MT903342.1:18842-17892;MT903347.1:20708-19758;ON568298.1:18973-18023;ON622713.1:19059-18109;ON622722.2:18830-17880;ON631963.1:18850-17900;ON649879.1:18660-17710;ON676707.1:18880-17930;ON755039.1:18490-17540;ON808413.1:18830-17880;ON880420.1:19057-18107;ON911481.2:18847-17897;ON918656.1:18862-17912;ON959133.1:18951-18001;ON959136.1:18952-18002;ON983168.1:17526-16576;OP019276.1:18824-17874;OP150924.1:18965-18015;OP160532.1:18790-17840;OP235282.1:18824-17874;OP289782.1:18827-17877;OP331335.1:18844-17894;OP382479.1:18831-17881;OP422341.1:18844-17894;OP434519.1:18823-17873;OP535324.1:18825-17875;OP535333.1:18934-17984;OP536770.1:18826-17876;OP597769.1:18848-17898;OP604566.1:18827-17877;OP612682.1:18753-17803;OP612684.1:18817-17867;OP612686.1:18817-17867;OP696841.1:16101-15151;OP879722.1:18050-17100;OQ419007.1:18060-17110;OQ419012.1:18052-17102;OQ419031.1:18057-17107;OQ419047.1:18060-17110;OQ451898.1:17912-16962;OQ451900.1:18825-17875;OQ581853.1:18851-17901;OQ581855.1:18932-17982;OQ721951.1:18865-17915;OQ721954.1:18822-17872;OR035652.1:18774-17824;OR035664.1:18774-17824;OR035702.1:18774-17824;OR146272.1:18790-17840;OR146330.1:18835-17885;OR264364.1:18845-17895;OR427332.1:18845-17895;OR459777.1:18829-17879;OR459797.1:18844-17894;OR943698.1:18857-17910;PP098589.2:18826-17876;PP496048.1:18830-17880;PP601206.1:18872-17925;PP776553.1:18775-17825;PP852971.1:18824-17874;PP852983.1:18829-17879;PP853000.1:18791-17841;PP853005.1:18799-17849;PP853007.1:18831-17881;PP853013.1:18805-17855;PP853027.1:18836-17886;PP853045.1:18835-17885;PP853048.1:18836-17886;PP860026.1:18831-17881;PP860027.1:18842-17892;PP860028.1:18798-17848;PP860031.1:18852-17902;PP860033.1:18841-17891;PQ153226.1:18824-17874;PQ178862.2:18870-17923;PQ221506.1:12694-11747;PQ221833.1:18864-17917;PQ340477.1:18807-17857;PQ340478.1:18792-17842;PQ340480.1:18871-17921;PQ340483.1:18855-17905;PQ340485.1:18841-17891;PQ340486.1:18856-17906;PQ340487.1:18881-17931;PQ340489.1:18856-17906;PQ340490.1:18807-17857;PQ340491.1:18865-17915;PQ340493.1:18841-17891;PQ340495.1:18871-17921;PQ340496.1:18904-17954;PQ368489.1:18827-17877;PQ451835.1:18915-17965;PQ451836.1:18847-17897;PQ451838.1:18898-17948;PQ451841.1:18858-17908;PQ451842.1:18810-17860 |
| OG0000014 | AY603973.1:24939-25136;AY741551.1:24800-24997;AY753185.1:25182-25379;DQ011154.1:25417-25614;DQ011155.1:25403-25600;EPI_ISL_13331598:23255-23452;EPI_ISL_13363142:23248-23445;EPI_ISL_13374487:23263-23460;EPI_ISL_13502582:23248-23445;EPI_ISL_13508471:23340-23537;EPI_ISL_13607904:23239-23436;EPI_ISL_13624509:23183-23380;EPI_ISL_13889442:23358-23555;EPI_ISL_13953610:23206-23403;EPI_ISL_14181948:23384-23581;EPI_ISL_14838587:25271-25468;EPI_ISL_15263355:23239-23436;EPI_ISL_15269699:23208-23405;EPI_ISL_15293002:23481-23678;EPI_ISL_15641561:23197-23394;EPI_ISL_15831211:23246-23443;EPI_ISL_15992098:23261-23458;EPI_ISL_16233784:23257-23454;EPI_ISL_16946400:23246-23443;EPI_ISL_17048206:22896-23093;EPI_ISL_17703567:23261-23458;EPI_ISL_17718458:23239-23436;EPI_ISL_18213374:23236-23433;EPI_ISL_18360394:22897-23094;EPI_ISL_18557816:23237-23434;EPI_ISL_18642365:23239-23436;EPI_ISL_18659846:23209-23406;EPI_ISL_18744049:23241-23438;EPI_ISL_19022858:23236-23433;EPI_ISL_19028778:23153-23350;EPI_ISL_19151800:23237-23434;EPI_ISL_19159116:23239-23436;EPI_ISL_19350788:24156-24353;EPI_ISL_19359677:23236-23433;EPI_ISL_19446306:23231-23428;EPI_ISL_19467692:25550-25747;JX878411.1:26265-26462;JX878425.1:25779-25976;JX878426.1:25219-25416;KC257460.1:25200-25397;KJ642612.1:23505-23702;KJ642613.1:25086-25283;KJ642616.1:24755-24952;KJ642618.1:22673-22870;KP849469.1:25587-25784;KP849471.1:25508-25705;LC831698.1:23237-23434;MN648051.1:23459-23656;MN702445.1:18932-19129;MN702446.1:25078-25275;MN702447.1:18443-18640;MN702449.1:18943-19140;MN702452.1:25116-25313;MT903338.1:23402-23599;MT903340.1:23239-23436;MT903341.1:14772-14969;MT903342.1:23254-23451;MT903347.1:25132-25329;ON568298.1:23385-23582;ON622713.1:23471-23668;ON622722.2:23242-23439;ON631963.1:23262-23459;ON649879.1:23072-23269;ON676707.1:23292-23489;ON755039.1:22902-23099;ON808413.1:23242-23439;ON880420.1:23469-23666;ON911481.2:23259-23456;ON918656.1:23274-23471;ON959133.1:23363-23560;ON959136.1:23364-23561;ON983168.1:21938-22135;OP019276.1:23236-23433;OP150924.1:23377-23574;OP160532.1:23202-23399;OP235282.1:23236-23433;OP289782.1:23239-23436;OP331335.1:23256-23453;OP382479.1:23243-23440;OP422341.1:23256-23453;OP434519.1:23235-23432;OP535324.1:23237-23434;OP535333.1:23346-23543;OP536770.1:23238-23435;OP597769.1:23260-23457;OP604566.1:23239-23436;OP612682.1:23165-23362;OP612684.1:23229-23426;OP612686.1:23229-23426;OP696841.1:20513-20710;OP879722.1:22462-22659;OQ419007.1:22472-22669;OQ419012.1:22464-22661;OQ419031.1:22469-22666;OQ419047.1:22472-22669;OQ451898.1:22324-22521;OQ451900.1:23237-23434;OQ581853.1:23263-23460;OQ581855.1:23344-23541;OQ721951.1:23277-23474;OQ721954.1:23234-23431;OR035652.1:23186-23383;OR035664.1:23186-23383;OR035702.1:23186-23383;OR146272.1:23202-23399;OR146330.1:23247-23444;OR264364.1:23257-23454;OR427332.1:23257-23454;OR459777.1:23241-23438;OR459797.1:23256-23453;OR943698.1:25247-25444;PP098589.2:23238-23435;PP496048.1:23242-23439;PP601206.1:25269-25466;PP776553.1:23187-23384;PP852971.1:23236-23433;PP852983.1:23241-23438;PP853000.1:23203-23400;PP853005.1:23211-23408;PP853007.1:23243-23440;PP853013.1:23217-23414;PP853027.1:23248-23445;PP853045.1:23229-23426;PP853048.1:23248-23445;PP860026.1:23243-23440;PP860027.1:23254-23451;PP860028.1:23210-23407;PP860031.1:23264-23461;PP860033.1:23253-23450;PQ153226.1:23236-23433;PQ178862.2:24133-24330;PQ221506.1:19089-19286;PQ221833.1:25261-25458;PQ340477.1:23219-23416;PQ340478.1:23204-23401;PQ340480.1:23283-23480;PQ340483.1:23267-23464;PQ340485.1:23253-23450;PQ340486.1:23268-23465;PQ340487.1:23293-23490;PQ340489.1:23268-23465;PQ340490.1:23219-23416;PQ340491.1:23277-23474;PQ340493.1:23253-23450;PQ340495.1:23283-23480;PQ340496.1:23316-23513;PQ368489.1:23239-23436;PQ451835.1:23327-23524;PQ451836.1:23259-23456;PQ451838.1:23310-23507;PQ451841.1:23270-23467;PQ451842.1:23222-23419 |
| OG0000015 | AY603973.1:28911-29129;AY741551.1:28925-29143;AY753185.1:29154-29372;DQ011154.1:29160-29378;DQ011155.1:29146-29364;EPI_ISL_13331598:26997-27215;EPI_ISL_13363142:26990-27208;EPI_ISL_13374487:27005-27223;EPI_ISL_13502582:26990-27208;EPI_ISL_13508471:27082-27300;EPI_ISL_13607904:26981-27199;EPI_ISL_13624509:26925-27143;EPI_ISL_13889442:27100-27318;EPI_ISL_13953610:26948-27166;EPI_ISL_14181948:27126-27344;EPI_ISL_14838587:29014-29232;EPI_ISL_15263355:26981-27199;EPI_ISL_15269699:26950-27168;EPI_ISL_15293002:27223-27441;EPI_ISL_15641561:26939-27157;EPI_ISL_15831211:26988-27206;EPI_ISL_15992098:27003-27221;EPI_ISL_16233784:26999-27217;EPI_ISL_16946400:26988-27206;EPI_ISL_17048206:26638-26856;EPI_ISL_17703567:27003-27221;EPI_ISL_17718458:26981-27199;EPI_ISL_18213374:26978-27196;EPI_ISL_18360394:26639-26857;EPI_ISL_18557816:26979-27197;EPI_ISL_18642365:26981-27199;EPI_ISL_18659846:26951-27169;EPI_ISL_18744049:26983-27201;EPI_ISL_19022858:26978-27196;EPI_ISL_19028778:26895-27113;EPI_ISL_19151800:26979-27197;EPI_ISL_19159116:26981-27199;EPI_ISL_19350788:27899-28117;EPI_ISL_19359677:26978-27196;EPI_ISL_19446306:26973-27191;EPI_ISL_19467692:29293-29511;JX878411.1:30008-30226;JX878425.1:29522-29740;JX878426.1:28962-29180;KC257460.1:28943-29161;KJ642612.1:27248-27466;KJ642613.1:28829-29047;KJ642616.1:28610-28828;KJ642618.1:26416-26634;KP849469.1:29330-29548;KP849471.1:29251-29469;LC831698.1:26979-27197;MN648051.1:27201-27419;MN702445.1:22675-22893;MN702446.1:28821-29039;MN702447.1:22186-22404;MN702449.1:22686-22904;MN702452.1:28859-29077;MT903338.1:27144-27362;MT903340.1:26981-27199;MT903341.1:18514-18732;MT903342.1:26996-27214;MT903347.1:28909-29127;ON568298.1:27127-27345;ON622713.1:27213-27431;ON622722.2:26984-27202;ON631963.1:27004-27222;ON649879.1:26814-27032;ON676707.1:27034-27252;ON755039.1:26644-26862;ON808413.1:26984-27202;ON880420.1:27211-27429;ON911481.2:27001-27219;ON918656.1:27016-27234;ON959133.1:27105-27323;ON959136.1:27106-27324;ON983168.1:25680-25898;OP019276.1:26978-27196;OP150924.1:27119-27337;OP160532.1:26944-27162;OP235282.1:26978-27196;OP289782.1:26981-27199;OP331335.1:26998-27216;OP382479.1:26985-27203;OP422341.1:26998-27216;OP434519.1:26977-27195;OP535324.1:26979-27197;OP535333.1:27088-27306;OP536770.1:26980-27198;OP597769.1:27002-27220;OP604566.1:26981-27199;OP612682.1:26907-27125;OP612684.1:26971-27189;OP612686.1:26971-27189;OP696841.1:24255-24473;OP879722.1:26204-26422;OQ419007.1:26214-26432;OQ419012.1:26206-26424;OQ419031.1:26185-26403;OQ419047.1:26214-26432;OQ451898.1:26066-26284;OQ451900.1:26979-27197;OQ581853.1:27005-27223;OQ581855.1:27086-27304;OQ721951.1:27019-27237;OQ721954.1:26976-27194;OR035652.1:26928-27146;OR035664.1:26928-27146;OR035702.1:26928-27146;OR146272.1:26944-27162;OR146330.1:26989-27207;OR264364.1:26999-27217;OR427332.1:26999-27217;OR459777.1:26983-27201;OR459797.1:26998-27216;OR943698.1:28990-29208;PP098589.2:26980-27198;PP496048.1:26984-27202;PP601206.1:29012-29230;PP776553.1:26905-27123;PP852971.1:26978-27196;PP852983.1:26983-27201;PP853000.1:26945-27163;PP853005.1:26953-27171;PP853007.1:26985-27203;PP853013.1:26959-27177;PP853027.1:26990-27208;PP853045.1:26971-27189;PP853048.1:26990-27208;PP860026.1:26985-27203;PP860027.1:26996-27214;PP860028.1:26952-27170;PP860031.1:27006-27224;PP860033.1:26995-27213;PQ153226.1:26978-27196;PQ178862.2:27876-28094;PQ221506.1:22832-23050;PQ221833.1:29004-29222;PQ340477.1:26961-27179;PQ340478.1:26946-27164;PQ340480.1:27025-27243;PQ340483.1:27009-27227;PQ340485.1:26995-27213;PQ340486.1:27010-27228;PQ340487.1:27035-27253;PQ340489.1:27010-27228;PQ340490.1:26961-27179;PQ340491.1:27019-27237;PQ340493.1:26995-27213;PQ340495.1:27025-27243;PQ340496.1:27058-27276;PQ368489.1:26981-27199;PQ451835.1:27069-27287;PQ451836.1:27001-27219;PQ451838.1:27052-27270;PQ451841.1:27012-27230;PQ451842.1:26964-27182 |
| OG0000016 | AY603973.1:42423-42581;AY741551.1:42427-42585;AY753185.1:42666-42824;DQ011154.1:42642-42800;DQ011155.1:42623-42781;EPI_ISL_13331598:40494-40652;EPI_ISL_13363142:40487-40645;EPI_ISL_13374487:40502-40660;EPI_ISL_13502582:40487-40645;EPI_ISL_13508471:40579-40737;EPI_ISL_13607904:40478-40636;EPI_ISL_13624509:40422-40580;EPI_ISL_13889442:40597-40755;EPI_ISL_13953610:40445-40603;EPI_ISL_14181948:40623-40781;EPI_ISL_14838587:42496-42654;EPI_ISL_15263355:40478-40636;EPI_ISL_15269699:40447-40605;EPI_ISL_15293002:40720-40878;EPI_ISL_15641561:40436-40594;EPI_ISL_15831211:40485-40643;EPI_ISL_15992098:40500-40658;EPI_ISL_16233784:40496-40654;EPI_ISL_16946400:40485-40643;EPI_ISL_17048206:40135-40293;EPI_ISL_17703567:40500-40658;EPI_ISL_17718458:40478-40636;EPI_ISL_18213374:40475-40633;EPI_ISL_18360394:40136-40294;EPI_ISL_18557816:40476-40634;EPI_ISL_18642365:40478-40636;EPI_ISL_18659846:40448-40606;EPI_ISL_18744049:40480-40638;EPI_ISL_19022858:40475-40633;EPI_ISL_19028778:40392-40550;EPI_ISL_19151800:40476-40634;EPI_ISL_19159116:40478-40636;EPI_ISL_19350788:41370-41528;EPI_ISL_19359677:40475-40633;EPI_ISL_19446306:40470-40628;EPI_ISL_19467692:42775-42933;JX878411.1:43490-43648;JX878425.1:43004-43162;JX878426.1:42449-42607;KC257460.1:42420-42578;KJ642612.1:40735-40893;KJ642613.1:42311-42469;KJ642616.1:42117-42275;KJ642618.1:39903-40061;KP849469.1:42812-42970;KP849471.1:42738-42896;LC831698.1:40476-40634;MN648051.1:40698-40856;MN702445.1:36157-36315;MN702446.1:42302-42460;MN702447.1:35668-35826;MN702449.1:36168-36326;MN702452.1:42341-42499;MT903338.1:40641-40799;MT903340.1:40478-40636;MT903341.1:32011-32169;MT903342.1:40493-40651;MT903347.1:42436-42594;ON568298.1:40624-40782;ON622713.1:40710-40868;ON622722.2:40481-40639;ON631963.1:40501-40659;ON649879.1:40311-40469;ON676707.1:40531-40689;ON755039.1:40141-40299;ON808413.1:40481-40639;ON880420.1:40708-40866;ON911481.2:40498-40656;ON918656.1:40513-40671;ON959133.1:40602-40760;ON959136.1:40603-40761;ON983168.1:39177-39335;OP019276.1:40475-40633;OP150924.1:40616-40774;OP160532.1:40441-40599;OP235282.1:40475-40633;OP289782.1:40478-40636;OP331335.1:40495-40653;OP382479.1:40482-40640;OP422341.1:40495-40653;OP434519.1:40474-40632;OP535324.1:40476-40634;OP535333.1:40585-40743;OP536770.1:40477-40635;OP597769.1:40499-40657;OP604566.1:40478-40636;OP612682.1:40404-40562;OP612684.1:40468-40626;OP612686.1:40468-40626;OP696841.1:37752-37910;OP879722.1:39701-39859;OQ419007.1:39711-39869;OQ419012.1:39700-39858;OQ419031.1:39682-39840;OQ419047.1:39711-39869;OQ451898.1:39563-39721;OQ451900.1:40476-40634;OQ581853.1:40502-40660;OQ581855.1:40583-40741;OQ721951.1:40516-40674;OQ721954.1:40473-40631;OR035652.1:40425-40583;OR035664.1:40425-40583;OR035702.1:40425-40583;OR146272.1:40441-40599;OR146330.1:40486-40644;OR264364.1:40496-40654;OR427332.1:40496-40654;OR459777.1:40480-40638;OR459797.1:40495-40653;OR943698.1:42477-42635;PP098589.2:40477-40635;PP496048.1:40481-40639;PP601206.1:42494-42652;PP776553.1:40402-40560;PP852971.1:40475-40633;PP852983.1:40481-40639;PP853000.1:40442-40600;PP853005.1:40450-40608;PP853007.1:40483-40641;PP853013.1:40456-40614;PP853027.1:40487-40645;PP853045.1:40468-40626;PP853048.1:40487-40645;PP860026.1:40485-40643;PP860027.1:40510-40668;PP860028.1:40451-40609;PP860031.1:40504-40662;PP860033.1:40509-40667;PQ153226.1:40475-40633;PQ178862.2:41347-41505;PQ221506.1:36317-36475;PQ221833.1:42486-42644;PQ340477.1:40458-40616;PQ340478.1:40443-40601;PQ340480.1:40522-40680;PQ340483.1:40506-40664;PQ340485.1:40492-40650;PQ340486.1:40507-40665;PQ340487.1:40532-40690;PQ340489.1:40507-40665;PQ340490.1:40458-40616;PQ340491.1:40513-40671;PQ340493.1:40492-40650;PQ340495.1:40522-40680;PQ340496.1:40555-40713;PQ368489.1:40478-40636;PQ451835.1:40563-40721;PQ451836.1:40495-40653;PQ451838.1:40546-40704;PQ451841.1:40506-40664;PQ451842.1:40458-40616 |
| OG0000017 | AY603973.1:50145-51848;AY741551.1:50149-51852;AY753185.1:50388-52091;DQ011154.1:50609-52312;DQ011155.1:50587-52290;EPI_ISL_13331598:48216-49919;EPI_ISL_13363142:48209-49912;EPI_ISL_13374487:48224-49927;EPI_ISL_13502582:48209-49912;EPI_ISL_13508471:48301-50004;EPI_ISL_13607904:48200-49903;EPI_ISL_13624509:48144-49847;EPI_ISL_13889442:48319-50022;EPI_ISL_13953610:48160-49863;EPI_ISL_14181948:48345-50048;EPI_ISL_14838587:50463-52166;EPI_ISL_15263355:48200-49903;EPI_ISL_15269699:48169-49872;EPI_ISL_15293002:48442-50145;EPI_ISL_15641561:48158-49861;EPI_ISL_15831211:48207-49910;EPI_ISL_15992098:48222-49925;EPI_ISL_16233784:48218-49921;EPI_ISL_16946400:48207-49910;EPI_ISL_17048206:47857-49560;EPI_ISL_17703567:48222-49925;EPI_ISL_17718458:48200-49903;EPI_ISL_18213374:48197-49900;EPI_ISL_18360394:47858-49561;EPI_ISL_18557816:48198-49901;EPI_ISL_18642365:48200-49903;EPI_ISL_18659846:48170-49873;EPI_ISL_18744049:48202-49905;EPI_ISL_19022858:48197-49900;EPI_ISL_19028778:48114-49817;EPI_ISL_19151800:48198-49901;EPI_ISL_19159116:48200-49903;EPI_ISL_19350788:49335-51038;EPI_ISL_19359677:48197-49900;EPI_ISL_19446306:48192-49895;EPI_ISL_19467692:50746-52449;JX878411.1:51454-53157;JX878425.1:50971-52674;JX878426.1:50420-52123;KC257460.1:50385-52088;KJ642612.1:48706-50409;KJ642613.1:50279-51982;KJ642616.1:49839-51542;KJ642618.1:47873-49576;KP849469.1:50779-52482;KP849471.1:50704-52407;LC831698.1:48198-49901;MN648051.1:48420-50123;MN702445.1:44122-45825;MN702446.1:50267-51970;MN702447.1:43635-45338;MN702449.1:44133-45836;MN702452.1:50308-52011;MT903338.1:48363-50066;MT903340.1:48200-49903;MT903341.1:39733-41436;MT903342.1:48215-49918;MT903347.1:50156-51859;ON568298.1:48346-50049;ON622713.1:48432-50135;ON622722.2:48203-49906;ON631963.1:48223-49926;ON649879.1:48033-49736;ON676707.1:48253-49956;ON755039.1:47863-49566;ON808413.1:48203-49906;ON880420.1:48430-50133;ON911481.2:48220-49923;ON918656.1:48235-49938;ON959133.1:48324-50027;ON959136.1:48325-50028;ON983168.1:46899-48602;OP019276.1:48197-49900;OP150924.1:48338-50041;OP160532.1:48163-49866;OP235282.1:48197-49900;OP289782.1:48200-49903;OP331335.1:48217-49920;OP382479.1:48204-49907;OP422341.1:48217-49920;OP434519.1:48196-49899;OP535324.1:48198-49901;OP535333.1:48307-50010;OP536770.1:48199-49902;OP597769.1:48221-49924;OP604566.1:48200-49903;OP612682.1:48126-49829;OP612684.1:48190-49893;OP612686.1:48190-49893;OP696841.1:45474-47177;OP879722.1:47423-49126;OQ419007.1:47433-49136;OQ419012.1:47422-49125;OQ419031.1:47404-49107;OQ419047.1:47433-49136;OQ451898.1:47285-48988;OQ451900.1:48198-49901;OQ581853.1:48224-49927;OQ581855.1:48305-50008;OQ721951.1:48238-49941;OQ721954.1:48195-49898;OR035652.1:48147-49850;OR035664.1:48147-49850;OR035702.1:48147-49850;OR146272.1:48163-49866;OR146330.1:48208-49911;OR264364.1:48218-49921;OR427332.1:48218-49921;OR459777.1:48202-49905;OR459797.1:48217-49920;OR943698.1:50449-52152;PP098589.2:48199-49902;PP496048.1:48203-49906;PP601206.1:50461-52164;PP776553.1:48124-49827;PP852971.1:48197-49900;PP852983.1:48203-49906;PP853000.1:48164-49867;PP853005.1:48172-49875;PP853007.1:48205-49908;PP853013.1:48178-49881;PP853027.1:48209-49912;PP853045.1:48190-49893;PP853048.1:48209-49912;PP860026.1:48207-49910;PP860027.1:48232-49935;PP860028.1:48173-49876;PP860031.1:48226-49929;PP860033.1:48231-49934;PQ153226.1:48197-49900;PQ178862.2:49312-51015;PQ221506.1:44288-45991;PQ221833.1:50454-52157;PQ340477.1:48180-49883;PQ340478.1:48165-49868;PQ340480.1:48244-49947;PQ340483.1:48228-49931;PQ340485.1:48214-49917;PQ340486.1:48229-49932;PQ340487.1:48254-49957;PQ340489.1:48229-49932;PQ340490.1:48180-49883;PQ340491.1:48235-49938;PQ340493.1:48214-49917;PQ340495.1:48244-49947;PQ340496.1:48277-49980;PQ368489.1:48200-49903;PQ451835.1:48285-49988;PQ451836.1:48217-49920;PQ451838.1:48268-49971;PQ451841.1:48228-49931;PQ451842.1:48180-49883 |
| OG0000018 | AY603973.1:51930-52430;AY741551.1:51934-52434;AY753185.1:52173-52673;DQ011154.1:52394-52894;DQ011155.1:52372-52872;EPI_ISL_13331598:50001-50501;EPI_ISL_13363142:49994-50494;EPI_ISL_13374487:50009-50509;EPI_ISL_13502582:49994-50494;EPI_ISL_13508471:50086-50586;EPI_ISL_13607904:49985-50485;EPI_ISL_13624509:49929-50429;EPI_ISL_13889442:50104-50604;EPI_ISL_13953610:49945-50445;EPI_ISL_14181948:50130-50630;EPI_ISL_14838587:52248-52748;EPI_ISL_15263355:49985-50485;EPI_ISL_15269699:49954-50454;EPI_ISL_15293002:50227-50727;EPI_ISL_15641561:49943-50443;EPI_ISL_15831211:49992-50492;EPI_ISL_15992098:50007-50507;EPI_ISL_16233784:50003-50503;EPI_ISL_16946400:49992-50492;EPI_ISL_17048206:49642-50142;EPI_ISL_17703567:50007-50507;EPI_ISL_17718458:49985-50485;EPI_ISL_18213374:49982-50482;EPI_ISL_18360394:49643-50143;EPI_ISL_18557816:49983-50483;EPI_ISL_18642365:49985-50485;EPI_ISL_18659846:49955-50455;EPI_ISL_18744049:49987-50487;EPI_ISL_19022858:49982-50482;EPI_ISL_19028778:49899-50399;EPI_ISL_19151800:49983-50483;EPI_ISL_19159116:49985-50485;EPI_ISL_19350788:51120-51620;EPI_ISL_19359677:49982-50482;EPI_ISL_19446306:49977-50477;EPI_ISL_19467692:52531-53031;JX878411.1:53239-53739;JX878425.1:52756-53256;JX878426.1:52205-52705;KC257460.1:52170-52670;KJ642612.1:50491-50991;KJ642613.1:52064-52564;KJ642616.1:51624-52124;KJ642618.1:49658-50158;KP849469.1:52564-53064;KP849471.1:52489-52989;LC831698.1:49983-50483;MN648051.1:50205-50705;MN702445.1:45907-46407;MN702446.1:52052-52552;MN702447.1:45420-45920;MN702449.1:45918-46418;MN702452.1:52093-52593;MT903338.1:50148-50648;MT903340.1:49985-50485;MT903341.1:41518-42018;MT903342.1:50000-50500;MT903347.1:51941-52441;ON568298.1:50131-50631;ON622713.1:50217-50717;ON622722.2:49988-50488;ON631963.1:50008-50508;ON649879.1:49818-50318;ON676707.1:50038-50538;ON755039.1:49648-50148;ON808413.1:49988-50488;ON880420.1:50215-50715;ON911481.2:50005-50505;ON918656.1:50020-50520;ON959133.1:50109-50609;ON959136.1:50110-50610;ON983168.1:48684-49184;OP019276.1:49982-50482;OP150924.1:50123-50623;OP160532.1:49948-50448;OP235282.1:49982-50482;OP289782.1:49985-50485;OP331335.1:50002-50502;OP382479.1:49989-50489;OP422341.1:50002-50502;OP434519.1:49981-50481;OP535324.1:49983-50483;OP535333.1:50092-50592;OP536770.1:49984-50484;OP597769.1:50006-50506;OP604566.1:49985-50485;OP612682.1:49911-50411;OP612684.1:49975-50475;OP612686.1:49975-50475;OP696841.1:47259-47759;OP879722.1:49208-49708;OQ419007.1:49218-49718;OQ419012.1:49207-49620;OQ419031.1:49189-49689;OQ419047.1:49218-49718;OQ451898.1:49070-49570;OQ451900.1:49983-50483;OQ581853.1:50009-50509;OQ581855.1:50090-50590;OQ721951.1:50023-50523;OQ721954.1:49980-50480;OR035652.1:49932-50432;OR035664.1:49932-50432;OR035702.1:49932-50432;OR146272.1:49948-50448;OR146330.1:49993-50493;OR264364.1:50003-50503;OR427332.1:50003-50503;OR459777.1:49987-50487;OR459797.1:50002-50502;OR943698.1:52234-52734;PP098589.2:49984-50484;PP496048.1:49988-50488;PP601206.1:52246-52746;PP776553.1:49909-50409;PP852971.1:49982-50482;PP852983.1:49988-50488;PP853000.1:49949-50449;PP853005.1:49957-50457;PP853007.1:49990-50490;PP853013.1:49963-50463;PP853027.1:49994-50494;PP853045.1:49975-50475;PP853048.1:49994-50494;PP860026.1:49992-50492;PP860027.1:50017-50517;PP860028.1:49958-50458;PP860031.1:50011-50511;PP860033.1:50016-50516;PQ153226.1:49982-50482;PQ178862.2:51097-51597;PQ221506.1:46073-46573;PQ221833.1:52239-52739;PQ340477.1:49965-50465;PQ340478.1:49950-50450;PQ340480.1:50029-50529;PQ340483.1:50013-50513;PQ340485.1:49999-50499;PQ340486.1:50014-50514;PQ340487.1:50039-50539;PQ340489.1:50014-50514;PQ340490.1:49965-50465;PQ340491.1:50020-50520;PQ340493.1:49999-50499;PQ340495.1:50029-50529;PQ340496.1:50062-50562;PQ368489.1:49985-50485;PQ451835.1:50070-50570;PQ451836.1:50002-50502;PQ451838.1:50053-50553;PQ451841.1:50013-50513;PQ451842.1:49965-50465 |
| OG0000019 | AY603973.1:71811-73115;AY741551.1:71815-73119;AY753185.1:72054-73358;DQ011154.1:72272-73576;DQ011155.1:72250-73554;EPI_ISL_13331598:69881-71185;EPI_ISL_13363142:69874-71178;EPI_ISL_13374487:69889-71193;EPI_ISL_13502582:69874-71178;EPI_ISL_13508471:69966-71270;EPI_ISL_13607904:69865-71169;EPI_ISL_13624509:69809-71113;EPI_ISL_13889442:69984-71288;EPI_ISL_13953610:69801-71105;EPI_ISL_14181948:70010-71314;EPI_ISL_14838587:72126-73430;EPI_ISL_15263355:69865-71169;EPI_ISL_15269699:69834-71138;EPI_ISL_15293002:70107-71411;EPI_ISL_15641561:69823-71127;EPI_ISL_15831211:69872-71176;EPI_ISL_15992098:69887-71191;EPI_ISL_16233784:69883-71187;EPI_ISL_16946400:69872-71176;EPI_ISL_17048206:69522-70826;EPI_ISL_17703567:69887-71191;EPI_ISL_17718458:69865-71169;EPI_ISL_18213374:69862-71166;EPI_ISL_18360394:69523-70827;EPI_ISL_18557816:69863-71167;EPI_ISL_18642365:69865-71169;EPI_ISL_18659846:69835-71139;EPI_ISL_18744049:69867-71171;EPI_ISL_19022858:69862-71166;EPI_ISL_19028778:69779-71083;EPI_ISL_19151800:69863-71167;EPI_ISL_19159116:69865-71169;EPI_ISL_19350788:70998-72302;EPI_ISL_19359677:69862-71166;EPI_ISL_19446306:69857-71161;EPI_ISL_19467692:72409-73713;JX878411.1:73117-74421;JX878425.1:72634-73938;JX878426.1:72083-73387;KC257460.1:72048-73352;KJ642612.1:70369-71673;KJ642613.1:71928-73232;KJ642616.1:71505-72809;KJ642618.1:69536-70840;KP849469.1:72442-73746;KP849471.1:72367-73671;LC831698.1:69863-71167;MN648051.1:70085-71389;MN702445.1:65785-67089;MN702446.1:71930-73234;MN702447.1:65298-66602;MN702449.1:65796-67100;MN702452.1:71971-73275;MT903338.1:70028-71332;MT903340.1:69865-71169;MT903341.1:61398-62702;MT903342.1:69880-71184;MT903347.1:71822-73126;ON568298.1:70011-71315;ON622713.1:70097-71401;ON622722.2:69868-71172;ON631963.1:69888-71192;ON649879.1:69698-71002;ON676707.1:69918-71222;ON755039.1:69528-70832;ON808413.1:69868-71172;ON880420.1:70095-71399;ON911481.2:69885-71189;ON918656.1:69900-71204;ON959133.1:69989-71293;ON959136.1:69990-71294;ON983168.1:68564-69868;OP019276.1:69862-71166;OP150924.1:70003-71307;OP160532.1:69828-71132;OP235282.1:69862-71166;OP289782.1:69865-71169;OP331335.1:69882-71186;OP382479.1:69869-71173;OP422341.1:69882-71186;OP434519.1:69861-71165;OP535324.1:69863-71167;OP535333.1:69972-71276;OP536770.1:69864-71168;OP597769.1:69886-71190;OP604566.1:69865-71169;OP612682.1:69791-71095;OP612684.1:69855-71159;OP612686.1:69855-71159;OP696841.1:67139-68443;OP879722.1:69088-70392;OQ419007.1:69098-70402;OQ419012.1:69065-70369;OQ419031.1:69069-70373;OQ419047.1:69071-70375;OQ451898.1:68950-70254;OQ451900.1:69863-71167;OQ581853.1:69889-71193;OQ581855.1:69970-71274;OQ721951.1:69903-71207;OQ721954.1:69860-71164;OR035652.1:69812-71116;OR035664.1:69812-71116;OR035702.1:69812-71116;OR146272.1:69828-71132;OR146330.1:69873-71177;OR264364.1:69883-71187;OR427332.1:69883-71187;OR459777.1:69867-71171;OR459797.1:69882-71186;OR943698.1:72112-73416;PP098589.2:69864-71168;PP496048.1:69868-71172;PP601206.1:72124-73428;PP776553.1:69789-71093;PP852971.1:69862-71166;PP852983.1:69868-71172;PP853000.1:69829-71133;PP853005.1:69837-71141;PP853007.1:69870-71174;PP853013.1:69843-71147;PP853027.1:69874-71178;PP853045.1:69855-71159;PP853048.1:69874-71178;PP860026.1:69872-71176;PP860027.1:69897-71201;PP860028.1:69837-71141;PP860031.1:69891-71195;PP860033.1:69896-71200;PQ153226.1:69862-71166;PQ178862.2:70975-72279;PQ221506.1:65951-67255;PQ221833.1:72117-73421;PQ340477.1:69845-71149;PQ340478.1:69830-71134;PQ340480.1:69909-71213;PQ340483.1:69893-71197;PQ340485.1:69879-71183;PQ340486.1:69894-71198;PQ340487.1:69919-71223;PQ340489.1:69894-71198;PQ340490.1:69845-71149;PQ340491.1:69900-71204;PQ340493.1:69879-71183;PQ340495.1:69909-71213;PQ340496.1:69942-71246;PQ368489.1:69865-71169;PQ451835.1:69950-71254;PQ451836.1:69882-71186;PQ451838.1:69933-71237;PQ451841.1:69893-71197;PQ451842.1:69845-71149 |
| OG0000020 | AY603973.1:77532-77810;AY741551.1:77536-77814;AY753185.1:77775-78053;DQ011154.1:77993-78271;DQ011155.1:77974-78252;EPI_ISL_13331598:75602-75880;EPI_ISL_13363142:75595-75873;EPI_ISL_13374487:75610-75888;EPI_ISL_13502582:75595-75873;EPI_ISL_13508471:75687-75965;EPI_ISL_13607904:75586-75864;EPI_ISL_13624509:75530-75808;EPI_ISL_13889442:75705-75983;EPI_ISL_13953610:75522-75800;EPI_ISL_14181948:75731-76009;EPI_ISL_14838587:77847-78125;EPI_ISL_15263355:75586-75864;EPI_ISL_15269699:75555-75833;EPI_ISL_15293002:75828-76106;EPI_ISL_15641561:75544-75822;EPI_ISL_15831211:75593-75871;EPI_ISL_15992098:75608-75886;EPI_ISL_16233784:75604-75882;EPI_ISL_16946400:75593-75871;EPI_ISL_17048206:75243-75521;EPI_ISL_17703567:75608-75886;EPI_ISL_17718458:75586-75864;EPI_ISL_18213374:75583-75861;EPI_ISL_18360394:75244-75522;EPI_ISL_18557816:75584-75862;EPI_ISL_18642365:75586-75864;EPI_ISL_18659846:75556-75834;EPI_ISL_18744049:75588-75866;EPI_ISL_19022858:75583-75861;EPI_ISL_19028778:75500-75778;EPI_ISL_19151800:75584-75862;EPI_ISL_19159116:75586-75864;EPI_ISL_19350788:76719-76997;EPI_ISL_19359677:75583-75861;EPI_ISL_19446306:75578-75856;EPI_ISL_19467692:78130-78408;JX878411.1:78838-79116;JX878425.1:78355-78633;JX878426.1:77804-78082;KC257460.1:77772-78050;KJ642612.1:76090-76368;KJ642613.1:77649-77927;KJ642616.1:77226-77504;KJ642618.1:75257-75535;KP849469.1:78163-78441;KP849471.1:78089-78367;LC831698.1:75584-75862;MN648051.1:75806-76084;MN702445.1:71506-71784;MN702446.1:77651-77929;MN702447.1:71019-71297;MN702449.1:71517-71795;MN702452.1:77692-77970;MT903338.1:75749-76027;MT903340.1:75586-75864;MT903341.1:67119-67397;MT903342.1:75601-75879;MT903347.1:77543-77821;ON568298.1:75732-76010;ON622713.1:75818-76096;ON622722.2:75589-75867;ON631963.1:75609-75887;ON649879.1:75419-75697;ON676707.1:75639-75917;ON755039.1:75249-75527;ON808413.1:75589-75867;ON880420.1:75816-76094;ON911481.2:75606-75884;ON918656.1:75621-75899;ON959133.1:75710-75988;ON959136.1:75711-75989;ON983168.1:74285-74563;OP019276.1:75583-75861;OP150924.1:75724-76002;OP160532.1:75549-75827;OP235282.1:75583-75861;OP289782.1:75586-75864;OP331335.1:75603-75881;OP382479.1:75590-75868;OP422341.1:75603-75881;OP434519.1:75582-75860;OP535324.1:75584-75862;OP535333.1:75693-75971;OP536770.1:75585-75863;OP597769.1:75607-75885;OP604566.1:75586-75864;OP612682.1:75512-75790;OP612684.1:75576-75854;OP612686.1:75576-75854;OP696841.1:72860-73138;OP879722.1:74809-75087;OQ419007.1:74819-75097;OQ419012.1:74786-75064;OQ419031.1:74790-75068;OQ419047.1:74792-75070;OQ451898.1:74671-74949;OQ451900.1:75584-75862;OQ581853.1:75610-75888;OQ581855.1:75691-75969;OQ721951.1:75624-75902;OQ721954.1:75581-75859;OR035652.1:75533-75811;OR035664.1:75533-75811;OR035702.1:75533-75811;OR146272.1:75549-75827;OR146330.1:75594-75872;OR264364.1:75604-75882;OR427332.1:75604-75882;OR459777.1:75588-75866;OR459797.1:75603-75881;OR943698.1:77833-78111;PP098589.2:75585-75863;PP496048.1:75589-75867;PP601206.1:77845-78123;PP776553.1:75510-75788;PP852971.1:75583-75861;PP852983.1:75589-75867;PP853000.1:75550-75828;PP853005.1:75558-75836;PP853007.1:75591-75869;PP853013.1:75564-75842;PP853027.1:75595-75873;PP853045.1:75576-75854;PP853048.1:75595-75873;PP860026.1:75593-75871;PP860027.1:75618-75896;PP860028.1:75558-75836;PP860031.1:75612-75890;PP860033.1:75617-75895;PQ153226.1:75583-75861;PQ178862.2:76696-76974;PQ221506.1:71672-71950;PQ221833.1:77838-78116;PQ340477.1:75566-75844;PQ340478.1:75551-75829;PQ340480.1:75630-75908;PQ340483.1:75614-75892;PQ340485.1:75600-75878;PQ340486.1:75615-75893;PQ340487.1:75640-75918;PQ340489.1:75615-75893;PQ340490.1:75566-75844;PQ340491.1:75621-75899;PQ340493.1:75600-75878;PQ340495.1:75630-75908;PQ340496.1:75663-75941;PQ368489.1:75586-75864;PQ451835.1:75671-75949;PQ451836.1:75603-75881;PQ451838.1:75654-75932;PQ451841.1:75614-75892;PQ451842.1:75566-75844 |
| OG0000021 | AY603973.1:79953-80411;AY741551.1:79957-80415;AY753185.1:80196-80654;DQ011154.1:80414-80872;DQ011155.1:80395-80853;EPI_ISL_13331598:78023-78481;EPI_ISL_13363142:78016-78474;EPI_ISL_13374487:78031-78489;EPI_ISL_13502582:78016-78474;EPI_ISL_13508471:78108-78566;EPI_ISL_13607904:78007-78465;EPI_ISL_13624509:77951-78409;EPI_ISL_13889442:78126-78584;EPI_ISL_13953610:77943-78401;EPI_ISL_14181948:78152-78610;EPI_ISL_14838587:80268-80726;EPI_ISL_15263355:78007-78465;EPI_ISL_15269699:77976-78434;EPI_ISL_15293002:78249-78707;EPI_ISL_15641561:77965-78423;EPI_ISL_15831211:78014-78472;EPI_ISL_15992098:78029-78487;EPI_ISL_16233784:78025-78483;EPI_ISL_16946400:78014-78472;EPI_ISL_17048206:77664-78122;EPI_ISL_17703567:78029-78487;EPI_ISL_17718458:78007-78465;EPI_ISL_18213374:78004-78462;EPI_ISL_18360394:77665-78123;EPI_ISL_18557816:78005-78463;EPI_ISL_18642365:78007-78465;EPI_ISL_18659846:77977-78435;EPI_ISL_18744049:78009-78467;EPI_ISL_19022858:78004-78462;EPI_ISL_19028778:77921-78379;EPI_ISL_19151800:78005-78463;EPI_ISL_19159116:78007-78465;EPI_ISL_19350788:79140-79598;EPI_ISL_19359677:78004-78462;EPI_ISL_19446306:77999-78457;EPI_ISL_19467692:80551-81009;JX878411.1:81259-81717;JX878425.1:80776-81234;JX878426.1:80225-80683;KC257460.1:80193-80651;KJ642612.1:78511-78969;KJ642613.1:80070-80528;KJ642616.1:79647-80105;KJ642618.1:77741-78199;KP849469.1:80584-81042;KP849471.1:80510-80968;LC831698.1:78005-78463;MN648051.1:78227-78685;MN702445.1:73927-74385;MN702446.1:80072-80530;MN702447.1:73440-73898;MN702449.1:73938-74396;MN702452.1:80113-80571;MT903338.1:78170-78628;MT903340.1:78007-78465;MT903341.1:69540-69998;MT903342.1:78022-78480;MT903347.1:79964-80422;ON568298.1:78153-78611;ON622713.1:78239-78697;ON622722.2:78010-78468;ON631963.1:78030-78488;ON649879.1:77840-78298;ON676707.1:78060-78518;ON755039.1:77670-78128;ON808413.1:78010-78468;ON880420.1:78237-78695;ON911481.2:78027-78485;ON918656.1:78042-78500;ON959133.1:78131-78589;ON959136.1:78132-78590;ON983168.1:76706-77164;OP019276.1:78004-78462;OP150924.1:78145-78603;OP160532.1:77970-78428;OP235282.1:78004-78462;OP289782.1:78007-78465;OP331335.1:78024-78482;OP382479.1:78011-78469;OP422341.1:78024-78482;OP434519.1:78003-78461;OP535324.1:78005-78463;OP535333.1:78114-78572;OP536770.1:78006-78464;OP597769.1:78028-78486;OP604566.1:78007-78465;OP612682.1:77932-78390;OP612684.1:77996-78454;OP612686.1:77996-78454;OP696841.1:75281-75739;OP879722.1:77230-77688;OQ419007.1:77240-77698;OQ419012.1:77207-77665;OQ419031.1:77211-77669;OQ419047.1:77213-77671;OQ451898.1:77092-77550;OQ451900.1:78005-78463;OQ581853.1:78031-78489;OQ581855.1:78112-78570;OQ721951.1:78045-78503;OQ721954.1:78002-78460;OR035652.1:77954-78412;OR035664.1:77954-78412;OR035702.1:77954-78412;OR146272.1:77970-78428;OR146330.1:78015-78473;OR264364.1:78025-78483;OR427332.1:78025-78483;OR459777.1:78009-78467;OR459797.1:78024-78482;OR943698.1:80317-80775;PP098589.2:78006-78464;PP496048.1:78010-78468;PP601206.1:80266-80724;PP776553.1:77931-78389;PP852971.1:78004-78462;PP852983.1:78010-78468;PP853000.1:77971-78429;PP853005.1:77979-78437;PP853007.1:78012-78470;PP853013.1:77985-78443;PP853027.1:78016-78474;PP853045.1:77997-78455;PP853048.1:78016-78474;PP860026.1:78014-78472;PP860027.1:78039-78497;PP860028.1:77979-78437;PP860031.1:78033-78491;PP860033.1:78038-78496;PQ153226.1:78004-78462;PQ178862.2:79117-79575;PQ221506.1:74093-74551;PQ221833.1:80259-80717;PQ340477.1:77987-78445;PQ340478.1:77972-78430;PQ340480.1:78051-78509;PQ340483.1:78035-78493;PQ340485.1:78021-78479;PQ340486.1:78036-78494;PQ340487.1:78061-78519;PQ340489.1:78036-78494;PQ340490.1:77987-78445;PQ340491.1:78042-78500;PQ340493.1:78021-78479;PQ340495.1:78051-78509;PQ340496.1:78084-78542;PQ368489.1:78007-78465;PQ451835.1:78092-78550;PQ451836.1:78024-78482;PQ451838.1:78075-78533;PQ451841.1:78035-78493;PQ451842.1:77987-78445 |
| OG0000022 | AY603973.1:81030-82031;AY741551.1:81034-82035;AY753185.1:81273-82274;DQ011154.1:81491-82492;DQ011155.1:81472-82473;EPI_ISL_13331598:79100-80101;EPI_ISL_13363142:79093-80094;EPI_ISL_13374487:79108-80109;EPI_ISL_13502582:79093-80094;EPI_ISL_13508471:79185-80186;EPI_ISL_13607904:79084-80085;EPI_ISL_13624509:79028-80029;EPI_ISL_13889442:79203-80204;EPI_ISL_13953610:79020-80021;EPI_ISL_14181948:79229-80230;EPI_ISL_14838587:81345-82346;EPI_ISL_15263355:79084-80085;EPI_ISL_15269699:79053-80054;EPI_ISL_15293002:79326-80327;EPI_ISL_15641561:79042-80043;EPI_ISL_15831211:79091-80092;EPI_ISL_15992098:79106-80107;EPI_ISL_16233784:79102-80103;EPI_ISL_16946400:79091-80092;EPI_ISL_17048206:78741-79742;EPI_ISL_17703567:79106-80107;EPI_ISL_17718458:79084-80085;EPI_ISL_18213374:79081-80082;EPI_ISL_18360394:78742-79743;EPI_ISL_18557816:79082-80083;EPI_ISL_18642365:79084-80085;EPI_ISL_18659846:79054-80055;EPI_ISL_18744049:79086-80087;EPI_ISL_19022858:79081-80082;EPI_ISL_19028778:78998-79999;EPI_ISL_19151800:79082-80083;EPI_ISL_19159116:79084-80085;EPI_ISL_19350788:80217-81218;EPI_ISL_19359677:79081-80082;EPI_ISL_19446306:79076-80077;EPI_ISL_19467692:81628-82629;JX878411.1:82336-83337;JX878425.1:81853-82854;JX878426.1:81302-82303;KC257460.1:81270-82271;KJ642612.1:79588-80589;KJ642613.1:81147-82148;KJ642616.1:80724-81725;KJ642618.1:78818-79819;KP849469.1:81661-82662;KP849471.1:81587-82588;LC831698.1:79082-80083;MN648051.1:79304-80305;MN702445.1:75004-76005;MN702446.1:81149-82150;MN702447.1:74517-75518;MN702449.1:75015-76016;MN702452.1:81190-82191;MT903338.1:79247-80248;MT903340.1:79084-80085;MT903341.1:70617-71618;MT903342.1:79099-80100;MT903347.1:81041-82042;ON568298.1:79230-80231;ON622713.1:79316-80317;ON622722.2:79087-80088;ON631963.1:79107-80108;ON649879.1:78917-79918;ON676707.1:79137-80138;ON755039.1:78747-79748;ON808413.1:79087-80088;ON880420.1:79314-80315;ON911481.2:79104-80105;ON918656.1:79119-80120;ON959133.1:79208-80209;ON959136.1:79209-80210;ON983168.1:77783-78784;OP019276.1:79081-80082;OP150924.1:79222-80223;OP160532.1:79047-80048;OP235282.1:79081-80082;OP289782.1:79084-80085;OP331335.1:79101-80102;OP382479.1:79088-80089;OP422341.1:79101-80102;OP434519.1:79080-80081;OP535324.1:79082-80083;OP535333.1:79191-80192;OP536770.1:79083-80084;OP597769.1:79105-80106;OP604566.1:79084-80085;OP612682.1:79009-80010;OP612684.1:79073-80074;OP612686.1:79073-80074;OP696841.1:76358-77359;OP879722.1:78307-79308;OQ419007.1:78317-79318;OQ419012.1:78284-79285;OQ419031.1:78288-79289;OQ419047.1:78290-79291;OQ451898.1:78169-79170;OQ451900.1:79082-80083;OQ581853.1:79108-80109;OQ581855.1:79189-80190;OQ721951.1:79122-80123;OQ721954.1:79079-80080;OR035652.1:79031-80032;OR035664.1:79031-80032;OR035702.1:79031-80032;OR146272.1:79047-80048;OR146330.1:79092-80093;OR264364.1:79102-80103;OR427332.1:79102-80103;OR459777.1:79086-80087;OR459797.1:79101-80102;OR943698.1:81394-82395;PP098589.2:79083-80084;PP496048.1:79087-80088;PP601206.1:81343-82344;PP776553.1:79008-80009;PP852971.1:79081-80082;PP852983.1:79087-80088;PP853000.1:79048-80049;PP853005.1:79056-80057;PP853007.1:79089-80090;PP853013.1:79062-80063;PP853027.1:79093-80094;PP853045.1:79074-80075;PP853048.1:79093-80094;PP860026.1:79091-80092;PP860027.1:79116-80117;PP860028.1:79056-80057;PP860031.1:79110-80111;PP860033.1:79115-80116;PQ153226.1:79081-80082;PQ178862.2:80194-81195;PQ221506.1:75170-76171;PQ221833.1:81336-82337;PQ340477.1:79064-80065;PQ340478.1:79049-80050;PQ340480.1:79128-80129;PQ340483.1:79112-80113;PQ340485.1:79098-80099;PQ340486.1:79113-80114;PQ340487.1:79138-80139;PQ340489.1:79113-80114;PQ340490.1:79064-80065;PQ340491.1:79119-80120;PQ340493.1:79098-80099;PQ340495.1:79128-80129;PQ340496.1:79161-80162;PQ368489.1:79084-80085;PQ451835.1:79169-80170;PQ451836.1:79101-80102;PQ451838.1:79152-80153;PQ451841.1:79112-80113;PQ451842.1:79064-80065 |
| OG0000023 | AY603973.1:83073-86933;AY741551.1:83077-86937;AY753185.1:83316-87176;DQ011154.1:83532-87392;DQ011155.1:83513-87373;EPI_ISL_13331598:81141-85001;EPI_ISL_13363142:81134-84994;EPI_ISL_13374487:81149-85009;EPI_ISL_13502582:81134-84994;EPI_ISL_13508471:81226-85086;EPI_ISL_13607904:81125-84985;EPI_ISL_13624509:81069-84929;EPI_ISL_13889442:81244-85104;EPI_ISL_13953610:81061-84921;EPI_ISL_14181948:81270-85130;EPI_ISL_14838587:83386-87246;EPI_ISL_15263355:81125-84985;EPI_ISL_15269699:81094-84954;EPI_ISL_15293002:81367-85227;EPI_ISL_15641561:81083-84943;EPI_ISL_15831211:81132-84992;EPI_ISL_15992098:81147-85007;EPI_ISL_16233784:81143-85003;EPI_ISL_16946400:81132-84992;EPI_ISL_17048206:80782-84642;EPI_ISL_17703567:81147-85007;EPI_ISL_17718458:81125-84985;EPI_ISL_18213374:81122-84982;EPI_ISL_18360394:80783-84643;EPI_ISL_18557816:81123-84983;EPI_ISL_18642365:81125-84985;EPI_ISL_18659846:81095-84955;EPI_ISL_18744049:81127-84987;EPI_ISL_19022858:81122-84982;EPI_ISL_19028778:81039-84899;EPI_ISL_19151800:81123-84983;EPI_ISL_19159116:81125-84985;EPI_ISL_19350788:82258-86118;EPI_ISL_19359677:81122-84982;EPI_ISL_19446306:81117-84977;EPI_ISL_19467692:83669-87529;JX878411.1:84377-88237;JX878425.1:83894-87754;JX878426.1:83343-87203;KC257460.1:83311-87171;KJ642612.1:81629-85489;KJ642613.1:83188-87048;KJ642616.1:82767-86627;KJ642618.1:80859-84719;KP849469.1:83702-87562;KP849471.1:83628-87488;LC831698.1:81123-84983;MN648051.1:81345-85205;MN702445.1:77045-80905;MN702446.1:83190-87050;MN702447.1:76558-80418;MN702449.1:77056-80916;MN702452.1:83231-87091;MT903338.1:81288-85148;MT903340.1:81125-84985;MT903341.1:72658-76518;MT903342.1:81140-85000;MT903347.1:83084-86944;ON568298.1:81271-85131;ON622713.1:81357-85217;ON622722.2:81128-84988;ON631963.1:81148-85008;ON649879.1:80958-84818;ON676707.1:81178-85038;ON755039.1:80788-84648;ON808413.1:81128-84988;ON880420.1:81355-85215;ON911481.2:81145-85005;ON918656.1:81160-85020;ON959133.1:81249-85109;ON959136.1:81250-85110;ON983168.1:79823-83683;OP019276.1:81122-84982;OP150924.1:81263-85123;OP160532.1:81088-84948;OP235282.1:81122-84982;OP289782.1:81125-84985;OP331335.1:81142-85002;OP382479.1:81129-84989;OP422341.1:81142-85002;OP434519.1:81121-84981;OP535324.1:81123-84983;OP535333.1:81232-85092;OP536770.1:81124-84984;OP597769.1:81146-85006;OP604566.1:81125-84985;OP612682.1:81050-84910;OP612684.1:81114-84974;OP612686.1:81114-84974;OP696841.1:78399-82259;OP879722.1:80348-84208;OQ419007.1:80358-84218;OQ419012.1:80325-84185;OQ419031.1:80329-84189;OQ419047.1:80331-84191;OQ451898.1:80210-84070;OQ451900.1:81123-84983;OQ581853.1:81149-85009;OQ581855.1:81230-85090;OQ721951.1:81163-85023;OQ721954.1:81120-84980;OR035652.1:81072-84932;OR035664.1:81072-84932;OR035702.1:81072-84932;OR146272.1:81088-84948;OR146330.1:81133-84993;OR264364.1:81143-85003;OR427332.1:81143-85003;OR459777.1:81127-84987;OR459797.1:81142-85002;OR943698.1:83435-87295;PP098589.2:81124-84984;PP496048.1:81128-84988;PP601206.1:83384-87244;PP776553.1:81049-84909;PP852971.1:81122-84982;PP852983.1:81128-84988;PP853000.1:81089-84949;PP853005.1:81097-84957;PP853007.1:81130-84990;PP853013.1:81103-84963;PP853027.1:81134-84994;PP853045.1:81115-84975;PP853048.1:81134-84994;PP860026.1:81132-84992;PP860027.1:81157-85017;PP860028.1:81097-84957;PP860031.1:81151-85011;PP860033.1:81156-85016;PQ153226.1:81122-84982;PQ178862.2:82235-86095;PQ221506.1:77211-81071;PQ221833.1:83377-87237;PQ340477.1:81105-84965;PQ340478.1:81090-84950;PQ340480.1:81169-85029;PQ340483.1:81153-85013;PQ340485.1:81139-84999;PQ340486.1:81154-85014;PQ340487.1:81179-85039;PQ340489.1:81154-85014;PQ340490.1:81105-84965;PQ340491.1:81160-85020;PQ340493.1:81139-84999;PQ340495.1:81169-85029;PQ340496.1:81202-85062;PQ368489.1:81125-84985;PQ451835.1:81210-85070;PQ451836.1:81142-85002;PQ451838.1:81193-85053;PQ451841.1:81153-85013;PQ451842.1:81105-84965 |
| OG0000024 | AY603973.1:87459-88028;AY741551.1:87463-88032;AY753185.1:87702-88271;DQ011154.1:87918-88487;DQ011155.1:87899-88468;EPI_ISL_13331598:85527-86096;EPI_ISL_13363142:85520-86089;EPI_ISL_13374487:85535-86104;EPI_ISL_13502582:85520-86089;EPI_ISL_13508471:85612-86181;EPI_ISL_13607904:85511-86080;EPI_ISL_13624509:85455-86024;EPI_ISL_13889442:85630-86199;EPI_ISL_13953610:85447-86016;EPI_ISL_14181948:85656-86225;EPI_ISL_14838587:87772-88341;EPI_ISL_15263355:85511-86080;EPI_ISL_15269699:85480-86049;EPI_ISL_15293002:85753-86322;EPI_ISL_15641561:85469-86038;EPI_ISL_15831211:85518-86087;EPI_ISL_15992098:85533-86102;EPI_ISL_16233784:85529-86098;EPI_ISL_16946400:85518-86087;EPI_ISL_17048206:85168-85737;EPI_ISL_17703567:85533-86102;EPI_ISL_17718458:85511-86080;EPI_ISL_18213374:85508-86077;EPI_ISL_18360394:85169-85738;EPI_ISL_18557816:85509-86078;EPI_ISL_18642365:85511-86080;EPI_ISL_18659846:85481-86050;EPI_ISL_18744049:85513-86082;EPI_ISL_19022858:85508-86077;EPI_ISL_19028778:85425-85994;EPI_ISL_19151800:85509-86078;EPI_ISL_19159116:85511-86080;EPI_ISL_19350788:86644-87213;EPI_ISL_19359677:85508-86077;EPI_ISL_19446306:85503-86072;EPI_ISL_19467692:88055-88624;JX878411.1:88763-89332;JX878425.1:88280-88849;JX878426.1:87729-88298;KC257460.1:87697-88266;KJ642612.1:86015-86584;KJ642613.1:87574-88143;KJ642616.1:87153-87722;KJ642618.1:85245-85814;KP849469.1:88088-88657;KP849471.1:88014-88583;LC831698.1:85509-86078;MN648051.1:85731-86300;MN702445.1:81431-82000;MN702446.1:87576-88145;MN702447.1:80944-81513;MN702449.1:81442-82011;MN702452.1:87617-88186;MT903338.1:85674-86243;MT903340.1:85511-86080;MT903341.1:77044-77613;MT903342.1:85526-86095;MT903347.1:87470-88039;ON568298.1:85657-86226;ON622713.1:85743-86312;ON622722.2:85514-86083;ON631963.1:85534-86103;ON649879.1:85344-85913;ON676707.1:85564-86133;ON755039.1:85174-85743;ON808413.1:85514-86083;ON880420.1:85741-86310;ON911481.2:85531-86100;ON918656.1:85546-86115;ON959133.1:85635-86204;ON959136.1:85636-86205;ON983168.1:84209-84778;OP019276.1:85508-86077;OP150924.1:85649-86218;OP160532.1:85474-86043;OP235282.1:85508-86077;OP289782.1:85511-86080;OP331335.1:85528-86097;OP382479.1:85515-86084;OP422341.1:85528-86097;OP434519.1:85507-86076;OP535324.1:85509-86078;OP535333.1:85618-86187;OP536770.1:85510-86079;OP597769.1:85532-86101;OP604566.1:85511-86080;OP612682.1:85436-86005;OP612684.1:85500-86069;OP612686.1:85500-86069;OP696841.1:82785-83354;OP879722.1:84734-85303;OQ419007.1:84744-85313;OQ419012.1:84711-85280;OQ419031.1:84715-85284;OQ419047.1:84717-85286;OQ451898.1:84596-85165;OQ451900.1:85509-86078;OQ581853.1:85535-86104;OQ581855.1:85616-86185;OQ721951.1:85549-86118;OQ721954.1:85506-86075;OR035652.1:85458-86027;OR035664.1:85458-86027;OR035702.1:85458-86027;OR146272.1:85474-86043;OR146330.1:85519-86088;OR264364.1:85529-86098;OR427332.1:85529-86098;OR459777.1:85513-86082;OR459797.1:85528-86097;OR943698.1:87821-88390;PP098589.2:85510-86079;PP496048.1:85514-86083;PP601206.1:87770-88339;PP776553.1:85435-86004;PP852971.1:85508-86077;PP852983.1:85514-86083;PP853000.1:85475-86044;PP853005.1:85483-86052;PP853007.1:85516-86085;PP853013.1:85489-86058;PP853027.1:85520-86089;PP853045.1:85501-86070;PP853048.1:85520-86089;PP860026.1:85518-86087;PP860027.1:85543-86112;PP860028.1:85483-86052;PP860031.1:85537-86106;PP860033.1:85542-86111;PQ153226.1:85508-86077;PQ178862.2:86621-87190;PQ221506.1:81597-82166;PQ221833.1:87763-88332;PQ340477.1:85491-86060;PQ340478.1:85476-86045;PQ340480.1:85555-86124;PQ340483.1:85539-86108;PQ340485.1:85525-86094;PQ340486.1:85540-86109;PQ340487.1:85565-86134;PQ340489.1:85540-86109;PQ340490.1:85491-86060;PQ340491.1:85546-86115;PQ340493.1:85525-86094;PQ340495.1:85555-86124;PQ340496.1:85588-86157;PQ368489.1:85511-86080;PQ451835.1:85596-86165;PQ451836.1:85528-86097;PQ451838.1:85579-86148;PQ451841.1:85539-86108;PQ451842.1:85491-86060 |
| OG0000025 | AY603973.1:93672-96209;AY741551.1:93676-96213;AY753185.1:93915-96452;DQ011154.1:94146-96683;DQ011155.1:94127-96664;EPI_ISL_13331598:91746-94283;EPI_ISL_13363142:91739-94276;EPI_ISL_13374487:91754-94291;EPI_ISL_13502582:91739-94276;EPI_ISL_13508471:91831-94368;EPI_ISL_13607904:91730-94267;EPI_ISL_13624509:91674-94211;EPI_ISL_13889442:91849-94386;EPI_ISL_13953610:91647-94184;EPI_ISL_14181948:91875-94412;EPI_ISL_14838587:94000-96537;EPI_ISL_15263355:91730-94267;EPI_ISL_15269699:91699-94236;EPI_ISL_15293002:91972-94509;EPI_ISL_15641561:91688-94225;EPI_ISL_15831211:91737-94274;EPI_ISL_15992098:91752-94289;EPI_ISL_16233784:91748-94285;EPI_ISL_16946400:91737-94274;EPI_ISL_17048206:91387-93924;EPI_ISL_17703567:91752-94289;EPI_ISL_17718458:91730-94267;EPI_ISL_18213374:91727-94264;EPI_ISL_18360394:91388-93925;EPI_ISL_18557816:91728-94265;EPI_ISL_18642365:91730-94267;EPI_ISL_18659846:91700-94237;EPI_ISL_18744049:91732-94269;EPI_ISL_19022858:91727-94264;EPI_ISL_19028778:91644-94181;EPI_ISL_19151800:91728-94265;EPI_ISL_19159116:91730-94267;EPI_ISL_19350788:92872-95409;EPI_ISL_19359677:91727-94264;EPI_ISL_19446306:91722-94259;EPI_ISL_19467692:94283-96820;JX878411.1:94991-97528;JX878425.1:94508-97045;JX878426.1:93957-96494;KC257460.1:93925-96462;KJ642612.1:92243-94780;KJ642613.1:93802-96339;KJ642616.1:93366-95903;KJ642618.1:91473-94010;KP849469.1:94316-96853;KP849471.1:94242-96779;LC831698.1:91728-94265;MN648051.1:91950-94487;MN702445.1:87659-90196;MN702446.1:93804-96341;MN702447.1:87172-89709;MN702449.1:87670-90207;MN702452.1:93845-96382;MT903338.1:91893-94430;MT903340.1:91730-94267;MT903341.1:83263-85800;MT903342.1:91745-94282;MT903347.1:93683-96220;ON568298.1:91876-94413;ON622713.1:91962-94499;ON622722.2:91733-94270;ON631963.1:91753-94290;ON649879.1:91563-94100;ON676707.1:91783-94320;ON755039.1:91393-93930;ON808413.1:91733-94270;ON880420.1:91960-94497;ON911481.2:91750-94287;ON918656.1:91765-94302;ON959133.1:91854-94391;ON959136.1:91855-94392;ON983168.1:90428-92965;OP019276.1:91727-94264;OP150924.1:91868-94405;OP160532.1:91693-94230;OP235282.1:91727-94264;OP289782.1:91730-94267;OP331335.1:91747-94284;OP382479.1:91734-94271;OP422341.1:91747-94284;OP434519.1:91726-94263;OP535324.1:91728-94265;OP535333.1:91837-94374;OP536770.1:91729-94266;OP597769.1:91751-94288;OP604566.1:91730-94267;OP612682.1:91655-94192;OP612684.1:91718-94255;OP612686.1:91719-94256;OP696841.1:89004-91541;OP879722.1:90953-93490;OQ419007.1:90963-93500;OQ419012.1:90930-93467;OQ419031.1:90934-93471;OQ419047.1:90936-93473;OQ451898.1:90815-93352;OQ451900.1:91728-94265;OQ581853.1:91754-94291;OQ581855.1:91835-94372;OQ721951.1:91768-94305;OQ721954.1:91725-94262;OR035652.1:91677-94214;OR035664.1:91677-94214;OR035702.1:91677-94214;OR146272.1:91693-94230;OR146330.1:91738-94275;OR264364.1:91748-94285;OR427332.1:91748-94285;OR459777.1:91732-94269;OR459797.1:91747-94284;OR943698.1:94058-96595;PP098589.2:91729-94266;PP496048.1:91733-94270;PP601206.1:93989-96526;PP776553.1:91654-94191;PP852971.1:91727-94264;PP852983.1:91733-94270;PP853000.1:91694-94231;PP853005.1:91702-94239;PP853007.1:91735-94272;PP853013.1:91708-94245;PP853027.1:91739-94276;PP853045.1:91720-94257;PP853048.1:91739-94276;PP860026.1:91737-94274;PP860027.1:91762-94299;PP860028.1:91702-94239;PP860031.1:91756-94293;PP860033.1:91761-94298;PQ153226.1:91727-94264;PQ178862.2:92849-95386;PQ221506.1:87825-90362;PQ221833.1:93991-96528;PQ340477.1:91710-94247;PQ340478.1:91695-94232;PQ340480.1:91774-94311;PQ340483.1:91758-94295;PQ340485.1:91744-94281;PQ340486.1:91759-94296;PQ340487.1:91784-94321;PQ340489.1:91759-94296;PQ340490.1:91710-94247;PQ340491.1:91765-94302;PQ340493.1:91744-94281;PQ340495.1:91774-94311;PQ340496.1:91807-94344;PQ368489.1:91730-94267;PQ451835.1:91815-94352;PQ451836.1:91747-94284;PQ451838.1:91798-94335;PQ451841.1:91758-94295;PQ451842.1:91710-94247 |
| OG0000026 | AY603973.1:97302-97958;AY741551.1:97306-97962;AY753185.1:97545-98201;DQ011154.1:97776-98432;DQ011155.1:97757-98413;EPI_ISL_13331598:95376-96032;EPI_ISL_13363142:95369-96025;EPI_ISL_13374487:95384-96040;EPI_ISL_13502582:95369-96025;EPI_ISL_13508471:95461-96117;EPI_ISL_13607904:95360-96016;EPI_ISL_13624509:95304-95960;EPI_ISL_13889442:95479-96135;EPI_ISL_13953610:95277-95933;EPI_ISL_14181948:95505-96161;EPI_ISL_14838587:97630-98286;EPI_ISL_15263355:95360-96016;EPI_ISL_15269699:95329-95985;EPI_ISL_15293002:95602-96258;EPI_ISL_15641561:95318-95974;EPI_ISL_15831211:95367-96023;EPI_ISL_15992098:95382-96038;EPI_ISL_16233784:95378-96034;EPI_ISL_16946400:95367-96023;EPI_ISL_17048206:95017-95673;EPI_ISL_17703567:95382-96038;EPI_ISL_17718458:95360-96016;EPI_ISL_18213374:95357-96013;EPI_ISL_18360394:95018-95674;EPI_ISL_18557816:95358-96014;EPI_ISL_18642365:95360-96016;EPI_ISL_18659846:95330-95986;EPI_ISL_18744049:95362-96018;EPI_ISL_19022858:95357-96013;EPI_ISL_19028778:95274-95930;EPI_ISL_19151800:95358-96014;EPI_ISL_19159116:95360-96016;EPI_ISL_19350788:96502-97158;EPI_ISL_19359677:95357-96013;EPI_ISL_19446306:95352-96008;EPI_ISL_19467692:97913-98569;JX878411.1:98621-99277;JX878425.1:98138-98794;JX878426.1:97587-98243;KC257460.1:97555-98211;KJ642612.1:95873-96529;KJ642613.1:97432-98088;KJ642616.1:96996-97652;KJ642618.1:95103-95759;KP849469.1:97946-98602;KP849471.1:97872-98528;LC831698.1:95358-96014;MN648051.1:95580-96236;MN702445.1:91289-91945;MN702446.1:97434-98090;MN702447.1:90802-91458;MN702449.1:91300-91956;MN702452.1:97475-98131;MT903338.1:95523-96179;MT903340.1:95360-96016;MT903341.1:86893-87549;MT903342.1:95375-96031;MT903347.1:97313-97969;ON568298.1:95506-96162;ON622713.1:95592-96248;ON622722.2:95363-96019;ON631963.1:95383-96039;ON649879.1:95193-95849;ON676707.1:95413-96069;ON755039.1:95023-95679;ON808413.1:95363-96019;ON880420.1:95590-96246;ON911481.2:95380-96036;ON918656.1:95395-96051;ON959133.1:95484-96140;ON959136.1:95485-96141;ON983168.1:94058-94714;OP019276.1:95357-96013;OP150924.1:95498-96154;OP160532.1:95323-95979;OP235282.1:95357-96013;OP289782.1:95360-96016;OP331335.1:95377-96033;OP382479.1:95364-96020;OP422341.1:95377-96033;OP434519.1:95356-96012;OP535324.1:95358-96014;OP535333.1:95467-96123;OP536770.1:95359-96015;OP597769.1:95381-96037;OP604566.1:95360-96016;OP612682.1:95285-95941;OP612684.1:95348-96004;OP612686.1:95349-96005;OP696841.1:92634-93290;OP879722.1:94583-95239;OQ419007.1:94593-95249;OQ419012.1:94560-95216;OQ419031.1:94564-95220;OQ419047.1:94566-95222;OQ451898.1:94445-95101;OQ451900.1:95358-96014;OQ581853.1:95384-96040;OQ581855.1:95465-96121;OQ721951.1:95398-96054;OQ721954.1:95355-96011;OR035652.1:95307-95963;OR035664.1:95307-95963;OR035702.1:95307-95963;OR146272.1:95323-95979;OR146330.1:95368-96024;OR264364.1:95378-96034;OR427332.1:95378-96034;OR459777.1:95362-96018;OR459797.1:95377-96033;OR943698.1:97688-98344;PP098589.2:95359-96015;PP496048.1:95363-96019;PP601206.1:97619-98275;PP776553.1:95284-95940;PP852971.1:95357-96013;PP852983.1:95363-96019;PP853000.1:95324-95980;PP853005.1:95332-95988;PP853007.1:95365-96021;PP853013.1:95338-95994;PP853027.1:95369-96025;PP853045.1:95350-96006;PP853048.1:95369-96025;PP860026.1:95367-96023;PP860027.1:95392-96048;PP860028.1:95332-95988;PP860031.1:95386-96042;PP860033.1:95391-96047;PQ153226.1:95357-96013;PQ178862.2:96479-97135;PQ221506.1:91455-92111;PQ221833.1:97621-98277;PQ340477.1:95340-95996;PQ340478.1:95325-95981;PQ340480.1:95404-96060;PQ340483.1:95388-96044;PQ340485.1:95374-96030;PQ340486.1:95389-96045;PQ340487.1:95414-96070;PQ340489.1:95389-96045;PQ340490.1:95340-95996;PQ340491.1:95395-96051;PQ340493.1:95374-96030;PQ340495.1:95404-96060;PQ340496.1:95437-96093;PQ368489.1:95360-96016;PQ451835.1:95445-96101;PQ451836.1:95377-96033;PQ451838.1:95428-96084;PQ451841.1:95388-96044;PQ451842.1:95340-95996 |
| OG0000027 | AY603973.1:102327-102812;AY741551.1:102331-102816;AY753185.1:102570-103055;DQ011154.1:102801-103286;DQ011155.1:102782-103267;EPI_ISL_13331598:100401-100886;EPI_ISL_13363142:100394-100879;EPI_ISL_13374487:100409-100894;EPI_ISL_13502582:100394-100879;EPI_ISL_13508471:100486-100971;EPI_ISL_13607904:100385-100870;EPI_ISL_13624509:100329-100814;EPI_ISL_13889442:100504-100989;EPI_ISL_13953610:100302-100787;EPI_ISL_14181948:100530-101015;EPI_ISL_14838587:102655-103140;EPI_ISL_15263355:100385-100870;EPI_ISL_15269699:100354-100839;EPI_ISL_15293002:100627-101112;EPI_ISL_15641561:100343-100828;EPI_ISL_15831211:100392-100877;EPI_ISL_15992098:100407-100892;EPI_ISL_16233784:100403-100888;EPI_ISL_16946400:100392-100877;EPI_ISL_17048206:100042-100527;EPI_ISL_17703567:100407-100892;EPI_ISL_17718458:100385-100870;EPI_ISL_18213374:100382-100867;EPI_ISL_18360394:100043-100528;EPI_ISL_18557816:100383-100868;EPI_ISL_18642365:100385-100870;EPI_ISL_18659846:100355-100840;EPI_ISL_18744049:100387-100872;EPI_ISL_19022858:100382-100867;EPI_ISL_19028778:100299-100784;EPI_ISL_19151800:100383-100868;EPI_ISL_19159116:100385-100870;EPI_ISL_19350788:101527-102012;EPI_ISL_19359677:100382-100867;EPI_ISL_19446306:100377-100862;EPI_ISL_19467692:102938-103423;JX878411.1:103646-104131;JX878425.1:103163-103648;JX878426.1:102612-103097;KC257460.1:102580-103065;KJ642612.1:100898-101383;KJ642613.1:102457-102942;KJ642616.1:102021-102506;KJ642618.1:100128-100613;KP849469.1:102971-103456;KP849471.1:102897-103382;LC831698.1:100383-100868;MN648051.1:100605-101090;MN702445.1:96314-96799;MN702446.1:102459-102944;MN702447.1:95827-96312;MN702449.1:96325-96810;MN702452.1:102500-102985;MT903338.1:100548-101033;MT903340.1:100385-100870;MT903341.1:91918-92403;MT903342.1:100400-100885;MT903347.1:102338-102823;ON568298.1:100531-101016;ON622713.1:100617-101102;ON622722.2:100388-100873;ON631963.1:100408-100893;ON649879.1:100218-100703;ON676707.1:100438-100923;ON755039.1:100048-100533;ON808413.1:100388-100873;ON880420.1:100615-101100;ON911481.2:100405-100890;ON918656.1:100420-100905;ON959133.1:100509-100994;ON959136.1:100510-100995;ON983168.1:99083-99568;OP019276.1:100382-100867;OP150924.1:100523-101008;OP160532.1:100348-100833;OP235282.1:100382-100867;OP289782.1:100385-100870;OP331335.1:100402-100887;OP382479.1:100389-100874;OP422341.1:100402-100887;OP434519.1:100381-100866;OP535324.1:100383-100868;OP535333.1:100492-100977;OP536770.1:100384-100869;OP597769.1:100406-100891;OP604566.1:100385-100870;OP612682.1:100310-100795;OP612684.1:100373-100858;OP612686.1:100374-100859;OP696841.1:97659-98144;OP879722.1:99608-100093;OQ419007.1:99618-100103;OQ419012.1:99585-100070;OQ419031.1:99589-100074;OQ419047.1:99591-100076;OQ451898.1:99470-99955;OQ451900.1:100383-100868;OQ581853.1:100409-100894;OQ581855.1:100490-100975;OQ721951.1:100423-100908;OQ721954.1:100380-100865;OR035652.1:100332-100817;OR035664.1:100332-100817;OR035702.1:100332-100817;OR146272.1:100348-100833;OR146330.1:100393-100878;OR264364.1:100403-100888;OR427332.1:100403-100888;OR459777.1:100387-100872;OR459797.1:100402-100887;OR943698.1:102713-103198;PP098589.2:100384-100869;PP496048.1:100388-100873;PP601206.1:102644-103129;PP776553.1:100309-100794;PP852971.1:100382-100867;PP852983.1:100388-100873;PP853000.1:100349-100834;PP853005.1:100357-100842;PP853007.1:100390-100875;PP853013.1:100363-100848;PP853027.1:100394-100879;PP853045.1:100375-100860;PP853048.1:100394-100879;PP860026.1:100392-100877;PP860027.1:100417-100902;PP860028.1:100357-100842;PP860031.1:100411-100896;PP860033.1:100416-100901;PQ153226.1:100382-100867;PQ178862.2:101504-101989;PQ221506.1:96480-96965;PQ221833.1:102646-103131;PQ340477.1:100365-100850;PQ340478.1:100350-100835;PQ340480.1:100429-100914;PQ340483.1:100413-100898;PQ340485.1:100399-100884;PQ340486.1:100414-100899;PQ340487.1:100439-100924;PQ340489.1:100414-100899;PQ340490.1:100365-100850;PQ340491.1:100420-100905;PQ340493.1:100399-100884;PQ340495.1:100429-100914;PQ340496.1:100462-100947;PQ368489.1:100385-100870;PQ451835.1:100470-100955;PQ451836.1:100402-100887;PQ451838.1:100453-100938;PQ451841.1:100413-100898;PQ451842.1:100365-100850 |
| OG0000028 | AY603973.1:103731-104372;AY741551.1:103735-104376;AY753185.1:103974-104615;DQ011154.1:104205-104846;DQ011155.1:104186-104827;EPI_ISL_13331598:101805-102446;EPI_ISL_13363142:101798-102439;EPI_ISL_13374487:101813-102454;EPI_ISL_13502582:101798-102439;EPI_ISL_13508471:101890-102531;EPI_ISL_13607904:101789-102430;EPI_ISL_13624509:101733-102374;EPI_ISL_13889442:101908-102549;EPI_ISL_13953610:101706-102347;EPI_ISL_14181948:101934-102575;EPI_ISL_14838587:104059-104700;EPI_ISL_15263355:101789-102430;EPI_ISL_15269699:101758-102399;EPI_ISL_15293002:102031-102672;EPI_ISL_15641561:101747-102388;EPI_ISL_15831211:101796-102437;EPI_ISL_15992098:101811-102452;EPI_ISL_16233784:101807-102448;EPI_ISL_16946400:101796-102437;EPI_ISL_17048206:101446-102087;EPI_ISL_17703567:101811-102452;EPI_ISL_17718458:101789-102430;EPI_ISL_18213374:101786-102427;EPI_ISL_18360394:101447-102088;EPI_ISL_18557816:101787-102428;EPI_ISL_18642365:101789-102430;EPI_ISL_18659846:101759-102400;EPI_ISL_18744049:101791-102432;EPI_ISL_19022858:101786-102427;EPI_ISL_19028778:101703-102344;EPI_ISL_19151800:101787-102428;EPI_ISL_19159116:101789-102430;EPI_ISL_19350788:102931-103572;EPI_ISL_19359677:101786-102427;EPI_ISL_19446306:101781-102422;EPI_ISL_19467692:104342-104983;JX878411.1:105050-105691;JX878425.1:104567-105208;JX878426.1:104016-104657;KC257460.1:103984-104625;KJ642612.1:102302-102943;KJ642613.1:103861-104502;KJ642616.1:103425-104066;KJ642618.1:101532-102173;KP849469.1:104375-105016;KP849471.1:104301-104942;LC831698.1:101787-102428;MN648051.1:102009-102650;MN702445.1:97718-98359;MN702446.1:103863-104504;MN702447.1:97231-97872;MN702449.1:97729-98370;MN702452.1:103904-104545;MT903338.1:101952-102593;MT903340.1:101789-102430;MT903341.1:93322-93963;MT903342.1:101804-102445;MT903347.1:103742-104383;ON568298.1:101935-102576;ON622713.1:102021-102662;ON622722.2:101792-102433;ON631963.1:101812-102453;ON649879.1:101622-102263;ON676707.1:101842-102483;ON755039.1:101452-102093;ON808413.1:101792-102433;ON880420.1:102019-102660;ON911481.2:101809-102450;ON918656.1:101824-102465;ON959133.1:101913-102554;ON959136.1:101914-102555;ON983168.1:100487-101128;OP019276.1:101786-102427;OP150924.1:101927-102568;OP160532.1:101752-102393;OP235282.1:101786-102427;OP289782.1:101789-102430;OP331335.1:101806-102447;OP382479.1:101793-102434;OP422341.1:101806-102447;OP434519.1:101785-102426;OP535324.1:101787-102428;OP535333.1:101896-102537;OP536770.1:101788-102429;OP597769.1:101810-102451;OP604566.1:101789-102430;OP612682.1:101714-102355;OP612684.1:101777-102418;OP612686.1:101778-102419;OP696841.1:99063-99704;OP879722.1:101012-101653;OQ419007.1:101022-101663;OQ419012.1:100989-101630;OQ419031.1:100993-101634;OQ419047.1:100995-101636;OQ451898.1:100874-101515;OQ451900.1:101787-102428;OQ581853.1:101813-102454;OQ581855.1:101894-102535;OQ721951.1:101827-102468;OQ721954.1:101784-102425;OR035652.1:101736-102377;OR035664.1:101736-102377;OR035702.1:101736-102377;OR146272.1:101752-102393;OR146330.1:101797-102438;OR264364.1:101807-102448;OR427332.1:101807-102448;OR459777.1:101791-102432;OR459797.1:101806-102447;OR943698.1:104117-104758;PP098589.2:101788-102429;PP496048.1:101792-102433;PP601206.1:104048-104689;PP776553.1:101713-102354;PP852971.1:101786-102427;PP852983.1:101792-102433;PP853000.1:101753-102394;PP853005.1:101761-102402;PP853007.1:101794-102435;PP853013.1:101767-102408;PP853027.1:101798-102439;PP853045.1:101779-102420;PP853048.1:101798-102439;PP860026.1:101796-102437;PP860027.1:101821-102462;PP860028.1:101761-102402;PP860031.1:101815-102456;PP860033.1:101820-102461;PQ153226.1:101786-102427;PQ178862.2:102908-103549;PQ221506.1:97884-98525;PQ221833.1:104050-104691;PQ340477.1:101769-102410;PQ340478.1:101754-102395;PQ340480.1:101833-102474;PQ340483.1:101817-102458;PQ340485.1:101803-102444;PQ340486.1:101818-102459;PQ340487.1:101843-102484;PQ340489.1:101818-102459;PQ340490.1:101769-102410;PQ340491.1:101824-102465;PQ340493.1:101803-102444;PQ340495.1:101833-102474;PQ340496.1:101866-102507;PQ368489.1:101789-102430;PQ451835.1:101874-102515;PQ451836.1:101806-102447;PQ451838.1:101857-102498;PQ451841.1:101817-102458;PQ451842.1:101769-102410 |
| OG0000029 | AY603973.1:107871-108089;AY741551.1:107875-108093;AY753185.1:108114-108332;DQ011154.1:108345-108563;DQ011155.1:108326-108544;EPI_ISL_13331598:105945-106163;EPI_ISL_13363142:105938-106156;EPI_ISL_13374487:105953-106171;EPI_ISL_13502582:105938-106156;EPI_ISL_13508471:106030-106248;EPI_ISL_13607904:105929-106147;EPI_ISL_13624509:105873-106091;EPI_ISL_13889442:106048-106266;EPI_ISL_13953610:105846-106064;EPI_ISL_14181948:106074-106292;EPI_ISL_14838587:108199-108417;EPI_ISL_15263355:105929-106147;EPI_ISL_15269699:105898-106116;EPI_ISL_15293002:106171-106389;EPI_ISL_15641561:105887-106105;EPI_ISL_15831211:105936-106154;EPI_ISL_15992098:105951-106169;EPI_ISL_16233784:105947-106165;EPI_ISL_16946400:105936-106154;EPI_ISL_17048206:105586-105804;EPI_ISL_17703567:105951-106169;EPI_ISL_17718458:105929-106147;EPI_ISL_18213374:105926-106144;EPI_ISL_18360394:105587-105805;EPI_ISL_18557816:105927-106145;EPI_ISL_18642365:105929-106147;EPI_ISL_18659846:105899-106117;EPI_ISL_18744049:105931-106149;EPI_ISL_19022858:105926-106144;EPI_ISL_19028778:105843-106061;EPI_ISL_19151800:105927-106145;EPI_ISL_19159116:105929-106147;EPI_ISL_19350788:107071-107289;EPI_ISL_19359677:105926-106144;EPI_ISL_19446306:105921-106139;EPI_ISL_19467692:108482-108700;JX878411.1:109190-109408;JX878425.1:108707-108925;JX878426.1:108156-108374;KC257460.1:108124-108342;KJ642612.1:106442-106660;KJ642613.1:108001-108219;KJ642616.1:107565-107783;KJ642618.1:105672-105890;KP849469.1:108515-108733;KP849471.1:108441-108659;LC831698.1:105927-106145;MN648051.1:106149-106367;MN702445.1:101858-102076;MN702446.1:108003-108221;MN702447.1:101371-101589;MN702449.1:101869-102087;MN702452.1:108044-108262;MT903338.1:106092-106310;MT903340.1:105929-106147;MT903341.1:97462-97680;MT903342.1:105944-106162;MT903347.1:107882-108100;ON568298.1:106075-106293;ON622713.1:106161-106379;ON622722.2:105932-106150;ON631963.1:105952-106170;ON649879.1:105762-105980;ON676707.1:105982-106200;ON755039.1:105592-105810;ON808413.1:105932-106150;ON880420.1:106159-106377;ON911481.2:105949-106167;ON918656.1:105964-106182;ON959133.1:106053-106271;ON959136.1:106054-106272;ON983168.1:104627-104845;OP019276.1:105926-106144;OP150924.1:106067-106285;OP160532.1:105892-106110;OP235282.1:105926-106144;OP289782.1:105929-106147;OP331335.1:105946-106164;OP382479.1:105933-106151;OP422341.1:105946-106164;OP434519.1:105925-106143;OP535324.1:105927-106145;OP535333.1:106036-106254;OP536770.1:105928-106146;OP597769.1:105950-106168;OP604566.1:105929-106147;OP612682.1:105854-106072;OP612684.1:105917-106135;OP612686.1:105918-106136;OP696841.1:103203-103421;OP879722.1:105152-105370;OQ419007.1:105162-105380;OQ419012.1:105129-105347;OQ419031.1:105133-105351;OQ419047.1:105135-105353;OQ451898.1:105014-105232;OQ451900.1:105927-106145;OQ581853.1:105953-106171;OQ581855.1:106034-106252;OQ721951.1:105967-106185;OQ721954.1:105924-106142;OR035652.1:105876-106094;OR035664.1:105876-106094;OR035702.1:105876-106094;OR146272.1:105892-106110;OR146330.1:105937-106155;OR264364.1:105947-106165;OR427332.1:105947-106165;OR459777.1:105931-106149;OR459797.1:105946-106164;OR943698.1:108257-108475;PP098589.2:105928-106146;PP496048.1:105932-106150;PP601206.1:108188-108406;PP776553.1:105853-106071;PP852971.1:105926-106144;PP852983.1:105932-106150;PP853000.1:105893-106111;PP853005.1:105901-106119;PP853007.1:105934-106152;PP853013.1:105907-106125;PP853027.1:105938-106156;PP853045.1:105919-106137;PP853048.1:105938-106156;PP860026.1:105936-106154;PP860027.1:105961-106179;PP860028.1:105901-106119;PP860031.1:105955-106173;PP860033.1:105960-106178;PQ153226.1:105926-106144;PQ178862.2:107048-107266;PQ221506.1:102024-102242;PQ221833.1:108190-108408;PQ340477.1:105909-106127;PQ340478.1:105894-106112;PQ340480.1:105973-106191;PQ340483.1:105957-106175;PQ340485.1:105943-106161;PQ340486.1:105958-106176;PQ340487.1:105983-106201;PQ340489.1:105958-106176;PQ340490.1:105909-106127;PQ340491.1:105964-106182;PQ340493.1:105943-106161;PQ340495.1:105973-106191;PQ340496.1:106006-106224;PQ368489.1:105929-106147;PQ451835.1:106014-106232;PQ451836.1:105946-106164;PQ451838.1:105997-106215;PQ451841.1:105957-106175;PQ451842.1:105909-106127 |
| OG0000030 | AY603973.1:129738-130886;AY741551.1:129760-130908;AY753185.1:129981-131129;DQ011154.1:130195-131343;DQ011155.1:130176-131324;EPI_ISL_13331598:127794-128942;EPI_ISL_13363142:127787-128935;EPI_ISL_13374487:127802-128950;EPI_ISL_13502582:127787-128935;EPI_ISL_13508471:127879-129027;EPI_ISL_13607904:127778-128926;EPI_ISL_13624509:127722-128870;EPI_ISL_13889442:127897-129045;EPI_ISL_13953610:127695-128843;EPI_ISL_14181948:127923-129071;EPI_ISL_14838587:130049-131197;EPI_ISL_15263355:127778-128926;EPI_ISL_15269699:127747-128895;EPI_ISL_15293002:128020-129168;EPI_ISL_15641561:127736-128884;EPI_ISL_15831211:127785-128933;EPI_ISL_15992098:127800-128948;EPI_ISL_16233784:127799-128947;EPI_ISL_16946400:127785-128933;EPI_ISL_17048206:127435-128583;EPI_ISL_17703567:127800-128948;EPI_ISL_17718458:127778-128926;EPI_ISL_18213374:127775-128923;EPI_ISL_18360394:127436-128584;EPI_ISL_18557816:127776-128924;EPI_ISL_18642365:127778-128926;EPI_ISL_18659846:127748-128896;EPI_ISL_18744049:127780-128928;EPI_ISL_19022858:127775-128923;EPI_ISL_19028778:127692-128840;EPI_ISL_19151800:127776-128924;EPI_ISL_19159116:127778-128926;EPI_ISL_19350788:128920-130068;EPI_ISL_19359677:127775-128923;EPI_ISL_19446306:127770-128918;EPI_ISL_19467692:130331-131479;JX878411.1:131040-132188;JX878425.1:130557-131705;JX878426.1:130007-131155;KC257460.1:129974-131122;KJ642612.1:128291-129439;KJ642613.1:129851-130999;KJ642616.1:129438-130586;KJ642618.1:127522-128670;KP849469.1:130365-131513;KP849471.1:130291-131439;LC831698.1:127776-128924;MN648051.1:127998-129146;MN702445.1:123708-124856;MN702446.1:129853-131001;MN702447.1:123221-124369;MN702449.1:123719-124867;MN702452.1:129894-131042;MT903338.1:127941-129089;MT903340.1:127778-128926;MT903341.1:119311-120459;MT903342.1:127793-128941;MT903347.1:129767-130915;ON568298.1:127924-129072;ON622713.1:128010-129158;ON622722.2:127781-128929;ON631963.1:127801-128949;ON649879.1:127611-128759;ON676707.1:127831-128979;ON755039.1:127441-128589;ON808413.1:127781-128929;ON880420.1:128008-129156;ON911481.2:127798-128946;ON918656.1:127813-128961;ON959133.1:127902-129050;ON959136.1:127903-129051;ON983168.1:126476-127624;OP019276.1:127775-128923;OP150924.1:127916-129064;OP160532.1:127741-128889;OP235282.1:127775-128923;OP289782.1:127778-128926;OP331335.1:127795-128943;OP382479.1:127782-128930;OP422341.1:127795-128943;OP434519.1:127774-128922;OP535324.1:127776-128924;OP535333.1:127885-129033;OP536770.1:127777-128925;OP597769.1:127799-128947;OP604566.1:127778-128926;OP612682.1:127703-128851;OP612684.1:127765-128913;OP612686.1:127767-128915;OP696841.1:125052-126200;OP879722.1:127001-128149;OQ419007.1:127011-128159;OQ419012.1:126978-128126;OQ419031.1:126982-128130;OQ419047.1:126984-128132;OQ451898.1:126863-128011;OQ451900.1:127776-128924;OQ581853.1:127802-128950;OQ581855.1:127883-129031;OQ721951.1:127816-128964;OQ721954.1:127773-128921;OR035652.1:127725-128873;OR035664.1:127725-128873;OR035702.1:127725-128873;OR146272.1:127741-128889;OR146330.1:127786-128934;OR264364.1:127796-128944;OR427332.1:127796-128944;OR459777.1:127780-128928;OR459797.1:127794-128942;OR943698.1:130107-131255;PP098589.2:127777-128925;PP496048.1:127781-128929;PP601206.1:130037-131185;PP776553.1:127702-128850;PP852971.1:127775-128923;PP852983.1:127781-128929;PP853000.1:127742-128890;PP853005.1:127750-128898;PP853007.1:127783-128931;PP853013.1:127756-128904;PP853027.1:127787-128935;PP853045.1:127768-128916;PP853048.1:127787-128935;PP860026.1:127785-128933;PP860027.1:127811-128959;PP860028.1:127750-128898;PP860031.1:127804-128952;PP860033.1:127810-128958;PQ153226.1:127775-128923;PQ178862.2:128897-130045;PQ221506.1:123874-125022;PQ221833.1:130040-131188;PQ340477.1:127758-128906;PQ340478.1:127743-128891;PQ340480.1:127822-128970;PQ340483.1:127806-128954;PQ340485.1:127792-128940;PQ340486.1:127807-128955;PQ340487.1:127832-128980;PQ340489.1:127807-128955;PQ340490.1:127758-128906;PQ340491.1:127813-128961;PQ340493.1:127792-128940;PQ340495.1:127822-128970;PQ340496.1:127855-129003;PQ368489.1:127778-128926;PQ451835.1:127863-129011;PQ451836.1:127795-128943;PQ451838.1:127846-128994;PQ451841.1:127806-128954;PQ451842.1:127758-128906 |
| OG0000031 | AY603973.1:141138-141353;AY741551.1:141183-141398;AY753185.1:141381-141596;DQ011154.1:141201-141413;DQ011155.1:141170-141382;EPI_ISL_13331598:139236-139451;EPI_ISL_13363142:139223-139438;EPI_ISL_13374487:139240-139455;EPI_ISL_13502582:139235-139450;EPI_ISL_13508471:139318-139533;EPI_ISL_13607904:139214-139429;EPI_ISL_13624509:139158-139373;EPI_ISL_13889442:139335-139550;EPI_ISL_13953610:139141-139356;EPI_ISL_14181948:139364-139579;EPI_ISL_14838587:141067-141279;EPI_ISL_15263355:139217-139432;EPI_ISL_15269699:139186-139401;EPI_ISL_15293002:139455-139670;EPI_ISL_15641561:139172-139387;EPI_ISL_15831211:139225-139440;EPI_ISL_15992098:139249-139464;EPI_ISL_16233784:139238-139453;EPI_ISL_16946400:139227-139442;EPI_ISL_17048206:138874-139089;EPI_ISL_17703567:139239-139454;EPI_ISL_17718458:139209-139424;EPI_ISL_18213374:139213-139428;EPI_ISL_18360394:138872-139087;EPI_ISL_18557816:139214-139429;EPI_ISL_18642365:139211-139426;EPI_ISL_18659846:139187-139402;EPI_ISL_18744049:139226-139441;EPI_ISL_19022858:139211-139426;EPI_ISL_19028778:139128-139343;EPI_ISL_19151800:139211-139426;EPI_ISL_19159116:139214-139429;EPI_ISL_19350788:139899-140114;EPI_ISL_19359677:139211-139426;EPI_ISL_19446306:139216-139431;EPI_ISL_19467692:141340-141552;JX878411.1:142046-142258;JX878425.1:141554-141766;JX878426.1:141016-141228;KC257460.1:140947-141159;KJ642612.1:139285-139497;KJ642613.1:140836-141048;KJ642616.1:140863-141078;KJ642618.1:138472-138684;KP849469.1:141368-141580;KP849471.1:141295-141507;LC831698.1:139212-139427;MN648051.1:139438-139653;MN702445.1:134702-134914;MN702446.1:140865-141077;MN702447.1:134221-134433;MN702449.1:134728-134940;MN702452.1:140903-141115;MT903338.1:139390-139605;MT903340.1:139224-139439;MT903341.1:130758-130973;MT903342.1:139242-139457;MT903347.1:141204-141419;ON568298.1:139365-139580;ON622713.1:139446-139661;ON622722.2:139217-139432;ON631963.1:139243-139458;ON649879.1:139049-139264;ON676707.1:139270-139485;ON755039.1:138871-139086;ON808413.1:139208-139423;ON880420.1:139444-139659;ON911481.2:139229-139444;ON918656.1:139251-139466;ON959133.1:139340-139555;ON959136.1:139341-139556;ON983168.1:137916-138131;OP019276.1:139211-139426;OP150924.1:139354-139569;OP160532.1:139177-139392;OP235282.1:139211-139426;OP289782.1:139214-139429;OP331335.1:139244-139459;OP382479.1:139221-139436;OP422341.1:139234-139449;OP434519.1:139210-139425;OP535324.1:139224-139439;OP535333.1:139331-139546;OP536770.1:139214-139429;OP597769.1:139248-139463;OP604566.1:139214-139429;OP612682.1:139149-139364;OP612684.1:139200-139415;OP612686.1:139202-139417;OP696841.1:136490-136705;OP879722.1:138437-138652;OQ419007.1:138440-138655;OQ419012.1:138414-138629;OQ419031.1:138418-138633;OQ419047.1:138420-138635;OQ451898.1:138301-138516;OQ451900.1:139214-139429;OQ581853.1:139240-139455;OQ581855.1:139321-139536;OQ721951.1:139241-139456;OQ721954.1:139214-139429;OR035652.1:139160-139375;OR035664.1:139164-139379;OR035702.1:139161-139376;OR146272.1:139180-139395;OR146330.1:139224-139439;OR264364.1:139235-139450;OR427332.1:139235-139450;OR459777.1:139214-139429;OR459797.1:139233-139448;OR943698.1:141086-141298;PP098589.2:139213-139428;PP496048.1:139217-139432;PP601206.1:141046-141258;PP776553.1:139137-139352;PP852971.1:139212-139427;PP852983.1:139229-139444;PP853000.1:139180-139395;PP853005.1:139188-139403;PP853007.1:139231-139446;PP853013.1:139203-139418;PP853027.1:139236-139451;PP853045.1:139196-139411;PP853048.1:139221-139436;PP860026.1:139235-139450;PP860027.1:139289-139504;PP860028.1:139198-139413;PP860031.1:139271-139486;PP860033.1:139272-139487;PQ153226.1:139211-139426;PQ178862.2:139879-140094;PQ221506.1:134874-135086;PQ221833.1:141049-141261;PQ340477.1:139198-139413;PQ340478.1:139180-139395;PQ340480.1:139258-139473;PQ340483.1:139244-139459;PQ340485.1:139227-139442;PQ340486.1:139245-139460;PQ340487.1:139269-139484;PQ340489.1:139244-139459;PQ340490.1:139196-139411;PQ340491.1:139251-139466;PQ340493.1:139235-139450;PQ340495.1:139261-139476;PQ340496.1:139296-139511;PQ368489.1:139227-139442;PQ451835.1:139301-139516;PQ451836.1:139239-139454;PQ451838.1:139289-139504;PQ451841.1:139252-139467;PQ451842.1:139198-139413 |
| OG0000032 | AY603973.1:141681-142109;AY741551.1:141726-142154;AY753185.1:141924-142352;DQ011154.1:141741-142178;DQ011155.1:141710-142147;EPI_ISL_13331598:139779-140216;EPI_ISL_13363142:139766-140203;EPI_ISL_13374487:139783-140220;EPI_ISL_13502582:139778-140215;EPI_ISL_13508471:139861-140298;EPI_ISL_13607904:139757-140194;EPI_ISL_13624509:139701-140138;EPI_ISL_13889442:139878-140315;EPI_ISL_13953610:139684-140121;EPI_ISL_14181948:139907-140344;EPI_ISL_14838587:141607-142044;EPI_ISL_15263355:139760-140197;EPI_ISL_15269699:139729-140166;EPI_ISL_15293002:139998-140435;EPI_ISL_15641561:139715-140152;EPI_ISL_15831211:139768-140205;EPI_ISL_15992098:139792-140229;EPI_ISL_16233784:139781-140218;EPI_ISL_16946400:139770-140207;EPI_ISL_17048206:139417-139854;EPI_ISL_17703567:139782-140219;EPI_ISL_17718458:139752-140189;EPI_ISL_18213374:139756-140193;EPI_ISL_18360394:139415-139852;EPI_ISL_18557816:139757-140194;EPI_ISL_18642365:139754-140191;EPI_ISL_18659846:139730-140167;EPI_ISL_18744049:139769-140206;EPI_ISL_19022858:139754-140173;EPI_ISL_19028778:139671-140108;EPI_ISL_19151800:139754-140191;EPI_ISL_19159116:139757-140194;EPI_ISL_19350788:140442-140870;EPI_ISL_19359677:139754-140191;EPI_ISL_19446306:139759-140196;EPI_ISL_19467692:141880-142308;JX878411.1:142586-143023;JX878425.1:142094-142531;JX878426.1:141556-141984;KC257460.1:141487-141924;KJ642612.1:139825-140253;KJ642613.1:141376-141813;KJ642616.1:141406-141834;KJ642618.1:139012-139440;KP849469.1:141908-142345;KP849471.1:141835-142272;LC831698.1:139755-140192;MN648051.1:139981-140418;MN702445.1:135242-135670;MN702446.1:141405-141833;MN702447.1:134761-135207;MN702449.1:135268-135696;MN702452.1:141443-141880;MT903338.1:139933-140370;MT903340.1:139767-140204;MT903341.1:131301-131738;MT903342.1:139785-140222;MT903347.1:141747-142175;ON568298.1:139908-140345;ON622713.1:139989-140426;ON622722.2:139760-140197;ON631963.1:139786-140223;ON649879.1:139592-140029;ON676707.1:139813-140250;ON755039.1:139414-139851;ON808413.1:139751-140188;ON880420.1:139987-140424;ON911481.2:139772-140209;ON918656.1:139794-140231;ON959133.1:139883-140320;ON959136.1:139884-140321;ON983168.1:138459-138896;OP019276.1:139754-140191;OP150924.1:139897-140334;OP160532.1:139720-140157;OP235282.1:139754-140191;OP289782.1:139757-140194;OP331335.1:139787-140224;OP382479.1:139764-140201;OP422341.1:139777-140214;OP434519.1:139753-140190;OP535324.1:139767-140204;OP535333.1:139874-140311;OP536770.1:139757-140194;OP597769.1:139791-140228;OP604566.1:139757-140194;OP612682.1:139692-140129;OP612684.1:139743-140180;OP612686.1:139745-140182;OP696841.1:137033-137470;OP879722.1:138980-139417;OQ419007.1:138983-139420;OQ419012.1:138957-139394;OQ419031.1:138961-139398;OQ419047.1:138963-139400;OQ451898.1:138844-139281;OQ451900.1:139757-140194;OQ581853.1:139783-140220;OQ581855.1:139864-140301;OQ721951.1:139784-140221;OQ721954.1:139757-140194;OR035652.1:139703-140140;OR035664.1:139707-140144;OR035702.1:139704-140141;OR146272.1:139723-140160;OR146330.1:139767-140204;OR264364.1:139778-140215;OR427332.1:139778-140215;OR459777.1:139757-140194;OR459797.1:139776-140213;OR943698.1:141626-142054;PP098589.2:139756-140193;PP496048.1:139760-140197;PP601206.1:141586-142014;PP776553.1:139680-140117;PP852971.1:139755-140192;PP852983.1:139772-140209;PP853000.1:139723-140160;PP853005.1:139731-140168;PP853007.1:139774-140211;PP853013.1:139746-140183;PP853027.1:139779-140216;PP853045.1:139739-140176;PP853048.1:139764-140201;PP860026.1:139778-140206;PP860027.1:139832-140260;PP860028.1:139741-140169;PP860031.1:139814-140242;PP860033.1:139815-140243;PQ153226.1:139754-140191;PQ178862.2:140422-140850;PQ221506.1:135414-135842;PQ221833.1:141589-142017;PQ340477.1:139741-140178;PQ340478.1:139723-140160;PQ340480.1:139801-140238;PQ340483.1:139787-140224;PQ340485.1:139770-140207;PQ340486.1:139788-140225;PQ340487.1:139812-140249;PQ340489.1:139787-140224;PQ340490.1:139739-140176;PQ340491.1:139794-140231;PQ340493.1:139778-140215;PQ340495.1:139804-140241;PQ340496.1:139839-140276;PQ368489.1:139770-140207;PQ451835.1:139844-140281;PQ451836.1:139782-140219;PQ451838.1:139832-140269;PQ451841.1:139795-140232;PQ451842.1:139741-140178 |
| OG0000033 | AY603973.1:143556-144062;AY741551.1:143601-144107;AY753185.1:143799-144305;DQ011154.1:143625-144131;DQ011155.1:143594-144100;EPI_ISL_13331598:141663-142169;EPI_ISL_13363142:141650-142156;EPI_ISL_13374487:141667-142173;EPI_ISL_13502582:141662-142168;EPI_ISL_13508471:141745-142251;EPI_ISL_13607904:141641-142147;EPI_ISL_13624509:141585-142091;EPI_ISL_13889442:141762-142268;EPI_ISL_13953610:141568-142074;EPI_ISL_14181948:141791-142297;EPI_ISL_14838587:143491-143997;EPI_ISL_15263355:141644-142150;EPI_ISL_15269699:141613-142119;EPI_ISL_15293002:141882-142388;EPI_ISL_15641561:141599-142105;EPI_ISL_15831211:141652-142158;EPI_ISL_15992098:141676-142182;EPI_ISL_16233784:141665-142171;EPI_ISL_16946400:141654-142160;EPI_ISL_17048206:141301-141807;EPI_ISL_17703567:141666-142172;EPI_ISL_17718458:141636-142142;EPI_ISL_18213374:141640-142146;EPI_ISL_18360394:141424-141804;EPI_ISL_18557816:141641-142147;EPI_ISL_18642365:141638-142144;EPI_ISL_18659846:141614-142120;EPI_ISL_18744049:141653-142159;EPI_ISL_19022858:141620-142126;EPI_ISL_19028778:141555-142061;EPI_ISL_19151800:141638-142144;EPI_ISL_19159116:141641-142147;EPI_ISL_19350788:142317-142823;EPI_ISL_19359677:141638-142144;EPI_ISL_19446306:141643-142149;EPI_ISL_19467692:143755-144261;JX878411.1:144470-144976;JX878425.1:143978-144484;JX878426.1:143431-143937;KC257460.1:143371-143877;KJ642612.1:141700-142206;KJ642613.1:143260-143766;KJ642616.1:143281-143787;KJ642618.1:140887-141393;KP849469.1:143792-144298;KP849471.1:143719-144225;LC831698.1:141639-142145;MN648051.1:141865-142371;MN702445.1:137117-137623;MN702446.1:143280-143786;MN702447.1:136654-137160;MN702449.1:137143-137649;MN702452.1:143327-143833;MT903338.1:141817-142323;MT903340.1:141651-142157;MT903341.1:133185-133691;MT903342.1:141669-142175;MT903347.1:143622-144128;ON568298.1:141792-142298;ON622713.1:141873-142379;ON622722.2:141644-142150;ON631963.1:141670-142176;ON649879.1:141476-141982;ON676707.1:141697-142203;ON755039.1:141298-141804;ON808413.1:141635-142141;ON880420.1:141871-142377;ON911481.2:141656-142162;ON918656.1:141678-142184;ON959133.1:141767-142273;ON959136.1:141768-142274;ON983168.1:140343-140849;OP019276.1:141638-142144;OP150924.1:141781-142287;OP160532.1:141604-142110;OP235282.1:141638-142144;OP289782.1:141641-142147;OP331335.1:141671-142177;OP382479.1:141648-142154;OP422341.1:141661-142167;OP434519.1:141637-142143;OP535324.1:141651-142157;OP535333.1:141758-142264;OP536770.1:141641-142147;OP597769.1:141675-142181;OP604566.1:141641-142147;OP612682.1:141576-142082;OP612684.1:141627-142133;OP612686.1:141629-142135;OP696841.1:138917-139423;OP879722.1:140864-141370;OQ419007.1:140867-141373;OQ419012.1:140841-141347;OQ419031.1:140845-141351;OQ419047.1:140847-141353;OQ451898.1:140728-141234;OQ451900.1:141641-142147;OQ581853.1:141667-142173;OQ581855.1:141748-142254;OQ721951.1:141668-142174;OQ721954.1:141641-142147;OR035652.1:141587-142093;OR035664.1:141591-142097;OR035702.1:141588-142094;OR146272.1:141607-142113;OR146330.1:141651-142157;OR264364.1:141662-142168;OR427332.1:141662-142168;OR459777.1:141641-142147;OR459797.1:141660-142166;OR943698.1:143501-144007;PP098589.2:141640-142146;PP496048.1:141644-142150;PP601206.1:143461-143967;PP776553.1:141564-142070;PP852971.1:141639-142145;PP852983.1:141656-142162;PP853000.1:141607-142113;PP853005.1:141615-142121;PP853007.1:141658-142164;PP853013.1:141630-142136;PP853027.1:141663-142169;PP853045.1:141623-142129;PP853048.1:141648-142154;PP860026.1:141653-142159;PP860027.1:141707-142213;PP860028.1:141616-142122;PP860031.1:141689-142195;PP860033.1:141690-142196;PQ153226.1:141638-142144;PQ178862.2:142297-142803;PQ221506.1:137289-137795;PQ221833.1:143464-143970;PQ340477.1:141625-142131;PQ340478.1:141607-142113;PQ340480.1:141685-142191;PQ340483.1:141671-142177;PQ340485.1:141654-142160;PQ340486.1:141672-142178;PQ340487.1:141696-142202;PQ340489.1:141671-142177;PQ340490.1:141623-142129;PQ340491.1:141678-142184;PQ340493.1:141662-142168;PQ340495.1:141688-142194;PQ340496.1:141723-142229;PQ368489.1:141654-142160;PQ451835.1:141728-142234;PQ451836.1:141666-142172;PQ451838.1:141716-142222;PQ451841.1:141679-142185;PQ451842.1:141625-142131 |
| OG0000034 | AY603973.1:149952-150176;AY741551.1:150009-150233;AY753185.1:150207-150431;DQ011154.1:150051-150275;DQ011155.1:150016-150240;EPI_ISL_13331598:148048-148272;EPI_ISL_13363142:148035-148259;EPI_ISL_13374487:148052-148276;EPI_ISL_13502582:148047-148271;EPI_ISL_13508471:148130-148354;EPI_ISL_13607904:148026-148250;EPI_ISL_13624509:147970-148194;EPI_ISL_13889442:148147-148371;EPI_ISL_13953610:147953-148177;EPI_ISL_14181948:148176-148400;EPI_ISL_14838587:149914-150138;EPI_ISL_15263355:148029-148253;EPI_ISL_15269699:147998-148222;EPI_ISL_15293002:148267-148491;EPI_ISL_15641561:147984-148208;EPI_ISL_15831211:148037-148261;EPI_ISL_15992098:148061-148285;EPI_ISL_16233784:148050-148274;EPI_ISL_16946400:148039-148263;EPI_ISL_17048206:147686-147910;EPI_ISL_17703567:148051-148275;EPI_ISL_17718458:148021-148245;EPI_ISL_18213374:148025-148249;EPI_ISL_18360394:147365-147556;EPI_ISL_18557816:148026-148250;EPI_ISL_18642365:148023-148247;EPI_ISL_18659846:147999-148223;EPI_ISL_18744049:148038-148262;EPI_ISL_19022858:148005-148229;EPI_ISL_19028778:147940-148164;EPI_ISL_19151800:148023-148247;EPI_ISL_19159116:148026-148250;EPI_ISL_19350788:148690-148914;EPI_ISL_19359677:148023-148247;EPI_ISL_19446306:148028-148252;EPI_ISL_19467692:150184-150408;JX878411.1:150897-151121;JX878425.1:150401-150625;JX878426.1:149839-150063;KC257460.1:149799-150023;KJ642612.1:148096-148320;KJ642613.1:149680-149904;KJ642616.1:149682-149906;KJ642618.1:147294-147518;KP849469.1:150218-150442;KP849471.1:150148-150372;LC831698.1:148024-148248;MN648051.1:148250-148474;MN702445.1:143548-143772;MN702446.1:149711-149935;MN702447.1:143083-143307;MN702449.1:143585-143809;MN702452.1:149753-149977;MT903338.1:148183-148407;MT903340.1:148036-148260;MT903341.1:139577-139801;MT903342.1:148073-148297;MT903347.1:150030-150254;ON568298.1:148177-148401;ON622713.1:148258-148482;ON622722.2:148029-148253;ON631963.1:148055-148279;ON649879.1:147861-148085;ON676707.1:148082-148306;ON755039.1:147683-147907;ON808413.1:148020-148244;ON880420.1:148256-148480;ON911481.2:148041-148265;ON918656.1:148063-148287;ON959133.1:148152-148376;ON959136.1:148153-148377;ON983168.1:146728-146952;OP019276.1:148023-148247;OP150924.1:148166-148390;OP160532.1:147989-148213;OP235282.1:148023-148247;OP289782.1:148026-148250;OP331335.1:148056-148280;OP382479.1:148033-148257;OP422341.1:148046-148270;OP434519.1:148022-148246;OP535324.1:148036-148260;OP535333.1:148143-148367;OP536770.1:148026-148250;OP597769.1:148060-148284;OP604566.1:148026-148250;OP612682.1:147961-148185;OP612684.1:148012-148236;OP612686.1:148014-148238;OP696841.1:145302-145526;OP879722.1:147249-147473;OQ419007.1:147252-147476;OQ419012.1:147226-147450;OQ419031.1:147230-147454;OQ419047.1:147232-147456;OQ451898.1:147113-147337;OQ451900.1:148026-148250;OQ581853.1:148052-148276;OQ581855.1:148133-148357;OQ721951.1:148053-148277;OQ721954.1:148026-148250;OR035652.1:147972-148196;OR035664.1:147976-148200;OR035702.1:147973-148197;OR146272.1:147992-148216;OR146330.1:148036-148260;OR264364.1:148047-148271;OR427332.1:148047-148271;OR459777.1:148026-148250;OR459797.1:148045-148269;OR943698.1:149906-150130;PP098589.2:148025-148249;PP496048.1:148029-148253;PP601206.1:149865-150089;PP776553.1:147949-148173;PP852971.1:148024-148248;PP852983.1:148041-148265;PP853000.1:147992-148216;PP853005.1:148000-148224;PP853007.1:148043-148267;PP853013.1:148015-148239;PP853027.1:148048-148272;PP853045.1:148008-148232;PP853048.1:148033-148257;PP860026.1:148030-148254;PP860027.1:148073-148297;PP860028.1:147993-148217;PP860031.1:148055-148279;PP860033.1:148056-148280;PQ153226.1:148023-148247;PQ178862.2:148669-148893;PQ221506.1:143685-143909;PQ221833.1:149859-150083;PQ340477.1:148010-148234;PQ340478.1:147992-148216;PQ340480.1:148070-148294;PQ340483.1:148056-148280;PQ340485.1:148039-148263;PQ340486.1:148057-148281;PQ340487.1:148081-148305;PQ340489.1:148056-148280;PQ340490.1:148008-148232;PQ340491.1:148063-148287;PQ340493.1:148047-148271;PQ340495.1:148073-148297;PQ340496.1:148108-148332;PQ368489.1:148039-148263;PQ451835.1:148113-148337;PQ451836.1:148051-148275;PQ451838.1:148101-148325;PQ451841.1:148064-148288;PQ451842.1:148010-148234 |
| OG0000035 | AY603973.1:153384-153998;AY741551.1:153458-154072;AY753185.1:153639-154253;DQ011154.1:153544-154158;DQ011155.1:153486-154100;EPI_ISL_13331598:151503-152117;EPI_ISL_13363142:151491-152105;EPI_ISL_13374487:151524-152138;EPI_ISL_13502582:151519-152133;EPI_ISL_13508471:151585-152199;EPI_ISL_13607904:151490-152104;EPI_ISL_13624509:151425-152039;EPI_ISL_13889442:151618-152232;EPI_ISL_13953610:151418-152032;EPI_ISL_14181948:151648-152262;EPI_ISL_14838587:153483-154097;EPI_ISL_15263355:151484-152098;EPI_ISL_15269699:151453-152067;EPI_ISL_15293002:151722-152336;EPI_ISL_15641561:151439-152053;EPI_ISL_15831211:151493-152107;EPI_ISL_15992098:151533-152147;EPI_ISL_16233784:151505-152119;EPI_ISL_16946400:151495-152109;EPI_ISL_17048206:151159-151773;EPI_ISL_17703567:151506-152120;EPI_ISL_17718458:151486-152100;EPI_ISL_18213374:151497-152111;EPI_ISL_18360394:150804-151418;EPI_ISL_18557816:151500-152114;EPI_ISL_18642365:151495-152109;EPI_ISL_18659846:151454-152068;EPI_ISL_18744049:151503-152117;EPI_ISL_19022858:151460-152074;EPI_ISL_19028778:151412-152026;EPI_ISL_19151800:151496-152110;EPI_ISL_19159116:151481-152095;EPI_ISL_19350788:152143-152757;EPI_ISL_19359677:151496-152110;EPI_ISL_19446306:151483-152097;EPI_ISL_19467692:153684-154298;JX878411.1:154385-154999;JX878425.1:153873-154487;JX878426.1:153413-154027;KC257460.1:153269-153883;KJ642612.1:151603-152217;KJ642613.1:153152-153766;KJ642616.1:153106-153720;KJ642618.1:151068-151682;KP849469.1:153701-154315;KP849471.1:153634-154248;LC831698.1:151479-152093;MN648051.1:151722-152336;MN702445.1:147020-147634;MN702446.1:153184-153798;MN702447.1:146547-147161;MN702449.1:147058-147672;MN702452.1:153235-153849;MT903338.1:151655-152269;MT903340.1:151508-152122;MT903341.1:143049-143663;MT903342.1:151545-152159;MT903347.1:153451-154065;ON568298.1:151649-152263;ON622713.1:151713-152327;ON622722.2:151501-152115;ON631963.1:151510-152124;ON649879.1:151316-151930;ON676707.1:151547-152161;ON755039.1:151138-151752;ON808413.1:151475-152089;ON880420.1:151711-152325;ON911481.2:151496-152110;ON918656.1:151518-152132;ON959133.1:151607-152221;ON959136.1:151608-152222;ON983168.1:150184-150798;OP019276.1:151495-152109;OP150924.1:151621-152235;OP160532.1:151444-152058;OP235282.1:151495-152109;OP289782.1:151499-152113;OP331335.1:151504-152118;OP382479.1:151488-152102;OP422341.1:151501-152115;OP434519.1:151477-152091;OP535324.1:151508-152122;OP535333.1:151615-152229;OP536770.1:151498-152112;OP597769.1:151532-152146;OP604566.1:151498-152112;OP612682.1:151433-152047;OP612684.1:151484-152098;OP612686.1:151486-152100;OP696841.1:148774-149388;OP879722.1:150721-151335;OQ419007.1:150715-151329;OQ419012.1:150689-151303;OQ419031.1:150693-151307;OQ419047.1:150695-151309;OQ451898.1:150585-151199;OQ451900.1:151499-152113;OQ581853.1:151507-152121;OQ581855.1:151606-152220;OQ721951.1:151525-152139;OQ721954.1:151498-152112;OR035652.1:151427-152041;OR035664.1:151431-152045;OR035702.1:151428-152042;OR146272.1:151447-152061;OR146330.1:151508-152122;OR264364.1:151502-152116;OR427332.1:151502-152116;OR459777.1:151491-152105;OR459797.1:151500-152114;OR943698.1:153389-154003;PP098589.2:151497-152111;PP496048.1:151501-152115;PP601206.1:153444-154058;PP776553.1:151421-152035;PP852971.1:151489-152103;PP852983.1:151506-152120;PP853000.1:151457-152071;PP853005.1:151465-152079;PP853007.1:151508-152122;PP853013.1:151480-152094;PP853027.1:151522-152136;PP853045.1:151473-152087;PP853048.1:151498-152112;PP860026.1:151493-152107;PP860027.1:151530-152144;PP860028.1:151456-152070;PP860031.1:151527-152141;PP860033.1:151513-152127;PQ153226.1:151478-152092;PQ178862.2:152122-152736;PQ221506.1:147233-147847;PQ221833.1:153420-154034;PQ340477.1:151465-152079;PQ340478.1:151447-152061;PQ340480.1:151525-152139;PQ340483.1:151511-152125;PQ340485.1:151494-152108;PQ340486.1:151512-152126;PQ340487.1:151536-152150;PQ340489.1:151511-152125;PQ340490.1:151463-152077;PQ340491.1:151518-152132;PQ340493.1:151502-152116;PQ340495.1:151528-152142;PQ340496.1:151563-152177;PQ368489.1:151511-152125;PQ451835.1:151568-152182;PQ451836.1:151506-152120;PQ451838.1:151556-152170;PQ451841.1:151519-152133;PQ451842.1:151465-152079 |
| OG0000036 | AY603973.1:154569-156248;AY741551.1:154641-156320;AY753185.1:154824-156503;DQ011154.1:154724-156388;DQ011155.1:154666-156330;EPI_ISL_13331598:152686-154365;EPI_ISL_13363142:152674-154353;EPI_ISL_13374487:152707-154386;EPI_ISL_13502582:152702-154381;EPI_ISL_13508471:152768-154447;EPI_ISL_13607904:152673-154352;EPI_ISL_13624509:152608-154287;EPI_ISL_13889442:152801-154480;EPI_ISL_13953610:152601-154280;EPI_ISL_14181948:152831-154510;EPI_ISL_14838587:154663-156327;EPI_ISL_15263355:152667-154346;EPI_ISL_15269699:152636-154315;EPI_ISL_15293002:152905-154584;EPI_ISL_15641561:152622-154301;EPI_ISL_15831211:152676-154355;EPI_ISL_15992098:152716-154395;EPI_ISL_16233784:152688-154367;EPI_ISL_16946400:152678-154357;EPI_ISL_17048206:152342-154021;EPI_ISL_17703567:152689-154368;EPI_ISL_17718458:152669-154348;EPI_ISL_18213374:152680-154359;EPI_ISL_18360394:151987-153666;EPI_ISL_18557816:152683-154362;EPI_ISL_18642365:152678-154357;EPI_ISL_18659846:152637-154316;EPI_ISL_18744049:152686-154365;EPI_ISL_19022858:152643-154322;EPI_ISL_19028778:152595-154274;EPI_ISL_19151800:152679-154358;EPI_ISL_19159116:152664-154343;EPI_ISL_19350788:153326-154990;EPI_ISL_19359677:152679-154358;EPI_ISL_19446306:152666-154345;EPI_ISL_19467692:154864-156528;JX878411.1:155565-157229;JX878425.1:155053-156717;JX878426.1:154593-156257;KC257460.1:154449-156113;KJ642612.1:152783-154447;KJ642613.1:154332-155996;KJ642616.1:154290-155969;KJ642618.1:152249-153913;KP849469.1:154881-156545;KP849471.1:154816-156480;LC831698.1:152662-154341;MN648051.1:152905-154584;MN702445.1:148200-149864;MN702446.1:154364-156028;MN702447.1:147727-149391;MN702449.1:148238-149902;MN702452.1:154415-156079;MT903338.1:152838-154517;MT903340.1:152691-154370;MT903341.1:144232-145911;MT903342.1:152728-154407;MT903347.1:154634-156313;ON568298.1:152832-154511;ON622713.1:152896-154575;ON622722.2:152684-154363;ON631963.1:152693-154372;ON649879.1:152499-154178;ON676707.1:152730-154409;ON755039.1:152321-154000;ON808413.1:152658-154337;ON880420.1:152894-154573;ON911481.2:152679-154358;ON918656.1:152701-154380;ON959133.1:152790-154469;ON959136.1:152791-154470;ON983168.1:151367-153046;OP019276.1:152678-154357;OP150924.1:152804-154483;OP160532.1:152627-154306;OP235282.1:152678-154357;OP289782.1:152682-154361;OP331335.1:152687-154366;OP382479.1:152671-154350;OP422341.1:152684-154363;OP434519.1:152660-154339;OP535324.1:152691-154370;OP535333.1:152798-154477;OP536770.1:152681-154360;OP597769.1:152715-154394;OP604566.1:152681-154360;OP612682.1:152616-154295;OP612684.1:152667-154346;OP612686.1:152669-154348;OP696841.1:149957-151636;OP879722.1:151904-153583;OQ419007.1:151898-153577;OQ419012.1:151872-153551;OQ419031.1:151876-153555;OQ419047.1:151878-153557;OQ451898.1:151768-153447;OQ451900.1:152682-154361;OQ581853.1:152690-154369;OQ581855.1:152789-154468;OQ721951.1:152708-154387;OQ721954.1:152681-154360;OR035652.1:152610-154289;OR035664.1:152614-154293;OR035702.1:152611-154290;OR146272.1:152630-154309;OR146330.1:152691-154370;OR264364.1:152685-154364;OR427332.1:152685-154364;OR459777.1:152674-154353;OR459797.1:152683-154362;OR943698.1:154569-156233;PP098589.2:152680-154359;PP496048.1:152684-154363;PP601206.1:154624-156288;PP776553.1:152604-154283;PP852971.1:152672-154351;PP852983.1:152689-154368;PP853000.1:152640-154319;PP853005.1:152648-154327;PP853007.1:152691-154370;PP853013.1:152663-154342;PP853027.1:152705-154384;PP853045.1:152656-154335;PP853048.1:152681-154360;PP860026.1:152676-154355;PP860027.1:152713-154392;PP860028.1:152639-154318;PP860031.1:152710-154389;PP860033.1:152696-154375;PQ153226.1:152661-154340;PQ178862.2:153305-154969;PQ221506.1:148413-150077;PQ221833.1:154600-156264;PQ340477.1:152648-154327;PQ340478.1:152630-154309;PQ340480.1:152708-154387;PQ340483.1:152694-154373;PQ340485.1:152677-154356;PQ340486.1:152695-154374;PQ340487.1:152719-154398;PQ340489.1:152694-154373;PQ340490.1:152646-154325;PQ340491.1:152701-154380;PQ340493.1:152685-154364;PQ340495.1:152711-154390;PQ340496.1:152746-154425;PQ368489.1:152694-154373;PQ451835.1:152751-154430;PQ451836.1:152689-154368;PQ451838.1:152739-154418;PQ451841.1:152702-154381;PQ451842.1:152648-154327 |
| OG0000037 | AY603973.1:160293-160466;AY741551.1:160366-160539;AY753185.1:160548-160721;DQ011154.1:158394-158642;DQ011155.1:158336-158584;EPI_ISL_13331598:158430-158861;EPI_ISL_13363142:158418-158849;EPI_ISL_13374487:158451-158882;EPI_ISL_13502582:158446-158877;EPI_ISL_13508471:158512-158943;EPI_ISL_13607904:158417-158848;EPI_ISL_13624509:158352-158783;EPI_ISL_13889442:158545-158976;EPI_ISL_13953610:158345-158776;EPI_ISL_14181948:158575-159006;EPI_ISL_14838587:158333-158581;EPI_ISL_15263355:158411-158842;EPI_ISL_15269699:158380-158811;EPI_ISL_15293002:158649-159080;EPI_ISL_15641561:158366-158797;EPI_ISL_15831211:158420-158851;EPI_ISL_15992098:158460-158891;EPI_ISL_16233784:158432-158863;EPI_ISL_16946400:158422-158853;EPI_ISL_17048206:158086-158517;EPI_ISL_17703567:158433-158864;EPI_ISL_17718458:158413-158844;EPI_ISL_18213374:158424-158855;EPI_ISL_18360394:157731-158162;EPI_ISL_18557816:158427-158858;EPI_ISL_18642365:158422-158853;EPI_ISL_18659846:158381-158812;EPI_ISL_18744049:158430-158861;EPI_ISL_19022858:158387-158818;EPI_ISL_19028778:158339-158770;EPI_ISL_19151800:158423-158854;EPI_ISL_19159116:158408-158839;EPI_ISL_19350788:156991-157239;EPI_ISL_19359677:158423-158854;EPI_ISL_19446306:158410-158841;EPI_ISL_19467692:158534-158782;JX878411.1:159236-159484;JX878425.1:158723-158971;JX878426.1:158263-158475;KC257460.1:158119-158367;KJ642612.1:156453-156701;KJ642613.1:158002-158250;KJ642616.1:160014-160187;KJ642618.1:155919-156167;KP849469.1:158551-158796;KP849471.1:158487-158735;LC831698.1:158406-158837;MN648051.1:158649-159080;MN702445.1:151870-152118;MN702446.1:158034-158282;MN702447.1:151397-151645;MN702449.1:151908-152156;MN702452.1:158085-158333;MT903338.1:158582-159013;MT903340.1:158435-158866;MT903341.1:149976-150407;MT903342.1:158472-158903;MT903347.1:160362-160535;ON568298.1:158576-159007;ON622713.1:158640-159071;ON622722.2:158428-158859;ON631963.1:158437-158868;ON649879.1:158243-158674;ON676707.1:158474-158905;ON755039.1:158065-158496;ON808413.1:158402-158833;ON880420.1:158638-159069;ON911481.2:158423-158854;ON918656.1:158445-158876;ON959133.1:158534-158965;ON959136.1:158535-158966;ON983168.1:157111-157542;OP019276.1:158422-158853;OP150924.1:158548-158979;OP160532.1:158371-158802;OP235282.1:158422-158853;OP289782.1:158426-158857;OP331335.1:158431-158862;OP382479.1:158415-158846;OP422341.1:158428-158859;OP434519.1:158404-158835;OP535324.1:158435-158866;OP535333.1:158542-158973;OP536770.1:158425-158856;OP597769.1:158459-158890;OP604566.1:158425-158856;OP612682.1:158360-158791;OP612684.1:158411-158842;OP612686.1:158413-158844;OP696841.1:155701-156132;OP879722.1:157648-158079;OQ419007.1:157642-158073;OQ419012.1:157616-158047;OQ419031.1:157620-158051;OQ419047.1:157622-158053;OQ451898.1:157512-157943;OQ451900.1:158426-158857;OQ581853.1:158434-158865;OQ581855.1:158533-158964;OQ721951.1:158452-158883;OQ721954.1:158425-158856;OR035652.1:158354-158785;OR035664.1:158358-158789;OR035702.1:158355-158786;OR146272.1:158374-158805;OR146330.1:158435-158866;OR264364.1:158429-158860;OR427332.1:158429-158860;OR459777.1:158418-158849;OR459797.1:158427-158858;OR943698.1:158239-158487;PP098589.2:158424-158855;PP496048.1:158428-158859;PP601206.1:158294-158542;PP776553.1:158348-158779;PP852971.1:158416-158847;PP852983.1:158433-158864;PP853000.1:158384-158815;PP853005.1:158392-158823;PP853007.1:158435-158866;PP853013.1:158407-158838;PP853027.1:158449-158880;PP853045.1:158400-158831;PP853048.1:158425-158856;PP860026.1:158420-158851;PP860027.1:158456-158887;PP860028.1:158383-158814;PP860031.1:158437-158868;PP860033.1:158439-158870;PQ153226.1:158405-158836;PQ178862.2:156970-157218;PQ221506.1:152083-152295;PQ221833.1:158270-158518;PQ340477.1:158392-158823;PQ340478.1:158374-158805;PQ340480.1:158452-158883;PQ340483.1:158438-158869;PQ340485.1:158421-158852;PQ340486.1:158439-158870;PQ340487.1:158463-158894;PQ340489.1:158438-158869;PQ340490.1:158390-158821;PQ340491.1:158445-158876;PQ340493.1:158429-158860;PQ340495.1:158455-158886;PQ340496.1:158490-158921;PQ368489.1:158438-158869;PQ451835.1:158495-158926;PQ451836.1:158433-158864;PQ451838.1:158483-158914;PQ451841.1:158446-158877;PQ451842.1:158392-158823 |
| OG0000038 | AY603973.1:160776-161717;AY741551.1:160849-161790;AY753185.1:161031-161972;DQ011154.1:158692-159633;DQ011155.1:158634-159575;EPI_ISL_13331598:158911-159852;EPI_ISL_13363142:158899-159840;EPI_ISL_13374487:158932-159873;EPI_ISL_13502582:158927-159868;EPI_ISL_13508471:158993-159934;EPI_ISL_13607904:158898-159839;EPI_ISL_13624509:158833-159774;EPI_ISL_13889442:159026-159967;EPI_ISL_13953610:158826-159767;EPI_ISL_14181948:159056-159997;EPI_ISL_14838587:158631-159572;EPI_ISL_15263355:158892-159833;EPI_ISL_15269699:158861-159802;EPI_ISL_15293002:159130-160071;EPI_ISL_15641561:158847-159788;EPI_ISL_15831211:158901-159842;EPI_ISL_15992098:158941-159882;EPI_ISL_16233784:158913-159854;EPI_ISL_16946400:158903-159844;EPI_ISL_17048206:158567-159508;EPI_ISL_17703567:158914-159855;EPI_ISL_17718458:158894-159835;EPI_ISL_18213374:158905-159846;EPI_ISL_18360394:158212-159153;EPI_ISL_18557816:158908-159849;EPI_ISL_18642365:158903-159844;EPI_ISL_18659846:158862-159803;EPI_ISL_18744049:158911-159852;EPI_ISL_19022858:158868-159809;EPI_ISL_19028778:158820-159761;EPI_ISL_19151800:158904-159845;EPI_ISL_19159116:158889-159830;EPI_ISL_19350788:157289-158230;EPI_ISL_19359677:158904-159845;EPI_ISL_19446306:158891-159832;EPI_ISL_19467692:158832-159773;JX878411.1:159534-160475;JX878425.1:159021-159962;JX878426.1:158562-159503;KC257460.1:158417-159358;KJ642612.1:156751-157692;KJ642613.1:158300-159241;KJ642616.1:160497-161438;KJ642618.1:156217-157158;KP849469.1:158850-159791;KP849471.1:158785-159726;LC831698.1:158887-159828;MN648051.1:159130-160071;MN702445.1:152168-153109;MN702446.1:158332-159273;MN702447.1:151695-152636;MN702449.1:152206-153147;MN702452.1:158383-159324;MT903338.1:159063-160004;MT903340.1:158916-159857;MT903341.1:150457-151398;MT903342.1:158953-159894;MT903347.1:160845-161786;ON568298.1:159057-159998;ON622713.1:159121-160062;ON622722.2:158909-159850;ON631963.1:158918-159859;ON649879.1:158724-159665;ON676707.1:158955-159896;ON755039.1:158546-159487;ON808413.1:158883-159824;ON880420.1:159119-160060;ON911481.2:158904-159845;ON918656.1:158926-159867;ON959133.1:159015-159956;ON959136.1:159016-159957;ON983168.1:157592-158533;OP019276.1:158903-159844;OP150924.1:159029-159970;OP160532.1:158852-159793;OP235282.1:158903-159844;OP289782.1:158907-159848;OP331335.1:158912-159853;OP382479.1:158896-159837;OP422341.1:158909-159850;OP434519.1:158885-159826;OP535324.1:158916-159857;OP535333.1:159023-159964;OP536770.1:158906-159847;OP597769.1:158940-159881;OP604566.1:158906-159847;OP612682.1:158841-159782;OP612684.1:158892-159833;OP612686.1:158894-159835;OP696841.1:156182-157123;OP879722.1:158129-159070;OQ419007.1:158123-159064;OQ419012.1:158097-159038;OQ419031.1:158101-159042;OQ419047.1:158103-159044;OQ451898.1:157993-158934;OQ451900.1:158907-159848;OQ581853.1:158915-159856;OQ581855.1:159014-159955;OQ721951.1:158933-159874;OQ721954.1:158906-159847;OR035652.1:158835-159776;OR035664.1:158839-159780;OR035702.1:158836-159777;OR146272.1:158855-159796;OR146330.1:158916-159857;OR264364.1:158910-159851;OR427332.1:158910-159851;OR459777.1:158899-159840;OR459797.1:158908-159849;OR943698.1:158537-159478;PP098589.2:158905-159846;PP496048.1:158909-159850;PP601206.1:158592-159533;PP776553.1:158829-159770;PP852971.1:158897-159838;PP852983.1:158914-159855;PP853000.1:158865-159806;PP853005.1:158873-159814;PP853007.1:158916-159857;PP853013.1:158888-159829;PP853027.1:158930-159871;PP853045.1:158881-159822;PP853048.1:158906-159847;PP860026.1:158901-159842;PP860027.1:158937-159878;PP860028.1:158864-159805;PP860031.1:158918-159859;PP860033.1:158920-159861;PQ153226.1:158886-159827;PQ178862.2:157268-158209;PQ221506.1:152382-153323;PQ221833.1:158568-159509;PQ340477.1:158873-159814;PQ340478.1:158855-159796;PQ340480.1:158933-159874;PQ340483.1:158919-159860;PQ340485.1:158902-159843;PQ340486.1:158920-159861;PQ340487.1:158944-159885;PQ340489.1:158919-159860;PQ340490.1:158871-159812;PQ340491.1:158926-159867;PQ340493.1:158910-159851;PQ340495.1:158936-159877;PQ340496.1:158971-159912;PQ368489.1:158919-159860;PQ451835.1:158976-159917;PQ451836.1:158914-159855;PQ451838.1:158964-159905;PQ451841.1:158927-159868;PQ451842.1:158873-159814 |
| OG0000039 | AY603973.1:166989-167942;AY741551.1:167023-167976;AY753185.1:167252-168205;DQ011154.1:164892-165845;DQ011155.1:164834-165787;EPI_ISL_13331598:165116-166069;EPI_ISL_13363142:165104-166057;EPI_ISL_13374487:165137-166090;EPI_ISL_13502582:165132-166085;EPI_ISL_13508471:165198-166151;EPI_ISL_13607904:165103-166056;EPI_ISL_13624509:165038-165991;EPI_ISL_13889442:165231-166184;EPI_ISL_13953610:165031-165984;EPI_ISL_14181948:165261-166214;EPI_ISL_14838587:164846-165799;EPI_ISL_15263355:165097-166050;EPI_ISL_15269699:165066-166019;EPI_ISL_15293002:165335-166288;EPI_ISL_15641561:165052-166005;EPI_ISL_15831211:165106-166059;EPI_ISL_15992098:165146-166099;EPI_ISL_16233784:165118-166071;EPI_ISL_16946400:165108-166061;EPI_ISL_17048206:164772-165725;EPI_ISL_17703567:165119-166072;EPI_ISL_17718458:165099-166052;EPI_ISL_18213374:165110-166063;EPI_ISL_18360394:164417-165370;EPI_ISL_18557816:165113-166066;EPI_ISL_18642365:165108-166061;EPI_ISL_18659846:165067-166020;EPI_ISL_18744049:165116-166069;EPI_ISL_19022858:165073-166026;EPI_ISL_19028778:165025-165978;EPI_ISL_19151800:165109-166062;EPI_ISL_19159116:165094-166047;EPI_ISL_19350788:163485-164438;EPI_ISL_19359677:165109-166062;EPI_ISL_19446306:165096-166049;EPI_ISL_19467692:165032-165985;JX878411.1:165734-166687;JX878425.1:165221-166174;JX878426.1:164791-165744;KC257460.1:164617-165570;KJ642612.1:162951-163904;KJ642613.1:164500-165453;KJ642616.1:166708-167661;KJ642618.1:162417-163370;KP849469.1:165050-166003;KP849471.1:164985-165938;LC831698.1:165092-166045;MN648051.1:165335-166288;MN702445.1:158368-159321;MN702446.1:164532-165485;MN702447.1:157895-158848;MN702449.1:158406-159359;MN702452.1:164583-165536;MT903338.1:165268-166221;MT903340.1:165121-166074;MT903341.1:156662-157615;MT903342.1:165158-166111;MT903347.1:167058-168011;ON568298.1:165262-166215;ON622713.1:165326-166279;ON622722.2:165114-166067;ON631963.1:165123-166076;ON649879.1:164929-165882;ON676707.1:165160-166113;ON755039.1:164751-165704;ON808413.1:165088-166041;ON880420.1:165324-166277;ON911481.2:165109-166062;ON918656.1:165131-166084;ON959133.1:165220-166173;ON959136.1:165221-166174;ON983168.1:163797-164750;OP019276.1:165108-166061;OP150924.1:165234-166187;OP160532.1:165057-166010;OP235282.1:165108-166061;OP289782.1:165112-166065;OP331335.1:165117-166070;OP382479.1:165101-166054;OP422341.1:165114-166067;OP434519.1:165090-166043;OP535324.1:165121-166074;OP535333.1:165228-166181;OP536770.1:165111-166064;OP597769.1:165145-166098;OP604566.1:165111-166064;OP612682.1:165046-165999;OP612684.1:165097-166050;OP612686.1:165099-166052;OP696841.1:162387-163340;OP879722.1:164334-165287;OQ419007.1:164328-165281;OQ419012.1:164302-165255;OQ419031.1:164306-165259;OQ419047.1:164308-165261;OQ451898.1:164198-165151;OQ451900.1:165112-166065;OQ581853.1:165120-166073;OQ581855.1:165219-166172;OQ721951.1:165138-166091;OQ721954.1:165111-166064;OR035652.1:165040-165993;OR035664.1:165044-165997;OR035702.1:165041-165994;OR146272.1:165060-166013;OR146330.1:165121-166074;OR264364.1:165115-166068;OR427332.1:165115-166068;OR459777.1:165104-166057;OR459797.1:165113-166066;OR943698.1:164740-165693;PP098589.2:165110-166063;PP496048.1:165114-166067;PP601206.1:164792-165745;PP776553.1:165034-165987;PP852971.1:165098-166051;PP852983.1:165119-166072;PP853000.1:165070-166023;PP853005.1:165078-166031;PP853007.1:165121-166074;PP853013.1:165093-166046;PP853027.1:165135-166088;PP853045.1:165086-166039;PP853048.1:165111-166064;PP860026.1:165106-166059;PP860027.1:165137-166090;PP860028.1:165069-166022;PP860031.1:165123-166076;PP860033.1:165120-166073;PQ153226.1:165091-166044;PQ178862.2:163464-164417;PQ221506.1:158627-159580;PQ221833.1:164767-165720;PQ340477.1:165078-166031;PQ340478.1:165060-166013;PQ340480.1:165138-166091;PQ340483.1:165124-166077;PQ340485.1:165107-166060;PQ340486.1:165125-166078;PQ340487.1:165149-166102;PQ340489.1:165124-166077;PQ340490.1:165076-166029;PQ340491.1:165131-166084;PQ340493.1:165115-166068;PQ340495.1:165141-166094;PQ340496.1:165176-166129;PQ368489.1:165124-166077;PQ451835.1:165181-166134;PQ451836.1:165119-166072;PQ451838.1:165169-166122;PQ451841.1:165132-166085;PQ451842.1:165078-166031 |
| OG0000040 | AY603973.1:168582-169130;AY741551.1:168616-169164;AY753185.1:168845-169393;DQ011154.1:166505-167053;DQ011155.1:166447-166995;EPI_ISL_13331598:166725-167273;EPI_ISL_13363142:166713-167261;EPI_ISL_13374487:166746-167294;EPI_ISL_13502582:166741-167289;EPI_ISL_13508471:166807-167355;EPI_ISL_13607904:166712-167260;EPI_ISL_13624509:166647-167195;EPI_ISL_13889442:166840-167388;EPI_ISL_13953610:166640-167188;EPI_ISL_14181948:166870-167418;EPI_ISL_14838587:166459-167007;EPI_ISL_15263355:166706-167254;EPI_ISL_15269699:166675-167223;EPI_ISL_15293002:166944-167492;EPI_ISL_15641561:166661-167209;EPI_ISL_15831211:166715-167263;EPI_ISL_15992098:166755-167303;EPI_ISL_16233784:166727-167275;EPI_ISL_16946400:166717-167265;EPI_ISL_17048206:166381-166929;EPI_ISL_17703567:166728-167276;EPI_ISL_17718458:166708-167256;EPI_ISL_18213374:166719-167267;EPI_ISL_18360394:166026-166574;EPI_ISL_18557816:166722-167270;EPI_ISL_18642365:166717-167265;EPI_ISL_18659846:166676-167224;EPI_ISL_18744049:166725-167273;EPI_ISL_19022858:166682-167230;EPI_ISL_19028778:166634-167182;EPI_ISL_19151800:166718-167266;EPI_ISL_19159116:166703-167251;EPI_ISL_19350788:165091-165639;EPI_ISL_19359677:166718-167266;EPI_ISL_19446306:166705-167253;EPI_ISL_19467692:166645-167193;JX878411.1:167347-167895;JX878425.1:166834-167382;JX878426.1:166407-166955;KC257460.1:166230-166778;KJ642612.1:164578-165126;KJ642613.1:166113-166661;KJ642616.1:168301-168849;KJ642618.1:164044-164592;KP849469.1:166663-167211;KP849471.1:166598-167146;LC831698.1:166701-167249;MN648051.1:166944-167492;MN702445.1:159981-160529;MN702446.1:166145-166693;MN702447.1:159515-160063;MN702449.1:160019-160567;MN702452.1:166203-166751;MT903338.1:166877-167425;MT903340.1:166730-167278;MT903341.1:158271-158819;MT903342.1:166767-167315;MT903347.1:168651-169199;ON568298.1:166871-167419;ON622713.1:166935-167483;ON622722.2:166723-167271;ON631963.1:166732-167280;ON649879.1:166538-167086;ON676707.1:166769-167317;ON755039.1:166360-166908;ON808413.1:166697-167245;ON880420.1:166933-167481;ON911481.2:166718-167266;ON918656.1:166740-167288;ON959133.1:166829-167377;ON959136.1:166830-167378;ON983168.1:165406-165954;OP019276.1:166717-167265;OP150924.1:166843-167391;OP160532.1:166666-167214;OP235282.1:166717-167265;OP289782.1:166721-167269;OP331335.1:166726-167274;OP382479.1:166710-167258;OP422341.1:166723-167271;OP434519.1:166699-167247;OP535324.1:166730-167278;OP535333.1:166837-167385;OP536770.1:166720-167268;OP597769.1:166754-167302;OP604566.1:166720-167268;OP612682.1:166655-167203;OP612684.1:166706-167254;OP612686.1:166708-167256;OP696841.1:163996-164544;OP879722.1:165943-166491;OQ419007.1:165937-166485;OQ419012.1:165911-166459;OQ419031.1:165915-166463;OQ419047.1:165917-166465;OQ451898.1:165807-166355;OQ451900.1:166721-167269;OQ581853.1:166729-167277;OQ581855.1:166828-167376;OQ721951.1:166747-167295;OQ721954.1:166720-167268;OR035652.1:166649-167197;OR035664.1:166653-167201;OR035702.1:166650-167198;OR146272.1:166669-167217;OR146330.1:166730-167278;OR264364.1:166724-167272;OR427332.1:166724-167272;OR459777.1:166713-167261;OR459797.1:166722-167270;OR943698.1:166353-166901;PP098589.2:166719-167267;PP496048.1:166723-167271;PP601206.1:166405-166953;PP776553.1:166643-167191;PP852971.1:166707-167255;PP852983.1:166728-167276;PP853000.1:166679-167227;PP853005.1:166687-167235;PP853007.1:166730-167278;PP853013.1:166702-167250;PP853027.1:166744-167292;PP853045.1:166695-167243;PP853048.1:166720-167268;PP860026.1:166727-167275;PP860027.1:166737-167285;PP860028.1:166676-167224;PP860031.1:166723-167271;PP860033.1:166720-167268;PQ153226.1:166700-167248;PQ178862.2:165070-165618;PQ221506.1:160240-160788;PQ221833.1:166387-166935;PQ340477.1:166687-167235;PQ340478.1:166669-167217;PQ340480.1:166747-167295;PQ340483.1:166733-167281;PQ340485.1:166716-167264;PQ340486.1:166734-167282;PQ340487.1:166758-167306;PQ340489.1:166733-167281;PQ340490.1:166685-167233;PQ340491.1:166740-167288;PQ340493.1:166724-167272;PQ340495.1:166750-167298;PQ340496.1:166785-167333;PQ368489.1:166733-167281;PQ451835.1:166790-167338;PQ451836.1:166728-167276;PQ451838.1:166778-167326;PQ451841.1:166741-167289;PQ451842.1:166687-167235 |
| OG0000041 | AY603973.1:169185-169988;AY741551.1:169219-170022;AY753185.1:169448-170251;DQ011154.1:167108-167911;DQ011155.1:167050-167853;EPI_ISL_13331598:167328-168131;EPI_ISL_13363142:167316-168119;EPI_ISL_13374487:167349-168152;EPI_ISL_13502582:167344-168147;EPI_ISL_13508471:167410-168213;EPI_ISL_13607904:167315-168118;EPI_ISL_13624509:167250-168053;EPI_ISL_13889442:167443-168246;EPI_ISL_13953610:167243-168046;EPI_ISL_14181948:167473-168276;EPI_ISL_14838587:167062-167865;EPI_ISL_15263355:167309-168112;EPI_ISL_15269699:167278-168081;EPI_ISL_15293002:167547-168350;EPI_ISL_15641561:167264-168067;EPI_ISL_15831211:167318-168121;EPI_ISL_15992098:167358-168161;EPI_ISL_16233784:167330-168133;EPI_ISL_16946400:167320-168123;EPI_ISL_17048206:166984-167787;EPI_ISL_17703567:167331-168134;EPI_ISL_17718458:167311-168114;EPI_ISL_18213374:167322-168125;EPI_ISL_18360394:166629-167432;EPI_ISL_18557816:167325-168128;EPI_ISL_18642365:167320-168123;EPI_ISL_18659846:167279-168082;EPI_ISL_18744049:167328-168131;EPI_ISL_19022858:167285-168088;EPI_ISL_19028778:167237-168040;EPI_ISL_19151800:167321-168124;EPI_ISL_19159116:167306-168109;EPI_ISL_19350788:165694-166497;EPI_ISL_19359677:167321-168124;EPI_ISL_19446306:167308-168111;EPI_ISL_19467692:167153-168052;JX878411.1:167950-168753;JX878425.1:167437-168240;JX878426.1:167010-167813;KC257460.1:166833-167636;KJ642612.1:165181-165984;KJ642613.1:166716-167519;KJ642616.1:168904-169707;KJ642618.1:164647-165450;KP849469.1:167266-168069;KP849471.1:167201-168004;LC831698.1:167304-168107;MN648051.1:167547-168350;MN702445.1:160584-161387;MN702446.1:166748-167551;MN702447.1:160118-160921;MN702449.1:160622-161425;MN702452.1:166806-167609;MT903338.1:167480-168283;MT903340.1:167333-168136;MT903341.1:158874-159677;MT903342.1:167370-168173;MT903347.1:169254-170057;ON568298.1:167474-168277;ON622713.1:167538-168341;ON622722.2:167326-168129;ON631963.1:167335-168138;ON649879.1:167141-167944;ON676707.1:167372-168175;ON755039.1:166963-167766;ON808413.1:167300-168103;ON880420.1:167536-168339;ON911481.2:167321-168124;ON918656.1:167343-168146;ON959133.1:167432-168235;ON959136.1:167433-168236;ON983168.1:166009-166812;OP019276.1:167320-168123;OP150924.1:167446-168249;OP160532.1:167269-168072;OP235282.1:167320-168123;OP289782.1:167324-168127;OP331335.1:167329-168132;OP382479.1:167313-168116;OP422341.1:167326-168129;OP434519.1:167302-168105;OP535324.1:167333-168136;OP535333.1:167440-168243;OP536770.1:167323-168126;OP597769.1:167357-168160;OP604566.1:167323-168126;OP612682.1:167258-168061;OP612684.1:167309-168112;OP612686.1:167311-168114;OP696841.1:164599-165402;OP879722.1:166546-167349;OQ419007.1:166540-167343;OQ419012.1:166514-167317;OQ419031.1:166518-167321;OQ419047.1:166520-167323;OQ451898.1:166410-167213;OQ451900.1:167324-168127;OQ581853.1:167332-168135;OQ581855.1:167431-168234;OQ721951.1:167350-168153;OQ721954.1:167323-168126;OR035652.1:167252-168055;OR035664.1:167256-168059;OR035702.1:167253-168056;OR146272.1:167272-168075;OR146330.1:167333-168136;OR264364.1:167327-168130;OR427332.1:167327-168130;OR459777.1:167316-168119;OR459797.1:167325-168128;OR943698.1:166956-167759;PP098589.2:167322-168125;PP496048.1:167326-168129;PP601206.1:166913-167812;PP776553.1:167246-168049;PP852971.1:167310-168113;PP852983.1:167331-168134;PP853000.1:167282-168085;PP853005.1:167290-168093;PP853007.1:167333-168136;PP853013.1:167305-168108;PP853027.1:167347-168150;PP853045.1:167298-168101;PP853048.1:167323-168126;PP860026.1:167330-168133;PP860027.1:167340-168143;PP860028.1:167279-168082;PP860031.1:167326-168129;PP860033.1:167323-168126;PQ153226.1:167303-168106;PQ178862.2:165673-166476;PQ221506.1:160843-161646;PQ221833.1:166990-167793;PQ340477.1:167290-168093;PQ340478.1:167272-168075;PQ340480.1:167350-168153;PQ340483.1:167336-168139;PQ340485.1:167319-168122;PQ340486.1:167337-168140;PQ340487.1:167361-168164;PQ340489.1:167336-168139;PQ340490.1:167288-168091;PQ340491.1:167343-168146;PQ340493.1:167327-168130;PQ340495.1:167353-168156;PQ340496.1:167388-168191;PQ368489.1:167336-168139;PQ451835.1:167393-168196;PQ451836.1:167331-168134;PQ451838.1:167381-168184;PQ451841.1:167344-168147;PQ451842.1:167290-168093 |
| OG0000042 | AY603973.1:174663-175154;AY741551.1:174672-175163;AY753185.1:174926-175417;DQ011154.1:172590-173570;DQ011155.1:172496-173128;EPI_ISL_13331598:172679-173221;EPI_ISL_13363142:172667-173209;EPI_ISL_13374487:172700-173242;EPI_ISL_13502582:172695-173237;EPI_ISL_13508471:172761-173303;EPI_ISL_13607904:172666-173208;EPI_ISL_13624509:172601-173143;EPI_ISL_13889442:172794-173336;EPI_ISL_13953610:172595-173137;EPI_ISL_14181948:172824-173366;EPI_ISL_14838587:172418-173398;EPI_ISL_15263355:172660-173202;EPI_ISL_15269699:172629-173171;EPI_ISL_15293002:172898-173440;EPI_ISL_15641561:172127-172669;EPI_ISL_15831211:172669-173211;EPI_ISL_15992098:172709-173251;EPI_ISL_16233784:172681-173223;EPI_ISL_16946400:172671-173213;EPI_ISL_17048206:172335-172877;EPI_ISL_17703567:172682-173224;EPI_ISL_17718458:172651-173193;EPI_ISL_18213374:172673-173215;EPI_ISL_18360394:171980-172522;EPI_ISL_18557816:172676-173218;EPI_ISL_18642365:172671-173213;EPI_ISL_18659846:172630-173172;EPI_ISL_18744049:172680-173222;EPI_ISL_19022858:172636-173178;EPI_ISL_19028778:172588-173130;EPI_ISL_19151800:171583-172125;EPI_ISL_19159116:172657-173199;EPI_ISL_19350788:171047-172039;EPI_ISL_19359677:172672-173214;EPI_ISL_19446306:172659-173201;EPI_ISL_19467692:172659-173639;JX878411.1:173431-174411;JX878425.1:172883-173863;JX878426.1:172354-173334;KC257460.1:172246-172869;KJ642612.1:170544-171524;KJ642613.1:172151-173131;KJ642616.1:174370-174861;KJ642618.1:170064-170690;KP849469.1:172778-173758;KP849471.1:172581-173561;LC831698.1:172655-173197;MN648051.1:172898-173440;MN702445.1:165958-166938;MN702446.1:172128-173108;MN702447.1:165474-166094;MN702449.1:165996-166976;MN702452.1:172180-173160;MT903338.1:172831-173373;MT903340.1:172684-173226;MT903341.1:164225-164767;MT903342.1:172721-173263;MT903347.1:174228-174719;ON568298.1:172825-173367;ON622713.1:172889-173431;ON622722.2:172677-173219;ON631963.1:172686-173228;ON649879.1:172492-173034;ON676707.1:172723-173265;ON755039.1:172314-172856;ON808413.1:172651-173193;ON880420.1:172887-173429;ON911481.2:172672-173214;ON918656.1:172694-173236;ON959133.1:172783-173325;ON959136.1:172784-173326;ON983168.1:171360-171902;OP019276.1:172671-173213;OP150924.1:172797-173339;OP160532.1:172620-173162;OP235282.1:172671-173213;OP289782.1:172675-173217;OP331335.1:172681-173223;OP382479.1:172664-173206;OP422341.1:172677-173219;OP434519.1:172653-173195;OP535324.1:172685-173227;OP535333.1:172791-173333;OP536770.1:172674-173216;OP597769.1:172708-173250;OP604566.1:172674-173216;OP612682.1:172609-173151;OP612684.1:172660-173202;OP612686.1:172662-173204;OP696841.1:169950-170492;OP879722.1:171897-172439;OQ419007.1:171891-172433;OQ419012.1:171865-172407;OQ419031.1:171869-172411;OQ419047.1:171871-172413;OQ451898.1:171761-172303;OQ451900.1:172675-173217;OQ581853.1:172683-173225;OQ581855.1:172782-173324;OQ721951.1:172701-173243;OQ721954.1:172674-173216;OR035652.1:172603-173145;OR035664.1:172607-173149;OR035702.1:172604-173146;OR146272.1:172623-173165;OR146330.1:172684-173226;OR264364.1:172678-173220;OR427332.1:172678-173220;OR459777.1:172656-173198;OR459797.1:172676-173218;OR943698.1:172189-173151;PP098589.2:172673-173215;PP496048.1:172677-173219;PP601206.1:172366-173346;PP776553.1:172597-173139;PP852971.1:172662-173204;PP852983.1:172682-173224;PP853000.1:172603-173145;PP853005.1:172641-173183;PP853007.1:172684-173226;PP853013.1:172657-173199;PP853027.1:172698-173240;PP853045.1:172638-173180;PP853048.1:172674-173216;PP860026.1:172671-173213;PP860027.1:172690-173232;PP860028.1:172620-173162;PP860031.1:172662-173204;PP860033.1:172673-173215;PQ153226.1:172654-173196;PQ178862.2:171017-172015;PQ221506.1:166218-167198;PQ221833.1:172346-173326;PQ340477.1:172641-173183;PQ340478.1:172623-173165;PQ340480.1:172701-173243;PQ340483.1:172687-173229;PQ340485.1:172670-173212;PQ340486.1:172688-173230;PQ340487.1:172712-173254;PQ340489.1:172687-173229;PQ340490.1:172639-173181;PQ340491.1:172694-173236;PQ340493.1:172678-173220;PQ340495.1:172704-173246;PQ340496.1:172739-173281;PQ368489.1:172687-173229;PQ451835.1:172744-173286;PQ451836.1:172682-173224;PQ451838.1:172732-173274;PQ451841.1:172695-173237;PQ451842.1:172641-173183 |
| OG0000043 | AY603973.1:22624-22869;AY741551.1:22486-22668;AY753185.1:22867-23112;DQ011154.1:23077-23328;DQ011155.1:23057-23308;EPI_ISL_13331598:20946-21191;EPI_ISL_13363142:20939-21184;EPI_ISL_13374487:20954-21199;EPI_ISL_13502582:20939-21184;EPI_ISL_13508471:21031-21276;EPI_ISL_13607904:20930-21175;EPI_ISL_13624509:20874-21119;EPI_ISL_13889442:21049-21294;EPI_ISL_13953610:20897-21142;EPI_ISL_14181948:21075-21320;EPI_ISL_14838587:22931-23182;EPI_ISL_15263355:20930-21175;EPI_ISL_15269699:20899-21144;EPI_ISL_15293002:21172-21417;EPI_ISL_15641561:20888-21133;EPI_ISL_15831211:20937-21182;EPI_ISL_15992098:20952-21197;EPI_ISL_16233784:20948-21193;EPI_ISL_16946400:20937-21182;EPI_ISL_17048206:20587-20832;EPI_ISL_17703567:20952-21197;EPI_ISL_17718458:20930-21175;EPI_ISL_18213374:20927-21172;EPI_ISL_18360394:20588-20833;EPI_ISL_18557816:20928-21173;EPI_ISL_18642365:20930-21175;EPI_ISL_18659846:20900-21145;EPI_ISL_18744049:20932-21177;EPI_ISL_19022858:20927-21172;EPI_ISL_19028778:20844-21089;EPI_ISL_19151800:20928-21173;EPI_ISL_19159116:20930-21175;EPI_ISL_19350788:21811-22056;EPI_ISL_19359677:20927-21172;EPI_ISL_19446306:20922-21167;EPI_ISL_19467692:23210-23461;JX878411.1:23925-24176;JX878425.1:23438-23617;JX878426.1:22880-23125;KC257460.1:22860-23111;KJ642612.1:21165-21416;KJ642613.1:22746-22997;KJ642616.1:22440-22685;KJ642618.1:20337-20585;KP849469.1:23247-23498;KP849471.1:23168-23419;LC831698.1:20928-21173;MN648051.1:21150-21395;MN702445.1:16593-16838;MN702446.1:22739-22984;MN702447.1:16099-16278;MN702449.1:16604-16849;MN702452.1:22776-23027;MT903338.1:21093-21338;MT903340.1:20930-21175;MT903341.1:12463-12708;MT903342.1:20945-21190;MT903347.1:22818-23000;ON568298.1:21076-21321;ON622713.1:21162-21407;ON622722.2:20933-21178;ON631963.1:20953-21198;ON649879.1:20763-21008;ON676707.1:20983-21228;ON755039.1:20593-20838;ON808413.1:20933-21178;ON880420.1:21160-21405;ON911481.2:20950-21195;ON918656.1:20965-21210;ON959133.1:21054-21299;ON959136.1:21055-21300;ON983168.1:19629-19874;OP019276.1:20927-21172;OP150924.1:21068-21313;OP160532.1:20893-21138;OP235282.1:20927-21172;OP289782.1:20930-21175;OP331335.1:20947-21192;OP382479.1:20934-21179;OP422341.1:20947-21192;OP434519.1:20926-21171;OP535324.1:20928-21173;OP535333.1:21037-21282;OP536770.1:20929-21174;OP597769.1:20951-21196;OP604566.1:20930-21175;OP612682.1:20856-21101;OP612684.1:20920-21165;OP612686.1:20920-21165;OP696841.1:18204-18449;OP879722.1:20153-20398;OQ419007.1:20163-20408;OQ419012.1:20155-20400;OQ419031.1:20160-20405;OQ419047.1:20163-20408;OQ451898.1:20015-20260;OQ451900.1:20928-21173;OQ581853.1:20954-21199;OQ581855.1:21035-21280;OQ721951.1:20968-21213;OQ721954.1:20925-21170;OR035652.1:20877-21122;OR035664.1:20877-21122;OR035702.1:20877-21122;OR146272.1:20893-21138;OR146330.1:20938-21183;OR264364.1:20948-21193;OR427332.1:20948-21193;OR459777.1:20932-21177;OR459797.1:20947-21192;OR943698.1:22914-23159;PP098589.2:20929-21174;PP496048.1:20933-21178;PP601206.1:22929-23180;PP776553.1:20878-21123;PP852971.1:20927-21172;PP852983.1:20932-21177;PP853000.1:20894-21139;PP853005.1:20902-21147;PP853007.1:20934-21179;PP853013.1:20908-21153;PP853027.1:20939-21184;PP853045.1:20920-21165;PP853048.1:20939-21184;PP860026.1:20934-21179;PP860027.1:20945-21190;PP860028.1:20901-21146;PP860031.1:20955-21200;PP860033.1:20944-21189;PQ153226.1:20927-21172;PQ178862.2:21788-22033;PQ221506.1:16750-16995;PQ221833.1:22921-23172;PQ340477.1:20910-21155;PQ340478.1:20895-21140;PQ340480.1:20974-21219;PQ340483.1:20958-21203;PQ340485.1:20944-21189;PQ340486.1:20959-21204;PQ340487.1:20984-21229;PQ340489.1:20959-21204;PQ340490.1:20910-21155;PQ340491.1:20968-21213;PQ340493.1:20944-21189;PQ340495.1:20974-21219;PQ340496.1:21007-21252;PQ368489.1:20930-21175;PQ451835.1:21018-21263;PQ451836.1:20950-21195;PQ451838.1:21001-21246;PQ451841.1:20961-21206;PQ451842.1:20913-21158 |
| OG0000044 | AY603973.1:27406-27621;AY741551.1:27420-27635;AY753185.1:27649-27864;DQ011154.1:27652-27870;DQ011155.1:27638-27856;EPI_ISL_13331598:25492-25707;EPI_ISL_13363142:25485-25700;EPI_ISL_13374487:25500-25715;EPI_ISL_13502582:25485-25700;EPI_ISL_13508471:25577-25792;EPI_ISL_13607904:25476-25691;EPI_ISL_13624509:25420-25635;EPI_ISL_13889442:25595-25810;EPI_ISL_13953610:25443-25658;EPI_ISL_14181948:25621-25836;EPI_ISL_14838587:27506-27724;EPI_ISL_15263355:25476-25691;EPI_ISL_15269699:25445-25660;EPI_ISL_15293002:25718-25933;EPI_ISL_15641561:25434-25649;EPI_ISL_15831211:25483-25698;EPI_ISL_15992098:25498-25713;EPI_ISL_16233784:25494-25709;EPI_ISL_16946400:25483-25698;EPI_ISL_17048206:25133-25348;EPI_ISL_17703567:25498-25713;EPI_ISL_17718458:25476-25691;EPI_ISL_18213374:25473-25688;EPI_ISL_18360394:25134-25349;EPI_ISL_18557816:25474-25689;EPI_ISL_18642365:25476-25691;EPI_ISL_18659846:25446-25661;EPI_ISL_18744049:25478-25693;EPI_ISL_19022858:25473-25688;EPI_ISL_19028778:25390-25605;EPI_ISL_19151800:25474-25689;EPI_ISL_19159116:25476-25691;EPI_ISL_19350788:26391-26609;EPI_ISL_19359677:25473-25688;EPI_ISL_19446306:25468-25683;EPI_ISL_19467692:27785-28003;JX878411.1:28500-28718;JX878425.1:28014-28232;JX878426.1:27454-27672;KC257460.1:27435-27653;KJ642612.1:25740-25958;KJ642613.1:27321-27539;KJ642616.1:27105-27320;KJ642618.1:24908-25126;KP849469.1:27822-28040;KP849471.1:27743-27961;LC831698.1:25474-25689;MN648051.1:25696-25911;MN702445.1:21167-21385;MN702446.1:27313-27531;MN702447.1:20678-20896;MN702449.1:21178-21396;MN702452.1:27351-27569;MT903338.1:25639-25854;MT903340.1:25476-25691;MT903341.1:17009-17224;MT903342.1:25491-25706;MT903347.1:27404-27619;ON568298.1:25622-25837;ON622713.1:25708-25923;ON622722.2:25479-25694;ON631963.1:25499-25714;ON649879.1:25309-25524;ON676707.1:25529-25744;ON755039.1:25139-25354;ON808413.1:25479-25694;ON880420.1:25706-25921;ON911481.2:25496-25711;ON918656.1:25511-25726;ON959133.1:25600-25815;ON959136.1:25601-25816;ON983168.1:24175-24390;OP019276.1:25473-25688;OP150924.1:25614-25829;OP160532.1:25439-25654;OP235282.1:25473-25688;OP289782.1:25476-25691;OP331335.1:25493-25708;OP382479.1:25480-25695;OP422341.1:25493-25708;OP434519.1:25472-25687;OP535324.1:25474-25689;OP535333.1:25583-25798;OP536770.1:25475-25690;OP597769.1:25497-25712;OP604566.1:25476-25691;OP612682.1:25402-25617;OP612684.1:25466-25681;OP612686.1:25466-25681;OP696841.1:22750-22965;OP879722.1:24699-24914;OQ419007.1:24709-24924;OQ419012.1:24701-24916;OQ419031.1:24680-24895;OQ419047.1:24709-24924;OQ451898.1:24561-24776;OQ451900.1:25474-25689;OQ581853.1:25500-25715;OQ581855.1:25581-25796;OQ721951.1:25514-25729;OQ721954.1:25471-25686;OR035652.1:25423-25638;OR035664.1:25423-25638;OR035702.1:25423-25638;OR146272.1:25439-25654;OR146330.1:25484-25699;OR264364.1:25494-25709;OR427332.1:25494-25709;OR459777.1:25478-25693;OR459797.1:25493-25708;OR943698.1:27482-27700;PP098589.2:25475-25690;PP496048.1:25479-25694;PP601206.1:27504-27722;PP776553.1:25400-25615;PP852971.1:25473-25688;PP852983.1:25478-25693;PP853000.1:25440-25655;PP853005.1:25448-25663;PP853007.1:25480-25695;PP853013.1:25454-25669;PP853027.1:25485-25700;PP853045.1:25466-25681;PP853048.1:25485-25700;PP860026.1:25480-25695;PP860027.1:25491-25706;PP860028.1:25447-25662;PP860031.1:25501-25716;PP860033.1:25490-25705;PQ153226.1:25473-25688;PQ178862.2:26368-26586;PQ221506.1:21324-21542;PQ221833.1:27496-27714;PQ340477.1:25456-25671;PQ340478.1:25441-25656;PQ340480.1:25520-25735;PQ340483.1:25504-25719;PQ340485.1:25490-25705;PQ340486.1:25505-25720;PQ340487.1:25530-25745;PQ340489.1:25505-25720;PQ340490.1:25456-25671;PQ340491.1:25514-25729;PQ340493.1:25490-25705;PQ340495.1:25520-25735;PQ340496.1:25553-25768;PQ368489.1:25476-25691;PQ451835.1:25564-25779;PQ451836.1:25496-25711;PQ451838.1:25547-25762;PQ451841.1:25507-25722;PQ451842.1:25459-25674 |
| OG0000045 | AY603973.1:43891-44196;AY741551.1:43895-44200;AY753185.1:44134-44439;DQ011154.1:44109-44414;DQ011155.1:44090-44395;EPI_ISL_13331598:41962-42267;EPI_ISL_13363142:41955-42260;EPI_ISL_13374487:41970-42275;EPI_ISL_13502582:41955-42260;EPI_ISL_13508471:42047-42352;EPI_ISL_13607904:41946-42251;EPI_ISL_13624509:41890-42195;EPI_ISL_13889442:42065-42370;EPI_ISL_13953610:41913-42218;EPI_ISL_14181948:42091-42396;EPI_ISL_14838587:43963-44268;EPI_ISL_15263355:41946-42251;EPI_ISL_15269699:41915-42220;EPI_ISL_15293002:42188-42493;EPI_ISL_15641561:41904-42209;EPI_ISL_15831211:41953-42258;EPI_ISL_15992098:41968-42273;EPI_ISL_16233784:41964-42269;EPI_ISL_16946400:41953-42258;EPI_ISL_17048206:41603-41908;EPI_ISL_17703567:41968-42273;EPI_ISL_17718458:41946-42251;EPI_ISL_18213374:41943-42248;EPI_ISL_18360394:41604-41909;EPI_ISL_18557816:41944-42249;EPI_ISL_18642365:41946-42251;EPI_ISL_18659846:41916-42221;EPI_ISL_18744049:41948-42253;EPI_ISL_19022858:41943-42248;EPI_ISL_19028778:41860-42165;EPI_ISL_19151800:41944-42249;EPI_ISL_19159116:41946-42251;EPI_ISL_19350788:42837-43142;EPI_ISL_19359677:41943-42248;EPI_ISL_19446306:41938-42243;EPI_ISL_19467692:44242-44547;JX878411.1:44957-45262;JX878425.1:44471-44776;JX878426.1:43916-44221;KC257460.1:43887-44192;KJ642612.1:42202-42507;KJ642613.1:43779-44084;KJ642616.1:43585-43890;KJ642618.1:41370-41675;KP849469.1:44279-44584;KP849471.1:44205-44510;LC831698.1:41944-42249;MN648051.1:42166-42471;MN702445.1:37624-37929;MN702446.1:43769-44074;MN702447.1:37135-37440;MN702449.1:37635-37940;MN702452.1:43808-44113;MT903338.1:42109-42414;MT903340.1:41946-42251;MT903341.1:33479-33784;MT903342.1:41961-42266;MT903347.1:43904-44209;ON568298.1:42092-42397;ON622713.1:42178-42483;ON622722.2:41949-42254;ON631963.1:41969-42274;ON649879.1:41779-42084;ON676707.1:41999-42304;ON755039.1:41609-41914;ON808413.1:41949-42254;ON880420.1:42176-42481;ON911481.2:41966-42271;ON918656.1:41981-42286;ON959133.1:42070-42375;ON959136.1:42071-42376;ON983168.1:40645-40950;OP019276.1:41943-42248;OP150924.1:42084-42389;OP160532.1:41909-42214;OP235282.1:41943-42248;OP289782.1:41946-42251;OP331335.1:41963-42268;OP382479.1:41950-42255;OP422341.1:41963-42268;OP434519.1:41942-42247;OP535324.1:41944-42249;OP535333.1:42053-42358;OP536770.1:41945-42250;OP597769.1:41967-42272;OP604566.1:41946-42251;OP612682.1:41872-42177;OP612684.1:41936-42241;OP612686.1:41936-42241;OP696841.1:39220-39525;OP879722.1:41169-41474;OQ419007.1:41179-41484;OQ419012.1:41168-41473;OQ419031.1:41150-41455;OQ419047.1:41179-41484;OQ451898.1:41031-41336;OQ451900.1:41944-42249;OQ581853.1:41970-42275;OQ581855.1:42051-42356;OQ721951.1:41984-42289;OQ721954.1:41941-42246;OR035652.1:41893-42198;OR035664.1:41893-42198;OR035702.1:41893-42198;OR146272.1:41909-42214;OR146330.1:41954-42259;OR264364.1:41964-42269;OR427332.1:41964-42269;OR459777.1:41948-42253;OR459797.1:41963-42268;OR943698.1:43944-44249;PP098589.2:41945-42250;PP496048.1:41949-42254;PP601206.1:43961-44266;PP776553.1:41870-42175;PP852971.1:41943-42248;PP852983.1:41949-42254;PP853000.1:41910-42215;PP853005.1:41918-42223;PP853007.1:41951-42256;PP853013.1:41924-42229;PP853027.1:41955-42260;PP853045.1:41936-42241;PP853048.1:41955-42260;PP860026.1:41953-42258;PP860027.1:41978-42283;PP860028.1:41919-42224;PP860031.1:41972-42277;PP860033.1:41977-42282;PQ153226.1:41943-42248;PQ178862.2:42814-43119;PQ221506.1:37784-38089;PQ221833.1:43953-44258;PQ340477.1:41926-42231;PQ340478.1:41911-42216;PQ340480.1:41990-42295;PQ340483.1:41974-42279;PQ340485.1:41960-42265;PQ340486.1:41975-42280;PQ340487.1:42000-42305;PQ340489.1:41975-42280;PQ340490.1:41926-42231;PQ340491.1:41981-42286;PQ340493.1:41960-42265;PQ340495.1:41990-42295;PQ340496.1:42023-42328;PQ368489.1:41946-42251;PQ451835.1:42031-42336;PQ451836.1:41963-42268;PQ451838.1:42014-42319;PQ451841.1:41974-42279;PQ451842.1:41926-42231 |
| OG0000046 | AY603973.1:52534-53355;AY741551.1:52538-53359;AY753185.1:52777-53598;DQ011154.1:52994-53815;DQ011155.1:52972-53793;EPI_ISL_13331598:50605-51426;EPI_ISL_13363142:50598-51419;EPI_ISL_13374487:50613-51434;EPI_ISL_13502582:50598-51419;EPI_ISL_13508471:50690-51511;EPI_ISL_13607904:50589-51410;EPI_ISL_13624509:50533-51354;EPI_ISL_13889442:50708-51529;EPI_ISL_13953610:50549-51370;EPI_ISL_14181948:50734-51555;EPI_ISL_14838587:52848-53669;EPI_ISL_15263355:50589-51410;EPI_ISL_15269699:50558-51379;EPI_ISL_15293002:50831-51652;EPI_ISL_15641561:50547-51368;EPI_ISL_15831211:50596-51417;EPI_ISL_15992098:50611-51432;EPI_ISL_16233784:50607-51428;EPI_ISL_16946400:50596-51417;EPI_ISL_17048206:50246-51067;EPI_ISL_17703567:50611-51432;EPI_ISL_17718458:50589-51410;EPI_ISL_18213374:50586-51407;EPI_ISL_18360394:50247-51068;EPI_ISL_18557816:50587-51408;EPI_ISL_18642365:50589-51410;EPI_ISL_18659846:50559-51380;EPI_ISL_18744049:50591-51412;EPI_ISL_19022858:50586-51407;EPI_ISL_19028778:50503-51324;EPI_ISL_19151800:50587-51408;EPI_ISL_19159116:50589-51410;EPI_ISL_19350788:51720-52541;EPI_ISL_19359677:50586-51407;EPI_ISL_19446306:50581-51402;EPI_ISL_19467692:53131-53952;JX878411.1:53839-54660;JX878425.1:53356-54177;JX878426.1:52805-53626;KC257460.1:52770-53591;KJ642612.1:51091-51912;KJ642613.1:52650-53471;KJ642616.1:52228-53049;KJ642618.1:50258-51079;KP849469.1:53164-53985;KP849471.1:53089-53910;LC831698.1:50587-51408;MN648051.1:50809-51630;MN702445.1:46507-47328;MN702446.1:52652-53473;MN702447.1:46020-46841;MN702449.1:46518-47339;MN702452.1:52693-53514;MT903338.1:50752-51573;MT903340.1:50589-51410;MT903341.1:42122-42943;MT903342.1:50604-51425;MT903347.1:52545-53366;ON568298.1:50735-51556;ON622713.1:50821-51642;ON622722.2:50592-51413;ON631963.1:50612-51433;ON649879.1:50422-51243;ON676707.1:50642-51463;ON755039.1:50252-51073;ON808413.1:50592-51413;ON880420.1:50819-51640;ON911481.2:50609-51430;ON918656.1:50624-51445;ON959133.1:50713-51534;ON959136.1:50714-51535;ON983168.1:49288-50109;OP019276.1:50586-51407;OP150924.1:50727-51548;OP160532.1:50552-51373;OP235282.1:50586-51407;OP289782.1:50589-51410;OP331335.1:50606-51427;OP382479.1:50593-51414;OP422341.1:50606-51427;OP434519.1:50585-51406;OP535324.1:50587-51408;OP535333.1:50696-51517;OP536770.1:50588-51409;OP597769.1:50610-51431;OP604566.1:50589-51410;OP612682.1:50515-51336;OP612684.1:50579-51400;OP612686.1:50579-51400;OP696841.1:47863-48684;OP879722.1:49812-50633;OQ419007.1:49822-50643;OQ419012.1:49789-50610;OQ419031.1:49793-50614;OQ419047.1:49822-50643;OQ451898.1:49674-50495;OQ451900.1:50587-51408;OQ581853.1:50613-51434;OQ581855.1:50694-51515;OQ721951.1:50627-51448;OQ721954.1:50584-51405;OR035652.1:50536-51357;OR035664.1:50536-51357;OR035702.1:50536-51357;OR146272.1:50552-51373;OR146330.1:50597-51418;OR264364.1:50607-51428;OR427332.1:50607-51428;OR459777.1:50591-51412;OR459797.1:50606-51427;OR943698.1:52834-53655;PP098589.2:50588-51409;PP496048.1:50592-51413;PP601206.1:52846-53667;PP776553.1:50513-51334;PP852971.1:50586-51407;PP852983.1:50592-51413;PP853000.1:50553-51374;PP853005.1:50561-51382;PP853007.1:50594-51415;PP853013.1:50567-51388;PP853027.1:50598-51419;PP853045.1:50579-51400;PP853048.1:50598-51419;PP860026.1:50596-51417;PP860027.1:50621-51442;PP860028.1:50562-51383;PP860031.1:50615-51436;PP860033.1:50620-51441;PQ153226.1:50586-51407;PQ178862.2:51697-52518;PQ221506.1:46673-47494;PQ221833.1:52839-53660;PQ340477.1:50569-51390;PQ340478.1:50554-51375;PQ340480.1:50633-51454;PQ340483.1:50617-51438;PQ340485.1:50603-51424;PQ340486.1:50618-51439;PQ340487.1:50643-51464;PQ340489.1:50618-51439;PQ340490.1:50569-51390;PQ340491.1:50624-51445;PQ340493.1:50603-51424;PQ340495.1:50633-51454;PQ340496.1:50666-51487;PQ368489.1:50589-51410;PQ451835.1:50674-51495;PQ451836.1:50606-51427;PQ451838.1:50657-51478;PQ451841.1:50617-51438;PQ451842.1:50569-51390 |
| OG0000047 | AY603973.1:66667-68697;AY741551.1:66671-68701;AY753185.1:66910-68940;DQ011154.1:67128-69158;DQ011155.1:67106-69136;EPI_ISL_13331598:64737-66767;EPI_ISL_13363142:64730-66760;EPI_ISL_13374487:64745-66775;EPI_ISL_13502582:64730-66760;EPI_ISL_13508471:64822-66852;EPI_ISL_13607904:64721-66751;EPI_ISL_13624509:64665-66695;EPI_ISL_13889442:64840-66870;EPI_ISL_13953610:64657-66687;EPI_ISL_14181948:64866-66896;EPI_ISL_14838587:66982-69012;EPI_ISL_15263355:64721-66751;EPI_ISL_15269699:64690-66720;EPI_ISL_15293002:64963-66993;EPI_ISL_15641561:64679-66709;EPI_ISL_15831211:64728-66758;EPI_ISL_15992098:64743-66773;EPI_ISL_16233784:64739-66769;EPI_ISL_16946400:64728-66758;EPI_ISL_17048206:64378-66408;EPI_ISL_17703567:64743-66773;EPI_ISL_17718458:64721-66751;EPI_ISL_18213374:64718-66748;EPI_ISL_18360394:64379-66409;EPI_ISL_18557816:64719-66749;EPI_ISL_18642365:64721-66751;EPI_ISL_18659846:64691-66721;EPI_ISL_18744049:64723-66753;EPI_ISL_19022858:64718-66748;EPI_ISL_19028778:64635-66665;EPI_ISL_19151800:64719-66749;EPI_ISL_19159116:64721-66751;EPI_ISL_19350788:65854-67884;EPI_ISL_19359677:64718-66748;EPI_ISL_19446306:64713-66743;EPI_ISL_19467692:67265-69295;JX878411.1:67973-70003;JX878425.1:67490-69520;JX878426.1:66939-68969;KC257460.1:66904-68934;KJ642612.1:65225-67255;KJ642613.1:66784-68814;KJ642616.1:66361-68391;KJ642618.1:64392-66422;KP849469.1:67298-69328;KP849471.1:67223-69253;LC831698.1:64719-66749;MN648051.1:64941-66971;MN702445.1:60641-62671;MN702446.1:66786-68816;MN702447.1:60154-62184;MN702449.1:60652-62682;MN702452.1:66827-68857;MT903338.1:64884-66914;MT903340.1:64721-66751;MT903341.1:56254-58284;MT903342.1:64736-66766;MT903347.1:66678-68708;ON568298.1:64867-66897;ON622713.1:64953-66983;ON622722.2:64724-66754;ON631963.1:64744-66774;ON649879.1:64554-66584;ON676707.1:64774-66804;ON755039.1:64384-66414;ON808413.1:64724-66754;ON880420.1:64951-66981;ON911481.2:64741-66771;ON918656.1:64756-66786;ON959133.1:64845-66875;ON959136.1:64846-66876;ON983168.1:63420-65450;OP019276.1:64718-66748;OP150924.1:64859-66889;OP160532.1:64684-66714;OP235282.1:64718-66748;OP289782.1:64721-66751;OP331335.1:64738-66768;OP382479.1:64725-66755;OP422341.1:64738-66768;OP434519.1:64717-66747;OP535324.1:64719-66749;OP535333.1:64828-66858;OP536770.1:64720-66750;OP597769.1:64742-66772;OP604566.1:64721-66751;OP612682.1:64647-66677;OP612684.1:64711-66741;OP612686.1:64711-66741;OP696841.1:61995-64025;OP879722.1:63944-65974;OQ419007.1:63954-65984;OQ419012.1:63921-65951;OQ419031.1:63925-65955;OQ419047.1:63927-65957;OQ451898.1:63806-65836;OQ451900.1:64719-66749;OQ581853.1:64745-66775;OQ581855.1:64826-66856;OQ721951.1:64759-66789;OQ721954.1:64716-66746;OR035652.1:64668-66698;OR035664.1:64668-66698;OR035702.1:64668-66698;OR146272.1:64684-66714;OR146330.1:64729-66759;OR264364.1:64739-66769;OR427332.1:64739-66769;OR459777.1:64723-66753;OR459797.1:64738-66768;OR943698.1:66968-68998;PP098589.2:64720-66750;PP496048.1:64724-66754;PP601206.1:66980-69010;PP776553.1:64645-66675;PP852971.1:64718-66748;PP852983.1:64724-66754;PP853000.1:64685-66715;PP853005.1:64693-66723;PP853007.1:64726-66756;PP853013.1:64699-66729;PP853027.1:64730-66760;PP853045.1:64711-66741;PP853048.1:64730-66760;PP860026.1:64728-66758;PP860027.1:64753-66783;PP860028.1:64693-66723;PP860031.1:64747-66777;PP860033.1:64752-66782;PQ153226.1:64718-66748;PQ178862.2:65831-67861;PQ221506.1:60807-62837;PQ221833.1:66973-69003;PQ340477.1:64701-66731;PQ340478.1:64686-66716;PQ340480.1:64765-66795;PQ340483.1:64749-66779;PQ340485.1:64735-66765;PQ340486.1:64750-66780;PQ340487.1:64775-66805;PQ340489.1:64750-66780;PQ340490.1:64701-66731;PQ340491.1:64756-66786;PQ340493.1:64735-66765;PQ340495.1:64765-66795;PQ340496.1:64798-66828;PQ368489.1:64721-66751;PQ451835.1:64806-66836;PQ451836.1:64738-66768;PQ451838.1:64789-66819;PQ451841.1:64749-66779;PQ451842.1:64701-66731 |
| OG0000048 | AY603973.1:73315-73812;AY741551.1:73319-73816;AY753185.1:73558-74055;DQ011154.1:73776-74273;DQ011155.1:73754-74251;EPI_ISL_13331598:71385-71882;EPI_ISL_13363142:71378-71875;EPI_ISL_13374487:71393-71890;EPI_ISL_13502582:71378-71875;EPI_ISL_13508471:71470-71967;EPI_ISL_13607904:71369-71866;EPI_ISL_13624509:71313-71810;EPI_ISL_13889442:71488-71985;EPI_ISL_13953610:71305-71802;EPI_ISL_14181948:71514-72011;EPI_ISL_14838587:73630-74127;EPI_ISL_15263355:71369-71866;EPI_ISL_15269699:71338-71835;EPI_ISL_15293002:71611-72108;EPI_ISL_15641561:71327-71824;EPI_ISL_15831211:71376-71873;EPI_ISL_15992098:71391-71888;EPI_ISL_16233784:71387-71884;EPI_ISL_16946400:71376-71873;EPI_ISL_17048206:71026-71523;EPI_ISL_17703567:71391-71888;EPI_ISL_17718458:71369-71866;EPI_ISL_18213374:71366-71863;EPI_ISL_18360394:71027-71524;EPI_ISL_18557816:71367-71864;EPI_ISL_18642365:71369-71866;EPI_ISL_18659846:71339-71836;EPI_ISL_18744049:71371-71868;EPI_ISL_19022858:71366-71863;EPI_ISL_19028778:71283-71780;EPI_ISL_19151800:71367-71864;EPI_ISL_19159116:71369-71866;EPI_ISL_19350788:72502-72999;EPI_ISL_19359677:71366-71863;EPI_ISL_19446306:71361-71858;EPI_ISL_19467692:73913-74410;JX878411.1:74621-75118;JX878425.1:74138-74635;JX878426.1:73587-74084;KC257460.1:73552-74049;KJ642612.1:71873-72370;KJ642613.1:73432-73929;KJ642616.1:73009-73506;KJ642618.1:71040-71537;KP849469.1:73946-74443;KP849471.1:73872-74369;LC831698.1:71367-71864;MN648051.1:71589-72086;MN702445.1:67289-67786;MN702446.1:73434-73931;MN702447.1:66802-67299;MN702449.1:67300-67797;MN702452.1:73475-73972;MT903338.1:71532-72029;MT903340.1:71369-71866;MT903341.1:62902-63399;MT903342.1:71384-71881;MT903347.1:73326-73823;ON568298.1:71515-72012;ON622713.1:71601-72098;ON622722.2:71372-71869;ON631963.1:71392-71889;ON649879.1:71202-71699;ON676707.1:71422-71919;ON755039.1:71032-71529;ON808413.1:71372-71869;ON880420.1:71599-72096;ON911481.2:71389-71886;ON918656.1:71404-71901;ON959133.1:71493-71990;ON959136.1:71494-71991;ON983168.1:70068-70565;OP019276.1:71366-71863;OP150924.1:71507-72004;OP160532.1:71332-71829;OP235282.1:71366-71863;OP289782.1:71369-71866;OP331335.1:71386-71883;OP382479.1:71373-71870;OP422341.1:71386-71883;OP434519.1:71365-71862;OP535324.1:71367-71864;OP535333.1:71476-71973;OP536770.1:71368-71865;OP597769.1:71390-71887;OP604566.1:71369-71866;OP612682.1:71295-71792;OP612684.1:71359-71856;OP612686.1:71359-71856;OP696841.1:68643-69140;OP879722.1:70592-71089;OQ419007.1:70602-71099;OQ419012.1:70569-71066;OQ419031.1:70573-71070;OQ419047.1:70575-71072;OQ451898.1:70454-70951;OQ451900.1:71367-71864;OQ581853.1:71393-71890;OQ581855.1:71474-71971;OQ721951.1:71407-71904;OQ721954.1:71364-71861;OR035652.1:71316-71813;OR035664.1:71316-71813;OR035702.1:71316-71813;OR146272.1:71332-71829;OR146330.1:71377-71874;OR264364.1:71387-71884;OR427332.1:71387-71884;OR459777.1:71371-71868;OR459797.1:71386-71883;OR943698.1:73616-74113;PP098589.2:71368-71865;PP496048.1:71372-71869;PP601206.1:73628-74125;PP776553.1:71293-71790;PP852971.1:71366-71863;PP852983.1:71372-71869;PP853000.1:71333-71830;PP853005.1:71341-71838;PP853007.1:71374-71871;PP853013.1:71347-71844;PP853027.1:71378-71875;PP853045.1:71359-71856;PP853048.1:71378-71875;PP860026.1:71376-71873;PP860027.1:71401-71898;PP860028.1:71341-71838;PP860031.1:71395-71892;PP860033.1:71400-71897;PQ153226.1:71366-71863;PQ178862.2:72479-72976;PQ221506.1:67455-67952;PQ221833.1:73621-74118;PQ340477.1:71349-71846;PQ340478.1:71334-71831;PQ340480.1:71413-71910;PQ340483.1:71397-71894;PQ340485.1:71383-71880;PQ340486.1:71398-71895;PQ340487.1:71423-71920;PQ340489.1:71398-71895;PQ340490.1:71349-71846;PQ340491.1:71404-71901;PQ340493.1:71383-71880;PQ340495.1:71413-71910;PQ340496.1:71446-71943;PQ368489.1:71369-71866;PQ451835.1:71454-71951;PQ451836.1:71386-71883;PQ451838.1:71437-71934;PQ451841.1:71397-71894;PQ451842.1:71349-71846 |
| OG0000049 | AY603973.1:74923-75705;AY741551.1:74927-75709;AY753185.1:75166-75948;DQ011154.1:75384-76166;DQ011155.1:75365-76147;EPI_ISL_13331598:72993-73775;EPI_ISL_13363142:72986-73768;EPI_ISL_13374487:73001-73783;EPI_ISL_13502582:72986-73768;EPI_ISL_13508471:73078-73860;EPI_ISL_13607904:72977-73759;EPI_ISL_13624509:72921-73703;EPI_ISL_13889442:73096-73878;EPI_ISL_13953610:72913-73695;EPI_ISL_14181948:73122-73904;EPI_ISL_14838587:75238-76020;EPI_ISL_15263355:72977-73759;EPI_ISL_15269699:72946-73728;EPI_ISL_15293002:73219-74001;EPI_ISL_15641561:72935-73717;EPI_ISL_15831211:72984-73766;EPI_ISL_15992098:72999-73781;EPI_ISL_16233784:72995-73777;EPI_ISL_16946400:72984-73766;EPI_ISL_17048206:72634-73416;EPI_ISL_17703567:72999-73781;EPI_ISL_17718458:72977-73759;EPI_ISL_18213374:72974-73756;EPI_ISL_18360394:72635-73417;EPI_ISL_18557816:72975-73757;EPI_ISL_18642365:72977-73759;EPI_ISL_18659846:72947-73729;EPI_ISL_18744049:72979-73761;EPI_ISL_19022858:72974-73756;EPI_ISL_19028778:72891-73673;EPI_ISL_19151800:72975-73757;EPI_ISL_19159116:72977-73759;EPI_ISL_19350788:74110-74892;EPI_ISL_19359677:72974-73756;EPI_ISL_19446306:72969-73751;EPI_ISL_19467692:75521-76303;JX878411.1:76229-77011;JX878425.1:75746-76528;JX878426.1:75195-75977;KC257460.1:75163-75945;KJ642612.1:73481-74263;KJ642613.1:75040-75822;KJ642616.1:74617-75399;KJ642618.1:72648-73430;KP849469.1:75554-76336;KP849471.1:75480-76262;LC831698.1:72975-73757;MN648051.1:73197-73979;MN702445.1:68897-69679;MN702446.1:75042-75824;MN702447.1:68410-69192;MN702449.1:68908-69690;MN702452.1:75083-75865;MT903338.1:73140-73922;MT903340.1:72977-73759;MT903341.1:64510-65292;MT903342.1:72992-73774;MT903347.1:74934-75716;ON568298.1:73123-73905;ON622713.1:73209-73991;ON622722.2:72980-73762;ON631963.1:73000-73782;ON649879.1:72810-73592;ON676707.1:73030-73812;ON755039.1:72640-73422;ON808413.1:72980-73762;ON880420.1:73207-73989;ON911481.2:72997-73779;ON918656.1:73012-73794;ON959133.1:73101-73883;ON959136.1:73102-73884;ON983168.1:71676-72458;OP019276.1:72974-73756;OP150924.1:73115-73897;OP160532.1:72940-73722;OP235282.1:72974-73756;OP289782.1:72977-73759;OP331335.1:72994-73776;OP382479.1:72981-73763;OP422341.1:72994-73776;OP434519.1:72973-73755;OP535324.1:72975-73757;OP535333.1:73084-73866;OP536770.1:72976-73758;OP597769.1:72998-73780;OP604566.1:72977-73759;OP612682.1:72903-73685;OP612684.1:72967-73749;OP612686.1:72967-73749;OP696841.1:70251-71033;OP879722.1:72200-72982;OQ419007.1:72210-72992;OQ419012.1:72177-72959;OQ419031.1:72181-72963;OQ419047.1:72183-72965;OQ451898.1:72062-72844;OQ451900.1:72975-73757;OQ581853.1:73001-73783;OQ581855.1:73082-73864;OQ721951.1:73015-73797;OQ721954.1:72972-73754;OR035652.1:72924-73706;OR035664.1:72924-73706;OR035702.1:72924-73706;OR146272.1:72940-73722;OR146330.1:72985-73767;OR264364.1:72995-73777;OR427332.1:72995-73777;OR459777.1:72979-73761;OR459797.1:72994-73776;OR943698.1:75224-76006;PP098589.2:72976-73758;PP496048.1:72980-73762;PP601206.1:75236-76018;PP776553.1:72901-73683;PP852971.1:72974-73756;PP852983.1:72980-73762;PP853000.1:72941-73723;PP853005.1:72949-73731;PP853007.1:72982-73764;PP853013.1:72955-73737;PP853027.1:72986-73768;PP853045.1:72967-73749;PP853048.1:72986-73768;PP860026.1:72984-73766;PP860027.1:73009-73791;PP860028.1:72949-73731;PP860031.1:73003-73785;PP860033.1:73008-73790;PQ153226.1:72974-73756;PQ178862.2:74087-74869;PQ221506.1:69063-69845;PQ221833.1:75229-76011;PQ340477.1:72957-73739;PQ340478.1:72942-73724;PQ340480.1:73021-73803;PQ340483.1:73005-73787;PQ340485.1:72991-73773;PQ340486.1:73006-73788;PQ340487.1:73031-73813;PQ340489.1:73006-73788;PQ340490.1:72957-73739;PQ340491.1:73012-73794;PQ340493.1:72991-73773;PQ340495.1:73021-73803;PQ340496.1:73054-73836;PQ368489.1:72977-73759;PQ451835.1:73062-73844;PQ451836.1:72994-73776;PQ451838.1:73045-73827;PQ451841.1:73005-73787;PQ451842.1:72957-73739 |
| OG0000050 | AY603973.1:80431-80964;AY741551.1:80435-80968;AY753185.1:80674-81207;DQ011154.1:80892-81425;DQ011155.1:80873-81406;EPI_ISL_13331598:78501-79034;EPI_ISL_13363142:78494-79027;EPI_ISL_13374487:78509-79042;EPI_ISL_13502582:78494-79027;EPI_ISL_13508471:78586-79119;EPI_ISL_13607904:78485-79018;EPI_ISL_13624509:78429-78962;EPI_ISL_13889442:78604-79137;EPI_ISL_13953610:78421-78954;EPI_ISL_14181948:78630-79163;EPI_ISL_14838587:80746-81279;EPI_ISL_15263355:78485-79018;EPI_ISL_15269699:78454-78987;EPI_ISL_15293002:78727-79260;EPI_ISL_15641561:78443-78976;EPI_ISL_15831211:78492-79025;EPI_ISL_15992098:78507-79040;EPI_ISL_16233784:78503-79036;EPI_ISL_16946400:78492-79025;EPI_ISL_17048206:78142-78675;EPI_ISL_17703567:78507-79040;EPI_ISL_17718458:78485-79018;EPI_ISL_18213374:78482-79015;EPI_ISL_18360394:78143-78676;EPI_ISL_18557816:78483-79016;EPI_ISL_18642365:78485-79018;EPI_ISL_18659846:78455-78988;EPI_ISL_18744049:78487-79020;EPI_ISL_19022858:78482-79015;EPI_ISL_19028778:78399-78932;EPI_ISL_19151800:78483-79016;EPI_ISL_19159116:78485-79018;EPI_ISL_19350788:79618-80151;EPI_ISL_19359677:78482-79015;EPI_ISL_19446306:78477-79010;EPI_ISL_19467692:81029-81562;JX878411.1:81737-82270;JX878425.1:81254-81787;JX878426.1:80703-81236;KC257460.1:80671-81204;KJ642612.1:78989-79522;KJ642613.1:80548-81081;KJ642616.1:80125-80658;KJ642618.1:78219-78752;KP849469.1:81062-81595;KP849471.1:80988-81521;LC831698.1:78483-79016;MN648051.1:78705-79238;MN702445.1:74405-74938;MN702446.1:80550-81083;MN702447.1:73918-74451;MN702449.1:74416-74949;MN702452.1:80591-81124;MT903338.1:78648-79181;MT903340.1:78485-79018;MT903341.1:70018-70551;MT903342.1:78500-79033;MT903347.1:80442-80975;ON568298.1:78631-79164;ON622713.1:78717-79250;ON622722.2:78488-79021;ON631963.1:78508-79041;ON649879.1:78318-78851;ON676707.1:78538-79071;ON755039.1:78148-78681;ON808413.1:78488-79021;ON880420.1:78715-79248;ON911481.2:78505-79038;ON918656.1:78520-79053;ON959133.1:78609-79142;ON959136.1:78610-79143;ON983168.1:77184-77717;OP019276.1:78482-79015;OP150924.1:78623-79156;OP160532.1:78448-78981;OP235282.1:78482-79015;OP289782.1:78485-79018;OP331335.1:78502-79035;OP382479.1:78489-79022;OP422341.1:78502-79035;OP434519.1:78481-79014;OP535324.1:78483-79016;OP535333.1:78592-79125;OP536770.1:78484-79017;OP597769.1:78506-79039;OP604566.1:78485-79018;OP612682.1:78410-78943;OP612684.1:78474-79007;OP612686.1:78474-79007;OP696841.1:75759-76292;OP879722.1:77708-78241;OQ419007.1:77718-78251;OQ419012.1:77685-78218;OQ419031.1:77689-78222;OQ419047.1:77691-78224;OQ451898.1:77570-78103;OQ451900.1:78483-79016;OQ581853.1:78509-79042;OQ581855.1:78590-79123;OQ721951.1:78523-79056;OQ721954.1:78480-79013;OR035652.1:78432-78965;OR035664.1:78432-78965;OR035702.1:78432-78965;OR146272.1:78448-78981;OR146330.1:78493-79026;OR264364.1:78503-79036;OR427332.1:78503-79036;OR459777.1:78487-79020;OR459797.1:78502-79035;OR943698.1:80795-81328;PP098589.2:78484-79017;PP496048.1:78488-79021;PP601206.1:80744-81277;PP776553.1:78409-78942;PP852971.1:78482-79015;PP852983.1:78488-79021;PP853000.1:78449-78982;PP853005.1:78457-78990;PP853007.1:78490-79023;PP853013.1:78463-78996;PP853027.1:78494-79027;PP853045.1:78475-79008;PP853048.1:78494-79027;PP860026.1:78492-79025;PP860027.1:78517-79050;PP860028.1:78457-78990;PP860031.1:78511-79044;PP860033.1:78516-79049;PQ153226.1:78482-79015;PQ178862.2:79595-80128;PQ221506.1:74571-75104;PQ221833.1:80737-81270;PQ340477.1:78465-78998;PQ340478.1:78450-78983;PQ340480.1:78529-79062;PQ340483.1:78513-79046;PQ340485.1:78499-79032;PQ340486.1:78514-79047;PQ340487.1:78539-79072;PQ340489.1:78514-79047;PQ340490.1:78465-78998;PQ340491.1:78520-79053;PQ340493.1:78499-79032;PQ340495.1:78529-79062;PQ340496.1:78562-79095;PQ368489.1:78485-79018;PQ451835.1:78570-79103;PQ451836.1:78502-79035;PQ451838.1:78553-79086;PQ451841.1:78513-79046;PQ451842.1:78465-78998 |
| OG0000051 | AY603973.1:81946-82503;AY741551.1:81950-82507;AY753185.1:82189-82746;DQ011154.1:82407-82964;DQ011155.1:82388-82945;EPI_ISL_13331598:80016-80573;EPI_ISL_13363142:80009-80566;EPI_ISL_13374487:80024-80581;EPI_ISL_13502582:80009-80566;EPI_ISL_13508471:80101-80658;EPI_ISL_13607904:80000-80557;EPI_ISL_13624509:79944-80501;EPI_ISL_13889442:80119-80676;EPI_ISL_13953610:79936-80493;EPI_ISL_14181948:80145-80702;EPI_ISL_14838587:82261-82818;EPI_ISL_15263355:80000-80557;EPI_ISL_15269699:79969-80526;EPI_ISL_15293002:80242-80799;EPI_ISL_15641561:79958-80515;EPI_ISL_15831211:80007-80564;EPI_ISL_15992098:80022-80579;EPI_ISL_16233784:80018-80575;EPI_ISL_16946400:80007-80564;EPI_ISL_17048206:79657-80214;EPI_ISL_17703567:80022-80579;EPI_ISL_17718458:80000-80557;EPI_ISL_18213374:79997-80554;EPI_ISL_18360394:79658-80215;EPI_ISL_18557816:79998-80555;EPI_ISL_18642365:80000-80557;EPI_ISL_18659846:79970-80527;EPI_ISL_18744049:80002-80559;EPI_ISL_19022858:79997-80554;EPI_ISL_19028778:79914-80471;EPI_ISL_19151800:79998-80555;EPI_ISL_19159116:80000-80557;EPI_ISL_19350788:81133-81690;EPI_ISL_19359677:79997-80554;EPI_ISL_19446306:79992-80549;EPI_ISL_19467692:82544-83101;JX878411.1:83252-83809;JX878425.1:82769-83326;JX878426.1:82218-82775;KC257460.1:82186-82743;KJ642612.1:80504-81061;KJ642613.1:82063-82620;KJ642616.1:81640-82197;KJ642618.1:79734-80291;KP849469.1:82577-83134;KP849471.1:82503-83060;LC831698.1:79998-80555;MN648051.1:80220-80777;MN702445.1:75920-76477;MN702446.1:82065-82622;MN702447.1:75433-75990;MN702449.1:75931-76488;MN702452.1:82106-82663;MT903338.1:80163-80720;MT903340.1:80000-80557;MT903341.1:71533-72090;MT903342.1:80015-80572;MT903347.1:81957-82514;ON568298.1:80146-80703;ON622713.1:80232-80789;ON622722.2:80003-80560;ON631963.1:80023-80580;ON649879.1:79833-80390;ON676707.1:80053-80610;ON755039.1:79663-80220;ON808413.1:80003-80560;ON880420.1:80230-80787;ON911481.2:80020-80577;ON918656.1:80035-80592;ON959133.1:80124-80681;ON959136.1:80125-80682;ON983168.1:78699-79256;OP019276.1:79997-80554;OP150924.1:80138-80695;OP160532.1:79963-80520;OP235282.1:79997-80554;OP289782.1:80000-80557;OP331335.1:80017-80574;OP382479.1:80004-80561;OP422341.1:80017-80574;OP434519.1:79996-80553;OP535324.1:79998-80555;OP535333.1:80107-80664;OP536770.1:79999-80556;OP597769.1:80021-80578;OP604566.1:80000-80557;OP612682.1:79925-80482;OP612684.1:79989-80546;OP612686.1:79989-80546;OP696841.1:77274-77831;OP879722.1:79223-79780;OQ419007.1:79233-79790;OQ419012.1:79200-79757;OQ419031.1:79204-79761;OQ419047.1:79206-79763;OQ451898.1:79085-79642;OQ451900.1:79998-80555;OQ581853.1:80024-80581;OQ581855.1:80105-80662;OQ721951.1:80038-80595;OQ721954.1:79995-80552;OR035652.1:79947-80504;OR035664.1:79947-80504;OR035702.1:79947-80504;OR146272.1:79963-80520;OR146330.1:80008-80565;OR264364.1:80018-80575;OR427332.1:80018-80575;OR459777.1:80002-80559;OR459797.1:80017-80574;OR943698.1:82310-82867;PP098589.2:79999-80556;PP496048.1:80003-80560;PP601206.1:82259-82816;PP776553.1:79924-80481;PP852971.1:79997-80554;PP852983.1:80003-80560;PP853000.1:79964-80521;PP853005.1:79972-80529;PP853007.1:80005-80562;PP853013.1:79978-80535;PP853027.1:80009-80566;PP853045.1:79990-80547;PP853048.1:80009-80566;PP860026.1:80007-80564;PP860027.1:80032-80589;PP860028.1:79972-80529;PP860031.1:80026-80583;PP860033.1:80031-80588;PQ153226.1:79997-80554;PQ178862.2:81110-81667;PQ221506.1:76086-76643;PQ221833.1:82252-82809;PQ340477.1:79980-80537;PQ340478.1:79965-80522;PQ340480.1:80044-80601;PQ340483.1:80028-80585;PQ340485.1:80014-80571;PQ340486.1:80029-80586;PQ340487.1:80054-80611;PQ340489.1:80029-80586;PQ340490.1:79980-80537;PQ340491.1:80035-80592;PQ340493.1:80014-80571;PQ340495.1:80044-80601;PQ340496.1:80077-80634;PQ368489.1:80000-80557;PQ451835.1:80085-80642;PQ451836.1:80017-80574;PQ451838.1:80068-80625;PQ451841.1:80028-80585;PQ451842.1:79980-80537 |
| OG0000052 | AY603973.1:91579-92211;AY741551.1:91583-92215;AY753185.1:91822-92454;DQ011154.1:92038-92679;DQ011155.1:92019-92660;EPI_ISL_13331598:89647-90279;EPI_ISL_13363142:89640-90272;EPI_ISL_13374487:89655-90287;EPI_ISL_13502582:89640-90272;EPI_ISL_13508471:89732-90364;EPI_ISL_13607904:89631-90263;EPI_ISL_13624509:89575-90207;EPI_ISL_13889442:89750-90382;EPI_ISL_13953610:89548-90180;EPI_ISL_14181948:89776-90408;EPI_ISL_14838587:91892-92533;EPI_ISL_15263355:89631-90263;EPI_ISL_15269699:89600-90232;EPI_ISL_15293002:89873-90505;EPI_ISL_15641561:89589-90221;EPI_ISL_15831211:89638-90270;EPI_ISL_15992098:89653-90285;EPI_ISL_16233784:89649-90281;EPI_ISL_16946400:89638-90270;EPI_ISL_17048206:89288-89920;EPI_ISL_17703567:89653-90285;EPI_ISL_17718458:89631-90263;EPI_ISL_18213374:89628-90260;EPI_ISL_18360394:89289-89921;EPI_ISL_18557816:89629-90261;EPI_ISL_18642365:89631-90263;EPI_ISL_18659846:89601-90233;EPI_ISL_18744049:89633-90265;EPI_ISL_19022858:89628-90260;EPI_ISL_19028778:89545-90177;EPI_ISL_19151800:89629-90261;EPI_ISL_19159116:89631-90263;EPI_ISL_19350788:90764-91405;EPI_ISL_19359677:89628-90260;EPI_ISL_19446306:89623-90255;EPI_ISL_19467692:92175-92816;JX878411.1:92883-93524;JX878425.1:92400-93041;JX878426.1:91849-92490;KC257460.1:91817-92458;KJ642612.1:90135-90776;KJ642613.1:91694-92335;KJ642616.1:91273-91905;KJ642618.1:89365-90006;KP849469.1:92208-92849;KP849471.1:92134-92775;LC831698.1:89629-90261;MN648051.1:89851-90483;MN702445.1:85551-86192;MN702446.1:91696-92337;MN702447.1:85064-85705;MN702449.1:85562-86203;MN702452.1:91737-92378;MT903338.1:89794-90426;MT903340.1:89631-90263;MT903341.1:81164-81796;MT903342.1:89646-90278;MT903347.1:91590-92222;ON568298.1:89777-90409;ON622713.1:89863-90495;ON622722.2:89634-90266;ON631963.1:89654-90286;ON649879.1:89464-90096;ON676707.1:89684-90316;ON755039.1:89294-89926;ON808413.1:89634-90266;ON880420.1:89861-90493;ON911481.2:89651-90283;ON918656.1:89666-90298;ON959133.1:89755-90387;ON959136.1:89756-90388;ON983168.1:88329-88961;OP019276.1:89628-90260;OP150924.1:89769-90401;OP160532.1:89594-90226;OP235282.1:89628-90260;OP289782.1:89631-90263;OP331335.1:89648-90280;OP382479.1:89635-90267;OP422341.1:89648-90280;OP434519.1:89627-90259;OP535324.1:89629-90261;OP535333.1:89738-90370;OP536770.1:89630-90262;OP597769.1:89652-90284;OP604566.1:89631-90263;OP612682.1:89556-90188;OP612684.1:89619-90251;OP612686.1:89620-90252;OP696841.1:86905-87537;OP879722.1:88854-89486;OQ419007.1:88864-89496;OQ419012.1:88831-89463;OQ419031.1:88835-89467;OQ419047.1:88837-89469;OQ451898.1:88716-89348;OQ451900.1:89629-90261;OQ581853.1:89655-90287;OQ581855.1:89736-90368;OQ721951.1:89669-90301;OQ721954.1:89626-90258;OR035652.1:89578-90210;OR035664.1:89578-90210;OR035702.1:89578-90210;OR146272.1:89594-90226;OR146330.1:89639-90271;OR264364.1:89649-90281;OR427332.1:89649-90281;OR459777.1:89633-90265;OR459797.1:89648-90280;OR943698.1:91941-92591;PP098589.2:89630-90262;PP496048.1:89634-90266;PP601206.1:91890-92522;PP776553.1:89555-90187;PP852971.1:89628-90260;PP852983.1:89634-90266;PP853000.1:89595-90227;PP853005.1:89603-90235;PP853007.1:89636-90268;PP853013.1:89609-90241;PP853027.1:89640-90272;PP853045.1:89621-90253;PP853048.1:89640-90272;PP860026.1:89638-90270;PP860027.1:89663-90295;PP860028.1:89603-90235;PP860031.1:89657-90289;PP860033.1:89662-90294;PQ153226.1:89628-90260;PQ178862.2:90741-91382;PQ221506.1:85717-86358;PQ221833.1:91883-92524;PQ340477.1:89611-90243;PQ340478.1:89596-90228;PQ340480.1:89675-90307;PQ340483.1:89659-90291;PQ340485.1:89645-90277;PQ340486.1:89660-90292;PQ340487.1:89685-90317;PQ340489.1:89660-90292;PQ340490.1:89611-90243;PQ340491.1:89666-90298;PQ340493.1:89645-90277;PQ340495.1:89675-90307;PQ340496.1:89708-90340;PQ368489.1:89631-90263;PQ451835.1:89716-90348;PQ451836.1:89648-90280;PQ451838.1:89699-90331;PQ451841.1:89659-90291;PQ451842.1:89611-90243 |
| OG0000053 | AY603973.1:92212-93156;AY741551.1:92216-93160;AY753185.1:92455-93399;DQ011154.1:92680-93624;DQ011155.1:92661-93605;EPI_ISL_13331598:90280-91224;EPI_ISL_13363142:90273-91217;EPI_ISL_13374487:90288-91232;EPI_ISL_13502582:90273-91217;EPI_ISL_13508471:90365-91309;EPI_ISL_13607904:90264-91208;EPI_ISL_13624509:90208-91152;EPI_ISL_13889442:90383-91327;EPI_ISL_13953610:90181-91125;EPI_ISL_14181948:90409-91353;EPI_ISL_14838587:92534-93478;EPI_ISL_15263355:90264-91208;EPI_ISL_15269699:90233-91177;EPI_ISL_15293002:90506-91450;EPI_ISL_15641561:90222-91166;EPI_ISL_15831211:90271-91215;EPI_ISL_15992098:90286-91230;EPI_ISL_16233784:90282-91226;EPI_ISL_16946400:90271-91215;EPI_ISL_17048206:89921-90865;EPI_ISL_17703567:90286-91230;EPI_ISL_17718458:90264-91208;EPI_ISL_18213374:90261-91205;EPI_ISL_18360394:89922-90866;EPI_ISL_18557816:90262-91206;EPI_ISL_18642365:90264-91208;EPI_ISL_18659846:90234-91178;EPI_ISL_18744049:90266-91210;EPI_ISL_19022858:90261-91205;EPI_ISL_19028778:90178-91122;EPI_ISL_19151800:90262-91206;EPI_ISL_19159116:90264-91208;EPI_ISL_19350788:91406-92350;EPI_ISL_19359677:90261-91205;EPI_ISL_19446306:90256-91200;EPI_ISL_19467692:92817-93761;JX878411.1:93525-94469;JX878425.1:93042-93986;JX878426.1:92491-93435;KC257460.1:92459-93403;KJ642612.1:90777-91721;KJ642613.1:92336-93280;KJ642616.1:91906-92850;KJ642618.1:90007-90951;KP849469.1:92850-93794;KP849471.1:92776-93720;LC831698.1:90262-91206;MN648051.1:90484-91428;MN702445.1:86193-87137;MN702446.1:92338-93282;MN702447.1:85706-86650;MN702449.1:86204-87148;MN702452.1:92379-93323;MT903338.1:90427-91371;MT903340.1:90264-91208;MT903341.1:81797-82741;MT903342.1:90279-91223;MT903347.1:92223-93167;ON568298.1:90410-91354;ON622713.1:90496-91440;ON622722.2:90267-91211;ON631963.1:90287-91231;ON649879.1:90097-91041;ON676707.1:90317-91261;ON755039.1:89927-90871;ON808413.1:90267-91211;ON880420.1:90494-91438;ON911481.2:90284-91228;ON918656.1:90299-91243;ON959133.1:90388-91332;ON959136.1:90389-91333;ON983168.1:88962-89906;OP019276.1:90261-91205;OP150924.1:90402-91346;OP160532.1:90227-91171;OP235282.1:90261-91205;OP289782.1:90264-91208;OP331335.1:90281-91225;OP382479.1:90268-91212;OP422341.1:90281-91225;OP434519.1:90260-91204;OP535324.1:90262-91206;OP535333.1:90371-91315;OP536770.1:90263-91207;OP597769.1:90285-91229;OP604566.1:90264-91208;OP612682.1:90189-91133;OP612684.1:90252-91196;OP612686.1:90253-91197;OP696841.1:87538-88482;OP879722.1:89487-90431;OQ419007.1:89497-90441;OQ419012.1:89464-90408;OQ419031.1:89468-90412;OQ419047.1:89470-90414;OQ451898.1:89349-90293;OQ451900.1:90262-91206;OQ581853.1:90288-91232;OQ581855.1:90369-91313;OQ721951.1:90302-91246;OQ721954.1:90259-91203;OR035652.1:90211-91155;OR035664.1:90211-91155;OR035702.1:90211-91155;OR146272.1:90227-91171;OR146330.1:90272-91216;OR264364.1:90282-91226;OR427332.1:90282-91226;OR459777.1:90266-91210;OR459797.1:90281-91225;OR943698.1:92592-93536;PP098589.2:90263-91207;PP496048.1:90267-91211;PP601206.1:92523-93467;PP776553.1:90188-91132;PP852971.1:90261-91205;PP852983.1:90267-91211;PP853000.1:90228-91172;PP853005.1:90236-91180;PP853007.1:90269-91213;PP853013.1:90242-91186;PP853027.1:90273-91217;PP853045.1:90254-91198;PP853048.1:90273-91217;PP860026.1:90271-91215;PP860027.1:90296-91240;PP860028.1:90236-91180;PP860031.1:90290-91234;PP860033.1:90295-91239;PQ153226.1:90261-91205;PQ178862.2:91383-92327;PQ221506.1:86359-87303;PQ221833.1:92525-93469;PQ340477.1:90244-91188;PQ340478.1:90229-91173;PQ340480.1:90308-91252;PQ340483.1:90292-91236;PQ340485.1:90278-91222;PQ340486.1:90293-91237;PQ340487.1:90318-91262;PQ340489.1:90293-91237;PQ340490.1:90244-91188;PQ340491.1:90299-91243;PQ340493.1:90278-91222;PQ340495.1:90308-91252;PQ340496.1:90341-91285;PQ368489.1:90264-91208;PQ451835.1:90349-91293;PQ451836.1:90281-91225;PQ451838.1:90332-91276;PQ451841.1:90292-91236;PQ451842.1:90244-91188 |
| OG0000054 | AY603973.1:96601-97302;AY741551.1:96605-97306;AY753185.1:96844-97545;DQ011154.1:97075-97776;DQ011155.1:97056-97757;EPI_ISL_13331598:94675-95376;EPI_ISL_13363142:94668-95369;EPI_ISL_13374487:94683-95384;EPI_ISL_13502582:94668-95369;EPI_ISL_13508471:94760-95461;EPI_ISL_13607904:94659-95360;EPI_ISL_13624509:94603-95304;EPI_ISL_13889442:94778-95479;EPI_ISL_13953610:94576-95277;EPI_ISL_14181948:94804-95505;EPI_ISL_14838587:96929-97630;EPI_ISL_15263355:94659-95360;EPI_ISL_15269699:94628-95329;EPI_ISL_15293002:94901-95602;EPI_ISL_15641561:94617-95318;EPI_ISL_15831211:94666-95367;EPI_ISL_15992098:94681-95382;EPI_ISL_16233784:94677-95378;EPI_ISL_16946400:94666-95367;EPI_ISL_17048206:94316-95017;EPI_ISL_17703567:94681-95382;EPI_ISL_17718458:94659-95360;EPI_ISL_18213374:94656-95357;EPI_ISL_18360394:94317-95018;EPI_ISL_18557816:94657-95358;EPI_ISL_18642365:94659-95360;EPI_ISL_18659846:94629-95330;EPI_ISL_18744049:94661-95362;EPI_ISL_19022858:94656-95357;EPI_ISL_19028778:94573-95274;EPI_ISL_19151800:94657-95358;EPI_ISL_19159116:94659-95360;EPI_ISL_19350788:95801-96502;EPI_ISL_19359677:94656-95357;EPI_ISL_19446306:94651-95352;EPI_ISL_19467692:97212-97913;JX878411.1:97920-98621;JX878425.1:97437-98138;JX878426.1:96886-97587;KC257460.1:96854-97555;KJ642612.1:95172-95873;KJ642613.1:96731-97432;KJ642616.1:96295-96996;KJ642618.1:94402-95103;KP849469.1:97245-97946;KP849471.1:97171-97872;LC831698.1:94657-95358;MN648051.1:94879-95580;MN702445.1:90588-91289;MN702446.1:96733-97434;MN702447.1:90101-90802;MN702449.1:90599-91300;MN702452.1:96774-97475;MT903338.1:94822-95523;MT903340.1:94659-95360;MT903341.1:86192-86893;MT903342.1:94674-95375;MT903347.1:96612-97313;ON568298.1:94805-95506;ON622713.1:94891-95592;ON622722.2:94662-95363;ON631963.1:94682-95383;ON649879.1:94492-95193;ON676707.1:94712-95413;ON755039.1:94322-95023;ON808413.1:94662-95363;ON880420.1:94889-95590;ON911481.2:94679-95380;ON918656.1:94694-95395;ON959133.1:94783-95484;ON959136.1:94784-95485;ON983168.1:93357-94058;OP019276.1:94656-95357;OP150924.1:94797-95498;OP160532.1:94622-95323;OP235282.1:94656-95357;OP289782.1:94659-95360;OP331335.1:94676-95377;OP382479.1:94663-95364;OP422341.1:94676-95377;OP434519.1:94655-95356;OP535324.1:94657-95358;OP535333.1:94766-95467;OP536770.1:94658-95359;OP597769.1:94680-95381;OP604566.1:94659-95360;OP612682.1:94584-95285;OP612684.1:94647-95348;OP612686.1:94648-95349;OP696841.1:91933-92634;OP879722.1:93882-94583;OQ419007.1:93892-94593;OQ419012.1:93859-94560;OQ419031.1:93863-94564;OQ419047.1:93865-94566;OQ451898.1:93744-94445;OQ451900.1:94657-95358;OQ581853.1:94683-95384;OQ581855.1:94764-95465;OQ721951.1:94697-95398;OQ721954.1:94654-95355;OR035652.1:94606-95307;OR035664.1:94606-95307;OR035702.1:94606-95307;OR146272.1:94622-95323;OR146330.1:94667-95368;OR264364.1:94677-95378;OR427332.1:94677-95378;OR459777.1:94661-95362;OR459797.1:94676-95377;OR943698.1:96987-97688;PP098589.2:94658-95359;PP496048.1:94662-95363;PP601206.1:96918-97619;PP776553.1:94583-95284;PP852971.1:94656-95357;PP852983.1:94662-95363;PP853000.1:94623-95324;PP853005.1:94631-95332;PP853007.1:94664-95365;PP853013.1:94637-95338;PP853027.1:94668-95369;PP853045.1:94649-95350;PP853048.1:94668-95369;PP860026.1:94666-95367;PP860027.1:94691-95392;PP860028.1:94631-95332;PP860031.1:94685-95386;PP860033.1:94690-95391;PQ153226.1:94656-95357;PQ178862.2:95778-96479;PQ221506.1:90754-91455;PQ221833.1:96920-97621;PQ340477.1:94639-95340;PQ340478.1:94624-95325;PQ340480.1:94703-95404;PQ340483.1:94687-95388;PQ340485.1:94673-95374;PQ340486.1:94688-95389;PQ340487.1:94713-95414;PQ340489.1:94688-95389;PQ340490.1:94639-95340;PQ340491.1:94694-95395;PQ340493.1:94673-95374;PQ340495.1:94703-95404;PQ340496.1:94736-95437;PQ368489.1:94659-95360;PQ451835.1:94744-95445;PQ451836.1:94676-95377;PQ451838.1:94727-95428;PQ451841.1:94687-95388;PQ451842.1:94639-95340 |
| OG0000055 | AY603973.1:97990-100347;AY741551.1:97994-100351;AY753185.1:98233-100590;DQ011154.1:98464-100821;DQ011155.1:98445-100802;EPI_ISL_13331598:96064-98421;EPI_ISL_13363142:96057-98414;EPI_ISL_13374487:96072-98429;EPI_ISL_13502582:96057-98414;EPI_ISL_13508471:96149-98506;EPI_ISL_13607904:96048-98405;EPI_ISL_13624509:95992-98349;EPI_ISL_13889442:96167-98524;EPI_ISL_13953610:95965-98322;EPI_ISL_14181948:96193-98550;EPI_ISL_14838587:98318-100675;EPI_ISL_15263355:96048-98405;EPI_ISL_15269699:96017-98374;EPI_ISL_15293002:96290-98647;EPI_ISL_15641561:96006-98363;EPI_ISL_15831211:96055-98412;EPI_ISL_15992098:96070-98427;EPI_ISL_16233784:96066-98423;EPI_ISL_16946400:96055-98412;EPI_ISL_17048206:95705-98062;EPI_ISL_17703567:96070-98427;EPI_ISL_17718458:96048-98405;EPI_ISL_18213374:96045-98402;EPI_ISL_18360394:95706-98063;EPI_ISL_18557816:96046-98403;EPI_ISL_18642365:96048-98405;EPI_ISL_18659846:96018-98375;EPI_ISL_18744049:96050-98407;EPI_ISL_19022858:96045-98402;EPI_ISL_19028778:95962-98319;EPI_ISL_19151800:96046-98403;EPI_ISL_19159116:96048-98405;EPI_ISL_19350788:97190-99547;EPI_ISL_19359677:96045-98402;EPI_ISL_19446306:96040-98397;EPI_ISL_19467692:98601-100958;JX878411.1:99309-101666;JX878425.1:98826-101183;JX878426.1:98275-100632;KC257460.1:98243-100600;KJ642612.1:96561-98918;KJ642613.1:98120-100477;KJ642616.1:97684-100041;KJ642618.1:95791-98148;KP849469.1:98634-100991;KP849471.1:98560-100917;LC831698.1:96046-98403;MN648051.1:96268-98625;MN702445.1:91977-94334;MN702446.1:98122-100479;MN702447.1:91490-93847;MN702449.1:91988-94345;MN702452.1:98163-100520;MT903338.1:96211-98568;MT903340.1:96048-98405;MT903341.1:87581-89938;MT903342.1:96063-98420;MT903347.1:98001-100358;ON568298.1:96194-98551;ON622713.1:96280-98637;ON622722.2:96051-98408;ON631963.1:96071-98428;ON649879.1:95881-98238;ON676707.1:96101-98458;ON755039.1:95711-98068;ON808413.1:96051-98408;ON880420.1:96278-98635;ON911481.2:96068-98425;ON918656.1:96083-98440;ON959133.1:96172-98529;ON959136.1:96173-98530;ON983168.1:94746-97103;OP019276.1:96045-98402;OP150924.1:96186-98543;OP160532.1:96011-98368;OP235282.1:96045-98402;OP289782.1:96048-98405;OP331335.1:96065-98422;OP382479.1:96052-98409;OP422341.1:96065-98422;OP434519.1:96044-98401;OP535324.1:96046-98403;OP535333.1:96155-98512;OP536770.1:96047-98404;OP597769.1:96069-98426;OP604566.1:96048-98405;OP612682.1:95973-98330;OP612684.1:96036-98393;OP612686.1:96037-98394;OP696841.1:93322-95679;OP879722.1:95271-97628;OQ419007.1:95281-97638;OQ419012.1:95248-97605;OQ419031.1:95252-97609;OQ419047.1:95254-97611;OQ451898.1:95133-97490;OQ451900.1:96046-98403;OQ581853.1:96072-98429;OQ581855.1:96153-98510;OQ721951.1:96086-98443;OQ721954.1:96043-98400;OR035652.1:95995-98352;OR035664.1:95995-98352;OR035702.1:95995-98352;OR146272.1:96011-98368;OR146330.1:96056-98413;OR264364.1:96066-98423;OR427332.1:96066-98423;OR459777.1:96050-98407;OR459797.1:96065-98422;OR943698.1:98376-100733;PP098589.2:96047-98404;PP496048.1:96051-98408;PP601206.1:98307-100664;PP776553.1:95972-98329;PP852971.1:96045-98402;PP852983.1:96051-98408;PP853000.1:96012-98369;PP853005.1:96020-98377;PP853007.1:96053-98410;PP853013.1:96026-98383;PP853027.1:96057-98414;PP853045.1:96038-98395;PP853048.1:96057-98414;PP860026.1:96055-98412;PP860027.1:96080-98437;PP860028.1:96020-98377;PP860031.1:96074-98431;PP860033.1:96079-98436;PQ153226.1:96045-98402;PQ178862.2:97167-99524;PQ221506.1:92143-94500;PQ221833.1:98309-100666;PQ340477.1:96028-98385;PQ340478.1:96013-98370;PQ340480.1:96092-98449;PQ340483.1:96076-98433;PQ340485.1:96062-98419;PQ340486.1:96077-98434;PQ340487.1:96102-98459;PQ340489.1:96077-98434;PQ340490.1:96028-98385;PQ340491.1:96083-98440;PQ340493.1:96062-98419;PQ340495.1:96092-98449;PQ340496.1:96125-98482;PQ368489.1:96048-98405;PQ451835.1:96133-98490;PQ451836.1:96065-98422;PQ451838.1:96116-98473;PQ451841.1:96076-98433;PQ451842.1:96028-98385 |
| OG0000056 | AY603973.1:100387-102300;AY741551.1:100391-102304;AY753185.1:100630-102543;DQ011154.1:100861-102774;DQ011155.1:100842-102755;EPI_ISL_13331598:98461-100374;EPI_ISL_13363142:98454-100367;EPI_ISL_13374487:98469-100382;EPI_ISL_13502582:98454-100367;EPI_ISL_13508471:98546-100459;EPI_ISL_13607904:98445-100358;EPI_ISL_13624509:98389-100302;EPI_ISL_13889442:98564-100477;EPI_ISL_13953610:98362-100275;EPI_ISL_14181948:98590-100503;EPI_ISL_14838587:100715-102628;EPI_ISL_15263355:98445-100358;EPI_ISL_15269699:98414-100327;EPI_ISL_15293002:98687-100600;EPI_ISL_15641561:98403-100316;EPI_ISL_15831211:98452-100365;EPI_ISL_15992098:98467-100380;EPI_ISL_16233784:98463-100376;EPI_ISL_16946400:98452-100365;EPI_ISL_17048206:98102-100015;EPI_ISL_17703567:98467-100380;EPI_ISL_17718458:98445-100358;EPI_ISL_18213374:98442-100355;EPI_ISL_18360394:98103-100016;EPI_ISL_18557816:98443-100356;EPI_ISL_18642365:98445-100358;EPI_ISL_18659846:98415-100328;EPI_ISL_18744049:98447-100360;EPI_ISL_19022858:98442-100355;EPI_ISL_19028778:98359-100272;EPI_ISL_19151800:98443-100356;EPI_ISL_19159116:98445-100358;EPI_ISL_19350788:99587-101500;EPI_ISL_19359677:98442-100355;EPI_ISL_19446306:98437-100350;EPI_ISL_19467692:100998-102911;JX878411.1:101706-103619;JX878425.1:101223-103136;JX878426.1:100672-102585;KC257460.1:100640-102553;KJ642612.1:98958-100871;KJ642613.1:100517-102430;KJ642616.1:100081-101994;KJ642618.1:98188-100101;KP849469.1:101031-102944;KP849471.1:100957-102870;LC831698.1:98443-100356;MN648051.1:98665-100578;MN702445.1:94374-96287;MN702446.1:100519-102432;MN702447.1:93887-95800;MN702449.1:94385-96298;MN702452.1:100560-102473;MT903338.1:98608-100521;MT903340.1:98445-100358;MT903341.1:89978-91891;MT903342.1:98460-100373;MT903347.1:100398-102311;ON568298.1:98591-100504;ON622713.1:98677-100590;ON622722.2:98448-100361;ON631963.1:98468-100381;ON649879.1:98278-100191;ON676707.1:98498-100411;ON755039.1:98108-100021;ON808413.1:98448-100361;ON880420.1:98675-100588;ON911481.2:98465-100378;ON918656.1:98480-100393;ON959133.1:98569-100482;ON959136.1:98570-100483;ON983168.1:97143-99056;OP019276.1:98442-100355;OP150924.1:98583-100496;OP160532.1:98408-100321;OP235282.1:98442-100355;OP289782.1:98445-100358;OP331335.1:98462-100375;OP382479.1:98449-100362;OP422341.1:98462-100375;OP434519.1:98441-100354;OP535324.1:98443-100356;OP535333.1:98552-100465;OP536770.1:98444-100357;OP597769.1:98466-100379;OP604566.1:98445-100358;OP612682.1:98370-100283;OP612684.1:98433-100346;OP612686.1:98434-100347;OP696841.1:95719-97632;OP879722.1:97668-99581;OQ419007.1:97678-99591;OQ419012.1:97645-99558;OQ419031.1:97649-99562;OQ419047.1:97651-99564;OQ451898.1:97530-99443;OQ451900.1:98443-100356;OQ581853.1:98469-100382;OQ581855.1:98550-100463;OQ721951.1:98483-100396;OQ721954.1:98440-100353;OR035652.1:98392-100305;OR035664.1:98392-100305;OR035702.1:98392-100305;OR146272.1:98408-100321;OR146330.1:98453-100366;OR264364.1:98463-100376;OR427332.1:98463-100376;OR459777.1:98447-100360;OR459797.1:98462-100375;OR943698.1:100773-102686;PP098589.2:98444-100357;PP496048.1:98448-100361;PP601206.1:100704-102617;PP776553.1:98369-100282;PP852971.1:98442-100355;PP852983.1:98448-100361;PP853000.1:98409-100322;PP853005.1:98417-100330;PP853007.1:98450-100363;PP853013.1:98423-100336;PP853027.1:98454-100367;PP853045.1:98435-100348;PP853048.1:98454-100367;PP860026.1:98452-100365;PP860027.1:98477-100390;PP860028.1:98417-100330;PP860031.1:98471-100384;PP860033.1:98476-100389;PQ153226.1:98442-100355;PQ178862.2:99564-101477;PQ221506.1:94540-96453;PQ221833.1:100706-102619;PQ340477.1:98425-100338;PQ340478.1:98410-100323;PQ340480.1:98489-100402;PQ340483.1:98473-100386;PQ340485.1:98459-100372;PQ340486.1:98474-100387;PQ340487.1:98499-100412;PQ340489.1:98474-100387;PQ340490.1:98425-100338;PQ340491.1:98480-100393;PQ340493.1:98459-100372;PQ340495.1:98489-100402;PQ340496.1:98522-100435;PQ368489.1:98445-100358;PQ451835.1:98530-100443;PQ451836.1:98462-100375;PQ451838.1:98513-100426;PQ451841.1:98473-100386;PQ451842.1:98425-100338 |
| OG0000057 | AY603973.1:113881-114366;AY741551.1:113885-114370;AY753185.1:114124-114609;DQ011154.1:114356-114841;DQ011155.1:114337-114822;EPI_ISL_13331598:111955-112440;EPI_ISL_13363142:111948-112433;EPI_ISL_13374487:111963-112448;EPI_ISL_13502582:111948-112433;EPI_ISL_13508471:112040-112525;EPI_ISL_13607904:111939-112424;EPI_ISL_13624509:111883-112368;EPI_ISL_13889442:112058-112543;EPI_ISL_13953610:111856-112341;EPI_ISL_14181948:112084-112569;EPI_ISL_14838587:114210-114695;EPI_ISL_15263355:111939-112424;EPI_ISL_15269699:111908-112393;EPI_ISL_15293002:112181-112666;EPI_ISL_15641561:111897-112382;EPI_ISL_15831211:111946-112431;EPI_ISL_15992098:111961-112446;EPI_ISL_16233784:111957-112442;EPI_ISL_16946400:111946-112431;EPI_ISL_17048206:111596-112081;EPI_ISL_17703567:111961-112446;EPI_ISL_17718458:111939-112424;EPI_ISL_18213374:111936-112421;EPI_ISL_18360394:111597-112082;EPI_ISL_18557816:111937-112422;EPI_ISL_18642365:111939-112424;EPI_ISL_18659846:111909-112394;EPI_ISL_18744049:111941-112426;EPI_ISL_19022858:111936-112421;EPI_ISL_19028778:111853-112338;EPI_ISL_19151800:111937-112422;EPI_ISL_19159116:111939-112424;EPI_ISL_19350788:113081-113566;EPI_ISL_19359677:111936-112421;EPI_ISL_19446306:111931-112416;EPI_ISL_19467692:114492-114977;JX878411.1:115201-115686;JX878425.1:114718-115203;JX878426.1:114168-114653;KC257460.1:114135-114620;KJ642612.1:112452-112937;KJ642613.1:114012-114497;KJ642616.1:113575-114060;KJ642618.1:111683-112168;KP849469.1:114526-115011;KP849471.1:114452-114937;LC831698.1:111937-112422;MN648051.1:112159-112644;MN702445.1:107869-108354;MN702446.1:114014-114499;MN702447.1:107382-107867;MN702449.1:107880-108365;MN702452.1:114055-114540;MT903338.1:112102-112587;MT903340.1:111939-112424;MT903341.1:103472-103957;MT903342.1:111954-112439;MT903347.1:113892-114377;ON568298.1:112085-112570;ON622713.1:112171-112656;ON622722.2:111942-112427;ON631963.1:111962-112447;ON649879.1:111772-112257;ON676707.1:111992-112477;ON755039.1:111602-112087;ON808413.1:111942-112427;ON880420.1:112169-112654;ON911481.2:111959-112444;ON918656.1:111974-112459;ON959133.1:112063-112548;ON959136.1:112064-112549;ON983168.1:110637-111122;OP019276.1:111936-112421;OP150924.1:112077-112562;OP160532.1:111902-112387;OP235282.1:111936-112421;OP289782.1:111939-112424;OP331335.1:111956-112441;OP382479.1:111943-112428;OP422341.1:111956-112441;OP434519.1:111935-112420;OP535324.1:111937-112422;OP535333.1:112046-112531;OP536770.1:111938-112423;OP597769.1:111960-112445;OP604566.1:111939-112424;OP612682.1:111864-112349;OP612684.1:111927-112412;OP612686.1:111928-112413;OP696841.1:109213-109698;OP879722.1:111162-111647;OQ419007.1:111172-111657;OQ419012.1:111139-111624;OQ419031.1:111143-111628;OQ419047.1:111145-111630;OQ451898.1:111024-111509;OQ451900.1:111937-112422;OQ581853.1:111963-112448;OQ581855.1:112044-112529;OQ721951.1:111977-112462;OQ721954.1:111934-112419;OR035652.1:111886-112371;OR035664.1:111886-112371;OR035702.1:111886-112371;OR146272.1:111902-112387;OR146330.1:111947-112432;OR264364.1:111957-112442;OR427332.1:111957-112442;OR459777.1:111941-112426;OR459797.1:111956-112441;OR943698.1:114268-114753;PP098589.2:111938-112423;PP496048.1:111942-112427;PP601206.1:114198-114683;PP776553.1:111863-112348;PP852971.1:111936-112421;PP852983.1:111942-112427;PP853000.1:111903-112388;PP853005.1:111911-112396;PP853007.1:111944-112429;PP853013.1:111917-112402;PP853027.1:111948-112433;PP853045.1:111929-112414;PP853048.1:111948-112433;PP860026.1:111946-112431;PP860027.1:111972-112457;PP860028.1:111911-112396;PP860031.1:111965-112450;PP860033.1:111971-112456;PQ153226.1:111936-112421;PQ178862.2:113058-113543;PQ221506.1:108035-108520;PQ221833.1:114201-114686;PQ340477.1:111919-112404;PQ340478.1:111904-112389;PQ340480.1:111983-112468;PQ340483.1:111967-112452;PQ340485.1:111953-112438;PQ340486.1:111968-112453;PQ340487.1:111993-112478;PQ340489.1:111968-112453;PQ340490.1:111919-112404;PQ340491.1:111974-112459;PQ340493.1:111953-112438;PQ340495.1:111983-112468;PQ340496.1:112016-112501;PQ368489.1:111939-112424;PQ451835.1:112024-112509;PQ451836.1:111956-112441;PQ451838.1:112007-112492;PQ451841.1:111967-112452;PQ451842.1:111919-112404 |
| OG0000058 | AY603973.1:117691-118569;AY741551.1:117695-118573;AY753185.1:117934-118812;DQ011154.1:118166-119044;DQ011155.1:118147-119025;EPI_ISL_13331598:115765-116643;EPI_ISL_13363142:115758-116636;EPI_ISL_13374487:115773-116651;EPI_ISL_13502582:115758-116636;EPI_ISL_13508471:115850-116728;EPI_ISL_13607904:115749-116627;EPI_ISL_13624509:115693-116571;EPI_ISL_13889442:115868-116746;EPI_ISL_13953610:115666-116544;EPI_ISL_14181948:115894-116772;EPI_ISL_14838587:118020-118898;EPI_ISL_15263355:115749-116627;EPI_ISL_15269699:115718-116596;EPI_ISL_15293002:115991-116869;EPI_ISL_15641561:115707-116585;EPI_ISL_15831211:115756-116634;EPI_ISL_15992098:115771-116649;EPI_ISL_16233784:115767-116645;EPI_ISL_16946400:115756-116634;EPI_ISL_17048206:115406-116284;EPI_ISL_17703567:115771-116649;EPI_ISL_17718458:115749-116627;EPI_ISL_18213374:115746-116624;EPI_ISL_18360394:115407-116285;EPI_ISL_18557816:115747-116625;EPI_ISL_18642365:115749-116627;EPI_ISL_18659846:115719-116597;EPI_ISL_18744049:115751-116629;EPI_ISL_19022858:115746-116624;EPI_ISL_19028778:115663-116541;EPI_ISL_19151800:115747-116625;EPI_ISL_19159116:115749-116627;EPI_ISL_19350788:116891-117769;EPI_ISL_19359677:115746-116624;EPI_ISL_19446306:115741-116619;EPI_ISL_19467692:118302-119180;JX878411.1:119011-119889;JX878425.1:118528-119406;JX878426.1:117978-118856;KC257460.1:117945-118823;KJ642612.1:116262-117140;KJ642613.1:117822-118700;KJ642616.1:117385-118263;KJ642618.1:115493-116371;KP849469.1:118336-119214;KP849471.1:118262-119140;LC831698.1:115747-116625;MN648051.1:115969-116847;MN702445.1:111679-112557;MN702446.1:117824-118702;MN702447.1:111192-112070;MN702449.1:111690-112568;MN702452.1:117865-118743;MT903338.1:115912-116790;MT903340.1:115749-116627;MT903341.1:107282-108160;MT903342.1:115764-116642;MT903347.1:117702-118580;ON568298.1:115895-116773;ON622713.1:115981-116859;ON622722.2:115752-116630;ON631963.1:115772-116650;ON649879.1:115582-116460;ON676707.1:115802-116680;ON755039.1:115412-116290;ON808413.1:115752-116630;ON880420.1:115979-116857;ON911481.2:115769-116647;ON918656.1:115784-116662;ON959133.1:115873-116751;ON959136.1:115874-116752;ON983168.1:114447-115325;OP019276.1:115746-116624;OP150924.1:115887-116765;OP160532.1:115712-116590;OP235282.1:115746-116624;OP289782.1:115749-116627;OP331335.1:115766-116644;OP382479.1:115753-116631;OP422341.1:115766-116644;OP434519.1:115745-116623;OP535324.1:115747-116625;OP535333.1:115856-116734;OP536770.1:115748-116626;OP597769.1:115770-116648;OP604566.1:115749-116627;OP612682.1:115674-116552;OP612684.1:115737-116615;OP612686.1:115738-116616;OP696841.1:113023-113901;OP879722.1:114972-115850;OQ419007.1:114982-115860;OQ419012.1:114949-115827;OQ419031.1:114953-115831;OQ419047.1:114955-115833;OQ451898.1:114834-115712;OQ451900.1:115747-116625;OQ581853.1:115773-116651;OQ581855.1:115854-116732;OQ721951.1:115787-116665;OQ721954.1:115744-116622;OR035652.1:115696-116574;OR035664.1:115696-116574;OR035702.1:115696-116574;OR146272.1:115712-116590;OR146330.1:115757-116635;OR264364.1:115767-116645;OR427332.1:115767-116645;OR459777.1:115751-116629;OR459797.1:115765-116643;OR943698.1:118078-118956;PP098589.2:115748-116626;PP496048.1:115752-116630;PP601206.1:118008-118886;PP776553.1:115673-116551;PP852971.1:115746-116624;PP852983.1:115752-116630;PP853000.1:115713-116591;PP853005.1:115721-116599;PP853007.1:115754-116632;PP853013.1:115727-116605;PP853027.1:115758-116636;PP853045.1:115739-116617;PP853048.1:115758-116636;PP860026.1:115756-116634;PP860027.1:115782-116660;PP860028.1:115721-116599;PP860031.1:115775-116653;PP860033.1:115781-116659;PQ153226.1:115746-116624;PQ178862.2:116868-117746;PQ221506.1:111845-112723;PQ221833.1:118011-118889;PQ340477.1:115729-116607;PQ340478.1:115714-116592;PQ340480.1:115793-116671;PQ340483.1:115777-116655;PQ340485.1:115763-116641;PQ340486.1:115778-116656;PQ340487.1:115803-116681;PQ340489.1:115778-116656;PQ340490.1:115729-116607;PQ340491.1:115784-116662;PQ340493.1:115763-116641;PQ340495.1:115793-116671;PQ340496.1:115826-116704;PQ368489.1:115749-116627;PQ451835.1:115834-116712;PQ451836.1:115766-116644;PQ451838.1:115817-116695;PQ451841.1:115777-116655;PQ451842.1:115729-116607 |
| OG0000059 | AY603973.1:121561-122517;AY741551.1:121583-122539;AY753185.1:121804-122760;DQ011154.1:122018-122974;DQ011155.1:121999-122955;EPI_ISL_13331598:119617-120573;EPI_ISL_13363142:119610-120566;EPI_ISL_13374487:119625-120581;EPI_ISL_13502582:119610-120566;EPI_ISL_13508471:119702-120658;EPI_ISL_13607904:119601-120557;EPI_ISL_13624509:119545-120501;EPI_ISL_13889442:119720-120676;EPI_ISL_13953610:119518-120474;EPI_ISL_14181948:119746-120702;EPI_ISL_14838587:121872-122828;EPI_ISL_15263355:119601-120557;EPI_ISL_15269699:119570-120526;EPI_ISL_15293002:119843-120799;EPI_ISL_15641561:119559-120515;EPI_ISL_15831211:119608-120564;EPI_ISL_15992098:119623-120579;EPI_ISL_16233784:119619-120575;EPI_ISL_16946400:119608-120564;EPI_ISL_17048206:119258-120214;EPI_ISL_17703567:119623-120579;EPI_ISL_17718458:119601-120557;EPI_ISL_18213374:119598-120554;EPI_ISL_18360394:119259-120215;EPI_ISL_18557816:119599-120555;EPI_ISL_18642365:119601-120557;EPI_ISL_18659846:119571-120527;EPI_ISL_18744049:119603-120559;EPI_ISL_19022858:119598-120554;EPI_ISL_19028778:119515-120471;EPI_ISL_19151800:119599-120555;EPI_ISL_19159116:119601-120557;EPI_ISL_19350788:120743-121699;EPI_ISL_19359677:119598-120554;EPI_ISL_19446306:119593-120549;EPI_ISL_19467692:122154-123110;JX878411.1:122863-123819;JX878425.1:122380-123336;JX878426.1:121830-122786;KC257460.1:121797-122753;KJ642612.1:120114-121070;KJ642613.1:121674-122630;KJ642616.1:121261-122217;KJ642618.1:119345-120301;KP849469.1:122188-123144;KP849471.1:122114-123070;LC831698.1:119599-120555;MN648051.1:119821-120777;MN702445.1:115531-116487;MN702446.1:121676-122632;MN702447.1:115044-116000;MN702449.1:115542-116498;MN702452.1:121717-122673;MT903338.1:119764-120720;MT903340.1:119601-120557;MT903341.1:111134-112090;MT903342.1:119616-120572;MT903347.1:121590-122546;ON568298.1:119747-120703;ON622713.1:119833-120789;ON622722.2:119604-120560;ON631963.1:119624-120580;ON649879.1:119434-120390;ON676707.1:119654-120610;ON755039.1:119264-120220;ON808413.1:119604-120560;ON880420.1:119831-120787;ON911481.2:119621-120577;ON918656.1:119636-120592;ON959133.1:119725-120681;ON959136.1:119726-120682;ON983168.1:118299-119255;OP019276.1:119598-120554;OP150924.1:119739-120695;OP160532.1:119564-120520;OP235282.1:119598-120554;OP289782.1:119601-120557;OP331335.1:119618-120574;OP382479.1:119605-120561;OP422341.1:119618-120574;OP434519.1:119597-120553;OP535324.1:119599-120555;OP535333.1:119708-120664;OP536770.1:119600-120556;OP597769.1:119622-120578;OP604566.1:119601-120557;OP612682.1:119526-120482;OP612684.1:119589-120545;OP612686.1:119590-120546;OP696841.1:116875-117831;OP879722.1:118824-119780;OQ419007.1:118834-119790;OQ419012.1:118801-119757;OQ419031.1:118805-119761;OQ419047.1:118807-119763;OQ451898.1:118686-119642;OQ451900.1:119599-120555;OQ581853.1:119625-120581;OQ581855.1:119706-120662;OQ721951.1:119639-120595;OQ721954.1:119596-120552;OR035652.1:119548-120504;OR035664.1:119548-120504;OR035702.1:119548-120504;OR146272.1:119564-120520;OR146330.1:119609-120565;OR264364.1:119619-120575;OR427332.1:119619-120575;OR459777.1:119603-120559;OR459797.1:119617-120573;OR943698.1:121930-122886;PP098589.2:119600-120556;PP496048.1:119604-120560;PP601206.1:121860-122816;PP776553.1:119525-120481;PP852971.1:119598-120554;PP852983.1:119604-120560;PP853000.1:119565-120521;PP853005.1:119573-120529;PP853007.1:119606-120562;PP853013.1:119579-120535;PP853027.1:119610-120566;PP853045.1:119591-120547;PP853048.1:119610-120566;PP860026.1:119608-120564;PP860027.1:119634-120590;PP860028.1:119573-120529;PP860031.1:119627-120583;PP860033.1:119633-120589;PQ153226.1:119598-120554;PQ178862.2:120720-121676;PQ221506.1:115697-116653;PQ221833.1:121863-122819;PQ340477.1:119581-120537;PQ340478.1:119566-120522;PQ340480.1:119645-120601;PQ340483.1:119629-120585;PQ340485.1:119615-120571;PQ340486.1:119630-120586;PQ340487.1:119655-120611;PQ340489.1:119630-120586;PQ340490.1:119581-120537;PQ340491.1:119636-120592;PQ340493.1:119615-120571;PQ340495.1:119645-120601;PQ340496.1:119678-120634;PQ368489.1:119601-120557;PQ451835.1:119686-120642;PQ451836.1:119618-120574;PQ451838.1:119669-120625;PQ451841.1:119629-120585;PQ451842.1:119581-120537 |
| OG0000060 | AY603973.1:127945-129225;AY741551.1:127967-129247;AY753185.1:128188-129468;DQ011154.1:128402-129682;DQ011155.1:128383-129663;EPI_ISL_13331598:126001-127281;EPI_ISL_13363142:125994-127274;EPI_ISL_13374487:126009-127289;EPI_ISL_13502582:125994-127274;EPI_ISL_13508471:126086-127366;EPI_ISL_13607904:125985-127265;EPI_ISL_13624509:125929-127209;EPI_ISL_13889442:126104-127384;EPI_ISL_13953610:125902-127182;EPI_ISL_14181948:126130-127410;EPI_ISL_14838587:128256-129536;EPI_ISL_15263355:125985-127265;EPI_ISL_15269699:125954-127234;EPI_ISL_15293002:126227-127507;EPI_ISL_15641561:125943-127223;EPI_ISL_15831211:125992-127272;EPI_ISL_15992098:126007-127287;EPI_ISL_16233784:126006-127286;EPI_ISL_16946400:125992-127272;EPI_ISL_17048206:125642-126922;EPI_ISL_17703567:126007-127287;EPI_ISL_17718458:125985-127265;EPI_ISL_18213374:125982-127262;EPI_ISL_18360394:125643-126923;EPI_ISL_18557816:125983-127263;EPI_ISL_18642365:125985-127265;EPI_ISL_18659846:125955-127235;EPI_ISL_18744049:125987-127267;EPI_ISL_19022858:125982-127262;EPI_ISL_19028778:125899-127179;EPI_ISL_19151800:125983-127263;EPI_ISL_19159116:125985-127265;EPI_ISL_19350788:127127-128407;EPI_ISL_19359677:125982-127262;EPI_ISL_19446306:125977-127257;EPI_ISL_19467692:128538-129818;JX878411.1:129247-130527;JX878425.1:128764-130044;JX878426.1:128214-129494;KC257460.1:128181-129461;KJ642612.1:126498-127778;KJ642613.1:128058-129338;KJ642616.1:127645-128925;KJ642618.1:125729-127009;KP849469.1:128572-129852;KP849471.1:128498-129778;LC831698.1:125983-127263;MN648051.1:126205-127485;MN702445.1:121915-123195;MN702446.1:128060-129340;MN702447.1:121428-122708;MN702449.1:121926-123206;MN702452.1:128101-129381;MT903338.1:126148-127428;MT903340.1:125985-127265;MT903341.1:117518-118798;MT903342.1:126000-127280;MT903347.1:127974-129254;ON568298.1:126131-127411;ON622713.1:126217-127497;ON622722.2:125988-127268;ON631963.1:126008-127288;ON649879.1:125818-127098;ON676707.1:126038-127318;ON755039.1:125648-126928;ON808413.1:125988-127268;ON880420.1:126215-127495;ON911481.2:126005-127285;ON918656.1:126020-127300;ON959133.1:126109-127389;ON959136.1:126110-127390;ON983168.1:124683-125963;OP019276.1:125982-127262;OP150924.1:126123-127403;OP160532.1:125948-127228;OP235282.1:125982-127262;OP289782.1:125985-127265;OP331335.1:126002-127282;OP382479.1:125989-127269;OP422341.1:126002-127282;OP434519.1:125981-127261;OP535324.1:125983-127263;OP535333.1:126092-127372;OP536770.1:125984-127264;OP597769.1:126006-127286;OP604566.1:125985-127265;OP612682.1:125910-127190;OP612684.1:125972-127252;OP612686.1:125974-127254;OP696841.1:123259-124539;OP879722.1:125208-126488;OQ419007.1:125218-126498;OQ419012.1:125185-126465;OQ419031.1:125189-126469;OQ419047.1:125191-126471;OQ451898.1:125070-126350;OQ451900.1:125983-127263;OQ581853.1:126009-127289;OQ581855.1:126090-127370;OQ721951.1:126023-127303;OQ721954.1:125980-127260;OR035652.1:125932-127212;OR035664.1:125932-127212;OR035702.1:125932-127212;OR146272.1:125948-127228;OR146330.1:125993-127273;OR264364.1:126003-127283;OR427332.1:126003-127283;OR459777.1:125987-127267;OR459797.1:126001-127281;OR943698.1:128314-129594;PP098589.2:125984-127264;PP496048.1:125988-127268;PP601206.1:128244-129524;PP776553.1:125909-127189;PP852971.1:125982-127262;PP852983.1:125988-127268;PP853000.1:125949-127229;PP853005.1:125957-127237;PP853007.1:125990-127270;PP853013.1:125963-127243;PP853027.1:125994-127274;PP853045.1:125975-127255;PP853048.1:125994-127274;PP860026.1:125992-127272;PP860027.1:126018-127298;PP860028.1:125957-127237;PP860031.1:126011-127291;PP860033.1:126017-127297;PQ153226.1:125982-127262;PQ178862.2:127104-128384;PQ221506.1:122081-123361;PQ221833.1:128247-129527;PQ340477.1:125965-127245;PQ340478.1:125950-127230;PQ340480.1:126029-127309;PQ340483.1:126013-127293;PQ340485.1:125999-127279;PQ340486.1:126014-127294;PQ340487.1:126039-127319;PQ340489.1:126014-127294;PQ340490.1:125965-127245;PQ340491.1:126020-127300;PQ340493.1:125999-127279;PQ340495.1:126029-127309;PQ340496.1:126062-127342;PQ368489.1:125985-127265;PQ451835.1:126070-127350;PQ451836.1:126002-127282;PQ451838.1:126053-127333;PQ451841.1:126013-127293;PQ451842.1:125965-127245 |
| OG0000061 | AY603973.1:144106-144636;AY741551.1:144151-144681;AY753185.1:144349-144879;DQ011154.1:144175-144705;DQ011155.1:144144-144674;EPI_ISL_13331598:142213-142743;EPI_ISL_13363142:142200-142730;EPI_ISL_13374487:142217-142747;EPI_ISL_13502582:142212-142742;EPI_ISL_13508471:142295-142825;EPI_ISL_13607904:142191-142721;EPI_ISL_13624509:142135-142665;EPI_ISL_13889442:142312-142842;EPI_ISL_13953610:142118-142648;EPI_ISL_14181948:142341-142871;EPI_ISL_14838587:144041-144571;EPI_ISL_15263355:142194-142724;EPI_ISL_15269699:142163-142693;EPI_ISL_15293002:142432-142962;EPI_ISL_15641561:142149-142679;EPI_ISL_15831211:142202-142732;EPI_ISL_15992098:142226-142756;EPI_ISL_16233784:142215-142745;EPI_ISL_16946400:142204-142734;EPI_ISL_17048206:141851-142381;EPI_ISL_17703567:142216-142746;EPI_ISL_17718458:142186-142716;EPI_ISL_18213374:142190-142720;EPI_ISL_18360394:141848-142378;EPI_ISL_18557816:142191-142721;EPI_ISL_18642365:142188-142718;EPI_ISL_18659846:142164-142694;EPI_ISL_18744049:142203-142733;EPI_ISL_19022858:142170-142700;EPI_ISL_19028778:142105-142635;EPI_ISL_19151800:142188-142718;EPI_ISL_19159116:142191-142721;EPI_ISL_19350788:142867-143397;EPI_ISL_19359677:142188-142718;EPI_ISL_19446306:142193-142723;EPI_ISL_19467692:144305-144835;JX878411.1:145020-145550;JX878425.1:144528-145058;JX878426.1:143981-144511;KC257460.1:143921-144451;KJ642612.1:142250-142780;KJ642613.1:143810-144340;KJ642616.1:143831-144361;KJ642618.1:141437-141967;KP849469.1:144342-144872;KP849471.1:144269-144799;LC831698.1:142189-142719;MN648051.1:142415-142945;MN702445.1:137667-138197;MN702446.1:143830-144360;MN702447.1:137204-137734;MN702449.1:137693-138223;MN702452.1:143877-144407;MT903338.1:142367-142897;MT903340.1:142201-142731;MT903341.1:133735-134265;MT903342.1:142219-142749;MT903347.1:144172-144702;ON568298.1:142342-142872;ON622713.1:142423-142953;ON622722.2:142194-142724;ON631963.1:142220-142750;ON649879.1:142026-142556;ON676707.1:142247-142777;ON755039.1:141848-142378;ON808413.1:142185-142715;ON880420.1:142421-142951;ON911481.2:142206-142736;ON918656.1:142228-142758;ON959133.1:142317-142847;ON959136.1:142318-142848;ON983168.1:140893-141423;OP019276.1:142188-142718;OP150924.1:142331-142861;OP160532.1:142154-142684;OP235282.1:142188-142718;OP289782.1:142191-142721;OP331335.1:142221-142751;OP382479.1:142198-142728;OP422341.1:142211-142741;OP434519.1:142187-142717;OP535324.1:142201-142731;OP535333.1:142308-142838;OP536770.1:142191-142721;OP597769.1:142225-142755;OP604566.1:142191-142721;OP612682.1:142126-142656;OP612684.1:142177-142707;OP612686.1:142179-142709;OP696841.1:139467-139997;OP879722.1:141414-141944;OQ419007.1:141417-141947;OQ419012.1:141391-141921;OQ419031.1:141395-141925;OQ419047.1:141397-141927;OQ451898.1:141278-141808;OQ451900.1:142191-142721;OQ581853.1:142217-142747;OQ581855.1:142298-142828;OQ721951.1:142218-142748;OQ721954.1:142191-142721;OR035652.1:142137-142667;OR035664.1:142141-142671;OR035702.1:142138-142668;OR146272.1:142157-142687;OR146330.1:142201-142731;OR264364.1:142212-142742;OR427332.1:142212-142742;OR459777.1:142191-142721;OR459797.1:142210-142740;OR943698.1:144051-144581;PP098589.2:142190-142720;PP496048.1:142194-142724;PP601206.1:144011-144541;PP776553.1:142114-142644;PP852971.1:142189-142719;PP852983.1:142206-142736;PP853000.1:142157-142687;PP853005.1:142165-142695;PP853007.1:142208-142738;PP853013.1:142180-142710;PP853027.1:142213-142743;PP853045.1:142173-142703;PP853048.1:142198-142728;PP860026.1:142203-142733;PP860027.1:142257-142787;PP860028.1:142166-142696;PP860031.1:142239-142769;PP860033.1:142240-142770;PQ153226.1:142188-142718;PQ178862.2:142847-143377;PQ221506.1:137839-138369;PQ221833.1:144014-144544;PQ340477.1:142175-142705;PQ340478.1:142157-142687;PQ340480.1:142235-142765;PQ340483.1:142221-142751;PQ340485.1:142204-142734;PQ340486.1:142222-142752;PQ340487.1:142246-142776;PQ340489.1:142221-142751;PQ340490.1:142173-142703;PQ340491.1:142228-142758;PQ340493.1:142212-142742;PQ340495.1:142238-142768;PQ340496.1:142273-142803;PQ368489.1:142204-142734;PQ451835.1:142278-142808;PQ451836.1:142216-142746;PQ451838.1:142266-142796;PQ451841.1:142229-142759;PQ451842.1:142175-142705 |
| OG0000062 | AY603973.1:144682-145368;AY741551.1:144727-145413;AY753185.1:144925-145611;DQ011154.1:144750-145388;DQ011155.1:144719-145357;EPI_ISL_13331598:142789-143475;EPI_ISL_13363142:142776-143462;EPI_ISL_13374487:142793-143479;EPI_ISL_13502582:142788-143474;EPI_ISL_13508471:142871-143557;EPI_ISL_13607904:142767-143453;EPI_ISL_13624509:142711-143397;EPI_ISL_13889442:142888-143574;EPI_ISL_13953610:142694-143380;EPI_ISL_14181948:142917-143603;EPI_ISL_14838587:144616-145254;EPI_ISL_15263355:142770-143456;EPI_ISL_15269699:142739-143425;EPI_ISL_15293002:143008-143694;EPI_ISL_15641561:142725-143411;EPI_ISL_15831211:142778-143464;EPI_ISL_15992098:142802-143488;EPI_ISL_16233784:142791-143477;EPI_ISL_16946400:142780-143466;EPI_ISL_17048206:142427-143113;EPI_ISL_17703567:142792-143478;EPI_ISL_17718458:142762-143448;EPI_ISL_18213374:142766-143452;EPI_ISL_18360394:142424-143110;EPI_ISL_18557816:142767-143453;EPI_ISL_18642365:142764-143450;EPI_ISL_18659846:142740-143426;EPI_ISL_18744049:142779-143465;EPI_ISL_19022858:142746-143432;EPI_ISL_19028778:142681-143367;EPI_ISL_19151800:142764-143450;EPI_ISL_19159116:142767-143453;EPI_ISL_19350788:143442-144032;EPI_ISL_19359677:142764-143450;EPI_ISL_19446306:142769-143455;EPI_ISL_19467692:144880-145518;JX878411.1:145595-146233;JX878425.1:145103-145741;JX878426.1:144556-145194;KC257460.1:144496-145134;KJ642612.1:142825-143463;KJ642613.1:144385-145023;KJ642616.1:144407-145093;KJ642618.1:142012-142650;KP849469.1:144917-145555;KP849471.1:144844-145482;LC831698.1:142765-143451;MN648051.1:142991-143677;MN702445.1:138242-138880;MN702446.1:144405-145043;MN702447.1:137779-138417;MN702449.1:138268-138906;MN702452.1:144452-145090;MT903338.1:142943-143629;MT903340.1:142777-143463;MT903341.1:134311-134997;MT903342.1:142795-143481;MT903347.1:144748-145434;ON568298.1:142918-143604;ON622713.1:142999-143685;ON622722.2:142770-143456;ON631963.1:142796-143482;ON649879.1:142602-143288;ON676707.1:142823-143509;ON755039.1:142424-143110;ON808413.1:142761-143447;ON880420.1:142997-143683;ON911481.2:142782-143468;ON918656.1:142804-143490;ON959133.1:142893-143579;ON959136.1:142894-143580;ON983168.1:141469-142155;OP019276.1:142764-143450;OP150924.1:142907-143593;OP160532.1:142730-143416;OP235282.1:142764-143450;OP289782.1:142767-143453;OP331335.1:142797-143483;OP382479.1:142774-143460;OP422341.1:142787-143473;OP434519.1:142763-143449;OP535324.1:142777-143463;OP535333.1:142884-143570;OP536770.1:142767-143453;OP597769.1:142801-143487;OP604566.1:142767-143453;OP612682.1:142702-143388;OP612684.1:142753-143439;OP612686.1:142755-143441;OP696841.1:140043-140729;OP879722.1:141990-142676;OQ419007.1:141993-142679;OQ419012.1:141967-142653;OQ419031.1:141971-142657;OQ419047.1:141973-142659;OQ451898.1:141854-142540;OQ451900.1:142767-143453;OQ581853.1:142793-143479;OQ581855.1:142874-143560;OQ721951.1:142794-143480;OQ721954.1:142767-143453;OR035652.1:142713-143399;OR035664.1:142717-143403;OR035702.1:142714-143400;OR146272.1:142733-143419;OR146330.1:142777-143463;OR264364.1:142788-143474;OR427332.1:142788-143474;OR459777.1:142767-143453;OR459797.1:142786-143472;OR943698.1:144626-145264;PP098589.2:142766-143452;PP496048.1:142770-143456;PP601206.1:144586-145224;PP776553.1:142690-143376;PP852971.1:142765-143451;PP852983.1:142782-143468;PP853000.1:142733-143419;PP853005.1:142741-143427;PP853007.1:142784-143470;PP853013.1:142756-143442;PP853027.1:142789-143475;PP853045.1:142749-143435;PP853048.1:142774-143460;PP860026.1:142779-143465;PP860027.1:142833-143519;PP860028.1:142742-143428;PP860031.1:142815-143501;PP860033.1:142816-143502;PQ153226.1:142764-143450;PQ178862.2:143422-144012;PQ221506.1:138414-139052;PQ221833.1:144589-145227;PQ340477.1:142751-143437;PQ340478.1:142733-143419;PQ340480.1:142811-143497;PQ340483.1:142797-143483;PQ340485.1:142780-143466;PQ340486.1:142798-143484;PQ340487.1:142822-143508;PQ340489.1:142797-143483;PQ340490.1:142749-143435;PQ340491.1:142804-143490;PQ340493.1:142788-143474;PQ340495.1:142814-143500;PQ340496.1:142849-143535;PQ368489.1:142780-143466;PQ451835.1:142854-143540;PQ451836.1:142792-143478;PQ451838.1:142842-143528;PQ451841.1:142805-143491;PQ451842.1:142751-143437 |
| OG0000063 | AY603973.1:145420-146226;AY741551.1:145465-146271;AY753185.1:145663-146469;DQ011154.1:145490-146296;DQ011155.1:145459-146265;EPI_ISL_13331598:143527-144333;EPI_ISL_13363142:143514-144320;EPI_ISL_13374487:143531-144337;EPI_ISL_13502582:143526-144332;EPI_ISL_13508471:143609-144415;EPI_ISL_13607904:143505-144311;EPI_ISL_13624509:143449-144255;EPI_ISL_13889442:143626-144432;EPI_ISL_13953610:143432-144238;EPI_ISL_14181948:143655-144461;EPI_ISL_14838587:145356-146162;EPI_ISL_15263355:143508-144314;EPI_ISL_15269699:143477-144283;EPI_ISL_15293002:143746-144552;EPI_ISL_15641561:143463-144269;EPI_ISL_15831211:143516-144322;EPI_ISL_15992098:143540-144346;EPI_ISL_16233784:143529-144335;EPI_ISL_16946400:143518-144324;EPI_ISL_17048206:143165-143971;EPI_ISL_17703567:143530-144336;EPI_ISL_17718458:143500-144306;EPI_ISL_18213374:143504-144310;EPI_ISL_18360394:143162-143968;EPI_ISL_18557816:143505-144311;EPI_ISL_18642365:143502-144308;EPI_ISL_18659846:143478-144284;EPI_ISL_18744049:143517-144323;EPI_ISL_19022858:143484-144290;EPI_ISL_19028778:143419-144225;EPI_ISL_19151800:143502-144308;EPI_ISL_19159116:143505-144311;EPI_ISL_19350788:144134-144940;EPI_ISL_19359677:143502-144308;EPI_ISL_19446306:143507-144313;EPI_ISL_19467692:145620-146426;JX878411.1:146335-147141;JX878425.1:145843-146649;JX878426.1:145308-146114;KC257460.1:145236-146042;KJ642612.1:143565-144371;KJ642613.1:145125-145931;KJ642616.1:145145-145951;KJ642618.1:142752-143558;KP849469.1:145657-146463;KP849471.1:145584-146390;LC831698.1:143503-144309;MN648051.1:143729-144535;MN702445.1:138982-139788;MN702446.1:145145-145951;MN702447.1:138519-139325;MN702449.1:139008-139814;MN702452.1:145192-145998;MT903338.1:143681-144487;MT903340.1:143515-144321;MT903341.1:135049-135855;MT903342.1:143533-144339;MT903347.1:145486-146292;ON568298.1:143656-144462;ON622713.1:143737-144543;ON622722.2:143508-144314;ON631963.1:143534-144340;ON649879.1:143340-144146;ON676707.1:143561-144367;ON755039.1:143162-143968;ON808413.1:143499-144305;ON880420.1:143735-144541;ON911481.2:143520-144326;ON918656.1:143542-144348;ON959133.1:143631-144437;ON959136.1:143632-144438;ON983168.1:142207-143013;OP019276.1:143502-144308;OP150924.1:143645-144451;OP160532.1:143468-144274;OP235282.1:143502-144308;OP289782.1:143505-144311;OP331335.1:143535-144341;OP382479.1:143512-144318;OP422341.1:143525-144331;OP434519.1:143501-144307;OP535324.1:143515-144321;OP535333.1:143622-144428;OP536770.1:143505-144311;OP597769.1:143539-144345;OP604566.1:143505-144311;OP612682.1:143440-144246;OP612684.1:143491-144297;OP612686.1:143493-144299;OP696841.1:140781-141587;OP879722.1:142728-143534;OQ419007.1:142731-143537;OQ419012.1:142705-143511;OQ419031.1:142709-143515;OQ419047.1:142711-143517;OQ451898.1:142592-143398;OQ451900.1:143505-144311;OQ581853.1:143531-144337;OQ581855.1:143612-144418;OQ721951.1:143532-144338;OQ721954.1:143505-144311;OR035652.1:143451-144257;OR035664.1:143455-144261;OR035702.1:143452-144258;OR146272.1:143471-144277;OR146330.1:143515-144321;OR264364.1:143526-144332;OR427332.1:143526-144332;OR459777.1:143505-144311;OR459797.1:143524-144330;OR943698.1:145366-146172;PP098589.2:143504-144310;PP496048.1:143508-144314;PP601206.1:145326-146132;PP776553.1:143428-144234;PP852971.1:143503-144309;PP852983.1:143520-144326;PP853000.1:143471-144277;PP853005.1:143479-144285;PP853007.1:143522-144328;PP853013.1:143494-144300;PP853027.1:143527-144333;PP853045.1:143487-144293;PP853048.1:143512-144318;PP860026.1:143517-144323;PP860027.1:143571-144377;PP860028.1:143480-144286;PP860031.1:143553-144359;PP860033.1:143554-144360;PQ153226.1:143502-144308;PQ178862.2:144114-144920;PQ221506.1:139154-139960;PQ221833.1:145329-146135;PQ340477.1:143489-144295;PQ340478.1:143471-144277;PQ340480.1:143549-144355;PQ340483.1:143535-144341;PQ340485.1:143518-144324;PQ340486.1:143536-144342;PQ340487.1:143560-144366;PQ340489.1:143535-144341;PQ340490.1:143487-144293;PQ340491.1:143542-144348;PQ340493.1:143526-144332;PQ340495.1:143552-144358;PQ340496.1:143587-144393;PQ368489.1:143518-144324;PQ451835.1:143592-144398;PQ451836.1:143530-144336;PQ451838.1:143580-144386;PQ451841.1:143543-144349;PQ451842.1:143489-144295 |
| OG0000064 | AY603973.1:146302-146481;AY741551.1:146347-146526;AY753185.1:146545-146724;DQ011154.1:146371-146550;DQ011155.1:146340-146519;EPI_ISL_13331598:144409-144588;EPI_ISL_13363142:144396-144575;EPI_ISL_13374487:144413-144592;EPI_ISL_13502582:144408-144587;EPI_ISL_13508471:144491-144670;EPI_ISL_13607904:144387-144566;EPI_ISL_13624509:144331-144510;EPI_ISL_13889442:144508-144687;EPI_ISL_13953610:144314-144493;EPI_ISL_14181948:144537-144716;EPI_ISL_14838587:146237-146416;EPI_ISL_15263355:144390-144569;EPI_ISL_15269699:144359-144538;EPI_ISL_15293002:144628-144807;EPI_ISL_15641561:144345-144524;EPI_ISL_15831211:144398-144577;EPI_ISL_15992098:144422-144601;EPI_ISL_16233784:144411-144590;EPI_ISL_16946400:144400-144579;EPI_ISL_17048206:144047-144226;EPI_ISL_17703567:144412-144591;EPI_ISL_17718458:144382-144561;EPI_ISL_18213374:144386-144565;EPI_ISL_18360394:144044-144223;EPI_ISL_18557816:144387-144566;EPI_ISL_18642365:144384-144563;EPI_ISL_18659846:144360-144539;EPI_ISL_18744049:144399-144578;EPI_ISL_19022858:144366-144545;EPI_ISL_19028778:144301-144480;EPI_ISL_19151800:144384-144563;EPI_ISL_19159116:144387-144566;EPI_ISL_19350788:145016-145195;EPI_ISL_19359677:144384-144563;EPI_ISL_19446306:144389-144568;EPI_ISL_19467692:146501-146680;JX878411.1:147217-147396;JX878425.1:146725-146904;JX878426.1:146190-146369;KC257460.1:146117-146296;KJ642612.1:144447-144626;KJ642613.1:146006-146185;KJ642616.1:146027-146206;KJ642618.1:143634-143813;KP849469.1:146538-146717;KP849471.1:146466-146645;LC831698.1:144385-144564;MN648051.1:144611-144790;MN702445.1:139863-140042;MN702446.1:146026-146205;MN702447.1:139400-139579;MN702449.1:139889-140068;MN702452.1:146073-146252;MT903338.1:144563-144742;MT903340.1:144397-144576;MT903341.1:135931-136110;MT903342.1:144415-144594;MT903347.1:146368-146547;ON568298.1:144538-144717;ON622713.1:144619-144798;ON622722.2:144390-144569;ON631963.1:144416-144595;ON649879.1:144222-144401;ON676707.1:144443-144622;ON755039.1:144044-144223;ON808413.1:144381-144560;ON880420.1:144617-144796;ON911481.2:144402-144581;ON918656.1:144424-144603;ON959133.1:144513-144692;ON959136.1:144514-144693;ON983168.1:143089-143268;OP019276.1:144384-144563;OP150924.1:144527-144706;OP160532.1:144350-144529;OP235282.1:144384-144563;OP289782.1:144387-144566;OP331335.1:144417-144596;OP382479.1:144394-144573;OP422341.1:144407-144586;OP434519.1:144383-144562;OP535324.1:144397-144576;OP535333.1:144504-144683;OP536770.1:144387-144566;OP597769.1:144421-144600;OP604566.1:144387-144566;OP612682.1:144322-144501;OP612684.1:144373-144552;OP612686.1:144375-144554;OP696841.1:141663-141842;OP879722.1:143610-143789;OQ419007.1:143613-143792;OQ419012.1:143587-143766;OQ419031.1:143591-143770;OQ419047.1:143593-143772;OQ451898.1:143474-143653;OQ451900.1:144387-144566;OQ581853.1:144413-144592;OQ581855.1:144494-144673;OQ721951.1:144414-144593;OQ721954.1:144387-144566;OR035652.1:144333-144512;OR035664.1:144337-144516;OR035702.1:144334-144513;OR146272.1:144353-144532;OR146330.1:144397-144576;OR264364.1:144408-144587;OR427332.1:144408-144587;OR459777.1:144387-144566;OR459797.1:144406-144585;OR943698.1:146248-146427;PP098589.2:144386-144565;PP496048.1:144390-144569;PP601206.1:146207-146386;PP776553.1:144310-144489;PP852971.1:144385-144564;PP852983.1:144402-144581;PP853000.1:144353-144532;PP853005.1:144361-144540;PP853007.1:144404-144583;PP853013.1:144376-144555;PP853027.1:144409-144588;PP853045.1:144369-144548;PP853048.1:144394-144573;PP860026.1:144399-144578;PP860027.1:144453-144632;PP860028.1:144362-144541;PP860031.1:144435-144614;PP860033.1:144436-144615;PQ153226.1:144384-144563;PQ178862.2:144996-145175;PQ221506.1:140036-140215;PQ221833.1:146210-146389;PQ340477.1:144371-144550;PQ340478.1:144353-144532;PQ340480.1:144431-144610;PQ340483.1:144417-144596;PQ340485.1:144400-144579;PQ340486.1:144418-144597;PQ340487.1:144442-144621;PQ340489.1:144417-144596;PQ340490.1:144369-144548;PQ340491.1:144424-144603;PQ340493.1:144408-144587;PQ340495.1:144434-144613;PQ340496.1:144469-144648;PQ368489.1:144400-144579;PQ451835.1:144474-144653;PQ451836.1:144412-144591;PQ451838.1:144462-144641;PQ451841.1:144425-144604;PQ451842.1:144371-144550 |
| OG0000065 | AY603973.1:148903-149304;AY741551.1:148960-149361;AY753185.1:149158-149559;DQ011154.1:148990-149391;DQ011155.1:148961-149362;EPI_ISL_13331598:146999-147400;EPI_ISL_13363142:146986-147387;EPI_ISL_13374487:147003-147404;EPI_ISL_13502582:146998-147399;EPI_ISL_13508471:147081-147482;EPI_ISL_13607904:146977-147378;EPI_ISL_13624509:146921-147322;EPI_ISL_13889442:147098-147499;EPI_ISL_13953610:146904-147305;EPI_ISL_14181948:147127-147528;EPI_ISL_14838587:148859-149260;EPI_ISL_15263355:146980-147381;EPI_ISL_15269699:146949-147350;EPI_ISL_15293002:147218-147619;EPI_ISL_15641561:146935-147336;EPI_ISL_15831211:146988-147389;EPI_ISL_15992098:147012-147413;EPI_ISL_16233784:147001-147402;EPI_ISL_16946400:146990-147391;EPI_ISL_17048206:146637-147038;EPI_ISL_17703567:147002-147403;EPI_ISL_17718458:146972-147373;EPI_ISL_18213374:146976-147377;EPI_ISL_18360394:146694-146873;EPI_ISL_18557816:146977-147378;EPI_ISL_18642365:146974-147375;EPI_ISL_18659846:146950-147351;EPI_ISL_18744049:146989-147390;EPI_ISL_19022858:146956-147357;EPI_ISL_19028778:146891-147292;EPI_ISL_19151800:146974-147375;EPI_ISL_19159116:146977-147378;EPI_ISL_19350788:147620-148021;EPI_ISL_19359677:146974-147375;EPI_ISL_19446306:146979-147380;EPI_ISL_19467692:149120-149521;JX878411.1:149836-150237;JX878425.1:149346-149747;JX878426.1:148787-149188;KC257460.1:148738-149139;KJ642612.1:147044-147445;KJ642613.1:148625-149026;KJ642616.1:148633-149034;KJ642618.1:146230-146631;KP849469.1:149157-149558;KP849471.1:149087-149488;LC831698.1:146975-147376;MN648051.1:147201-147602;MN702445.1:142481-142882;MN702446.1:148644-149045;MN702447.1:142019-142420;MN702449.1:142521-142922;MN702452.1:148692-149093;MT903338.1:147134-147535;MT903340.1:146987-147388;MT903341.1:138528-138929;MT903342.1:147024-147425;MT903347.1:148981-149382;ON568298.1:147128-147529;ON622713.1:147209-147610;ON622722.2:146980-147381;ON631963.1:147006-147407;ON649879.1:146812-147213;ON676707.1:147033-147434;ON755039.1:146634-147035;ON808413.1:146971-147372;ON880420.1:147207-147608;ON911481.2:146992-147393;ON918656.1:147014-147415;ON959133.1:147103-147504;ON959136.1:147104-147505;ON983168.1:145679-146080;OP019276.1:146974-147375;OP150924.1:147117-147518;OP160532.1:146940-147341;OP235282.1:146974-147375;OP289782.1:146977-147378;OP331335.1:147007-147408;OP382479.1:146984-147385;OP422341.1:146997-147398;OP434519.1:146973-147374;OP535324.1:146987-147388;OP535333.1:147094-147495;OP536770.1:146977-147378;OP597769.1:147011-147412;OP604566.1:146977-147378;OP612682.1:146912-147313;OP612684.1:146963-147364;OP612686.1:146965-147366;OP696841.1:144253-144654;OP879722.1:146200-146601;OQ419007.1:146203-146604;OQ419012.1:146177-146578;OQ419031.1:146181-146582;OQ419047.1:146183-146584;OQ451898.1:146064-146465;OQ451900.1:146977-147378;OQ581853.1:147003-147404;OQ581855.1:147084-147485;OQ721951.1:147004-147405;OQ721954.1:146977-147378;OR035652.1:146923-147324;OR035664.1:146927-147328;OR035702.1:146924-147325;OR146272.1:146943-147344;OR146330.1:146987-147388;OR264364.1:146998-147399;OR427332.1:146998-147399;OR459777.1:146977-147378;OR459797.1:146996-147397;OR943698.1:148845-149246;PP098589.2:146976-147377;PP496048.1:146980-147381;PP601206.1:148804-149205;PP776553.1:146900-147301;PP852971.1:146975-147376;PP852983.1:146992-147393;PP853000.1:146943-147344;PP853005.1:146951-147352;PP853007.1:146994-147395;PP853013.1:146966-147367;PP853027.1:146999-147400;PP853045.1:146959-147360;PP853048.1:146984-147385;PP860026.1:146981-147382;PP860027.1:147024-147425;PP860028.1:146944-147345;PP860031.1:147006-147407;PP860033.1:147007-147408;PQ153226.1:146974-147375;PQ178862.2:147599-148000;PQ221506.1:142633-143034;PQ221833.1:148807-149208;PQ340477.1:146961-147362;PQ340478.1:146943-147344;PQ340480.1:147021-147422;PQ340483.1:147007-147408;PQ340485.1:146990-147391;PQ340486.1:147008-147409;PQ340487.1:147032-147433;PQ340489.1:147007-147408;PQ340490.1:146959-147360;PQ340491.1:147014-147415;PQ340493.1:146998-147399;PQ340495.1:147024-147425;PQ340496.1:147059-147460;PQ368489.1:146990-147391;PQ451835.1:147064-147465;PQ451836.1:147002-147403;PQ451838.1:147052-147453;PQ451841.1:147015-147416;PQ451842.1:146961-147362 |
| OG0000066 | AY603973.1:151357-151734;AY741551.1:151416-151793;AY753185.1:151612-151989;DQ011154.1:151461-151838;DQ011155.1:151422-151799;EPI_ISL_13331598:149453-149830;EPI_ISL_13363142:149440-149817;EPI_ISL_13374487:149457-149834;EPI_ISL_13502582:149452-149829;EPI_ISL_13508471:149535-149912;EPI_ISL_13607904:149431-149808;EPI_ISL_13624509:149375-149752;EPI_ISL_13889442:149552-149929;EPI_ISL_13953610:149351-149728;EPI_ISL_14181948:149581-149958;EPI_ISL_14838587:151321-151698;EPI_ISL_15263355:149434-149811;EPI_ISL_15269699:149403-149780;EPI_ISL_15293002:149672-150049;EPI_ISL_15641561:149389-149766;EPI_ISL_15831211:149442-149819;EPI_ISL_15992098:149466-149843;EPI_ISL_16233784:149455-149832;EPI_ISL_16946400:149444-149821;EPI_ISL_17048206:149091-149468;EPI_ISL_17703567:149456-149833;EPI_ISL_17718458:149419-149796;EPI_ISL_18213374:149430-149807;EPI_ISL_18360394:148737-149114;EPI_ISL_18557816:149431-149808;EPI_ISL_18642365:149428-149805;EPI_ISL_18659846:149404-149781;EPI_ISL_18744049:149436-149813;EPI_ISL_19022858:149410-149787;EPI_ISL_19028778:149345-149722;EPI_ISL_19151800:149428-149805;EPI_ISL_19159116:149431-149808;EPI_ISL_19350788:150098-150475;EPI_ISL_19359677:149428-149805;EPI_ISL_19446306:149433-149810;EPI_ISL_19467692:151592-151969;JX878411.1:152302-152679;JX878425.1:151809-152186;JX878426.1:151244-151621;KC257460.1:151205-151582;KJ642612.1:149503-149880;KJ642613.1:151088-151465;KJ642616.1:151088-151465;KJ642618.1:148701-149078;KP849469.1:151627-152004;KP849471.1:151552-151929;LC831698.1:149429-149806;MN648051.1:149655-150032;MN702445.1:144956-145333;MN702446.1:151120-151497;MN702447.1:144491-144868;MN702449.1:144994-145371;MN702452.1:151161-151538;MT903338.1:149588-149965;MT903340.1:149441-149818;MT903341.1:140982-141359;MT903342.1:149478-149855;MT903347.1:151436-151813;ON568298.1:149582-149959;ON622713.1:149663-150040;ON622722.2:149434-149811;ON631963.1:149460-149837;ON649879.1:149266-149643;ON676707.1:149480-149857;ON755039.1:149088-149465;ON808413.1:149425-149802;ON880420.1:149661-150038;ON911481.2:149446-149823;ON918656.1:149468-149845;ON959133.1:149557-149934;ON959136.1:149558-149935;ON983168.1:148134-148511;OP019276.1:149428-149805;OP150924.1:149571-149948;OP160532.1:149394-149771;OP235282.1:149428-149805;OP289782.1:149431-149808;OP331335.1:149454-149831;OP382479.1:149438-149815;OP422341.1:149451-149828;OP434519.1:149427-149804;OP535324.1:149441-149818;OP535333.1:149548-149925;OP536770.1:149431-149808;OP597769.1:149465-149842;OP604566.1:149431-149808;OP612682.1:149366-149743;OP612684.1:149417-149794;OP612686.1:149419-149796;OP696841.1:146707-147084;OP879722.1:148654-149031;OQ419007.1:148657-149034;OQ419012.1:148631-149008;OQ419031.1:148635-149012;OQ419047.1:148637-149014;OQ451898.1:148518-148895;OQ451900.1:149431-149808;OQ581853.1:149457-149834;OQ581855.1:149538-149915;OQ721951.1:149458-149835;OQ721954.1:149431-149808;OR035652.1:149377-149754;OR035664.1:149381-149758;OR035702.1:149378-149755;OR146272.1:149397-149774;OR146330.1:149441-149818;OR264364.1:149452-149829;OR427332.1:149452-149829;OR459777.1:149424-149801;OR459797.1:149450-149827;OR943698.1:151310-151687;PP098589.2:149430-149807;PP496048.1:149434-149811;PP601206.1:151274-151651;PP776553.1:149354-149731;PP852971.1:149422-149799;PP852983.1:149439-149816;PP853000.1:149390-149767;PP853005.1:149398-149775;PP853007.1:149441-149818;PP853013.1:149413-149790;PP853027.1:149446-149823;PP853045.1:149406-149783;PP853048.1:149431-149808;PP860026.1:149435-149812;PP860027.1:149478-149855;PP860028.1:149398-149775;PP860031.1:149460-149837;PP860033.1:149461-149838;PQ153226.1:149428-149805;PQ178862.2:150077-150454;PQ221506.1:145091-145468;PQ221833.1:151267-151644;PQ340477.1:149415-149792;PQ340478.1:149397-149774;PQ340480.1:149475-149852;PQ340483.1:149461-149838;PQ340485.1:149444-149821;PQ340486.1:149462-149839;PQ340487.1:149486-149863;PQ340489.1:149461-149838;PQ340490.1:149413-149790;PQ340491.1:149468-149845;PQ340493.1:149452-149829;PQ340495.1:149478-149855;PQ340496.1:149513-149890;PQ368489.1:149444-149821;PQ451835.1:149518-149895;PQ451836.1:149456-149833;PQ451838.1:149506-149883;PQ451841.1:149469-149846;PQ451842.1:149415-149792 |
| OG0000067 | AY603973.1:156289-157293;AY741551.1:156361-157365;AY753185.1:156544-157548;DQ011154.1:156442-157446;DQ011155.1:156384-157388;EPI_ISL_13331598:154406-155410;EPI_ISL_13363142:154394-155398;EPI_ISL_13374487:154427-155431;EPI_ISL_13502582:154422-155426;EPI_ISL_13508471:154488-155492;EPI_ISL_13607904:154393-155397;EPI_ISL_13624509:154328-155332;EPI_ISL_13889442:154521-155525;EPI_ISL_13953610:154321-155325;EPI_ISL_14181948:154551-155555;EPI_ISL_14838587:156381-157385;EPI_ISL_15263355:154387-155391;EPI_ISL_15269699:154356-155360;EPI_ISL_15293002:154625-155629;EPI_ISL_15641561:154342-155346;EPI_ISL_15831211:154396-155400;EPI_ISL_15992098:154436-155440;EPI_ISL_16233784:154408-155412;EPI_ISL_16946400:154398-155402;EPI_ISL_17048206:154062-155066;EPI_ISL_17703567:154409-155413;EPI_ISL_17718458:154389-155393;EPI_ISL_18213374:154400-155404;EPI_ISL_18360394:153707-154711;EPI_ISL_18557816:154403-155407;EPI_ISL_18642365:154398-155402;EPI_ISL_18659846:154357-155361;EPI_ISL_18744049:154406-155410;EPI_ISL_19022858:154363-155367;EPI_ISL_19028778:154315-155319;EPI_ISL_19151800:154399-155403;EPI_ISL_19159116:154384-155388;EPI_ISL_19350788:155044-156048;EPI_ISL_19359677:154399-155403;EPI_ISL_19446306:154386-155390;EPI_ISL_19467692:156582-157586;JX878411.1:157283-158287;JX878425.1:156771-157775;JX878426.1:156311-157315;KC257460.1:156167-157171;KJ642612.1:154501-155505;KJ642613.1:156050-157054;KJ642616.1:156010-157014;KJ642618.1:153967-154971;KP849469.1:156599-157603;KP849471.1:156534-157538;LC831698.1:154382-155386;MN648051.1:154625-155629;MN702445.1:149918-150922;MN702446.1:156082-157086;MN702447.1:149445-150449;MN702449.1:149956-150960;MN702452.1:156133-157137;MT903338.1:154558-155562;MT903340.1:154411-155415;MT903341.1:145952-146956;MT903342.1:154448-155452;MT903347.1:156354-157358;ON568298.1:154552-155556;ON622713.1:154616-155620;ON622722.2:154404-155408;ON631963.1:154413-155417;ON649879.1:154219-155223;ON676707.1:154450-155454;ON755039.1:154041-155045;ON808413.1:154378-155382;ON880420.1:154614-155618;ON911481.2:154399-155403;ON918656.1:154421-155425;ON959133.1:154510-155514;ON959136.1:154511-155515;ON983168.1:153087-154091;OP019276.1:154398-155402;OP150924.1:154524-155528;OP160532.1:154347-155351;OP235282.1:154398-155402;OP289782.1:154402-155406;OP331335.1:154407-155411;OP382479.1:154391-155395;OP422341.1:154404-155408;OP434519.1:154380-155384;OP535324.1:154411-155415;OP535333.1:154518-155522;OP536770.1:154401-155405;OP597769.1:154435-155439;OP604566.1:154401-155405;OP612682.1:154336-155340;OP612684.1:154387-155391;OP612686.1:154389-155393;OP696841.1:151677-152681;OP879722.1:153624-154628;OQ419007.1:153618-154622;OQ419012.1:153592-154596;OQ419031.1:153596-154600;OQ419047.1:153598-154602;OQ451898.1:153488-154492;OQ451900.1:154402-155406;OQ581853.1:154410-155414;OQ581855.1:154509-155513;OQ721951.1:154428-155432;OQ721954.1:154401-155405;OR035652.1:154330-155334;OR035664.1:154334-155338;OR035702.1:154331-155335;OR146272.1:154350-155354;OR146330.1:154411-155415;OR264364.1:154405-155409;OR427332.1:154405-155409;OR459777.1:154394-155398;OR459797.1:154403-155407;OR943698.1:156287-157291;PP098589.2:154400-155404;PP496048.1:154404-155408;PP601206.1:156342-157346;PP776553.1:154324-155328;PP852971.1:154392-155396;PP852983.1:154409-155413;PP853000.1:154360-155364;PP853005.1:154368-155372;PP853007.1:154411-155415;PP853013.1:154383-155387;PP853027.1:154425-155429;PP853045.1:154376-155380;PP853048.1:154401-155405;PP860026.1:154396-155400;PP860027.1:154433-155437;PP860028.1:154359-155363;PP860031.1:154430-155434;PP860033.1:154416-155420;PQ153226.1:154381-155385;PQ178862.2:155023-156027;PQ221506.1:150131-151135;PQ221833.1:156318-157322;PQ340477.1:154368-155372;PQ340478.1:154350-155354;PQ340480.1:154428-155432;PQ340483.1:154414-155418;PQ340485.1:154397-155401;PQ340486.1:154415-155419;PQ340487.1:154439-155443;PQ340489.1:154414-155418;PQ340490.1:154366-155370;PQ340491.1:154421-155425;PQ340493.1:154405-155409;PQ340495.1:154431-155435;PQ340496.1:154466-155470;PQ368489.1:154414-155418;PQ451835.1:154471-155475;PQ451836.1:154409-155413;PQ451838.1:154459-155463;PQ451841.1:154422-155426;PQ451842.1:154368-155372 |
| OG0000068 | AY603973.1:162481-163392;AY741551.1:162552-163463;AY753185.1:162736-163647;DQ011154.1:160400-161299;DQ011155.1:160342-161241;EPI_ISL_13331598:160616-161527;EPI_ISL_13363142:160604-161515;EPI_ISL_13374487:160637-161548;EPI_ISL_13502582:160632-161543;EPI_ISL_13508471:160698-161609;EPI_ISL_13607904:160603-161514;EPI_ISL_13624509:160538-161449;EPI_ISL_13889442:160731-161642;EPI_ISL_13953610:160531-161442;EPI_ISL_14181948:160761-161672;EPI_ISL_14838587:160339-161238;EPI_ISL_15263355:160597-161508;EPI_ISL_15269699:160566-161477;EPI_ISL_15293002:160835-161746;EPI_ISL_15641561:160552-161463;EPI_ISL_15831211:160606-161517;EPI_ISL_15992098:160646-161557;EPI_ISL_16233784:160618-161529;EPI_ISL_16946400:160608-161519;EPI_ISL_17048206:160272-161183;EPI_ISL_17703567:160619-161530;EPI_ISL_17718458:160599-161510;EPI_ISL_18213374:160610-161521;EPI_ISL_18360394:159917-160828;EPI_ISL_18557816:160613-161524;EPI_ISL_18642365:160608-161519;EPI_ISL_18659846:160567-161478;EPI_ISL_18744049:160616-161527;EPI_ISL_19022858:160573-161484;EPI_ISL_19028778:160525-161436;EPI_ISL_19151800:160609-161520;EPI_ISL_19159116:160594-161505;EPI_ISL_19350788:158997-159896;EPI_ISL_19359677:160609-161520;EPI_ISL_19446306:160596-161507;EPI_ISL_19467692:160540-161439;JX878411.1:161242-162141;JX878425.1:160729-161628;JX878426.1:160269-161168;KC257460.1:160125-161024;KJ642612.1:158459-159358;KJ642613.1:160008-160907;KJ642616.1:162200-163111;KJ642618.1:157925-158824;KP849469.1:160558-161457;KP849471.1:160493-161392;LC831698.1:160592-161503;MN648051.1:160835-161746;MN702445.1:153876-154775;MN702446.1:160040-160939;MN702447.1:153403-154302;MN702449.1:153914-154813;MN702452.1:160091-160990;MT903338.1:160768-161679;MT903340.1:160621-161532;MT903341.1:152162-153073;MT903342.1:160658-161569;MT903347.1:162550-163461;ON568298.1:160762-161673;ON622713.1:160826-161737;ON622722.2:160614-161525;ON631963.1:160623-161534;ON649879.1:160429-161340;ON676707.1:160660-161571;ON755039.1:160251-161162;ON808413.1:160588-161499;ON880420.1:160824-161735;ON911481.2:160609-161520;ON918656.1:160631-161542;ON959133.1:160720-161631;ON959136.1:160721-161632;ON983168.1:159297-160208;OP019276.1:160608-161519;OP150924.1:160734-161645;OP160532.1:160557-161468;OP235282.1:160608-161519;OP289782.1:160612-161523;OP331335.1:160617-161528;OP382479.1:160601-161512;OP422341.1:160614-161525;OP434519.1:160590-161501;OP535324.1:160621-161532;OP535333.1:160728-161639;OP536770.1:160611-161522;OP597769.1:160645-161556;OP604566.1:160611-161522;OP612682.1:160546-161457;OP612684.1:160597-161508;OP612686.1:160599-161510;OP696841.1:157887-158798;OP879722.1:159834-160745;OQ419007.1:159828-160739;OQ419012.1:159802-160713;OQ419031.1:159806-160717;OQ419047.1:159808-160719;OQ451898.1:159698-160609;OQ451900.1:160612-161523;OQ581853.1:160620-161531;OQ581855.1:160719-161630;OQ721951.1:160638-161549;OQ721954.1:160611-161522;OR035652.1:160540-161451;OR035664.1:160544-161455;OR035702.1:160541-161452;OR146272.1:160560-161471;OR146330.1:160621-161532;OR264364.1:160615-161526;OR427332.1:160615-161526;OR459777.1:160604-161515;OR459797.1:160613-161524;OR943698.1:160244-161143;PP098589.2:160610-161521;PP496048.1:160614-161525;PP601206.1:160300-161199;PP776553.1:160534-161445;PP852971.1:160602-161513;PP852983.1:160619-161530;PP853000.1:160570-161481;PP853005.1:160578-161489;PP853007.1:160621-161532;PP853013.1:160593-161504;PP853027.1:160635-161546;PP853045.1:160586-161497;PP853048.1:160611-161522;PP860026.1:160606-161517;PP860027.1:160645-161556;PP860028.1:160569-161480;PP860031.1:160623-161534;PP860033.1:160628-161539;PQ153226.1:160591-161502;PQ178862.2:158976-159875;PQ221506.1:154090-154989;PQ221833.1:160276-161175;PQ340477.1:160578-161489;PQ340478.1:160560-161471;PQ340480.1:160638-161549;PQ340483.1:160624-161535;PQ340485.1:160607-161518;PQ340486.1:160625-161536;PQ340487.1:160649-161560;PQ340489.1:160624-161535;PQ340490.1:160576-161487;PQ340491.1:160631-161542;PQ340493.1:160615-161526;PQ340495.1:160641-161552;PQ340496.1:160676-161587;PQ368489.1:160624-161535;PQ451835.1:160681-161592;PQ451836.1:160619-161530;PQ451838.1:160669-161580;PQ451841.1:160632-161543;PQ451842.1:160578-161489 |
| OG0000069 | AY603973.1:171910-172758;AY741551.1:171920-172768;AY753185.1:172173-173021;DQ011154.1:169938-170786;DQ011155.1:169844-170692;EPI_ISL_13331598:170032-170880;EPI_ISL_13363142:170020-170868;EPI_ISL_13374487:170053-170901;EPI_ISL_13502582:170048-170896;EPI_ISL_13508471:170114-170962;EPI_ISL_13607904:170019-170867;EPI_ISL_13624509:169954-170802;EPI_ISL_13889442:170147-170995;EPI_ISL_13953610:169947-170795;EPI_ISL_14181948:170177-171025;EPI_ISL_14838587:169766-170614;EPI_ISL_15263355:170013-170861;EPI_ISL_15269699:169982-170830;EPI_ISL_15293002:170251-171099;EPI_ISL_15641561:169480-170328;EPI_ISL_15831211:170022-170870;EPI_ISL_15992098:170062-170910;EPI_ISL_16233784:170034-170882;EPI_ISL_16946400:170024-170872;EPI_ISL_17048206:169688-170536;EPI_ISL_17703567:170035-170883;EPI_ISL_17718458:170003-170851;EPI_ISL_18213374:170026-170874;EPI_ISL_18360394:169333-170181;EPI_ISL_18557816:170029-170877;EPI_ISL_18642365:170024-170872;EPI_ISL_18659846:169983-170831;EPI_ISL_18744049:170032-170880;EPI_ISL_19022858:169989-170837;EPI_ISL_19028778:169941-170789;EPI_ISL_19151800:168936-169784;EPI_ISL_19159116:170010-170858;EPI_ISL_19350788:168388-169236;EPI_ISL_19359677:170025-170873;EPI_ISL_19446306:170012-170860;EPI_ISL_19467692:170007-170855;JX878411.1:170780-171628;JX878425.1:170231-171079;JX878426.1:169695-170543;KC257460.1:169593-170441;KJ642612.1:167891-168739;KJ642613.1:169498-170346;KJ642616.1:171617-172465;KJ642618.1:167411-168259;KP849469.1:170126-170974;KP849471.1:169929-170777;LC831698.1:170008-170856;MN648051.1:170251-171099;MN702445.1:163306-164154;MN702446.1:169476-170324;MN702447.1:162822-163670;MN702449.1:163344-164192;MN702452.1:169528-170376;MT903338.1:170184-171032;MT903340.1:170037-170885;MT903341.1:161578-162426;MT903342.1:170074-170922;MT903347.1:171475-172323;ON568298.1:170178-171026;ON622713.1:170242-171090;ON622722.2:170030-170878;ON631963.1:170039-170887;ON649879.1:169845-170693;ON676707.1:170076-170924;ON755039.1:169667-170515;ON808413.1:170004-170852;ON880420.1:170240-171088;ON911481.2:170025-170873;ON918656.1:170047-170895;ON959133.1:170136-170984;ON959136.1:170137-170985;ON983168.1:168713-169561;OP019276.1:170024-170872;OP150924.1:170150-170998;OP160532.1:169973-170821;OP235282.1:170024-170872;OP289782.1:170028-170876;OP331335.1:170033-170881;OP382479.1:170017-170865;OP422341.1:170030-170878;OP434519.1:170006-170854;OP535324.1:170037-170885;OP535333.1:170144-170992;OP536770.1:170027-170875;OP597769.1:170061-170909;OP604566.1:170027-170875;OP612682.1:169962-170810;OP612684.1:170013-170861;OP612686.1:170015-170863;OP696841.1:167303-168151;OP879722.1:169250-170098;OQ419007.1:169244-170092;OQ419012.1:169218-170066;OQ419031.1:169222-170070;OQ419047.1:169224-170072;OQ451898.1:169114-169962;OQ451900.1:170028-170876;OQ581853.1:170036-170884;OQ581855.1:170135-170983;OQ721951.1:170054-170902;OQ721954.1:170027-170875;OR035652.1:169956-170804;OR035664.1:169960-170808;OR035702.1:169957-170805;OR146272.1:169976-170824;OR146330.1:170037-170885;OR264364.1:170031-170879;OR427332.1:170031-170879;OR459777.1:170008-170856;OR459797.1:170029-170877;OR943698.1:169537-170385;PP098589.2:170026-170874;PP496048.1:170030-170878;PP601206.1:169713-170561;PP776553.1:169950-170798;PP852971.1:170014-170862;PP852983.1:170035-170883;PP853000.1:169956-170804;PP853005.1:170420-170842;PP853007.1:170037-170885;PP853013.1:170009-170857;PP853027.1:170051-170899;PP853045.1:169990-170838;PP853048.1:170027-170875;PP860026.1:170022-170870;PP860027.1:170044-170892;PP860028.1:169971-170819;PP860031.1:170015-170863;PP860033.1:170027-170875;PQ153226.1:170007-170855;PQ178862.2:168358-169206;PQ221506.1:163559-164407;PQ221833.1:169694-170542;PQ340477.1:169994-170842;PQ340478.1:169976-170824;PQ340480.1:170054-170902;PQ340483.1:170040-170888;PQ340485.1:170023-170871;PQ340486.1:170041-170889;PQ340487.1:170065-170913;PQ340489.1:170040-170888;PQ340490.1:169992-170840;PQ340491.1:170047-170895;PQ340493.1:170031-170879;PQ340495.1:170057-170905;PQ340496.1:170092-170940;PQ368489.1:170040-170888;PQ451835.1:170097-170945;PQ451836.1:170035-170883;PQ451838.1:170085-170933;PQ451841.1:170048-170896;PQ451842.1:169994-170842 |
| OG0000070 | AY603973.1:172858-173892;AY741551.1:172868-173902;AY753185.1:173121-174155;DQ011154.1:170894-171928;DQ011155.1:170800-171834;EPI_ISL_13331598:170982-172016;EPI_ISL_13363142:170970-172004;EPI_ISL_13374487:171003-172037;EPI_ISL_13502582:170998-172032;EPI_ISL_13508471:171064-172098;EPI_ISL_13607904:170969-172003;EPI_ISL_13624509:170904-171938;EPI_ISL_13889442:171097-172131;EPI_ISL_13953610:170898-171932;EPI_ISL_14181948:171127-172161;EPI_ISL_14838587:170722-171756;EPI_ISL_15263355:170963-171997;EPI_ISL_15269699:170932-171966;EPI_ISL_15293002:171201-172235;EPI_ISL_15641561:170430-171464;EPI_ISL_15831211:170972-172006;EPI_ISL_15992098:171012-172046;EPI_ISL_16233784:170984-172018;EPI_ISL_16946400:170974-172008;EPI_ISL_17048206:170638-171672;EPI_ISL_17703567:170985-172019;EPI_ISL_17718458:170954-171988;EPI_ISL_18213374:170976-172010;EPI_ISL_18360394:170283-171317;EPI_ISL_18557816:170979-172013;EPI_ISL_18642365:170974-172008;EPI_ISL_18659846:170933-171967;EPI_ISL_18744049:170983-172017;EPI_ISL_19022858:170939-171973;EPI_ISL_19028778:170891-171925;EPI_ISL_19151800:169886-170920;EPI_ISL_19159116:170960-171994;EPI_ISL_19350788:169344-170378;EPI_ISL_19359677:170975-172009;EPI_ISL_19446306:170962-171996;EPI_ISL_19467692:170963-171997;JX878411.1:171735-172769;JX878425.1:171187-172221;JX878426.1:170658-171692;KC257460.1:170550-171584;KJ642612.1:168847-169881;KJ642613.1:170455-171489;KJ642616.1:172565-173599;KJ642618.1:168367-169401;KP849469.1:171082-172116;KP849471.1:170885-171919;LC831698.1:170958-171992;MN648051.1:171201-172235;MN702445.1:164262-165296;MN702446.1:170432-171466;MN702447.1:163778-164812;MN702449.1:164300-165334;MN702452.1:170484-171518;MT903338.1:171134-172168;MT903340.1:170987-172021;MT903341.1:162528-163562;MT903342.1:171024-172058;MT903347.1:172424-173458;ON568298.1:171128-172162;ON622713.1:171192-172226;ON622722.2:170980-172014;ON631963.1:170989-172023;ON649879.1:170795-171829;ON676707.1:171026-172060;ON755039.1:170617-171651;ON808413.1:170954-171988;ON880420.1:171190-172224;ON911481.2:170975-172009;ON918656.1:170997-172031;ON959133.1:171086-172120;ON959136.1:171087-172121;ON983168.1:169663-170697;OP019276.1:170974-172008;OP150924.1:171100-172134;OP160532.1:170923-171957;OP235282.1:170974-172008;OP289782.1:170978-172012;OP331335.1:170984-172018;OP382479.1:170967-172001;OP422341.1:170980-172014;OP434519.1:170956-171990;OP535324.1:170987-172021;OP535333.1:171094-172128;OP536770.1:170977-172011;OP597769.1:171011-172045;OP604566.1:170977-172011;OP612682.1:170912-171946;OP612684.1:170963-171997;OP612686.1:170965-171999;OP696841.1:168253-169287;OP879722.1:170200-171234;OQ419007.1:170194-171228;OQ419012.1:170168-171202;OQ419031.1:170172-171206;OQ419047.1:170174-171208;OQ451898.1:170064-171098;OQ451900.1:170978-172012;OQ581853.1:170986-172020;OQ581855.1:171085-172119;OQ721951.1:171004-172038;OQ721954.1:170977-172011;OR035652.1:170906-171940;OR035664.1:170910-171944;OR035702.1:170907-171941;OR146272.1:170926-171960;OR146330.1:170987-172021;OR264364.1:170981-172015;OR427332.1:170981-172015;OR459777.1:170959-171993;OR459797.1:170979-172013;OR943698.1:170493-171527;PP098589.2:170976-172010;PP496048.1:170980-172014;PP601206.1:170670-171704;PP776553.1:170900-171934;PP852971.1:170965-171999;PP852983.1:170985-172019;PP853000.1:170906-171940;PP853005.1:170944-171978;PP853007.1:170987-172021;PP853013.1:170960-171994;PP853027.1:171001-172035;PP853045.1:170941-171975;PP853048.1:170977-172011;PP860026.1:170972-172006;PP860027.1:170994-172028;PP860028.1:170921-171955;PP860031.1:170964-171998;PP860033.1:170977-172011;PQ153226.1:170957-171991;PQ178862.2:169314-170348;PQ221506.1:164522-165556;PQ221833.1:170650-171684;PQ340477.1:170944-171978;PQ340478.1:170926-171960;PQ340480.1:171004-172038;PQ340483.1:170990-172024;PQ340485.1:170973-172007;PQ340486.1:170991-172025;PQ340487.1:171015-172049;PQ340489.1:170990-172024;PQ340490.1:170942-171976;PQ340491.1:170997-172031;PQ340493.1:170981-172015;PQ340495.1:171007-172041;PQ340496.1:171042-172076;PQ368489.1:170990-172024;PQ451835.1:171047-172081;PQ451836.1:170985-172019;PQ451838.1:171035-172069;PQ451841.1:170998-172032;PQ451842.1:170944-171978 |
| OG0000071 | AY603973.1:177478-179841;AY741551.1:177487-179850;AY753185.1:177741-180104;DQ011154.1:175517-177886;DQ011155.1:175439-177808;EPI_ISL_13331598:175652-178015;EPI_ISL_13363142:175690-178053;EPI_ISL_13374487:175747-178110;EPI_ISL_13502582:175739-178102;EPI_ISL_13508471:175736-178099;EPI_ISL_13607904:175642-178005;EPI_ISL_13624509:175572-177935;EPI_ISL_13889442:175831-178194;EPI_ISL_13953610:175566-177929;EPI_ISL_14181948:175799-178162;EPI_ISL_14838587:175345-177708;EPI_ISL_15263355:175537-177900;EPI_ISL_15269699:175604-177967;EPI_ISL_15293002:175875-178238;EPI_ISL_15641561:175098-177461;EPI_ISL_15831211:175710-178073;EPI_ISL_15992098:175716-178079;EPI_ISL_16233784:175656-178019;EPI_ISL_16946400:175634-177997;EPI_ISL_17048206:175342-177705;EPI_ISL_17703567:175657-178020;EPI_ISL_17718458:175616-177979;EPI_ISL_18213374:175644-178007;EPI_ISL_18360394:174920-177283;EPI_ISL_18557816:175663-178026;EPI_ISL_18642365:175650-178013;EPI_ISL_18659846:175605-177968;EPI_ISL_18744049:175645-178008;EPI_ISL_19022858:175607-177970;EPI_ISL_19028778:175559-177922;EPI_ISL_19151800:174570-176933;EPI_ISL_19159116:175628-177991;EPI_ISL_19350788:173989-176352;EPI_ISL_19359677:175643-178006;EPI_ISL_19446306:175630-177993;EPI_ISL_19467692:175586-177955;JX878411.1:176361-178730;JX878425.1:175810-178179;JX878426.1:175281-177650;KC257460.1:175175-177544;KJ642612.1:173471-175846;KJ642613.1:175078-177453;KJ642616.1:177185-179548;KJ642618.1:173001-175376;KP849469.1:175705-178074;KP849471.1:175508-177877;LC831698.1:175626-177989;MN648051.1:175865-178228;MN702445.1:168885-171248;MN702446.1:175055-177418;MN702447.1:168411-170780;MN702449.1:168923-171286;MN702452.1:175107-177470;MT903338.1:175838-178201;MT903340.1:175655-178018;MT903341.1:167218-169581;MT903342.1:175758-178121;MT903347.1:177049-179412;ON568298.1:175800-178163;ON622713.1:175872-178235;ON622722.2:175660-178023;ON631963.1:175659-178022;ON649879.1:175465-177828;ON676707.1:175730-178093;ON755039.1:175281-177644;ON808413.1:175628-177991;ON880420.1:175862-178225;ON911481.2:175652-178015;ON918656.1:175669-178032;ON959133.1:175760-178123;ON959136.1:175755-178118;ON983168.1:174335-176698;OP019276.1:175642-178005;OP150924.1:175776-178139;OP160532.1:175591-177954;OP235282.1:175642-178005;OP289782.1:175646-178009;OP331335.1:175656-178019;OP382479.1:175637-178000;OP422341.1:175652-178015;OP434519.1:175624-177987;OP535324.1:175682-178045;OP535333.1:175764-178127;OP536770.1:175645-178008;OP597769.1:175725-178088;OP604566.1:175651-178014;OP612682.1:175552-177915;OP612684.1:175603-177966;OP612686.1:175605-177968;OP696841.1:172937-175300;OP879722.1:174904-177267;OQ419007.1:174862-177225;OQ419012.1:174836-177199;OQ419031.1:174840-177203;OQ419047.1:174842-177205;OQ451898.1:174748-177111;OQ451900.1:175662-178025;OQ581853.1:175660-178023;OQ581855.1:175769-178132;OQ721951.1:175648-178011;OQ721954.1:175681-178044;OR035652.1:175578-177941;OR035664.1:175582-177945;OR035702.1:175581-177944;OR146272.1:175598-177961;OR146330.1:175731-178094;OR264364.1:175653-178016;OR427332.1:175653-178016;OR459777.1:175621-177984;OR459797.1:175651-178014;OR943698.1:175099-177474;PP098589.2:175644-178007;PP496048.1:175660-178023;PP601206.1:175293-177662;PP776553.1:175586-177949;PP852971.1:175633-177996;PP852983.1:175657-178020;PP853000.1:175596-177959;PP853005.1:175616-177979;PP853007.1:175653-178016;PP853013.1:175650-178013;PP853027.1:175691-178054;PP853045.1:175456-177819;PP853048.1:175651-178014;PP860026.1:175652-178015;PP860027.1:175635-177998;PP860028.1:175589-177952;PP860031.1:175631-177994;PP860033.1:175628-177991;PQ153226.1:175625-177988;PQ178862.2:173965-176328;PQ221506.1:169145-171520;PQ221833.1:175276-177639;PQ340477.1:175616-177979;PQ340478.1:175606-177969;PQ340480.1:175674-178037;PQ340483.1:175666-178029;PQ340485.1:175651-178014;PQ340486.1:175665-178028;PQ340487.1:175691-178054;PQ340489.1:175668-178031;PQ340490.1:175616-177979;PQ340491.1:175679-178042;PQ340493.1:175651-178014;PQ340495.1:175685-178048;PQ340496.1:175720-178083;PQ368489.1:175658-178021;PQ451835.1:175729-178092;PQ451836.1:175669-178032;PQ451838.1:175721-178084;PQ451841.1:175686-178049;PQ451842.1:175622-177985 |
| OG0000072 | AY603973.1:30020-30469;AY741551.1:30034-30483;AY753185.1:30263-30712;DQ011154.1:30269-30718;DQ011155.1:30255-30704;EPI_ISL_13331598:28106-28555;EPI_ISL_13363142:28099-28548;EPI_ISL_13374487:28114-28563;EPI_ISL_13502582:28099-28548;EPI_ISL_13508471:28191-28640;EPI_ISL_13607904:28090-28539;EPI_ISL_13624509:28034-28483;EPI_ISL_13889442:28209-28658;EPI_ISL_13953610:28057-28506;EPI_ISL_14181948:28235-28684;EPI_ISL_14838587:30123-30572;EPI_ISL_15263355:28090-28539;EPI_ISL_15269699:28059-28508;EPI_ISL_15293002:28332-28781;EPI_ISL_15641561:28048-28497;EPI_ISL_15831211:28097-28546;EPI_ISL_15992098:28112-28561;EPI_ISL_16233784:28108-28557;EPI_ISL_16946400:28097-28546;EPI_ISL_17048206:27747-28196;EPI_ISL_17703567:28112-28561;EPI_ISL_17718458:28090-28539;EPI_ISL_18213374:28087-28536;EPI_ISL_18360394:27748-28197;EPI_ISL_18557816:28088-28537;EPI_ISL_18642365:28090-28539;EPI_ISL_18659846:28060-28509;EPI_ISL_18744049:28092-28541;EPI_ISL_19022858:28087-28536;EPI_ISL_19028778:28004-28453;EPI_ISL_19151800:28088-28537;EPI_ISL_19159116:28090-28539;EPI_ISL_19350788:29008-29457;EPI_ISL_19359677:28087-28536;EPI_ISL_19446306:28082-28531;EPI_ISL_19467692:30402-30851;JX878411.1:31117-31566;JX878425.1:30631-31080;JX878426.1:30071-30520;KC257460.1:30052-30501;KJ642612.1:28357-28806;KJ642613.1:29938-30387;KJ642616.1:29719-30168;KJ642618.1:27525-27974;KP849469.1:30439-30888;KP849471.1:30360-30809;LC831698.1:28088-28537;MN648051.1:28310-28759;MN702445.1:23784-24233;MN702446.1:29930-30379;MN702447.1:23295-23744;MN702449.1:23795-24244;MN702452.1:29968-30417;MT903338.1:28253-28702;MT903340.1:28090-28539;MT903341.1:19623-20072;MT903342.1:28105-28554;MT903347.1:30018-30467;ON568298.1:28236-28685;ON622713.1:28322-28771;ON622722.2:28093-28542;ON631963.1:28113-28562;ON649879.1:27923-28372;ON676707.1:28143-28592;ON755039.1:27753-28202;ON808413.1:28093-28542;ON880420.1:28320-28769;ON911481.2:28110-28559;ON918656.1:28125-28574;ON959133.1:28214-28663;ON959136.1:28215-28664;ON983168.1:26789-27238;OP019276.1:28087-28536;OP150924.1:28228-28677;OP160532.1:28053-28502;OP235282.1:28087-28536;OP289782.1:28090-28539;OP331335.1:28107-28556;OP382479.1:28094-28543;OP422341.1:28107-28556;OP434519.1:28086-28535;OP535324.1:28088-28537;OP535333.1:28197-28646;OP536770.1:28089-28538;OP597769.1:28111-28560;OP604566.1:28090-28539;OP612682.1:28016-28465;OP612684.1:28080-28529;OP612686.1:28080-28529;OP696841.1:25364-25813;OP879722.1:27313-27762;OQ419007.1:27323-27772;OQ419012.1:27315-27764;OQ419031.1:27294-27743;OQ419047.1:27323-27772;OQ451898.1:27175-27624;OQ451900.1:28088-28537;OQ581853.1:28114-28563;OQ581855.1:28195-28644;OQ721951.1:28128-28577;OQ721954.1:28085-28534;OR035652.1:28037-28486;OR035664.1:28037-28486;OR035702.1:28037-28486;OR146272.1:28053-28502;OR146330.1:28098-28547;OR264364.1:28108-28557;OR427332.1:28108-28557;OR459777.1:28092-28541;OR459797.1:28107-28556;OR943698.1:30099-30548;PP098589.2:28089-28538;PP496048.1:28093-28542;PP601206.1:30121-30570;PP776553.1:28014-28463;PP852971.1:28087-28536;PP852983.1:28092-28541;PP853000.1:28054-28503;PP853005.1:28062-28511;PP853007.1:28094-28543;PP853013.1:28068-28517;PP853027.1:28099-28548;PP853045.1:28080-28529;PP853048.1:28099-28548;PP860026.1:28094-28552;PP860027.1:28105-28554;PP860028.1:28061-28510;PP860031.1:28115-28564;PP860033.1:28104-28553;PQ153226.1:28087-28536;PQ178862.2:28985-29434;PQ221506.1:23941-24390;PQ221833.1:30113-30562;PQ340477.1:28070-28519;PQ340478.1:28055-28504;PQ340480.1:28134-28583;PQ340483.1:28118-28567;PQ340485.1:28104-28553;PQ340486.1:28119-28568;PQ340487.1:28144-28593;PQ340489.1:28119-28568;PQ340490.1:28070-28519;PQ340491.1:28128-28577;PQ340493.1:28104-28553;PQ340495.1:28134-28583;PQ340496.1:28167-28616;PQ368489.1:28090-28539;PQ451835.1:28178-28627;PQ451836.1:28110-28559;PQ451838.1:28161-28610;PQ451841.1:28121-28570;PQ451842.1:28073-28522 |
| OG0000073 | AY603973.1:37919-38176;AY741551.1:37923-38180;AY753185.1:38162-38419;DQ011154.1:38139-38396;DQ011155.1:38120-38377;EPI_ISL_13331598:35990-36247;EPI_ISL_13363142:35983-36240;EPI_ISL_13374487:35998-36255;EPI_ISL_13502582:35983-36240;EPI_ISL_13508471:36075-36332;EPI_ISL_13607904:35974-36231;EPI_ISL_13624509:35918-36175;EPI_ISL_13889442:36093-36350;EPI_ISL_13953610:35941-36198;EPI_ISL_14181948:36119-36376;EPI_ISL_14838587:37993-38250;EPI_ISL_15263355:35974-36231;EPI_ISL_15269699:35943-36200;EPI_ISL_15293002:36216-36473;EPI_ISL_15641561:35932-36189;EPI_ISL_15831211:35981-36238;EPI_ISL_15992098:35996-36253;EPI_ISL_16233784:35992-36249;EPI_ISL_16946400:35981-36238;EPI_ISL_17048206:35631-35888;EPI_ISL_17703567:35996-36253;EPI_ISL_17718458:35974-36231;EPI_ISL_18213374:35971-36228;EPI_ISL_18360394:35632-35889;EPI_ISL_18557816:35972-36229;EPI_ISL_18642365:35974-36231;EPI_ISL_18659846:35944-36201;EPI_ISL_18744049:35976-36233;EPI_ISL_19022858:35971-36228;EPI_ISL_19028778:35888-36145;EPI_ISL_19151800:35972-36229;EPI_ISL_19159116:35974-36231;EPI_ISL_19350788:36867-37124;EPI_ISL_19359677:35971-36228;EPI_ISL_19446306:35966-36223;EPI_ISL_19467692:38272-38529;JX878411.1:38987-39244;JX878425.1:38501-38758;JX878426.1:37946-38203;KC257460.1:37917-38174;KJ642612.1:36232-36489;KJ642613.1:37808-38065;KJ642616.1:37613-37870;KJ642618.1:35400-35657;KP849469.1:38309-38566;KP849471.1:38235-38492;LC831698.1:35972-36229;MN648051.1:36194-36451;MN702445.1:31654-31911;MN702446.1:37800-38057;MN702447.1:31165-31422;MN702449.1:31665-31922;MN702452.1:37838-38095;MT903338.1:36137-36394;MT903340.1:35974-36231;MT903341.1:27507-27764;MT903342.1:35989-36246;MT903347.1:37932-38189;ON568298.1:36120-36377;ON622713.1:36206-36463;ON622722.2:35977-36234;ON631963.1:35997-36254;ON649879.1:35807-36064;ON676707.1:36027-36284;ON755039.1:35637-35894;ON808413.1:35977-36234;ON880420.1:36204-36461;ON911481.2:35994-36251;ON918656.1:36009-36266;ON959133.1:36098-36355;ON959136.1:36099-36356;ON983168.1:34673-34930;OP019276.1:35971-36228;OP150924.1:36112-36369;OP160532.1:35937-36194;OP235282.1:35971-36228;OP289782.1:35974-36231;OP331335.1:35991-36248;OP382479.1:35978-36235;OP422341.1:35991-36248;OP434519.1:35970-36227;OP535324.1:35972-36229;OP535333.1:36081-36338;OP536770.1:35973-36230;OP597769.1:35995-36252;OP604566.1:35974-36231;OP612682.1:35900-36157;OP612684.1:35964-36221;OP612686.1:35964-36221;OP696841.1:33248-33505;OP879722.1:35197-35454;OQ419007.1:35207-35464;OQ419012.1:35199-35456;OQ419031.1:35178-35435;OQ419047.1:35207-35464;OQ451898.1:35059-35316;OQ451900.1:35972-36229;OQ581853.1:35998-36255;OQ581855.1:36079-36336;OQ721951.1:36012-36269;OQ721954.1:35969-36226;OR035652.1:35921-36178;OR035664.1:35921-36178;OR035702.1:35921-36178;OR146272.1:35937-36194;OR146330.1:35982-36239;OR264364.1:35992-36249;OR427332.1:35992-36249;OR459777.1:35976-36233;OR459797.1:35991-36248;OR943698.1:37974-38231;PP098589.2:35973-36230;PP496048.1:35977-36234;PP601206.1:37991-38248;PP776553.1:35898-36155;PP852971.1:35971-36228;PP852983.1:35977-36234;PP853000.1:35938-36195;PP853005.1:35946-36203;PP853007.1:35979-36236;PP853013.1:35952-36209;PP853027.1:35983-36240;PP853045.1:35964-36221;PP853048.1:35983-36240;PP860026.1:35981-36238;PP860027.1:36006-36263;PP860028.1:35947-36204;PP860031.1:36000-36257;PP860033.1:36005-36262;PQ153226.1:35971-36228;PQ178862.2:36844-37101;PQ221506.1:31814-32071;PQ221833.1:37983-38240;PQ340477.1:35954-36211;PQ340478.1:35939-36196;PQ340480.1:36018-36275;PQ340483.1:36002-36259;PQ340485.1:35988-36245;PQ340486.1:36003-36260;PQ340487.1:36028-36285;PQ340489.1:36003-36260;PQ340490.1:35954-36211;PQ340491.1:36012-36269;PQ340493.1:35988-36245;PQ340495.1:36018-36275;PQ340496.1:36051-36308;PQ368489.1:35974-36231;PQ451835.1:36062-36319;PQ451836.1:35994-36251;PQ451838.1:36045-36302;PQ451841.1:36005-36262;PQ451842.1:35957-36214 |
| OG0000074 | AY603973.1:56414-56701;AY741551.1:56418-56705;AY753185.1:56657-56944;DQ011154.1:56875-57162;DQ011155.1:56853-57140;EPI_ISL_13331598:54485-54772;EPI_ISL_13363142:54478-54765;EPI_ISL_13374487:54493-54780;EPI_ISL_13502582:54478-54765;EPI_ISL_13508471:54570-54857;EPI_ISL_13607904:54469-54756;EPI_ISL_13624509:54413-54700;EPI_ISL_13889442:54588-54875;EPI_ISL_13953610:54405-54692;EPI_ISL_14181948:54614-54901;EPI_ISL_14838587:56729-57016;EPI_ISL_15263355:54469-54756;EPI_ISL_15269699:54438-54725;EPI_ISL_15293002:54711-54998;EPI_ISL_15641561:54427-54714;EPI_ISL_15831211:54476-54763;EPI_ISL_15992098:54491-54778;EPI_ISL_16233784:54487-54774;EPI_ISL_16946400:54476-54763;EPI_ISL_17048206:54126-54413;EPI_ISL_17703567:54491-54778;EPI_ISL_17718458:54469-54756;EPI_ISL_18213374:54466-54753;EPI_ISL_18360394:54127-54414;EPI_ISL_18557816:54467-54754;EPI_ISL_18642365:54469-54756;EPI_ISL_18659846:54439-54726;EPI_ISL_18744049:54471-54758;EPI_ISL_19022858:54466-54753;EPI_ISL_19028778:54383-54670;EPI_ISL_19151800:54467-54754;EPI_ISL_19159116:54469-54756;EPI_ISL_19350788:55601-55888;EPI_ISL_19359677:54466-54753;EPI_ISL_19446306:54461-54748;EPI_ISL_19467692:57012-57299;JX878411.1:57720-58007;JX878425.1:57237-57524;JX878426.1:56686-56973;KC257460.1:56651-56938;KJ642612.1:54972-55259;KJ642613.1:56531-56818;KJ642616.1:56108-56395;KJ642618.1:54139-54426;KP849469.1:57045-57332;KP849471.1:56970-57257;LC831698.1:54467-54754;MN648051.1:54689-54976;MN702445.1:50388-50675;MN702446.1:56533-56820;MN702447.1:49901-50188;MN702449.1:50399-50686;MN702452.1:56574-56861;MT903338.1:54632-54919;MT903340.1:54469-54756;MT903341.1:46002-46289;MT903342.1:54484-54771;MT903347.1:56425-56712;ON568298.1:54615-54902;ON622713.1:54701-54988;ON622722.2:54472-54759;ON631963.1:54492-54779;ON649879.1:54302-54589;ON676707.1:54522-54809;ON755039.1:54132-54419;ON808413.1:54472-54759;ON880420.1:54699-54986;ON911481.2:54489-54776;ON918656.1:54504-54791;ON959133.1:54593-54880;ON959136.1:54594-54881;ON983168.1:53168-53455;OP019276.1:54466-54753;OP150924.1:54607-54894;OP160532.1:54432-54719;OP235282.1:54466-54753;OP289782.1:54469-54756;OP331335.1:54486-54773;OP382479.1:54473-54760;OP422341.1:54486-54773;OP434519.1:54465-54752;OP535324.1:54467-54754;OP535333.1:54576-54863;OP536770.1:54468-54755;OP597769.1:54490-54777;OP604566.1:54469-54756;OP612682.1:54395-54682;OP612684.1:54459-54746;OP612686.1:54459-54746;OP696841.1:51743-52030;OP879722.1:53692-53979;OQ419007.1:53702-53989;OQ419012.1:53669-53956;OQ419031.1:53673-53960;OQ419047.1:53702-53989;OQ451898.1:53554-53841;OQ451900.1:54467-54754;OQ581853.1:54493-54780;OQ581855.1:54574-54861;OQ721951.1:54507-54794;OQ721954.1:54464-54751;OR035652.1:54416-54703;OR035664.1:54416-54703;OR035702.1:54416-54703;OR146272.1:54432-54719;OR146330.1:54477-54764;OR264364.1:54487-54774;OR427332.1:54487-54774;OR459777.1:54471-54758;OR459797.1:54486-54773;OR943698.1:56715-57002;PP098589.2:54468-54755;PP496048.1:54472-54759;PP601206.1:56727-57014;PP776553.1:54393-54680;PP852971.1:54466-54753;PP852983.1:54472-54759;PP853000.1:54433-54720;PP853005.1:54441-54728;PP853007.1:54474-54761;PP853013.1:54447-54734;PP853027.1:54478-54765;PP853045.1:54459-54746;PP853048.1:54478-54765;PP860026.1:54476-54763;PP860027.1:54501-54788;PP860028.1:54442-54729;PP860031.1:54495-54782;PP860033.1:54500-54787;PQ153226.1:54466-54753;PQ178862.2:55578-55865;PQ221506.1:50554-50841;PQ221833.1:56720-57007;PQ340477.1:54449-54736;PQ340478.1:54434-54721;PQ340480.1:54513-54800;PQ340483.1:54497-54784;PQ340485.1:54483-54770;PQ340486.1:54498-54785;PQ340487.1:54523-54810;PQ340489.1:54498-54785;PQ340490.1:54449-54736;PQ340491.1:54504-54791;PQ340493.1:54483-54770;PQ340495.1:54513-54800;PQ340496.1:54546-54833;PQ368489.1:54469-54756;PQ451835.1:54554-54841;PQ451836.1:54486-54773;PQ451838.1:54537-54824;PQ451841.1:54497-54784;PQ451842.1:54449-54736 |
| OG0000075 | AY603973.1:70802-71464;AY741551.1:70806-71468;AY753185.1:71045-71707;DQ011154.1:71263-71925;DQ011155.1:71241-71903;EPI_ISL_13331598:68872-69534;EPI_ISL_13363142:68865-69527;EPI_ISL_13374487:68880-69542;EPI_ISL_13502582:68865-69527;EPI_ISL_13508471:68957-69619;EPI_ISL_13607904:68856-69518;EPI_ISL_13624509:68800-69462;EPI_ISL_13889442:68975-69637;EPI_ISL_13953610:68792-69454;EPI_ISL_14181948:69001-69663;EPI_ISL_14838587:71117-71779;EPI_ISL_15263355:68856-69518;EPI_ISL_15269699:68825-69487;EPI_ISL_15293002:69098-69760;EPI_ISL_15641561:68814-69476;EPI_ISL_15831211:68863-69525;EPI_ISL_15992098:68878-69540;EPI_ISL_16233784:68874-69536;EPI_ISL_16946400:68863-69525;EPI_ISL_17048206:68513-69175;EPI_ISL_17703567:68878-69540;EPI_ISL_17718458:68856-69518;EPI_ISL_18213374:68853-69515;EPI_ISL_18360394:68514-69176;EPI_ISL_18557816:68854-69516;EPI_ISL_18642365:68856-69518;EPI_ISL_18659846:68826-69488;EPI_ISL_18744049:68858-69520;EPI_ISL_19022858:68853-69515;EPI_ISL_19028778:68770-69432;EPI_ISL_19151800:68854-69516;EPI_ISL_19159116:68856-69518;EPI_ISL_19350788:69989-70651;EPI_ISL_19359677:68853-69515;EPI_ISL_19446306:68848-69510;EPI_ISL_19467692:71400-72062;JX878411.1:72108-72770;JX878425.1:71625-72287;JX878426.1:71074-71736;KC257460.1:71039-71701;KJ642612.1:69360-70022;KJ642613.1:70919-71581;KJ642616.1:70496-71158;KJ642618.1:68527-69189;KP849469.1:71433-72095;KP849471.1:71358-72020;LC831698.1:68854-69516;MN648051.1:69076-69738;MN702445.1:64776-65438;MN702446.1:70921-71583;MN702447.1:64289-64951;MN702449.1:64787-65449;MN702452.1:70962-71624;MT903338.1:69019-69681;MT903340.1:68856-69518;MT903341.1:60389-61051;MT903342.1:68871-69533;MT903347.1:70813-71475;ON568298.1:69002-69664;ON622713.1:69088-69750;ON622722.2:68859-69521;ON631963.1:68879-69541;ON649879.1:68689-69351;ON676707.1:68909-69571;ON755039.1:68519-69181;ON808413.1:68859-69521;ON880420.1:69086-69748;ON911481.2:68876-69538;ON918656.1:68891-69553;ON959133.1:68980-69642;ON959136.1:68981-69643;ON983168.1:67555-68217;OP019276.1:68853-69515;OP150924.1:68994-69656;OP160532.1:68819-69481;OP235282.1:68853-69515;OP289782.1:68856-69518;OP331335.1:68873-69535;OP382479.1:68860-69522;OP422341.1:68873-69535;OP434519.1:68852-69514;OP535324.1:68854-69516;OP535333.1:68963-69625;OP536770.1:68855-69517;OP597769.1:68877-69539;OP604566.1:68856-69518;OP612682.1:68782-69444;OP612684.1:68846-69508;OP612686.1:68846-69508;OP696841.1:66130-66792;OP879722.1:68079-68741;OQ419007.1:68089-68751;OQ419012.1:68056-68718;OQ419031.1:68060-68722;OQ419047.1:68062-68724;OQ451898.1:67941-68603;OQ451900.1:68854-69516;OQ581853.1:68880-69542;OQ581855.1:68961-69623;OQ721951.1:68894-69556;OQ721954.1:68851-69513;OR035652.1:68803-69465;OR035664.1:68803-69465;OR035702.1:68803-69465;OR146272.1:68819-69481;OR146330.1:68864-69526;OR264364.1:68874-69536;OR427332.1:68874-69536;OR459777.1:68858-69520;OR459797.1:68873-69535;OR943698.1:71103-71765;PP098589.2:68855-69517;PP496048.1:68859-69521;PP601206.1:71115-71777;PP776553.1:68780-69442;PP852971.1:68853-69515;PP852983.1:68859-69521;PP853000.1:68820-69482;PP853005.1:68828-69490;PP853007.1:68861-69523;PP853013.1:68834-69496;PP853027.1:68865-69527;PP853045.1:68846-69508;PP853048.1:68865-69527;PP860026.1:68863-69525;PP860027.1:68888-69550;PP860028.1:68828-69490;PP860031.1:68882-69544;PP860033.1:68887-69549;PQ153226.1:68853-69515;PQ178862.2:69966-70628;PQ221506.1:64942-65604;PQ221833.1:71108-71770;PQ340477.1:68836-69498;PQ340478.1:68821-69483;PQ340480.1:68900-69562;PQ340483.1:68884-69546;PQ340485.1:68870-69532;PQ340486.1:68885-69547;PQ340487.1:68910-69572;PQ340489.1:68885-69547;PQ340490.1:68836-69498;PQ340491.1:68891-69553;PQ340493.1:68870-69532;PQ340495.1:68900-69562;PQ340496.1:68933-69595;PQ368489.1:68856-69518;PQ451835.1:68941-69603;PQ451836.1:68873-69535;PQ451838.1:68924-69586;PQ451841.1:68884-69546;PQ451842.1:68836-69498 |
| OG0000076 | AY603973.1:73124-73315;AY741551.1:73128-73319;AY753185.1:73367-73558;DQ011154.1:73585-73776;DQ011155.1:73563-73754;EPI_ISL_13331598:71194-71385;EPI_ISL_13363142:71187-71378;EPI_ISL_13374487:71202-71393;EPI_ISL_13502582:71187-71378;EPI_ISL_13508471:71279-71470;EPI_ISL_13607904:71178-71369;EPI_ISL_13624509:71122-71313;EPI_ISL_13889442:71297-71488;EPI_ISL_13953610:71114-71305;EPI_ISL_14181948:71323-71514;EPI_ISL_14838587:73439-73630;EPI_ISL_15263355:71178-71369;EPI_ISL_15269699:71147-71338;EPI_ISL_15293002:71420-71611;EPI_ISL_15641561:71136-71327;EPI_ISL_15831211:71185-71376;EPI_ISL_15992098:71200-71391;EPI_ISL_16233784:71196-71387;EPI_ISL_16946400:71185-71376;EPI_ISL_17048206:70835-71026;EPI_ISL_17703567:71200-71391;EPI_ISL_17718458:71178-71369;EPI_ISL_18213374:71175-71366;EPI_ISL_18360394:70836-71027;EPI_ISL_18557816:71176-71367;EPI_ISL_18642365:71178-71369;EPI_ISL_18659846:71148-71339;EPI_ISL_18744049:71180-71371;EPI_ISL_19022858:71175-71366;EPI_ISL_19028778:71092-71283;EPI_ISL_19151800:71176-71367;EPI_ISL_19159116:71178-71369;EPI_ISL_19350788:72311-72502;EPI_ISL_19359677:71175-71366;EPI_ISL_19446306:71170-71361;EPI_ISL_19467692:73722-73913;JX878411.1:74430-74621;JX878425.1:73947-74138;JX878426.1:73396-73587;KC257460.1:73361-73552;KJ642612.1:71682-71873;KJ642613.1:73241-73432;KJ642616.1:72818-73009;KJ642618.1:70849-71040;KP849469.1:73755-73946;KP849471.1:73681-73872;LC831698.1:71176-71367;MN648051.1:71398-71589;MN702445.1:67098-67289;MN702446.1:73243-73434;MN702447.1:66611-66802;MN702449.1:67109-67300;MN702452.1:73284-73475;MT903338.1:71341-71532;MT903340.1:71178-71369;MT903341.1:62711-62902;MT903342.1:71193-71384;MT903347.1:73135-73326;ON568298.1:71324-71515;ON622713.1:71410-71601;ON622722.2:71181-71372;ON631963.1:71201-71392;ON649879.1:71011-71202;ON676707.1:71231-71422;ON755039.1:70841-71032;ON808413.1:71181-71372;ON880420.1:71408-71599;ON911481.2:71198-71389;ON918656.1:71213-71404;ON959133.1:71302-71493;ON959136.1:71303-71494;ON983168.1:69877-70068;OP019276.1:71175-71366;OP150924.1:71316-71507;OP160532.1:71141-71332;OP235282.1:71175-71366;OP289782.1:71178-71369;OP331335.1:71195-71386;OP382479.1:71182-71373;OP422341.1:71195-71386;OP434519.1:71174-71365;OP535324.1:71176-71367;OP535333.1:71285-71476;OP536770.1:71177-71368;OP597769.1:71199-71390;OP604566.1:71178-71369;OP612682.1:71104-71295;OP612684.1:71168-71359;OP612686.1:71168-71359;OP696841.1:68452-68643;OP879722.1:70401-70592;OQ419007.1:70411-70602;OQ419012.1:70378-70569;OQ419031.1:70382-70573;OQ419047.1:70384-70575;OQ451898.1:70263-70454;OQ451900.1:71176-71367;OQ581853.1:71202-71393;OQ581855.1:71283-71474;OQ721951.1:71216-71407;OQ721954.1:71173-71364;OR035652.1:71125-71316;OR035664.1:71125-71316;OR035702.1:71125-71316;OR146272.1:71141-71332;OR146330.1:71186-71377;OR264364.1:71196-71387;OR427332.1:71196-71387;OR459777.1:71180-71371;OR459797.1:71195-71386;OR943698.1:73425-73616;PP098589.2:71177-71368;PP496048.1:71181-71372;PP601206.1:73437-73628;PP776553.1:71102-71293;PP852971.1:71175-71366;PP852983.1:71181-71372;PP853000.1:71142-71333;PP853005.1:71150-71341;PP853007.1:71183-71374;PP853013.1:71156-71347;PP853027.1:71187-71378;PP853045.1:71168-71359;PP853048.1:71187-71378;PP860026.1:71185-71376;PP860027.1:71210-71401;PP860028.1:71150-71341;PP860031.1:71204-71395;PP860033.1:71209-71400;PQ153226.1:71175-71366;PQ178862.2:72288-72479;PQ221506.1:67264-67455;PQ221833.1:73430-73621;PQ340477.1:71158-71349;PQ340478.1:71143-71334;PQ340480.1:71222-71413;PQ340483.1:71206-71397;PQ340485.1:71192-71383;PQ340486.1:71207-71398;PQ340487.1:71232-71423;PQ340489.1:71207-71398;PQ340490.1:71158-71349;PQ340491.1:71213-71404;PQ340493.1:71192-71383;PQ340495.1:71222-71413;PQ340496.1:71255-71446;PQ368489.1:71178-71369;PQ451835.1:71263-71454;PQ451836.1:71195-71386;PQ451838.1:71246-71437;PQ451841.1:71206-71397;PQ451842.1:71158-71349 |
| OG0000077 | AY603973.1:75725-76747;AY741551.1:75729-76751;AY753185.1:75968-76990;DQ011154.1:76186-77208;DQ011155.1:76167-77189;EPI_ISL_13331598:73795-74817;EPI_ISL_13363142:73788-74810;EPI_ISL_13374487:73803-74825;EPI_ISL_13502582:73788-74810;EPI_ISL_13508471:73880-74902;EPI_ISL_13607904:73779-74801;EPI_ISL_13624509:73723-74745;EPI_ISL_13889442:73898-74920;EPI_ISL_13953610:73715-74737;EPI_ISL_14181948:73924-74946;EPI_ISL_14838587:76040-77062;EPI_ISL_15263355:73779-74801;EPI_ISL_15269699:73748-74770;EPI_ISL_15293002:74021-75043;EPI_ISL_15641561:73737-74759;EPI_ISL_15831211:73786-74808;EPI_ISL_15992098:73801-74823;EPI_ISL_16233784:73797-74819;EPI_ISL_16946400:73786-74808;EPI_ISL_17048206:73436-74458;EPI_ISL_17703567:73801-74823;EPI_ISL_17718458:73779-74801;EPI_ISL_18213374:73776-74798;EPI_ISL_18360394:73437-74459;EPI_ISL_18557816:73777-74799;EPI_ISL_18642365:73779-74801;EPI_ISL_18659846:73749-74771;EPI_ISL_18744049:73781-74803;EPI_ISL_19022858:73776-74798;EPI_ISL_19028778:73693-74715;EPI_ISL_19151800:73777-74799;EPI_ISL_19159116:73779-74801;EPI_ISL_19350788:74912-75934;EPI_ISL_19359677:73776-74798;EPI_ISL_19446306:73771-74793;EPI_ISL_19467692:76323-77345;JX878411.1:77031-78053;JX878425.1:76548-77570;JX878426.1:75997-77019;KC257460.1:75965-76987;KJ642612.1:74283-75305;KJ642613.1:75842-76864;KJ642616.1:75419-76441;KJ642618.1:73450-74472;KP849469.1:76356-77378;KP849471.1:76282-77304;LC831698.1:73777-74799;MN648051.1:73999-75021;MN702445.1:69699-70721;MN702446.1:75844-76866;MN702447.1:69212-70234;MN702449.1:69710-70732;MN702452.1:75885-76907;MT903338.1:73942-74964;MT903340.1:73779-74801;MT903341.1:65312-66334;MT903342.1:73794-74816;MT903347.1:75736-76758;ON568298.1:73925-74947;ON622713.1:74011-75033;ON622722.2:73782-74804;ON631963.1:73802-74824;ON649879.1:73612-74634;ON676707.1:73832-74854;ON755039.1:73442-74464;ON808413.1:73782-74804;ON880420.1:74009-75031;ON911481.2:73799-74821;ON918656.1:73814-74836;ON959133.1:73903-74925;ON959136.1:73904-74926;ON983168.1:72478-73500;OP019276.1:73776-74798;OP150924.1:73917-74939;OP160532.1:73742-74764;OP235282.1:73776-74798;OP289782.1:73779-74801;OP331335.1:73796-74818;OP382479.1:73783-74805;OP422341.1:73796-74818;OP434519.1:73775-74797;OP535324.1:73777-74799;OP535333.1:73886-74908;OP536770.1:73778-74800;OP597769.1:73800-74822;OP604566.1:73779-74801;OP612682.1:73705-74727;OP612684.1:73769-74791;OP612686.1:73769-74791;OP696841.1:71053-72075;OP879722.1:73002-74024;OQ419007.1:73012-74034;OQ419012.1:72979-74001;OQ419031.1:72983-74005;OQ419047.1:72985-74007;OQ451898.1:72864-73886;OQ451900.1:73777-74799;OQ581853.1:73803-74825;OQ581855.1:73884-74906;OQ721951.1:73817-74839;OQ721954.1:73774-74796;OR035652.1:73726-74748;OR035664.1:73726-74748;OR035702.1:73726-74748;OR146272.1:73742-74764;OR146330.1:73787-74809;OR264364.1:73797-74819;OR427332.1:73797-74819;OR459777.1:73781-74803;OR459797.1:73796-74818;OR943698.1:76026-77048;PP098589.2:73778-74800;PP496048.1:73782-74804;PP601206.1:76038-77060;PP776553.1:73703-74725;PP852971.1:73776-74798;PP852983.1:73782-74804;PP853000.1:73743-74765;PP853005.1:73751-74773;PP853007.1:73784-74806;PP853013.1:73757-74779;PP853027.1:73788-74810;PP853045.1:73769-74791;PP853048.1:73788-74810;PP860026.1:73786-74808;PP860027.1:73811-74833;PP860028.1:73751-74773;PP860031.1:73805-74827;PP860033.1:73810-74832;PQ153226.1:73776-74798;PQ178862.2:74889-75911;PQ221506.1:69865-70887;PQ221833.1:76031-77053;PQ340477.1:73759-74781;PQ340478.1:73744-74766;PQ340480.1:73823-74845;PQ340483.1:73807-74829;PQ340485.1:73793-74815;PQ340486.1:73808-74830;PQ340487.1:73833-74855;PQ340489.1:73808-74830;PQ340490.1:73759-74781;PQ340491.1:73814-74836;PQ340493.1:73793-74815;PQ340495.1:73823-74845;PQ340496.1:73856-74878;PQ368489.1:73779-74801;PQ451835.1:73864-74886;PQ451836.1:73796-74818;PQ451838.1:73847-74869;PQ451841.1:73807-74829;PQ451842.1:73759-74781 |
| OG0000078 | AY603973.1:76748-77500;AY741551.1:76752-77504;AY753185.1:76991-77743;DQ011154.1:77209-77961;DQ011155.1:77190-77942;EPI_ISL_13331598:74818-75570;EPI_ISL_13363142:74811-75563;EPI_ISL_13374487:74826-75578;EPI_ISL_13502582:74811-75563;EPI_ISL_13508471:74903-75655;EPI_ISL_13607904:74802-75554;EPI_ISL_13624509:74746-75498;EPI_ISL_13889442:74921-75673;EPI_ISL_13953610:74738-75490;EPI_ISL_14181948:74947-75699;EPI_ISL_14838587:77063-77815;EPI_ISL_15263355:74802-75554;EPI_ISL_15269699:74771-75523;EPI_ISL_15293002:75044-75796;EPI_ISL_15641561:74760-75512;EPI_ISL_15831211:74809-75561;EPI_ISL_15992098:74824-75576;EPI_ISL_16233784:74820-75572;EPI_ISL_16946400:74809-75561;EPI_ISL_17048206:74459-75211;EPI_ISL_17703567:74824-75576;EPI_ISL_17718458:74802-75554;EPI_ISL_18213374:74799-75551;EPI_ISL_18360394:74460-75212;EPI_ISL_18557816:74800-75552;EPI_ISL_18642365:74802-75554;EPI_ISL_18659846:74772-75524;EPI_ISL_18744049:74804-75556;EPI_ISL_19022858:74799-75551;EPI_ISL_19028778:74716-75468;EPI_ISL_19151800:74800-75552;EPI_ISL_19159116:74802-75554;EPI_ISL_19350788:75935-76687;EPI_ISL_19359677:74799-75551;EPI_ISL_19446306:74794-75546;EPI_ISL_19467692:77346-78098;JX878411.1:78054-78806;JX878425.1:77571-78323;JX878426.1:77020-77772;KC257460.1:76988-77740;KJ642612.1:75306-76058;KJ642613.1:76865-77617;KJ642616.1:76442-77194;KJ642618.1:74473-75225;KP849469.1:77379-78131;KP849471.1:77305-78057;LC831698.1:74800-75552;MN648051.1:75022-75774;MN702445.1:70722-71474;MN702446.1:76867-77619;MN702447.1:70235-70987;MN702449.1:70733-71485;MN702452.1:76908-77660;MT903338.1:74965-75717;MT903340.1:74802-75554;MT903341.1:66335-67087;MT903342.1:74817-75569;MT903347.1:76759-77511;ON568298.1:74948-75700;ON622713.1:75034-75786;ON622722.2:74805-75557;ON631963.1:74825-75577;ON649879.1:74635-75387;ON676707.1:74855-75607;ON755039.1:74465-75217;ON808413.1:74805-75557;ON880420.1:75032-75784;ON911481.2:74822-75574;ON918656.1:74837-75589;ON959133.1:74926-75678;ON959136.1:74927-75679;ON983168.1:73501-74253;OP019276.1:74799-75551;OP150924.1:74940-75692;OP160532.1:74765-75517;OP235282.1:74799-75551;OP289782.1:74802-75554;OP331335.1:74819-75571;OP382479.1:74806-75558;OP422341.1:74819-75571;OP434519.1:74798-75550;OP535324.1:74800-75552;OP535333.1:74909-75661;OP536770.1:74801-75553;OP597769.1:74823-75575;OP604566.1:74802-75554;OP612682.1:74728-75480;OP612684.1:74792-75544;OP612686.1:74792-75544;OP696841.1:72076-72828;OP879722.1:74025-74777;OQ419007.1:74035-74787;OQ419012.1:74002-74754;OQ419031.1:74006-74758;OQ419047.1:74008-74760;OQ451898.1:73887-74639;OQ451900.1:74800-75552;OQ581853.1:74826-75578;OQ581855.1:74907-75659;OQ721951.1:74840-75592;OQ721954.1:74797-75549;OR035652.1:74749-75501;OR035664.1:74749-75501;OR035702.1:74749-75501;OR146272.1:74765-75517;OR146330.1:74810-75562;OR264364.1:74820-75572;OR427332.1:74820-75572;OR459777.1:74804-75556;OR459797.1:74819-75571;OR943698.1:77049-77801;PP098589.2:74801-75553;PP496048.1:74805-75557;PP601206.1:77061-77813;PP776553.1:74726-75478;PP852971.1:74799-75551;PP852983.1:74805-75557;PP853000.1:74766-75518;PP853005.1:74774-75526;PP853007.1:74807-75559;PP853013.1:74780-75532;PP853027.1:74811-75563;PP853045.1:74792-75544;PP853048.1:74811-75563;PP860026.1:74809-75561;PP860027.1:74834-75586;PP860028.1:74774-75526;PP860031.1:74828-75580;PP860033.1:74833-75585;PQ153226.1:74799-75551;PQ178862.2:75912-76664;PQ221506.1:70888-71640;PQ221833.1:77054-77806;PQ340477.1:74782-75534;PQ340478.1:74767-75519;PQ340480.1:74846-75598;PQ340483.1:74830-75582;PQ340485.1:74816-75568;PQ340486.1:74831-75583;PQ340487.1:74856-75608;PQ340489.1:74831-75583;PQ340490.1:74782-75534;PQ340491.1:74837-75589;PQ340493.1:74816-75568;PQ340495.1:74846-75598;PQ340496.1:74879-75631;PQ368489.1:74802-75554;PQ451835.1:74887-75639;PQ451836.1:74819-75571;PQ451838.1:74870-75622;PQ451841.1:74830-75582;PQ451842.1:74782-75534 |
| OG0000079 | AY603973.1:78845-79600;AY741551.1:78849-79604;AY753185.1:79088-79843;DQ011154.1:79306-80061;DQ011155.1:79287-80042;EPI_ISL_13331598:76915-77670;EPI_ISL_13363142:76908-77663;EPI_ISL_13374487:76923-77678;EPI_ISL_13502582:76908-77663;EPI_ISL_13508471:77000-77755;EPI_ISL_13607904:76899-77654;EPI_ISL_13624509:76843-77598;EPI_ISL_13889442:77018-77773;EPI_ISL_13953610:76835-77590;EPI_ISL_14181948:77044-77799;EPI_ISL_14838587:79160-79915;EPI_ISL_15263355:76899-77654;EPI_ISL_15269699:76868-77623;EPI_ISL_15293002:77141-77896;EPI_ISL_15641561:76857-77612;EPI_ISL_15831211:76906-77661;EPI_ISL_15992098:76921-77676;EPI_ISL_16233784:76917-77672;EPI_ISL_16946400:76906-77661;EPI_ISL_17048206:76556-77311;EPI_ISL_17703567:76921-77676;EPI_ISL_17718458:76899-77654;EPI_ISL_18213374:76896-77651;EPI_ISL_18360394:76557-77312;EPI_ISL_18557816:76897-77652;EPI_ISL_18642365:76899-77654;EPI_ISL_18659846:76869-77624;EPI_ISL_18744049:76901-77656;EPI_ISL_19022858:76896-77651;EPI_ISL_19028778:76813-77568;EPI_ISL_19151800:76897-77652;EPI_ISL_19159116:76899-77654;EPI_ISL_19350788:78032-78787;EPI_ISL_19359677:76896-77651;EPI_ISL_19446306:76891-77646;EPI_ISL_19467692:79443-80198;JX878411.1:80151-80906;JX878425.1:79668-80423;JX878426.1:79117-79872;KC257460.1:79085-79840;KJ642612.1:77403-78158;KJ642613.1:78962-79717;KJ642616.1:78539-79294;KJ642618.1:76633-77388;KP849469.1:79476-80231;KP849471.1:79402-80157;LC831698.1:76897-77652;MN648051.1:77119-77874;MN702445.1:72819-73574;MN702446.1:78964-79719;MN702447.1:72332-73087;MN702449.1:72830-73585;MN702452.1:79005-79760;MT903338.1:77062-77817;MT903340.1:76899-77654;MT903341.1:68432-69187;MT903342.1:76914-77669;MT903347.1:78856-79611;ON568298.1:77045-77800;ON622713.1:77131-77886;ON622722.2:76902-77657;ON631963.1:76922-77677;ON649879.1:76732-77487;ON676707.1:76952-77707;ON755039.1:76562-77317;ON808413.1:76902-77657;ON880420.1:77129-77884;ON911481.2:76919-77674;ON918656.1:76934-77689;ON959133.1:77023-77778;ON959136.1:77024-77779;ON983168.1:75598-76353;OP019276.1:76896-77651;OP150924.1:77037-77792;OP160532.1:76862-77617;OP235282.1:76896-77651;OP289782.1:76899-77654;OP331335.1:76916-77671;OP382479.1:76903-77658;OP422341.1:76916-77671;OP434519.1:76895-77650;OP535324.1:76897-77652;OP535333.1:77006-77761;OP536770.1:76898-77653;OP597769.1:76920-77675;OP604566.1:76899-77654;OP612682.1:76824-77579;OP612684.1:76888-77643;OP612686.1:76888-77643;OP696841.1:74173-74928;OP879722.1:76122-76877;OQ419007.1:76132-76887;OQ419012.1:76099-76854;OQ419031.1:76103-76858;OQ419047.1:76105-76860;OQ451898.1:75984-76739;OQ451900.1:76897-77652;OQ581853.1:76923-77678;OQ581855.1:77004-77759;OQ721951.1:76937-77692;OQ721954.1:76894-77649;OR035652.1:76846-77601;OR035664.1:76846-77601;OR035702.1:76846-77601;OR146272.1:76862-77617;OR146330.1:76907-77662;OR264364.1:76917-77672;OR427332.1:76917-77672;OR459777.1:76901-77656;OR459797.1:76916-77671;OR943698.1:79209-79964;PP098589.2:76898-77653;PP496048.1:76902-77657;PP601206.1:79158-79913;PP776553.1:76823-77578;PP852971.1:76896-77651;PP852983.1:76902-77657;PP853000.1:76863-77618;PP853005.1:76871-77626;PP853007.1:76904-77659;PP853013.1:76877-77632;PP853027.1:76908-77663;PP853045.1:76889-77644;PP853048.1:76908-77663;PP860026.1:76906-77661;PP860027.1:76931-77686;PP860028.1:76871-77626;PP860031.1:76925-77680;PP860033.1:76930-77685;PQ153226.1:76896-77651;PQ178862.2:78009-78764;PQ221506.1:72985-73740;PQ221833.1:79151-79906;PQ340477.1:76879-77634;PQ340478.1:76864-77619;PQ340480.1:76943-77698;PQ340483.1:76927-77682;PQ340485.1:76913-77668;PQ340486.1:76928-77683;PQ340487.1:76953-77708;PQ340489.1:76928-77683;PQ340490.1:76879-77634;PQ340491.1:76934-77689;PQ340493.1:76913-77668;PQ340495.1:76943-77698;PQ340496.1:76976-77731;PQ368489.1:76899-77654;PQ451835.1:76984-77739;PQ451836.1:76916-77671;PQ451838.1:76967-77722;PQ451841.1:76927-77682;PQ451842.1:76879-77634 |
| OG0000080 | AY603973.1:79610-79996;AY741551.1:79614-80000;AY753185.1:79853-80239;DQ011154.1:80071-80457;DQ011155.1:80052-80438;EPI_ISL_13331598:77680-78066;EPI_ISL_13363142:77673-78059;EPI_ISL_13374487:77688-78074;EPI_ISL_13502582:77673-78059;EPI_ISL_13508471:77765-78151;EPI_ISL_13607904:77664-78050;EPI_ISL_13624509:77608-77994;EPI_ISL_13889442:77783-78169;EPI_ISL_13953610:77600-77986;EPI_ISL_14181948:77809-78195;EPI_ISL_14838587:79925-80311;EPI_ISL_15263355:77664-78050;EPI_ISL_15269699:77633-78019;EPI_ISL_15293002:77906-78292;EPI_ISL_15641561:77622-78008;EPI_ISL_15831211:77671-78057;EPI_ISL_15992098:77686-78072;EPI_ISL_16233784:77682-78068;EPI_ISL_16946400:77671-78057;EPI_ISL_17048206:77321-77707;EPI_ISL_17703567:77686-78072;EPI_ISL_17718458:77664-78050;EPI_ISL_18213374:77661-78047;EPI_ISL_18360394:77322-77708;EPI_ISL_18557816:77662-78048;EPI_ISL_18642365:77664-78050;EPI_ISL_18659846:77634-78020;EPI_ISL_18744049:77666-78052;EPI_ISL_19022858:77661-78047;EPI_ISL_19028778:77578-77964;EPI_ISL_19151800:77662-78048;EPI_ISL_19159116:77664-78050;EPI_ISL_19350788:78797-79183;EPI_ISL_19359677:77661-78047;EPI_ISL_19446306:77656-78042;EPI_ISL_19467692:80208-80594;JX878411.1:80916-81302;JX878425.1:80433-80819;JX878426.1:79882-80268;KC257460.1:79850-80236;KJ642612.1:78168-78554;KJ642613.1:79727-80113;KJ642616.1:79304-79690;KJ642618.1:77398-77784;KP849469.1:80241-80627;KP849471.1:80167-80553;LC831698.1:77662-78048;MN648051.1:77884-78270;MN702445.1:73584-73970;MN702446.1:79729-80115;MN702447.1:73097-73483;MN702449.1:73595-73981;MN702452.1:79770-80156;MT903338.1:77827-78213;MT903340.1:77664-78050;MT903341.1:69197-69583;MT903342.1:77679-78065;MT903347.1:79621-80007;ON568298.1:77810-78196;ON622713.1:77896-78282;ON622722.2:77667-78053;ON631963.1:77687-78073;ON649879.1:77497-77883;ON676707.1:77717-78103;ON755039.1:77327-77713;ON808413.1:77667-78053;ON880420.1:77894-78280;ON911481.2:77684-78070;ON918656.1:77699-78085;ON959133.1:77788-78174;ON959136.1:77789-78175;ON983168.1:76363-76749;OP019276.1:77661-78047;OP150924.1:77802-78188;OP160532.1:77627-78013;OP235282.1:77661-78047;OP289782.1:77664-78050;OP331335.1:77681-78067;OP382479.1:77668-78054;OP422341.1:77681-78067;OP434519.1:77660-78046;OP535324.1:77662-78048;OP535333.1:77771-78157;OP536770.1:77663-78049;OP597769.1:77685-78071;OP604566.1:77664-78050;OP612682.1:77589-77975;OP612684.1:77653-78039;OP612686.1:77653-78039;OP696841.1:74938-75324;OP879722.1:76887-77273;OQ419007.1:76897-77283;OQ419012.1:76864-77250;OQ419031.1:76868-77254;OQ419047.1:76870-77256;OQ451898.1:76749-77135;OQ451900.1:77662-78048;OQ581853.1:77688-78074;OQ581855.1:77769-78155;OQ721951.1:77702-78088;OQ721954.1:77659-78045;OR035652.1:77611-77997;OR035664.1:77611-77997;OR035702.1:77611-77997;OR146272.1:77627-78013;OR146330.1:77672-78058;OR264364.1:77682-78068;OR427332.1:77682-78068;OR459777.1:77666-78052;OR459797.1:77681-78067;OR943698.1:79974-80360;PP098589.2:77663-78049;PP496048.1:77667-78053;PP601206.1:79923-80309;PP776553.1:77588-77974;PP852971.1:77661-78047;PP852983.1:77667-78053;PP853000.1:77628-78014;PP853005.1:77636-78022;PP853007.1:77669-78055;PP853013.1:77642-78028;PP853027.1:77673-78059;PP853045.1:77654-78040;PP853048.1:77673-78059;PP860026.1:77671-78057;PP860027.1:77696-78082;PP860028.1:77636-78022;PP860031.1:77690-78076;PP860033.1:77695-78081;PQ153226.1:77661-78047;PQ178862.2:78774-79160;PQ221506.1:73750-74136;PQ221833.1:79916-80302;PQ340477.1:77644-78030;PQ340478.1:77629-78015;PQ340480.1:77708-78094;PQ340483.1:77692-78078;PQ340485.1:77678-78064;PQ340486.1:77693-78079;PQ340487.1:77718-78104;PQ340489.1:77693-78079;PQ340490.1:77644-78030;PQ340491.1:77699-78085;PQ340493.1:77678-78064;PQ340495.1:77708-78094;PQ340496.1:77741-78127;PQ368489.1:77664-78050;PQ451835.1:77749-78135;PQ451836.1:77681-78067;PQ451838.1:77732-78118;PQ451841.1:77692-78078;PQ451842.1:77644-78030 |
| OG0000081 | AY603973.1:93194-93628;AY741551.1:93198-93632;AY753185.1:93437-93871;DQ011154.1:93662-94102;DQ011155.1:93643-94083;EPI_ISL_13331598:91262-91702;EPI_ISL_13363142:91255-91695;EPI_ISL_13374487:91270-91710;EPI_ISL_13502582:91255-91695;EPI_ISL_13508471:91347-91787;EPI_ISL_13607904:91246-91686;EPI_ISL_13624509:91190-91630;EPI_ISL_13889442:91365-91805;EPI_ISL_13953610:91163-91603;EPI_ISL_14181948:91391-91831;EPI_ISL_14838587:93516-93956;EPI_ISL_15263355:91246-91686;EPI_ISL_15269699:91215-91655;EPI_ISL_15293002:91488-91928;EPI_ISL_15641561:91204-91644;EPI_ISL_15831211:91253-91693;EPI_ISL_15992098:91268-91708;EPI_ISL_16233784:91264-91704;EPI_ISL_16946400:91253-91693;EPI_ISL_17048206:90903-91343;EPI_ISL_17703567:91268-91708;EPI_ISL_17718458:91246-91686;EPI_ISL_18213374:91243-91683;EPI_ISL_18360394:90904-91344;EPI_ISL_18557816:91244-91684;EPI_ISL_18642365:91246-91686;EPI_ISL_18659846:91216-91656;EPI_ISL_18744049:91248-91688;EPI_ISL_19022858:91243-91683;EPI_ISL_19028778:91160-91600;EPI_ISL_19151800:91244-91684;EPI_ISL_19159116:91246-91686;EPI_ISL_19350788:92388-92828;EPI_ISL_19359677:91243-91683;EPI_ISL_19446306:91238-91678;EPI_ISL_19467692:93799-94239;JX878411.1:94507-94947;JX878425.1:94024-94464;JX878426.1:93473-93913;KC257460.1:93441-93881;KJ642612.1:91759-92199;KJ642613.1:93318-93758;KJ642616.1:92888-93322;KJ642618.1:90989-91429;KP849469.1:93832-94272;KP849471.1:93758-94198;LC831698.1:91244-91684;MN648051.1:91466-91906;MN702445.1:87175-87615;MN702446.1:93320-93760;MN702447.1:86688-87128;MN702449.1:87186-87626;MN702452.1:93361-93801;MT903338.1:91409-91849;MT903340.1:91246-91686;MT903341.1:82779-83219;MT903342.1:91261-91701;MT903347.1:93205-93639;ON568298.1:91392-91832;ON622713.1:91478-91918;ON622722.2:91249-91689;ON631963.1:91269-91709;ON649879.1:91079-91519;ON676707.1:91299-91739;ON755039.1:90909-91349;ON808413.1:91249-91689;ON880420.1:91476-91916;ON911481.2:91266-91706;ON918656.1:91281-91721;ON959133.1:91370-91810;ON959136.1:91371-91811;ON983168.1:89944-90384;OP019276.1:91243-91683;OP150924.1:91384-91824;OP160532.1:91209-91649;OP235282.1:91243-91683;OP289782.1:91246-91686;OP331335.1:91263-91703;OP382479.1:91250-91690;OP422341.1:91263-91703;OP434519.1:91242-91682;OP535324.1:91244-91684;OP535333.1:91353-91793;OP536770.1:91245-91685;OP597769.1:91267-91707;OP604566.1:91246-91686;OP612682.1:91171-91611;OP612684.1:91234-91674;OP612686.1:91235-91675;OP696841.1:88520-88960;OP879722.1:90469-90909;OQ419007.1:90479-90919;OQ419012.1:90446-90886;OQ419031.1:90450-90890;OQ419047.1:90452-90892;OQ451898.1:90331-90771;OQ451900.1:91244-91684;OQ581853.1:91270-91710;OQ581855.1:91351-91791;OQ721951.1:91284-91724;OQ721954.1:91241-91681;OR035652.1:91193-91633;OR035664.1:91193-91633;OR035702.1:91193-91633;OR146272.1:91209-91649;OR146330.1:91254-91694;OR264364.1:91264-91704;OR427332.1:91264-91704;OR459777.1:91248-91688;OR459797.1:91263-91703;OR943698.1:93574-94014;PP098589.2:91245-91685;PP496048.1:91249-91689;PP601206.1:93505-93945;PP776553.1:91170-91610;PP852971.1:91243-91683;PP852983.1:91249-91689;PP853000.1:91210-91650;PP853005.1:91218-91658;PP853007.1:91251-91691;PP853013.1:91224-91664;PP853027.1:91255-91695;PP853045.1:91236-91676;PP853048.1:91255-91695;PP860026.1:91253-91693;PP860027.1:91278-91718;PP860028.1:91218-91658;PP860031.1:91272-91712;PP860033.1:91277-91717;PQ153226.1:91243-91683;PQ178862.2:92365-92805;PQ221506.1:87341-87781;PQ221833.1:93507-93947;PQ340477.1:91226-91666;PQ340478.1:91211-91651;PQ340480.1:91290-91730;PQ340483.1:91274-91714;PQ340485.1:91260-91700;PQ340486.1:91275-91715;PQ340487.1:91300-91740;PQ340489.1:91275-91715;PQ340490.1:91226-91666;PQ340491.1:91281-91721;PQ340493.1:91260-91700;PQ340495.1:91290-91730;PQ340496.1:91323-91763;PQ368489.1:91246-91686;PQ451835.1:91331-91771;PQ451836.1:91263-91703;PQ451838.1:91314-91754;PQ451841.1:91274-91714;PQ451842.1:91226-91666 |
| OG0000082 | AY603973.1:104369-105115;AY741551.1:104373-105119;AY753185.1:104612-105358;DQ011154.1:104843-105589;DQ011155.1:104824-105570;EPI_ISL_13331598:102443-103189;EPI_ISL_13363142:102436-103182;EPI_ISL_13374487:102451-103197;EPI_ISL_13502582:102436-103182;EPI_ISL_13508471:102528-103274;EPI_ISL_13607904:102427-103173;EPI_ISL_13624509:102371-103117;EPI_ISL_13889442:102546-103292;EPI_ISL_13953610:102344-103090;EPI_ISL_14181948:102572-103318;EPI_ISL_14838587:104697-105443;EPI_ISL_15263355:102427-103173;EPI_ISL_15269699:102396-103142;EPI_ISL_15293002:102669-103415;EPI_ISL_15641561:102385-103131;EPI_ISL_15831211:102434-103180;EPI_ISL_15992098:102449-103195;EPI_ISL_16233784:102445-103191;EPI_ISL_16946400:102434-103180;EPI_ISL_17048206:102084-102830;EPI_ISL_17703567:102449-103195;EPI_ISL_17718458:102427-103173;EPI_ISL_18213374:102424-103170;EPI_ISL_18360394:102085-102831;EPI_ISL_18557816:102425-103171;EPI_ISL_18642365:102427-103173;EPI_ISL_18659846:102397-103143;EPI_ISL_18744049:102429-103175;EPI_ISL_19022858:102424-103170;EPI_ISL_19028778:102341-103087;EPI_ISL_19151800:102425-103171;EPI_ISL_19159116:102427-103173;EPI_ISL_19350788:103569-104315;EPI_ISL_19359677:102424-103170;EPI_ISL_19446306:102419-103165;EPI_ISL_19467692:104980-105726;JX878411.1:105688-106434;JX878425.1:105205-105951;JX878426.1:104654-105400;KC257460.1:104622-105368;KJ642612.1:102940-103686;KJ642613.1:104499-105245;KJ642616.1:104063-104809;KJ642618.1:102170-102916;KP849469.1:105013-105759;KP849471.1:104939-105685;LC831698.1:102425-103171;MN648051.1:102647-103393;MN702445.1:98356-99102;MN702446.1:104501-105247;MN702447.1:97869-98615;MN702449.1:98367-99113;MN702452.1:104542-105288;MT903338.1:102590-103336;MT903340.1:102427-103173;MT903341.1:93960-94706;MT903342.1:102442-103188;MT903347.1:104380-105126;ON568298.1:102573-103319;ON622713.1:102659-103405;ON622722.2:102430-103176;ON631963.1:102450-103196;ON649879.1:102260-103006;ON676707.1:102480-103226;ON755039.1:102090-102836;ON808413.1:102430-103176;ON880420.1:102657-103403;ON911481.2:102447-103193;ON918656.1:102462-103208;ON959133.1:102551-103297;ON959136.1:102552-103298;ON983168.1:101125-101871;OP019276.1:102424-103170;OP150924.1:102565-103311;OP160532.1:102390-103136;OP235282.1:102424-103170;OP289782.1:102427-103173;OP331335.1:102444-103190;OP382479.1:102431-103177;OP422341.1:102444-103190;OP434519.1:102423-103169;OP535324.1:102425-103171;OP535333.1:102534-103280;OP536770.1:102426-103172;OP597769.1:102448-103194;OP604566.1:102427-103173;OP612682.1:102352-103098;OP612684.1:102415-103161;OP612686.1:102416-103162;OP696841.1:99701-100447;OP879722.1:101650-102396;OQ419007.1:101660-102406;OQ419012.1:101627-102373;OQ419031.1:101631-102377;OQ419047.1:101633-102379;OQ451898.1:101512-102258;OQ451900.1:102425-103171;OQ581853.1:102451-103197;OQ581855.1:102532-103278;OQ721951.1:102465-103211;OQ721954.1:102422-103168;OR035652.1:102374-103120;OR035664.1:102374-103120;OR035702.1:102374-103120;OR146272.1:102390-103136;OR146330.1:102435-103181;OR264364.1:102445-103191;OR427332.1:102445-103191;OR459777.1:102429-103175;OR459797.1:102444-103190;OR943698.1:104755-105501;PP098589.2:102426-103172;PP496048.1:102430-103176;PP601206.1:104686-105432;PP776553.1:102351-103097;PP852971.1:102424-103170;PP852983.1:102430-103176;PP853000.1:102391-103137;PP853005.1:102399-103145;PP853007.1:102432-103178;PP853013.1:102405-103151;PP853027.1:102436-103182;PP853045.1:102417-103163;PP853048.1:102436-103182;PP860026.1:102434-103180;PP860027.1:102459-103205;PP860028.1:102399-103145;PP860031.1:102453-103199;PP860033.1:102458-103204;PQ153226.1:102424-103170;PQ178862.2:103546-104292;PQ221506.1:98522-99268;PQ221833.1:104688-105434;PQ340477.1:102407-103153;PQ340478.1:102392-103138;PQ340480.1:102471-103217;PQ340483.1:102455-103201;PQ340485.1:102441-103187;PQ340486.1:102456-103202;PQ340487.1:102481-103227;PQ340489.1:102456-103202;PQ340490.1:102407-103153;PQ340491.1:102462-103208;PQ340493.1:102441-103187;PQ340495.1:102471-103217;PQ340496.1:102504-103250;PQ368489.1:102427-103173;PQ451835.1:102512-103258;PQ451836.1:102444-103190;PQ451838.1:102495-103241;PQ451841.1:102455-103201;PQ451842.1:102407-103153 |
| OG0000083 | AY603973.1:125906-127384;AY741551.1:125928-127406;AY753185.1:126149-127627;DQ011154.1:126363-127841;DQ011155.1:126344-127822;EPI_ISL_13331598:123962-125440;EPI_ISL_13363142:123955-125433;EPI_ISL_13374487:123970-125448;EPI_ISL_13502582:123955-125433;EPI_ISL_13508471:124047-125525;EPI_ISL_13607904:123946-125424;EPI_ISL_13624509:123890-125368;EPI_ISL_13889442:124065-125543;EPI_ISL_13953610:123863-125341;EPI_ISL_14181948:124091-125569;EPI_ISL_14838587:126217-127695;EPI_ISL_15263355:123946-125424;EPI_ISL_15269699:123915-125393;EPI_ISL_15293002:124188-125666;EPI_ISL_15641561:123904-125382;EPI_ISL_15831211:123953-125431;EPI_ISL_15992098:123968-125446;EPI_ISL_16233784:123964-125445;EPI_ISL_16946400:123953-125431;EPI_ISL_17048206:123603-125081;EPI_ISL_17703567:123968-125446;EPI_ISL_17718458:123946-125424;EPI_ISL_18213374:123943-125421;EPI_ISL_18360394:123604-125082;EPI_ISL_18557816:123944-125422;EPI_ISL_18642365:123946-125424;EPI_ISL_18659846:123916-125394;EPI_ISL_18744049:123948-125426;EPI_ISL_19022858:123943-125421;EPI_ISL_19028778:123860-125338;EPI_ISL_19151800:123944-125422;EPI_ISL_19159116:123946-125424;EPI_ISL_19350788:125088-126566;EPI_ISL_19359677:123943-125421;EPI_ISL_19446306:123938-125416;EPI_ISL_19467692:126499-127977;JX878411.1:127208-128686;JX878425.1:126725-128203;JX878426.1:126175-127653;KC257460.1:126142-127620;KJ642612.1:124459-125937;KJ642613.1:126019-127497;KJ642616.1:125606-127084;KJ642618.1:123690-125168;KP849469.1:126533-128011;KP849471.1:126459-127937;LC831698.1:123944-125422;MN648051.1:124166-125644;MN702445.1:119876-121354;MN702446.1:126021-127499;MN702447.1:119389-120867;MN702449.1:119887-121365;MN702452.1:126062-127540;MT903338.1:124109-125587;MT903340.1:123946-125424;MT903341.1:115479-116957;MT903342.1:123961-125439;MT903347.1:125935-127413;ON568298.1:124092-125570;ON622713.1:124178-125656;ON622722.2:123949-125427;ON631963.1:123969-125447;ON649879.1:123779-125257;ON676707.1:123999-125477;ON755039.1:123609-125087;ON808413.1:123949-125427;ON880420.1:124176-125654;ON911481.2:123966-125444;ON918656.1:123981-125459;ON959133.1:124070-125548;ON959136.1:124071-125549;ON983168.1:122644-124122;OP019276.1:123943-125421;OP150924.1:124084-125562;OP160532.1:123909-125387;OP235282.1:123943-125421;OP289782.1:123946-125424;OP331335.1:123963-125441;OP382479.1:123950-125428;OP422341.1:123963-125441;OP434519.1:123942-125420;OP535324.1:123944-125422;OP535333.1:124053-125531;OP536770.1:123945-125423;OP597769.1:123967-125445;OP604566.1:123946-125424;OP612682.1:123871-125349;OP612684.1:123933-125411;OP612686.1:123935-125413;OP696841.1:121220-122698;OP879722.1:123169-124647;OQ419007.1:123179-124657;OQ419012.1:123146-124624;OQ419031.1:123150-124628;OQ419047.1:123152-124630;OQ451898.1:123031-124509;OQ451900.1:123944-125422;OQ581853.1:123970-125448;OQ581855.1:124051-125529;OQ721951.1:123984-125462;OQ721954.1:123941-125419;OR035652.1:123893-125371;OR035664.1:123893-125371;OR035702.1:123893-125371;OR146272.1:123909-125387;OR146330.1:123954-125432;OR264364.1:123964-125442;OR427332.1:123964-125442;OR459777.1:123948-125426;OR459797.1:123962-125440;OR943698.1:126275-127753;PP098589.2:123945-125423;PP496048.1:123949-125427;PP601206.1:126205-127683;PP776553.1:123870-125348;PP852971.1:123943-125421;PP852983.1:123949-125427;PP853000.1:123910-125388;PP853005.1:123918-125396;PP853007.1:123951-125429;PP853013.1:123924-125402;PP853027.1:123955-125433;PP853045.1:123936-125414;PP853048.1:123955-125433;PP860026.1:123953-125431;PP860027.1:123979-125457;PP860028.1:123918-125396;PP860031.1:123972-125450;PP860033.1:123978-125456;PQ153226.1:123943-125421;PQ178862.2:125065-126543;PQ221506.1:120042-121520;PQ221833.1:126208-127686;PQ340477.1:123926-125404;PQ340478.1:123911-125389;PQ340480.1:123990-125468;PQ340483.1:123974-125452;PQ340485.1:123960-125438;PQ340486.1:123975-125453;PQ340487.1:124000-125478;PQ340489.1:123975-125453;PQ340490.1:123926-125404;PQ340491.1:123981-125459;PQ340493.1:123960-125438;PQ340495.1:123990-125468;PQ340496.1:124023-125501;PQ368489.1:123946-125424;PQ451835.1:124031-125509;PQ451836.1:123963-125441;PQ451838.1:124014-125492;PQ451841.1:123974-125452;PQ451842.1:123926-125404 |
| OG0000084 | AY603973.1:129155-129718;AY741551.1:129177-129740;AY753185.1:129398-129961;DQ011154.1:129612-130175;DQ011155.1:129593-130156;EPI_ISL_13331598:127211-127774;EPI_ISL_13363142:127204-127767;EPI_ISL_13374487:127219-127782;EPI_ISL_13502582:127204-127767;EPI_ISL_13508471:127296-127859;EPI_ISL_13607904:127195-127758;EPI_ISL_13624509:127139-127702;EPI_ISL_13889442:127314-127877;EPI_ISL_13953610:127112-127675;EPI_ISL_14181948:127340-127903;EPI_ISL_14838587:129466-130029;EPI_ISL_15263355:127195-127758;EPI_ISL_15269699:127164-127727;EPI_ISL_15293002:127437-128000;EPI_ISL_15641561:127153-127716;EPI_ISL_15831211:127202-127765;EPI_ISL_15992098:127217-127780;EPI_ISL_16233784:127216-127779;EPI_ISL_16946400:127202-127765;EPI_ISL_17048206:126852-127415;EPI_ISL_17703567:127217-127780;EPI_ISL_17718458:127195-127758;EPI_ISL_18213374:127192-127755;EPI_ISL_18360394:126853-127416;EPI_ISL_18557816:127193-127756;EPI_ISL_18642365:127195-127758;EPI_ISL_18659846:127165-127728;EPI_ISL_18744049:127197-127760;EPI_ISL_19022858:127192-127755;EPI_ISL_19028778:127109-127672;EPI_ISL_19151800:127193-127756;EPI_ISL_19159116:127195-127758;EPI_ISL_19350788:128337-128900;EPI_ISL_19359677:127192-127755;EPI_ISL_19446306:127187-127750;EPI_ISL_19467692:129748-130311;JX878411.1:130457-131020;JX878425.1:129974-130537;JX878426.1:129424-129987;KC257460.1:129391-129954;KJ642612.1:127708-128271;KJ642613.1:129268-129831;KJ642616.1:128855-129418;KJ642618.1:126939-127502;KP849469.1:129782-130345;KP849471.1:129708-130271;LC831698.1:127193-127756;MN648051.1:127415-127978;MN702445.1:123125-123688;MN702446.1:129270-129833;MN702447.1:122638-123201;MN702449.1:123136-123699;MN702452.1:129311-129874;MT903338.1:127358-127921;MT903340.1:127195-127758;MT903341.1:118728-119291;MT903342.1:127210-127773;MT903347.1:129184-129747;ON568298.1:127341-127904;ON622713.1:127427-127990;ON622722.2:127198-127761;ON631963.1:127218-127781;ON649879.1:127028-127591;ON676707.1:127248-127811;ON755039.1:126858-127421;ON808413.1:127198-127761;ON880420.1:127425-127988;ON911481.2:127215-127778;ON918656.1:127230-127793;ON959133.1:127319-127882;ON959136.1:127320-127883;ON983168.1:125893-126456;OP019276.1:127192-127755;OP150924.1:127333-127896;OP160532.1:127158-127721;OP235282.1:127192-127755;OP289782.1:127195-127758;OP331335.1:127212-127775;OP382479.1:127199-127762;OP422341.1:127212-127775;OP434519.1:127191-127754;OP535324.1:127193-127756;OP535333.1:127302-127865;OP536770.1:127194-127757;OP597769.1:127216-127779;OP604566.1:127195-127758;OP612682.1:127120-127683;OP612684.1:127182-127745;OP612686.1:127184-127747;OP696841.1:124469-125032;OP879722.1:126418-126981;OQ419007.1:126428-126991;OQ419012.1:126395-126958;OQ419031.1:126399-126962;OQ419047.1:126401-126964;OQ451898.1:126280-126843;OQ451900.1:127193-127756;OQ581853.1:127219-127782;OQ581855.1:127300-127863;OQ721951.1:127233-127796;OQ721954.1:127190-127753;OR035652.1:127142-127705;OR035664.1:127142-127705;OR035702.1:127142-127705;OR146272.1:127158-127721;OR146330.1:127203-127766;OR264364.1:127213-127776;OR427332.1:127213-127776;OR459777.1:127197-127760;OR459797.1:127211-127774;OR943698.1:129524-130087;PP098589.2:127194-127757;PP496048.1:127198-127761;PP601206.1:129454-130017;PP776553.1:127119-127682;PP852971.1:127192-127755;PP852983.1:127198-127761;PP853000.1:127159-127722;PP853005.1:127167-127730;PP853007.1:127200-127763;PP853013.1:127173-127736;PP853027.1:127204-127767;PP853045.1:127185-127748;PP853048.1:127204-127767;PP860026.1:127202-127765;PP860027.1:127228-127791;PP860028.1:127167-127730;PP860031.1:127221-127784;PP860033.1:127227-127790;PQ153226.1:127192-127755;PQ178862.2:128314-128877;PQ221506.1:123291-123854;PQ221833.1:129457-130020;PQ340477.1:127175-127738;PQ340478.1:127160-127723;PQ340480.1:127239-127802;PQ340483.1:127223-127786;PQ340485.1:127209-127772;PQ340486.1:127224-127787;PQ340487.1:127249-127812;PQ340489.1:127224-127787;PQ340490.1:127175-127738;PQ340491.1:127230-127793;PQ340493.1:127209-127772;PQ340495.1:127239-127802;PQ340496.1:127272-127835;PQ368489.1:127195-127758;PQ451835.1:127280-127843;PQ451836.1:127212-127775;PQ451838.1:127263-127826;PQ451841.1:127223-127786;PQ451842.1:127175-127738 |
| OG0000085 | AY603973.1:130883-134377;AY741551.1:130905-134399;AY753185.1:131126-134620;DQ011154.1:131340-134834;DQ011155.1:131321-134815;EPI_ISL_13331598:128939-132433;EPI_ISL_13363142:128932-132426;EPI_ISL_13374487:128947-132441;EPI_ISL_13502582:128932-132426;EPI_ISL_13508471:129024-132518;EPI_ISL_13607904:128923-132417;EPI_ISL_13624509:128867-132361;EPI_ISL_13889442:129042-132536;EPI_ISL_13953610:128840-132334;EPI_ISL_14181948:129068-132562;EPI_ISL_14838587:131194-134688;EPI_ISL_15263355:128923-132417;EPI_ISL_15269699:128892-132386;EPI_ISL_15293002:129165-132659;EPI_ISL_15641561:128881-132375;EPI_ISL_15831211:128930-132424;EPI_ISL_15992098:128945-132439;EPI_ISL_16233784:128944-132438;EPI_ISL_16946400:128930-132424;EPI_ISL_17048206:128580-132074;EPI_ISL_17703567:128945-132439;EPI_ISL_17718458:128923-132417;EPI_ISL_18213374:128920-132414;EPI_ISL_18360394:128581-132075;EPI_ISL_18557816:128921-132415;EPI_ISL_18642365:128923-132417;EPI_ISL_18659846:128893-132387;EPI_ISL_18744049:128925-132419;EPI_ISL_19022858:128920-132414;EPI_ISL_19028778:128837-132331;EPI_ISL_19151800:128921-132415;EPI_ISL_19159116:128923-132417;EPI_ISL_19350788:130065-133559;EPI_ISL_19359677:128920-132414;EPI_ISL_19446306:128915-132409;EPI_ISL_19467692:131476-134970;JX878411.1:132185-135679;JX878425.1:131702-135196;JX878426.1:131152-134646;KC257460.1:131119-134613;KJ642612.1:129436-132930;KJ642613.1:130996-134490;KJ642616.1:130583-134077;KJ642618.1:128667-132161;KP849469.1:131510-135004;KP849471.1:131436-134930;LC831698.1:128921-132415;MN648051.1:129143-132637;MN702445.1:124853-128347;MN702446.1:130998-134492;MN702447.1:124366-127860;MN702449.1:124864-128358;MN702452.1:131039-134533;MT903338.1:129086-132580;MT903340.1:128923-132417;MT903341.1:120456-123950;MT903342.1:128938-132432;MT903347.1:130912-134406;ON568298.1:129069-132563;ON622713.1:129155-132649;ON622722.2:128926-132420;ON631963.1:128946-132440;ON649879.1:128756-132250;ON676707.1:128976-132470;ON755039.1:128586-132080;ON808413.1:128926-132420;ON880420.1:129153-132647;ON911481.2:128943-132437;ON918656.1:128958-132452;ON959133.1:129047-132541;ON959136.1:129048-132542;ON983168.1:127621-131115;OP019276.1:128920-132414;OP150924.1:129061-132555;OP160532.1:128886-132380;OP235282.1:128920-132414;OP289782.1:128923-132417;OP331335.1:128940-132434;OP382479.1:128927-132421;OP422341.1:128940-132434;OP434519.1:128919-132413;OP535324.1:128921-132415;OP535333.1:129030-132524;OP536770.1:128922-132416;OP597769.1:128944-132438;OP604566.1:128923-132417;OP612682.1:128848-132342;OP612684.1:128910-132404;OP612686.1:128912-132406;OP696841.1:126197-129691;OP879722.1:128146-131640;OQ419007.1:128156-131650;OQ419012.1:128123-131617;OQ419031.1:128127-131621;OQ419047.1:128129-131623;OQ451898.1:128008-131502;OQ451900.1:128921-132415;OQ581853.1:128947-132441;OQ581855.1:129028-132522;OQ721951.1:128961-132455;OQ721954.1:128918-132412;OR035652.1:128870-132364;OR035664.1:128870-132364;OR035702.1:128870-132364;OR146272.1:128886-132380;OR146330.1:128931-132425;OR264364.1:128941-132435;OR427332.1:128941-132435;OR459777.1:128925-132419;OR459797.1:128939-132433;OR943698.1:131252-134746;PP098589.2:128922-132416;PP496048.1:128926-132420;PP601206.1:131182-134676;PP776553.1:128847-132341;PP852971.1:128920-132414;PP852983.1:128926-132420;PP853000.1:128887-132381;PP853005.1:128895-132389;PP853007.1:128928-132422;PP853013.1:128901-132395;PP853027.1:128932-132426;PP853045.1:128913-132407;PP853048.1:128932-132426;PP860026.1:128930-132424;PP860027.1:128956-132450;PP860028.1:128895-132389;PP860031.1:128949-132443;PP860033.1:128955-132449;PQ153226.1:128920-132414;PQ178862.2:130042-133536;PQ221506.1:125019-128513;PQ221833.1:131185-134679;PQ340477.1:128903-132397;PQ340478.1:128888-132382;PQ340480.1:128967-132461;PQ340483.1:128951-132445;PQ340485.1:128937-132431;PQ340486.1:128952-132446;PQ340487.1:128977-132471;PQ340489.1:128952-132446;PQ340490.1:128903-132397;PQ340491.1:128958-132452;PQ340493.1:128937-132431;PQ340495.1:128967-132461;PQ340496.1:129000-132494;PQ368489.1:128923-132417;PQ451835.1:129008-132502;PQ451836.1:128940-132434;PQ451838.1:128991-132485;PQ451841.1:128951-132445;PQ451842.1:128903-132397 |
| OG0000086 | AY603973.1:143006-143551;AY741551.1:143051-143596;AY753185.1:143249-143794;DQ011154.1:143075-143620;DQ011155.1:143044-143589;EPI_ISL_13331598:141113-141658;EPI_ISL_13363142:141100-141645;EPI_ISL_13374487:141117-141662;EPI_ISL_13502582:141112-141657;EPI_ISL_13508471:141195-141740;EPI_ISL_13607904:141091-141636;EPI_ISL_13624509:141035-141580;EPI_ISL_13889442:141212-141757;EPI_ISL_13953610:141018-141563;EPI_ISL_14181948:141241-141786;EPI_ISL_14838587:142941-143486;EPI_ISL_15263355:141094-141639;EPI_ISL_15269699:141063-141608;EPI_ISL_15293002:141332-141877;EPI_ISL_15641561:141049-141594;EPI_ISL_15831211:141102-141647;EPI_ISL_15992098:141126-141671;EPI_ISL_16233784:141115-141660;EPI_ISL_16946400:141104-141649;EPI_ISL_17048206:140751-141296;EPI_ISL_17703567:141116-141661;EPI_ISL_17718458:141086-141631;EPI_ISL_18213374:141090-141635;EPI_ISL_18360394:140749-141294;EPI_ISL_18557816:141091-141636;EPI_ISL_18642365:141088-141633;EPI_ISL_18659846:141064-141609;EPI_ISL_18744049:141103-141648;EPI_ISL_19022858:141070-141615;EPI_ISL_19028778:141005-141550;EPI_ISL_19151800:141088-141633;EPI_ISL_19159116:141091-141636;EPI_ISL_19350788:141767-142312;EPI_ISL_19359677:141088-141633;EPI_ISL_19446306:141093-141638;EPI_ISL_19467692:143205-143750;JX878411.1:143920-144465;JX878425.1:143428-143973;JX878426.1:142881-143426;KC257460.1:142821-143366;KJ642612.1:141150-141695;KJ642613.1:142710-143255;KJ642616.1:142731-143276;KJ642618.1:140337-140882;KP849469.1:143242-143787;KP849471.1:143169-143714;LC831698.1:141089-141634;MN648051.1:141315-141860;MN702445.1:136567-137112;MN702446.1:142730-143275;MN702447.1:136104-136649;MN702449.1:136593-137138;MN702452.1:142777-143322;MT903338.1:141267-141812;MT903340.1:141101-141646;MT903341.1:132635-133180;MT903342.1:141119-141664;MT903347.1:143072-143617;ON568298.1:141242-141787;ON622713.1:141323-141868;ON622722.2:141094-141639;ON631963.1:141120-141665;ON649879.1:140926-141471;ON676707.1:141147-141692;ON755039.1:140748-141293;ON808413.1:141085-141630;ON880420.1:141321-141866;ON911481.2:141106-141651;ON918656.1:141128-141673;ON959133.1:141217-141762;ON959136.1:141218-141763;ON983168.1:139793-140338;OP019276.1:141088-141633;OP150924.1:141231-141776;OP160532.1:141054-141599;OP235282.1:141088-141633;OP289782.1:141091-141636;OP331335.1:141121-141666;OP382479.1:141098-141643;OP422341.1:141111-141656;OP434519.1:141087-141632;OP535324.1:141101-141646;OP535333.1:141208-141753;OP536770.1:141091-141636;OP597769.1:141125-141670;OP604566.1:141091-141636;OP612682.1:141026-141571;OP612684.1:141077-141622;OP612686.1:141079-141624;OP696841.1:138367-138912;OP879722.1:140314-140859;OQ419007.1:140317-140862;OQ419012.1:140291-140836;OQ419031.1:140295-140840;OQ419047.1:140297-140842;OQ451898.1:140178-140723;OQ451900.1:141091-141636;OQ581853.1:141117-141662;OQ581855.1:141198-141743;OQ721951.1:141118-141663;OQ721954.1:141091-141636;OR035652.1:141037-141582;OR035664.1:141041-141586;OR035702.1:141038-141583;OR146272.1:141057-141602;OR146330.1:141101-141646;OR264364.1:141112-141657;OR427332.1:141112-141657;OR459777.1:141091-141636;OR459797.1:141110-141655;OR943698.1:142951-143496;PP098589.2:141090-141635;PP496048.1:141094-141639;PP601206.1:142911-143456;PP776553.1:141014-141559;PP852971.1:141089-141634;PP852983.1:141106-141651;PP853000.1:141057-141602;PP853005.1:141065-141610;PP853007.1:141108-141653;PP853013.1:141080-141625;PP853027.1:141113-141658;PP853045.1:141073-141618;PP853048.1:141098-141643;PP860026.1:141103-141648;PP860027.1:141157-141702;PP860028.1:141066-141611;PP860031.1:141139-141684;PP860033.1:141140-141685;PQ153226.1:141088-141633;PQ178862.2:141747-142292;PQ221506.1:136739-137284;PQ221833.1:142914-143459;PQ340477.1:141075-141620;PQ340478.1:141057-141602;PQ340480.1:141135-141680;PQ340483.1:141121-141666;PQ340485.1:141104-141649;PQ340486.1:141122-141667;PQ340487.1:141146-141691;PQ340489.1:141121-141666;PQ340490.1:141073-141618;PQ340491.1:141128-141673;PQ340493.1:141112-141657;PQ340495.1:141138-141683;PQ340496.1:141173-141718;PQ368489.1:141104-141649;PQ451835.1:141178-141723;PQ451836.1:141116-141661;PQ451838.1:141166-141711;PQ451841.1:141129-141674;PQ451842.1:141075-141620 |
| OG0000087 | AY603973.1:149342-149932;AY741551.1:149399-149989;AY753185.1:149597-150187;DQ011154.1:149429-150031;DQ011155.1:149400-149996;EPI_ISL_13331598:147438-148028;EPI_ISL_13363142:147425-148015;EPI_ISL_13374487:147442-148032;EPI_ISL_13502582:147437-148027;EPI_ISL_13508471:147520-148110;EPI_ISL_13607904:147416-148006;EPI_ISL_13624509:147360-147950;EPI_ISL_13889442:147537-148127;EPI_ISL_13953610:147343-147933;EPI_ISL_14181948:147566-148156;EPI_ISL_14838587:149298-149894;EPI_ISL_15263355:147419-148009;EPI_ISL_15269699:147388-147978;EPI_ISL_15293002:147657-148247;EPI_ISL_15641561:147374-147964;EPI_ISL_15831211:147427-148017;EPI_ISL_15992098:147451-148041;EPI_ISL_16233784:147440-148030;EPI_ISL_16946400:147429-148019;EPI_ISL_17048206:147076-147666;EPI_ISL_17703567:147441-148031;EPI_ISL_17718458:147411-148001;EPI_ISL_18213374:147415-148005;EPI_ISL_18360394:147276-147455;EPI_ISL_18557816:147416-148006;EPI_ISL_18642365:147413-148003;EPI_ISL_18659846:147389-147979;EPI_ISL_18744049:147428-148018;EPI_ISL_19022858:147395-147985;EPI_ISL_19028778:147330-147920;EPI_ISL_19151800:147413-148003;EPI_ISL_19159116:147416-148006;EPI_ISL_19350788:148059-148670;EPI_ISL_19359677:147413-148003;EPI_ISL_19446306:147418-148008;EPI_ISL_19467692:149559-150164;JX878411.1:150275-150877;JX878425.1:149785-150381;JX878426.1:149226-149819;KC257460.1:149177-149779;KJ642612.1:147483-148076;KJ642613.1:149064-149660;KJ642616.1:149072-149662;KJ642618.1:146669-147274;KP849469.1:149596-150198;KP849471.1:149526-150128;LC831698.1:147414-148004;MN648051.1:147640-148230;MN702445.1:142920-143528;MN702446.1:149083-149691;MN702447.1:142458-143063;MN702449.1:142960-143565;MN702452.1:149131-149733;MT903338.1:147573-148163;MT903340.1:147426-148016;MT903341.1:138967-139557;MT903342.1:147463-148053;MT903347.1:149420-150010;ON568298.1:147567-148157;ON622713.1:147648-148238;ON622722.2:147419-148009;ON631963.1:147445-148035;ON649879.1:147251-147841;ON676707.1:147472-148062;ON755039.1:147073-147663;ON808413.1:147410-148000;ON880420.1:147646-148236;ON911481.2:147431-148021;ON918656.1:147453-148043;ON959133.1:147542-148132;ON959136.1:147543-148133;ON983168.1:146118-146708;OP019276.1:147413-148003;OP150924.1:147556-148146;OP160532.1:147379-147969;OP235282.1:147413-148003;OP289782.1:147416-148006;OP331335.1:147446-148036;OP382479.1:147423-148013;OP422341.1:147436-148026;OP434519.1:147412-148002;OP535324.1:147426-148016;OP535333.1:147533-148123;OP536770.1:147416-148006;OP597769.1:147450-148040;OP604566.1:147416-148006;OP612682.1:147351-147941;OP612684.1:147402-147992;OP612686.1:147404-147994;OP696841.1:144692-145282;OP879722.1:146639-147229;OQ419007.1:146642-147232;OQ419012.1:146616-147206;OQ419031.1:146620-147210;OQ419047.1:146622-147212;OQ451898.1:146503-147093;OQ451900.1:147416-148006;OQ581853.1:147442-148032;OQ581855.1:147523-148113;OQ721951.1:147443-148033;OQ721954.1:147416-148006;OR035652.1:147362-147952;OR035664.1:147366-147956;OR035702.1:147363-147953;OR146272.1:147382-147972;OR146330.1:147426-148016;OR264364.1:147437-148027;OR427332.1:147437-148027;OR459777.1:147416-148006;OR459797.1:147435-148025;OR943698.1:149284-149886;PP098589.2:147415-148005;PP496048.1:147419-148009;PP601206.1:149243-149845;PP776553.1:147339-147929;PP852971.1:147414-148004;PP852983.1:147431-148021;PP853000.1:147382-147972;PP853005.1:147390-147980;PP853007.1:147433-148023;PP853013.1:147405-147995;PP853027.1:147438-148028;PP853045.1:147398-147988;PP853048.1:147423-148013;PP860026.1:147420-148010;PP860027.1:147463-148053;PP860028.1:147383-147973;PP860031.1:147445-148035;PP860033.1:147446-148036;PQ153226.1:147413-148003;PQ178862.2:148038-148649;PQ221506.1:143072-143665;PQ221833.1:149246-149839;PQ340477.1:147400-147990;PQ340478.1:147382-147972;PQ340480.1:147460-148050;PQ340483.1:147446-148036;PQ340485.1:147429-148019;PQ340486.1:147447-148037;PQ340487.1:147471-148061;PQ340489.1:147446-148036;PQ340490.1:147398-147988;PQ340491.1:147453-148043;PQ340493.1:147437-148027;PQ340495.1:147463-148053;PQ340496.1:147498-148088;PQ368489.1:147429-148019;PQ451835.1:147503-148093;PQ451836.1:147441-148031;PQ451838.1:147491-148081;PQ451841.1:147454-148044;PQ451842.1:147400-147990 |
| OG0000088 | AY603973.1:151724-152446;AY741551.1:151783-152505;AY753185.1:151979-152701;DQ011154.1:151828-152550;DQ011155.1:151789-152511;EPI_ISL_13331598:149820-150542;EPI_ISL_13363142:149807-150529;EPI_ISL_13374487:149824-150546;EPI_ISL_13502582:149819-150541;EPI_ISL_13508471:149902-150624;EPI_ISL_13607904:149798-150520;EPI_ISL_13624509:149742-150464;EPI_ISL_13889442:149919-150641;EPI_ISL_13953610:149961-150440;EPI_ISL_14181948:149948-150670;EPI_ISL_14838587:151688-152410;EPI_ISL_15263355:149801-150523;EPI_ISL_15269699:149770-150492;EPI_ISL_15293002:150039-150761;EPI_ISL_15641561:149756-150478;EPI_ISL_15831211:149809-150531;EPI_ISL_15992098:149833-150555;EPI_ISL_16233784:149822-150544;EPI_ISL_16946400:149811-150533;EPI_ISL_17048206:149458-150180;EPI_ISL_17703567:149823-150545;EPI_ISL_17718458:150029-150508;EPI_ISL_18213374:149797-150519;EPI_ISL_18360394:149104-149826;EPI_ISL_18557816:149798-150520;EPI_ISL_18642365:149795-150517;EPI_ISL_18659846:149771-150493;EPI_ISL_18744049:150046-150525;EPI_ISL_19022858:149777-150499;EPI_ISL_19028778:149712-150434;EPI_ISL_19151800:149795-150517;EPI_ISL_19159116:149798-150520;EPI_ISL_19350788:150465-151187;EPI_ISL_19359677:149795-150517;EPI_ISL_19446306:149800-150522;EPI_ISL_19467692:151959-152681;JX878411.1:152669-153391;JX878425.1:152176-152898;JX878426.1:151611-152333;KC257460.1:151572-152294;KJ642612.1:149870-150592;KJ642613.1:151455-152177;KJ642616.1:151455-152177;KJ642618.1:149068-149790;KP849469.1:151994-152716;KP849471.1:151919-152641;LC831698.1:149796-150518;MN648051.1:150022-150744;MN702445.1:145323-146045;MN702446.1:151487-152209;MN702447.1:144858-145580;MN702449.1:145361-146083;MN702452.1:151528-152250;MT903338.1:149955-150677;MT903340.1:149808-150530;MT903341.1:141349-142071;MT903342.1:149845-150567;MT903347.1:151803-152525;ON568298.1:149949-150671;ON622713.1:150030-150752;ON622722.2:149801-150523;ON631963.1:149827-150549;ON649879.1:149633-150355;ON676707.1:150090-150569;ON755039.1:149455-150177;ON808413.1:149792-150514;ON880420.1:150028-150750;ON911481.2:149813-150535;ON918656.1:149835-150557;ON959133.1:149924-150646;ON959136.1:149925-150647;ON983168.1:148501-149223;OP019276.1:149795-150517;OP150924.1:149938-150660;OP160532.1:149761-150483;OP235282.1:149795-150517;OP289782.1:149798-150520;OP331335.1:150064-150543;OP382479.1:149805-150527;OP422341.1:149818-150540;OP434519.1:149794-150516;OP535324.1:149808-150530;OP535333.1:149915-150637;OP536770.1:149798-150520;OP597769.1:150075-150554;OP604566.1:149798-150520;OP612682.1:149733-150455;OP612684.1:149784-150506;OP612686.1:149786-150508;OP696841.1:147074-147796;OP879722.1:149021-149743;OQ419007.1:149024-149746;OQ419012.1:148998-149720;OQ419031.1:149002-149724;OQ419047.1:149004-149726;OQ451898.1:148885-149607;OQ451900.1:149798-150520;OQ581853.1:149824-150546;OQ581855.1:149905-150627;OQ721951.1:149825-150547;OQ721954.1:149798-150520;OR035652.1:149744-150466;OR035664.1:149748-150470;OR035702.1:149745-150467;OR146272.1:149764-150486;OR146330.1:149808-150530;OR264364.1:149819-150541;OR427332.1:149819-150541;OR459777.1:150034-150513;OR459797.1:149817-150539;OR943698.1:151677-152399;PP098589.2:149797-150519;PP496048.1:149801-150523;PP601206.1:151641-152363;PP776553.1:149721-150443;PP852971.1:150032-150511;PP852983.1:150049-150528;PP853000.1:150000-150479;PP853005.1:150008-150487;PP853007.1:150051-150530;PP853013.1:150023-150502;PP853027.1:150056-150535;PP853045.1:150016-150495;PP853048.1:150041-150520;PP860026.1:149802-150524;PP860027.1:149845-150567;PP860028.1:149765-150487;PP860031.1:149827-150549;PP860033.1:149828-150550;PQ153226.1:149795-150517;PQ178862.2:150444-151166;PQ221506.1:145458-146180;PQ221833.1:151634-152356;PQ340477.1:149782-150504;PQ340478.1:149764-150486;PQ340480.1:149842-150564;PQ340483.1:149828-150550;PQ340485.1:149811-150533;PQ340486.1:149829-150551;PQ340487.1:149853-150575;PQ340489.1:149828-150550;PQ340490.1:149780-150502;PQ340491.1:149835-150557;PQ340493.1:149819-150541;PQ340495.1:149845-150567;PQ340496.1:149880-150602;PQ368489.1:149811-150533;PQ451835.1:149885-150607;PQ451836.1:149823-150545;PQ451838.1:149873-150595;PQ451841.1:149836-150558;PQ451842.1:149782-150504 |
| OG0000089 | AY603973.1:163448-164965;AY741551.1:163519-165036;AY753185.1:163703-165220;DQ011154.1:161374-162885;DQ011155.1:161316-162827;EPI_ISL_13331598:161584-163101;EPI_ISL_13363142:161572-163089;EPI_ISL_13374487:161605-163122;EPI_ISL_13502582:161600-163117;EPI_ISL_13508471:161666-163183;EPI_ISL_13607904:161571-163088;EPI_ISL_13624509:161506-163023;EPI_ISL_13889442:161699-163216;EPI_ISL_13953610:161499-163016;EPI_ISL_14181948:161729-163246;EPI_ISL_14838587:161313-162824;EPI_ISL_15263355:161565-163082;EPI_ISL_15269699:161534-163051;EPI_ISL_15293002:161803-163320;EPI_ISL_15641561:161520-163037;EPI_ISL_15831211:161574-163091;EPI_ISL_15992098:161614-163131;EPI_ISL_16233784:161586-163103;EPI_ISL_16946400:161576-163093;EPI_ISL_17048206:161240-162757;EPI_ISL_17703567:161587-163104;EPI_ISL_17718458:161567-163084;EPI_ISL_18213374:161578-163095;EPI_ISL_18360394:160885-162402;EPI_ISL_18557816:161581-163098;EPI_ISL_18642365:161576-163093;EPI_ISL_18659846:161535-163052;EPI_ISL_18744049:161584-163101;EPI_ISL_19022858:161541-163058;EPI_ISL_19028778:161493-163010;EPI_ISL_19151800:161577-163094;EPI_ISL_19159116:161562-163079;EPI_ISL_19350788:159971-161482;EPI_ISL_19359677:161577-163094;EPI_ISL_19446306:161564-163081;EPI_ISL_19467692:161514-163025;JX878411.1:162216-163727;JX878425.1:161703-163214;JX878426.1:161243-162754;KC257460.1:161099-162610;KJ642612.1:159433-160944;KJ642613.1:160982-162493;KJ642616.1:163167-164684;KJ642618.1:158899-160410;KP849469.1:161532-163043;KP849471.1:161467-162978;LC831698.1:161560-163077;MN648051.1:161803-163320;MN702445.1:154850-156361;MN702446.1:161014-162525;MN702447.1:154377-155888;MN702449.1:154888-156399;MN702452.1:161065-162576;MT903338.1:161736-163253;MT903340.1:161589-163106;MT903341.1:153130-154647;MT903342.1:161626-163143;MT903347.1:163518-165035;ON568298.1:161730-163247;ON622713.1:161794-163311;ON622722.2:161582-163099;ON631963.1:161591-163108;ON649879.1:161397-162914;ON676707.1:161628-163145;ON755039.1:161219-162736;ON808413.1:161556-163073;ON880420.1:161792-163309;ON911481.2:161577-163094;ON918656.1:161599-163116;ON959133.1:161688-163205;ON959136.1:161689-163206;ON983168.1:160265-161782;OP019276.1:161576-163093;OP150924.1:161702-163219;OP160532.1:161525-163042;OP235282.1:161576-163093;OP289782.1:161580-163097;OP331335.1:161585-163102;OP382479.1:161569-163086;OP422341.1:161582-163099;OP434519.1:161558-163075;OP535324.1:161589-163106;OP535333.1:161696-163213;OP536770.1:161579-163096;OP597769.1:161613-163130;OP604566.1:161579-163096;OP612682.1:161514-163031;OP612684.1:161565-163082;OP612686.1:161567-163084;OP696841.1:158855-160372;OP879722.1:160802-162319;OQ419007.1:160796-162313;OQ419012.1:160770-162287;OQ419031.1:160774-162291;OQ419047.1:160776-162293;OQ451898.1:160666-162183;OQ451900.1:161580-163097;OQ581853.1:161588-163105;OQ581855.1:161687-163204;OQ721951.1:161606-163123;OQ721954.1:161579-163096;OR035652.1:161508-163025;OR035664.1:161512-163029;OR035702.1:161509-163026;OR146272.1:161528-163045;OR146330.1:161589-163106;OR264364.1:161583-163100;OR427332.1:161583-163100;OR459777.1:161572-163089;OR459797.1:161581-163098;OR943698.1:161218-162729;PP098589.2:161578-163095;PP496048.1:161582-163099;PP601206.1:161274-162785;PP776553.1:161502-163019;PP852971.1:161570-163087;PP852983.1:161587-163104;PP853000.1:161538-163055;PP853005.1:161546-163063;PP853007.1:161589-163106;PP853013.1:161561-163078;PP853027.1:161603-163120;PP853045.1:161554-163071;PP853048.1:161579-163096;PP860026.1:161574-163091;PP860027.1:161613-163130;PP860028.1:161537-163054;PP860031.1:161591-163108;PP860033.1:161596-163113;PQ153226.1:161559-163076;PQ178862.2:159950-161461;PQ221506.1:155064-156575;PQ221833.1:161250-162761;PQ340477.1:161546-163063;PQ340478.1:161528-163045;PQ340480.1:161606-163123;PQ340483.1:161592-163109;PQ340485.1:161575-163092;PQ340486.1:161593-163110;PQ340487.1:161617-163134;PQ340489.1:161592-163109;PQ340490.1:161544-163061;PQ340491.1:161599-163116;PQ340493.1:161583-163100;PQ340495.1:161609-163126;PQ340496.1:161644-163161;PQ368489.1:161592-163109;PQ451835.1:161649-163166;PQ451836.1:161587-163104;PQ451838.1:161637-163154;PQ451841.1:161600-163117;PQ451842.1:161546-163063 |
| OG0000090 | AY603973.1:165191-166885;AY741551.1:165225-166919;AY753185.1:165454-167148;DQ011154.1:163103-164788;DQ011155.1:163045-164730;EPI_ISL_13331598:163327-165012;EPI_ISL_13363142:163315-165000;EPI_ISL_13374487:163348-165033;EPI_ISL_13502582:163343-165028;EPI_ISL_13508471:163409-165094;EPI_ISL_13607904:163314-164999;EPI_ISL_13624509:163249-164934;EPI_ISL_13889442:163442-165127;EPI_ISL_13953610:163242-164927;EPI_ISL_14181948:163472-165157;EPI_ISL_14838587:163057-164742;EPI_ISL_15263355:163308-164993;EPI_ISL_15269699:163277-164962;EPI_ISL_15293002:163546-165231;EPI_ISL_15641561:163263-164948;EPI_ISL_15831211:163317-165002;EPI_ISL_15992098:163357-165042;EPI_ISL_16233784:163329-165014;EPI_ISL_16946400:163319-165004;EPI_ISL_17048206:162983-164668;EPI_ISL_17703567:163330-165015;EPI_ISL_17718458:163310-164995;EPI_ISL_18213374:163321-165006;EPI_ISL_18360394:162628-164313;EPI_ISL_18557816:163324-165009;EPI_ISL_18642365:163319-165004;EPI_ISL_18659846:163278-164963;EPI_ISL_18744049:163327-165012;EPI_ISL_19022858:163284-164969;EPI_ISL_19028778:163236-164921;EPI_ISL_19151800:163320-165005;EPI_ISL_19159116:163305-164990;EPI_ISL_19350788:161696-163381;EPI_ISL_19359677:163320-165005;EPI_ISL_19446306:163307-164992;EPI_ISL_19467692:163243-164928;JX878411.1:163945-165630;JX878425.1:163432-165117;JX878426.1:163002-164687;KC257460.1:162828-164513;KJ642612.1:161162-162847;KJ642613.1:162711-164396;KJ642616.1:164910-166604;KJ642618.1:160628-162313;KP849469.1:163261-164946;KP849471.1:163196-164881;LC831698.1:163303-164988;MN648051.1:163546-165231;MN702445.1:156579-158264;MN702446.1:162743-164428;MN702447.1:156106-157791;MN702449.1:156617-158302;MN702452.1:162794-164479;MT903338.1:163479-165164;MT903340.1:163332-165017;MT903341.1:154873-156558;MT903342.1:163369-165054;MT903347.1:165269-166954;ON568298.1:163473-165158;ON622713.1:163537-165222;ON622722.2:163325-165010;ON631963.1:163334-165019;ON649879.1:163140-164825;ON676707.1:163371-165056;ON755039.1:162962-164647;ON808413.1:163299-164984;ON880420.1:163535-165220;ON911481.2:163320-165005;ON918656.1:163342-165027;ON959133.1:163431-165116;ON959136.1:163432-165117;ON983168.1:162008-163693;OP019276.1:163319-165004;OP150924.1:163445-165130;OP160532.1:163268-164953;OP235282.1:163319-165004;OP289782.1:163323-165008;OP331335.1:163328-165013;OP382479.1:163312-164997;OP422341.1:163325-165010;OP434519.1:163301-164986;OP535324.1:163332-165017;OP535333.1:163439-165124;OP536770.1:163322-165007;OP597769.1:163356-165041;OP604566.1:163322-165007;OP612682.1:163257-164942;OP612684.1:163308-164993;OP612686.1:163310-164995;OP696841.1:160598-162283;OP879722.1:162545-164230;OQ419007.1:162539-164224;OQ419012.1:162513-164198;OQ419031.1:162517-164202;OQ419047.1:162519-164204;OQ451898.1:162409-164094;OQ451900.1:163323-165008;OQ581853.1:163331-165016;OQ581855.1:163430-165115;OQ721951.1:163349-165034;OQ721954.1:163322-165007;OR035652.1:163251-164936;OR035664.1:163255-164940;OR035702.1:163252-164937;OR146272.1:163271-164956;OR146330.1:163332-165017;OR264364.1:163326-165011;OR427332.1:163326-165011;OR459777.1:163315-165000;OR459797.1:163324-165009;OR943698.1:162951-164636;PP098589.2:163321-165006;PP496048.1:163325-165010;PP601206.1:163003-164688;PP776553.1:163245-164930;PP852971.1:163309-164994;PP852983.1:163330-165015;PP853000.1:163281-164966;PP853005.1:163289-164974;PP853007.1:163332-165017;PP853013.1:163304-164989;PP853027.1:163346-165031;PP853045.1:163297-164982;PP853048.1:163322-165007;PP860026.1:163317-165002;PP860027.1:163348-165033;PP860028.1:163280-164965;PP860031.1:163334-165019;PP860033.1:163331-165016;PQ153226.1:163302-164987;PQ178862.2:161675-163360;PQ221506.1:156838-158523;PQ221833.1:162978-164663;PQ340477.1:163289-164974;PQ340478.1:163271-164956;PQ340480.1:163349-165034;PQ340483.1:163335-165020;PQ340485.1:163318-165003;PQ340486.1:163336-165021;PQ340487.1:163360-165045;PQ340489.1:163335-165020;PQ340490.1:163287-164972;PQ340491.1:163342-165027;PQ340493.1:163326-165011;PQ340495.1:163352-165037;PQ340496.1:163387-165072;PQ368489.1:163335-165020;PQ451835.1:163392-165077;PQ451836.1:163330-165015;PQ451838.1:163380-165065;PQ451841.1:163343-165028;PQ451842.1:163289-164974 |
| OG0000091 | AY603973.1:168014-168544;AY741551.1:168048-168578;AY753185.1:168277-168807;DQ011154.1:165938-166468;DQ011155.1:166051-166410;EPI_ISL_13331598:166155-166661;EPI_ISL_13363142:166143-166649;EPI_ISL_13374487:166176-166682;EPI_ISL_13502582:166171-166677;EPI_ISL_13508471:166237-166743;EPI_ISL_13607904:166142-166648;EPI_ISL_13624509:166077-166583;EPI_ISL_13889442:166270-166776;EPI_ISL_13953610:166070-166576;EPI_ISL_14181948:166300-166806;EPI_ISL_14838587:165892-166422;EPI_ISL_15263355:166136-166642;EPI_ISL_15269699:166105-166611;EPI_ISL_15293002:166374-166880;EPI_ISL_15641561:166091-166597;EPI_ISL_15831211:166145-166651;EPI_ISL_15992098:166185-166691;EPI_ISL_16233784:166157-166663;EPI_ISL_16946400:166147-166653;EPI_ISL_17048206:165811-166317;EPI_ISL_17703567:166158-166664;EPI_ISL_17718458:166138-166644;EPI_ISL_18213374:166149-166655;EPI_ISL_18360394:165456-165962;EPI_ISL_18557816:166152-166658;EPI_ISL_18642365:166147-166653;EPI_ISL_18659846:166106-166612;EPI_ISL_18744049:166155-166661;EPI_ISL_19022858:166112-166618;EPI_ISL_19028778:166064-166570;EPI_ISL_19151800:166148-166654;EPI_ISL_19159116:166133-166639;EPI_ISL_19350788:164524-165054;EPI_ISL_19359677:166148-166654;EPI_ISL_19446306:166135-166641;EPI_ISL_19467692:166078-166608;JX878411.1:166780-167310;JX878425.1:166267-166797;JX878426.1:165837-166370;KC257460.1:165663-166193;KJ642612.1:164011-164541;KJ642613.1:165546-166076;KJ642616.1:167733-168263;KJ642618.1:163681-164007;KP849469.1:166096-166626;KP849471.1:166031-166561;LC831698.1:166131-166637;MN648051.1:166374-166880;MN702445.1:159414-159944;MN702446.1:165578-166108;MN702447.1:158948-159478;MN702449.1:159452-159982;MN702452.1:165636-166166;MT903338.1:166307-166813;MT903340.1:166160-166666;MT903341.1:157701-158207;MT903342.1:166197-166703;MT903347.1:168083-168613;ON568298.1:166301-166807;ON622713.1:166365-166871;ON622722.2:166153-166659;ON631963.1:166162-166668;ON649879.1:165968-166474;ON676707.1:166199-166705;ON755039.1:165790-166296;ON808413.1:166127-166633;ON880420.1:166363-166869;ON911481.2:166148-166654;ON918656.1:166170-166676;ON959133.1:166259-166765;ON959136.1:166260-166766;ON983168.1:164836-165342;OP019276.1:166147-166653;OP150924.1:166273-166779;OP160532.1:166096-166602;OP235282.1:166147-166653;OP289782.1:166151-166657;OP331335.1:166156-166662;OP382479.1:166140-166646;OP422341.1:166153-166659;OP434519.1:166129-166635;OP535324.1:166160-166666;OP535333.1:166267-166773;OP536770.1:166150-166656;OP597769.1:166184-166690;OP604566.1:166150-166656;OP612682.1:166085-166591;OP612684.1:166136-166642;OP612686.1:166138-166644;OP696841.1:163426-163932;OP879722.1:165373-165879;OQ419007.1:165367-165873;OQ419012.1:165341-165847;OQ419031.1:165345-165851;OQ419047.1:165347-165853;OQ451898.1:165237-165743;OQ451900.1:166151-166657;OQ581853.1:166159-166665;OQ581855.1:166258-166764;OQ721951.1:166177-166683;OQ721954.1:166150-166656;OR035652.1:166079-166585;OR035664.1:166083-166589;OR035702.1:166080-166586;OR146272.1:166099-166605;OR146330.1:166160-166666;OR264364.1:166154-166660;OR427332.1:166154-166660;OR459777.1:166143-166649;OR459797.1:166152-166658;OR943698.1:165786-166316;PP098589.2:166149-166655;PP496048.1:166153-166659;PP601206.1:165838-166368;PP776553.1:166073-166579;PP852971.1:166137-166643;PP852983.1:166158-166664;PP853000.1:166109-166615;PP853005.1:166117-166623;PP853007.1:166160-166666;PP853013.1:166132-166638;PP853027.1:166174-166680;PP853045.1:166125-166631;PP853048.1:166150-166656;PP860026.1:166159-166689;PP860027.1:166169-166699;PP860028.1:166108-166638;PP860031.1:166155-166685;PP860033.1:166152-166682;PQ153226.1:166130-166636;PQ178862.2:164503-165033;PQ221506.1:159673-160203;PQ221833.1:165820-166350;PQ340477.1:166117-166623;PQ340478.1:166099-166605;PQ340480.1:166177-166683;PQ340483.1:166163-166669;PQ340485.1:166146-166652;PQ340486.1:166164-166670;PQ340487.1:166188-166694;PQ340489.1:166163-166669;PQ340490.1:166115-166621;PQ340491.1:166170-166676;PQ340493.1:166154-166660;PQ340495.1:166180-166686;PQ340496.1:166215-166721;PQ368489.1:166163-166669;PQ451835.1:166220-166726;PQ451836.1:166158-166664;PQ451838.1:166208-166714;PQ451841.1:166171-166677;PQ451842.1:166117-166623 |
| OG0000092 | AY603973.1:171524-171844;AY741551.1:171558-171854;AY753185.1:171787-172107;DQ011154.1:169495-169872;DQ011155.1:169437-169778;EPI_ISL_13331598:169664-169966;EPI_ISL_13363142:169652-169954;EPI_ISL_13374487:169685-169987;EPI_ISL_13502582:169680-169982;EPI_ISL_13508471:169746-170048;EPI_ISL_13607904:169651-169953;EPI_ISL_13624509:169586-169888;EPI_ISL_13889442:169779-170081;EPI_ISL_13953610:169579-169881;EPI_ISL_14181948:169809-170111;EPI_ISL_14838587:169449-169700;EPI_ISL_15263355:169645-169947;EPI_ISL_15269699:169614-169916;EPI_ISL_15293002:169883-170185;EPI_ISL_15641561:169112-169414;EPI_ISL_15831211:169654-169956;EPI_ISL_15992098:169694-169996;EPI_ISL_16233784:169666-169968;EPI_ISL_16946400:169656-169958;EPI_ISL_17048206:169320-169622;EPI_ISL_17703567:169667-169969;EPI_ISL_17718458:169647-169937;EPI_ISL_18213374:169658-169960;EPI_ISL_18360394:168965-169267;EPI_ISL_18557816:169661-169963;EPI_ISL_18642365:169656-169958;EPI_ISL_18659846:169615-169917;EPI_ISL_18744049:169664-169966;EPI_ISL_19022858:169621-169923;EPI_ISL_19028778:169573-169875;EPI_ISL_19151800:168568-168870;EPI_ISL_19159116:169642-169944;EPI_ISL_19350788:168080-168322;EPI_ISL_19359677:169657-169959;EPI_ISL_19446306:169644-169946;EPI_ISL_19467692:169636-169941;JX878411.1:170337-170714;JX878425.1:169824-170165;JX878426.1:169450-169629;KC257460.1:169219-169527;KJ642612.1:167568-167825;KJ642613.1:169103-169432;KJ642616.1:171243-171551;KJ642618.1:167034-167345;KP849469.1:169653-170060;KP849471.1:169588-169863;LC831698.1:169640-169942;MN648051.1:169883-170185;MN702445.1:162971-163240;MN702446.1:169135-169410;MN702447.1:162505-162756;MN702449.1:163009-163278;MN702452.1:169193-169462;MT903338.1:169816-170118;MT903340.1:169669-169971;MT903341.1:161210-161512;MT903342.1:169706-170008;MT903347.1:171113-171409;ON568298.1:169810-170112;ON622713.1:169874-170176;ON622722.2:169662-169964;ON631963.1:169671-169973;ON649879.1:169477-169779;ON676707.1:169708-170010;ON755039.1:169299-169601;ON808413.1:169636-169938;ON880420.1:169872-170174;ON911481.2:169657-169959;ON918656.1:169679-169981;ON959133.1:169768-170070;ON959136.1:169769-170071;ON983168.1:168345-168647;OP019276.1:169656-169958;OP150924.1:169782-170084;OP160532.1:169605-169907;OP235282.1:169656-169958;OP289782.1:169660-169962;OP331335.1:169665-169967;OP382479.1:169649-169951;OP422341.1:169662-169964;OP434519.1:169638-169940;OP535324.1:169669-169971;OP535333.1:169776-170078;OP536770.1:169659-169961;OP597769.1:169693-169995;OP604566.1:169659-169961;OP612682.1:169594-169896;OP612684.1:169645-169947;OP612686.1:169647-169949;OP696841.1:166935-167237;OP879722.1:168882-169184;OQ419007.1:168876-169178;OQ419012.1:168850-169152;OQ419031.1:168854-169156;OQ419047.1:168856-169158;OQ451898.1:168746-169048;OQ451900.1:169660-169962;OQ581853.1:169668-169970;OQ581855.1:169767-170069;OQ721951.1:169686-169988;OQ721954.1:169659-169961;OR035652.1:169588-169890;OR035664.1:169592-169894;OR035702.1:169589-169891;OR146272.1:169608-169910;OR146330.1:169669-169971;OR264364.1:169663-169965;OR427332.1:169663-169965;OR459777.1:169652-169942;OR459797.1:169661-169963;OR943698.1:169235-169471;PP098589.2:169658-169960;PP496048.1:169662-169964;PP601206.1:169396-169647;PP776553.1:169582-169884;PP852971.1:169646-169948;PP852983.1:169667-169969;PP853000.1:169618-169890;PP853005.1:169626-169928;PP853007.1:169669-169971;PP853013.1:169641-169943;PP853027.1:169683-169985;PP853045.1:169634-169924;PP853048.1:169659-169961;PP860026.1:169666-169956;PP860027.1:169676-169978;PP860028.1:169615-169905;PP860031.1:169647-169949;PP860033.1:169659-169961;PQ153226.1:169639-169941;PQ178862.2:168059-168292;PQ221506.1:163230-163493;PQ221833.1:169377-169628;PQ340477.1:169626-169928;PQ340478.1:169608-169910;PQ340480.1:169686-169988;PQ340483.1:169672-169974;PQ340485.1:169655-169957;PQ340486.1:169673-169975;PQ340487.1:169697-169999;PQ340489.1:169672-169974;PQ340490.1:169624-169926;PQ340491.1:169679-169981;PQ340493.1:169663-169965;PQ340495.1:169689-169991;PQ340496.1:169724-170026;PQ368489.1:169672-169974;PQ451835.1:169729-170031;PQ451836.1:169667-169969;PQ451838.1:169717-170019;PQ451841.1:169680-169982;PQ451842.1:169626-169928 |
| OG0000093 | AY603973.1:174020-174469;AY741551.1:174029-174478;AY753185.1:174283-174732;DQ011154.1:172055-172504;DQ011155.1:171961-172410;EPI_ISL_13331598:172144-172593;EPI_ISL_13363142:172132-172581;EPI_ISL_13374487:172165-172614;EPI_ISL_13502582:172160-172609;EPI_ISL_13508471:172226-172675;EPI_ISL_13607904:172131-172580;EPI_ISL_13624509:172066-172515;EPI_ISL_13889442:172259-172708;EPI_ISL_13953610:172060-172509;EPI_ISL_14181948:172289-172738;EPI_ISL_14838587:171883-172332;EPI_ISL_15263355:172125-172574;EPI_ISL_15269699:172094-172543;EPI_ISL_15293002:172363-172812;EPI_ISL_15641561:171592-172041;EPI_ISL_15831211:172134-172583;EPI_ISL_15992098:172174-172623;EPI_ISL_16233784:172146-172595;EPI_ISL_16946400:172136-172585;EPI_ISL_17048206:171800-172249;EPI_ISL_17703567:172147-172596;EPI_ISL_17718458:172116-172565;EPI_ISL_18213374:172138-172587;EPI_ISL_18360394:171445-171894;EPI_ISL_18557816:172141-172590;EPI_ISL_18642365:172136-172585;EPI_ISL_18659846:172095-172544;EPI_ISL_18744049:172145-172594;EPI_ISL_19022858:172101-172550;EPI_ISL_19028778:172053-172502;EPI_ISL_19151800:171048-171497;EPI_ISL_19159116:172122-172571;EPI_ISL_19350788:170512-170961;EPI_ISL_19359677:172137-172586;EPI_ISL_19446306:172124-172573;EPI_ISL_19467692:172124-172573;JX878411.1:172896-173345;JX878425.1:172348-172797;JX878426.1:171819-172268;KC257460.1:171711-172160;KJ642612.1:170009-170458;KJ642613.1:171616-172065;KJ642616.1:173727-174176;KJ642618.1:169529-169978;KP849469.1:172243-172692;KP849471.1:172046-172495;LC831698.1:172120-172569;MN648051.1:172363-172812;MN702445.1:165423-165872;MN702446.1:171593-172042;MN702447.1:164939-165388;MN702449.1:165461-165910;MN702452.1:171645-172094;MT903338.1:172296-172745;MT903340.1:172149-172598;MT903341.1:163690-164139;MT903342.1:172186-172635;MT903347.1:173585-174034;ON568298.1:172290-172739;ON622713.1:172354-172803;ON622722.2:172142-172591;ON631963.1:172151-172600;ON649879.1:171957-172406;ON676707.1:172188-172637;ON755039.1:171779-172228;ON808413.1:172116-172565;ON880420.1:172352-172801;ON911481.2:172137-172586;ON918656.1:172159-172608;ON959133.1:172248-172697;ON959136.1:172249-172698;ON983168.1:170825-171274;OP019276.1:172136-172585;OP150924.1:172262-172711;OP160532.1:172085-172534;OP235282.1:172136-172585;OP289782.1:172140-172589;OP331335.1:172146-172595;OP382479.1:172129-172578;OP422341.1:172142-172591;OP434519.1:172118-172567;OP535324.1:172150-172599;OP535333.1:172256-172705;OP536770.1:172139-172588;OP597769.1:172173-172622;OP604566.1:172139-172588;OP612682.1:172074-172523;OP612684.1:172125-172574;OP612686.1:172127-172576;OP696841.1:169415-169864;OP879722.1:171362-171811;OQ419007.1:171356-171805;OQ419012.1:171330-171779;OQ419031.1:171334-171783;OQ419047.1:171336-171785;OQ451898.1:171226-171675;OQ451900.1:172140-172589;OQ581853.1:172148-172597;OQ581855.1:172247-172696;OQ721951.1:172166-172615;OQ721954.1:172139-172588;OR035652.1:172068-172517;OR035664.1:172072-172521;OR035702.1:172069-172518;OR146272.1:172088-172537;OR146330.1:172149-172598;OR264364.1:172143-172592;OR427332.1:172143-172592;OR459777.1:172121-172570;OR459797.1:172141-172590;OR943698.1:171654-172103;PP098589.2:172138-172587;PP496048.1:172142-172591;PP601206.1:171831-172280;PP776553.1:172062-172511;PP852971.1:172127-172576;PP852983.1:172147-172596;PP853000.1:172068-172517;PP853005.1:172106-172555;PP853007.1:172149-172598;PP853013.1:172122-172571;PP853027.1:172163-172612;PP853045.1:172103-172552;PP853048.1:172139-172588;PP860026.1:172136-172585;PP860027.1:172155-172604;PP860028.1:172085-172534;PP860031.1:172127-172576;PP860033.1:172138-172587;PQ153226.1:172119-172568;PQ178862.2:170482-170931;PQ221506.1:165683-166132;PQ221833.1:171811-172260;PQ340477.1:172106-172555;PQ340478.1:172088-172537;PQ340480.1:172166-172615;PQ340483.1:172152-172601;PQ340485.1:172135-172584;PQ340486.1:172153-172602;PQ340487.1:172177-172626;PQ340489.1:172152-172601;PQ340490.1:172104-172553;PQ340491.1:172159-172608;PQ340493.1:172143-172592;PQ340495.1:172169-172618;PQ340496.1:172204-172653;PQ368489.1:172152-172601;PQ451835.1:172209-172658;PQ451836.1:172147-172596;PQ451838.1:172197-172646;PQ451841.1:172160-172609;PQ451842.1:172106-172555 |
| OG0000094 | AY603973.1:176351-177409;AY741551.1:176360-177418;AY753185.1:176614-177672;DQ011154.1:174390-175448;DQ011155.1:174312-175370;EPI_ISL_13331598:174513-175583;EPI_ISL_13363142:174551-175621;EPI_ISL_13374487:174608-175678;EPI_ISL_13502582:174600-175670;EPI_ISL_13508471:174597-175667;EPI_ISL_13607904:174503-175573;EPI_ISL_13624509:174433-175503;EPI_ISL_13889442:174692-175762;EPI_ISL_13953610:174427-175497;EPI_ISL_14181948:174660-175730;EPI_ISL_14838587:174218-175276;EPI_ISL_15263355:174398-175468;EPI_ISL_15269699:174465-175535;EPI_ISL_15293002:174736-175806;EPI_ISL_15641561:173959-175029;EPI_ISL_15831211:174577-175641;EPI_ISL_15992098:174577-175647;EPI_ISL_16233784:174517-175587;EPI_ISL_16946400:174501-175565;EPI_ISL_17048206:174203-175273;EPI_ISL_17703567:174518-175588;EPI_ISL_17718458:174483-175547;EPI_ISL_18213374:174505-175575;EPI_ISL_18360394:173781-174851;EPI_ISL_18557816:174524-175594;EPI_ISL_18642365:174511-175581;EPI_ISL_18659846:174466-175536;EPI_ISL_18744049:174512-175576;EPI_ISL_19022858:174468-175538;EPI_ISL_19028778:174420-175490;EPI_ISL_19151800:173431-174501;EPI_ISL_19159116:174489-175559;EPI_ISL_19350788:172859-173917;EPI_ISL_19359677:174504-175574;EPI_ISL_19446306:174491-175561;EPI_ISL_19467692:174459-175517;JX878411.1:175231-176289;JX878425.1:174683-175741;JX878426.1:174154-175212;KC257460.1:174048-175106;KJ642612.1:172344-173402;KJ642613.1:173951-175009;KJ642616.1:176058-177116;KJ642618.1:171874-172932;KP849469.1:174578-175636;KP849471.1:174381-175439;LC831698.1:174487-175557;MN648051.1:174726-175796;MN702445.1:167758-168816;MN702446.1:173928-174986;MN702447.1:167278-168342;MN702449.1:167796-168854;MN702452.1:173980-175038;MT903338.1:174699-175769;MT903340.1:174516-175586;MT903341.1:166079-167149;MT903342.1:174619-175689;MT903347.1:175916-176980;ON568298.1:174661-175731;ON622713.1:174733-175803;ON622722.2:174521-175591;ON631963.1:174520-175590;ON649879.1:174326-175396;ON676707.1:174591-175661;ON755039.1:174142-175212;ON808413.1:174489-175559;ON880420.1:174723-175793;ON911481.2:174513-175583;ON918656.1:174530-175600;ON959133.1:174621-175691;ON959136.1:174616-175686;ON983168.1:173196-174266;OP019276.1:174503-175573;OP150924.1:174637-175707;OP160532.1:174452-175522;OP235282.1:174503-175573;OP289782.1:174507-175577;OP331335.1:174517-175587;OP382479.1:174498-175568;OP422341.1:174513-175583;OP434519.1:174485-175555;OP535324.1:174543-175613;OP535333.1:174625-175695;OP536770.1:174506-175576;OP597769.1:174586-175656;OP604566.1:174506-175582;OP612682.1:174413-175483;OP612684.1:174464-175534;OP612686.1:174466-175536;OP696841.1:171798-172868;OP879722.1:173765-174835;OQ419007.1:173723-174793;OQ419012.1:173697-174767;OQ419031.1:173701-174771;OQ419047.1:173703-174773;OQ451898.1:173609-174679;OQ451900.1:174523-175593;OQ581853.1:174521-175591;OQ581855.1:174630-175700;OQ721951.1:174509-175579;OQ721954.1:174542-175612;OR035652.1:174439-175509;OR035664.1:174443-175513;OR035702.1:174442-175512;OR146272.1:174459-175529;OR146330.1:174592-175662;OR264364.1:174514-175584;OR427332.1:174514-175584;OR459777.1:174488-175552;OR459797.1:174512-175582;OR943698.1:173969-175027;PP098589.2:174505-175575;PP496048.1:174521-175591;PP601206.1:174166-175224;PP776553.1:174447-175517;PP852971.1:174494-175564;PP852983.1:174518-175588;PP853000.1:174457-175527;PP853005.1:174477-175547;PP853007.1:174514-175584;PP853013.1:174511-175581;PP853027.1:174552-175622;PP853045.1:174492-175220;PP853048.1:174512-175582;PP860026.1:174519-175583;PP860027.1:174502-175566;PP860028.1:174456-175520;PP860031.1:174498-175562;PP860033.1:174495-175559;PQ153226.1:174486-175556;PQ178862.2:172835-173893;PQ221506.1:168018-169076;PQ221833.1:174146-175210;PQ340477.1:174477-175547;PQ340478.1:174467-175537;PQ340480.1:174535-175605;PQ340483.1:174527-175597;PQ340485.1:174512-175582;PQ340486.1:174526-175596;PQ340487.1:174546-175622;PQ340489.1:174529-175599;PQ340490.1:174477-175547;PQ340491.1:174540-175610;PQ340493.1:174512-175582;PQ340495.1:174546-175616;PQ340496.1:174581-175651;PQ368489.1:174519-175589;PQ451835.1:174590-175660;PQ451836.1:174530-175600;PQ451838.1:174582-175652;PQ451841.1:174547-175617;PQ451842.1:174483-175553 |
| OG0000095 | AY603973.1:179987-180241;AY741551.1:179994-180248;AY753185.1:180250-180504;DQ011154.1:178031-178243;DQ011155.1:177952-178164;EPI_ISL_13331598:178157-178411;EPI_ISL_13363142:178195-178449;EPI_ISL_13374487:178252-178506;EPI_ISL_13502582:178244-178498;EPI_ISL_13508471:178241-178495;EPI_ISL_13607904:178147-178401;EPI_ISL_13624509:178077-178331;EPI_ISL_13889442:178336-178590;EPI_ISL_13953610:178071-178325;EPI_ISL_14181948:178304-178558;EPI_ISL_14838587:177854-178066;EPI_ISL_15263355:178042-178296;EPI_ISL_15269699:178109-178363;EPI_ISL_15293002:178380-178634;EPI_ISL_15641561:177603-177857;EPI_ISL_15831211:178215-178469;EPI_ISL_15992098:178221-178475;EPI_ISL_16233784:178161-178415;EPI_ISL_16946400:178139-178393;EPI_ISL_17048206:177847-178101;EPI_ISL_17703567:178162-178416;EPI_ISL_17718458:178121-178375;EPI_ISL_18213374:178149-178403;EPI_ISL_18360394:177425-177679;EPI_ISL_18557816:178168-178422;EPI_ISL_18642365:178155-178409;EPI_ISL_18659846:178110-178364;EPI_ISL_18744049:178150-178404;EPI_ISL_19022858:178112-178366;EPI_ISL_19028778:178064-178318;EPI_ISL_19151800:177075-177329;EPI_ISL_19159116:178133-178387;EPI_ISL_19350788:176498-176710;EPI_ISL_19359677:178148-178402;EPI_ISL_19446306:178135-178389;EPI_ISL_19467692:178101-178313;JX878411.1:178875-179087;JX878425.1:178324-178536;JX878426.1:177800-178012;KC257460.1:177688-177900;KJ642612.1:175994-176206;KJ642613.1:177597-177809;KJ642616.1:179692-179946;KJ642618.1:175521-175733;KP849469.1:178218-178430;KP849471.1:178022-178234;LC831698.1:178131-178385;MN648051.1:178370-178624;MN702445.1:171392-171604;MN702446.1:177562-177774;MN702447.1:170925-171137;MN702449.1:171430-171642;MN702452.1:177616-177828;MT903338.1:178343-178597;MT903340.1:178160-178414;MT903341.1:169723-169977;MT903342.1:178263-178517;MT903347.1:179558-179812;ON568298.1:178305-178559;ON622713.1:178377-178631;ON622722.2:178165-178419;ON631963.1:178164-178418;ON649879.1:177970-178224;ON676707.1:178235-178489;ON755039.1:177786-178040;ON808413.1:178133-178387;ON880420.1:178367-178621;ON911481.2:178157-178411;ON918656.1:178174-178428;ON959133.1:178265-178519;ON959136.1:178260-178514;ON983168.1:176840-177094;OP019276.1:178147-178401;OP150924.1:178281-178535;OP160532.1:178096-178350;OP235282.1:178147-178401;OP289782.1:178151-178405;OP331335.1:178161-178415;OP382479.1:178142-178396;OP422341.1:178157-178411;OP434519.1:178129-178383;OP535324.1:178187-178441;OP535333.1:178269-178523;OP536770.1:178150-178404;OP597769.1:178230-178484;OP604566.1:178156-178410;OP612682.1:178057-178311;OP612684.1:178108-178362;OP612686.1:178110-178364;OP696841.1:175442-175696;OP879722.1:177409-177663;OQ419007.1:177367-177621;OQ419012.1:177341-177595;OQ419031.1:177345-177599;OQ419047.1:177347-177601;OQ451898.1:177253-177507;OQ451900.1:178167-178421;OQ581853.1:178165-178419;OQ581855.1:178274-178528;OQ721951.1:178153-178407;OQ721954.1:178186-178440;OR035652.1:178083-178337;OR035664.1:178087-178341;OR035702.1:178086-178340;OR146272.1:178103-178357;OR146330.1:178236-178490;OR264364.1:178158-178412;OR427332.1:178158-178412;OR459777.1:178126-178380;OR459797.1:178156-178410;OR943698.1:177619-177831;PP098589.2:178149-178403;PP496048.1:178165-178419;PP601206.1:177809-178021;PP776553.1:178091-178345;PP852971.1:178138-178392;PP852983.1:178162-178416;PP853000.1:178101-178355;PP853005.1:178121-178375;PP853007.1:178158-178412;PP853013.1:178155-178409;PP853027.1:178196-178450;PP853045.1:177961-178215;PP853048.1:178156-178410;PP860026.1:178159-178413;PP860027.1:178140-178394;PP860028.1:178095-178349;PP860031.1:178136-178390;PP860033.1:178133-178387;PQ153226.1:178130-178384;PQ178862.2:176474-176686;PQ221506.1:171667-171879;PQ221833.1:177784-177996;PQ340477.1:178121-178375;PQ340478.1:178111-178365;PQ340480.1:178179-178433;PQ340483.1:178171-178425;PQ340485.1:178156-178410;PQ340486.1:178170-178424;PQ340487.1:178196-178450;PQ340489.1:178173-178427;PQ340490.1:178121-178375;PQ340491.1:178184-178438;PQ340493.1:178156-178410;PQ340495.1:178190-178444;PQ340496.1:178225-178479;PQ368489.1:178163-178417;PQ451835.1:178234-178488;PQ451836.1:178174-178428;PQ451838.1:178226-178480;PQ451841.1:178191-178445;PQ451842.1:178127-178381 |
| OG0000096 | AY603973.1:135453-135151;AY741551.1:135476-135174;AY753185.1:135696-135394;DQ011154.1:135833-135606;DQ011155.1:135814-135587;EPI_ISL_13331598:133520-133218;EPI_ISL_13363142:133507-133205;EPI_ISL_13374487:133524-133222;EPI_ISL_13502582:133519-133217;EPI_ISL_13508471:133602-133300;EPI_ISL_13607904:133498-133196;EPI_ISL_13624509:133442-133140;EPI_ISL_13889442:133619-133317;EPI_ISL_13953610:133425-133123;EPI_ISL_14181948:133648-133346;EPI_ISL_14838587:135687-135460;EPI_ISL_15263355:133501-133199;EPI_ISL_15269699:133470-133168;EPI_ISL_15293002:133739-133437;EPI_ISL_15641561:133456-133154;EPI_ISL_15831211:133509-133207;EPI_ISL_15992098:133533-133231;EPI_ISL_16233784:133522-133220;EPI_ISL_16946400:133511-133209;EPI_ISL_17048206:133158-132856;EPI_ISL_17703567:133523-133221;EPI_ISL_17718458:133508-133206;EPI_ISL_18213374:133497-133195;EPI_ISL_18360394:133156-132854;EPI_ISL_18557816:133498-133196;EPI_ISL_18642365:133498-133196;EPI_ISL_18659846:133471-133169;EPI_ISL_18744049:133510-133208;EPI_ISL_19022858:133495-133193;EPI_ISL_19028778:133412-133110;EPI_ISL_19151800:133498-133196;EPI_ISL_19159116:133498-133196;EPI_ISL_19350788:134558-134331;EPI_ISL_19359677:133495-133193;EPI_ISL_19446306:133500-133198;EPI_ISL_19467692:135969-135742;JX878411.1:136678-136451;JX878425.1:136195-135968;JX878426.1:135645-135418;KC257460.1:135612-135385;KJ642612.1:133929-133702;KJ642613.1:135489-135262;KJ642616.1:135153-134851;KJ642618.1:133161-132934;KP849469.1:136003-135776;KP849471.1:135929-135702;LC831698.1:133496-133194;MN648051.1:133722-133420;MN702445.1:129346-129119;MN702446.1:135491-135264;MN702447.1:128859-128632;MN702449.1:129357-129130;MN702452.1:135532-135305;MT903338.1:133674-133372;MT903340.1:133508-133206;MT903341.1:125042-124740;MT903342.1:133526-133224;MT903347.1:135482-135180;ON568298.1:133649-133347;ON622713.1:133730-133428;ON622722.2:133501-133199;ON631963.1:133527-133225;ON649879.1:133333-133031;ON676707.1:133563-133261;ON755039.1:133155-132853;ON808413.1:133495-133193;ON880420.1:133728-133426;ON911481.2:133513-133211;ON918656.1:133535-133233;ON959133.1:133624-133322;ON959136.1:133625-133323;ON983168.1:132200-131898;OP019276.1:133495-133193;OP150924.1:133638-133336;OP160532.1:133461-133159;OP235282.1:133495-133193;OP289782.1:133498-133196;OP331335.1:133528-133226;OP382479.1:133505-133203;OP422341.1:133518-133216;OP434519.1:133494-133192;OP535324.1:133508-133206;OP535333.1:133615-133313;OP536770.1:133498-133196;OP597769.1:133532-133230;OP604566.1:133498-133196;OP612682.1:133433-133131;OP612684.1:133496-133194;OP612686.1:133486-133184;OP696841.1:130774-130472;OP879722.1:132724-132422;OQ419007.1:132727-132425;OQ419012.1:132698-132396;OQ419031.1:132702-132400;OQ419047.1:132704-132402;OQ451898.1:132585-132283;OQ451900.1:133498-133196;OQ581853.1:133524-133222;OQ581855.1:133605-133303;OQ721951.1:133525-133223;OQ721954.1:133495-133193;OR035652.1:133444-133142;OR035664.1:133448-133146;OR035702.1:133445-133143;OR146272.1:133464-133162;OR146330.1:133508-133206;OR264364.1:133519-133217;OR427332.1:133519-133217;OR459777.1:133510-133208;OR459797.1:133517-133215;OR943698.1:135745-135518;PP098589.2:133497-133195;PP496048.1:133501-133199;PP601206.1:135675-135448;PP776553.1:133421-133119;PP852971.1:133505-133203;PP852983.1:133513-133211;PP853000.1:133470-133168;PP853005.1:133478-133176;PP853007.1:133515-133213;PP853013.1:133496-133194;PP853027.1:133526-133224;PP853045.1:133498-133196;PP853048.1:133514-133212;PP860026.1:133513-133211;PP860027.1:133567-133265;PP860028.1:133470-133168;PP860031.1:133561-133259;PP860033.1:133556-133254;PQ153226.1:133495-133193;PQ178862.2:134535-134308;PQ221506.1:129512-129285;PQ221833.1:135678-135451;PQ340477.1:133482-133180;PQ340478.1:133464-133162;PQ340480.1:133545-133243;PQ340483.1:133528-133226;PQ340485.1:133511-133209;PQ340486.1:133529-133227;PQ340487.1:133553-133251;PQ340489.1:133528-133226;PQ340490.1:133480-133178;PQ340491.1:133535-133233;PQ340493.1:133519-133217;PQ340495.1:133545-133243;PQ340496.1:133580-133278;PQ368489.1:133511-133209;PQ451835.1:133585-133283;PQ451836.1:133523-133221;PQ451838.1:133576-133274;PQ451841.1:133536-133234;PQ451842.1:133482-133180 |
| OG0000097 | AY603973.1:135093-134845;AY741551.1:135115-134867;AY753185.1:135336-135088;DQ011154.1:135466-135302;DQ011155.1:135447-135283;EPI_ISL_13331598:133057-132893;EPI_ISL_13363142:133050-132886;EPI_ISL_13374487:133164-132901;EPI_ISL_13502582:133149-132886;EPI_ISL_13508471:133142-132978;EPI_ISL_13607904:133041-132877;EPI_ISL_13624509:132985-132821;EPI_ISL_13889442:133259-132996;EPI_ISL_13953610:132958-132794;EPI_ISL_14181948:133288-133022;EPI_ISL_14838587:135320-135156;EPI_ISL_15263355:133041-132877;EPI_ISL_15269699:133010-132846;EPI_ISL_15293002:133379-133119;EPI_ISL_15641561:132999-132835;EPI_ISL_15831211:133048-132884;EPI_ISL_15992098:133063-132899;EPI_ISL_16233784:133062-132898;EPI_ISL_16946400:133048-132884;EPI_ISL_17048206:132698-132534;EPI_ISL_17703567:133063-132899;EPI_ISL_17718458:133041-132877;EPI_ISL_18213374:133137-132874;EPI_ISL_18360394:132699-132535;EPI_ISL_18557816:133138-132875;EPI_ISL_18642365:133041-132877;EPI_ISL_18659846:133011-132847;EPI_ISL_18744049:133043-132879;EPI_ISL_19022858:133038-132874;EPI_ISL_19028778:132955-132791;EPI_ISL_19151800:133138-132875;EPI_ISL_19159116:133041-132877;EPI_ISL_19350788:134191-134027;EPI_ISL_19359677:133038-132874;EPI_ISL_19446306:133033-132869;EPI_ISL_19467692:135602-135438;JX878411.1:136311-136147;JX878425.1:135828-135664;JX878426.1:135278-135114;KC257460.1:135245-135081;KJ642612.1:133562-133398;KJ642613.1:135122-134958;KJ642616.1:134793-134545;KJ642618.1:132794-132630;KP849469.1:135636-135472;KP849471.1:135562-135398;LC831698.1:133039-132875;MN648051.1:133261-133097;MN702445.1:128979-128815;MN702446.1:135124-134960;MN702447.1:128492-128328;MN702449.1:128990-128826;MN702452.1:135165-135001;MT903338.1:133204-133040;MT903340.1:133041-132877;MT903341.1:124574-124410;MT903342.1:133056-132892;MT903347.1:135122-134874;ON568298.1:133289-133023;ON622713.1:133273-133109;ON622722.2:133044-132880;ON631963.1:133064-132900;ON649879.1:132973-132710;ON676707.1:133193-132930;ON755039.1:132704-132540;ON808413.1:133044-132880;ON880420.1:133271-133107;ON911481.2:133061-132897;ON918656.1:133175-132912;ON959133.1:133264-133001;ON959136.1:133265-133002;ON983168.1:131739-131575;OP019276.1:133038-132874;OP150924.1:133278-133015;OP160532.1:133004-132840;OP235282.1:133038-132874;OP289782.1:133041-132877;OP331335.1:133058-132894;OP382479.1:133045-132881;OP422341.1:133058-132894;OP434519.1:133037-132873;OP535324.1:133138-132875;OP535333.1:133148-132984;OP536770.1:133040-132876;OP597769.1:133062-132898;OP604566.1:133041-132877;OP612682.1:132966-132802;OP612684.1:133028-132864;OP612686.1:133126-132866;OP696841.1:130414-130151;OP879722.1:132264-132100;OQ419007.1:132367-132110;OQ419012.1:132241-132077;OQ419031.1:132245-132081;OQ419047.1:132247-132083;OQ451898.1:132225-131962;OQ451900.1:133138-132875;OQ581853.1:133164-132901;OQ581855.1:133245-132982;OQ721951.1:133079-132915;OQ721954.1:133135-132872;OR035652.1:133084-132824;OR035664.1:132988-132824;OR035702.1:132988-132824;OR146272.1:133004-132840;OR146330.1:133148-132885;OR264364.1:133059-132895;OR427332.1:133059-132895;OR459777.1:133043-132879;OR459797.1:133057-132893;OR943698.1:135378-135214;PP098589.2:133040-132876;PP496048.1:133044-132880;PP601206.1:135308-135144;PP776553.1:133061-132801;PP852971.1:133038-132874;PP852983.1:133143-132880;PP853000.1:133005-132841;PP853005.1:133013-132849;PP853007.1:133145-132882;PP853013.1:133019-132855;PP853027.1:133050-132886;PP853045.1:133031-132867;PP853048.1:133050-132886;PP860026.1:133048-132884;PP860027.1:133197-132910;PP860028.1:133100-132849;PP860031.1:133067-132903;PP860033.1:133073-132909;PQ153226.1:133038-132874;PQ178862.2:134168-134004;PQ221506.1:129145-128981;PQ221833.1:135311-135147;PQ340477.1:133021-132857;PQ340478.1:133006-132842;PQ340480.1:133085-132921;PQ340483.1:133168-132905;PQ340485.1:133151-132891;PQ340486.1:133169-132906;PQ340487.1:133095-132931;PQ340489.1:133070-132906;PQ340490.1:133120-132857;PQ340491.1:133175-132912;PQ340493.1:133055-132891;PQ340495.1:133085-132921;PQ340496.1:133220-132954;PQ368489.1:133041-132877;PQ451835.1:133225-132962;PQ451836.1:133163-132894;PQ451838.1:133109-132945;PQ451841.1:133069-132905;PQ451842.1:133021-132857 |
| OG0000098 | AY603973.1:125274-124141;AY741551.1:125296-124163;AY753185.1:125517-124384;DQ011154.1:125731-124598;DQ011155.1:125712-124579;EPI_ISL_13331598:123330-122197;EPI_ISL_13363142:123323-122190;EPI_ISL_13374487:123338-122205;EPI_ISL_13502582:123323-122190;EPI_ISL_13508471:123415-122282;EPI_ISL_13607904:123314-122181;EPI_ISL_13624509:123258-122125;EPI_ISL_13889442:123433-122300;EPI_ISL_13953610:123231-122098;EPI_ISL_14181948:123459-122326;EPI_ISL_14838587:125585-124452;EPI_ISL_15263355:123314-122181;EPI_ISL_15269699:123283-122150;EPI_ISL_15293002:123556-122423;EPI_ISL_15641561:123272-122139;EPI_ISL_15831211:123321-122188;EPI_ISL_15992098:123336-122203;EPI_ISL_16233784:123332-122199;EPI_ISL_16946400:123321-122188;EPI_ISL_17048206:122971-121838;EPI_ISL_17703567:123336-122203;EPI_ISL_17718458:123314-122181;EPI_ISL_18213374:123311-122178;EPI_ISL_18360394:122972-121839;EPI_ISL_18557816:123312-122179;EPI_ISL_18642365:123314-122181;EPI_ISL_18659846:123284-122151;EPI_ISL_18744049:123316-122183;EPI_ISL_19022858:123311-122178;EPI_ISL_19028778:123228-122095;EPI_ISL_19151800:123312-122179;EPI_ISL_19159116:123314-122181;EPI_ISL_19350788:124456-123323;EPI_ISL_19359677:123311-122178;EPI_ISL_19446306:123306-122173;EPI_ISL_19467692:125867-124734;JX878411.1:126576-125443;JX878425.1:126093-124960;JX878426.1:125543-124410;KC257460.1:125510-124377;KJ642612.1:123827-122694;KJ642613.1:125387-124254;KJ642616.1:124974-123841;KJ642618.1:123058-121925;KP849469.1:125901-124768;KP849471.1:125827-124694;LC831698.1:123312-122179;MN648051.1:123534-122401;MN702445.1:119244-118111;MN702446.1:125389-124256;MN702447.1:118757-117624;MN702449.1:119255-118122;MN702452.1:125430-124297;MT903338.1:123477-122344;MT903340.1:123314-122181;MT903341.1:114847-113714;MT903342.1:123329-122196;MT903347.1:125303-124170;ON568298.1:123460-122327;ON622713.1:123546-122413;ON622722.2:123317-122184;ON631963.1:123337-122204;ON649879.1:123147-122014;ON676707.1:123367-122234;ON755039.1:122977-121844;ON808413.1:123317-122184;ON880420.1:123544-122411;ON911481.2:123334-122201;ON918656.1:123349-122216;ON959133.1:123438-122305;ON959136.1:123439-122306;ON983168.1:122012-120879;OP019276.1:123311-122178;OP150924.1:123452-122319;OP160532.1:123277-122144;OP235282.1:123311-122178;OP289782.1:123314-122181;OP331335.1:123331-122198;OP382479.1:123318-122185;OP422341.1:123331-122198;OP434519.1:123310-122177;OP535324.1:123312-122179;OP535333.1:123421-122288;OP536770.1:123313-122180;OP597769.1:123335-122202;OP604566.1:123314-122181;OP612682.1:123239-122106;OP612684.1:123301-122168;OP612686.1:123303-122170;OP696841.1:120588-119455;OP879722.1:122537-121404;OQ419007.1:122547-121414;OQ419012.1:122514-121381;OQ419031.1:122518-121385;OQ419047.1:122520-121387;OQ451898.1:122399-121266;OQ451900.1:123312-122179;OQ581853.1:123338-122205;OQ581855.1:123419-122286;OQ721951.1:123352-122219;OQ721954.1:123309-122176;OR035652.1:123261-122128;OR035664.1:123261-122128;OR035702.1:123261-122128;OR146272.1:123277-122144;OR146330.1:123322-122189;OR264364.1:123332-122199;OR427332.1:123332-122199;OR459777.1:123316-122183;OR459797.1:123330-122197;OR943698.1:125643-124510;PP098589.2:123313-122180;PP496048.1:123317-122184;PP601206.1:125573-124440;PP776553.1:123238-122105;PP852971.1:123311-122178;PP852983.1:123317-122184;PP853000.1:123278-122145;PP853005.1:123286-122153;PP853007.1:123319-122186;PP853013.1:123292-122159;PP853027.1:123323-122190;PP853045.1:123304-122171;PP853048.1:123323-122190;PP860026.1:123321-122188;PP860027.1:123347-122214;PP860028.1:123286-122153;PP860031.1:123340-122207;PP860033.1:123346-122213;PQ153226.1:123311-122178;PQ178862.2:124433-123300;PQ221506.1:119410-118277;PQ221833.1:125576-124443;PQ340477.1:123294-122161;PQ340478.1:123279-122146;PQ340480.1:123358-122225;PQ340483.1:123342-122209;PQ340485.1:123328-122195;PQ340486.1:123343-122210;PQ340487.1:123368-122235;PQ340489.1:123343-122210;PQ340490.1:123294-122161;PQ340491.1:123349-122216;PQ340493.1:123328-122195;PQ340495.1:123358-122225;PQ340496.1:123391-122258;PQ368489.1:123314-122181;PQ451835.1:123399-122266;PQ451836.1:123331-122198;PQ451838.1:123382-122249;PQ451841.1:123342-122209;PQ451842.1:123294-122161 |
| OG0000099 | AY603973.1:123705-123433;AY741551.1:123727-123455;AY753185.1:123948-123676;DQ011154.1:124162-123890;DQ011155.1:124143-123871;EPI_ISL_13331598:121761-121489;EPI_ISL_13363142:121754-121482;EPI_ISL_13374487:121769-121497;EPI_ISL_13502582:121754-121482;EPI_ISL_13508471:121846-121574;EPI_ISL_13607904:121745-121473;EPI_ISL_13624509:121689-121417;EPI_ISL_13889442:121864-121592;EPI_ISL_13953610:121662-121390;EPI_ISL_14181948:121890-121618;EPI_ISL_14838587:124016-123744;EPI_ISL_15263355:121745-121473;EPI_ISL_15269699:121714-121442;EPI_ISL_15293002:121987-121715;EPI_ISL_15641561:121703-121431;EPI_ISL_15831211:121752-121480;EPI_ISL_15992098:121767-121495;EPI_ISL_16233784:121763-121491;EPI_ISL_16946400:121752-121480;EPI_ISL_17048206:121402-121130;EPI_ISL_17703567:121767-121495;EPI_ISL_17718458:121745-121473;EPI_ISL_18213374:121742-121470;EPI_ISL_18360394:121403-121131;EPI_ISL_18557816:121743-121471;EPI_ISL_18642365:121745-121473;EPI_ISL_18659846:121715-121443;EPI_ISL_18744049:121747-121475;EPI_ISL_19022858:121742-121470;EPI_ISL_19028778:121659-121387;EPI_ISL_19151800:121743-121471;EPI_ISL_19159116:121745-121473;EPI_ISL_19350788:122887-122615;EPI_ISL_19359677:121742-121470;EPI_ISL_19446306:121737-121465;EPI_ISL_19467692:124298-124026;JX878411.1:125007-124735;JX878425.1:124524-124252;JX878426.1:123974-123702;KC257460.1:123941-123669;KJ642612.1:122258-121986;KJ642613.1:123818-123546;KJ642616.1:123405-123133;KJ642618.1:121489-121217;KP849469.1:124332-124060;KP849471.1:124258-123986;LC831698.1:121743-121471;MN648051.1:121965-121693;MN702445.1:117675-117403;MN702446.1:123820-123548;MN702447.1:117188-116916;MN702449.1:117686-117414;MN702452.1:123861-123589;MT903338.1:121908-121636;MT903340.1:121745-121473;MT903341.1:113278-113006;MT903342.1:121760-121488;MT903347.1:123734-123462;ON568298.1:121891-121619;ON622713.1:121977-121705;ON622722.2:121748-121476;ON631963.1:121768-121496;ON649879.1:121578-121306;ON676707.1:121798-121526;ON755039.1:121408-121136;ON808413.1:121748-121476;ON880420.1:121975-121703;ON911481.2:121765-121493;ON918656.1:121780-121508;ON959133.1:121869-121597;ON959136.1:121870-121598;ON983168.1:120443-120171;OP019276.1:121742-121470;OP150924.1:121883-121611;OP160532.1:121708-121436;OP235282.1:121742-121470;OP289782.1:121745-121473;OP331335.1:121762-121490;OP382479.1:121749-121477;OP422341.1:121762-121490;OP434519.1:121741-121469;OP535324.1:121743-121471;OP535333.1:121852-121580;OP536770.1:121744-121472;OP597769.1:121766-121494;OP604566.1:121745-121473;OP612682.1:121670-121398;OP612684.1:121732-121460;OP612686.1:121734-121462;OP696841.1:119019-118747;OP879722.1:120968-120696;OQ419007.1:120978-120706;OQ419012.1:120945-120673;OQ419031.1:120949-120677;OQ419047.1:120951-120679;OQ451898.1:120830-120558;OQ451900.1:121743-121471;OQ581853.1:121769-121497;OQ581855.1:121850-121578;OQ721951.1:121783-121511;OQ721954.1:121740-121468;OR035652.1:121692-121420;OR035664.1:121692-121420;OR035702.1:121692-121420;OR146272.1:121708-121436;OR146330.1:121753-121481;OR264364.1:121763-121491;OR427332.1:121763-121491;OR459777.1:121747-121475;OR459797.1:121761-121489;OR943698.1:124074-123802;PP098589.2:121744-121472;PP496048.1:121748-121476;PP601206.1:124004-123732;PP776553.1:121669-121397;PP852971.1:121742-121470;PP852983.1:121748-121476;PP853000.1:121709-121437;PP853005.1:121717-121445;PP853007.1:121750-121478;PP853013.1:121723-121451;PP853027.1:121754-121482;PP853045.1:121735-121463;PP853048.1:121754-121482;PP860026.1:121752-121480;PP860027.1:121778-121506;PP860028.1:121717-121445;PP860031.1:121771-121499;PP860033.1:121777-121505;PQ153226.1:121742-121470;PQ178862.2:122864-122592;PQ221506.1:117841-117569;PQ221833.1:124007-123735;PQ340477.1:121725-121453;PQ340478.1:121710-121438;PQ340480.1:121789-121517;PQ340483.1:121773-121501;PQ340485.1:121759-121487;PQ340486.1:121774-121502;PQ340487.1:121799-121527;PQ340489.1:121774-121502;PQ340490.1:121725-121453;PQ340491.1:121780-121508;PQ340493.1:121759-121487;PQ340495.1:121789-121517;PQ340496.1:121822-121550;PQ368489.1:121745-121473;PQ451835.1:121830-121558;PQ451836.1:121762-121490;PQ451838.1:121813-121541;PQ451841.1:121773-121501;PQ451842.1:121725-121453 |
| OG0000100 | AY603973.1:123327-123115;AY741551.1:123349-123137;AY753185.1:123570-123358;DQ011154.1:123784-123572;DQ011155.1:123765-123553;EPI_ISL_13331598:121383-121171;EPI_ISL_13363142:121376-121164;EPI_ISL_13374487:121391-121179;EPI_ISL_13502582:121376-121164;EPI_ISL_13508471:121468-121256;EPI_ISL_13607904:121367-121155;EPI_ISL_13624509:121311-121099;EPI_ISL_13889442:121486-121274;EPI_ISL_13953610:121284-121072;EPI_ISL_14181948:121512-121300;EPI_ISL_14838587:123638-123426;EPI_ISL_15263355:121367-121155;EPI_ISL_15269699:121336-121124;EPI_ISL_15293002:121609-121397;EPI_ISL_15641561:121325-121113;EPI_ISL_15831211:121374-121162;EPI_ISL_15992098:121389-121177;EPI_ISL_16233784:121385-121173;EPI_ISL_16946400:121374-121162;EPI_ISL_17048206:121024-120812;EPI_ISL_17703567:121389-121177;EPI_ISL_17718458:121367-121155;EPI_ISL_18213374:121364-121152;EPI_ISL_18360394:121025-120813;EPI_ISL_18557816:121365-121153;EPI_ISL_18642365:121367-121155;EPI_ISL_18659846:121337-121125;EPI_ISL_18744049:121369-121157;EPI_ISL_19022858:121364-121152;EPI_ISL_19028778:121281-121069;EPI_ISL_19151800:121365-121153;EPI_ISL_19159116:121367-121155;EPI_ISL_19350788:122509-122297;EPI_ISL_19359677:121364-121152;EPI_ISL_19446306:121359-121147;EPI_ISL_19467692:123920-123708;JX878411.1:124629-124417;JX878425.1:124146-123934;JX878426.1:123596-123384;KC257460.1:123563-123351;KJ642612.1:121880-121668;KJ642613.1:123440-123228;KJ642616.1:123027-122815;KJ642618.1:121111-120899;KP849469.1:123954-123742;KP849471.1:123880-123668;LC831698.1:121365-121153;MN648051.1:121587-121375;MN702445.1:117297-117085;MN702446.1:123442-123230;MN702447.1:116810-116598;MN702449.1:117308-117096;MN702452.1:123483-123271;MT903338.1:121530-121318;MT903340.1:121367-121155;MT903341.1:112900-112688;MT903342.1:121382-121170;MT903347.1:123356-123144;ON568298.1:121513-121301;ON622713.1:121599-121387;ON622722.2:121370-121158;ON631963.1:121390-121178;ON649879.1:121200-120988;ON676707.1:121420-121208;ON755039.1:121030-120818;ON808413.1:121370-121158;ON880420.1:121597-121385;ON911481.2:121387-121175;ON918656.1:121402-121190;ON959133.1:121491-121279;ON959136.1:121492-121280;ON983168.1:120065-119853;OP019276.1:121364-121152;OP150924.1:121505-121293;OP160532.1:121330-121118;OP235282.1:121364-121152;OP289782.1:121367-121155;OP331335.1:121384-121172;OP382479.1:121371-121159;OP422341.1:121384-121172;OP434519.1:121363-121151;OP535324.1:121365-121153;OP535333.1:121474-121262;OP536770.1:121366-121154;OP597769.1:121388-121176;OP604566.1:121367-121155;OP612682.1:121292-121080;OP612684.1:121354-121142;OP612686.1:121356-121144;OP696841.1:118641-118429;OP879722.1:120590-120378;OQ419007.1:120600-120388;OQ419012.1:120567-120355;OQ419031.1:120571-120359;OQ419047.1:120573-120361;OQ451898.1:120452-120240;OQ451900.1:121365-121153;OQ581853.1:121391-121179;OQ581855.1:121472-121260;OQ721951.1:121405-121193;OQ721954.1:121362-121150;OR035652.1:121314-121102;OR035664.1:121314-121102;OR035702.1:121314-121102;OR146272.1:121330-121118;OR146330.1:121375-121163;OR264364.1:121385-121173;OR427332.1:121385-121173;OR459777.1:121369-121157;OR459797.1:121383-121171;OR943698.1:123696-123484;PP098589.2:121366-121154;PP496048.1:121370-121158;PP601206.1:123626-123414;PP776553.1:121291-121079;PP852971.1:121364-121152;PP852983.1:121370-121158;PP853000.1:121331-121119;PP853005.1:121339-121127;PP853007.1:121372-121160;PP853013.1:121345-121133;PP853027.1:121376-121164;PP853045.1:121357-121145;PP853048.1:121376-121164;PP860026.1:121374-121162;PP860027.1:121400-121188;PP860028.1:121339-121127;PP860031.1:121393-121181;PP860033.1:121399-121187;PQ153226.1:121364-121152;PQ178862.2:122486-122274;PQ221506.1:117463-117251;PQ221833.1:123629-123417;PQ340477.1:121347-121135;PQ340478.1:121332-121120;PQ340480.1:121411-121199;PQ340483.1:121395-121183;PQ340485.1:121381-121169;PQ340486.1:121396-121184;PQ340487.1:121421-121209;PQ340489.1:121396-121184;PQ340490.1:121347-121135;PQ340491.1:121402-121190;PQ340493.1:121381-121169;PQ340495.1:121411-121199;PQ340496.1:121444-121232;PQ368489.1:121367-121155;PQ451835.1:121452-121240;PQ451836.1:121384-121172;PQ451838.1:121435-121223;PQ451841.1:121395-121183;PQ451842.1:121347-121135 |
| OG0000101 | AY603973.1:110766-110092;AY741551.1:110770-110096;AY753185.1:111009-110335;DQ011154.1:111241-110567;DQ011155.1:111222-110548;EPI_ISL_13331598:108840-108166;EPI_ISL_13363142:108833-108159;EPI_ISL_13374487:108848-108174;EPI_ISL_13502582:108833-108159;EPI_ISL_13508471:108925-108251;EPI_ISL_13607904:108824-108150;EPI_ISL_13624509:108768-108094;EPI_ISL_13889442:108943-108269;EPI_ISL_13953610:108741-108067;EPI_ISL_14181948:108969-108295;EPI_ISL_14838587:111095-110421;EPI_ISL_15263355:108824-108150;EPI_ISL_15269699:108793-108119;EPI_ISL_15293002:109066-108392;EPI_ISL_15641561:108782-108108;EPI_ISL_15831211:108831-108157;EPI_ISL_15992098:108846-108172;EPI_ISL_16233784:108842-108168;EPI_ISL_16946400:108831-108157;EPI_ISL_17048206:108481-107807;EPI_ISL_17703567:108846-108172;EPI_ISL_17718458:108824-108150;EPI_ISL_18213374:108821-108147;EPI_ISL_18360394:108482-107808;EPI_ISL_18557816:108822-108148;EPI_ISL_18642365:108824-108150;EPI_ISL_18659846:108794-108120;EPI_ISL_18744049:108826-108152;EPI_ISL_19022858:108821-108147;EPI_ISL_19028778:108738-108064;EPI_ISL_19151800:108822-108148;EPI_ISL_19159116:108824-108150;EPI_ISL_19350788:109966-109292;EPI_ISL_19359677:108821-108147;EPI_ISL_19446306:108816-108142;EPI_ISL_19467692:111377-110703;JX878411.1:112086-111412;JX878425.1:111603-110929;JX878426.1:111053-110379;KC257460.1:111020-110346;KJ642612.1:109337-108663;KJ642613.1:110897-110223;KJ642616.1:110460-109786;KJ642618.1:108568-107894;KP849469.1:111411-110737;KP849471.1:111337-110663;LC831698.1:108822-108148;MN648051.1:109044-108370;MN702445.1:104754-104080;MN702446.1:110899-110225;MN702447.1:104267-103593;MN702449.1:104765-104091;MN702452.1:110940-110266;MT903338.1:108987-108313;MT903340.1:108824-108150;MT903341.1:100357-99683;MT903342.1:108839-108165;MT903347.1:110777-110103;ON568298.1:108970-108296;ON622713.1:109056-108382;ON622722.2:108827-108153;ON631963.1:108847-108173;ON649879.1:108657-107983;ON676707.1:108877-108203;ON755039.1:108487-107813;ON808413.1:108827-108153;ON880420.1:109054-108380;ON911481.2:108844-108170;ON918656.1:108859-108185;ON959133.1:108948-108274;ON959136.1:108949-108275;ON983168.1:107522-106848;OP019276.1:108821-108147;OP150924.1:108962-108288;OP160532.1:108787-108113;OP235282.1:108821-108147;OP289782.1:108824-108150;OP331335.1:108841-108167;OP382479.1:108828-108154;OP422341.1:108841-108167;OP434519.1:108820-108146;OP535324.1:108822-108148;OP535333.1:108931-108257;OP536770.1:108823-108149;OP597769.1:108845-108171;OP604566.1:108824-108150;OP612682.1:108749-108075;OP612684.1:108812-108138;OP612686.1:108813-108139;OP696841.1:106098-105424;OP879722.1:108047-107373;OQ419007.1:108057-107383;OQ419012.1:108024-107350;OQ419031.1:108028-107354;OQ419047.1:108030-107356;OQ451898.1:107909-107235;OQ451900.1:108822-108148;OQ581853.1:108848-108174;OQ581855.1:108929-108255;OQ721951.1:108862-108188;OQ721954.1:108819-108145;OR035652.1:108771-108097;OR035664.1:108771-108097;OR035702.1:108771-108097;OR146272.1:108787-108113;OR146330.1:108832-108158;OR264364.1:108842-108168;OR427332.1:108842-108168;OR459777.1:108826-108152;OR459797.1:108841-108167;OR943698.1:111153-110479;PP098589.2:108823-108149;PP496048.1:108827-108153;PP601206.1:111083-110409;PP776553.1:108748-108074;PP852971.1:108821-108147;PP852983.1:108827-108153;PP853000.1:108788-108114;PP853005.1:108796-108122;PP853007.1:108829-108155;PP853013.1:108802-108128;PP853027.1:108833-108159;PP853045.1:108814-108140;PP853048.1:108833-108159;PP860026.1:108831-108157;PP860027.1:108857-108183;PP860028.1:108796-108122;PP860031.1:108850-108176;PP860033.1:108856-108182;PQ153226.1:108821-108147;PQ178862.2:109943-109269;PQ221506.1:104920-104246;PQ221833.1:111086-110412;PQ340477.1:108804-108130;PQ340478.1:108789-108115;PQ340480.1:108868-108194;PQ340483.1:108852-108178;PQ340485.1:108838-108164;PQ340486.1:108853-108179;PQ340487.1:108878-108204;PQ340489.1:108853-108179;PQ340490.1:108804-108130;PQ340491.1:108859-108185;PQ340493.1:108838-108164;PQ340495.1:108868-108194;PQ340496.1:108901-108227;PQ368489.1:108824-108150;PQ451835.1:108909-108235;PQ451836.1:108841-108167;PQ451838.1:108892-108218;PQ451841.1:108852-108178;PQ451842.1:108804-108130 |
| OG0000102 | AY603973.1:103689-102775;AY741551.1:103693-102779;AY753185.1:103932-103018;DQ011154.1:104163-103249;DQ011155.1:104144-103230;EPI_ISL_13331598:101763-100849;EPI_ISL_13363142:101756-100842;EPI_ISL_13374487:101771-100857;EPI_ISL_13502582:101756-100842;EPI_ISL_13508471:101848-100934;EPI_ISL_13607904:101747-100833;EPI_ISL_13624509:101691-100777;EPI_ISL_13889442:101866-100952;EPI_ISL_13953610:101664-100750;EPI_ISL_14181948:101892-100978;EPI_ISL_14838587:104017-103103;EPI_ISL_15263355:101747-100833;EPI_ISL_15269699:101716-100802;EPI_ISL_15293002:101989-101075;EPI_ISL_15641561:101705-100791;EPI_ISL_15831211:101754-100840;EPI_ISL_15992098:101769-100855;EPI_ISL_16233784:101765-100851;EPI_ISL_16946400:101754-100840;EPI_ISL_17048206:101404-100490;EPI_ISL_17703567:101769-100855;EPI_ISL_17718458:101747-100833;EPI_ISL_18213374:101744-100830;EPI_ISL_18360394:101405-100491;EPI_ISL_18557816:101745-100831;EPI_ISL_18642365:101747-100833;EPI_ISL_18659846:101717-100803;EPI_ISL_18744049:101749-100835;EPI_ISL_19022858:101744-100830;EPI_ISL_19028778:101661-100747;EPI_ISL_19151800:101745-100831;EPI_ISL_19159116:101747-100833;EPI_ISL_19350788:102889-101975;EPI_ISL_19359677:101744-100830;EPI_ISL_19446306:101739-100825;EPI_ISL_19467692:104300-103386;JX878411.1:105008-104094;JX878425.1:104525-103611;JX878426.1:103974-103060;KC257460.1:103942-103028;KJ642612.1:102260-101346;KJ642613.1:103819-102905;KJ642616.1:103383-102469;KJ642618.1:101490-100576;KP849469.1:104333-103419;KP849471.1:104259-103345;LC831698.1:101745-100831;MN648051.1:101967-101053;MN702445.1:97676-96762;MN702446.1:103821-102907;MN702447.1:97189-96275;MN702449.1:97687-96773;MN702452.1:103862-102948;MT903338.1:101910-100996;MT903340.1:101747-100833;MT903341.1:93280-92366;MT903342.1:101762-100848;MT903347.1:103700-102786;ON568298.1:101893-100979;ON622713.1:101979-101065;ON622722.2:101750-100836;ON631963.1:101770-100856;ON649879.1:101580-100666;ON676707.1:101800-100886;ON755039.1:101410-100496;ON808413.1:101750-100836;ON880420.1:101977-101063;ON911481.2:101767-100853;ON918656.1:101782-100868;ON959133.1:101871-100957;ON959136.1:101872-100958;ON983168.1:100445-99531;OP019276.1:101744-100830;OP150924.1:101885-100971;OP160532.1:101710-100796;OP235282.1:101744-100830;OP289782.1:101747-100833;OP331335.1:101764-100850;OP382479.1:101751-100837;OP422341.1:101764-100850;OP434519.1:101743-100829;OP535324.1:101745-100831;OP535333.1:101854-100940;OP536770.1:101746-100832;OP597769.1:101768-100854;OP604566.1:101747-100833;OP612682.1:101672-100758;OP612684.1:101735-100821;OP612686.1:101736-100822;OP696841.1:99021-98107;OP879722.1:100970-100056;OQ419007.1:100980-100066;OQ419012.1:100947-100033;OQ419031.1:100951-100037;OQ419047.1:100953-100039;OQ451898.1:100832-99918;OQ451900.1:101745-100831;OQ581853.1:101771-100857;OQ581855.1:101852-100938;OQ721951.1:101785-100871;OQ721954.1:101742-100828;OR035652.1:101694-100780;OR035664.1:101694-100780;OR035702.1:101694-100780;OR146272.1:101710-100796;OR146330.1:101755-100841;OR264364.1:101765-100851;OR427332.1:101765-100851;OR459777.1:101749-100835;OR459797.1:101764-100850;OR943698.1:104075-103161;PP098589.2:101746-100832;PP496048.1:101750-100836;PP601206.1:104006-103092;PP776553.1:101671-100757;PP852971.1:101744-100830;PP852983.1:101750-100836;PP853000.1:101711-100797;PP853005.1:101719-100805;PP853007.1:101752-100838;PP853013.1:101725-100811;PP853027.1:101756-100842;PP853045.1:101737-100823;PP853048.1:101756-100842;PP860026.1:101754-100840;PP860027.1:101779-100865;PP860028.1:101719-100805;PP860031.1:101773-100859;PP860033.1:101778-100864;PQ153226.1:101744-100830;PQ178862.2:102866-101952;PQ221506.1:97842-96928;PQ221833.1:104008-103094;PQ340477.1:101727-100813;PQ340478.1:101712-100798;PQ340480.1:101791-100877;PQ340483.1:101775-100861;PQ340485.1:101761-100847;PQ340486.1:101776-100862;PQ340487.1:101801-100887;PQ340489.1:101776-100862;PQ340490.1:101727-100813;PQ340491.1:101782-100868;PQ340493.1:101761-100847;PQ340495.1:101791-100877;PQ340496.1:101824-100910;PQ368489.1:101747-100833;PQ451835.1:101832-100918;PQ451836.1:101764-100850;PQ451838.1:101815-100901;PQ451841.1:101775-100861;PQ451842.1:101727-100813 |
| OG0000103 | AY603973.1:74892-73777;AY741551.1:74896-73781;AY753185.1:75135-74020;DQ011154.1:75353-74238;DQ011155.1:75334-74216;EPI_ISL_13331598:72962-71847;EPI_ISL_13363142:72955-71840;EPI_ISL_13374487:72970-71855;EPI_ISL_13502582:72955-71840;EPI_ISL_13508471:73047-71932;EPI_ISL_13607904:72946-71831;EPI_ISL_13624509:72890-71775;EPI_ISL_13889442:73065-71950;EPI_ISL_13953610:72882-71767;EPI_ISL_14181948:73091-71976;EPI_ISL_14838587:75207-74092;EPI_ISL_15263355:72946-71831;EPI_ISL_15269699:72915-71800;EPI_ISL_15293002:73188-72073;EPI_ISL_15641561:72904-71789;EPI_ISL_15831211:72953-71838;EPI_ISL_15992098:72968-71853;EPI_ISL_16233784:72964-71849;EPI_ISL_16946400:72953-71838;EPI_ISL_17048206:72603-71488;EPI_ISL_17703567:72968-71853;EPI_ISL_17718458:72946-71831;EPI_ISL_18213374:72943-71828;EPI_ISL_18360394:72604-71489;EPI_ISL_18557816:72944-71829;EPI_ISL_18642365:72946-71831;EPI_ISL_18659846:72916-71801;EPI_ISL_18744049:72948-71833;EPI_ISL_19022858:72943-71828;EPI_ISL_19028778:72860-71745;EPI_ISL_19151800:72944-71829;EPI_ISL_19159116:72946-71831;EPI_ISL_19350788:74079-72964;EPI_ISL_19359677:72943-71828;EPI_ISL_19446306:72938-71823;EPI_ISL_19467692:75490-74375;JX878411.1:76198-75083;JX878425.1:75715-74600;JX878426.1:75164-74049;KC257460.1:75132-74014;KJ642612.1:73450-72335;KJ642613.1:75009-73894;KJ642616.1:74586-73471;KJ642618.1:72617-71502;KP849469.1:75523-74408;KP849471.1:75449-74334;LC831698.1:72944-71829;MN648051.1:73166-72051;MN702445.1:68866-67751;MN702446.1:75011-73896;MN702447.1:68379-67264;MN702449.1:68877-67762;MN702452.1:75052-73937;MT903338.1:73109-71994;MT903340.1:72946-71831;MT903341.1:64479-63364;MT903342.1:72961-71846;MT903347.1:74903-73788;ON568298.1:73092-71977;ON622713.1:73178-72063;ON622722.2:72949-71834;ON631963.1:72969-71854;ON649879.1:72779-71664;ON676707.1:72999-71884;ON755039.1:72609-71494;ON808413.1:72949-71834;ON880420.1:73176-72061;ON911481.2:72966-71851;ON918656.1:72981-71866;ON959133.1:73070-71955;ON959136.1:73071-71956;ON983168.1:71645-70530;OP019276.1:72943-71828;OP150924.1:73084-71969;OP160532.1:72909-71794;OP235282.1:72943-71828;OP289782.1:72946-71831;OP331335.1:72963-71848;OP382479.1:72950-71835;OP422341.1:72963-71848;OP434519.1:72942-71827;OP535324.1:72944-71829;OP535333.1:73053-71938;OP536770.1:72945-71830;OP597769.1:72967-71852;OP604566.1:72946-71831;OP612682.1:72872-71757;OP612684.1:72936-71821;OP612686.1:72936-71821;OP696841.1:70220-69105;OP879722.1:72169-71054;OQ419007.1:72179-71064;OQ419012.1:72146-71031;OQ419031.1:72150-71035;OQ419047.1:72152-71037;OQ451898.1:72031-70916;OQ451900.1:72944-71829;OQ581853.1:72970-71855;OQ581855.1:73051-71936;OQ721951.1:72984-71869;OQ721954.1:72941-71826;OR035652.1:72893-71778;OR035664.1:72893-71778;OR035702.1:72893-71778;OR146272.1:72909-71794;OR146330.1:72954-71839;OR264364.1:72964-71849;OR427332.1:72964-71849;OR459777.1:72948-71833;OR459797.1:72963-71848;OR943698.1:75193-74078;PP098589.2:72945-71830;PP496048.1:72949-71834;PP601206.1:75205-74090;PP776553.1:72870-71755;PP852971.1:72943-71828;PP852983.1:72949-71834;PP853000.1:72910-71795;PP853005.1:72918-71803;PP853007.1:72951-71836;PP853013.1:72924-71809;PP853027.1:72955-71840;PP853045.1:72936-71821;PP853048.1:72955-71840;PP860026.1:72953-71838;PP860027.1:72978-71863;PP860028.1:72918-71803;PP860031.1:72972-71857;PP860033.1:72977-71862;PQ153226.1:72943-71828;PQ178862.2:74056-72941;PQ221506.1:69032-67917;PQ221833.1:75198-74083;PQ340477.1:72926-71811;PQ340478.1:72911-71796;PQ340480.1:72990-71875;PQ340483.1:72974-71859;PQ340485.1:72960-71845;PQ340486.1:72975-71860;PQ340487.1:73000-71885;PQ340489.1:72975-71860;PQ340490.1:72926-71811;PQ340491.1:72981-71866;PQ340493.1:72960-71845;PQ340495.1:72990-71875;PQ340496.1:73023-71908;PQ368489.1:72946-71831;PQ451835.1:73031-71916;PQ451836.1:72963-71848;PQ451838.1:73014-71899;PQ451841.1:72974-71859;PQ451842.1:72926-71811 |
| OG0000104 | AY603973.1:71808-71434;AY741551.1:71812-71438;AY753185.1:72051-71677;DQ011154.1:72269-71895;DQ011155.1:72247-71873;EPI_ISL_13331598:69878-69504;EPI_ISL_13363142:69871-69497;EPI_ISL_13374487:69886-69512;EPI_ISL_13502582:69871-69497;EPI_ISL_13508471:69963-69589;EPI_ISL_13607904:69862-69488;EPI_ISL_13624509:69806-69432;EPI_ISL_13889442:69981-69607;EPI_ISL_13953610:69798-69424;EPI_ISL_14181948:70007-69633;EPI_ISL_14838587:72123-71749;EPI_ISL_15263355:69862-69488;EPI_ISL_15269699:69831-69457;EPI_ISL_15293002:70104-69730;EPI_ISL_15641561:69820-69446;EPI_ISL_15831211:69869-69495;EPI_ISL_15992098:69884-69510;EPI_ISL_16233784:69880-69506;EPI_ISL_16946400:69869-69495;EPI_ISL_17048206:69519-69145;EPI_ISL_17703567:69884-69510;EPI_ISL_17718458:69862-69488;EPI_ISL_18213374:69859-69485;EPI_ISL_18360394:69520-69146;EPI_ISL_18557816:69860-69486;EPI_ISL_18642365:69862-69488;EPI_ISL_18659846:69832-69458;EPI_ISL_18744049:69864-69490;EPI_ISL_19022858:69859-69485;EPI_ISL_19028778:69776-69402;EPI_ISL_19151800:69860-69486;EPI_ISL_19159116:69862-69488;EPI_ISL_19350788:70995-70621;EPI_ISL_19359677:69859-69485;EPI_ISL_19446306:69854-69480;EPI_ISL_19467692:72406-72032;JX878411.1:73114-72740;JX878425.1:72631-72257;JX878426.1:72080-71706;KC257460.1:72045-71671;KJ642612.1:70366-69992;KJ642613.1:71925-71551;KJ642616.1:71502-71128;KJ642618.1:69533-69159;KP849469.1:72439-72065;KP849471.1:72364-71990;LC831698.1:69860-69486;MN648051.1:70082-69708;MN702445.1:65782-65408;MN702446.1:71927-71553;MN702447.1:65295-64921;MN702449.1:65793-65419;MN702452.1:71968-71594;MT903338.1:70025-69651;MT903340.1:69862-69488;MT903341.1:61395-61021;MT903342.1:69877-69503;MT903347.1:71819-71445;ON568298.1:70008-69634;ON622713.1:70094-69720;ON622722.2:69865-69491;ON631963.1:69885-69511;ON649879.1:69695-69321;ON676707.1:69915-69541;ON755039.1:69525-69151;ON808413.1:69865-69491;ON880420.1:70092-69718;ON911481.2:69882-69508;ON918656.1:69897-69523;ON959133.1:69986-69612;ON959136.1:69987-69613;ON983168.1:68561-68187;OP019276.1:69859-69485;OP150924.1:70000-69626;OP160532.1:69825-69451;OP235282.1:69859-69485;OP289782.1:69862-69488;OP331335.1:69879-69505;OP382479.1:69866-69492;OP422341.1:69879-69505;OP434519.1:69858-69484;OP535324.1:69860-69486;OP535333.1:69969-69595;OP536770.1:69861-69487;OP597769.1:69883-69509;OP604566.1:69862-69488;OP612682.1:69788-69414;OP612684.1:69852-69478;OP612686.1:69852-69478;OP696841.1:67136-66762;OP879722.1:69085-68711;OQ419007.1:69095-68721;OQ419012.1:69062-68688;OQ419031.1:69066-68692;OQ419047.1:69068-68694;OQ451898.1:68947-68573;OQ451900.1:69860-69486;OQ581853.1:69886-69512;OQ581855.1:69967-69593;OQ721951.1:69900-69526;OQ721954.1:69857-69483;OR035652.1:69809-69435;OR035664.1:69809-69435;OR035702.1:69809-69435;OR146272.1:69825-69451;OR146330.1:69870-69496;OR264364.1:69880-69506;OR427332.1:69880-69506;OR459777.1:69864-69490;OR459797.1:69879-69505;OR943698.1:72109-71735;PP098589.2:69861-69487;PP496048.1:69865-69491;PP601206.1:72121-71747;PP776553.1:69786-69412;PP852971.1:69859-69485;PP852983.1:69865-69491;PP853000.1:69826-69452;PP853005.1:69834-69460;PP853007.1:69867-69493;PP853013.1:69840-69466;PP853027.1:69871-69497;PP853045.1:69852-69478;PP853048.1:69871-69497;PP860026.1:69869-69495;PP860027.1:69894-69520;PP860028.1:69834-69460;PP860031.1:69888-69514;PP860033.1:69893-69519;PQ153226.1:69859-69485;PQ178862.2:70972-70598;PQ221506.1:65948-65574;PQ221833.1:72114-71740;PQ340477.1:69842-69468;PQ340478.1:69827-69453;PQ340480.1:69906-69532;PQ340483.1:69890-69516;PQ340485.1:69876-69502;PQ340486.1:69891-69517;PQ340487.1:69916-69542;PQ340489.1:69891-69517;PQ340490.1:69842-69468;PQ340491.1:69897-69523;PQ340493.1:69876-69502;PQ340495.1:69906-69532;PQ340496.1:69939-69565;PQ368489.1:69862-69488;PQ451835.1:69947-69573;PQ451836.1:69879-69505;PQ451838.1:69930-69556;PQ451841.1:69890-69516;PQ451842.1:69842-69468 |
| OG0000105 | AY603973.1:70476-68701;AY741551.1:70480-68705;AY753185.1:70719-68944;DQ011154.1:70937-69162;DQ011155.1:70915-69140;EPI_ISL_13331598:68546-66771;EPI_ISL_13363142:68539-66764;EPI_ISL_13374487:68554-66779;EPI_ISL_13502582:68539-66764;EPI_ISL_13508471:68631-66856;EPI_ISL_13607904:68530-66755;EPI_ISL_13624509:68474-66699;EPI_ISL_13889442:68649-66874;EPI_ISL_13953610:68466-66691;EPI_ISL_14181948:68675-66900;EPI_ISL_14838587:70791-69016;EPI_ISL_15263355:68530-66755;EPI_ISL_15269699:68499-66724;EPI_ISL_15293002:68772-66997;EPI_ISL_15641561:68488-66713;EPI_ISL_15831211:68537-66762;EPI_ISL_15992098:68552-66777;EPI_ISL_16233784:68548-66773;EPI_ISL_16946400:68537-66762;EPI_ISL_17048206:68187-66412;EPI_ISL_17703567:68552-66777;EPI_ISL_17718458:68530-66755;EPI_ISL_18213374:68527-66752;EPI_ISL_18360394:68188-66413;EPI_ISL_18557816:68528-66753;EPI_ISL_18642365:68530-66755;EPI_ISL_18659846:68500-66725;EPI_ISL_18744049:68532-66757;EPI_ISL_19022858:68527-66752;EPI_ISL_19028778:68444-66669;EPI_ISL_19151800:68528-66753;EPI_ISL_19159116:68530-66755;EPI_ISL_19350788:69663-67888;EPI_ISL_19359677:68527-66752;EPI_ISL_19446306:68522-66747;EPI_ISL_19467692:71074-69299;JX878411.1:71782-70007;JX878425.1:71299-69524;JX878426.1:70748-68973;KC257460.1:70713-68938;KJ642612.1:69034-67259;KJ642613.1:70593-68818;KJ642616.1:70170-68395;KJ642618.1:68201-66426;KP849469.1:71107-69332;KP849471.1:71032-69257;LC831698.1:68528-66753;MN648051.1:68750-66975;MN702445.1:64450-62675;MN702446.1:70595-68820;MN702447.1:63963-62188;MN702449.1:64461-62686;MN702452.1:70636-68861;MT903338.1:68693-66918;MT903340.1:68530-66755;MT903341.1:60063-58288;MT903342.1:68545-66770;MT903347.1:70487-68712;ON568298.1:68676-66901;ON622713.1:68762-66987;ON622722.2:68533-66758;ON631963.1:68553-66778;ON649879.1:68363-66588;ON676707.1:68583-66808;ON755039.1:68193-66418;ON808413.1:68533-66758;ON880420.1:68760-66985;ON911481.2:68550-66775;ON918656.1:68565-66790;ON959133.1:68654-66879;ON959136.1:68655-66880;ON983168.1:67229-65454;OP019276.1:68527-66752;OP150924.1:68668-66893;OP160532.1:68493-66718;OP235282.1:68527-66752;OP289782.1:68530-66755;OP331335.1:68547-66772;OP382479.1:68534-66759;OP422341.1:68547-66772;OP434519.1:68526-66751;OP535324.1:68528-66753;OP535333.1:68637-66862;OP536770.1:68529-66754;OP597769.1:68551-66776;OP604566.1:68530-66755;OP612682.1:68456-66681;OP612684.1:68520-66745;OP612686.1:68520-66745;OP696841.1:65804-64029;OP879722.1:67753-65978;OQ419007.1:67763-65988;OQ419012.1:67730-65955;OQ419031.1:67734-65959;OQ419047.1:67736-65961;OQ451898.1:67615-65840;OQ451900.1:68528-66753;OQ581853.1:68554-66779;OQ581855.1:68635-66860;OQ721951.1:68568-66793;OQ721954.1:68525-66750;OR035652.1:68477-66702;OR035664.1:68477-66702;OR035702.1:68477-66702;OR146272.1:68493-66718;OR146330.1:68538-66763;OR264364.1:68548-66773;OR427332.1:68548-66773;OR459777.1:68532-66757;OR459797.1:68547-66772;OR943698.1:70777-69002;PP098589.2:68529-66754;PP496048.1:68533-66758;PP601206.1:70789-69014;PP776553.1:68454-66679;PP852971.1:68527-66752;PP852983.1:68533-66758;PP853000.1:68494-66719;PP853005.1:68502-66727;PP853007.1:68535-66760;PP853013.1:68508-66733;PP853027.1:68539-66764;PP853045.1:68520-66745;PP853048.1:68539-66764;PP860026.1:68537-66762;PP860027.1:68562-66787;PP860028.1:68502-66727;PP860031.1:68556-66781;PP860033.1:68561-66786;PQ153226.1:68527-66752;PQ178862.2:69640-67865;PQ221506.1:64616-62841;PQ221833.1:70782-69007;PQ340477.1:68510-66735;PQ340478.1:68495-66720;PQ340480.1:68574-66799;PQ340483.1:68558-66783;PQ340485.1:68544-66769;PQ340486.1:68559-66784;PQ340487.1:68584-66809;PQ340489.1:68559-66784;PQ340490.1:68510-66735;PQ340491.1:68565-66790;PQ340493.1:68544-66769;PQ340495.1:68574-66799;PQ340496.1:68607-66832;PQ368489.1:68530-66755;PQ451835.1:68615-66840;PQ451836.1:68547-66772;PQ451838.1:68598-66823;PQ451841.1:68558-66783;PQ451842.1:68510-66735 |
| OG0000106 | AY603973.1:65397-64249;AY741551.1:65401-64253;AY753185.1:65640-64492;DQ011154.1:65858-64710;DQ011155.1:65836-64688;EPI_ISL_13331598:63467-62319;EPI_ISL_13363142:63460-62312;EPI_ISL_13374487:63475-62327;EPI_ISL_13502582:63460-62312;EPI_ISL_13508471:63552-62404;EPI_ISL_13607904:63451-62303;EPI_ISL_13624509:63395-62247;EPI_ISL_13889442:63570-62422;EPI_ISL_13953610:63387-62239;EPI_ISL_14181948:63596-62448;EPI_ISL_14838587:65712-64564;EPI_ISL_15263355:63451-62303;EPI_ISL_15269699:63420-62272;EPI_ISL_15293002:63693-62545;EPI_ISL_15641561:63409-62261;EPI_ISL_15831211:63458-62310;EPI_ISL_15992098:63473-62325;EPI_ISL_16233784:63469-62321;EPI_ISL_16946400:63458-62310;EPI_ISL_17048206:63108-61960;EPI_ISL_17703567:63473-62325;EPI_ISL_17718458:63451-62303;EPI_ISL_18213374:63448-62300;EPI_ISL_18360394:63109-61961;EPI_ISL_18557816:63449-62301;EPI_ISL_18642365:63451-62303;EPI_ISL_18659846:63421-62273;EPI_ISL_18744049:63453-62305;EPI_ISL_19022858:63448-62300;EPI_ISL_19028778:63365-62217;EPI_ISL_19151800:63449-62301;EPI_ISL_19159116:63451-62303;EPI_ISL_19350788:64584-63436;EPI_ISL_19359677:63448-62300;EPI_ISL_19446306:63443-62295;EPI_ISL_19467692:65995-64847;JX878411.1:66703-65555;JX878425.1:66220-65072;JX878426.1:65669-64521;KC257460.1:65634-64486;KJ642612.1:63955-62807;KJ642613.1:65514-64366;KJ642616.1:65091-63943;KJ642618.1:63122-61974;KP849469.1:66028-64880;KP849471.1:65953-64805;LC831698.1:63449-62301;MN648051.1:63671-62523;MN702445.1:59371-58223;MN702446.1:65516-64368;MN702447.1:58884-57736;MN702449.1:59382-58234;MN702452.1:65557-64409;MT903338.1:63614-62466;MT903340.1:63451-62303;MT903341.1:54984-53836;MT903342.1:63466-62318;MT903347.1:65408-64260;ON568298.1:63597-62449;ON622713.1:63683-62535;ON622722.2:63454-62306;ON631963.1:63474-62326;ON649879.1:63284-62136;ON676707.1:63504-62356;ON755039.1:63114-61966;ON808413.1:63454-62306;ON880420.1:63681-62533;ON911481.2:63471-62323;ON918656.1:63486-62338;ON959133.1:63575-62427;ON959136.1:63576-62428;ON983168.1:62150-61002;OP019276.1:63448-62300;OP150924.1:63589-62441;OP160532.1:63414-62266;OP235282.1:63448-62300;OP289782.1:63451-62303;OP331335.1:63468-62320;OP382479.1:63455-62307;OP422341.1:63468-62320;OP434519.1:63447-62299;OP535324.1:63449-62301;OP535333.1:63558-62410;OP536770.1:63450-62302;OP597769.1:63472-62324;OP604566.1:63451-62303;OP612682.1:63377-62229;OP612684.1:63441-62293;OP612686.1:63441-62293;OP696841.1:60725-59577;OP879722.1:62674-61526;OQ419007.1:62684-61536;OQ419012.1:62651-61503;OQ419031.1:62655-61507;OQ419047.1:62657-61509;OQ451898.1:62536-61388;OQ451900.1:63449-62301;OQ581853.1:63475-62327;OQ581855.1:63556-62408;OQ721951.1:63489-62341;OQ721954.1:63446-62298;OR035652.1:63398-62250;OR035664.1:63398-62250;OR035702.1:63398-62250;OR146272.1:63414-62266;OR146330.1:63459-62311;OR264364.1:63469-62321;OR427332.1:63469-62321;OR459777.1:63453-62305;OR459797.1:63468-62320;OR943698.1:65698-64550;PP098589.2:63450-62302;PP496048.1:63454-62306;PP601206.1:65710-64562;PP776553.1:63375-62227;PP852971.1:63448-62300;PP852983.1:63454-62306;PP853000.1:63415-62267;PP853005.1:63423-62275;PP853007.1:63456-62308;PP853013.1:63429-62281;PP853027.1:63460-62312;PP853045.1:63441-62293;PP853048.1:63460-62312;PP860026.1:63458-62310;PP860027.1:63483-62335;PP860028.1:63423-62275;PP860031.1:63477-62329;PP860033.1:63482-62334;PQ153226.1:63448-62300;PQ178862.2:64561-63413;PQ221506.1:59537-58389;PQ221833.1:65703-64555;PQ340477.1:63431-62283;PQ340478.1:63416-62268;PQ340480.1:63495-62347;PQ340483.1:63479-62331;PQ340485.1:63465-62317;PQ340486.1:63480-62332;PQ340487.1:63505-62357;PQ340489.1:63480-62332;PQ340490.1:63431-62283;PQ340491.1:63486-62338;PQ340493.1:63465-62317;PQ340495.1:63495-62347;PQ340496.1:63528-62380;PQ368489.1:63451-62303;PQ451835.1:63536-62388;PQ451836.1:63468-62320;PQ451838.1:63519-62371;PQ451841.1:63479-62331;PQ451842.1:63431-62283 |
| OG0000107 | AY603973.1:64230-63991;AY741551.1:64234-63995;AY753185.1:64473-64234;DQ011154.1:64691-64452;DQ011155.1:64669-64430;EPI_ISL_13331598:62300-62061;EPI_ISL_13363142:62293-62054;EPI_ISL_13374487:62308-62069;EPI_ISL_13502582:62293-62054;EPI_ISL_13508471:62385-62146;EPI_ISL_13607904:62284-62045;EPI_ISL_13624509:62228-61989;EPI_ISL_13889442:62403-62164;EPI_ISL_13953610:62220-61981;EPI_ISL_14181948:62429-62190;EPI_ISL_14838587:64545-64306;EPI_ISL_15263355:62284-62045;EPI_ISL_15269699:62253-62014;EPI_ISL_15293002:62526-62287;EPI_ISL_15641561:62242-62003;EPI_ISL_15831211:62291-62052;EPI_ISL_15992098:62306-62067;EPI_ISL_16233784:62302-62063;EPI_ISL_16946400:62291-62052;EPI_ISL_17048206:61941-61702;EPI_ISL_17703567:62306-62067;EPI_ISL_17718458:62284-62045;EPI_ISL_18213374:62281-62042;EPI_ISL_18360394:61942-61703;EPI_ISL_18557816:62282-62043;EPI_ISL_18642365:62284-62045;EPI_ISL_18659846:62254-62015;EPI_ISL_18744049:62286-62047;EPI_ISL_19022858:62281-62042;EPI_ISL_19028778:62198-61959;EPI_ISL_19151800:62282-62043;EPI_ISL_19159116:62284-62045;EPI_ISL_19350788:63417-63178;EPI_ISL_19359677:62281-62042;EPI_ISL_19446306:62276-62037;EPI_ISL_19467692:64828-64589;JX878411.1:65536-65297;JX878425.1:65053-64814;JX878426.1:64502-64263;KC257460.1:64467-64228;KJ642612.1:62788-62549;KJ642613.1:64347-64108;KJ642616.1:63924-63685;KJ642618.1:61955-61716;KP849469.1:64861-64622;KP849471.1:64786-64547;LC831698.1:62282-62043;MN648051.1:62504-62265;MN702445.1:58204-57965;MN702446.1:64349-64110;MN702447.1:57717-57478;MN702449.1:58215-57976;MN702452.1:64390-64151;MT903338.1:62447-62208;MT903340.1:62284-62045;MT903341.1:53817-53578;MT903342.1:62299-62060;MT903347.1:64241-64002;ON568298.1:62430-62191;ON622713.1:62516-62277;ON622722.2:62287-62048;ON631963.1:62307-62068;ON649879.1:62117-61878;ON676707.1:62337-62098;ON755039.1:61947-61708;ON808413.1:62287-62048;ON880420.1:62514-62275;ON911481.2:62304-62065;ON918656.1:62319-62080;ON959133.1:62408-62169;ON959136.1:62409-62170;ON983168.1:60983-60744;OP019276.1:62281-62042;OP150924.1:62422-62183;OP160532.1:62247-62008;OP235282.1:62281-62042;OP289782.1:62284-62045;OP331335.1:62301-62062;OP382479.1:62288-62049;OP422341.1:62301-62062;OP434519.1:62280-62041;OP535324.1:62282-62043;OP535333.1:62391-62152;OP536770.1:62283-62044;OP597769.1:62305-62066;OP604566.1:62284-62045;OP612682.1:62210-61971;OP612684.1:62274-62035;OP612686.1:62274-62035;OP696841.1:59558-59319;OP879722.1:61507-61268;OQ419007.1:61517-61278;OQ419012.1:61484-61245;OQ419031.1:61488-61249;OQ419047.1:61490-61251;OQ451898.1:61369-61130;OQ451900.1:62282-62043;OQ581853.1:62308-62069;OQ581855.1:62389-62150;OQ721951.1:62322-62083;OQ721954.1:62279-62040;OR035652.1:62231-61992;OR035664.1:62231-61992;OR035702.1:62231-61992;OR146272.1:62247-62008;OR146330.1:62292-62053;OR264364.1:62302-62063;OR427332.1:62302-62063;OR459777.1:62286-62047;OR459797.1:62301-62062;OR943698.1:64531-64292;PP098589.2:62283-62044;PP496048.1:62287-62048;PP601206.1:64543-64304;PP776553.1:62208-61969;PP852971.1:62281-62042;PP852983.1:62287-62048;PP853000.1:62248-62009;PP853005.1:62256-62017;PP853007.1:62289-62050;PP853013.1:62262-62023;PP853027.1:62293-62054;PP853045.1:62274-62035;PP853048.1:62293-62054;PP860026.1:62291-62052;PP860027.1:62316-62077;PP860028.1:62256-62017;PP860031.1:62310-62071;PP860033.1:62315-62076;PQ153226.1:62281-62042;PQ178862.2:63394-63155;PQ221506.1:58370-58131;PQ221833.1:64536-64297;PQ340477.1:62264-62025;PQ340478.1:62249-62010;PQ340480.1:62328-62089;PQ340483.1:62312-62073;PQ340485.1:62298-62059;PQ340486.1:62313-62074;PQ340487.1:62338-62099;PQ340489.1:62313-62074;PQ340490.1:62264-62025;PQ340491.1:62319-62080;PQ340493.1:62298-62059;PQ340495.1:62328-62089;PQ340496.1:62361-62122;PQ368489.1:62284-62045;PQ451835.1:62369-62130;PQ451836.1:62301-62062;PQ451838.1:62352-62113;PQ451841.1:62312-62073;PQ451842.1:62264-62025 |
| OG0000108 | AY603973.1:59442-59116;AY741551.1:59446-59120;AY753185.1:59685-59359;DQ011154.1:59903-59577;DQ011155.1:59881-59555;EPI_ISL_13331598:57513-57187;EPI_ISL_13363142:57506-57180;EPI_ISL_13374487:57521-57195;EPI_ISL_13502582:57506-57180;EPI_ISL_13508471:57598-57272;EPI_ISL_13607904:57497-57171;EPI_ISL_13624509:57441-57115;EPI_ISL_13889442:57616-57290;EPI_ISL_13953610:57433-57107;EPI_ISL_14181948:57642-57316;EPI_ISL_14838587:59757-59431;EPI_ISL_15263355:57497-57171;EPI_ISL_15269699:57466-57140;EPI_ISL_15293002:57739-57413;EPI_ISL_15641561:57455-57129;EPI_ISL_15831211:57504-57178;EPI_ISL_15992098:57519-57193;EPI_ISL_16233784:57515-57189;EPI_ISL_16946400:57504-57178;EPI_ISL_17048206:57154-56828;EPI_ISL_17703567:57519-57193;EPI_ISL_17718458:57497-57171;EPI_ISL_18213374:57494-57168;EPI_ISL_18360394:57155-56829;EPI_ISL_18557816:57495-57169;EPI_ISL_18642365:57497-57171;EPI_ISL_18659846:57467-57141;EPI_ISL_18744049:57499-57173;EPI_ISL_19022858:57494-57168;EPI_ISL_19028778:57411-57085;EPI_ISL_19151800:57495-57169;EPI_ISL_19159116:57497-57171;EPI_ISL_19350788:58629-58303;EPI_ISL_19359677:57494-57168;EPI_ISL_19446306:57489-57163;EPI_ISL_19467692:60040-59714;JX878411.1:60748-60422;JX878425.1:60265-59939;JX878426.1:59714-59388;KC257460.1:59679-59353;KJ642612.1:58000-57674;KJ642613.1:59559-59233;KJ642616.1:59136-58810;KJ642618.1:57167-56841;KP849469.1:60073-59747;KP849471.1:59998-59672;LC831698.1:57495-57169;MN648051.1:57717-57391;MN702445.1:53416-53090;MN702446.1:59561-59235;MN702447.1:52929-52603;MN702449.1:53427-53101;MN702452.1:59602-59276;MT903338.1:57660-57334;MT903340.1:57497-57171;MT903341.1:49030-48704;MT903342.1:57512-57186;MT903347.1:59453-59127;ON568298.1:57643-57317;ON622713.1:57729-57403;ON622722.2:57500-57174;ON631963.1:57520-57194;ON649879.1:57330-57004;ON676707.1:57550-57224;ON755039.1:57160-56834;ON808413.1:57500-57174;ON880420.1:57727-57401;ON911481.2:57517-57191;ON918656.1:57532-57206;ON959133.1:57621-57295;ON959136.1:57622-57296;ON983168.1:56196-55870;OP019276.1:57494-57168;OP150924.1:57635-57309;OP160532.1:57460-57134;OP235282.1:57494-57168;OP289782.1:57497-57171;OP331335.1:57514-57188;OP382479.1:57501-57175;OP422341.1:57514-57188;OP434519.1:57493-57167;OP535324.1:57495-57169;OP535333.1:57604-57278;OP536770.1:57496-57170;OP597769.1:57518-57192;OP604566.1:57497-57171;OP612682.1:57423-57097;OP612684.1:57487-57161;OP612686.1:57487-57161;OP696841.1:54771-54445;OP879722.1:56720-56394;OQ419007.1:56730-56404;OQ419012.1:56697-56371;OQ419031.1:56701-56375;OQ419047.1:56703-56404;OQ451898.1:56582-56256;OQ451900.1:57495-57169;OQ581853.1:57521-57195;OQ581855.1:57602-57276;OQ721951.1:57535-57209;OQ721954.1:57492-57166;OR035652.1:57444-57118;OR035664.1:57444-57118;OR035702.1:57444-57118;OR146272.1:57460-57134;OR146330.1:57505-57179;OR264364.1:57515-57189;OR427332.1:57515-57189;OR459777.1:57499-57173;OR459797.1:57514-57188;OR943698.1:59743-59417;PP098589.2:57496-57170;PP496048.1:57500-57174;PP601206.1:59755-59429;PP776553.1:57421-57095;PP852971.1:57494-57168;PP852983.1:57500-57174;PP853000.1:57461-57135;PP853005.1:57469-57143;PP853007.1:57502-57176;PP853013.1:57475-57149;PP853027.1:57506-57180;PP853045.1:57487-57161;PP853048.1:57506-57180;PP860026.1:57504-57178;PP860027.1:57529-57203;PP860028.1:57470-57144;PP860031.1:57523-57197;PP860033.1:57528-57202;PQ153226.1:57494-57168;PQ178862.2:58606-58280;PQ221506.1:53582-53256;PQ221833.1:59748-59422;PQ340477.1:57477-57151;PQ340478.1:57462-57136;PQ340480.1:57541-57215;PQ340483.1:57525-57199;PQ340485.1:57511-57185;PQ340486.1:57526-57200;PQ340487.1:57551-57225;PQ340489.1:57526-57200;PQ340490.1:57477-57151;PQ340491.1:57532-57206;PQ340493.1:57511-57185;PQ340495.1:57541-57215;PQ340496.1:57574-57248;PQ368489.1:57497-57171;PQ451835.1:57582-57256;PQ451836.1:57514-57188;PQ451838.1:57565-57239;PQ451841.1:57525-57199;PQ451842.1:57477-57151 |
| OG0000109 | AY603973.1:56592-56410;AY741551.1:56596-56414;AY753185.1:56835-56653;DQ011154.1:57053-56871;DQ011155.1:57031-56849;EPI_ISL_13331598:54663-54481;EPI_ISL_13363142:54656-54474;EPI_ISL_13374487:54671-54489;EPI_ISL_13502582:54656-54474;EPI_ISL_13508471:54748-54566;EPI_ISL_13607904:54647-54465;EPI_ISL_13624509:54591-54409;EPI_ISL_13889442:54766-54584;EPI_ISL_13953610:54583-54401;EPI_ISL_14181948:54792-54610;EPI_ISL_14838587:56907-56725;EPI_ISL_15263355:54647-54465;EPI_ISL_15269699:54616-54434;EPI_ISL_15293002:54889-54707;EPI_ISL_15641561:54605-54423;EPI_ISL_15831211:54654-54472;EPI_ISL_15992098:54669-54487;EPI_ISL_16233784:54665-54483;EPI_ISL_16946400:54654-54472;EPI_ISL_17048206:54304-54122;EPI_ISL_17703567:54669-54487;EPI_ISL_17718458:54647-54465;EPI_ISL_18213374:54644-54462;EPI_ISL_18360394:54305-54123;EPI_ISL_18557816:54645-54463;EPI_ISL_18642365:54647-54465;EPI_ISL_18659846:54617-54435;EPI_ISL_18744049:54649-54467;EPI_ISL_19022858:54644-54462;EPI_ISL_19028778:54561-54379;EPI_ISL_19151800:54645-54463;EPI_ISL_19159116:54647-54465;EPI_ISL_19350788:55779-55597;EPI_ISL_19359677:54644-54462;EPI_ISL_19446306:54639-54457;EPI_ISL_19467692:57190-57008;JX878411.1:57898-57716;JX878425.1:57415-57233;JX878426.1:56864-56682;KC257460.1:56829-56647;KJ642612.1:55150-54968;KJ642613.1:56709-56527;KJ642616.1:56286-56104;KJ642618.1:54317-54135;KP849469.1:57223-57041;KP849471.1:57148-56966;LC831698.1:54645-54463;MN648051.1:54867-54685;MN702445.1:50566-50384;MN702446.1:56711-56529;MN702447.1:50079-49897;MN702449.1:50577-50395;MN702452.1:56752-56570;MT903338.1:54810-54628;MT903340.1:54647-54465;MT903341.1:46180-45998;MT903342.1:54662-54480;MT903347.1:56603-56421;ON568298.1:54793-54611;ON622713.1:54879-54697;ON622722.2:54650-54468;ON631963.1:54670-54488;ON649879.1:54480-54298;ON676707.1:54700-54518;ON755039.1:54310-54128;ON808413.1:54650-54468;ON880420.1:54877-54695;ON911481.2:54667-54485;ON918656.1:54682-54500;ON959133.1:54771-54589;ON959136.1:54772-54590;ON983168.1:53346-53164;OP019276.1:54644-54462;OP150924.1:54785-54603;OP160532.1:54610-54428;OP235282.1:54644-54462;OP289782.1:54647-54465;OP331335.1:54664-54482;OP382479.1:54651-54469;OP422341.1:54664-54482;OP434519.1:54643-54461;OP535324.1:54645-54463;OP535333.1:54754-54572;OP536770.1:54646-54464;OP597769.1:54668-54486;OP604566.1:54647-54465;OP612682.1:54573-54391;OP612684.1:54637-54455;OP612686.1:54637-54455;OP696841.1:51921-51739;OP879722.1:53870-53688;OQ419007.1:53880-53698;OQ419012.1:53847-53665;OQ419031.1:53851-53669;OQ419047.1:53880-53698;OQ451898.1:53732-53550;OQ451900.1:54645-54463;OQ581853.1:54671-54489;OQ581855.1:54752-54570;OQ721951.1:54685-54503;OQ721954.1:54642-54460;OR035652.1:54594-54412;OR035664.1:54594-54412;OR035702.1:54594-54412;OR146272.1:54610-54428;OR146330.1:54655-54473;OR264364.1:54665-54483;OR427332.1:54665-54483;OR459777.1:54649-54467;OR459797.1:54664-54482;OR943698.1:56893-56711;PP098589.2:54646-54464;PP496048.1:54650-54468;PP601206.1:56905-56723;PP776553.1:54571-54389;PP852971.1:54644-54462;PP852983.1:54650-54468;PP853000.1:54611-54429;PP853005.1:54619-54437;PP853007.1:54652-54470;PP853013.1:54625-54443;PP853027.1:54656-54474;PP853045.1:54637-54455;PP853048.1:54656-54474;PP860026.1:54654-54472;PP860027.1:54679-54497;PP860028.1:54620-54438;PP860031.1:54673-54491;PP860033.1:54678-54496;PQ153226.1:54644-54462;PQ178862.2:55756-55574;PQ221506.1:50732-50550;PQ221833.1:56898-56716;PQ340477.1:54627-54445;PQ340478.1:54612-54430;PQ340480.1:54691-54509;PQ340483.1:54675-54493;PQ340485.1:54661-54479;PQ340486.1:54676-54494;PQ340487.1:54701-54519;PQ340489.1:54676-54494;PQ340490.1:54627-54445;PQ340491.1:54682-54500;PQ340493.1:54661-54479;PQ340495.1:54691-54509;PQ340496.1:54724-54542;PQ368489.1:54647-54465;PQ451835.1:54732-54550;PQ451836.1:54664-54482;PQ451838.1:54715-54533;PQ451841.1:54675-54493;PQ451842.1:54627-54445 |
| OG0000110 | AY603973.1:56382-53362;AY741551.1:56386-53366;AY753185.1:56625-53605;DQ011154.1:56843-53823;DQ011155.1:56821-53801;EPI_ISL_13331598:54453-51433;EPI_ISL_13363142:54446-51426;EPI_ISL_13374487:54461-51441;EPI_ISL_13502582:54446-51426;EPI_ISL_13508471:54538-51518;EPI_ISL_13607904:54437-51417;EPI_ISL_13624509:54381-51361;EPI_ISL_13889442:54556-51536;EPI_ISL_13953610:54373-51377;EPI_ISL_14181948:54582-51562;EPI_ISL_14838587:56697-53677;EPI_ISL_15263355:54437-51417;EPI_ISL_15269699:54406-51386;EPI_ISL_15293002:54679-51659;EPI_ISL_15641561:54395-51375;EPI_ISL_15831211:54444-51424;EPI_ISL_15992098:54459-51439;EPI_ISL_16233784:54455-51435;EPI_ISL_16946400:54444-51424;EPI_ISL_17048206:54094-51074;EPI_ISL_17703567:54459-51439;EPI_ISL_17718458:54437-51417;EPI_ISL_18213374:54434-51414;EPI_ISL_18360394:54095-51075;EPI_ISL_18557816:54435-51415;EPI_ISL_18642365:54437-51417;EPI_ISL_18659846:54407-51387;EPI_ISL_18744049:54439-51419;EPI_ISL_19022858:54434-51414;EPI_ISL_19028778:54351-51331;EPI_ISL_19151800:54435-51415;EPI_ISL_19159116:54437-51417;EPI_ISL_19350788:55569-52549;EPI_ISL_19359677:54434-51414;EPI_ISL_19446306:54429-51409;EPI_ISL_19467692:56980-53960;JX878411.1:57688-54668;JX878425.1:57205-54185;JX878426.1:56654-53634;KC257460.1:56619-53599;KJ642612.1:54940-51920;KJ642613.1:56499-53479;KJ642616.1:56076-53056;KJ642618.1:54107-51087;KP849469.1:57013-53993;KP849471.1:56938-53918;LC831698.1:54435-51415;MN648051.1:54657-51637;MN702445.1:50356-47336;MN702446.1:56501-53481;MN702447.1:49869-46849;MN702449.1:50367-47347;MN702452.1:56542-53522;MT903338.1:54600-51580;MT903340.1:54437-51417;MT903341.1:45970-42950;MT903342.1:54452-51432;MT903347.1:56393-53373;ON568298.1:54583-51563;ON622713.1:54669-51649;ON622722.2:54440-51420;ON631963.1:54460-51440;ON649879.1:54270-51250;ON676707.1:54490-51470;ON755039.1:54100-51080;ON808413.1:54440-51420;ON880420.1:54667-51647;ON911481.2:54457-51437;ON918656.1:54472-51452;ON959133.1:54561-51541;ON959136.1:54562-51542;ON983168.1:53136-50116;OP019276.1:54434-51414;OP150924.1:54575-51555;OP160532.1:54400-51380;OP235282.1:54434-51414;OP289782.1:54437-51417;OP331335.1:54454-51434;OP382479.1:54441-51421;OP422341.1:54454-51434;OP434519.1:54433-51413;OP535324.1:54435-51415;OP535333.1:54544-51524;OP536770.1:54436-51416;OP597769.1:54458-51438;OP604566.1:54437-51417;OP612682.1:54363-51343;OP612684.1:54427-51407;OP612686.1:54427-51407;OP696841.1:51711-48691;OP879722.1:53660-50640;OQ419007.1:53670-50650;OQ419012.1:53637-50617;OQ419031.1:53641-50621;OQ419047.1:53670-50650;OQ451898.1:53522-50502;OQ451900.1:54435-51415;OQ581853.1:54461-51441;OQ581855.1:54542-51522;OQ721951.1:54475-51455;OQ721954.1:54432-51412;OR035652.1:54384-51364;OR035664.1:54384-51364;OR035702.1:54384-51364;OR146272.1:54400-51380;OR146330.1:54445-51425;OR264364.1:54455-51435;OR427332.1:54455-51435;OR459777.1:54439-51419;OR459797.1:54454-51434;OR943698.1:56683-53663;PP098589.2:54436-51416;PP496048.1:54440-51420;PP601206.1:56695-53675;PP776553.1:54361-51341;PP852971.1:54434-51414;PP852983.1:54440-51420;PP853000.1:54401-51381;PP853005.1:54409-51389;PP853007.1:54442-51422;PP853013.1:54415-51395;PP853027.1:54446-51426;PP853045.1:54427-51407;PP853048.1:54446-51426;PP860026.1:54444-51424;PP860027.1:54469-51449;PP860028.1:54410-51390;PP860031.1:54463-51443;PP860033.1:54468-51448;PQ153226.1:54434-51414;PQ178862.2:55546-52526;PQ221506.1:50522-47502;PQ221833.1:56688-53668;PQ340477.1:54417-51397;PQ340478.1:54402-51382;PQ340480.1:54481-51461;PQ340483.1:54465-51445;PQ340485.1:54451-51431;PQ340486.1:54466-51446;PQ340487.1:54491-51471;PQ340489.1:54466-51446;PQ340490.1:54417-51397;PQ340491.1:54472-51452;PQ340493.1:54451-51431;PQ340495.1:54481-51461;PQ340496.1:54514-51494;PQ368489.1:54437-51417;PQ451835.1:54522-51502;PQ451836.1:54454-51434;PQ451838.1:54505-51485;PQ451841.1:54465-51445;PQ451842.1:54417-51397 |
| OG0000111 | AY603973.1:42378-42157;AY741551.1:42382-42161;AY753185.1:42621-42400;DQ011154.1:42597-42376;DQ011155.1:42578-42357;EPI_ISL_13331598:40449-40228;EPI_ISL_13363142:40442-40221;EPI_ISL_13374487:40457-40236;EPI_ISL_13502582:40442-40221;EPI_ISL_13508471:40534-40313;EPI_ISL_13607904:40433-40212;EPI_ISL_13624509:40377-40156;EPI_ISL_13889442:40552-40331;EPI_ISL_13953610:40400-40179;EPI_ISL_14181948:40578-40357;EPI_ISL_14838587:42451-42230;EPI_ISL_15263355:40433-40212;EPI_ISL_15269699:40402-40181;EPI_ISL_15293002:40675-40454;EPI_ISL_15641561:40391-40170;EPI_ISL_15831211:40440-40219;EPI_ISL_15992098:40455-40234;EPI_ISL_16233784:40451-40230;EPI_ISL_16946400:40440-40219;EPI_ISL_17048206:40090-39869;EPI_ISL_17703567:40455-40234;EPI_ISL_17718458:40433-40212;EPI_ISL_18213374:40430-40209;EPI_ISL_18360394:40091-39870;EPI_ISL_18557816:40431-40210;EPI_ISL_18642365:40433-40212;EPI_ISL_18659846:40403-40182;EPI_ISL_18744049:40435-40214;EPI_ISL_19022858:40430-40209;EPI_ISL_19028778:40347-40126;EPI_ISL_19151800:40431-40210;EPI_ISL_19159116:40433-40212;EPI_ISL_19350788:41325-41104;EPI_ISL_19359677:40430-40209;EPI_ISL_19446306:40425-40204;EPI_ISL_19467692:42730-42509;JX878411.1:43445-43224;JX878425.1:42959-42738;JX878426.1:42404-42183;KC257460.1:42375-42154;KJ642612.1:40690-40469;KJ642613.1:42266-42045;KJ642616.1:42072-41851;KJ642618.1:39858-39637;KP849469.1:42767-42546;KP849471.1:42693-42472;LC831698.1:40431-40210;MN648051.1:40653-40432;MN702445.1:36112-35891;MN702446.1:42257-42036;MN702447.1:35623-35402;MN702449.1:36123-35902;MN702452.1:42296-42075;MT903338.1:40596-40375;MT903340.1:40433-40212;MT903341.1:31966-31745;MT903342.1:40448-40227;MT903347.1:42391-42170;ON568298.1:40579-40358;ON622713.1:40665-40444;ON622722.2:40436-40215;ON631963.1:40456-40235;ON649879.1:40266-40045;ON676707.1:40486-40265;ON755039.1:40096-39875;ON808413.1:40436-40215;ON880420.1:40663-40442;ON911481.2:40453-40232;ON918656.1:40468-40247;ON959133.1:40557-40336;ON959136.1:40558-40337;ON983168.1:39132-38911;OP019276.1:40430-40209;OP150924.1:40571-40350;OP160532.1:40396-40175;OP235282.1:40430-40209;OP289782.1:40433-40212;OP331335.1:40450-40229;OP382479.1:40437-40216;OP422341.1:40450-40229;OP434519.1:40429-40208;OP535324.1:40431-40210;OP535333.1:40540-40319;OP536770.1:40432-40211;OP597769.1:40454-40233;OP604566.1:40433-40212;OP612682.1:40359-40138;OP612684.1:40423-40202;OP612686.1:40423-40202;OP696841.1:37707-37486;OP879722.1:39656-39435;OQ419007.1:39666-39445;OQ419012.1:39655-39434;OQ419031.1:39637-39416;OQ419047.1:39666-39445;OQ451898.1:39518-39297;OQ451900.1:40431-40210;OQ581853.1:40457-40236;OQ581855.1:40538-40317;OQ721951.1:40471-40250;OQ721954.1:40428-40207;OR035652.1:40380-40159;OR035664.1:40380-40159;OR035702.1:40380-40159;OR146272.1:40396-40175;OR146330.1:40441-40220;OR264364.1:40451-40230;OR427332.1:40451-40230;OR459777.1:40435-40214;OR459797.1:40450-40229;OR943698.1:42432-42211;PP098589.2:40432-40211;PP496048.1:40436-40215;PP601206.1:42449-42228;PP776553.1:40357-40136;PP852971.1:40430-40209;PP852983.1:40436-40215;PP853000.1:40397-40176;PP853005.1:40405-40184;PP853007.1:40438-40217;PP853013.1:40411-40190;PP853027.1:40442-40221;PP853045.1:40423-40202;PP853048.1:40442-40221;PP860026.1:40440-40219;PP860027.1:40465-40244;PP860028.1:40406-40185;PP860031.1:40459-40238;PP860033.1:40464-40243;PQ153226.1:40430-40209;PQ178862.2:41302-41081;PQ221506.1:36272-36051;PQ221833.1:42441-42220;PQ340477.1:40413-40192;PQ340478.1:40398-40177;PQ340480.1:40477-40256;PQ340483.1:40461-40240;PQ340485.1:40447-40226;PQ340486.1:40462-40241;PQ340487.1:40487-40266;PQ340489.1:40462-40241;PQ340490.1:40413-40192;PQ340491.1:40468-40247;PQ340493.1:40447-40226;PQ340495.1:40477-40256;PQ340496.1:40510-40289;PQ368489.1:40433-40212;PQ451835.1:40518-40297;PQ451836.1:40450-40229;PQ451838.1:40501-40280;PQ451841.1:40461-40240;PQ451842.1:40413-40192 |
| OG0000112 | AY603973.1:39027-37963;AY741551.1:39031-37967;AY753185.1:39270-38206;DQ011154.1:39247-38183;DQ011155.1:39228-38164;EPI_ISL_13331598:37098-36034;EPI_ISL_13363142:37091-36027;EPI_ISL_13374487:37106-36042;EPI_ISL_13502582:37091-36027;EPI_ISL_13508471:37183-36119;EPI_ISL_13607904:37082-36018;EPI_ISL_13624509:37026-35962;EPI_ISL_13889442:37201-36137;EPI_ISL_13953610:37049-35985;EPI_ISL_14181948:37227-36163;EPI_ISL_14838587:39101-38037;EPI_ISL_15263355:37082-36018;EPI_ISL_15269699:37051-35987;EPI_ISL_15293002:37324-36260;EPI_ISL_15641561:37040-35976;EPI_ISL_15831211:37089-36025;EPI_ISL_15992098:37104-36040;EPI_ISL_16233784:37100-36036;EPI_ISL_16946400:37089-36025;EPI_ISL_17048206:36739-35675;EPI_ISL_17703567:37104-36040;EPI_ISL_17718458:37082-36018;EPI_ISL_18213374:37079-36015;EPI_ISL_18360394:36740-35676;EPI_ISL_18557816:37080-36016;EPI_ISL_18642365:37082-36018;EPI_ISL_18659846:37052-35988;EPI_ISL_18744049:37084-36020;EPI_ISL_19022858:37079-36015;EPI_ISL_19028778:36996-35932;EPI_ISL_19151800:37080-36016;EPI_ISL_19159116:37082-36018;EPI_ISL_19350788:37975-36911;EPI_ISL_19359677:37079-36015;EPI_ISL_19446306:37074-36010;EPI_ISL_19467692:39380-38316;JX878411.1:40095-39031;JX878425.1:39609-38545;JX878426.1:39054-37990;KC257460.1:39025-37961;KJ642612.1:37340-36276;KJ642613.1:38916-37852;KJ642616.1:38721-37657;KJ642618.1:36508-35444;KP849469.1:39417-38353;KP849471.1:39343-38279;LC831698.1:37080-36016;MN648051.1:37302-36238;MN702445.1:32762-31698;MN702446.1:38908-37844;MN702447.1:32273-31209;MN702449.1:32773-31709;MN702452.1:38946-37882;MT903338.1:37245-36181;MT903340.1:37082-36018;MT903341.1:28615-27551;MT903342.1:37097-36033;MT903347.1:39040-37976;ON568298.1:37228-36164;ON622713.1:37314-36250;ON622722.2:37085-36021;ON631963.1:37105-36041;ON649879.1:36915-35851;ON676707.1:37135-36071;ON755039.1:36745-35681;ON808413.1:37085-36021;ON880420.1:37312-36248;ON911481.2:37102-36038;ON918656.1:37117-36053;ON959133.1:37206-36142;ON959136.1:37207-36143;ON983168.1:35781-34717;OP019276.1:37079-36015;OP150924.1:37220-36156;OP160532.1:37045-35981;OP235282.1:37079-36015;OP289782.1:37082-36018;OP331335.1:37099-36035;OP382479.1:37086-36022;OP422341.1:37099-36035;OP434519.1:37078-36014;OP535324.1:37080-36016;OP535333.1:37189-36125;OP536770.1:37081-36017;OP597769.1:37103-36039;OP604566.1:37082-36018;OP612682.1:37008-35944;OP612684.1:37072-36008;OP612686.1:37072-36008;OP696841.1:34356-33292;OP879722.1:36305-35241;OQ419007.1:36315-35251;OQ419012.1:36307-35243;OQ419031.1:36286-35222;OQ419047.1:36315-35251;OQ451898.1:36167-35103;OQ451900.1:37080-36016;OQ581853.1:37106-36042;OQ581855.1:37187-36123;OQ721951.1:37120-36056;OQ721954.1:37077-36013;OR035652.1:37029-35965;OR035664.1:37029-35965;OR035702.1:37029-35965;OR146272.1:37045-35981;OR146330.1:37090-36026;OR264364.1:37100-36036;OR427332.1:37100-36036;OR459777.1:37084-36020;OR459797.1:37099-36035;OR943698.1:39082-38018;PP098589.2:37081-36017;PP496048.1:37085-36021;PP601206.1:39099-38035;PP776553.1:37006-35942;PP852971.1:37079-36015;PP852983.1:37085-36021;PP853000.1:37046-35982;PP853005.1:37054-35990;PP853007.1:37087-36023;PP853013.1:37060-35996;PP853027.1:37091-36027;PP853045.1:37072-36008;PP853048.1:37091-36027;PP860026.1:37089-36025;PP860027.1:37114-36050;PP860028.1:37055-35991;PP860031.1:37108-36044;PP860033.1:37113-36049;PQ153226.1:37079-36015;PQ178862.2:37952-36888;PQ221506.1:32922-31858;PQ221833.1:39091-38027;PQ340477.1:37062-35998;PQ340478.1:37047-35983;PQ340480.1:37126-36062;PQ340483.1:37110-36046;PQ340485.1:37096-36032;PQ340486.1:37111-36047;PQ340487.1:37136-36072;PQ340489.1:37111-36047;PQ340490.1:37062-35998;PQ340491.1:37120-36056;PQ340493.1:37096-36032;PQ340495.1:37126-36062;PQ340496.1:37159-36095;PQ368489.1:37082-36018;PQ451835.1:37170-36106;PQ451836.1:37102-36038;PQ451838.1:37153-36089;PQ451841.1:37113-36049;PQ451842.1:37065-36001 |
| OG0000113 | AY603973.1:35175-34144;AY741551.1:35179-34148;AY753185.1:35418-34387;DQ011154.1:35395-34364;DQ011155.1:35376-34345;EPI_ISL_13331598:33166-32210;EPI_ISL_13363142:33159-32203;EPI_ISL_13374487:33174-32218;EPI_ISL_13502582:33159-32203;EPI_ISL_13508471:33251-32295;EPI_ISL_13607904:33150-32194;EPI_ISL_13624509:33094-32138;EPI_ISL_13889442:33269-32313;EPI_ISL_13953610:33117-32161;EPI_ISL_14181948:33295-32339;EPI_ISL_14838587:35249-34218;EPI_ISL_15263355:33150-32194;EPI_ISL_15269699:33119-32163;EPI_ISL_15293002:33392-32436;EPI_ISL_15641561:33108-32152;EPI_ISL_15831211:33157-32201;EPI_ISL_15992098:33172-32216;EPI_ISL_16233784:33168-32212;EPI_ISL_16946400:33157-32201;EPI_ISL_17048206:32807-31851;EPI_ISL_17703567:33172-32216;EPI_ISL_17718458:33150-32194;EPI_ISL_18213374:33147-32191;EPI_ISL_18360394:32808-31852;EPI_ISL_18557816:33148-32192;EPI_ISL_18642365:33150-32194;EPI_ISL_18659846:33120-32164;EPI_ISL_18744049:33152-32196;EPI_ISL_19022858:33147-32191;EPI_ISL_19028778:33064-32108;EPI_ISL_19151800:33148-32192;EPI_ISL_19159116:33150-32194;EPI_ISL_19350788:34123-33092;EPI_ISL_19359677:33147-32191;EPI_ISL_19446306:33142-32186;EPI_ISL_19467692:35528-34497;JX878411.1:36243-35212;JX878425.1:35757-34726;JX878426.1:35202-34171;KC257460.1:35173-34142;KJ642612.1:33488-32457;KJ642613.1:35064-34033;KJ642616.1:34869-33838;KJ642618.1:32656-31625;KP849469.1:35565-34534;KP849471.1:35491-34460;LC831698.1:33148-32192;MN648051.1:33370-32414;MN702445.1:28910-27879;MN702446.1:35056-34025;MN702447.1:28421-27390;MN702449.1:28921-27890;MN702452.1:35094-34063;MT903338.1:33313-32357;MT903340.1:33150-32194;MT903341.1:24683-23727;MT903342.1:33165-32209;MT903347.1:35188-34157;ON568298.1:33296-32340;ON622713.1:33382-32426;ON622722.2:33153-32197;ON631963.1:33173-32217;ON649879.1:32983-32027;ON676707.1:33203-32247;ON755039.1:32813-31857;ON808413.1:33153-32197;ON880420.1:33380-32424;ON911481.2:33170-32214;ON918656.1:33185-32229;ON959133.1:33274-32318;ON959136.1:33275-32319;ON983168.1:31849-30893;OP019276.1:33147-32191;OP150924.1:33288-32332;OP160532.1:33113-32157;OP235282.1:33147-32191;OP289782.1:33150-32194;OP331335.1:33167-32211;OP382479.1:33154-32198;OP422341.1:33167-32211;OP434519.1:33146-32190;OP535324.1:33148-32192;OP535333.1:33257-32301;OP536770.1:33149-32193;OP597769.1:33171-32215;OP604566.1:33150-32194;OP612682.1:33076-32120;OP612684.1:33140-32184;OP612686.1:33140-32184;OP696841.1:30424-29468;OP879722.1:32373-31417;OQ419007.1:32383-31427;OQ419012.1:32375-31419;OQ419031.1:32354-31398;OQ419047.1:32383-31427;OQ451898.1:32235-31279;OQ451900.1:33148-32192;OQ581853.1:33174-32218;OQ581855.1:33255-32299;OQ721951.1:33188-32232;OQ721954.1:33145-32189;OR035652.1:33097-32141;OR035664.1:33097-32141;OR035702.1:33097-32141;OR146272.1:33113-32157;OR146330.1:33158-32202;OR264364.1:33168-32212;OR427332.1:33168-32212;OR459777.1:33152-32196;OR459797.1:33167-32211;OR943698.1:35230-34199;PP098589.2:33149-32193;PP496048.1:33153-32197;PP601206.1:35247-34216;PP776553.1:33074-32118;PP852971.1:33147-32191;PP852983.1:33152-32196;PP853000.1:33114-32158;PP853005.1:33122-32166;PP853007.1:33154-32198;PP853013.1:33128-32172;PP853027.1:33159-32203;PP853045.1:33140-32184;PP853048.1:33159-32203;PP860026.1:33157-32204;PP860027.1:33182-32229;PP860028.1:33123-32170;PP860031.1:33255-32224;PP860033.1:33181-32228;PQ153226.1:33147-32191;PQ178862.2:34100-33069;PQ221506.1:29070-28039;PQ221833.1:35239-34208;PQ340477.1:33130-32174;PQ340478.1:33115-32159;PQ340480.1:33194-32238;PQ340483.1:33178-32222;PQ340485.1:33164-32208;PQ340486.1:33179-32223;PQ340487.1:33204-32248;PQ340489.1:33179-32223;PQ340490.1:33130-32174;PQ340491.1:33188-32232;PQ340493.1:33164-32208;PQ340495.1:33194-32238;PQ340496.1:33227-32271;PQ368489.1:33150-32194;PQ451835.1:33238-32282;PQ451836.1:33170-32214;PQ451838.1:33221-32265;PQ451841.1:33181-32225;PQ451842.1:33133-32177 |
| OG0000114 | AY603973.1:34113-33154;AY741551.1:34117-33158;AY753185.1:34356-33397;DQ011154.1:34333-33374;DQ011155.1:34314-33355;EPI_ISL_13331598:32179-31220;EPI_ISL_13363142:32172-31213;EPI_ISL_13374487:32187-31228;EPI_ISL_13502582:32172-31213;EPI_ISL_13508471:32264-31305;EPI_ISL_13607904:32163-31204;EPI_ISL_13624509:32107-31148;EPI_ISL_13889442:32282-31323;EPI_ISL_13953610:32130-31171;EPI_ISL_14181948:32308-31349;EPI_ISL_14838587:34187-33228;EPI_ISL_15263355:32163-31204;EPI_ISL_15269699:32132-31173;EPI_ISL_15293002:32405-31446;EPI_ISL_15641561:32121-31162;EPI_ISL_15831211:32170-31211;EPI_ISL_15992098:32185-31226;EPI_ISL_16233784:32181-31222;EPI_ISL_16946400:32170-31211;EPI_ISL_17048206:31820-30861;EPI_ISL_17703567:32185-31226;EPI_ISL_17718458:32163-31204;EPI_ISL_18213374:32160-31201;EPI_ISL_18360394:31821-30862;EPI_ISL_18557816:32161-31202;EPI_ISL_18642365:32163-31204;EPI_ISL_18659846:32133-31174;EPI_ISL_18744049:32165-31206;EPI_ISL_19022858:32160-31201;EPI_ISL_19028778:32077-31118;EPI_ISL_19151800:32161-31202;EPI_ISL_19159116:32163-31204;EPI_ISL_19350788:33061-32102;EPI_ISL_19359677:32160-31201;EPI_ISL_19446306:32155-31196;EPI_ISL_19467692:34466-33507;JX878411.1:35181-34222;JX878425.1:34695-33736;JX878426.1:34140-33181;KC257460.1:34111-33152;KJ642612.1:32426-31467;KJ642613.1:34002-33043;KJ642616.1:33807-32848;KJ642618.1:31594-30635;KP849469.1:34503-33544;KP849471.1:34429-33470;LC831698.1:32161-31202;MN648051.1:32383-31424;MN702445.1:27848-26889;MN702446.1:33994-33035;MN702447.1:27359-26400;MN702449.1:27859-26900;MN702452.1:34032-33073;MT903338.1:32326-31367;MT903340.1:32163-31204;MT903341.1:23696-22737;MT903342.1:32178-31219;MT903347.1:34126-33167;ON568298.1:32309-31350;ON622713.1:32395-31436;ON622722.2:32166-31207;ON631963.1:32186-31227;ON649879.1:31996-31037;ON676707.1:32216-31257;ON755039.1:31826-30867;ON808413.1:32166-31207;ON880420.1:32393-31434;ON911481.2:32183-31224;ON918656.1:32198-31239;ON959133.1:32287-31328;ON959136.1:32288-31329;ON983168.1:30862-29903;OP019276.1:32160-31201;OP150924.1:32301-31342;OP160532.1:32126-31167;OP235282.1:32160-31201;OP289782.1:32163-31204;OP331335.1:32180-31221;OP382479.1:32167-31208;OP422341.1:32180-31221;OP434519.1:32159-31200;OP535324.1:32161-31202;OP535333.1:32270-31311;OP536770.1:32162-31203;OP597769.1:32184-31225;OP604566.1:32163-31204;OP612682.1:32089-31130;OP612684.1:32153-31194;OP612686.1:32153-31194;OP696841.1:29437-28478;OP879722.1:31386-30427;OQ419007.1:31396-30437;OQ419012.1:31388-30429;OQ419031.1:31367-30408;OQ419047.1:31396-30437;OQ451898.1:31248-30289;OQ451900.1:32161-31202;OQ581853.1:32187-31228;OQ581855.1:32268-31309;OQ721951.1:32201-31242;OQ721954.1:32158-31199;OR035652.1:32110-31151;OR035664.1:32110-31151;OR035702.1:32110-31151;OR146272.1:32126-31167;OR146330.1:32171-31212;OR264364.1:32181-31222;OR427332.1:32181-31222;OR459777.1:32165-31206;OR459797.1:32180-31221;OR943698.1:34168-33209;PP098589.2:32162-31203;PP496048.1:32166-31207;PP601206.1:34185-33226;PP776553.1:32087-31128;PP852971.1:32160-31201;PP852983.1:32165-31206;PP853000.1:32127-31168;PP853005.1:32135-31176;PP853007.1:32167-31208;PP853013.1:32141-31182;PP853027.1:32172-31213;PP853045.1:32153-31194;PP853048.1:32172-31213;PP860026.1:32173-31214;PP860027.1:32198-31239;PP860028.1:32139-31180;PP860031.1:32193-31234;PP860033.1:32197-31238;PQ153226.1:32160-31201;PQ178862.2:33038-32079;PQ221506.1:28008-27049;PQ221833.1:34177-33218;PQ340477.1:32143-31184;PQ340478.1:32128-31169;PQ340480.1:32207-31248;PQ340483.1:32191-31232;PQ340485.1:32177-31218;PQ340486.1:32192-31233;PQ340487.1:32217-31258;PQ340489.1:32192-31233;PQ340490.1:32143-31184;PQ340491.1:32201-31242;PQ340493.1:32177-31218;PQ340495.1:32207-31248;PQ340496.1:32240-31281;PQ368489.1:32163-31204;PQ451835.1:32251-31292;PQ451836.1:32183-31224;PQ451838.1:32234-31275;PQ451841.1:32194-31235;PQ451842.1:32146-31187 |
| OG0000115 | AY603973.1:31191-30532;AY741551.1:31205-30546;AY753185.1:31434-30775;DQ011154.1:31421-30762;DQ011155.1:31407-30748;EPI_ISL_13331598:29277-28618;EPI_ISL_13363142:29270-28611;EPI_ISL_13374487:29285-28626;EPI_ISL_13502582:29270-28611;EPI_ISL_13508471:29362-28703;EPI_ISL_13607904:29261-28602;EPI_ISL_13624509:29205-28546;EPI_ISL_13889442:29380-28721;EPI_ISL_13953610:29228-28569;EPI_ISL_14181948:29406-28747;EPI_ISL_14838587:31275-30616;EPI_ISL_15263355:29261-28602;EPI_ISL_15269699:29230-28571;EPI_ISL_15293002:29503-28844;EPI_ISL_15641561:29219-28560;EPI_ISL_15831211:29268-28609;EPI_ISL_15992098:29283-28624;EPI_ISL_16233784:29279-28620;EPI_ISL_16946400:29268-28609;EPI_ISL_17048206:28918-28259;EPI_ISL_17703567:29283-28624;EPI_ISL_17718458:29261-28602;EPI_ISL_18213374:29258-28599;EPI_ISL_18360394:28919-28260;EPI_ISL_18557816:29259-28600;EPI_ISL_18642365:29261-28602;EPI_ISL_18659846:29231-28572;EPI_ISL_18744049:29263-28604;EPI_ISL_19022858:29258-28599;EPI_ISL_19028778:29175-28516;EPI_ISL_19151800:29259-28600;EPI_ISL_19159116:29261-28602;EPI_ISL_19350788:30154-29501;EPI_ISL_19359677:29258-28599;EPI_ISL_19446306:29253-28594;EPI_ISL_19467692:31554-30895;JX878411.1:32269-31610;JX878425.1:31783-31124;JX878426.1:31223-30564;KC257460.1:31204-30545;KJ642612.1:29509-28850;KJ642613.1:31090-30431;KJ642616.1:30890-30231;KJ642618.1:28677-28018;KP849469.1:31591-30932;KP849471.1:31512-30853;LC831698.1:29259-28600;MN648051.1:29481-28822;MN702445.1:24936-24277;MN702446.1:31082-30423;MN702447.1:24447-23788;MN702449.1:24947-24288;MN702452.1:31120-30461;MT903338.1:29424-28765;MT903340.1:29261-28602;MT903341.1:20794-20135;MT903342.1:29276-28617;MT903347.1:31189-30530;ON568298.1:29407-28748;ON622713.1:29493-28834;ON622722.2:29264-28605;ON631963.1:29284-28625;ON649879.1:29094-28435;ON676707.1:29314-28655;ON755039.1:28924-28265;ON808413.1:29264-28605;ON880420.1:29491-28832;ON911481.2:29281-28622;ON918656.1:29296-28637;ON959133.1:29385-28726;ON959136.1:29386-28727;ON983168.1:27960-27301;OP019276.1:29258-28599;OP150924.1:29399-28740;OP160532.1:29224-28565;OP235282.1:29258-28599;OP289782.1:29261-28602;OP331335.1:29278-28619;OP382479.1:29265-28606;OP422341.1:29278-28619;OP434519.1:29257-28598;OP535324.1:29259-28600;OP535333.1:29368-28709;OP536770.1:29260-28601;OP597769.1:29282-28623;OP604566.1:29261-28602;OP612682.1:29187-28528;OP612684.1:29251-28592;OP612686.1:29251-28592;OP696841.1:26535-25876;OP879722.1:28484-27825;OQ419007.1:28494-27835;OQ419012.1:28486-27827;OQ419031.1:28465-27806;OQ419047.1:28494-27835;OQ451898.1:28346-27687;OQ451900.1:29259-28600;OQ581853.1:29285-28626;OQ581855.1:29366-28707;OQ721951.1:29299-28640;OQ721954.1:29256-28597;OR035652.1:29208-28549;OR035664.1:29208-28549;OR035702.1:29208-28549;OR146272.1:29224-28565;OR146330.1:29269-28610;OR264364.1:29279-28620;OR427332.1:29279-28620;OR459777.1:29263-28604;OR459797.1:29278-28619;OR943698.1:31251-30592;PP098589.2:29260-28601;PP496048.1:29264-28605;PP601206.1:31273-30614;PP776553.1:29185-28526;PP852971.1:29258-28599;PP852983.1:29263-28604;PP853000.1:29225-28566;PP853005.1:29233-28574;PP853007.1:29265-28606;PP853013.1:29239-28580;PP853027.1:29270-28611;PP853045.1:29251-28592;PP853048.1:29270-28611;PP860026.1:29266-28607;PP860027.1:29276-28617;PP860028.1:29232-28573;PP860031.1:29286-28627;PP860033.1:29275-28616;PQ153226.1:29258-28599;PQ178862.2:30131-29478;PQ221506.1:25084-24434;PQ221833.1:31265-30606;PQ340477.1:29241-28582;PQ340478.1:29226-28567;PQ340480.1:29305-28646;PQ340483.1:29289-28630;PQ340485.1:29275-28616;PQ340486.1:29290-28631;PQ340487.1:29315-28656;PQ340489.1:29290-28631;PQ340490.1:29241-28582;PQ340491.1:29299-28640;PQ340493.1:29275-28616;PQ340495.1:29305-28646;PQ340496.1:29338-28679;PQ368489.1:29261-28602;PQ451835.1:29349-28690;PQ451836.1:29281-28622;PQ451838.1:29332-28673;PQ451841.1:29292-28633;PQ451842.1:29244-28585 |
| OG0000116 | AY603973.1:24858-24196;AY741551.1:24719-24057;AY753185.1:25101-24439;DQ011154.1:25312-24650;DQ011155.1:25298-24636;EPI_ISL_13331598:23174-22512;EPI_ISL_13363142:23167-22505;EPI_ISL_13374487:23182-22520;EPI_ISL_13502582:23167-22505;EPI_ISL_13508471:23259-22597;EPI_ISL_13607904:23158-22496;EPI_ISL_13624509:23102-22440;EPI_ISL_13889442:23277-22615;EPI_ISL_13953610:23125-22463;EPI_ISL_14181948:23303-22641;EPI_ISL_14838587:25166-24504;EPI_ISL_15263355:23158-22496;EPI_ISL_15269699:23127-22465;EPI_ISL_15293002:23400-22738;EPI_ISL_15641561:23116-22454;EPI_ISL_15831211:23165-22503;EPI_ISL_15992098:23180-22518;EPI_ISL_16233784:23176-22514;EPI_ISL_16946400:23165-22503;EPI_ISL_17048206:22815-22153;EPI_ISL_17703567:23180-22518;EPI_ISL_17718458:23158-22496;EPI_ISL_18213374:23155-22493;EPI_ISL_18360394:22816-22154;EPI_ISL_18557816:23156-22494;EPI_ISL_18642365:23158-22496;EPI_ISL_18659846:23128-22466;EPI_ISL_18744049:23160-22498;EPI_ISL_19022858:23155-22493;EPI_ISL_19028778:23072-22410;EPI_ISL_19151800:23156-22494;EPI_ISL_19159116:23158-22496;EPI_ISL_19350788:24051-23389;EPI_ISL_19359677:23155-22493;EPI_ISL_19446306:23150-22488;EPI_ISL_19467692:25445-24783;JX878411.1:26160-25498;JX878425.1:25674-25012;JX878426.1:25114-24452;KC257460.1:25095-24433;KJ642612.1:23400-22738;KJ642613.1:24981-24319;KJ642616.1:24674-24012;KJ642618.1:22568-21906;KP849469.1:25482-24820;KP849471.1:25403-24741;LC831698.1:23156-22494;MN648051.1:23378-22716;MN702445.1:18827-18165;MN702446.1:24973-24311;MN702447.1:18338-17676;MN702449.1:18838-18176;MN702452.1:25011-24349;MT903338.1:23321-22659;MT903340.1:23158-22496;MT903341.1:14691-14029;MT903342.1:23173-22511;MT903347.1:25051-24389;ON568298.1:23304-22642;ON622713.1:23390-22728;ON622722.2:23161-22499;ON631963.1:23181-22519;ON649879.1:22991-22329;ON676707.1:23211-22549;ON755039.1:22821-22159;ON808413.1:23161-22499;ON880420.1:23388-22726;ON911481.2:23178-22516;ON918656.1:23193-22531;ON959133.1:23282-22620;ON959136.1:23283-22621;ON983168.1:21857-21195;OP019276.1:23155-22493;OP150924.1:23296-22634;OP160532.1:23121-22459;OP235282.1:23155-22493;OP289782.1:23158-22496;OP331335.1:23175-22513;OP382479.1:23162-22500;OP422341.1:23175-22513;OP434519.1:23154-22492;OP535324.1:23156-22494;OP535333.1:23265-22603;OP536770.1:23157-22495;OP597769.1:23179-22517;OP604566.1:23158-22496;OP612682.1:23084-22422;OP612684.1:23148-22486;OP612686.1:23148-22486;OP696841.1:20432-19770;OP879722.1:22381-21719;OQ419007.1:22391-21729;OQ419012.1:22383-21721;OQ419031.1:22388-21726;OQ419047.1:22391-21729;OQ451898.1:22243-21581;OQ451900.1:23156-22494;OQ581853.1:23182-22520;OQ581855.1:23263-22601;OQ721951.1:23196-22534;OQ721954.1:23153-22491;OR035652.1:23105-22443;OR035664.1:23105-22443;OR035702.1:23105-22443;OR146272.1:23121-22459;OR146330.1:23166-22504;OR264364.1:23176-22514;OR427332.1:23176-22514;OR459777.1:23160-22498;OR459797.1:23175-22513;OR943698.1:25142-24480;PP098589.2:23157-22495;PP496048.1:23161-22499;PP601206.1:25164-24502;PP776553.1:23106-22444;PP852971.1:23155-22493;PP852983.1:23160-22498;PP853000.1:23122-22460;PP853005.1:23130-22468;PP853007.1:23162-22500;PP853013.1:23136-22474;PP853027.1:23167-22505;PP853045.1:23148-22486;PP853048.1:23167-22505;PP860026.1:23162-22500;PP860027.1:23173-22511;PP860028.1:23129-22467;PP860031.1:23183-22521;PP860033.1:23172-22510;PQ153226.1:23155-22493;PQ178862.2:24028-23366;PQ221506.1:18984-18322;PQ221833.1:25156-24494;PQ340477.1:23138-22476;PQ340478.1:23123-22461;PQ340480.1:23202-22540;PQ340483.1:23186-22524;PQ340485.1:23172-22510;PQ340486.1:23187-22525;PQ340487.1:23212-22550;PQ340489.1:23187-22525;PQ340490.1:23138-22476;PQ340491.1:23196-22534;PQ340493.1:23172-22510;PQ340495.1:23202-22540;PQ340496.1:23235-22573;PQ368489.1:23158-22496;PQ451835.1:23246-22584;PQ451836.1:23178-22516;PQ451838.1:23229-22567;PQ451841.1:23189-22527;PQ451842.1:23141-22479 |
| OG0000117 | AY603973.1:24126-22786;AY741551.1:23987-22647;AY753185.1:24369-23029;DQ011154.1:24580-23252;DQ011155.1:24560-23232;EPI_ISL_13331598:22448-21120;EPI_ISL_13363142:22441-21113;EPI_ISL_13374487:22456-21128;EPI_ISL_13502582:22441-21113;EPI_ISL_13508471:22533-21205;EPI_ISL_13607904:22432-21104;EPI_ISL_13624509:22376-21048;EPI_ISL_13889442:22551-21223;EPI_ISL_13953610:22399-21071;EPI_ISL_14181948:22577-21249;EPI_ISL_14838587:24434-23106;EPI_ISL_15263355:22432-21104;EPI_ISL_15269699:22401-21073;EPI_ISL_15293002:22674-21346;EPI_ISL_15641561:22390-21062;EPI_ISL_15831211:22439-21111;EPI_ISL_15992098:22454-21126;EPI_ISL_16233784:22450-21122;EPI_ISL_16946400:22439-21111;EPI_ISL_17048206:22089-20761;EPI_ISL_17703567:22454-21126;EPI_ISL_17718458:22432-21104;EPI_ISL_18213374:22429-21101;EPI_ISL_18360394:22090-20762;EPI_ISL_18557816:22430-21102;EPI_ISL_18642365:22432-21104;EPI_ISL_18659846:22402-21074;EPI_ISL_18744049:22434-21106;EPI_ISL_19022858:22429-21101;EPI_ISL_19028778:22346-21018;EPI_ISL_19151800:22430-21102;EPI_ISL_19159116:22432-21104;EPI_ISL_19350788:23313-21985;EPI_ISL_19359677:22429-21101;EPI_ISL_19446306:22424-21096;EPI_ISL_19467692:24713-23385;JX878411.1:25428-24100;JX878425.1:24942-23614;JX878426.1:24382-23054;KC257460.1:24363-23035;KJ642612.1:22668-21340;KJ642613.1:24249-22921;KJ642616.1:23942-22602;KJ642618.1:21842-20514;KP849469.1:24750-23422;KP849471.1:24671-23343;LC831698.1:22430-21102;MN648051.1:22652-21324;MN702445.1:18095-16767;MN702446.1:24241-22913;MN702447.1:17606-16275;MN702449.1:18106-16778;MN702452.1:24279-22951;MT903338.1:22595-21267;MT903340.1:22432-21104;MT903341.1:13965-12637;MT903342.1:22447-21119;MT903347.1:24319-22979;ON568298.1:22578-21250;ON622713.1:22664-21336;ON622722.2:22435-21107;ON631963.1:22455-21127;ON649879.1:22265-20937;ON676707.1:22485-21157;ON755039.1:22095-20767;ON808413.1:22435-21107;ON880420.1:22662-21334;ON911481.2:22452-21124;ON918656.1:22467-21139;ON959133.1:22556-21228;ON959136.1:22557-21229;ON983168.1:21131-19803;OP019276.1:22429-21101;OP150924.1:22570-21242;OP160532.1:22395-21067;OP235282.1:22429-21101;OP289782.1:22432-21104;OP331335.1:22449-21121;OP382479.1:22436-21108;OP422341.1:22449-21121;OP434519.1:22428-21100;OP535324.1:22430-21102;OP535333.1:22539-21211;OP536770.1:22431-21103;OP597769.1:22453-21125;OP604566.1:22432-21104;OP612682.1:22358-21030;OP612684.1:22422-21094;OP612686.1:22422-21094;OP696841.1:19706-18378;OP879722.1:21655-20327;OQ419007.1:21665-20337;OQ419012.1:21657-20329;OQ419031.1:21662-20334;OQ419047.1:21665-20337;OQ451898.1:21517-20189;OQ451900.1:22430-21102;OQ581853.1:22456-21128;OQ581855.1:22537-21209;OQ721951.1:22470-21142;OQ721954.1:22427-21099;OR035652.1:22379-21051;OR035664.1:22379-21051;OR035702.1:22379-21051;OR146272.1:22395-21067;OR146330.1:22440-21112;OR264364.1:22450-21122;OR427332.1:22450-21122;OR459777.1:22434-21106;OR459797.1:22449-21121;OR943698.1:24416-23088;PP098589.2:22431-21103;PP496048.1:22435-21107;PP601206.1:24432-23104;PP776553.1:22380-21052;PP852971.1:22429-21101;PP852983.1:22434-21106;PP853000.1:22396-21068;PP853005.1:22404-21076;PP853007.1:22436-21108;PP853013.1:22410-21082;PP853027.1:22441-21113;PP853045.1:22422-21094;PP853048.1:22441-21113;PP860026.1:22436-21108;PP860027.1:22447-21119;PP860028.1:22403-21075;PP860031.1:22457-21129;PP860033.1:22446-21118;PQ153226.1:22429-21101;PQ178862.2:23290-21962;PQ221506.1:18252-16924;PQ221833.1:24424-23096;PQ340477.1:22412-21084;PQ340478.1:22397-21069;PQ340480.1:22476-21148;PQ340483.1:22460-21132;PQ340485.1:22446-21118;PQ340486.1:22461-21133;PQ340487.1:22486-21158;PQ340489.1:22461-21133;PQ340490.1:22412-21084;PQ340491.1:22470-21142;PQ340493.1:22446-21118;PQ340495.1:22476-21148;PQ340496.1:22509-21181;PQ368489.1:22432-21104;PQ451835.1:22520-21192;PQ451836.1:22452-21124;PQ451838.1:22503-21175;PQ451841.1:22463-21135;PQ451842.1:22415-21087 |
| OG0000118 | AY603973.1:18105-17602;AY741551.1:17963-17460;AY753185.1:18351-17848;DQ011154.1:16574-16122;DQ011155.1:16555-16103;EPI_ISL_13331598:16443-15940;EPI_ISL_13363142:16436-15933;EPI_ISL_13374487:16451-15948;EPI_ISL_13502582:16436-15933;EPI_ISL_13508471:16528-16025;EPI_ISL_13607904:16427-15924;EPI_ISL_13624509:16371-15868;EPI_ISL_13889442:16546-16043;EPI_ISL_13953610:16427-15924;EPI_ISL_14181948:16572-16069;EPI_ISL_14838587:16428-15976;EPI_ISL_15263355:16424-15921;EPI_ISL_15269699:16396-15893;EPI_ISL_15293002:16669-16166;EPI_ISL_15641561:16385-15882;EPI_ISL_15831211:16434-15931;EPI_ISL_15992098:16449-15946;EPI_ISL_16233784:16445-15942;EPI_ISL_16946400:16434-15931;EPI_ISL_17048206:16084-15581;EPI_ISL_17703567:16449-15946;EPI_ISL_17718458:16427-15924;EPI_ISL_18213374:16424-15921;EPI_ISL_18360394:16085-15582;EPI_ISL_18557816:16425-15922;EPI_ISL_18642365:16427-15924;EPI_ISL_18659846:16397-15894;EPI_ISL_18744049:16429-15926;EPI_ISL_19022858:16424-15921;EPI_ISL_19028778:16341-15838;EPI_ISL_19151800:16425-15922;EPI_ISL_19159116:16427-15924;EPI_ISL_19350788:16441-15989;EPI_ISL_19359677:16424-15921;EPI_ISL_19446306:16419-15916;EPI_ISL_19467692:16706-16254;JX878411.1:17420-16968;JX878425.1:16934-16482;JX878426.1:16377-15925;KC257460.1:16354-15902;KJ642612.1:14662-14210;KJ642613.1:16243-15791;KJ642616.1:17922-17419;KJ642618.1:13833-13381;KP849469.1:16744-16292;KP849471.1:16665-16213;LC831698.1:16425-15922;MN648051.1:16647-16144;MN702445.1:10085-9633;MN702446.1:16231-15779;MN702447.1:9596-9144;MN702449.1:10093-9641;MN702452.1:16273-15821;MT903338.1:16590-16087;MT903340.1:16427-15924;MT903341.1:7960-7457;MT903342.1:16442-15939;MT903347.1:18299-17796;ON568298.1:16573-16070;ON622713.1:16659-16156;ON622722.2:16430-15927;ON631963.1:16450-15947;ON649879.1:16260-15757;ON676707.1:16480-15977;ON755039.1:16090-15587;ON808413.1:16430-15927;ON880420.1:16657-16154;ON911481.2:16447-15944;ON918656.1:16462-15959;ON959133.1:16551-16048;ON959136.1:16552-16049;ON983168.1:15126-14623;OP019276.1:16424-15921;OP150924.1:16565-16062;OP160532.1:16390-15887;OP235282.1:16424-15921;OP289782.1:16427-15924;OP331335.1:16444-15941;OP382479.1:16431-15928;OP422341.1:16444-15941;OP434519.1:16423-15920;OP535324.1:16425-15922;OP535333.1:16534-16031;OP536770.1:16426-15923;OP597769.1:16448-15945;OP604566.1:16427-15924;OP612682.1:16353-15850;OP612684.1:16417-15914;OP612686.1:16417-15914;OP696841.1:13701-13198;OP879722.1:15650-15147;OQ419007.1:15660-15157;OQ419012.1:15652-15149;OQ419031.1:15657-15154;OQ419047.1:15660-15157;OQ451898.1:15512-15009;OQ451900.1:16425-15922;OQ581853.1:16451-15948;OQ581855.1:16532-16029;OQ721951.1:16465-15962;OQ721954.1:16422-15919;OR035652.1:16374-15871;OR035664.1:16374-15871;OR035702.1:16374-15871;OR146272.1:16390-15887;OR146330.1:16435-15932;OR264364.1:16445-15942;OR427332.1:16445-15942;OR459777.1:16429-15926;OR459797.1:16444-15941;OR943698.1:16411-15959;PP098589.2:16426-15923;PP496048.1:16430-15927;PP601206.1:16426-15974;PP776553.1:16375-15872;PP852971.1:16424-15921;PP852983.1:16429-15926;PP853000.1:16391-15888;PP853005.1:16399-15896;PP853007.1:16431-15928;PP853013.1:16405-15902;PP853027.1:16436-15933;PP853045.1:16435-15932;PP853048.1:16436-15933;PP860026.1:16431-15928;PP860027.1:16430-15927;PP860028.1:16398-15895;PP860031.1:16437-15934;PP860033.1:16429-15926;PQ153226.1:16424-15921;PQ178862.2:16418-15966;PQ221506.1:10248-9796;PQ221833.1:16418-15966;PQ340477.1:16407-15904;PQ340478.1:16392-15889;PQ340480.1:16471-15968;PQ340483.1:16455-15952;PQ340485.1:16441-15938;PQ340486.1:16456-15953;PQ340487.1:16481-15978;PQ340489.1:16456-15953;PQ340490.1:16407-15904;PQ340491.1:16465-15962;PQ340493.1:16441-15938;PQ340495.1:16471-15968;PQ340496.1:16504-16001;PQ368489.1:16427-15924;PQ451835.1:16515-16012;PQ451836.1:16447-15944;PQ451838.1:16498-15995;PQ451841.1:16458-15955;PQ451842.1:16410-15907 |
| OG0000119 | AY603973.1:16965-15073;AY741551.1:16802-14910;AY753185.1:17211-15319;DQ011154.1:15468-13576;DQ011155.1:15448-13556;EPI_ISL_13331598:15282-13390;EPI_ISL_13363142:15275-13383;EPI_ISL_13374487:15290-13398;EPI_ISL_13502582:15275-13383;EPI_ISL_13508471:15367-13475;EPI_ISL_13607904:15266-13374;EPI_ISL_13624509:15210-13318;EPI_ISL_13889442:15385-13493;EPI_ISL_13953610:15266-13374;EPI_ISL_14181948:15411-13519;EPI_ISL_14838587:15322-13430;EPI_ISL_15263355:15263-13371;EPI_ISL_15269699:15235-13343;EPI_ISL_15293002:15508-13616;EPI_ISL_15641561:15224-13332;EPI_ISL_15831211:15273-13381;EPI_ISL_15992098:15288-13396;EPI_ISL_16233784:15284-13392;EPI_ISL_16946400:15273-13381;EPI_ISL_17048206:14923-13031;EPI_ISL_17703567:15288-13396;EPI_ISL_17718458:15266-13374;EPI_ISL_18213374:15263-13371;EPI_ISL_18360394:14924-13032;EPI_ISL_18557816:15264-13372;EPI_ISL_18642365:15266-13374;EPI_ISL_18659846:15236-13344;EPI_ISL_18744049:15268-13376;EPI_ISL_19022858:15263-13371;EPI_ISL_19028778:15180-13288;EPI_ISL_19151800:15264-13372;EPI_ISL_19159116:15266-13374;EPI_ISL_19350788:15334-13442;EPI_ISL_19359677:15263-13371;EPI_ISL_19446306:15258-13366;EPI_ISL_19467692:15600-13708;JX878411.1:16313-14421;JX878425.1:15828-13936;JX878426.1:15270-13378;KC257460.1:15247-13355;KJ642612.1:13555-11663;KJ642613.1:15136-13244;KJ642616.1:16782-14890;KJ642618.1:12208-10835;KP849469.1:15638-13746;KP849471.1:15558-13666;LC831698.1:15264-13372;MN648051.1:15485-13593;MN702445.1:8978-7086;MN702446.1:15124-13232;MN702447.1:8490-6598;MN702449.1:8986-7094;MN702452.1:15167-13275;MT903338.1:15429-13537;MT903340.1:15266-13374;MT903341.1:6799-4907;MT903342.1:15281-13389;MT903347.1:17138-15246;ON568298.1:15412-13520;ON622713.1:15497-13605;ON622722.2:15269-13377;ON631963.1:15289-13397;ON649879.1:15099-13207;ON676707.1:15319-13427;ON755039.1:14929-13037;ON808413.1:15269-13377;ON880420.1:15496-13604;ON911481.2:15286-13394;ON918656.1:15301-13409;ON959133.1:15390-13498;ON959136.1:15391-13499;ON983168.1:13965-12073;OP019276.1:15263-13371;OP150924.1:15404-13512;OP160532.1:15229-13337;OP235282.1:15263-13371;OP289782.1:15266-13374;OP331335.1:15283-13391;OP382479.1:15270-13378;OP422341.1:15283-13391;OP434519.1:15262-13370;OP535324.1:15264-13372;OP535333.1:15373-13481;OP536770.1:15265-13373;OP597769.1:15287-13395;OP604566.1:15266-13374;OP612682.1:15192-13300;OP612684.1:15256-13364;OP612686.1:15256-13364;OP696841.1:12540-10675;OP879722.1:14489-12597;OQ419007.1:14499-12607;OQ419012.1:14491-12599;OQ419031.1:14496-12604;OQ419047.1:14499-12607;OQ451898.1:14351-12459;OQ451900.1:15264-13372;OQ581853.1:15290-13398;OQ581855.1:15371-13479;OQ721951.1:15304-13412;OQ721954.1:15261-13369;OR035652.1:15213-13321;OR035664.1:15213-13321;OR035702.1:15213-13321;OR146272.1:15229-13337;OR146330.1:15274-13382;OR264364.1:15284-13392;OR427332.1:15284-13392;OR459777.1:15268-13376;OR459797.1:15283-13391;OR943698.1:15305-13413;PP098589.2:15265-13373;PP496048.1:15269-13377;PP601206.1:15320-13428;PP776553.1:15214-13322;PP852971.1:15263-13371;PP852983.1:15268-13376;PP853000.1:15230-13338;PP853005.1:15238-13346;PP853007.1:15270-13378;PP853013.1:15244-13352;PP853027.1:15275-13383;PP853045.1:15274-13382;PP853048.1:15275-13383;PP860026.1:15270-13378;PP860027.1:15269-13377;PP860028.1:15237-13345;PP860031.1:15276-13384;PP860033.1:15268-13376;PQ153226.1:15263-13371;PQ178862.2:15311-13419;PQ221506.1:9141-7249;PQ221833.1:15312-13420;PQ340477.1:15246-13354;PQ340478.1:15231-13339;PQ340480.1:15310-13418;PQ340483.1:15294-13402;PQ340485.1:15280-13388;PQ340486.1:15295-13403;PQ340487.1:15320-13428;PQ340489.1:15295-13403;PQ340490.1:15246-13354;PQ340491.1:15304-13412;PQ340493.1:15280-13388;PQ340495.1:15310-13418;PQ340496.1:15343-13451;PQ368489.1:15266-13374;PQ451835.1:15354-13462;PQ451836.1:15286-13394;PQ451838.1:15337-13445;PQ451841.1:15297-13405;PQ451842.1:15249-13357 |
| OG0000120 | AY603973.1:14928-14734;AY741551.1:14763-14569;AY753185.1:15174-14980;DQ011154.1:13416-13222;DQ011155.1:13396-13202;EPI_ISL_13331598:13243-13049;EPI_ISL_13363142:13236-13042;EPI_ISL_13374487:13251-13057;EPI_ISL_13502582:13236-13042;EPI_ISL_13508471:13328-13134;EPI_ISL_13607904:13227-13033;EPI_ISL_13624509:13171-12977;EPI_ISL_13889442:13346-13152;EPI_ISL_13953610:13227-13033;EPI_ISL_14181948:13372-13178;EPI_ISL_14838587:13270-13076;EPI_ISL_15263355:13224-13030;EPI_ISL_15269699:13196-13002;EPI_ISL_15293002:13469-13275;EPI_ISL_15641561:13185-12991;EPI_ISL_15831211:13234-13040;EPI_ISL_15992098:13249-13055;EPI_ISL_16233784:13245-13051;EPI_ISL_16946400:13234-13040;EPI_ISL_17048206:12884-12690;EPI_ISL_17703567:13249-13055;EPI_ISL_17718458:13227-13033;EPI_ISL_18213374:13224-13030;EPI_ISL_18360394:12885-12691;EPI_ISL_18557816:13225-13031;EPI_ISL_18642365:13227-13033;EPI_ISL_18659846:13197-13003;EPI_ISL_18744049:13229-13035;EPI_ISL_19022858:13224-13030;EPI_ISL_19028778:13141-12947;EPI_ISL_19151800:13225-13031;EPI_ISL_19159116:13227-13033;EPI_ISL_19350788:13282-13088;EPI_ISL_19359677:13224-13030;EPI_ISL_19446306:13219-13025;EPI_ISL_19467692:13548-13354;JX878411.1:14261-14067;JX878425.1:13776-13582;JX878426.1:13218-13024;KC257460.1:13195-13001;KJ642612.1:11503-11309;KJ642613.1:13084-12890;KJ642616.1:14745-14551;KJ642618.1:10675-10481;KP849469.1:13586-13392;KP849471.1:13506-13312;LC831698.1:13225-13031;MN648051.1:13446-13252;MN702445.1:6926-6732;MN702446.1:13072-12878;MN702447.1:6438-6244;MN702449.1:6934-6740;MN702452.1:13115-12921;MT903338.1:13390-13196;MT903340.1:13227-13033;MT903341.1:4760-4566;MT903342.1:13242-13048;MT903347.1:15099-14905;ON568298.1:13373-13179;ON622713.1:13458-13264;ON622722.2:13230-13036;ON631963.1:13250-13056;ON649879.1:13060-12866;ON676707.1:13280-13086;ON755039.1:12890-12696;ON808413.1:13230-13036;ON880420.1:13457-13263;ON911481.2:13247-13053;ON918656.1:13262-13068;ON959133.1:13351-13157;ON959136.1:13352-13158;ON983168.1:11926-11732;OP019276.1:13224-13030;OP150924.1:13365-13171;OP160532.1:13190-12996;OP235282.1:13224-13030;OP289782.1:13227-13033;OP331335.1:13244-13050;OP382479.1:13231-13037;OP422341.1:13244-13050;OP434519.1:13223-13029;OP535324.1:13225-13031;OP535333.1:13334-13140;OP536770.1:13226-13032;OP597769.1:13248-13054;OP604566.1:13227-13033;OP612682.1:13153-12959;OP612684.1:13217-13023;OP612686.1:13217-13023;OP696841.1:10528-10334;OP879722.1:12450-12256;OQ419007.1:12460-12266;OQ419012.1:12452-12258;OQ419031.1:12457-12263;OQ419047.1:12460-12266;OQ451898.1:12312-12118;OQ451900.1:13225-13031;OQ581853.1:13251-13057;OQ581855.1:13332-13138;OQ721951.1:13265-13071;OQ721954.1:13222-13028;OR035652.1:13174-12980;OR035664.1:13174-12980;OR035702.1:13174-12980;OR146272.1:13190-12996;OR146330.1:13235-13041;OR264364.1:13245-13051;OR427332.1:13245-13051;OR459777.1:13229-13035;OR459797.1:13244-13050;OR943698.1:13253-13059;PP098589.2:13226-13032;PP496048.1:13230-13036;PP601206.1:13268-13074;PP776553.1:13175-12981;PP852971.1:13224-13030;PP852983.1:13229-13035;PP853000.1:13191-12997;PP853005.1:13199-13005;PP853007.1:13231-13037;PP853013.1:13205-13011;PP853027.1:13236-13042;PP853045.1:13235-13041;PP853048.1:13236-13042;PP860026.1:13231-13037;PP860027.1:13230-13036;PP860028.1:13198-13004;PP860031.1:13237-13043;PP860033.1:13229-13035;PQ153226.1:13224-13030;PQ178862.2:13259-13065;PQ221506.1:7089-6895;PQ221833.1:13260-13066;PQ340477.1:13207-13013;PQ340478.1:13192-12998;PQ340480.1:13271-13077;PQ340483.1:13255-13061;PQ340485.1:13241-13047;PQ340486.1:13256-13062;PQ340487.1:13281-13087;PQ340489.1:13256-13062;PQ340490.1:13207-13013;PQ340491.1:13265-13071;PQ340493.1:13241-13047;PQ340495.1:13271-13077;PQ340496.1:13304-13110;PQ368489.1:13227-13033;PQ451835.1:13315-13121;PQ451836.1:13247-13053;PQ451838.1:13298-13104;PQ451841.1:13258-13064;PQ451842.1:13210-13016 |
| OG0000121 | AY603973.1:151310-150270;AY741551.1:151369-150329;AY753185.1:151565-150525;DQ011154.1:151414-150374;DQ011155.1:151375-150335;EPI_ISL_13331598:149406-148366;EPI_ISL_13363142:149393-148353;EPI_ISL_13374487:149410-148370;EPI_ISL_13502582:149405-148365;EPI_ISL_13508471:149488-148448;EPI_ISL_13607904:149384-148344;EPI_ISL_13624509:149328-148288;EPI_ISL_13889442:149505-148465;EPI_ISL_13953610:149304-148396;EPI_ISL_14181948:149534-148494;EPI_ISL_14838587:151274-150234;EPI_ISL_15263355:149387-148347;EPI_ISL_15269699:149356-148316;EPI_ISL_15293002:149625-148585;EPI_ISL_15641561:149342-148302;EPI_ISL_15831211:149395-148355;EPI_ISL_15992098:149419-148379;EPI_ISL_16233784:149408-148368;EPI_ISL_16946400:149397-148357;EPI_ISL_17048206:149044-148004;EPI_ISL_17703567:149409-148369;EPI_ISL_17718458:149372-148464;EPI_ISL_18213374:149383-148343;EPI_ISL_18360394:148690-147650;EPI_ISL_18557816:149384-148344;EPI_ISL_18642365:149381-148341;EPI_ISL_18659846:149357-148317;EPI_ISL_18744049:149389-148481;EPI_ISL_19022858:149363-148323;EPI_ISL_19028778:149298-148258;EPI_ISL_19151800:149381-148341;EPI_ISL_19159116:149384-148344;EPI_ISL_19350788:150051-149011;EPI_ISL_19359677:149381-148341;EPI_ISL_19446306:149386-148346;EPI_ISL_19467692:151545-150505;JX878411.1:152255-151215;JX878425.1:151762-150722;JX878426.1:151197-150157;KC257460.1:151158-150118;KJ642612.1:149456-148416;KJ642613.1:151041-150001;KJ642616.1:151041-150001;KJ642618.1:148654-147614;KP849469.1:151580-150540;KP849471.1:151505-150465;LC831698.1:149382-148342;MN648051.1:149608-148568;MN702445.1:144909-143869;MN702446.1:151073-150033;MN702447.1:144444-143404;MN702449.1:144947-143907;MN702452.1:151114-150074;MT903338.1:149541-148501;MT903340.1:149394-148354;MT903341.1:140935-139895;MT903342.1:149431-148391;MT903347.1:151389-150349;ON568298.1:149535-148495;ON622713.1:149616-148576;ON622722.2:149387-148347;ON631963.1:149413-148373;ON649879.1:149219-148179;ON676707.1:149433-148525;ON755039.1:149041-148001;ON808413.1:149378-148338;ON880420.1:149614-148574;ON911481.2:149399-148359;ON918656.1:149421-148381;ON959133.1:149510-148470;ON959136.1:149511-148471;ON983168.1:148087-147047;OP019276.1:149381-148341;OP150924.1:149524-148484;OP160532.1:149347-148307;OP235282.1:149381-148341;OP289782.1:149384-148344;OP331335.1:149407-148499;OP382479.1:149391-148351;OP422341.1:149404-148364;OP434519.1:149380-148340;OP535324.1:149394-148354;OP535333.1:149501-148461;OP536770.1:149384-148344;OP597769.1:149418-148378;OP604566.1:149384-148344;OP612682.1:149319-148279;OP612684.1:149370-148330;OP612686.1:149372-148332;OP696841.1:146660-145620;OP879722.1:148607-147567;OQ419007.1:148610-147570;OQ419012.1:148584-147544;OQ419031.1:148588-147548;OQ419047.1:148590-147550;OQ451898.1:148471-147431;OQ451900.1:149384-148344;OQ581853.1:149410-148370;OQ581855.1:149491-148451;OQ721951.1:149411-148371;OQ721954.1:149384-148344;OR035652.1:149330-148290;OR035664.1:149334-148294;OR035702.1:149331-148291;OR146272.1:149350-148310;OR146330.1:149394-148354;OR264364.1:149405-148365;OR427332.1:149405-148365;OR459777.1:149377-148469;OR459797.1:149403-148363;OR943698.1:151263-150223;PP098589.2:149383-148343;PP496048.1:149387-148347;PP601206.1:151227-150187;PP776553.1:149307-148267;PP852971.1:149375-148467;PP852983.1:149392-148484;PP853000.1:149343-148435;PP853005.1:149351-148443;PP853007.1:149394-148486;PP853013.1:149366-148458;PP853027.1:149399-148491;PP853045.1:149359-148451;PP853048.1:149384-148476;PP860026.1:149388-148348;PP860027.1:149431-148391;PP860028.1:149351-148311;PP860031.1:149413-148373;PP860033.1:149414-148374;PQ153226.1:149381-148341;PQ178862.2:150030-148990;PQ221506.1:145044-144004;PQ221833.1:151220-150180;PQ340477.1:149368-148328;PQ340478.1:149350-148310;PQ340480.1:149428-148388;PQ340483.1:149414-148374;PQ340485.1:149397-148357;PQ340486.1:149415-148375;PQ340487.1:149439-148399;PQ340489.1:149414-148374;PQ340490.1:149366-148326;PQ340491.1:149421-148381;PQ340493.1:149405-148365;PQ340495.1:149431-148391;PQ340496.1:149466-148426;PQ368489.1:149397-148357;PQ451835.1:149471-148431;PQ451836.1:149409-148369;PQ451838.1:149459-148419;PQ451841.1:149422-148382;PQ451842.1:149368-148328 |
| OG0000122 | AY603973.1:148712-148047;AY741551.1:148757-148092;AY753185.1:148955-148290;DQ011154.1:148772-148107;DQ011155.1:148741-148076;EPI_ISL_13331598:146789-146124;EPI_ISL_13363142:146776-146111;EPI_ISL_13374487:146793-146128;EPI_ISL_13502582:146788-146123;EPI_ISL_13508471:146871-146206;EPI_ISL_13607904:146767-146102;EPI_ISL_13624509:146711-146046;EPI_ISL_13889442:146888-146223;EPI_ISL_13953610:146694-146029;EPI_ISL_14181948:146917-146252;EPI_ISL_14838587:148638-147973;EPI_ISL_15263355:146770-146105;EPI_ISL_15269699:146739-146074;EPI_ISL_15293002:147008-146343;EPI_ISL_15641561:146725-146060;EPI_ISL_15831211:146778-146113;EPI_ISL_15992098:146802-146137;EPI_ISL_16233784:146791-146126;EPI_ISL_16946400:146780-146115;EPI_ISL_17048206:146427-145762;EPI_ISL_17703567:146792-146127;EPI_ISL_17718458:146762-146097;EPI_ISL_18213374:146766-146101;EPI_ISL_18360394:146424-145759;EPI_ISL_18557816:146767-146102;EPI_ISL_18642365:146764-146099;EPI_ISL_18659846:146740-146075;EPI_ISL_18744049:146779-146114;EPI_ISL_19022858:146746-146081;EPI_ISL_19028778:146681-146016;EPI_ISL_19151800:146764-146099;EPI_ISL_19159116:146767-146102;EPI_ISL_19350788:147417-146752;EPI_ISL_19359677:146764-146099;EPI_ISL_19446306:146769-146104;EPI_ISL_19467692:148902-148237;JX878411.1:149618-148953;JX878425.1:149126-148461;JX878426.1:148591-147926;KC257460.1:148518-147853;KJ642612.1:146848-146183;KJ642613.1:148407-147742;KJ642616.1:148437-147772;KJ642618.1:146034-145369;KP849469.1:148939-148274;KP849471.1:148867-148202;LC831698.1:146765-146100;MN648051.1:146991-146326;MN702445.1:142264-141599;MN702446.1:148427-147762;MN702447.1:141801-141136;MN702449.1:142290-141625;MN702452.1:148474-147809;MT903338.1:146943-146278;MT903340.1:146777-146112;MT903341.1:138311-137646;MT903342.1:146795-146130;MT903347.1:148778-148113;ON568298.1:146918-146253;ON622713.1:146999-146334;ON622722.2:146770-146105;ON631963.1:146796-146131;ON649879.1:146602-145937;ON676707.1:146823-146158;ON755039.1:146424-145759;ON808413.1:146761-146096;ON880420.1:146997-146332;ON911481.2:146782-146117;ON918656.1:146804-146139;ON959133.1:146893-146228;ON959136.1:146894-146229;ON983168.1:145469-144804;OP019276.1:146764-146099;OP150924.1:146907-146242;OP160532.1:146730-146065;OP235282.1:146764-146099;OP289782.1:146767-146102;OP331335.1:146797-146132;OP382479.1:146774-146109;OP422341.1:146787-146122;OP434519.1:146763-146098;OP535324.1:146777-146112;OP535333.1:146884-146219;OP536770.1:146767-146102;OP597769.1:146801-146136;OP604566.1:146767-146102;OP612682.1:146702-146037;OP612684.1:146753-146088;OP612686.1:146755-146090;OP696841.1:144043-143378;OP879722.1:145990-145325;OQ419007.1:145993-145328;OQ419012.1:145967-145302;OQ419031.1:145971-145306;OQ419047.1:145973-145308;OQ451898.1:145854-145189;OQ451900.1:146767-146102;OQ581853.1:146793-146128;OQ581855.1:146874-146209;OQ721951.1:146794-146129;OQ721954.1:146767-146102;OR035652.1:146713-146048;OR035664.1:146717-146052;OR035702.1:146714-146049;OR146272.1:146733-146068;OR146330.1:146777-146112;OR264364.1:146788-146123;OR427332.1:146788-146123;OR459777.1:146767-146102;OR459797.1:146786-146121;OR943698.1:148649-147984;PP098589.2:146766-146101;PP496048.1:146770-146105;PP601206.1:148608-147943;PP776553.1:146690-146025;PP852971.1:146765-146100;PP852983.1:146782-146117;PP853000.1:146733-146068;PP853005.1:146741-146076;PP853007.1:146784-146119;PP853013.1:146756-146091;PP853027.1:146789-146124;PP853045.1:146749-146084;PP853048.1:146774-146109;PP860026.1:146778-146113;PP860027.1:146833-146168;PP860028.1:146741-146076;PP860031.1:146815-146150;PP860033.1:146816-146151;PQ153226.1:146764-146099;PQ178862.2:147397-146732;PQ221506.1:142437-141772;PQ221833.1:148611-147946;PQ340477.1:146751-146086;PQ340478.1:146733-146068;PQ340480.1:146811-146146;PQ340483.1:146797-146132;PQ340485.1:146780-146115;PQ340486.1:146798-146133;PQ340487.1:146822-146157;PQ340489.1:146797-146132;PQ340490.1:146749-146084;PQ340491.1:146804-146139;PQ340493.1:146788-146123;PQ340495.1:146814-146149;PQ340496.1:146849-146184;PQ368489.1:146780-146115;PQ451835.1:146854-146189;PQ451836.1:146792-146127;PQ451838.1:146842-146177;PQ451841.1:146805-146140;PQ451842.1:146751-146086 |
| OG0000123 | AY603973.1:147311-146478;AY741551.1:147356-146523;AY753185.1:147554-146721;DQ011154.1:147380-146547;DQ011155.1:147349-146516;EPI_ISL_13331598:145418-144585;EPI_ISL_13363142:145405-144572;EPI_ISL_13374487:145422-144589;EPI_ISL_13502582:145417-144584;EPI_ISL_13508471:145500-144667;EPI_ISL_13607904:145396-144563;EPI_ISL_13624509:145340-144507;EPI_ISL_13889442:145517-144684;EPI_ISL_13953610:145323-144490;EPI_ISL_14181948:145546-144713;EPI_ISL_14838587:147246-146413;EPI_ISL_15263355:145399-144566;EPI_ISL_15269699:145368-144535;EPI_ISL_15293002:145637-144804;EPI_ISL_15641561:145354-144521;EPI_ISL_15831211:145407-144574;EPI_ISL_15992098:145431-144598;EPI_ISL_16233784:145420-144587;EPI_ISL_16946400:145409-144576;EPI_ISL_17048206:145056-144223;EPI_ISL_17703567:145421-144588;EPI_ISL_17718458:145391-144558;EPI_ISL_18213374:145395-144562;EPI_ISL_18360394:145053-144220;EPI_ISL_18557816:145396-144563;EPI_ISL_18642365:145393-144560;EPI_ISL_18659846:145369-144536;EPI_ISL_18744049:145408-144575;EPI_ISL_19022858:145375-144542;EPI_ISL_19028778:145310-144477;EPI_ISL_19151800:145393-144560;EPI_ISL_19159116:145396-144563;EPI_ISL_19350788:146025-145192;EPI_ISL_19359677:145393-144560;EPI_ISL_19446306:145398-144565;EPI_ISL_19467692:147510-146677;JX878411.1:148226-147393;JX878425.1:147734-146901;JX878426.1:147199-146366;KC257460.1:147126-146293;KJ642612.1:145456-144623;KJ642613.1:147015-146182;KJ642616.1:147036-146203;KJ642618.1:144643-143810;KP849469.1:147547-146714;KP849471.1:147475-146642;LC831698.1:145394-144561;MN648051.1:145620-144787;MN702445.1:140872-140039;MN702446.1:147035-146202;MN702447.1:140409-139576;MN702449.1:140898-140065;MN702452.1:147082-146249;MT903338.1:145572-144739;MT903340.1:145406-144573;MT903341.1:136940-136107;MT903342.1:145424-144591;MT903347.1:147377-146544;ON568298.1:145547-144714;ON622713.1:145628-144795;ON622722.2:145399-144566;ON631963.1:145425-144592;ON649879.1:145231-144398;ON676707.1:145452-144619;ON755039.1:145053-144220;ON808413.1:145390-144557;ON880420.1:145626-144793;ON911481.2:145411-144578;ON918656.1:145433-144600;ON959133.1:145522-144689;ON959136.1:145523-144690;ON983168.1:144098-143265;OP019276.1:145393-144560;OP150924.1:145536-144703;OP160532.1:145359-144526;OP235282.1:145393-144560;OP289782.1:145396-144563;OP331335.1:145426-144593;OP382479.1:145403-144570;OP422341.1:145416-144583;OP434519.1:145392-144559;OP535324.1:145406-144573;OP535333.1:145513-144680;OP536770.1:145396-144563;OP597769.1:145430-144597;OP604566.1:145396-144563;OP612682.1:145331-144498;OP612684.1:145382-144549;OP612686.1:145384-144551;OP696841.1:142672-141839;OP879722.1:144619-143786;OQ419007.1:144622-143789;OQ419012.1:144596-143763;OQ419031.1:144600-143767;OQ419047.1:144602-143769;OQ451898.1:144483-143650;OQ451900.1:145396-144563;OQ581853.1:145422-144589;OQ581855.1:145503-144670;OQ721951.1:145423-144590;OQ721954.1:145396-144563;OR035652.1:145342-144509;OR035664.1:145346-144513;OR035702.1:145343-144510;OR146272.1:145362-144529;OR146330.1:145406-144573;OR264364.1:145417-144584;OR427332.1:145417-144584;OR459777.1:145396-144563;OR459797.1:145415-144582;OR943698.1:147257-146424;PP098589.2:145395-144562;PP496048.1:145399-144566;PP601206.1:147216-146383;PP776553.1:145319-144486;PP852971.1:145394-144561;PP852983.1:145411-144578;PP853000.1:145362-144529;PP853005.1:145370-144537;PP853007.1:145413-144580;PP853013.1:145385-144552;PP853027.1:145418-144585;PP853045.1:145378-144545;PP853048.1:145403-144570;PP860026.1:145408-144575;PP860027.1:145462-144629;PP860028.1:145371-144538;PP860031.1:145444-144611;PP860033.1:145445-144612;PQ153226.1:145393-144560;PQ178862.2:146005-145172;PQ221506.1:141045-140212;PQ221833.1:147219-146386;PQ340477.1:145380-144547;PQ340478.1:145362-144529;PQ340480.1:145440-144607;PQ340483.1:145426-144593;PQ340485.1:145409-144576;PQ340486.1:145427-144594;PQ340487.1:145451-144618;PQ340489.1:145426-144593;PQ340490.1:145378-144545;PQ340491.1:145433-144600;PQ340493.1:145417-144584;PQ340495.1:145443-144610;PQ340496.1:145478-144645;PQ368489.1:145409-144576;PQ451835.1:145483-144650;PQ451836.1:145421-144588;PQ451838.1:145471-144638;PQ451841.1:145434-144601;PQ451842.1:145380-144547 |
| OG0000124 | AY603973.1:144266-144036;AY741551.1:144311-144081;AY753185.1:144509-144279;DQ011154.1:144335-144105;DQ011155.1:144304-144074;EPI_ISL_13331598:142373-142143;EPI_ISL_13363142:142360-142130;EPI_ISL_13374487:142377-142147;EPI_ISL_13502582:142372-142142;EPI_ISL_13508471:142455-142225;EPI_ISL_13607904:142351-142121;EPI_ISL_13624509:142295-142065;EPI_ISL_13889442:142472-142242;EPI_ISL_13953610:142278-142048;EPI_ISL_14181948:142501-142271;EPI_ISL_14838587:144201-143971;EPI_ISL_15263355:142354-142124;EPI_ISL_15269699:142323-142093;EPI_ISL_15293002:142592-142362;EPI_ISL_15641561:142309-142079;EPI_ISL_15831211:142362-142132;EPI_ISL_15992098:142386-142156;EPI_ISL_16233784:142375-142145;EPI_ISL_16946400:142364-142134;EPI_ISL_17048206:142011-141781;EPI_ISL_17703567:142376-142146;EPI_ISL_17718458:142346-142116;EPI_ISL_18213374:142350-142120;EPI_ISL_18360394:142008-141778;EPI_ISL_18557816:142351-142121;EPI_ISL_18642365:142348-142118;EPI_ISL_18659846:142324-142094;EPI_ISL_18744049:142363-142133;EPI_ISL_19022858:142330-142100;EPI_ISL_19028778:142265-142035;EPI_ISL_19151800:142348-142118;EPI_ISL_19159116:142351-142121;EPI_ISL_19350788:143027-142797;EPI_ISL_19359677:142348-142118;EPI_ISL_19446306:142353-142123;EPI_ISL_19467692:144465-144235;JX878411.1:145180-144950;JX878425.1:144688-144458;JX878426.1:144141-143911;KC257460.1:144081-143851;KJ642612.1:142410-142180;KJ642613.1:143970-143740;KJ642616.1:143991-143761;KJ642618.1:141552-141367;KP849469.1:144502-144272;KP849471.1:144429-144199;LC831698.1:142349-142119;MN648051.1:142575-142345;MN702445.1:137827-137597;MN702446.1:143990-143760;MN702447.1:137364-137134;MN702449.1:137853-137623;MN702452.1:144037-143807;MT903338.1:142527-142297;MT903340.1:142361-142131;MT903341.1:133895-133665;MT903342.1:142379-142149;MT903347.1:144332-144102;ON568298.1:142502-142272;ON622713.1:142583-142353;ON622722.2:142354-142124;ON631963.1:142380-142150;ON649879.1:142186-141956;ON676707.1:142407-142177;ON755039.1:142008-141778;ON808413.1:142345-142115;ON880420.1:142581-142351;ON911481.2:142366-142136;ON918656.1:142388-142158;ON959133.1:142477-142247;ON959136.1:142478-142248;ON983168.1:141053-140823;OP019276.1:142348-142118;OP150924.1:142491-142261;OP160532.1:142314-142084;OP235282.1:142348-142118;OP289782.1:142351-142121;OP331335.1:142381-142151;OP382479.1:142358-142128;OP422341.1:142371-142141;OP434519.1:142347-142117;OP535324.1:142361-142131;OP535333.1:142468-142238;OP536770.1:142351-142121;OP597769.1:142385-142155;OP604566.1:142351-142121;OP612682.1:142286-142056;OP612684.1:142337-142107;OP612686.1:142339-142109;OP696841.1:139627-139397;OP879722.1:141574-141344;OQ419007.1:141577-141347;OQ419012.1:141551-141321;OQ419031.1:141555-141325;OQ419047.1:141557-141327;OQ451898.1:141438-141208;OQ451900.1:142351-142121;OQ581853.1:142377-142147;OQ581855.1:142458-142228;OQ721951.1:142378-142148;OQ721954.1:142351-142121;OR035652.1:142297-142067;OR035664.1:142301-142071;OR035702.1:142298-142068;OR146272.1:142317-142087;OR146330.1:142361-142131;OR264364.1:142372-142142;OR427332.1:142372-142142;OR459777.1:142351-142121;OR459797.1:142370-142140;OR943698.1:144211-143981;PP098589.2:142350-142120;PP496048.1:142354-142124;PP601206.1:144171-143941;PP776553.1:142274-142044;PP852971.1:142349-142119;PP852983.1:142366-142136;PP853000.1:142317-142087;PP853005.1:142325-142095;PP853007.1:142368-142138;PP853013.1:142340-142110;PP853027.1:142373-142143;PP853045.1:142333-142103;PP853048.1:142358-142128;PP860026.1:142363-142133;PP860027.1:142417-142187;PP860028.1:142326-142096;PP860031.1:142399-142169;PP860033.1:142400-142170;PQ153226.1:142348-142118;PQ178862.2:143007-142777;PQ221506.1:137999-137769;PQ221833.1:144174-143944;PQ340477.1:142335-142105;PQ340478.1:142317-142087;PQ340480.1:142395-142165;PQ340483.1:142381-142151;PQ340485.1:142364-142134;PQ340486.1:142382-142152;PQ340487.1:142406-142176;PQ340489.1:142381-142151;PQ340490.1:142333-142103;PQ340491.1:142388-142158;PQ340493.1:142372-142142;PQ340495.1:142398-142168;PQ340496.1:142433-142203;PQ368489.1:142364-142134;PQ451835.1:142438-142208;PQ451836.1:142376-142146;PQ451838.1:142426-142196;PQ451841.1:142389-142159;PQ451842.1:142335-142105 |
| OG0000125 | AY603973.1:141521-141285;AY741551.1:141566-141330;AY753185.1:141764-141528;DQ011154.1:141581-141348;DQ011155.1:141550-141317;EPI_ISL_13331598:139619-139383;EPI_ISL_13363142:139606-139370;EPI_ISL_13374487:139623-139387;EPI_ISL_13502582:139618-139382;EPI_ISL_13508471:139701-139465;EPI_ISL_13607904:139597-139361;EPI_ISL_13624509:139541-139305;EPI_ISL_13889442:139718-139482;EPI_ISL_13953610:139524-139288;EPI_ISL_14181948:139747-139511;EPI_ISL_14838587:141447-141214;EPI_ISL_15263355:139600-139364;EPI_ISL_15269699:139569-139333;EPI_ISL_15293002:139838-139602;EPI_ISL_15641561:139555-139319;EPI_ISL_15831211:139608-139372;EPI_ISL_15992098:139632-139396;EPI_ISL_16233784:139621-139385;EPI_ISL_16946400:139610-139374;EPI_ISL_17048206:139257-139021;EPI_ISL_17703567:139622-139386;EPI_ISL_17718458:139592-139356;EPI_ISL_18213374:139596-139360;EPI_ISL_18360394:139255-139019;EPI_ISL_18557816:139597-139361;EPI_ISL_18642365:139594-139358;EPI_ISL_18659846:139570-139334;EPI_ISL_18744049:139609-139373;EPI_ISL_19022858:139594-139358;EPI_ISL_19028778:139511-139275;EPI_ISL_19151800:139594-139358;EPI_ISL_19159116:139597-139361;EPI_ISL_19350788:140282-140046;EPI_ISL_19359677:139594-139358;EPI_ISL_19446306:139599-139363;EPI_ISL_19467692:141720-141487;JX878411.1:142426-142193;JX878425.1:141934-141701;JX878426.1:141396-141163;KC257460.1:141327-141094;KJ642612.1:139665-139432;KJ642613.1:141216-140983;KJ642616.1:141246-141010;KJ642618.1:138852-138619;KP849469.1:141748-141515;KP849471.1:141675-141442;LC831698.1:139595-139359;MN648051.1:139821-139585;MN702445.1:135082-134849;MN702446.1:141245-141012;MN702447.1:134601-134368;MN702449.1:135108-134875;MN702452.1:141283-141050;MT903338.1:139773-139537;MT903340.1:139607-139371;MT903341.1:131141-130905;MT903342.1:139625-139389;MT903347.1:141587-141351;ON568298.1:139748-139512;ON622713.1:139829-139593;ON622722.2:139600-139364;ON631963.1:139626-139390;ON649879.1:139432-139196;ON676707.1:139653-139417;ON755039.1:139254-139018;ON808413.1:139591-139355;ON880420.1:139827-139591;ON911481.2:139612-139376;ON918656.1:139634-139398;ON959133.1:139723-139487;ON959136.1:139724-139488;ON983168.1:138299-138063;OP019276.1:139594-139358;OP150924.1:139737-139501;OP160532.1:139560-139324;OP235282.1:139594-139358;OP289782.1:139597-139361;OP331335.1:139627-139391;OP382479.1:139604-139368;OP422341.1:139617-139381;OP434519.1:139593-139357;OP535324.1:139607-139371;OP535333.1:139714-139478;OP536770.1:139597-139361;OP597769.1:139631-139395;OP604566.1:139597-139361;OP612682.1:139532-139296;OP612684.1:139583-139347;OP612686.1:139585-139349;OP696841.1:136873-136637;OP879722.1:138820-138584;OQ419007.1:138823-138587;OQ419012.1:138797-138561;OQ419031.1:138801-138565;OQ419047.1:138803-138567;OQ451898.1:138684-138448;OQ451900.1:139597-139361;OQ581853.1:139623-139387;OQ581855.1:139704-139468;OQ721951.1:139624-139388;OQ721954.1:139597-139361;OR035652.1:139543-139307;OR035664.1:139547-139311;OR035702.1:139544-139308;OR146272.1:139563-139327;OR146330.1:139607-139371;OR264364.1:139618-139382;OR427332.1:139618-139382;OR459777.1:139597-139361;OR459797.1:139616-139380;OR943698.1:141466-141233;PP098589.2:139596-139360;PP496048.1:139600-139364;PP601206.1:141426-141193;PP776553.1:139520-139284;PP852971.1:139595-139359;PP852983.1:139612-139376;PP853000.1:139563-139327;PP853005.1:139571-139335;PP853007.1:139614-139378;PP853013.1:139586-139350;PP853027.1:139619-139383;PP853045.1:139579-139343;PP853048.1:139604-139368;PP860026.1:139618-139382;PP860027.1:139672-139436;PP860028.1:139581-139345;PP860031.1:139654-139418;PP860033.1:139655-139419;PQ153226.1:139594-139358;PQ178862.2:140262-140026;PQ221506.1:135254-135021;PQ221833.1:141429-141196;PQ340477.1:139581-139345;PQ340478.1:139563-139327;PQ340480.1:139641-139405;PQ340483.1:139627-139391;PQ340485.1:139610-139374;PQ340486.1:139628-139392;PQ340487.1:139652-139416;PQ340489.1:139627-139391;PQ340490.1:139579-139343;PQ340491.1:139634-139398;PQ340493.1:139618-139382;PQ340495.1:139644-139408;PQ340496.1:139679-139443;PQ368489.1:139610-139374;PQ451835.1:139684-139448;PQ451836.1:139622-139386;PQ451838.1:139672-139436;PQ451841.1:139635-139399;PQ451842.1:139581-139345 |
| OG0000126 | AY603973.1:139580-138081;AY741551.1:139625-138105;AY753185.1:139823-138324;DQ011154.1:139643-138084;DQ011155.1:139612-138062;EPI_ISL_13331598:137678-136149;EPI_ISL_13363142:137665-136136;EPI_ISL_13374487:137682-136153;EPI_ISL_13502582:137677-136148;EPI_ISL_13508471:137760-136231;EPI_ISL_13607904:137656-136127;EPI_ISL_13624509:137600-136071;EPI_ISL_13889442:137777-136248;EPI_ISL_13953610:137583-136054;EPI_ISL_14181948:137806-136277;EPI_ISL_14838587:139509-137938;EPI_ISL_15263355:137659-136130;EPI_ISL_15269699:137628-136099;EPI_ISL_15293002:137897-136368;EPI_ISL_15641561:137614-136085;EPI_ISL_15831211:137667-136138;EPI_ISL_15992098:137691-136162;EPI_ISL_16233784:137680-136151;EPI_ISL_16946400:137669-136140;EPI_ISL_17048206:137316-135787;EPI_ISL_17703567:137681-136152;EPI_ISL_17718458:137651-136137;EPI_ISL_18213374:137655-136126;EPI_ISL_18360394:137314-135785;EPI_ISL_18557816:137656-136127;EPI_ISL_18642365:137653-136127;EPI_ISL_18659846:137629-136100;EPI_ISL_18744049:137668-136139;EPI_ISL_19022858:137653-136124;EPI_ISL_19028778:137570-136041;EPI_ISL_19151800:137653-136127;EPI_ISL_19159116:137656-136127;EPI_ISL_19350788:138341-136809;EPI_ISL_19359677:137653-136124;EPI_ISL_19446306:137658-136129;EPI_ISL_19467692:139782-138220;JX878411.1:140488-138929;JX878425.1:139996-138446;JX878426.1:139458-137896;KC257460.1:139389-137860;KJ642612.1:137727-136180;KJ642613.1:139278-137740;KJ642616.1:139305-137782;KJ642618.1:136914-135412;KP849469.1:139810-138254;KP849471.1:139737-138820;LC831698.1:137654-136125;MN648051.1:137880-136351;MN702445.1:133144-131597;MN702446.1:139307-137742;MN702447.1:132663-131110;MN702449.1:133170-131608;MN702452.1:139345-137783;MT903338.1:137832-136303;MT903340.1:137666-136137;MT903341.1:129200-127671;MT903342.1:137684-136155;MT903347.1:139646-138111;ON568298.1:137807-136278;ON622713.1:137888-136359;ON622722.2:137659-136130;ON631963.1:137685-136156;ON649879.1:137491-135962;ON676707.1:137712-136192;ON755039.1:137313-135784;ON808413.1:137650-136124;ON880420.1:137886-136357;ON911481.2:137671-136142;ON918656.1:137693-136164;ON959133.1:137782-136253;ON959136.1:137783-136254;ON983168.1:136358-134829;OP019276.1:137653-136124;OP150924.1:137796-136267;OP160532.1:137619-136090;OP235282.1:137653-136124;OP289782.1:137656-136127;OP331335.1:137686-136157;OP382479.1:137663-136134;OP422341.1:137676-136147;OP434519.1:137652-136123;OP535324.1:137666-136137;OP535333.1:137773-136244;OP536770.1:137656-136127;OP597769.1:137690-136161;OP604566.1:137656-136127;OP612682.1:137591-136062;OP612684.1:137642-136125;OP612686.1:137644-136115;OP696841.1:134932-133403;OP879722.1:136879-135353;OQ419007.1:136882-135356;OQ419012.1:136856-135327;OQ419031.1:136860-135331;OQ419047.1:136862-135333;OQ451898.1:136743-135214;OQ451900.1:137656-136127;OQ581853.1:137682-136153;OQ581855.1:137763-136234;OQ721951.1:137683-136154;OQ721954.1:137656-136124;OR035652.1:137602-136073;OR035664.1:137606-136077;OR035702.1:137603-136074;OR146272.1:137622-136093;OR146330.1:137666-136137;OR264364.1:137677-136148;OR427332.1:137677-136148;OR459777.1:137656-136139;OR459797.1:137675-136146;OR943698.1:139528-137996;PP098589.2:137655-136126;PP496048.1:137659-136130;PP601206.1:139488-137926;PP776553.1:137579-136050;PP852971.1:137654-136134;PP852983.1:137671-136142;PP853000.1:137622-136099;PP853005.1:137630-136107;PP853007.1:137673-136144;PP853013.1:137645-136125;PP853027.1:137678-136155;PP853045.1:137638-136127;PP853048.1:137663-136143;PP860026.1:137677-136142;PP860027.1:137731-136196;PP860028.1:137640-136099;PP860031.1:137713-136190;PP860033.1:137714-136185;PQ153226.1:137653-136124;PQ178862.2:138321-136786;PQ221506.1:133316-131763;PQ221833.1:139491-137929;PQ340477.1:137640-136111;PQ340478.1:137622-136093;PQ340480.1:137700-136174;PQ340483.1:137686-136157;PQ340485.1:137669-136140;PQ340486.1:137687-136158;PQ340487.1:137711-136182;PQ340489.1:137686-136157;PQ340490.1:137638-136109;PQ340491.1:137693-136164;PQ340493.1:137677-136148;PQ340495.1:137703-136174;PQ340496.1:137738-136209;PQ368489.1:137669-136140;PQ451835.1:137743-136214;PQ451836.1:137681-136152;PQ451838.1:137731-136205;PQ451841.1:137694-136165;PQ451842.1:137640-136111 |
| OG0000127 | AY603973.1:138035-135945;AY741551.1:138059-135969;AY753185.1:138278-136188;DQ011154.1:138038-135948;DQ011155.1:138016-135926;EPI_ISL_13331598:136103-134013;EPI_ISL_13363142:136090-134000;EPI_ISL_13374487:136107-134017;EPI_ISL_13502582:136102-134012;EPI_ISL_13508471:136185-134095;EPI_ISL_13607904:136081-133991;EPI_ISL_13624509:136025-133935;EPI_ISL_13889442:136202-134112;EPI_ISL_13953610:136008-133918;EPI_ISL_14181948:136231-134141;EPI_ISL_14838587:137892-135802;EPI_ISL_15263355:136084-133994;EPI_ISL_15269699:136053-133963;EPI_ISL_15293002:136322-134232;EPI_ISL_15641561:136039-133949;EPI_ISL_15831211:136092-134002;EPI_ISL_15992098:136116-134026;EPI_ISL_16233784:136105-134015;EPI_ISL_16946400:136094-134004;EPI_ISL_17048206:135741-133651;EPI_ISL_17703567:136106-134016;EPI_ISL_17718458:136091-134001;EPI_ISL_18213374:136080-133990;EPI_ISL_18360394:135739-133649;EPI_ISL_18557816:136081-133991;EPI_ISL_18642365:136081-133991;EPI_ISL_18659846:136054-133964;EPI_ISL_18744049:136093-134003;EPI_ISL_19022858:136078-133988;EPI_ISL_19028778:135995-133905;EPI_ISL_19151800:136081-133991;EPI_ISL_19159116:136081-133991;EPI_ISL_19350788:136763-134673;EPI_ISL_19359677:136078-133988;EPI_ISL_19446306:136083-133993;EPI_ISL_19467692:138174-136084;JX878411.1:138883-136793;JX878425.1:138400-136310;JX878426.1:137850-135760;KC257460.1:137814-135724;KJ642612.1:136134-134044;KJ642613.1:137694-135604;KJ642616.1:137736-135646;KJ642618.1:135366-133276;KP849469.1:138208-136118;KP849471.1:138134-136044;LC831698.1:136079-133989;MN648051.1:136305-134215;MN702445.1:131551-129461;MN702446.1:137696-135606;MN702447.1:131064-128974;MN702449.1:131562-129472;MN702452.1:137737-135647;MT903338.1:136257-134167;MT903340.1:136091-134001;MT903341.1:127625-125535;MT903342.1:136109-134019;MT903347.1:138065-135975;ON568298.1:136232-134142;ON622713.1:136313-134223;ON622722.2:136084-133994;ON631963.1:136110-134020;ON649879.1:135916-133826;ON676707.1:136146-134056;ON755039.1:135738-133648;ON808413.1:136078-133988;ON880420.1:136311-134221;ON911481.2:136096-134006;ON918656.1:136118-134028;ON959133.1:136207-134117;ON959136.1:136208-134118;ON983168.1:134783-132693;OP019276.1:136078-133988;OP150924.1:136221-134131;OP160532.1:136044-133954;OP235282.1:136078-133988;OP289782.1:136081-133991;OP331335.1:136111-134021;OP382479.1:136088-133998;OP422341.1:136101-134011;OP434519.1:136077-133987;OP535324.1:136091-134001;OP535333.1:136198-134108;OP536770.1:136081-133991;OP597769.1:136115-134025;OP604566.1:136081-133991;OP612682.1:136016-133926;OP612684.1:136079-133989;OP612686.1:136069-133979;OP696841.1:133357-131267;OP879722.1:135307-133217;OQ419007.1:135310-133220;OQ419012.1:135281-133191;OQ419031.1:135285-133195;OQ419047.1:135287-133197;OQ451898.1:135168-133078;OQ451900.1:136081-133991;OQ581853.1:136107-134017;OQ581855.1:136188-134098;OQ721951.1:136108-134018;OQ721954.1:136078-133988;OR035652.1:136027-133937;OR035664.1:136031-133941;OR035702.1:136028-133938;OR146272.1:136047-133957;OR146330.1:136091-134001;OR264364.1:136102-134012;OR427332.1:136102-134012;OR459777.1:136093-134003;OR459797.1:136100-134010;OR943698.1:137950-135860;PP098589.2:136080-133990;PP496048.1:136084-133994;PP601206.1:137880-135790;PP776553.1:136004-133914;PP852971.1:136088-133998;PP852983.1:136096-134006;PP853000.1:136053-133963;PP853005.1:136061-133971;PP853007.1:136098-134008;PP853013.1:136079-133989;PP853027.1:136109-134019;PP853045.1:136081-133991;PP853048.1:136097-134007;PP860026.1:136096-134006;PP860027.1:136150-134060;PP860028.1:136053-133963;PP860031.1:136144-134054;PP860033.1:136139-134049;PQ153226.1:136078-133988;PQ178862.2:136740-134650;PQ221506.1:131717-129627;PQ221833.1:137883-135793;PQ340477.1:136065-133975;PQ340478.1:136047-133957;PQ340480.1:136128-134038;PQ340483.1:136111-134021;PQ340485.1:136094-134004;PQ340486.1:136112-134022;PQ340487.1:136136-134046;PQ340489.1:136111-134021;PQ340490.1:136063-133973;PQ340491.1:136118-134028;PQ340493.1:136102-134012;PQ340495.1:136128-134038;PQ340496.1:136163-134073;PQ368489.1:136094-134004;PQ451835.1:136168-134078;PQ451836.1:136106-134016;PQ451838.1:136159-134069;PQ451841.1:136119-134029;PQ451842.1:136065-133975 |
| OG0000128 | AY603973.1:127946-127599;AY741551.1:127968-127621;AY753185.1:128189-127842;DQ011154.1:128403-128056;DQ011155.1:128384-128037;EPI_ISL_13331598:126002-125655;EPI_ISL_13363142:125995-125648;EPI_ISL_13374487:126010-125663;EPI_ISL_13502582:125995-125648;EPI_ISL_13508471:126087-125740;EPI_ISL_13607904:125986-125639;EPI_ISL_13624509:125930-125583;EPI_ISL_13889442:126105-125758;EPI_ISL_13953610:125903-125556;EPI_ISL_14181948:126131-125784;EPI_ISL_14838587:128257-127910;EPI_ISL_15263355:125986-125639;EPI_ISL_15269699:125955-125608;EPI_ISL_15293002:126228-125881;EPI_ISL_15641561:125944-125597;EPI_ISL_15831211:125993-125646;EPI_ISL_15992098:126008-125661;EPI_ISL_16233784:126007-125660;EPI_ISL_16946400:125993-125646;EPI_ISL_17048206:125643-125296;EPI_ISL_17703567:126008-125661;EPI_ISL_17718458:125986-125639;EPI_ISL_18213374:125983-125636;EPI_ISL_18360394:125644-125297;EPI_ISL_18557816:125984-125637;EPI_ISL_18642365:125986-125639;EPI_ISL_18659846:125956-125609;EPI_ISL_18744049:125988-125641;EPI_ISL_19022858:125983-125636;EPI_ISL_19028778:125900-125553;EPI_ISL_19151800:125984-125637;EPI_ISL_19159116:125986-125639;EPI_ISL_19350788:127128-126781;EPI_ISL_19359677:125983-125636;EPI_ISL_19446306:125978-125631;EPI_ISL_19467692:128539-128192;JX878411.1:129248-128901;JX878425.1:128765-128418;JX878426.1:128215-127868;KC257460.1:128182-127835;KJ642612.1:126499-126152;KJ642613.1:128059-127712;KJ642616.1:127646-127299;KJ642618.1:125730-125383;KP849469.1:128573-128226;KP849471.1:128499-128152;LC831698.1:125984-125637;MN648051.1:126206-125859;MN702445.1:121916-121569;MN702446.1:128061-127714;MN702447.1:121429-121082;MN702449.1:121927-121580;MN702452.1:128102-127755;MT903338.1:126149-125802;MT903340.1:125986-125639;MT903341.1:117519-117172;MT903342.1:126001-125654;MT903347.1:127975-127628;ON568298.1:126132-125785;ON622713.1:126218-125871;ON622722.2:125989-125642;ON631963.1:126009-125662;ON649879.1:125819-125472;ON676707.1:126039-125692;ON755039.1:125649-125302;ON808413.1:125989-125642;ON880420.1:126216-125869;ON911481.2:126006-125659;ON918656.1:126021-125674;ON959133.1:126110-125763;ON959136.1:126111-125764;ON983168.1:124684-124337;OP019276.1:125983-125636;OP150924.1:126124-125777;OP160532.1:125949-125602;OP235282.1:125983-125636;OP289782.1:125986-125639;OP331335.1:126003-125656;OP382479.1:125990-125643;OP422341.1:126003-125656;OP434519.1:125982-125635;OP535324.1:125984-125637;OP535333.1:126093-125746;OP536770.1:125985-125638;OP597769.1:126007-125660;OP604566.1:125986-125639;OP612682.1:125911-125564;OP612684.1:125973-125626;OP612686.1:125975-125628;OP696841.1:123260-122913;OP879722.1:125209-124862;OQ419007.1:125219-124872;OQ419012.1:125186-124839;OQ419031.1:125190-124843;OQ419047.1:125192-124845;OQ451898.1:125071-124724;OQ451900.1:125984-125637;OQ581853.1:126010-125663;OQ581855.1:126091-125744;OQ721951.1:126024-125677;OQ721954.1:125981-125634;OR035652.1:125933-125586;OR035664.1:125933-125586;OR035702.1:125933-125586;OR146272.1:125949-125602;OR146330.1:125994-125647;OR264364.1:126004-125657;OR427332.1:126004-125657;OR459777.1:125988-125641;OR459797.1:126002-125655;OR943698.1:128315-127968;PP098589.2:125985-125638;PP496048.1:125989-125642;PP601206.1:128245-127898;PP776553.1:125910-125563;PP852971.1:125983-125636;PP852983.1:125989-125642;PP853000.1:125950-125603;PP853005.1:125958-125611;PP853007.1:125991-125644;PP853013.1:125964-125617;PP853027.1:125995-125648;PP853045.1:125976-125629;PP853048.1:125995-125648;PP860026.1:125993-125646;PP860027.1:126019-125672;PP860028.1:125958-125611;PP860031.1:126012-125665;PP860033.1:126018-125671;PQ153226.1:125983-125636;PQ178862.2:127105-126758;PQ221506.1:122082-121735;PQ221833.1:128248-127901;PQ340477.1:125966-125619;PQ340478.1:125951-125604;PQ340480.1:126030-125683;PQ340483.1:126014-125667;PQ340485.1:126000-125653;PQ340486.1:126015-125668;PQ340487.1:126040-125693;PQ340489.1:126015-125668;PQ340490.1:125966-125619;PQ340491.1:126021-125674;PQ340493.1:126000-125653;PQ340495.1:126030-125683;PQ340496.1:126063-125716;PQ368489.1:125986-125639;PQ451835.1:126071-125724;PQ451836.1:126003-125656;PQ451838.1:126054-125707;PQ451841.1:126014-125667;PQ451842.1:125966-125619 |
| OG0000129 | AY603973.1:127598-127365;AY741551.1:127620-127387;AY753185.1:127841-127608;DQ011154.1:128055-127822;DQ011155.1:128036-127803;EPI_ISL_13331598:125654-125421;EPI_ISL_13363142:125647-125414;EPI_ISL_13374487:125662-125429;EPI_ISL_13502582:125647-125414;EPI_ISL_13508471:125739-125506;EPI_ISL_13607904:125638-125405;EPI_ISL_13624509:125582-125349;EPI_ISL_13889442:125757-125524;EPI_ISL_13953610:125555-125322;EPI_ISL_14181948:125783-125550;EPI_ISL_14838587:127909-127676;EPI_ISL_15263355:125638-125405;EPI_ISL_15269699:125607-125374;EPI_ISL_15293002:125880-125647;EPI_ISL_15641561:125596-125363;EPI_ISL_15831211:125645-125412;EPI_ISL_15992098:125660-125427;EPI_ISL_16233784:125659-125426;EPI_ISL_16946400:125645-125412;EPI_ISL_17048206:125295-125062;EPI_ISL_17703567:125660-125427;EPI_ISL_17718458:125638-125405;EPI_ISL_18213374:125635-125402;EPI_ISL_18360394:125296-125063;EPI_ISL_18557816:125636-125403;EPI_ISL_18642365:125638-125405;EPI_ISL_18659846:125608-125375;EPI_ISL_18744049:125640-125407;EPI_ISL_19022858:125635-125402;EPI_ISL_19028778:125552-125319;EPI_ISL_19151800:125636-125403;EPI_ISL_19159116:125638-125405;EPI_ISL_19350788:126780-126547;EPI_ISL_19359677:125635-125402;EPI_ISL_19446306:125630-125397;EPI_ISL_19467692:128191-127958;JX878411.1:128900-128667;JX878425.1:128417-128184;JX878426.1:127867-127634;KC257460.1:127834-127601;KJ642612.1:126151-125918;KJ642613.1:127711-127478;KJ642616.1:127298-127065;KJ642618.1:125382-125149;KP849469.1:128225-127992;KP849471.1:128151-127918;LC831698.1:125636-125403;MN648051.1:125858-125625;MN702445.1:121568-121335;MN702446.1:127713-127480;MN702447.1:121081-120848;MN702449.1:121579-121346;MN702452.1:127754-127521;MT903338.1:125801-125568;MT903340.1:125638-125405;MT903341.1:117171-116938;MT903342.1:125653-125420;MT903347.1:127627-127394;ON568298.1:125784-125551;ON622713.1:125870-125637;ON622722.2:125641-125408;ON631963.1:125661-125428;ON649879.1:125471-125238;ON676707.1:125691-125458;ON755039.1:125301-125068;ON808413.1:125641-125408;ON880420.1:125868-125635;ON911481.2:125658-125425;ON918656.1:125673-125440;ON959133.1:125762-125529;ON959136.1:125763-125530;ON983168.1:124336-124103;OP019276.1:125635-125402;OP150924.1:125776-125543;OP160532.1:125601-125368;OP235282.1:125635-125402;OP289782.1:125638-125405;OP331335.1:125655-125422;OP382479.1:125642-125409;OP422341.1:125655-125422;OP434519.1:125634-125401;OP535324.1:125636-125403;OP535333.1:125745-125512;OP536770.1:125637-125404;OP597769.1:125659-125426;OP604566.1:125638-125405;OP612682.1:125563-125330;OP612684.1:125625-125392;OP612686.1:125627-125394;OP696841.1:122912-122679;OP879722.1:124861-124628;OQ419007.1:124871-124638;OQ419012.1:124838-124605;OQ419031.1:124842-124609;OQ419047.1:124844-124611;OQ451898.1:124723-124490;OQ451900.1:125636-125403;OQ581853.1:125662-125429;OQ581855.1:125743-125510;OQ721951.1:125676-125443;OQ721954.1:125633-125400;OR035652.1:125585-125352;OR035664.1:125585-125352;OR035702.1:125585-125352;OR146272.1:125601-125368;OR146330.1:125646-125413;OR264364.1:125656-125423;OR427332.1:125656-125423;OR459777.1:125640-125407;OR459797.1:125654-125421;OR943698.1:127967-127734;PP098589.2:125637-125404;PP496048.1:125641-125408;PP601206.1:127897-127664;PP776553.1:125562-125329;PP852971.1:125635-125402;PP852983.1:125641-125408;PP853000.1:125602-125369;PP853005.1:125610-125377;PP853007.1:125643-125410;PP853013.1:125616-125383;PP853027.1:125647-125414;PP853045.1:125628-125395;PP853048.1:125647-125414;PP860026.1:125645-125412;PP860027.1:125671-125438;PP860028.1:125610-125377;PP860031.1:125664-125431;PP860033.1:125670-125437;PQ153226.1:125635-125402;PQ178862.2:126757-126524;PQ221506.1:121734-121501;PQ221833.1:127900-127667;PQ340477.1:125618-125385;PQ340478.1:125603-125370;PQ340480.1:125682-125449;PQ340483.1:125666-125433;PQ340485.1:125652-125419;PQ340486.1:125667-125434;PQ340487.1:125692-125459;PQ340489.1:125667-125434;PQ340490.1:125618-125385;PQ340491.1:125673-125440;PQ340493.1:125652-125419;PQ340495.1:125682-125449;PQ340496.1:125715-125482;PQ368489.1:125638-125405;PQ451835.1:125723-125490;PQ451836.1:125655-125422;PQ451838.1:125706-125473;PQ451841.1:125666-125433;PQ451842.1:125618-125385 |
| OG0000130 | AY603973.1:125891-125277;AY741551.1:125913-125299;AY753185.1:126134-125520;DQ011154.1:126348-125734;DQ011155.1:126329-125715;EPI_ISL_13331598:123947-123333;EPI_ISL_13363142:123940-123326;EPI_ISL_13374487:123955-123341;EPI_ISL_13502582:123940-123326;EPI_ISL_13508471:124032-123418;EPI_ISL_13607904:123931-123317;EPI_ISL_13624509:123875-123261;EPI_ISL_13889442:124050-123436;EPI_ISL_13953610:123848-123234;EPI_ISL_14181948:124076-123462;EPI_ISL_14838587:126202-125588;EPI_ISL_15263355:123931-123317;EPI_ISL_15269699:123900-123286;EPI_ISL_15293002:124173-123559;EPI_ISL_15641561:123889-123275;EPI_ISL_15831211:123938-123324;EPI_ISL_15992098:123953-123339;EPI_ISL_16233784:123949-123335;EPI_ISL_16946400:123938-123324;EPI_ISL_17048206:123588-122974;EPI_ISL_17703567:123953-123339;EPI_ISL_17718458:123931-123317;EPI_ISL_18213374:123928-123314;EPI_ISL_18360394:123589-122975;EPI_ISL_18557816:123929-123315;EPI_ISL_18642365:123931-123317;EPI_ISL_18659846:123901-123287;EPI_ISL_18744049:123933-123319;EPI_ISL_19022858:123928-123314;EPI_ISL_19028778:123845-123231;EPI_ISL_19151800:123929-123315;EPI_ISL_19159116:123931-123317;EPI_ISL_19350788:125073-124459;EPI_ISL_19359677:123928-123314;EPI_ISL_19446306:123923-123309;EPI_ISL_19467692:126484-125870;JX878411.1:127193-126579;JX878425.1:126710-126096;JX878426.1:126160-125546;KC257460.1:126127-125513;KJ642612.1:124444-123830;KJ642613.1:126004-125390;KJ642616.1:125591-124977;KJ642618.1:123675-123061;KP849469.1:126518-125904;KP849471.1:126444-125830;LC831698.1:123929-123315;MN648051.1:124151-123537;MN702445.1:119861-119247;MN702446.1:126006-125392;MN702447.1:119374-118760;MN702449.1:119872-119258;MN702452.1:126047-125433;MT903338.1:124094-123480;MT903340.1:123931-123317;MT903341.1:115464-114850;MT903342.1:123946-123332;MT903347.1:125920-125306;ON568298.1:124077-123463;ON622713.1:124163-123549;ON622722.2:123934-123320;ON631963.1:123954-123340;ON649879.1:123764-123150;ON676707.1:123984-123370;ON755039.1:123594-122980;ON808413.1:123934-123320;ON880420.1:124161-123547;ON911481.2:123951-123337;ON918656.1:123966-123352;ON959133.1:124055-123441;ON959136.1:124056-123442;ON983168.1:122629-122015;OP019276.1:123928-123314;OP150924.1:124069-123455;OP160532.1:123894-123280;OP235282.1:123928-123314;OP289782.1:123931-123317;OP331335.1:123948-123334;OP382479.1:123935-123321;OP422341.1:123948-123334;OP434519.1:123927-123313;OP535324.1:123929-123315;OP535333.1:124038-123424;OP536770.1:123930-123316;OP597769.1:123952-123338;OP604566.1:123931-123317;OP612682.1:123856-123242;OP612684.1:123918-123304;OP612686.1:123920-123306;OP696841.1:121205-120591;OP879722.1:123154-122540;OQ419007.1:123164-122550;OQ419012.1:123131-122517;OQ419031.1:123135-122521;OQ419047.1:123137-122523;OQ451898.1:123016-122402;OQ451900.1:123929-123315;OQ581853.1:123955-123341;OQ581855.1:124036-123422;OQ721951.1:123969-123355;OQ721954.1:123926-123312;OR035652.1:123878-123264;OR035664.1:123878-123264;OR035702.1:123878-123264;OR146272.1:123894-123280;OR146330.1:123939-123325;OR264364.1:123949-123335;OR427332.1:123949-123335;OR459777.1:123933-123319;OR459797.1:123947-123333;OR943698.1:126260-125646;PP098589.2:123930-123316;PP496048.1:123934-123320;PP601206.1:126190-125576;PP776553.1:123855-123241;PP852971.1:123928-123314;PP852983.1:123934-123320;PP853000.1:123895-123281;PP853005.1:123903-123289;PP853007.1:123936-123322;PP853013.1:123909-123295;PP853027.1:123940-123326;PP853045.1:123921-123307;PP853048.1:123940-123326;PP860026.1:123938-123324;PP860027.1:123964-123350;PP860028.1:123903-123289;PP860031.1:123957-123343;PP860033.1:123963-123349;PQ153226.1:123928-123314;PQ178862.2:125050-124436;PQ221506.1:120027-119413;PQ221833.1:126193-125579;PQ340477.1:123911-123297;PQ340478.1:123896-123282;PQ340480.1:123975-123361;PQ340483.1:123959-123345;PQ340485.1:123945-123331;PQ340486.1:123960-123346;PQ340487.1:123985-123371;PQ340489.1:123960-123346;PQ340490.1:123911-123297;PQ340491.1:123966-123352;PQ340493.1:123945-123331;PQ340495.1:123975-123361;PQ340496.1:124008-123394;PQ368489.1:123931-123317;PQ451835.1:124016-123402;PQ451836.1:123948-123334;PQ451838.1:123999-123385;PQ451841.1:123959-123345;PQ451842.1:123911-123297 |
| OG0000131 | AY603973.1:124157-123873;AY741551.1:124179-123895;AY753185.1:124400-124116;DQ011154.1:124614-124330;DQ011155.1:124595-124311;EPI_ISL_13331598:122213-121929;EPI_ISL_13363142:122206-121922;EPI_ISL_13374487:122221-121937;EPI_ISL_13502582:122206-121922;EPI_ISL_13508471:122298-122014;EPI_ISL_13607904:122197-121913;EPI_ISL_13624509:122141-121857;EPI_ISL_13889442:122316-122032;EPI_ISL_13953610:122114-121830;EPI_ISL_14181948:122342-122058;EPI_ISL_14838587:124468-124184;EPI_ISL_15263355:122197-121913;EPI_ISL_15269699:122166-121882;EPI_ISL_15293002:122439-122155;EPI_ISL_15641561:122155-121871;EPI_ISL_15831211:122204-121920;EPI_ISL_15992098:122219-121935;EPI_ISL_16233784:122215-121931;EPI_ISL_16946400:122204-121920;EPI_ISL_17048206:121854-121570;EPI_ISL_17703567:122219-121935;EPI_ISL_17718458:122197-121913;EPI_ISL_18213374:122194-121910;EPI_ISL_18360394:121855-121571;EPI_ISL_18557816:122195-121911;EPI_ISL_18642365:122197-121913;EPI_ISL_18659846:122167-121883;EPI_ISL_18744049:122199-121915;EPI_ISL_19022858:122194-121910;EPI_ISL_19028778:122111-121827;EPI_ISL_19151800:122195-121911;EPI_ISL_19159116:122197-121913;EPI_ISL_19350788:123339-123055;EPI_ISL_19359677:122194-121910;EPI_ISL_19446306:122189-121905;EPI_ISL_19467692:124750-124466;JX878411.1:125459-125175;JX878425.1:124976-124692;JX878426.1:124426-124142;KC257460.1:124393-124109;KJ642612.1:122710-122426;KJ642613.1:124270-123986;KJ642616.1:123857-123573;KJ642618.1:121941-121657;KP849469.1:124784-124500;KP849471.1:124710-124426;LC831698.1:122195-121911;MN648051.1:122417-122133;MN702445.1:118127-117843;MN702446.1:124272-123988;MN702447.1:117640-117356;MN702449.1:118138-117854;MN702452.1:124313-124029;MT903338.1:122360-122076;MT903340.1:122197-121913;MT903341.1:113730-113446;MT903342.1:122212-121928;MT903347.1:124186-123902;ON568298.1:122343-122059;ON622713.1:122429-122145;ON622722.2:122200-121916;ON631963.1:122220-121936;ON649879.1:122030-121746;ON676707.1:122250-121966;ON755039.1:121860-121576;ON808413.1:122200-121916;ON880420.1:122427-122143;ON911481.2:122217-121933;ON918656.1:122232-121948;ON959133.1:122321-122037;ON959136.1:122322-122038;ON983168.1:120895-120611;OP019276.1:122194-121910;OP150924.1:122335-122051;OP160532.1:122160-121876;OP235282.1:122194-121910;OP289782.1:122197-121913;OP331335.1:122214-121930;OP382479.1:122201-121917;OP422341.1:122214-121930;OP434519.1:122193-121909;OP535324.1:122195-121911;OP535333.1:122304-122020;OP536770.1:122196-121912;OP597769.1:122218-121934;OP604566.1:122197-121913;OP612682.1:122122-121838;OP612684.1:122184-121900;OP612686.1:122186-121902;OP696841.1:119471-119187;OP879722.1:121420-121136;OQ419007.1:121430-121146;OQ419012.1:121397-121113;OQ419031.1:121401-121117;OQ419047.1:121403-121119;OQ451898.1:121282-120998;OQ451900.1:122195-121911;OQ581853.1:122221-121937;OQ581855.1:122302-122018;OQ721951.1:122235-121951;OQ721954.1:122192-121908;OR035652.1:122144-121860;OR035664.1:122144-121860;OR035702.1:122144-121860;OR146272.1:122160-121876;OR146330.1:122205-121921;OR264364.1:122215-121931;OR427332.1:122215-121931;OR459777.1:122199-121915;OR459797.1:122213-121929;OR943698.1:124526-124242;PP098589.2:122196-121912;PP496048.1:122200-121916;PP601206.1:124456-124172;PP776553.1:122121-121837;PP852971.1:122194-121910;PP852983.1:122200-121916;PP853000.1:122161-121877;PP853005.1:122169-121885;PP853007.1:122202-121918;PP853013.1:122175-121891;PP853027.1:122206-121922;PP853045.1:122187-121903;PP853048.1:122206-121922;PP860026.1:122204-121920;PP860027.1:122230-121946;PP860028.1:122169-121885;PP860031.1:122223-121939;PP860033.1:122229-121945;PQ153226.1:122194-121910;PQ178862.2:123316-123032;PQ221506.1:118293-118009;PQ221833.1:124459-124175;PQ340477.1:122177-121893;PQ340478.1:122162-121878;PQ340480.1:122241-121957;PQ340483.1:122225-121941;PQ340485.1:122211-121927;PQ340486.1:122226-121942;PQ340487.1:122251-121967;PQ340489.1:122226-121942;PQ340490.1:122177-121893;PQ340491.1:122232-121948;PQ340493.1:122211-121927;PQ340495.1:122241-121957;PQ340496.1:122274-121990;PQ368489.1:122197-121913;PQ451835.1:122282-121998;PQ451836.1:122214-121930;PQ451838.1:122265-121981;PQ451841.1:122225-121941;PQ451842.1:122177-121893 |
| OG0000132 | AY603973.1:115481-114363;AY741551.1:115485-114367;AY753185.1:115724-114606;DQ011154.1:115956-114838;DQ011155.1:115937-114819;EPI_ISL_13331598:113555-112437;EPI_ISL_13363142:113548-112430;EPI_ISL_13374487:113563-112445;EPI_ISL_13502582:113548-112430;EPI_ISL_13508471:113640-112522;EPI_ISL_13607904:113539-112421;EPI_ISL_13624509:113483-112365;EPI_ISL_13889442:113658-112540;EPI_ISL_13953610:113456-112338;EPI_ISL_14181948:113684-112566;EPI_ISL_14838587:115810-114692;EPI_ISL_15263355:113539-112421;EPI_ISL_15269699:113508-112390;EPI_ISL_15293002:113781-112663;EPI_ISL_15641561:113497-112379;EPI_ISL_15831211:113546-112428;EPI_ISL_15992098:113561-112443;EPI_ISL_16233784:113557-112439;EPI_ISL_16946400:113546-112428;EPI_ISL_17048206:113196-112078;EPI_ISL_17703567:113561-112443;EPI_ISL_17718458:113539-112421;EPI_ISL_18213374:113536-112418;EPI_ISL_18360394:113197-112079;EPI_ISL_18557816:113537-112419;EPI_ISL_18642365:113539-112421;EPI_ISL_18659846:113509-112391;EPI_ISL_18744049:113541-112423;EPI_ISL_19022858:113536-112418;EPI_ISL_19028778:113453-112335;EPI_ISL_19151800:113537-112419;EPI_ISL_19159116:113539-112421;EPI_ISL_19350788:114681-113563;EPI_ISL_19359677:113536-112418;EPI_ISL_19446306:113531-112413;EPI_ISL_19467692:116092-114974;JX878411.1:116801-115683;JX878425.1:116318-115200;JX878426.1:115768-114650;KC257460.1:115735-114617;KJ642612.1:114052-112934;KJ642613.1:115612-114494;KJ642616.1:115175-114057;KJ642618.1:113283-112165;KP849469.1:116126-115008;KP849471.1:116052-114934;LC831698.1:113537-112419;MN648051.1:113759-112641;MN702445.1:109469-108351;MN702446.1:115614-114496;MN702447.1:108982-107864;MN702449.1:109480-108362;MN702452.1:115655-114537;MT903338.1:113702-112584;MT903340.1:113539-112421;MT903341.1:105072-103954;MT903342.1:113554-112436;MT903347.1:115492-114374;ON568298.1:113685-112567;ON622713.1:113771-112653;ON622722.2:113542-112424;ON631963.1:113562-112444;ON649879.1:113372-112254;ON676707.1:113592-112474;ON755039.1:113202-112084;ON808413.1:113542-112424;ON880420.1:113769-112651;ON911481.2:113559-112441;ON918656.1:113574-112456;ON959133.1:113663-112545;ON959136.1:113664-112546;ON983168.1:112237-111119;OP019276.1:113536-112418;OP150924.1:113677-112559;OP160532.1:113502-112384;OP235282.1:113536-112418;OP289782.1:113539-112421;OP331335.1:113556-112438;OP382479.1:113543-112425;OP422341.1:113556-112438;OP434519.1:113535-112417;OP535324.1:113537-112419;OP535333.1:113646-112528;OP536770.1:113538-112420;OP597769.1:113560-112442;OP604566.1:113539-112421;OP612682.1:113464-112346;OP612684.1:113527-112409;OP612686.1:113528-112410;OP696841.1:110813-109695;OP879722.1:112762-111644;OQ419007.1:112772-111654;OQ419012.1:112739-111621;OQ419031.1:112743-111625;OQ419047.1:112745-111627;OQ451898.1:112624-111506;OQ451900.1:113537-112419;OQ581853.1:113563-112445;OQ581855.1:113644-112526;OQ721951.1:113577-112459;OQ721954.1:113534-112416;OR035652.1:113486-112368;OR035664.1:113486-112368;OR035702.1:113486-112368;OR146272.1:113502-112384;OR146330.1:113547-112429;OR264364.1:113557-112439;OR427332.1:113557-112439;OR459777.1:113541-112423;OR459797.1:113556-112438;OR943698.1:115868-114750;PP098589.2:113538-112420;PP496048.1:113542-112424;PP601206.1:115798-114680;PP776553.1:113463-112345;PP852971.1:113536-112418;PP852983.1:113542-112424;PP853000.1:113503-112385;PP853005.1:113511-112393;PP853007.1:113544-112426;PP853013.1:113517-112399;PP853027.1:113548-112430;PP853045.1:113529-112411;PP853048.1:113548-112430;PP860026.1:113546-112428;PP860027.1:113572-112454;PP860028.1:113511-112393;PP860031.1:113565-112447;PP860033.1:113571-112453;PQ153226.1:113536-112418;PQ178862.2:114658-113540;PQ221506.1:109635-108517;PQ221833.1:115801-114683;PQ340477.1:113519-112401;PQ340478.1:113504-112386;PQ340480.1:113583-112465;PQ340483.1:113567-112449;PQ340485.1:113553-112435;PQ340486.1:113568-112450;PQ340487.1:113593-112475;PQ340489.1:113568-112450;PQ340490.1:113519-112401;PQ340491.1:113574-112456;PQ340493.1:113553-112435;PQ340495.1:113583-112465;PQ340496.1:113616-112498;PQ368489.1:113539-112421;PQ451835.1:113624-112506;PQ451836.1:113556-112438;PQ451838.1:113607-112489;PQ451841.1:113567-112449;PQ451842.1:113519-112401 |
| OG0000133 | AY603973.1:113843-112998;AY741551.1:113847-113002;AY753185.1:114086-113241;DQ011154.1:114318-113473;DQ011155.1:114299-113454;EPI_ISL_13331598:111917-111072;EPI_ISL_13363142:111910-111065;EPI_ISL_13374487:111925-111080;EPI_ISL_13502582:111910-111065;EPI_ISL_13508471:112002-111157;EPI_ISL_13607904:111901-111056;EPI_ISL_13624509:111845-111000;EPI_ISL_13889442:112020-111175;EPI_ISL_13953610:111818-110973;EPI_ISL_14181948:112046-111201;EPI_ISL_14838587:114172-113327;EPI_ISL_15263355:111901-111056;EPI_ISL_15269699:111870-111025;EPI_ISL_15293002:112143-111298;EPI_ISL_15641561:111859-111014;EPI_ISL_15831211:111908-111063;EPI_ISL_15992098:111923-111078;EPI_ISL_16233784:111919-111074;EPI_ISL_16946400:111908-111063;EPI_ISL_17048206:111558-110713;EPI_ISL_17703567:111923-111078;EPI_ISL_17718458:111901-111056;EPI_ISL_18213374:111898-111053;EPI_ISL_18360394:111559-110714;EPI_ISL_18557816:111899-111054;EPI_ISL_18642365:111901-111056;EPI_ISL_18659846:111871-111026;EPI_ISL_18744049:111903-111058;EPI_ISL_19022858:111898-111053;EPI_ISL_19028778:111815-110970;EPI_ISL_19151800:111899-111054;EPI_ISL_19159116:111901-111056;EPI_ISL_19350788:113043-112198;EPI_ISL_19359677:111898-111053;EPI_ISL_19446306:111893-111048;EPI_ISL_19467692:114454-113609;JX878411.1:115163-114318;JX878425.1:114680-113835;JX878426.1:114130-113285;KC257460.1:114097-113252;KJ642612.1:112414-111569;KJ642613.1:113974-113129;KJ642616.1:113537-112692;KJ642618.1:111645-110800;KP849469.1:114488-113643;KP849471.1:114414-113569;LC831698.1:111899-111054;MN648051.1:112121-111276;MN702445.1:107831-106986;MN702446.1:113976-113131;MN702447.1:107344-106499;MN702449.1:107842-106997;MN702452.1:114017-113172;MT903338.1:112064-111219;MT903340.1:111901-111056;MT903341.1:103434-102589;MT903342.1:111916-111071;MT903347.1:113854-113009;ON568298.1:112047-111202;ON622713.1:112133-111288;ON622722.2:111904-111059;ON631963.1:111924-111079;ON649879.1:111734-110889;ON676707.1:111954-111109;ON755039.1:111564-110719;ON808413.1:111904-111059;ON880420.1:112131-111286;ON911481.2:111921-111076;ON918656.1:111936-111091;ON959133.1:112025-111180;ON959136.1:112026-111181;ON983168.1:110599-109754;OP019276.1:111898-111053;OP150924.1:112039-111194;OP160532.1:111864-111019;OP235282.1:111898-111053;OP289782.1:111901-111056;OP331335.1:111918-111073;OP382479.1:111905-111060;OP422341.1:111918-111073;OP434519.1:111897-111052;OP535324.1:111899-111054;OP535333.1:112008-111163;OP536770.1:111900-111055;OP597769.1:111922-111077;OP604566.1:111901-111056;OP612682.1:111826-110981;OP612684.1:111889-111044;OP612686.1:111890-111045;OP696841.1:109175-108330;OP879722.1:111124-110279;OQ419007.1:111134-110289;OQ419012.1:111101-110256;OQ419031.1:111105-110260;OQ419047.1:111107-110262;OQ451898.1:110986-110141;OQ451900.1:111899-111054;OQ581853.1:111925-111080;OQ581855.1:112006-111161;OQ721951.1:111939-111094;OQ721954.1:111896-111051;OR035652.1:111848-111003;OR035664.1:111848-111003;OR035702.1:111848-111003;OR146272.1:111864-111019;OR146330.1:111909-111064;OR264364.1:111919-111074;OR427332.1:111919-111074;OR459777.1:111903-111058;OR459797.1:111918-111073;OR943698.1:114230-113385;PP098589.2:111900-111055;PP496048.1:111904-111059;PP601206.1:114160-113315;PP776553.1:111825-110980;PP852971.1:111898-111053;PP852983.1:111904-111059;PP853000.1:111865-111020;PP853005.1:111873-111028;PP853007.1:111906-111061;PP853013.1:111879-111034;PP853027.1:111910-111065;PP853045.1:111891-111046;PP853048.1:111910-111065;PP860026.1:111908-111063;PP860027.1:111934-111089;PP860028.1:111873-111028;PP860031.1:111927-111082;PP860033.1:111933-111088;PQ153226.1:111898-111053;PQ178862.2:113020-112175;PQ221506.1:107997-107152;PQ221833.1:114163-113318;PQ340477.1:111881-111036;PQ340478.1:111866-111021;PQ340480.1:111945-111100;PQ340483.1:111929-111084;PQ340485.1:111915-111070;PQ340486.1:111930-111085;PQ340487.1:111955-111110;PQ340489.1:111930-111085;PQ340490.1:111881-111036;PQ340491.1:111936-111091;PQ340493.1:111915-111070;PQ340495.1:111945-111100;PQ340496.1:111978-111133;PQ368489.1:111901-111056;PQ451835.1:111986-111141;PQ451836.1:111918-111073;PQ451838.1:111969-111124;PQ451841.1:111929-111084;PQ451842.1:111881-111036 |
| OG0000134 | AY603973.1:110996-110763;AY741551.1:111000-110767;AY753185.1:111239-111006;DQ011154.1:111471-111238;DQ011155.1:111452-111219;EPI_ISL_13331598:109070-108837;EPI_ISL_13363142:109063-108830;EPI_ISL_13374487:109078-108845;EPI_ISL_13502582:109063-108830;EPI_ISL_13508471:109155-108922;EPI_ISL_13607904:109054-108821;EPI_ISL_13624509:108998-108765;EPI_ISL_13889442:109173-108940;EPI_ISL_13953610:108971-108738;EPI_ISL_14181948:109199-108966;EPI_ISL_14838587:111325-111092;EPI_ISL_15263355:109054-108821;EPI_ISL_15269699:109023-108790;EPI_ISL_15293002:109296-109063;EPI_ISL_15641561:109012-108779;EPI_ISL_15831211:109061-108828;EPI_ISL_15992098:109076-108843;EPI_ISL_16233784:109072-108839;EPI_ISL_16946400:109061-108828;EPI_ISL_17048206:108711-108478;EPI_ISL_17703567:109076-108843;EPI_ISL_17718458:109054-108821;EPI_ISL_18213374:109051-108818;EPI_ISL_18360394:108712-108479;EPI_ISL_18557816:109052-108819;EPI_ISL_18642365:109054-108821;EPI_ISL_18659846:109024-108791;EPI_ISL_18744049:109056-108823;EPI_ISL_19022858:109051-108818;EPI_ISL_19028778:108968-108735;EPI_ISL_19151800:109052-108819;EPI_ISL_19159116:109054-108821;EPI_ISL_19350788:110196-109963;EPI_ISL_19359677:109051-108818;EPI_ISL_19446306:109046-108813;EPI_ISL_19467692:111607-111374;JX878411.1:112316-112083;JX878425.1:111833-111600;JX878426.1:111283-111050;KC257460.1:111250-111017;KJ642612.1:109567-109334;KJ642613.1:111127-110894;KJ642616.1:110690-110457;KJ642618.1:108798-108565;KP849469.1:111641-111408;KP849471.1:111567-111334;LC831698.1:109052-108819;MN648051.1:109274-109041;MN702445.1:104984-104751;MN702446.1:111129-110896;MN702447.1:104497-104264;MN702449.1:104995-104762;MN702452.1:111170-110937;MT903338.1:109217-108984;MT903340.1:109054-108821;MT903341.1:100587-100354;MT903342.1:109069-108836;MT903347.1:111007-110774;ON568298.1:109200-108967;ON622713.1:109286-109053;ON622722.2:109057-108824;ON631963.1:109077-108844;ON649879.1:108887-108654;ON676707.1:109107-108874;ON755039.1:108717-108484;ON808413.1:109057-108824;ON880420.1:109284-109051;ON911481.2:109074-108841;ON918656.1:109089-108856;ON959133.1:109178-108945;ON959136.1:109179-108946;ON983168.1:107752-107519;OP019276.1:109051-108818;OP150924.1:109192-108959;OP160532.1:109017-108784;OP235282.1:109051-108818;OP289782.1:109054-108821;OP331335.1:109071-108838;OP382479.1:109058-108825;OP422341.1:109071-108838;OP434519.1:109050-108817;OP535324.1:109052-108819;OP535333.1:109161-108928;OP536770.1:109053-108820;OP597769.1:109075-108842;OP604566.1:109054-108821;OP612682.1:108979-108746;OP612684.1:109042-108809;OP612686.1:109043-108810;OP696841.1:106328-106095;OP879722.1:108277-108044;OQ419007.1:108287-108054;OQ419012.1:108254-108021;OQ419031.1:108258-108025;OQ419047.1:108260-108027;OQ451898.1:108139-107906;OQ451900.1:109052-108819;OQ581853.1:109078-108845;OQ581855.1:109159-108926;OQ721951.1:109092-108859;OQ721954.1:109049-108816;OR035652.1:109001-108768;OR035664.1:109001-108768;OR035702.1:109001-108768;OR146272.1:109017-108784;OR146330.1:109062-108829;OR264364.1:109072-108839;OR427332.1:109072-108839;OR459777.1:109056-108823;OR459797.1:109071-108838;OR943698.1:111383-111150;PP098589.2:109053-108820;PP496048.1:109057-108824;PP601206.1:111313-111080;PP776553.1:108978-108745;PP852971.1:109051-108818;PP852983.1:109057-108824;PP853000.1:109018-108785;PP853005.1:109026-108793;PP853007.1:109059-108826;PP853013.1:109032-108799;PP853027.1:109063-108830;PP853045.1:109044-108811;PP853048.1:109063-108830;PP860026.1:109061-108828;PP860027.1:109087-108854;PP860028.1:109026-108793;PP860031.1:109080-108847;PP860033.1:109086-108853;PQ153226.1:109051-108818;PQ178862.2:110173-109940;PQ221506.1:105150-104917;PQ221833.1:111316-111083;PQ340477.1:109034-108801;PQ340478.1:109019-108786;PQ340480.1:109098-108865;PQ340483.1:109082-108849;PQ340485.1:109068-108835;PQ340486.1:109083-108850;PQ340487.1:109108-108875;PQ340489.1:109083-108850;PQ340490.1:109034-108801;PQ340491.1:109089-108856;PQ340493.1:109068-108835;PQ340495.1:109098-108865;PQ340496.1:109131-108898;PQ368489.1:109054-108821;PQ451835.1:109139-108906;PQ451836.1:109071-108838;PQ451838.1:109122-108889;PQ451841.1:109082-108849;PQ451842.1:109034-108801 |
| OG0000135 | AY603973.1:109595-107940;AY741551.1:109599-107944;AY753185.1:109838-108183;DQ011154.1:110069-108414;DQ011155.1:110050-108395;EPI_ISL_13331598:107669-106014;EPI_ISL_13363142:107662-106007;EPI_ISL_13374487:107677-106022;EPI_ISL_13502582:107662-106007;EPI_ISL_13508471:107754-106099;EPI_ISL_13607904:107653-105998;EPI_ISL_13624509:107597-105942;EPI_ISL_13889442:107772-106117;EPI_ISL_13953610:107570-105915;EPI_ISL_14181948:107798-106143;EPI_ISL_14838587:109923-108268;EPI_ISL_15263355:107653-105998;EPI_ISL_15269699:107622-105967;EPI_ISL_15293002:107895-106240;EPI_ISL_15641561:107611-105956;EPI_ISL_15831211:107660-106005;EPI_ISL_15992098:107675-106020;EPI_ISL_16233784:107671-106016;EPI_ISL_16946400:107660-106005;EPI_ISL_17048206:107310-105655;EPI_ISL_17703567:107675-106020;EPI_ISL_17718458:107653-105998;EPI_ISL_18213374:107650-105995;EPI_ISL_18360394:107311-105656;EPI_ISL_18557816:107651-105996;EPI_ISL_18642365:107653-105998;EPI_ISL_18659846:107623-105968;EPI_ISL_18744049:107655-106000;EPI_ISL_19022858:107650-105995;EPI_ISL_19028778:107567-105912;EPI_ISL_19151800:107651-105996;EPI_ISL_19159116:107653-105998;EPI_ISL_19350788:108795-107140;EPI_ISL_19359677:107650-105995;EPI_ISL_19446306:107645-105990;EPI_ISL_19467692:110206-108551;JX878411.1:110914-109259;JX878425.1:110431-108776;JX878426.1:109880-108225;KC257460.1:109848-108193;KJ642612.1:108166-106511;KJ642613.1:109725-108070;KJ642616.1:109289-107634;KJ642618.1:107396-105741;KP849469.1:110239-108584;KP849471.1:110165-108510;LC831698.1:107651-105996;MN648051.1:107873-106218;MN702445.1:103582-101927;MN702446.1:109727-108072;MN702447.1:103095-101440;MN702449.1:103593-101938;MN702452.1:109768-108113;MT903338.1:107816-106161;MT903340.1:107653-105998;MT903341.1:99186-97531;MT903342.1:107668-106013;MT903347.1:109606-107951;ON568298.1:107799-106144;ON622713.1:107885-106230;ON622722.2:107656-106001;ON631963.1:107676-106021;ON649879.1:107486-105831;ON676707.1:107706-106051;ON755039.1:107316-105661;ON808413.1:107656-106001;ON880420.1:107883-106228;ON911481.2:107673-106018;ON918656.1:107688-106033;ON959133.1:107777-106122;ON959136.1:107778-106123;ON983168.1:106351-104696;OP019276.1:107650-105995;OP150924.1:107791-106136;OP160532.1:107616-105961;OP235282.1:107650-105995;OP289782.1:107653-105998;OP331335.1:107670-106015;OP382479.1:107657-106002;OP422341.1:107670-106015;OP434519.1:107649-105994;OP535324.1:107651-105996;OP535333.1:107760-106105;OP536770.1:107652-105997;OP597769.1:107674-106019;OP604566.1:107653-105998;OP612682.1:107578-105923;OP612684.1:107641-105986;OP612686.1:107642-105987;OP696841.1:104927-103272;OP879722.1:106876-105221;OQ419007.1:106886-105231;OQ419012.1:106853-105198;OQ419031.1:106857-105202;OQ419047.1:106859-105204;OQ451898.1:106738-105083;OQ451900.1:107651-105996;OQ581853.1:107677-106022;OQ581855.1:107758-106103;OQ721951.1:107691-106036;OQ721954.1:107648-105993;OR035652.1:107600-105945;OR035664.1:107600-105945;OR035702.1:107600-105945;OR146272.1:107616-105961;OR146330.1:107661-106006;OR264364.1:107671-106016;OR427332.1:107671-106016;OR459777.1:107655-106000;OR459797.1:107670-106015;OR943698.1:109981-108326;PP098589.2:107652-105997;PP496048.1:107656-106001;PP601206.1:109912-108257;PP776553.1:107577-105922;PP852971.1:107650-105995;PP852983.1:107656-106001;PP853000.1:107617-105962;PP853005.1:107625-105970;PP853007.1:107658-106003;PP853013.1:107631-105976;PP853027.1:107662-106007;PP853045.1:107643-105988;PP853048.1:107662-106007;PP860026.1:107660-106005;PP860027.1:107685-106030;PP860028.1:107625-105970;PP860031.1:107679-106024;PP860033.1:107684-106029;PQ153226.1:107650-105995;PQ178862.2:108772-107117;PQ221506.1:103748-102093;PQ221833.1:109914-108259;PQ340477.1:107633-105978;PQ340478.1:107618-105963;PQ340480.1:107697-106042;PQ340483.1:107681-106026;PQ340485.1:107667-106012;PQ340486.1:107682-106027;PQ340487.1:107707-106052;PQ340489.1:107682-106027;PQ340490.1:107633-105978;PQ340491.1:107688-106033;PQ340493.1:107667-106012;PQ340495.1:107697-106042;PQ340496.1:107730-106075;PQ368489.1:107653-105998;PQ451835.1:107738-106083;PQ451836.1:107670-106015;PQ451838.1:107721-106066;PQ451841.1:107681-106026;PQ451842.1:107633-105978 |
| OG0000136 | AY603973.1:107909-107046;AY741551.1:107913-107050;AY753185.1:108152-107289;DQ011154.1:108383-107520;DQ011155.1:108364-107501;EPI_ISL_13331598:105983-105120;EPI_ISL_13363142:105976-105113;EPI_ISL_13374487:105991-105128;EPI_ISL_13502582:105976-105113;EPI_ISL_13508471:106068-105205;EPI_ISL_13607904:105967-105104;EPI_ISL_13624509:105911-105048;EPI_ISL_13889442:106086-105223;EPI_ISL_13953610:105884-105021;EPI_ISL_14181948:106112-105249;EPI_ISL_14838587:108237-107374;EPI_ISL_15263355:105967-105104;EPI_ISL_15269699:105936-105073;EPI_ISL_15293002:106209-105346;EPI_ISL_15641561:105925-105062;EPI_ISL_15831211:105974-105111;EPI_ISL_15992098:105989-105126;EPI_ISL_16233784:105985-105122;EPI_ISL_16946400:105974-105111;EPI_ISL_17048206:105624-104761;EPI_ISL_17703567:105989-105126;EPI_ISL_17718458:105967-105104;EPI_ISL_18213374:105964-105101;EPI_ISL_18360394:105625-104762;EPI_ISL_18557816:105965-105102;EPI_ISL_18642365:105967-105104;EPI_ISL_18659846:105937-105074;EPI_ISL_18744049:105969-105106;EPI_ISL_19022858:105964-105101;EPI_ISL_19028778:105881-105018;EPI_ISL_19151800:105965-105102;EPI_ISL_19159116:105967-105104;EPI_ISL_19350788:107109-106246;EPI_ISL_19359677:105964-105101;EPI_ISL_19446306:105959-105096;EPI_ISL_19467692:108520-107657;JX878411.1:109228-108365;JX878425.1:108745-107882;JX878426.1:108194-107331;KC257460.1:108162-107299;KJ642612.1:106480-105617;KJ642613.1:108039-107176;KJ642616.1:107603-106740;KJ642618.1:105710-104847;KP849469.1:108553-107690;KP849471.1:108479-107616;LC831698.1:105965-105102;MN648051.1:106187-105324;MN702445.1:101896-101033;MN702446.1:108041-107178;MN702447.1:101409-100546;MN702449.1:101907-101044;MN702452.1:108082-107219;MT903338.1:106130-105267;MT903340.1:105967-105104;MT903341.1:97500-96637;MT903342.1:105982-105119;MT903347.1:107920-107057;ON568298.1:106113-105250;ON622713.1:106199-105336;ON622722.2:105970-105107;ON631963.1:105990-105127;ON649879.1:105800-104937;ON676707.1:106020-105157;ON755039.1:105630-104767;ON808413.1:105970-105107;ON880420.1:106197-105334;ON911481.2:105987-105124;ON918656.1:106002-105139;ON959133.1:106091-105228;ON959136.1:106092-105229;ON983168.1:104665-103802;OP019276.1:105964-105101;OP150924.1:106105-105242;OP160532.1:105930-105067;OP235282.1:105964-105101;OP289782.1:105967-105104;OP331335.1:105984-105121;OP382479.1:105971-105108;OP422341.1:105984-105121;OP434519.1:105963-105100;OP535324.1:105965-105102;OP535333.1:106074-105211;OP536770.1:105966-105103;OP597769.1:105988-105125;OP604566.1:105967-105104;OP612682.1:105892-105029;OP612684.1:105955-105092;OP612686.1:105956-105093;OP696841.1:103241-102378;OP879722.1:105190-104327;OQ419007.1:105200-104337;OQ419012.1:105167-104304;OQ419031.1:105171-104308;OQ419047.1:105173-104310;OQ451898.1:105052-104189;OQ451900.1:105965-105102;OQ581853.1:105991-105128;OQ581855.1:106072-105209;OQ721951.1:106005-105142;OQ721954.1:105962-105099;OR035652.1:105914-105051;OR035664.1:105914-105051;OR035702.1:105914-105051;OR146272.1:105930-105067;OR146330.1:105975-105112;OR264364.1:105985-105122;OR427332.1:105985-105122;OR459777.1:105969-105106;OR459797.1:105984-105121;OR943698.1:108295-107432;PP098589.2:105966-105103;PP496048.1:105970-105107;PP601206.1:108226-107363;PP776553.1:105891-105028;PP852971.1:105964-105101;PP852983.1:105970-105107;PP853000.1:105931-105068;PP853005.1:105939-105076;PP853007.1:105972-105109;PP853013.1:105945-105082;PP853027.1:105976-105113;PP853045.1:105957-105094;PP853048.1:105976-105113;PP860026.1:105974-105111;PP860027.1:105999-105136;PP860028.1:105939-105076;PP860031.1:105993-105130;PP860033.1:105998-105135;PQ153226.1:105964-105101;PQ178862.2:107086-106223;PQ221506.1:102062-101199;PQ221833.1:108228-107365;PQ340477.1:105947-105084;PQ340478.1:105932-105069;PQ340480.1:106011-105148;PQ340483.1:105995-105132;PQ340485.1:105981-105118;PQ340486.1:105996-105133;PQ340487.1:106021-105158;PQ340489.1:105996-105133;PQ340490.1:105947-105084;PQ340491.1:106002-105139;PQ340493.1:105981-105118;PQ340495.1:106011-105148;PQ340496.1:106044-105181;PQ368489.1:105967-105104;PQ451835.1:106052-105189;PQ451836.1:105984-105121;PQ451838.1:106035-105172;PQ451841.1:105995-105132;PQ451842.1:105947-105084 |
| OG0000137 | AY603973.1:100358-100083;AY741551.1:100362-100087;AY753185.1:100601-100326;DQ011154.1:100832-100557;DQ011155.1:100813-100538;EPI_ISL_13331598:98432-98157;EPI_ISL_13363142:98425-98150;EPI_ISL_13374487:98440-98165;EPI_ISL_13502582:98425-98150;EPI_ISL_13508471:98517-98242;EPI_ISL_13607904:98416-98141;EPI_ISL_13624509:98360-98085;EPI_ISL_13889442:98535-98260;EPI_ISL_13953610:98333-98058;EPI_ISL_14181948:98561-98286;EPI_ISL_14838587:100686-100411;EPI_ISL_15263355:98416-98141;EPI_ISL_15269699:98385-98110;EPI_ISL_15293002:98658-98383;EPI_ISL_15641561:98374-98099;EPI_ISL_15831211:98423-98148;EPI_ISL_15992098:98438-98163;EPI_ISL_16233784:98434-98159;EPI_ISL_16946400:98423-98148;EPI_ISL_17048206:98073-97798;EPI_ISL_17703567:98438-98163;EPI_ISL_17718458:98416-98141;EPI_ISL_18213374:98413-98138;EPI_ISL_18360394:98074-97799;EPI_ISL_18557816:98414-98139;EPI_ISL_18642365:98416-98141;EPI_ISL_18659846:98386-98111;EPI_ISL_18744049:98418-98143;EPI_ISL_19022858:98413-98138;EPI_ISL_19028778:98330-98055;EPI_ISL_19151800:98414-98139;EPI_ISL_19159116:98416-98141;EPI_ISL_19350788:99558-99283;EPI_ISL_19359677:98413-98138;EPI_ISL_19446306:98408-98133;EPI_ISL_19467692:100969-100694;JX878411.1:101677-101402;JX878425.1:101194-100919;JX878426.1:100643-100368;KC257460.1:100611-100336;KJ642612.1:98929-98654;KJ642613.1:100488-100213;KJ642616.1:100052-99777;KJ642618.1:98159-97884;KP849469.1:101002-100727;KP849471.1:100928-100653;LC831698.1:98414-98139;MN648051.1:98636-98361;MN702445.1:94345-94070;MN702446.1:100490-100215;MN702447.1:93858-93583;MN702449.1:94356-94081;MN702452.1:100531-100256;MT903338.1:98579-98304;MT903340.1:98416-98141;MT903341.1:89949-89674;MT903342.1:98431-98156;MT903347.1:100369-100094;ON568298.1:98562-98287;ON622713.1:98648-98373;ON622722.2:98419-98144;ON631963.1:98439-98164;ON649879.1:98249-97974;ON676707.1:98469-98194;ON755039.1:98079-97804;ON808413.1:98419-98144;ON880420.1:98646-98371;ON911481.2:98436-98161;ON918656.1:98451-98176;ON959133.1:98540-98265;ON959136.1:98541-98266;ON983168.1:97114-96839;OP019276.1:98413-98138;OP150924.1:98554-98279;OP160532.1:98379-98104;OP235282.1:98413-98138;OP289782.1:98416-98141;OP331335.1:98433-98158;OP382479.1:98420-98145;OP422341.1:98433-98158;OP434519.1:98412-98137;OP535324.1:98414-98139;OP535333.1:98523-98248;OP536770.1:98415-98140;OP597769.1:98437-98162;OP604566.1:98416-98141;OP612682.1:98341-98066;OP612684.1:98404-98129;OP612686.1:98405-98130;OP696841.1:95690-95415;OP879722.1:97639-97364;OQ419007.1:97649-97374;OQ419012.1:97616-97341;OQ419031.1:97620-97345;OQ419047.1:97622-97347;OQ451898.1:97501-97226;OQ451900.1:98414-98139;OQ581853.1:98440-98165;OQ581855.1:98521-98246;OQ721951.1:98454-98179;OQ721954.1:98411-98136;OR035652.1:98363-98088;OR035664.1:98363-98088;OR035702.1:98363-98088;OR146272.1:98379-98104;OR146330.1:98424-98149;OR264364.1:98434-98159;OR427332.1:98434-98159;OR459777.1:98418-98143;OR459797.1:98433-98158;OR943698.1:100744-100469;PP098589.2:98415-98140;PP496048.1:98419-98144;PP601206.1:100675-100400;PP776553.1:98340-98065;PP852971.1:98413-98138;PP852983.1:98419-98144;PP853000.1:98380-98105;PP853005.1:98388-98113;PP853007.1:98421-98146;PP853013.1:98394-98119;PP853027.1:98425-98150;PP853045.1:98406-98131;PP853048.1:98425-98150;PP860026.1:98423-98148;PP860027.1:98448-98173;PP860028.1:98388-98113;PP860031.1:98442-98167;PP860033.1:98447-98172;PQ153226.1:98413-98138;PQ178862.2:99535-99260;PQ221506.1:94511-94236;PQ221833.1:100677-100402;PQ340477.1:98396-98121;PQ340478.1:98381-98106;PQ340480.1:98460-98185;PQ340483.1:98444-98169;PQ340485.1:98430-98155;PQ340486.1:98445-98170;PQ340487.1:98470-98195;PQ340489.1:98445-98170;PQ340490.1:98396-98121;PQ340491.1:98451-98176;PQ340493.1:98430-98155;PQ340495.1:98460-98185;PQ340496.1:98493-98218;PQ368489.1:98416-98141;PQ451835.1:98501-98226;PQ451836.1:98433-98158;PQ451838.1:98484-98209;PQ451841.1:98444-98169;PQ451842.1:98396-98121 |
| OG0000138 | AY603973.1:98195-97986;AY741551.1:98199-97990;AY753185.1:98438-98229;DQ011154.1:98669-98460;DQ011155.1:98650-98441;EPI_ISL_13331598:96269-96060;EPI_ISL_13363142:96262-96053;EPI_ISL_13374487:96277-96068;EPI_ISL_13502582:96262-96053;EPI_ISL_13508471:96354-96145;EPI_ISL_13607904:96253-96044;EPI_ISL_13624509:96197-95988;EPI_ISL_13889442:96372-96163;EPI_ISL_13953610:96170-95961;EPI_ISL_14181948:96398-96189;EPI_ISL_14838587:98523-98314;EPI_ISL_15263355:96253-96044;EPI_ISL_15269699:96222-96013;EPI_ISL_15293002:96495-96286;EPI_ISL_15641561:96211-96002;EPI_ISL_15831211:96260-96051;EPI_ISL_15992098:96275-96066;EPI_ISL_16233784:96271-96062;EPI_ISL_16946400:96260-96051;EPI_ISL_17048206:95910-95701;EPI_ISL_17703567:96275-96066;EPI_ISL_17718458:96253-96044;EPI_ISL_18213374:96250-96041;EPI_ISL_18360394:95911-95702;EPI_ISL_18557816:96251-96042;EPI_ISL_18642365:96253-96044;EPI_ISL_18659846:96223-96014;EPI_ISL_18744049:96255-96046;EPI_ISL_19022858:96250-96041;EPI_ISL_19028778:96167-95958;EPI_ISL_19151800:96251-96042;EPI_ISL_19159116:96253-96044;EPI_ISL_19350788:97395-97186;EPI_ISL_19359677:96250-96041;EPI_ISL_19446306:96245-96036;EPI_ISL_19467692:98806-98597;JX878411.1:99514-99305;JX878425.1:99031-98822;JX878426.1:98480-98271;KC257460.1:98448-98239;KJ642612.1:96766-96557;KJ642613.1:98325-98116;KJ642616.1:97889-97680;KJ642618.1:95996-95787;KP849469.1:98839-98630;KP849471.1:98765-98556;LC831698.1:96251-96042;MN648051.1:96473-96264;MN702445.1:92182-91973;MN702446.1:98327-98118;MN702447.1:91695-91486;MN702449.1:92193-91984;MN702452.1:98368-98159;MT903338.1:96416-96207;MT903340.1:96253-96044;MT903341.1:87786-87577;MT903342.1:96268-96059;MT903347.1:98206-97997;ON568298.1:96399-96190;ON622713.1:96485-96276;ON622722.2:96256-96047;ON631963.1:96276-96067;ON649879.1:96086-95877;ON676707.1:96306-96097;ON755039.1:95916-95707;ON808413.1:96256-96047;ON880420.1:96483-96274;ON911481.2:96273-96064;ON918656.1:96288-96079;ON959133.1:96377-96168;ON959136.1:96378-96169;ON983168.1:94951-94742;OP019276.1:96250-96041;OP150924.1:96391-96182;OP160532.1:96216-96007;OP235282.1:96250-96041;OP289782.1:96253-96044;OP331335.1:96270-96061;OP382479.1:96257-96048;OP422341.1:96270-96061;OP434519.1:96249-96040;OP535324.1:96251-96042;OP535333.1:96360-96151;OP536770.1:96252-96043;OP597769.1:96274-96065;OP604566.1:96253-96044;OP612682.1:96178-95969;OP612684.1:96241-96032;OP612686.1:96242-96033;OP696841.1:93527-93318;OP879722.1:95476-95267;OQ419007.1:95486-95277;OQ419012.1:95453-95244;OQ419031.1:95457-95248;OQ419047.1:95459-95250;OQ451898.1:95338-95129;OQ451900.1:96251-96042;OQ581853.1:96277-96068;OQ581855.1:96358-96149;OQ721951.1:96291-96082;OQ721954.1:96248-96039;OR035652.1:96200-95991;OR035664.1:96200-95991;OR035702.1:96200-95991;OR146272.1:96216-96007;OR146330.1:96261-96052;OR264364.1:96271-96062;OR427332.1:96271-96062;OR459777.1:96255-96046;OR459797.1:96270-96061;OR943698.1:98581-98372;PP098589.2:96252-96043;PP496048.1:96256-96047;PP601206.1:98512-98303;PP776553.1:96177-95968;PP852971.1:96250-96041;PP852983.1:96256-96047;PP853000.1:96217-96008;PP853005.1:96225-96016;PP853007.1:96258-96049;PP853013.1:96231-96022;PP853027.1:96262-96053;PP853045.1:96243-96034;PP853048.1:96262-96053;PP860026.1:96260-96051;PP860027.1:96285-96076;PP860028.1:96225-96016;PP860031.1:96279-96070;PP860033.1:96284-96075;PQ153226.1:96250-96041;PQ178862.2:97372-97163;PQ221506.1:92348-92139;PQ221833.1:98514-98305;PQ340477.1:96233-96024;PQ340478.1:96218-96009;PQ340480.1:96297-96088;PQ340483.1:96281-96072;PQ340485.1:96267-96058;PQ340486.1:96282-96073;PQ340487.1:96307-96098;PQ340489.1:96282-96073;PQ340490.1:96233-96024;PQ340491.1:96288-96079;PQ340493.1:96267-96058;PQ340495.1:96297-96088;PQ340496.1:96330-96121;PQ368489.1:96253-96044;PQ451835.1:96338-96129;PQ451836.1:96270-96061;PQ451838.1:96321-96112;PQ451841.1:96281-96072;PQ451842.1:96233-96024 |
| OG0000139 | AY603973.1:96608-96168;AY741551.1:96612-96172;AY753185.1:96851-96411;DQ011154.1:97082-96642;DQ011155.1:97063-96623;EPI_ISL_13331598:94682-94242;EPI_ISL_13363142:94675-94235;EPI_ISL_13374487:94690-94250;EPI_ISL_13502582:94675-94235;EPI_ISL_13508471:94767-94327;EPI_ISL_13607904:94666-94226;EPI_ISL_13624509:94610-94170;EPI_ISL_13889442:94785-94345;EPI_ISL_13953610:94583-94143;EPI_ISL_14181948:94811-94371;EPI_ISL_14838587:96936-96496;EPI_ISL_15263355:94666-94226;EPI_ISL_15269699:94635-94195;EPI_ISL_15293002:94908-94468;EPI_ISL_15641561:94624-94184;EPI_ISL_15831211:94673-94233;EPI_ISL_15992098:94688-94248;EPI_ISL_16233784:94684-94244;EPI_ISL_16946400:94673-94233;EPI_ISL_17048206:94323-93883;EPI_ISL_17703567:94688-94248;EPI_ISL_17718458:94666-94226;EPI_ISL_18213374:94663-94223;EPI_ISL_18360394:94324-93884;EPI_ISL_18557816:94664-94224;EPI_ISL_18642365:94666-94226;EPI_ISL_18659846:94636-94196;EPI_ISL_18744049:94668-94228;EPI_ISL_19022858:94663-94223;EPI_ISL_19028778:94580-94140;EPI_ISL_19151800:94664-94224;EPI_ISL_19159116:94666-94226;EPI_ISL_19350788:95808-95368;EPI_ISL_19359677:94663-94223;EPI_ISL_19446306:94658-94218;EPI_ISL_19467692:97219-96779;JX878411.1:97927-97487;JX878425.1:97444-97004;JX878426.1:96893-96453;KC257460.1:96861-96421;KJ642612.1:95179-94739;KJ642613.1:96738-96298;KJ642616.1:96302-95862;KJ642618.1:94409-93969;KP849469.1:97252-96812;KP849471.1:97178-96738;LC831698.1:94664-94224;MN648051.1:94886-94446;MN702445.1:90595-90155;MN702446.1:96740-96300;MN702447.1:90108-89668;MN702449.1:90606-90166;MN702452.1:96781-96341;MT903338.1:94829-94389;MT903340.1:94666-94226;MT903341.1:86199-85759;MT903342.1:94681-94241;MT903347.1:96619-96179;ON568298.1:94812-94372;ON622713.1:94898-94458;ON622722.2:94669-94229;ON631963.1:94689-94249;ON649879.1:94499-94059;ON676707.1:94719-94279;ON755039.1:94329-93889;ON808413.1:94669-94229;ON880420.1:94896-94456;ON911481.2:94686-94246;ON918656.1:94701-94261;ON959133.1:94790-94350;ON959136.1:94791-94351;ON983168.1:93364-92924;OP019276.1:94663-94223;OP150924.1:94804-94364;OP160532.1:94629-94189;OP235282.1:94663-94223;OP289782.1:94666-94226;OP331335.1:94683-94243;OP382479.1:94670-94230;OP422341.1:94683-94243;OP434519.1:94662-94222;OP535324.1:94664-94224;OP535333.1:94773-94333;OP536770.1:94665-94225;OP597769.1:94687-94247;OP604566.1:94666-94226;OP612682.1:94591-94151;OP612684.1:94654-94214;OP612686.1:94655-94215;OP696841.1:91940-91500;OP879722.1:93889-93449;OQ419007.1:93899-93459;OQ419012.1:93866-93426;OQ419031.1:93870-93430;OQ419047.1:93872-93432;OQ451898.1:93751-93311;OQ451900.1:94664-94224;OQ581853.1:94690-94250;OQ581855.1:94771-94331;OQ721951.1:94704-94264;OQ721954.1:94661-94221;OR035652.1:94613-94173;OR035664.1:94613-94173;OR035702.1:94613-94173;OR146272.1:94629-94189;OR146330.1:94674-94234;OR264364.1:94684-94244;OR427332.1:94684-94244;OR459777.1:94668-94228;OR459797.1:94683-94243;OR943698.1:96994-96554;PP098589.2:94665-94225;PP496048.1:94669-94229;PP601206.1:96925-96485;PP776553.1:94590-94150;PP852971.1:94663-94223;PP852983.1:94669-94229;PP853000.1:94630-94190;PP853005.1:94638-94198;PP853007.1:94671-94231;PP853013.1:94644-94204;PP853027.1:94675-94235;PP853045.1:94656-94216;PP853048.1:94675-94235;PP860026.1:94673-94233;PP860027.1:94698-94258;PP860028.1:94638-94198;PP860031.1:94692-94252;PP860033.1:94697-94257;PQ153226.1:94663-94223;PQ178862.2:95785-95345;PQ221506.1:90761-90321;PQ221833.1:96927-96487;PQ340477.1:94646-94206;PQ340478.1:94631-94191;PQ340480.1:94710-94270;PQ340483.1:94694-94254;PQ340485.1:94680-94240;PQ340486.1:94695-94255;PQ340487.1:94720-94280;PQ340489.1:94695-94255;PQ340490.1:94646-94206;PQ340491.1:94701-94261;PQ340493.1:94680-94240;PQ340495.1:94710-94270;PQ340496.1:94743-94303;PQ368489.1:94666-94226;PQ451835.1:94751-94311;PQ451836.1:94683-94243;PQ451838.1:94734-94294;PQ451841.1:94694-94254;PQ451842.1:94646-94206 |
| OG0000140 | AY603973.1:91394-89007;AY741551.1:91398-89011;AY753185.1:91637-89250;DQ011154.1:91853-89466;DQ011155.1:91834-89447;EPI_ISL_13331598:89462-87075;EPI_ISL_13363142:89455-87068;EPI_ISL_13374487:89470-87083;EPI_ISL_13502582:89455-87068;EPI_ISL_13508471:89547-87160;EPI_ISL_13607904:89446-87059;EPI_ISL_13624509:89390-87003;EPI_ISL_13889442:89565-87178;EPI_ISL_13953610:89064-86995;EPI_ISL_14181948:89591-87204;EPI_ISL_14838587:91707-89320;EPI_ISL_15263355:89446-87059;EPI_ISL_15269699:89415-87028;EPI_ISL_15293002:89688-87301;EPI_ISL_15641561:89404-87017;EPI_ISL_15831211:89453-87066;EPI_ISL_15992098:89468-87081;EPI_ISL_16233784:89464-87077;EPI_ISL_16946400:89453-87066;EPI_ISL_17048206:89103-86716;EPI_ISL_17703567:89468-87081;EPI_ISL_17718458:89446-87059;EPI_ISL_18213374:89443-87056;EPI_ISL_18360394:89104-86717;EPI_ISL_18557816:89444-87057;EPI_ISL_18642365:89446-87059;EPI_ISL_18659846:89416-87029;EPI_ISL_18744049:89448-87061;EPI_ISL_19022858:89443-87056;EPI_ISL_19028778:89360-86973;EPI_ISL_19151800:89444-87057;EPI_ISL_19159116:89446-87059;EPI_ISL_19350788:90579-88192;EPI_ISL_19359677:89443-87056;EPI_ISL_19446306:89438-87051;EPI_ISL_19467692:91990-89603;JX878411.1:92698-90311;JX878425.1:92215-89828;JX878426.1:91664-89277;KC257460.1:91632-89245;KJ642612.1:89950-87563;KJ642613.1:91509-89122;KJ642616.1:91088-88701;KJ642618.1:89180-86793;KP849469.1:92023-89636;KP849471.1:91949-89562;LC831698.1:89444-87057;MN648051.1:89666-87279;MN702445.1:85366-82979;MN702446.1:91511-89124;MN702447.1:84879-82492;MN702449.1:85377-82990;MN702452.1:91552-89165;MT903338.1:89609-87222;MT903340.1:89446-87059;MT903341.1:80979-78592;MT903342.1:89461-87074;MT903347.1:91405-89018;ON568298.1:89592-87205;ON622713.1:89678-87291;ON622722.2:89449-87062;ON631963.1:89469-87082;ON649879.1:89279-86892;ON676707.1:89499-87112;ON755039.1:89109-86722;ON808413.1:89449-87062;ON880420.1:89676-87289;ON911481.2:89466-87079;ON918656.1:89481-87094;ON959133.1:89570-87183;ON959136.1:89571-87184;ON983168.1:88144-85757;OP019276.1:89443-87056;OP150924.1:89584-87197;OP160532.1:89409-87022;OP235282.1:89443-87056;OP289782.1:89446-87059;OP331335.1:89463-87076;OP382479.1:89450-87063;OP422341.1:89463-87076;OP434519.1:89442-87055;OP535324.1:89444-87057;OP535333.1:89553-87166;OP536770.1:89445-87058;OP597769.1:89467-87080;OP604566.1:89446-87059;OP612682.1:89371-86984;OP612684.1:89075-87048;OP612686.1:89435-87048;OP696841.1:86720-84333;OP879722.1:88669-86282;OQ419007.1:88679-86292;OQ419012.1:88646-86259;OQ419031.1:88650-86263;OQ419047.1:88652-86265;OQ451898.1:88531-86144;OQ451900.1:89444-87057;OQ581853.1:89470-87083;OQ581855.1:89551-87164;OQ721951.1:89484-87097;OQ721954.1:89441-87054;OR035652.1:89393-87006;OR035664.1:89393-87006;OR035702.1:89393-87006;OR146272.1:89409-87022;OR146330.1:89454-87067;OR264364.1:89464-87077;OR427332.1:89464-87077;OR459777.1:89448-87061;OR459797.1:89463-87076;OR943698.1:91756-89369;PP098589.2:89445-87058;PP496048.1:89449-87062;PP601206.1:91705-89318;PP776553.1:89370-86983;PP852971.1:89443-87056;PP852983.1:89449-87062;PP853000.1:89410-87023;PP853005.1:89418-87031;PP853007.1:89451-87064;PP853013.1:89424-87037;PP853027.1:89455-87068;PP853045.1:89436-87049;PP853048.1:89455-87068;PP860026.1:89453-87066;PP860027.1:89478-87091;PP860028.1:89418-87031;PP860031.1:89472-87085;PP860033.1:89477-87090;PQ153226.1:89443-87056;PQ178862.2:90556-88169;PQ221506.1:85532-83145;PQ221833.1:91698-89311;PQ340477.1:89426-87039;PQ340478.1:89411-87024;PQ340480.1:89490-87103;PQ340483.1:89474-87087;PQ340485.1:89460-87073;PQ340486.1:89475-87088;PQ340487.1:89500-87113;PQ340489.1:89475-87088;PQ340490.1:89426-87039;PQ340491.1:89481-87094;PQ340493.1:89460-87073;PQ340495.1:89490-87103;PQ340496.1:89523-87136;PQ368489.1:89446-87059;PQ451835.1:89531-87144;PQ451836.1:89463-87076;PQ451838.1:89514-87127;PQ451841.1:89474-87087;PQ451842.1:89426-87039 |
| OG0000141 | AY603973.1:89006-88032;AY741551.1:89010-88036;AY753185.1:89249-88275;DQ011154.1:89465-88491;DQ011155.1:89446-88472;EPI_ISL_13331598:87074-86100;EPI_ISL_13363142:87067-86093;EPI_ISL_13374487:87082-86108;EPI_ISL_13502582:87067-86093;EPI_ISL_13508471:87159-86185;EPI_ISL_13607904:87058-86084;EPI_ISL_13624509:87002-86028;EPI_ISL_13889442:87177-86203;EPI_ISL_13953610:86994-86020;EPI_ISL_14181948:87203-86229;EPI_ISL_14838587:89319-88345;EPI_ISL_15263355:87058-86084;EPI_ISL_15269699:87027-86053;EPI_ISL_15293002:87300-86326;EPI_ISL_15641561:87016-86042;EPI_ISL_15831211:87065-86091;EPI_ISL_15992098:87080-86106;EPI_ISL_16233784:87076-86102;EPI_ISL_16946400:87065-86091;EPI_ISL_17048206:86715-85741;EPI_ISL_17703567:87080-86106;EPI_ISL_17718458:87058-86084;EPI_ISL_18213374:87055-86081;EPI_ISL_18360394:86716-85742;EPI_ISL_18557816:87056-86082;EPI_ISL_18642365:87058-86084;EPI_ISL_18659846:87028-86054;EPI_ISL_18744049:87060-86086;EPI_ISL_19022858:87055-86081;EPI_ISL_19028778:86972-85998;EPI_ISL_19151800:87056-86082;EPI_ISL_19159116:87058-86084;EPI_ISL_19350788:88191-87217;EPI_ISL_19359677:87055-86081;EPI_ISL_19446306:87050-86076;EPI_ISL_19467692:89602-88628;JX878411.1:90310-89336;JX878425.1:89827-88853;JX878426.1:89276-88302;KC257460.1:89244-88270;KJ642612.1:87562-86588;KJ642613.1:89121-88147;KJ642616.1:88700-87726;KJ642618.1:86792-85818;KP849469.1:89635-88661;KP849471.1:89561-88587;LC831698.1:87056-86082;MN648051.1:87278-86304;MN702445.1:82978-82004;MN702446.1:89123-88149;MN702447.1:82491-81517;MN702449.1:82989-82015;MN702452.1:89164-88190;MT903338.1:87221-86247;MT903340.1:87058-86084;MT903341.1:78591-77617;MT903342.1:87073-86099;MT903347.1:89017-88043;ON568298.1:87204-86230;ON622713.1:87290-86316;ON622722.2:87061-86087;ON631963.1:87081-86107;ON649879.1:86891-85917;ON676707.1:87111-86137;ON755039.1:86721-85747;ON808413.1:87061-86087;ON880420.1:87288-86314;ON911481.2:87078-86104;ON918656.1:87093-86119;ON959133.1:87182-86208;ON959136.1:87183-86209;ON983168.1:85756-84782;OP019276.1:87055-86081;OP150924.1:87196-86222;OP160532.1:87021-86047;OP235282.1:87055-86081;OP289782.1:87058-86084;OP331335.1:87075-86101;OP382479.1:87062-86088;OP422341.1:87075-86101;OP434519.1:87054-86080;OP535324.1:87056-86082;OP535333.1:87165-86191;OP536770.1:87057-86083;OP597769.1:87079-86105;OP604566.1:87058-86084;OP612682.1:86983-86009;OP612684.1:87047-86073;OP612686.1:87047-86073;OP696841.1:84332-83358;OP879722.1:86281-85307;OQ419007.1:86291-85317;OQ419012.1:86258-85284;OQ419031.1:86262-85288;OQ419047.1:86264-85290;OQ451898.1:86143-85169;OQ451900.1:87056-86082;OQ581853.1:87082-86108;OQ581855.1:87163-86189;OQ721951.1:87096-86122;OQ721954.1:87053-86079;OR035652.1:87005-86031;OR035664.1:87005-86031;OR035702.1:87005-86031;OR146272.1:87021-86047;OR146330.1:87066-86092;OR264364.1:87076-86102;OR427332.1:87076-86102;OR459777.1:87060-86086;OR459797.1:87075-86101;OR943698.1:89368-88394;PP098589.2:87057-86083;PP496048.1:87061-86087;PP601206.1:89317-88343;PP776553.1:86982-86008;PP852971.1:87055-86081;PP852983.1:87061-86087;PP853000.1:87022-86048;PP853005.1:87030-86056;PP853007.1:87063-86089;PP853013.1:87036-86062;PP853027.1:87067-86093;PP853045.1:87048-86074;PP853048.1:87067-86093;PP860026.1:87065-86091;PP860027.1:87090-86116;PP860028.1:87030-86056;PP860031.1:87084-86110;PP860033.1:87089-86115;PQ153226.1:87055-86081;PQ178862.2:88168-87194;PQ221506.1:83144-82170;PQ221833.1:89310-88336;PQ340477.1:87038-86064;PQ340478.1:87023-86049;PQ340480.1:87102-86128;PQ340483.1:87086-86112;PQ340485.1:87072-86098;PQ340486.1:87087-86113;PQ340487.1:87112-86138;PQ340489.1:87087-86113;PQ340490.1:87038-86064;PQ340491.1:87093-86119;PQ340493.1:87072-86098;PQ340495.1:87102-86128;PQ340496.1:87135-86161;PQ368489.1:87058-86084;PQ451835.1:87143-86169;PQ451836.1:87075-86101;PQ451838.1:87126-86152;PQ451841.1:87086-86112;PQ451842.1:87038-86064 |
| OG0000142 | AY603973.1:75071-74853;AY741551.1:75075-74857;AY753185.1:75314-75096;DQ011154.1:75532-75314;DQ011155.1:75513-75292;EPI_ISL_13331598:73141-72923;EPI_ISL_13363142:73134-72916;EPI_ISL_13374487:73149-72931;EPI_ISL_13502582:73134-72916;EPI_ISL_13508471:73226-73008;EPI_ISL_13607904:73125-72907;EPI_ISL_13624509:73069-72851;EPI_ISL_13889442:73244-73026;EPI_ISL_13953610:73061-72843;EPI_ISL_14181948:73270-73052;EPI_ISL_14838587:75386-75168;EPI_ISL_15263355:73125-72907;EPI_ISL_15269699:73094-72876;EPI_ISL_15293002:73367-73149;EPI_ISL_15641561:73083-72865;EPI_ISL_15831211:73132-72914;EPI_ISL_15992098:73147-72929;EPI_ISL_16233784:73143-72925;EPI_ISL_16946400:73132-72914;EPI_ISL_17048206:72782-72564;EPI_ISL_17703567:73147-72929;EPI_ISL_17718458:73125-72907;EPI_ISL_18213374:73122-72904;EPI_ISL_18360394:72783-72565;EPI_ISL_18557816:73123-72905;EPI_ISL_18642365:73125-72907;EPI_ISL_18659846:73095-72877;EPI_ISL_18744049:73127-72909;EPI_ISL_19022858:73122-72904;EPI_ISL_19028778:73039-72821;EPI_ISL_19151800:73123-72905;EPI_ISL_19159116:73125-72907;EPI_ISL_19350788:74258-74040;EPI_ISL_19359677:73122-72904;EPI_ISL_19446306:73117-72899;EPI_ISL_19467692:75669-75451;JX878411.1:76377-76159;JX878425.1:75894-75676;JX878426.1:75343-75125;KC257460.1:75311-75090;KJ642612.1:73629-73411;KJ642613.1:75188-74970;KJ642616.1:74765-74547;KJ642618.1:72796-72578;KP849469.1:75702-75484;KP849471.1:75628-75410;LC831698.1:73123-72905;MN648051.1:73345-73127;MN702445.1:69045-68827;MN702446.1:75190-74972;MN702447.1:68558-68340;MN702449.1:69056-68838;MN702452.1:75231-75013;MT903338.1:73288-73070;MT903340.1:73125-72907;MT903341.1:64658-64440;MT903342.1:73140-72922;MT903347.1:75082-74864;ON568298.1:73271-73053;ON622713.1:73357-73139;ON622722.2:73128-72910;ON631963.1:73148-72930;ON649879.1:72958-72740;ON676707.1:73178-72960;ON755039.1:72788-72570;ON808413.1:73128-72910;ON880420.1:73355-73137;ON911481.2:73145-72927;ON918656.1:73160-72942;ON959133.1:73249-73031;ON959136.1:73250-73032;ON983168.1:71824-71606;OP019276.1:73122-72904;OP150924.1:73263-73045;OP160532.1:73088-72870;OP235282.1:73122-72904;OP289782.1:73125-72907;OP331335.1:73142-72924;OP382479.1:73129-72911;OP422341.1:73142-72924;OP434519.1:73121-72903;OP535324.1:73123-72905;OP535333.1:73232-73014;OP536770.1:73124-72906;OP597769.1:73146-72928;OP604566.1:73125-72907;OP612682.1:73051-72833;OP612684.1:73115-72897;OP612686.1:73115-72897;OP696841.1:70399-70181;OP879722.1:72348-72130;OQ419007.1:72358-72140;OQ419012.1:72325-72107;OQ419031.1:72329-72111;OQ419047.1:72331-72113;OQ451898.1:72210-71992;OQ451900.1:73123-72905;OQ581853.1:73149-72931;OQ581855.1:73230-73012;OQ721951.1:73163-72945;OQ721954.1:73120-72902;OR035652.1:73072-72854;OR035664.1:73072-72854;OR035702.1:73072-72854;OR146272.1:73088-72870;OR146330.1:73133-72915;OR264364.1:73143-72925;OR427332.1:73143-72925;OR459777.1:73127-72909;OR459797.1:73142-72924;OR943698.1:75372-75154;PP098589.2:73124-72906;PP496048.1:73128-72910;PP601206.1:75384-75166;PP776553.1:73049-72831;PP852971.1:73122-72904;PP852983.1:73128-72910;PP853000.1:73089-72871;PP853005.1:73097-72879;PP853007.1:73130-72912;PP853013.1:73103-72885;PP853027.1:73134-72916;PP853045.1:73115-72897;PP853048.1:73134-72916;PP860026.1:73132-72914;PP860027.1:73157-72939;PP860028.1:73097-72879;PP860031.1:73151-72933;PP860033.1:73156-72938;PQ153226.1:73122-72904;PQ178862.2:74235-74017;PQ221506.1:69211-68993;PQ221833.1:75377-75159;PQ340477.1:73105-72887;PQ340478.1:73090-72872;PQ340480.1:73169-72951;PQ340483.1:73153-72935;PQ340485.1:73139-72921;PQ340486.1:73154-72936;PQ340487.1:73179-72961;PQ340489.1:73154-72936;PQ340490.1:73105-72887;PQ340491.1:73160-72942;PQ340493.1:73139-72921;PQ340495.1:73169-72951;PQ340496.1:73202-72984;PQ368489.1:73125-72907;PQ451835.1:73210-72992;PQ451836.1:73142-72924;PQ451838.1:73193-72975;PQ451841.1:73153-72935;PQ451842.1:73105-72887 |
| OG0000143 | AY603973.1:70808-70473;AY741551.1:70812-70477;AY753185.1:71051-70716;DQ011154.1:71269-70934;DQ011155.1:71247-70912;EPI_ISL_13331598:68878-68543;EPI_ISL_13363142:68871-68536;EPI_ISL_13374487:68886-68551;EPI_ISL_13502582:68871-68536;EPI_ISL_13508471:68963-68628;EPI_ISL_13607904:68862-68527;EPI_ISL_13624509:68806-68471;EPI_ISL_13889442:68981-68646;EPI_ISL_13953610:68798-68463;EPI_ISL_14181948:69007-68672;EPI_ISL_14838587:71123-70788;EPI_ISL_15263355:68862-68527;EPI_ISL_15269699:68831-68496;EPI_ISL_15293002:69104-68769;EPI_ISL_15641561:68820-68485;EPI_ISL_15831211:68869-68534;EPI_ISL_15992098:68884-68549;EPI_ISL_16233784:68880-68545;EPI_ISL_16946400:68869-68534;EPI_ISL_17048206:68519-68184;EPI_ISL_17703567:68884-68549;EPI_ISL_17718458:68862-68527;EPI_ISL_18213374:68859-68524;EPI_ISL_18360394:68520-68185;EPI_ISL_18557816:68860-68525;EPI_ISL_18642365:68862-68527;EPI_ISL_18659846:68832-68497;EPI_ISL_18744049:68864-68529;EPI_ISL_19022858:68859-68524;EPI_ISL_19028778:68776-68441;EPI_ISL_19151800:68860-68525;EPI_ISL_19159116:68862-68527;EPI_ISL_19350788:69995-69660;EPI_ISL_19359677:68859-68524;EPI_ISL_19446306:68854-68519;EPI_ISL_19467692:71406-71071;JX878411.1:72114-71779;JX878425.1:71631-71296;JX878426.1:71080-70745;KC257460.1:71045-70710;KJ642612.1:69366-69031;KJ642613.1:70925-70590;KJ642616.1:70502-70167;KJ642618.1:68533-68198;KP849469.1:71439-71104;KP849471.1:71364-71029;LC831698.1:68860-68525;MN648051.1:69082-68747;MN702445.1:64782-64447;MN702446.1:70927-70592;MN702447.1:64295-63960;MN702449.1:64793-64458;MN702452.1:70968-70633;MT903338.1:69025-68690;MT903340.1:68862-68527;MT903341.1:60395-60060;MT903342.1:68877-68542;MT903347.1:70819-70484;ON568298.1:69008-68673;ON622713.1:69094-68759;ON622722.2:68865-68530;ON631963.1:68885-68550;ON649879.1:68695-68360;ON676707.1:68915-68580;ON755039.1:68525-68190;ON808413.1:68865-68530;ON880420.1:69092-68757;ON911481.2:68882-68547;ON918656.1:68897-68562;ON959133.1:68986-68651;ON959136.1:68987-68652;ON983168.1:67561-67226;OP019276.1:68859-68524;OP150924.1:69000-68665;OP160532.1:68825-68490;OP235282.1:68859-68524;OP289782.1:68862-68527;OP331335.1:68879-68544;OP382479.1:68866-68531;OP422341.1:68879-68544;OP434519.1:68858-68523;OP535324.1:68860-68525;OP535333.1:68969-68634;OP536770.1:68861-68526;OP597769.1:68883-68548;OP604566.1:68862-68527;OP612682.1:68788-68453;OP612684.1:68852-68517;OP612686.1:68852-68517;OP696841.1:66136-65801;OP879722.1:68085-67750;OQ419007.1:68095-67760;OQ419012.1:68062-67727;OQ419031.1:68066-67731;OQ419047.1:68068-67733;OQ451898.1:67947-67612;OQ451900.1:68860-68525;OQ581853.1:68886-68551;OQ581855.1:68967-68632;OQ721951.1:68900-68565;OQ721954.1:68857-68522;OR035652.1:68809-68474;OR035664.1:68809-68474;OR035702.1:68809-68474;OR146272.1:68825-68490;OR146330.1:68870-68535;OR264364.1:68880-68545;OR427332.1:68880-68545;OR459777.1:68864-68529;OR459797.1:68879-68544;OR943698.1:71109-70774;PP098589.2:68861-68526;PP496048.1:68865-68530;PP601206.1:71121-70786;PP776553.1:68786-68451;PP852971.1:68859-68524;PP852983.1:68865-68530;PP853000.1:68826-68491;PP853005.1:68834-68499;PP853007.1:68867-68532;PP853013.1:68840-68505;PP853027.1:68871-68536;PP853045.1:68852-68517;PP853048.1:68871-68536;PP860026.1:68869-68534;PP860027.1:68894-68559;PP860028.1:68834-68499;PP860031.1:68888-68553;PP860033.1:68893-68558;PQ153226.1:68859-68524;PQ178862.2:69972-69637;PQ221506.1:64948-64613;PQ221833.1:71114-70779;PQ340477.1:68842-68507;PQ340478.1:68827-68492;PQ340480.1:68906-68571;PQ340483.1:68890-68555;PQ340485.1:68876-68541;PQ340486.1:68891-68556;PQ340487.1:68916-68581;PQ340489.1:68891-68556;PQ340490.1:68842-68507;PQ340491.1:68897-68562;PQ340493.1:68876-68541;PQ340495.1:68906-68571;PQ340496.1:68939-68604;PQ368489.1:68862-68527;PQ451835.1:68947-68612;PQ451836.1:68879-68544;PQ451838.1:68930-68595;PQ451841.1:68890-68555;PQ451842.1:68842-68507 |
| OG0000144 | AY603973.1:63962-61647;AY741551.1:63966-61651;AY753185.1:64205-61890;DQ011154.1:64423-62108;DQ011155.1:64401-62086;EPI_ISL_13331598:62033-59718;EPI_ISL_13363142:62026-59711;EPI_ISL_13374487:62041-59726;EPI_ISL_13502582:62026-59711;EPI_ISL_13508471:62118-59803;EPI_ISL_13607904:62017-59702;EPI_ISL_13624509:61961-59646;EPI_ISL_13889442:62136-59821;EPI_ISL_13953610:61953-59638;EPI_ISL_14181948:62162-59847;EPI_ISL_14838587:64277-61962;EPI_ISL_15263355:62017-59702;EPI_ISL_15269699:61986-59671;EPI_ISL_15293002:62259-59944;EPI_ISL_15641561:61975-59660;EPI_ISL_15831211:62024-59709;EPI_ISL_15992098:62039-59724;EPI_ISL_16233784:62035-59720;EPI_ISL_16946400:62024-59709;EPI_ISL_17048206:61674-59359;EPI_ISL_17703567:62039-59724;EPI_ISL_17718458:62017-59702;EPI_ISL_18213374:62014-59699;EPI_ISL_18360394:61675-59360;EPI_ISL_18557816:62015-59700;EPI_ISL_18642365:62017-59702;EPI_ISL_18659846:61987-59672;EPI_ISL_18744049:62019-59704;EPI_ISL_19022858:62014-59699;EPI_ISL_19028778:61931-59616;EPI_ISL_19151800:62015-59700;EPI_ISL_19159116:62017-59702;EPI_ISL_19350788:63149-60834;EPI_ISL_19359677:62014-59699;EPI_ISL_19446306:62009-59694;EPI_ISL_19467692:64560-62245;JX878411.1:65268-62953;JX878425.1:64785-62470;JX878426.1:64234-61919;KC257460.1:64199-61884;KJ642612.1:62520-60205;KJ642613.1:64079-61764;KJ642616.1:63656-61341;KJ642618.1:61687-59372;KP849469.1:64593-62278;KP849471.1:64518-62203;LC831698.1:62015-59700;MN648051.1:62237-59922;MN702445.1:57936-55621;MN702446.1:64081-61766;MN702447.1:57449-55134;MN702449.1:57947-55632;MN702452.1:64122-61807;MT903338.1:62180-59865;MT903340.1:62017-59702;MT903341.1:53550-51235;MT903342.1:62032-59717;MT903347.1:63973-61658;ON568298.1:62163-59848;ON622713.1:62249-59934;ON622722.2:62020-59705;ON631963.1:62040-59725;ON649879.1:61850-59535;ON676707.1:62070-59755;ON755039.1:61680-59365;ON808413.1:62020-59705;ON880420.1:62247-59932;ON911481.2:62037-59722;ON918656.1:62052-59737;ON959133.1:62141-59826;ON959136.1:62142-59827;ON983168.1:60716-58401;OP019276.1:62014-59699;OP150924.1:62155-59840;OP160532.1:61980-59665;OP235282.1:62014-59699;OP289782.1:62017-59702;OP331335.1:62034-59719;OP382479.1:62021-59706;OP422341.1:62034-59719;OP434519.1:62013-59698;OP535324.1:62015-59700;OP535333.1:62124-59809;OP536770.1:62016-59701;OP597769.1:62038-59723;OP604566.1:62017-59702;OP612682.1:61943-59628;OP612684.1:62007-59692;OP612686.1:62007-59692;OP696841.1:59291-56976;OP879722.1:61240-58925;OQ419007.1:61250-58935;OQ419012.1:61217-58902;OQ419031.1:61221-58906;OQ419047.1:61223-58908;OQ451898.1:61102-58787;OQ451900.1:62015-59700;OQ581853.1:62041-59726;OQ581855.1:62122-59807;OQ721951.1:62055-59740;OQ721954.1:62012-59697;OR035652.1:61964-59649;OR035664.1:61964-59649;OR035702.1:61964-59649;OR146272.1:61980-59665;OR146330.1:62025-59710;OR264364.1:62035-59720;OR427332.1:62035-59720;OR459777.1:62019-59704;OR459797.1:62034-59719;OR943698.1:64263-61948;PP098589.2:62016-59701;PP496048.1:62020-59705;PP601206.1:64275-61960;PP776553.1:61941-59626;PP852971.1:62014-59699;PP852983.1:62020-59705;PP853000.1:61981-59666;PP853005.1:61989-59674;PP853007.1:62022-59707;PP853013.1:61995-59680;PP853027.1:62026-59711;PP853045.1:62007-59692;PP853048.1:62026-59711;PP860026.1:62024-59709;PP860027.1:62049-59734;PP860028.1:61990-59675;PP860031.1:62043-59728;PP860033.1:62048-59733;PQ153226.1:62014-59699;PQ178862.2:63126-60811;PQ221506.1:58102-55787;PQ221833.1:64268-61953;PQ340477.1:61997-59682;PQ340478.1:61982-59667;PQ340480.1:62061-59746;PQ340483.1:62045-59730;PQ340485.1:62031-59716;PQ340486.1:62046-59731;PQ340487.1:62071-59756;PQ340489.1:62046-59731;PQ340490.1:61997-59682;PQ340491.1:62052-59737;PQ340493.1:62031-59716;PQ340495.1:62061-59746;PQ340496.1:62094-59779;PQ368489.1:62017-59702;PQ451835.1:62102-59787;PQ451836.1:62034-59719;PQ451838.1:62085-59770;PQ451841.1:62045-59730;PQ451842.1:61997-59682 |
| OG0000145 | AY603973.1:61565-60756;AY741551.1:61569-60760;AY753185.1:61808-60999;DQ011154.1:62026-61217;DQ011155.1:62004-61195;EPI_ISL_13331598:59636-58827;EPI_ISL_13363142:59629-58820;EPI_ISL_13374487:59644-58835;EPI_ISL_13502582:59629-58820;EPI_ISL_13508471:59721-58912;EPI_ISL_13607904:59620-58811;EPI_ISL_13624509:59564-58755;EPI_ISL_13889442:59739-58930;EPI_ISL_13953610:59556-58747;EPI_ISL_14181948:59765-58956;EPI_ISL_14838587:61880-61071;EPI_ISL_15263355:59620-58811;EPI_ISL_15269699:59589-58780;EPI_ISL_15293002:59862-59053;EPI_ISL_15641561:59578-58769;EPI_ISL_15831211:59627-58818;EPI_ISL_15992098:59642-58833;EPI_ISL_16233784:59638-58829;EPI_ISL_16946400:59627-58818;EPI_ISL_17048206:59277-58468;EPI_ISL_17703567:59642-58833;EPI_ISL_17718458:59620-58811;EPI_ISL_18213374:59617-58808;EPI_ISL_18360394:59278-58469;EPI_ISL_18557816:59618-58809;EPI_ISL_18642365:59620-58811;EPI_ISL_18659846:59590-58781;EPI_ISL_18744049:59622-58813;EPI_ISL_19022858:59617-58808;EPI_ISL_19028778:59534-58725;EPI_ISL_19151800:59618-58809;EPI_ISL_19159116:59620-58811;EPI_ISL_19350788:60752-59943;EPI_ISL_19359677:59617-58808;EPI_ISL_19446306:59612-58803;EPI_ISL_19467692:62163-61354;JX878411.1:62871-62062;JX878425.1:62388-61579;JX878426.1:61837-61028;KC257460.1:61802-60993;KJ642612.1:60123-59314;KJ642613.1:61682-60873;KJ642616.1:61259-60450;KJ642618.1:59290-58481;KP849469.1:62196-61387;KP849471.1:62121-61312;LC831698.1:59618-58809;MN648051.1:59840-59031;MN702445.1:55539-54730;MN702446.1:61684-60875;MN702447.1:55052-54243;MN702449.1:55550-54741;MN702452.1:61725-60916;MT903338.1:59783-58974;MT903340.1:59620-58811;MT903341.1:51153-50344;MT903342.1:59635-58826;MT903347.1:61576-60767;ON568298.1:59766-58957;ON622713.1:59852-59043;ON622722.2:59623-58814;ON631963.1:59643-58834;ON649879.1:59453-58644;ON676707.1:59673-58864;ON755039.1:59283-58474;ON808413.1:59623-58814;ON880420.1:59850-59041;ON911481.2:59640-58831;ON918656.1:59655-58846;ON959133.1:59744-58935;ON959136.1:59745-58936;ON983168.1:58319-57510;OP019276.1:59617-58808;OP150924.1:59758-58949;OP160532.1:59583-58774;OP235282.1:59617-58808;OP289782.1:59620-58811;OP331335.1:59637-58828;OP382479.1:59624-58815;OP422341.1:59637-58828;OP434519.1:59616-58807;OP535324.1:59618-58809;OP535333.1:59727-58918;OP536770.1:59619-58810;OP597769.1:59641-58832;OP604566.1:59620-58811;OP612682.1:59546-58737;OP612684.1:59610-58801;OP612686.1:59610-58801;OP696841.1:56894-56085;OP879722.1:58843-58034;OQ419007.1:58853-58044;OQ419012.1:58820-58011;OQ419031.1:58824-58015;OQ419047.1:58826-58017;OQ451898.1:58705-57896;OQ451900.1:59618-58809;OQ581853.1:59644-58835;OQ581855.1:59725-58916;OQ721951.1:59658-58849;OQ721954.1:59615-58806;OR035652.1:59567-58758;OR035664.1:59567-58758;OR035702.1:59567-58758;OR146272.1:59583-58774;OR146330.1:59628-58819;OR264364.1:59638-58829;OR427332.1:59638-58829;OR459777.1:59622-58813;OR459797.1:59637-58828;OR943698.1:61866-61057;PP098589.2:59619-58810;PP496048.1:59623-58814;PP601206.1:61878-61069;PP776553.1:59544-58735;PP852971.1:59617-58808;PP852983.1:59623-58814;PP853000.1:59584-58775;PP853005.1:59592-58783;PP853007.1:59625-58816;PP853013.1:59598-58789;PP853027.1:59629-58820;PP853045.1:59610-58801;PP853048.1:59629-58820;PP860026.1:59627-58818;PP860027.1:59652-58843;PP860028.1:59593-58784;PP860031.1:59646-58837;PP860033.1:59651-58842;PQ153226.1:59617-58808;PQ178862.2:60729-59920;PQ221506.1:55705-54896;PQ221833.1:61871-61062;PQ340477.1:59600-58791;PQ340478.1:59585-58776;PQ340480.1:59664-58855;PQ340483.1:59648-58839;PQ340485.1:59634-58825;PQ340486.1:59649-58840;PQ340487.1:59674-58865;PQ340489.1:59649-58840;PQ340490.1:59600-58791;PQ340491.1:59655-58846;PQ340493.1:59634-58825;PQ340495.1:59664-58855;PQ340496.1:59697-58888;PQ368489.1:59620-58811;PQ451835.1:59705-58896;PQ451836.1:59637-58828;PQ451838.1:59688-58879;PQ451841.1:59648-58839;PQ451842.1:59600-58791 |
| OG0000146 | AY603973.1:60755-60534;AY741551.1:60759-60538;AY753185.1:60998-60777;DQ011154.1:61216-60995;DQ011155.1:61194-60973;EPI_ISL_13331598:58826-58605;EPI_ISL_13363142:58819-58598;EPI_ISL_13374487:58834-58613;EPI_ISL_13502582:58819-58598;EPI_ISL_13508471:58911-58690;EPI_ISL_13607904:58810-58589;EPI_ISL_13624509:58754-58533;EPI_ISL_13889442:58929-58708;EPI_ISL_13953610:58746-58525;EPI_ISL_14181948:58955-58734;EPI_ISL_14838587:61070-60849;EPI_ISL_15263355:58810-58589;EPI_ISL_15269699:58779-58558;EPI_ISL_15293002:59052-58831;EPI_ISL_15641561:58768-58547;EPI_ISL_15831211:58817-58596;EPI_ISL_15992098:58832-58611;EPI_ISL_16233784:58828-58607;EPI_ISL_16946400:58817-58596;EPI_ISL_17048206:58467-58246;EPI_ISL_17703567:58832-58611;EPI_ISL_17718458:58810-58589;EPI_ISL_18213374:58807-58586;EPI_ISL_18360394:58468-58247;EPI_ISL_18557816:58808-58587;EPI_ISL_18642365:58810-58589;EPI_ISL_18659846:58780-58559;EPI_ISL_18744049:58812-58591;EPI_ISL_19022858:58807-58586;EPI_ISL_19028778:58724-58503;EPI_ISL_19151800:58808-58587;EPI_ISL_19159116:58810-58589;EPI_ISL_19350788:59942-59721;EPI_ISL_19359677:58807-58586;EPI_ISL_19446306:58802-58581;EPI_ISL_19467692:61353-61132;JX878411.1:62061-61840;JX878425.1:61578-61357;JX878426.1:61027-60806;KC257460.1:60992-60771;KJ642612.1:59313-59092;KJ642613.1:60872-60651;KJ642616.1:60449-60228;KJ642618.1:58480-58259;KP849469.1:61386-61165;KP849471.1:61311-61090;LC831698.1:58808-58587;MN648051.1:59030-58809;MN702445.1:54729-54508;MN702446.1:60874-60653;MN702447.1:54242-54021;MN702449.1:54740-54519;MN702452.1:60915-60694;MT903338.1:58973-58752;MT903340.1:58810-58589;MT903341.1:50343-50122;MT903342.1:58825-58604;MT903347.1:60766-60545;ON568298.1:58956-58735;ON622713.1:59042-58821;ON622722.2:58813-58592;ON631963.1:58833-58612;ON649879.1:58643-58422;ON676707.1:58863-58642;ON755039.1:58473-58252;ON808413.1:58813-58592;ON880420.1:59040-58819;ON911481.2:58830-58609;ON918656.1:58845-58624;ON959133.1:58934-58713;ON959136.1:58935-58714;ON983168.1:57509-57288;OP019276.1:58807-58586;OP150924.1:58948-58727;OP160532.1:58773-58552;OP235282.1:58807-58586;OP289782.1:58810-58589;OP331335.1:58827-58606;OP382479.1:58814-58593;OP422341.1:58827-58606;OP434519.1:58806-58585;OP535324.1:58808-58587;OP535333.1:58917-58696;OP536770.1:58809-58588;OP597769.1:58831-58610;OP604566.1:58810-58589;OP612682.1:58736-58515;OP612684.1:58800-58579;OP612686.1:58800-58579;OP696841.1:56084-55863;OP879722.1:58033-57812;OQ419007.1:58043-57822;OQ419012.1:58010-57789;OQ419031.1:58014-57793;OQ419047.1:58016-57795;OQ451898.1:57895-57674;OQ451900.1:58808-58587;OQ581853.1:58834-58613;OQ581855.1:58915-58694;OQ721951.1:58848-58627;OQ721954.1:58805-58584;OR035652.1:58757-58536;OR035664.1:58757-58536;OR035702.1:58757-58536;OR146272.1:58773-58552;OR146330.1:58818-58597;OR264364.1:58828-58607;OR427332.1:58828-58607;OR459777.1:58812-58591;OR459797.1:58827-58606;OR943698.1:61056-60835;PP098589.2:58809-58588;PP496048.1:58813-58592;PP601206.1:61068-60847;PP776553.1:58734-58513;PP852971.1:58807-58586;PP852983.1:58813-58592;PP853000.1:58774-58553;PP853005.1:58782-58561;PP853007.1:58815-58594;PP853013.1:58788-58567;PP853027.1:58819-58598;PP853045.1:58800-58579;PP853048.1:58819-58598;PP860026.1:58817-58596;PP860027.1:58842-58621;PP860028.1:58783-58562;PP860031.1:58836-58615;PP860033.1:58841-58620;PQ153226.1:58807-58586;PQ178862.2:59919-59698;PQ221506.1:54895-54674;PQ221833.1:61061-60840;PQ340477.1:58790-58569;PQ340478.1:58775-58554;PQ340480.1:58854-58633;PQ340483.1:58838-58617;PQ340485.1:58824-58603;PQ340486.1:58839-58618;PQ340487.1:58864-58643;PQ340489.1:58839-58618;PQ340490.1:58790-58569;PQ340491.1:58845-58624;PQ340493.1:58824-58603;PQ340495.1:58854-58633;PQ340496.1:58887-58666;PQ368489.1:58810-58589;PQ451835.1:58895-58674;PQ451836.1:58827-58606;PQ451838.1:58878-58657;PQ451841.1:58838-58617;PQ451842.1:58790-58569 |
| OG0000147 | AY603973.1:60527-59589;AY741551.1:60531-59593;AY753185.1:60770-59832;DQ011154.1:60988-60050;DQ011155.1:60966-60028;EPI_ISL_13331598:58598-57660;EPI_ISL_13363142:58591-57653;EPI_ISL_13374487:58606-57668;EPI_ISL_13502582:58591-57653;EPI_ISL_13508471:58683-57745;EPI_ISL_13607904:58582-57644;EPI_ISL_13624509:58526-57588;EPI_ISL_13889442:58701-57763;EPI_ISL_13953610:58518-57580;EPI_ISL_14181948:58727-57789;EPI_ISL_14838587:60842-59904;EPI_ISL_15263355:58582-57644;EPI_ISL_15269699:58551-57613;EPI_ISL_15293002:58824-57886;EPI_ISL_15641561:58540-57602;EPI_ISL_15831211:58589-57651;EPI_ISL_15992098:58604-57666;EPI_ISL_16233784:58600-57662;EPI_ISL_16946400:58589-57651;EPI_ISL_17048206:58239-57301;EPI_ISL_17703567:58604-57666;EPI_ISL_17718458:58582-57644;EPI_ISL_18213374:58579-57641;EPI_ISL_18360394:58240-57302;EPI_ISL_18557816:58580-57642;EPI_ISL_18642365:58582-57644;EPI_ISL_18659846:58552-57614;EPI_ISL_18744049:58584-57646;EPI_ISL_19022858:58579-57641;EPI_ISL_19028778:58496-57558;EPI_ISL_19151800:58580-57642;EPI_ISL_19159116:58582-57644;EPI_ISL_19350788:59714-58776;EPI_ISL_19359677:58579-57641;EPI_ISL_19446306:58574-57636;EPI_ISL_19467692:61125-60187;JX878411.1:61833-60895;JX878425.1:61350-60412;JX878426.1:60799-59861;KC257460.1:60764-59826;KJ642612.1:59085-58147;KJ642613.1:60644-59706;KJ642616.1:60221-59283;KJ642618.1:58252-57314;KP849469.1:61158-60220;KP849471.1:61083-60145;LC831698.1:58580-57642;MN648051.1:58802-57864;MN702445.1:54501-53563;MN702446.1:60646-59708;MN702447.1:54014-53076;MN702449.1:54512-53574;MN702452.1:60687-59749;MT903338.1:58745-57807;MT903340.1:58582-57644;MT903341.1:50115-49177;MT903342.1:58597-57659;MT903347.1:60538-59600;ON568298.1:58728-57790;ON622713.1:58814-57876;ON622722.2:58585-57647;ON631963.1:58605-57667;ON649879.1:58415-57477;ON676707.1:58635-57697;ON755039.1:58245-57307;ON808413.1:58585-57647;ON880420.1:58812-57874;ON911481.2:58602-57664;ON918656.1:58617-57679;ON959133.1:58706-57768;ON959136.1:58707-57769;ON983168.1:57281-56343;OP019276.1:58579-57641;OP150924.1:58720-57782;OP160532.1:58545-57607;OP235282.1:58579-57641;OP289782.1:58582-57644;OP331335.1:58599-57661;OP382479.1:58586-57648;OP422341.1:58599-57661;OP434519.1:58578-57640;OP535324.1:58580-57642;OP535333.1:58689-57751;OP536770.1:58581-57643;OP597769.1:58603-57665;OP604566.1:58582-57644;OP612682.1:58508-57570;OP612684.1:58572-57634;OP612686.1:58572-57634;OP696841.1:55856-54918;OP879722.1:57805-56867;OQ419007.1:57815-56877;OQ419012.1:57782-56844;OQ419031.1:57786-56848;OQ419047.1:57788-56850;OQ451898.1:57667-56729;OQ451900.1:58580-57642;OQ581853.1:58606-57668;OQ581855.1:58687-57749;OQ721951.1:58620-57682;OQ721954.1:58577-57639;OR035652.1:58529-57591;OR035664.1:58529-57591;OR035702.1:58529-57591;OR146272.1:58545-57607;OR146330.1:58590-57652;OR264364.1:58600-57662;OR427332.1:58600-57662;OR459777.1:58584-57646;OR459797.1:58599-57661;OR943698.1:60828-59890;PP098589.2:58581-57643;PP496048.1:58585-57647;PP601206.1:60840-59902;PP776553.1:58506-57568;PP852971.1:58579-57641;PP852983.1:58585-57647;PP853000.1:58546-57608;PP853005.1:58554-57616;PP853007.1:58587-57649;PP853013.1:58560-57622;PP853027.1:58591-57653;PP853045.1:58572-57634;PP853048.1:58591-57653;PP860026.1:58589-57651;PP860027.1:58614-57676;PP860028.1:58555-57617;PP860031.1:58608-57670;PP860033.1:58613-57675;PQ153226.1:58579-57641;PQ178862.2:59691-58753;PQ221506.1:54667-53729;PQ221833.1:60833-59895;PQ340477.1:58562-57624;PQ340478.1:58547-57609;PQ340480.1:58626-57688;PQ340483.1:58610-57672;PQ340485.1:58596-57658;PQ340486.1:58611-57673;PQ340487.1:58636-57698;PQ340489.1:58611-57673;PQ340490.1:58562-57624;PQ340491.1:58617-57679;PQ340493.1:58596-57658;PQ340495.1:58626-57688;PQ340496.1:58659-57721;PQ368489.1:58582-57644;PQ451835.1:58667-57729;PQ451836.1:58599-57661;PQ451838.1:58650-57712;PQ451841.1:58610-57672;PQ451842.1:58562-57624 |
| OG0000148 | AY603973.1:59069-57072;AY741551.1:59073-57076;AY753185.1:59312-57315;DQ011154.1:59530-57533;DQ011155.1:59508-57511;EPI_ISL_13331598:57140-55143;EPI_ISL_13363142:57133-55136;EPI_ISL_13374487:57148-55151;EPI_ISL_13502582:57133-55136;EPI_ISL_13508471:57225-55228;EPI_ISL_13607904:57124-55127;EPI_ISL_13624509:57068-55071;EPI_ISL_13889442:57243-55246;EPI_ISL_13953610:57060-55063;EPI_ISL_14181948:57269-55272;EPI_ISL_14838587:59384-57387;EPI_ISL_15263355:57124-55127;EPI_ISL_15269699:57093-55096;EPI_ISL_15293002:57366-55369;EPI_ISL_15641561:57082-55085;EPI_ISL_15831211:57131-55134;EPI_ISL_15992098:57146-55149;EPI_ISL_16233784:57142-55145;EPI_ISL_16946400:57131-55134;EPI_ISL_17048206:56781-54784;EPI_ISL_17703567:57146-55149;EPI_ISL_17718458:57124-55127;EPI_ISL_18213374:57121-55124;EPI_ISL_18360394:56782-54785;EPI_ISL_18557816:57122-55125;EPI_ISL_18642365:57124-55127;EPI_ISL_18659846:57094-55097;EPI_ISL_18744049:57126-55129;EPI_ISL_19022858:57121-55124;EPI_ISL_19028778:57038-55041;EPI_ISL_19151800:57122-55125;EPI_ISL_19159116:57124-55127;EPI_ISL_19350788:58256-56259;EPI_ISL_19359677:57121-55124;EPI_ISL_19446306:57116-55119;EPI_ISL_19467692:59667-57670;JX878411.1:60375-58378;JX878425.1:59892-57895;JX878426.1:59341-57344;KC257460.1:59306-57309;KJ642612.1:57627-55630;KJ642613.1:59186-57189;KJ642616.1:58763-56766;KJ642618.1:56794-54797;KP849469.1:59700-57703;KP849471.1:59625-57628;LC831698.1:57122-55125;MN648051.1:57344-55347;MN702445.1:53043-51046;MN702446.1:59188-57191;MN702447.1:52556-50559;MN702449.1:53054-51057;MN702452.1:59229-57232;MT903338.1:57287-55290;MT903340.1:57124-55127;MT903341.1:48657-46660;MT903342.1:57139-55142;MT903347.1:59080-57083;ON568298.1:57270-55273;ON622713.1:57356-55359;ON622722.2:57127-55130;ON631963.1:57147-55150;ON649879.1:56957-54960;ON676707.1:57177-55180;ON755039.1:56787-54790;ON808413.1:57127-55130;ON880420.1:57354-55357;ON911481.2:57144-55147;ON918656.1:57159-55162;ON959133.1:57248-55251;ON959136.1:57249-55252;ON983168.1:55823-53826;OP019276.1:57121-55124;OP150924.1:57262-55265;OP160532.1:57087-55090;OP235282.1:57121-55124;OP289782.1:57124-55127;OP331335.1:57141-55144;OP382479.1:57128-55131;OP422341.1:57141-55144;OP434519.1:57120-55123;OP535324.1:57122-55125;OP535333.1:57231-55234;OP536770.1:57123-55126;OP597769.1:57145-55148;OP604566.1:57124-55127;OP612682.1:57050-55053;OP612684.1:57114-55117;OP612686.1:57114-55117;OP696841.1:54398-52401;OP879722.1:56347-54350;OQ419007.1:56357-54360;OQ419012.1:56324-54327;OQ419031.1:56328-54331;OQ419047.1:56357-54360;OQ451898.1:56209-54212;OQ451900.1:57122-55125;OQ581853.1:57148-55151;OQ581855.1:57229-55232;OQ721951.1:57162-55165;OQ721954.1:57119-55122;OR035652.1:57071-55074;OR035664.1:57071-55074;OR035702.1:57071-55074;OR146272.1:57087-55090;OR146330.1:57132-55135;OR264364.1:57142-55145;OR427332.1:57142-55145;OR459777.1:57126-55129;OR459797.1:57141-55144;OR943698.1:59370-57373;PP098589.2:57123-55126;PP496048.1:57127-55130;PP601206.1:59382-57385;PP776553.1:57048-55051;PP852971.1:57121-55124;PP852983.1:57127-55130;PP853000.1:57088-55091;PP853005.1:57096-55099;PP853007.1:57129-55132;PP853013.1:57102-55105;PP853027.1:57133-55136;PP853045.1:57114-55117;PP853048.1:57133-55136;PP860026.1:57131-55134;PP860027.1:57156-55159;PP860028.1:57097-55100;PP860031.1:57150-55153;PP860033.1:57155-55158;PQ153226.1:57121-55124;PQ178862.2:58233-56236;PQ221506.1:53209-51212;PQ221833.1:59375-57378;PQ340477.1:57104-55107;PQ340478.1:57089-55092;PQ340480.1:57168-55171;PQ340483.1:57152-55155;PQ340485.1:57138-55141;PQ340486.1:57153-55156;PQ340487.1:57178-55181;PQ340489.1:57153-55156;PQ340490.1:57104-55107;PQ340491.1:57159-55162;PQ340493.1:57138-55141;PQ340495.1:57168-55171;PQ340496.1:57201-55204;PQ368489.1:57124-55127;PQ451835.1:57209-55212;PQ451836.1:57141-55144;PQ451838.1:57192-55195;PQ451841.1:57152-55155;PQ451842.1:57104-55107 |
| OG0000149 | AY603973.1:45632-44193;AY741551.1:45636-44197;AY753185.1:45875-44436;DQ011154.1:45850-44411;DQ011155.1:45831-44392;EPI_ISL_13331598:43703-42264;EPI_ISL_13363142:43696-42257;EPI_ISL_13374487:43711-42272;EPI_ISL_13502582:43696-42257;EPI_ISL_13508471:43788-42349;EPI_ISL_13607904:43687-42248;EPI_ISL_13624509:43631-42192;EPI_ISL_13889442:43806-42367;EPI_ISL_13953610:43647-42868;EPI_ISL_14181948:43832-42393;EPI_ISL_14838587:45704-44265;EPI_ISL_15263355:43687-42248;EPI_ISL_15269699:43656-42217;EPI_ISL_15293002:43929-42490;EPI_ISL_15641561:43645-42206;EPI_ISL_15831211:43694-42255;EPI_ISL_15992098:43709-42270;EPI_ISL_16233784:43705-42266;EPI_ISL_16946400:43694-42255;EPI_ISL_17048206:43344-41905;EPI_ISL_17703567:43709-42270;EPI_ISL_17718458:43687-42248;EPI_ISL_18213374:43684-42245;EPI_ISL_18360394:43345-41906;EPI_ISL_18557816:43685-42246;EPI_ISL_18642365:43687-42248;EPI_ISL_18659846:43657-42218;EPI_ISL_18744049:43689-42250;EPI_ISL_19022858:43684-42245;EPI_ISL_19028778:43601-42162;EPI_ISL_19151800:43685-42246;EPI_ISL_19159116:43687-42248;EPI_ISL_19350788:44578-43139;EPI_ISL_19359677:43684-42245;EPI_ISL_19446306:43679-42240;EPI_ISL_19467692:45983-44544;JX878411.1:46698-45259;JX878425.1:46212-44773;JX878426.1:45657-44218;KC257460.1:45628-44189;KJ642612.1:43943-42504;KJ642613.1:45520-44081;KJ642616.1:45326-43887;KJ642618.1:43111-41672;KP849469.1:46020-44581;KP849471.1:45946-44507;LC831698.1:43685-42246;MN648051.1:43907-42468;MN702445.1:39365-37926;MN702446.1:45510-44071;MN702447.1:38876-37437;MN702449.1:39376-37937;MN702452.1:45549-44110;MT903338.1:43850-42411;MT903340.1:43687-42248;MT903341.1:35220-33781;MT903342.1:43702-42263;MT903347.1:45645-44206;ON568298.1:43833-42394;ON622713.1:43919-42480;ON622722.2:43690-42251;ON631963.1:43710-42271;ON649879.1:43520-42081;ON676707.1:43740-42301;ON755039.1:43350-41911;ON808413.1:43690-42251;ON880420.1:43917-42478;ON911481.2:43707-42268;ON918656.1:43722-42283;ON959133.1:43811-42372;ON959136.1:43812-42373;ON983168.1:42386-40947;OP019276.1:43684-42245;OP150924.1:43825-42386;OP160532.1:43650-42211;OP235282.1:43684-42245;OP289782.1:43687-42248;OP331335.1:43704-42265;OP382479.1:43691-42252;OP422341.1:43704-42265;OP434519.1:43683-42244;OP535324.1:43685-42246;OP535333.1:43794-42355;OP536770.1:43686-42247;OP597769.1:43708-42269;OP604566.1:43687-42248;OP612682.1:43613-42174;OP612684.1:43677-42238;OP612686.1:43677-42238;OP696841.1:40961-39522;OP879722.1:42910-41471;OQ419007.1:42920-41481;OQ419012.1:42909-41470;OQ419031.1:42891-41452;OQ419047.1:42920-41481;OQ451898.1:42772-41333;OQ451900.1:43685-42246;OQ581853.1:43711-42272;OQ581855.1:43792-42353;OQ721951.1:43725-42286;OQ721954.1:43682-42243;OR035652.1:43634-42195;OR035664.1:43634-42195;OR035702.1:43634-42195;OR146272.1:43650-42211;OR146330.1:43695-42256;OR264364.1:43705-42266;OR427332.1:43705-42266;OR459777.1:43689-42250;OR459797.1:43704-42265;OR943698.1:45685-44246;PP098589.2:43686-42247;PP496048.1:43690-42251;PP601206.1:45702-44263;PP776553.1:43611-42172;PP852971.1:43684-42245;PP852983.1:43690-42251;PP853000.1:43651-42212;PP853005.1:43659-42220;PP853007.1:43692-42253;PP853013.1:43665-42226;PP853027.1:43696-42257;PP853045.1:43677-42238;PP853048.1:43696-42257;PP860026.1:43694-42255;PP860027.1:43719-42280;PP860028.1:43660-42221;PP860031.1:43713-42274;PP860033.1:43718-42279;PQ153226.1:43684-42245;PQ178862.2:44555-43116;PQ221506.1:39525-38086;PQ221833.1:45694-44255;PQ340477.1:43667-42228;PQ340478.1:43652-42213;PQ340480.1:43731-42292;PQ340483.1:43715-42276;PQ340485.1:43701-42262;PQ340486.1:43716-42277;PQ340487.1:43741-42302;PQ340489.1:43716-42277;PQ340490.1:43667-42228;PQ340491.1:43722-42283;PQ340493.1:43701-42262;PQ340495.1:43731-42292;PQ340496.1:43764-42325;PQ368489.1:43687-42248;PQ451835.1:43772-42333;PQ451836.1:43704-42265;PQ451838.1:43755-42316;PQ451841.1:43715-42276;PQ451842.1:43667-42228 |
| OG0000150 | AY603973.1:37940-36621;AY741551.1:37944-36625;AY753185.1:38183-36864;DQ011154.1:38160-36841;DQ011155.1:38141-36822;EPI_ISL_13331598:36011-34692;EPI_ISL_13363142:36004-34685;EPI_ISL_13374487:36019-34700;EPI_ISL_13502582:36004-34685;EPI_ISL_13508471:36096-34777;EPI_ISL_13607904:35995-34676;EPI_ISL_13624509:35939-34620;EPI_ISL_13889442:36114-34795;EPI_ISL_13953610:35962-34643;EPI_ISL_14181948:36140-34821;EPI_ISL_14838587:38014-36695;EPI_ISL_15263355:35995-34676;EPI_ISL_15269699:35964-34645;EPI_ISL_15293002:36237-34918;EPI_ISL_15641561:35953-34634;EPI_ISL_15831211:36002-34683;EPI_ISL_15992098:36017-34698;EPI_ISL_16233784:36013-34694;EPI_ISL_16946400:36002-34683;EPI_ISL_17048206:35652-34333;EPI_ISL_17703567:36017-34698;EPI_ISL_17718458:35995-34676;EPI_ISL_18213374:35992-34673;EPI_ISL_18360394:35653-34334;EPI_ISL_18557816:35993-34674;EPI_ISL_18642365:35995-34676;EPI_ISL_18659846:35965-34646;EPI_ISL_18744049:35997-34678;EPI_ISL_19022858:35992-34673;EPI_ISL_19028778:35909-34590;EPI_ISL_19151800:35993-34674;EPI_ISL_19159116:35995-34676;EPI_ISL_19350788:36888-35569;EPI_ISL_19359677:35992-34673;EPI_ISL_19446306:35987-34668;EPI_ISL_19467692:38293-36974;JX878411.1:39008-37689;JX878425.1:38522-37203;JX878426.1:37967-36648;KC257460.1:37938-36619;KJ642612.1:36253-34934;KJ642613.1:37829-36510;KJ642616.1:37634-36315;KJ642618.1:35421-34102;KP849469.1:38330-37011;KP849471.1:38256-36937;LC831698.1:35993-34674;MN648051.1:36215-34896;MN702445.1:31675-30356;MN702446.1:37821-36502;MN702447.1:31186-29867;MN702449.1:31686-30367;MN702452.1:37859-36540;MT903338.1:36158-34839;MT903340.1:35995-34676;MT903341.1:27528-26209;MT903342.1:36010-34691;MT903347.1:37953-36634;ON568298.1:36141-34822;ON622713.1:36227-34908;ON622722.2:35998-34679;ON631963.1:36018-34699;ON649879.1:35828-34509;ON676707.1:36048-34729;ON755039.1:35658-34339;ON808413.1:35998-34679;ON880420.1:36225-34906;ON911481.2:36015-34696;ON918656.1:36030-34711;ON959133.1:36119-34800;ON959136.1:36120-34801;ON983168.1:34694-33375;OP019276.1:35992-34673;OP150924.1:36133-34814;OP160532.1:35958-34639;OP235282.1:35992-34673;OP289782.1:35995-34676;OP331335.1:36012-34693;OP382479.1:35999-34680;OP422341.1:36012-34693;OP434519.1:35991-34672;OP535324.1:35993-34674;OP535333.1:36102-34783;OP536770.1:35994-34675;OP597769.1:36016-34697;OP604566.1:35995-34676;OP612682.1:35921-34602;OP612684.1:35985-34666;OP612686.1:35985-34666;OP696841.1:33269-31950;OP879722.1:35218-33899;OQ419007.1:35228-33909;OQ419012.1:35220-33901;OQ419031.1:35199-33880;OQ419047.1:35228-33909;OQ451898.1:35080-33761;OQ451900.1:35993-34674;OQ581853.1:36019-34700;OQ581855.1:36100-34781;OQ721951.1:36033-34714;OQ721954.1:35990-34671;OR035652.1:35942-34623;OR035664.1:35942-34623;OR035702.1:35942-34623;OR146272.1:35958-34639;OR146330.1:36003-34684;OR264364.1:36013-34694;OR427332.1:36013-34694;OR459777.1:35997-34678;OR459797.1:36012-34693;OR943698.1:37995-36676;PP098589.2:35994-34675;PP496048.1:35998-34679;PP601206.1:38012-36693;PP776553.1:35919-34600;PP852971.1:35992-34673;PP852983.1:35998-34679;PP853000.1:35959-34640;PP853005.1:35967-34648;PP853007.1:36000-34681;PP853013.1:35973-34654;PP853027.1:36004-34685;PP853045.1:35985-34666;PP853048.1:36004-34685;PP860026.1:36002-34683;PP860027.1:36027-34708;PP860028.1:35968-34649;PP860031.1:36021-34702;PP860033.1:36026-34707;PQ153226.1:35992-34673;PQ178862.2:36865-35546;PQ221506.1:31835-30516;PQ221833.1:38004-36685;PQ340477.1:35975-34656;PQ340478.1:35960-34641;PQ340480.1:36039-34720;PQ340483.1:36023-34704;PQ340485.1:36009-34690;PQ340486.1:36024-34705;PQ340487.1:36049-34730;PQ340489.1:36024-34705;PQ340490.1:35975-34656;PQ340491.1:36033-34714;PQ340493.1:36009-34690;PQ340495.1:36039-34720;PQ340496.1:36072-34753;PQ368489.1:35995-34676;PQ451835.1:36083-34764;PQ451836.1:36015-34696;PQ451838.1:36066-34747;PQ451841.1:36026-34707;PQ451842.1:35978-34659 |
| OG0000151 | AY603973.1:35939-35745;AY741551.1:35943-35749;AY753185.1:36182-35988;DQ011154.1:36159-35965;DQ011155.1:36140-35946;EPI_ISL_13331598:34010-33816;EPI_ISL_13363142:34003-33809;EPI_ISL_13374487:34018-33824;EPI_ISL_13502582:34003-33809;EPI_ISL_13508471:34095-33901;EPI_ISL_13607904:33994-33800;EPI_ISL_13624509:33938-33744;EPI_ISL_13889442:34113-33919;EPI_ISL_13953610:33961-33767;EPI_ISL_14181948:34139-33945;EPI_ISL_14838587:36013-35819;EPI_ISL_15263355:33994-33800;EPI_ISL_15269699:33963-33769;EPI_ISL_15293002:34236-34042;EPI_ISL_15641561:33952-33758;EPI_ISL_15831211:34001-33807;EPI_ISL_15992098:34016-33822;EPI_ISL_16233784:34012-33818;EPI_ISL_16946400:34001-33807;EPI_ISL_17048206:33651-33457;EPI_ISL_17703567:34016-33822;EPI_ISL_17718458:33994-33800;EPI_ISL_18213374:33991-33797;EPI_ISL_18360394:33652-33458;EPI_ISL_18557816:33992-33798;EPI_ISL_18642365:33994-33800;EPI_ISL_18659846:33964-33770;EPI_ISL_18744049:33996-33802;EPI_ISL_19022858:33991-33797;EPI_ISL_19028778:33908-33714;EPI_ISL_19151800:33992-33798;EPI_ISL_19159116:33994-33800;EPI_ISL_19350788:34887-34693;EPI_ISL_19359677:33991-33797;EPI_ISL_19446306:33986-33792;EPI_ISL_19467692:36292-36098;JX878411.1:37007-36813;JX878425.1:36521-36327;JX878426.1:35966-35772;KC257460.1:35937-35743;KJ642612.1:34252-34058;KJ642613.1:35828-35634;KJ642616.1:35633-35439;KJ642618.1:33420-33226;KP849469.1:36329-36135;KP849471.1:36255-36061;LC831698.1:33992-33798;MN648051.1:34214-34020;MN702445.1:29674-29480;MN702446.1:35820-35626;MN702447.1:29185-28991;MN702449.1:29685-29491;MN702452.1:35858-35664;MT903338.1:34157-33963;MT903340.1:33994-33800;MT903341.1:25527-25333;MT903342.1:34009-33815;MT903347.1:35952-35758;ON568298.1:34140-33946;ON622713.1:34226-34032;ON622722.2:33997-33803;ON631963.1:34017-33823;ON649879.1:33827-33633;ON676707.1:34047-33853;ON755039.1:33657-33463;ON808413.1:33997-33803;ON880420.1:34224-34030;ON911481.2:34014-33820;ON918656.1:34029-33835;ON959133.1:34118-33924;ON959136.1:34119-33925;ON983168.1:32693-32499;OP019276.1:33991-33797;OP150924.1:34132-33938;OP160532.1:33957-33763;OP235282.1:33991-33797;OP289782.1:33994-33800;OP331335.1:34011-33817;OP382479.1:33998-33804;OP422341.1:34011-33817;OP434519.1:33990-33796;OP535324.1:33992-33798;OP535333.1:34101-33907;OP536770.1:33993-33799;OP597769.1:34015-33821;OP604566.1:33994-33800;OP612682.1:33920-33726;OP612684.1:33984-33790;OP612686.1:33984-33790;OP696841.1:31268-31074;OP879722.1:33217-33023;OQ419007.1:33227-33033;OQ419012.1:33219-33025;OQ419031.1:33198-33004;OQ419047.1:33227-33033;OQ451898.1:33079-32885;OQ451900.1:33992-33798;OQ581853.1:34018-33824;OQ581855.1:34099-33905;OQ721951.1:34032-33838;OQ721954.1:33989-33795;OR035652.1:33941-33747;OR035664.1:33941-33747;OR035702.1:33941-33747;OR146272.1:33957-33763;OR146330.1:34002-33808;OR264364.1:34012-33818;OR427332.1:34012-33818;OR459777.1:33996-33802;OR459797.1:34011-33817;OR943698.1:35994-35800;PP098589.2:33993-33799;PP496048.1:33997-33803;PP601206.1:36011-35817;PP776553.1:33918-33724;PP852971.1:33991-33797;PP852983.1:33997-33803;PP853000.1:33958-33764;PP853005.1:33966-33772;PP853007.1:33999-33805;PP853013.1:33972-33778;PP853027.1:34003-33809;PP853045.1:33984-33790;PP853048.1:34003-33809;PP860026.1:34001-33807;PP860027.1:34026-33832;PP860028.1:33967-33773;PP860031.1:34020-33826;PP860033.1:34025-33831;PQ153226.1:33991-33797;PQ178862.2:34864-34670;PQ221506.1:29834-29640;PQ221833.1:36003-35809;PQ340477.1:33974-33780;PQ340478.1:33959-33765;PQ340480.1:34038-33844;PQ340483.1:34022-33828;PQ340485.1:34008-33814;PQ340486.1:34023-33829;PQ340487.1:34048-33854;PQ340489.1:34023-33829;PQ340490.1:33974-33780;PQ340491.1:34032-33838;PQ340493.1:34008-33814;PQ340495.1:34038-33844;PQ340496.1:34071-33877;PQ368489.1:33994-33800;PQ451835.1:34082-33888;PQ451836.1:34014-33820;PQ451838.1:34065-33871;PQ451841.1:34025-33831;PQ451842.1:33977-33783 |
| OG0000152 | AY603973.1:33143-31671;AY741551.1:33147-31669;AY753185.1:33386-31914;DQ011154.1:33363-31885;DQ011155.1:33344-31887;EPI_ISL_13331598:31209-29761;EPI_ISL_13363142:31202-29754;EPI_ISL_13374487:31217-29769;EPI_ISL_13502582:31202-29754;EPI_ISL_13508471:31294-29846;EPI_ISL_13607904:31193-29745;EPI_ISL_13624509:31137-29689;EPI_ISL_13889442:31312-29864;EPI_ISL_13953610:31160-29712;EPI_ISL_14181948:31338-29890;EPI_ISL_14838587:33217-31739;EPI_ISL_15263355:31193-29745;EPI_ISL_15269699:31162-29714;EPI_ISL_15293002:31435-29987;EPI_ISL_15641561:31151-29703;EPI_ISL_15831211:31200-29752;EPI_ISL_15992098:31215-29767;EPI_ISL_16233784:31211-29763;EPI_ISL_16946400:31200-29752;EPI_ISL_17048206:30850-29402;EPI_ISL_17703567:31215-29767;EPI_ISL_17718458:31193-29745;EPI_ISL_18213374:31190-29742;EPI_ISL_18360394:30851-29403;EPI_ISL_18557816:31191-29743;EPI_ISL_18642365:31193-29745;EPI_ISL_18659846:31163-29715;EPI_ISL_18744049:31195-29747;EPI_ISL_19022858:31190-29742;EPI_ISL_19028778:31107-29659;EPI_ISL_19151800:31191-29743;EPI_ISL_19159116:31193-29745;EPI_ISL_19350788:32091-30634;EPI_ISL_19359677:31190-29742;EPI_ISL_19446306:31185-29737;EPI_ISL_19467692:33496-32018;JX878411.1:34211-32733;JX878425.1:33725-32247;JX878426.1:33170-31707;KC257460.1:33141-31684;KJ642612.1:31456-29993;KJ642613.1:33032-31554;KJ642616.1:32837-31374;KJ642618.1:30624-29161;KP849469.1:33533-32055;KP849471.1:33459-31996;LC831698.1:31191-29743;MN648051.1:31413-29965;MN702445.1:26878-25400;MN702446.1:33024-31546;MN702447.1:26389-24911;MN702449.1:26889-25411;MN702452.1:33062-31584;MT903338.1:31356-29908;MT903340.1:31193-29745;MT903341.1:22726-21278;MT903342.1:31208-29760;MT903347.1:33156-31669;ON568298.1:31339-29891;ON622713.1:31425-29977;ON622722.2:31196-29748;ON631963.1:31216-29768;ON649879.1:31026-29578;ON676707.1:31246-29798;ON755039.1:30856-29408;ON808413.1:31196-29748;ON880420.1:31423-29975;ON911481.2:31213-29765;ON918656.1:31228-29780;ON959133.1:31317-29869;ON959136.1:31318-29870;ON983168.1:29892-28444;OP019276.1:31190-29742;OP150924.1:31331-29883;OP160532.1:31156-29708;OP235282.1:31190-29742;OP289782.1:31193-29745;OP331335.1:31210-29762;OP382479.1:31197-29749;OP422341.1:31210-29762;OP434519.1:31189-29741;OP535324.1:31191-29743;OP535333.1:31300-29852;OP536770.1:31192-29744;OP597769.1:31214-29766;OP604566.1:31193-29745;OP612682.1:31119-29671;OP612684.1:31183-29735;OP612686.1:31183-29735;OP696841.1:28467-27019;OP879722.1:30416-28968;OQ419007.1:30426-28978;OQ419012.1:30418-28970;OQ419031.1:30397-28949;OQ419047.1:30426-28978;OQ451898.1:30278-28830;OQ451900.1:31191-29743;OQ581853.1:31217-29769;OQ581855.1:31298-29850;OQ721951.1:31231-29783;OQ721954.1:31188-29740;OR035652.1:31140-29692;OR035664.1:31140-29692;OR035702.1:31140-29692;OR146272.1:31156-29708;OR146330.1:31201-29753;OR264364.1:31211-29763;OR427332.1:31211-29763;OR459777.1:31195-29747;OR459797.1:31210-29762;OR943698.1:33198-31735;PP098589.2:31192-29744;PP496048.1:31196-29748;PP601206.1:33215-31737;PP776553.1:31117-29669;PP852971.1:31190-29742;PP852983.1:31195-29747;PP853000.1:31157-29709;PP853005.1:31165-29717;PP853007.1:31197-29749;PP853013.1:31171-29723;PP853027.1:31202-29754;PP853045.1:31183-29735;PP853048.1:31202-29754;PP860026.1:31203-29746;PP860027.1:31228-29756;PP860028.1:31169-29712;PP860031.1:31223-29766;PP860033.1:31227-29755;PQ153226.1:31190-29742;PQ178862.2:32068-30611;PQ221506.1:27038-25575;PQ221833.1:33207-31729;PQ340477.1:31173-29725;PQ340478.1:31158-29710;PQ340480.1:31237-29789;PQ340483.1:31221-29773;PQ340485.1:31207-29759;PQ340486.1:31222-29774;PQ340487.1:31247-29799;PQ340489.1:31222-29774;PQ340490.1:31173-29725;PQ340491.1:31231-29783;PQ340493.1:31207-29759;PQ340495.1:31237-29789;PQ340496.1:31270-29822;PQ368489.1:31193-29745;PQ451835.1:31281-29833;PQ451836.1:31213-29765;PQ451838.1:31264-29816;PQ451841.1:31224-29776;PQ451842.1:31176-29728 |
| OG0000153 | AY603973.1:31658-31203;AY741551.1:31672-31217;AY753185.1:31901-31446;DQ011154.1:31888-31433;DQ011155.1:31874-31419;EPI_ISL_13331598:29744-29289;EPI_ISL_13363142:29737-29282;EPI_ISL_13374487:29752-29297;EPI_ISL_13502582:29737-29282;EPI_ISL_13508471:29829-29374;EPI_ISL_13607904:29728-29273;EPI_ISL_13624509:29672-29217;EPI_ISL_13889442:29847-29392;EPI_ISL_13953610:29695-29240;EPI_ISL_14181948:29873-29418;EPI_ISL_14838587:31742-31287;EPI_ISL_15263355:29728-29273;EPI_ISL_15269699:29697-29242;EPI_ISL_15293002:29970-29515;EPI_ISL_15641561:29686-29231;EPI_ISL_15831211:29735-29280;EPI_ISL_15992098:29750-29295;EPI_ISL_16233784:29746-29291;EPI_ISL_16946400:29735-29280;EPI_ISL_17048206:29385-28930;EPI_ISL_17703567:29750-29295;EPI_ISL_17718458:29728-29273;EPI_ISL_18213374:29725-29270;EPI_ISL_18360394:29386-28931;EPI_ISL_18557816:29726-29271;EPI_ISL_18642365:29728-29273;EPI_ISL_18659846:29698-29243;EPI_ISL_18744049:29730-29275;EPI_ISL_19022858:29725-29270;EPI_ISL_19028778:29642-29187;EPI_ISL_19151800:29726-29271;EPI_ISL_19159116:29728-29273;EPI_ISL_19350788:30621-30166;EPI_ISL_19359677:29725-29270;EPI_ISL_19446306:29720-29265;EPI_ISL_19467692:32021-31566;JX878411.1:32736-32281;JX878425.1:32250-31795;JX878426.1:31690-31235;KC257460.1:31671-31216;KJ642612.1:29976-29521;KJ642613.1:31557-31102;KJ642616.1:31357-30902;KJ642618.1:29144-28689;KP849469.1:32058-31603;KP849471.1:31979-31524;LC831698.1:29726-29271;MN648051.1:29948-29493;MN702445.1:25403-24948;MN702446.1:31549-31094;MN702447.1:24914-24459;MN702449.1:25414-24959;MN702452.1:31587-31132;MT903338.1:29891-29436;MT903340.1:29728-29273;MT903341.1:21261-20806;MT903342.1:29743-29288;MT903347.1:31656-31201;ON568298.1:29874-29419;ON622713.1:29960-29505;ON622722.2:29731-29276;ON631963.1:29751-29296;ON649879.1:29561-29106;ON676707.1:29781-29326;ON755039.1:29391-28936;ON808413.1:29731-29276;ON880420.1:29958-29503;ON911481.2:29748-29293;ON918656.1:29763-29308;ON959133.1:29852-29397;ON959136.1:29853-29398;ON983168.1:28427-27972;OP019276.1:29725-29270;OP150924.1:29866-29411;OP160532.1:29691-29236;OP235282.1:29725-29270;OP289782.1:29728-29273;OP331335.1:29745-29290;OP382479.1:29732-29277;OP422341.1:29745-29290;OP434519.1:29724-29269;OP535324.1:29726-29271;OP535333.1:29835-29380;OP536770.1:29727-29272;OP597769.1:29749-29294;OP604566.1:29728-29273;OP612682.1:29654-29199;OP612684.1:29718-29263;OP612686.1:29718-29263;OP696841.1:27002-26547;OP879722.1:28951-28496;OQ419007.1:28961-28506;OQ419012.1:28953-28498;OQ419031.1:28932-28477;OQ419047.1:28961-28506;OQ451898.1:28813-28358;OQ451900.1:29726-29271;OQ581853.1:29752-29297;OQ581855.1:29833-29378;OQ721951.1:29766-29311;OQ721954.1:29723-29268;OR035652.1:29675-29220;OR035664.1:29675-29220;OR035702.1:29675-29220;OR146272.1:29691-29236;OR146330.1:29736-29281;OR264364.1:29746-29291;OR427332.1:29746-29291;OR459777.1:29730-29275;OR459797.1:29745-29290;OR943698.1:31718-31263;PP098589.2:29727-29272;PP496048.1:29731-29276;PP601206.1:31740-31285;PP776553.1:29652-29197;PP852971.1:29725-29270;PP852983.1:29730-29275;PP853000.1:29692-29237;PP853005.1:29700-29245;PP853007.1:29732-29277;PP853013.1:29706-29251;PP853027.1:29737-29282;PP853045.1:29718-29263;PP853048.1:29737-29282;PP860026.1:29733-29278;PP860027.1:29743-29288;PP860028.1:29699-29244;PP860031.1:29753-29298;PP860033.1:29742-29287;PQ153226.1:29725-29270;PQ178862.2:30598-30143;PQ221506.1:25558-25103;PQ221833.1:31732-31277;PQ340477.1:29708-29253;PQ340478.1:29693-29238;PQ340480.1:29772-29317;PQ340483.1:29756-29301;PQ340485.1:29742-29287;PQ340486.1:29757-29302;PQ340487.1:29782-29327;PQ340489.1:29757-29302;PQ340490.1:29708-29253;PQ340491.1:29766-29311;PQ340493.1:29742-29287;PQ340495.1:29772-29317;PQ340496.1:29805-29350;PQ368489.1:29728-29273;PQ451835.1:29816-29361;PQ451836.1:29748-29293;PQ451838.1:29799-29344;PQ451841.1:29759-29304;PQ451842.1:29711-29256 |
| OG0000154 | AY603973.1:27407-26280;AY741551.1:27421-26297;AY753185.1:27650-26523;DQ011154.1:27653-26526;DQ011155.1:27639-26512;EPI_ISL_13331598:25493-24366;EPI_ISL_13363142:25486-24359;EPI_ISL_13374487:25501-24374;EPI_ISL_13502582:25486-24359;EPI_ISL_13508471:25578-24451;EPI_ISL_13607904:25477-24350;EPI_ISL_13624509:25421-24294;EPI_ISL_13889442:25596-24469;EPI_ISL_13953610:25444-24317;EPI_ISL_14181948:25622-24495;EPI_ISL_14838587:27507-26380;EPI_ISL_15263355:25477-24350;EPI_ISL_15269699:25446-24319;EPI_ISL_15293002:25719-24592;EPI_ISL_15641561:25435-24308;EPI_ISL_15831211:25484-24357;EPI_ISL_15992098:25499-24372;EPI_ISL_16233784:25495-24368;EPI_ISL_16946400:25484-24357;EPI_ISL_17048206:25134-24007;EPI_ISL_17703567:25499-24372;EPI_ISL_17718458:25477-24350;EPI_ISL_18213374:25474-24347;EPI_ISL_18360394:25135-24008;EPI_ISL_18557816:25475-24348;EPI_ISL_18642365:25477-24350;EPI_ISL_18659846:25447-24320;EPI_ISL_18744049:25479-24352;EPI_ISL_19022858:25474-24347;EPI_ISL_19028778:25391-24264;EPI_ISL_19151800:25475-24348;EPI_ISL_19159116:25477-24350;EPI_ISL_19350788:26392-25265;EPI_ISL_19359677:25474-24347;EPI_ISL_19446306:25469-24342;EPI_ISL_19467692:27786-26659;JX878411.1:28501-27374;JX878425.1:28015-26888;JX878426.1:27455-26328;KC257460.1:27436-26309;KJ642612.1:25741-24614;KJ642613.1:27322-26195;KJ642616.1:27106-25979;KJ642618.1:24909-23782;KP849469.1:27823-26696;KP849471.1:27744-26617;LC831698.1:25475-24348;MN648051.1:25697-24570;MN702445.1:21168-20041;MN702446.1:27314-26187;MN702447.1:20679-19552;MN702449.1:21179-20052;MN702452.1:27352-26225;MT903338.1:25640-24513;MT903340.1:25477-24350;MT903341.1:17010-15883;MT903342.1:25492-24365;MT903347.1:27405-26278;ON568298.1:25623-24496;ON622713.1:25709-24582;ON622722.2:25480-24353;ON631963.1:25500-24373;ON649879.1:25310-24183;ON676707.1:25530-24403;ON755039.1:25140-24013;ON808413.1:25480-24353;ON880420.1:25707-24580;ON911481.2:25497-24370;ON918656.1:25512-24385;ON959133.1:25601-24474;ON959136.1:25602-24475;ON983168.1:24176-23049;OP019276.1:25474-24347;OP150924.1:25615-24488;OP160532.1:25440-24313;OP235282.1:25474-24347;OP289782.1:25477-24350;OP331335.1:25494-24367;OP382479.1:25481-24354;OP422341.1:25494-24367;OP434519.1:25473-24346;OP535324.1:25475-24348;OP535333.1:25584-24457;OP536770.1:25476-24349;OP597769.1:25498-24371;OP604566.1:25477-24350;OP612682.1:25403-24276;OP612684.1:25467-24340;OP612686.1:25467-24340;OP696841.1:22751-21624;OP879722.1:24700-23573;OQ419007.1:24710-23583;OQ419012.1:24702-23575;OQ419031.1:24681-23554;OQ419047.1:24710-23583;OQ451898.1:24562-23435;OQ451900.1:25475-24348;OQ581853.1:25501-24374;OQ581855.1:25582-24455;OQ721951.1:25515-24388;OQ721954.1:25472-24345;OR035652.1:25424-24297;OR035664.1:25424-24297;OR035702.1:25424-24297;OR146272.1:25440-24313;OR146330.1:25485-24358;OR264364.1:25495-24368;OR427332.1:25495-24368;OR459777.1:25479-24352;OR459797.1:25494-24367;OR943698.1:27483-26356;PP098589.2:25476-24349;PP496048.1:25480-24353;PP601206.1:27505-26378;PP776553.1:25401-24247;PP852971.1:25474-24347;PP852983.1:25479-24352;PP853000.1:25441-24314;PP853005.1:25449-24322;PP853007.1:25481-24354;PP853013.1:25455-24328;PP853027.1:25486-24359;PP853045.1:25467-24340;PP853048.1:25486-24359;PP860026.1:25481-24354;PP860027.1:25492-24365;PP860028.1:25448-24321;PP860031.1:25502-24375;PP860033.1:25491-24364;PQ153226.1:25474-24347;PQ178862.2:26369-25242;PQ221506.1:21325-20198;PQ221833.1:27497-26370;PQ340477.1:25457-24330;PQ340478.1:25442-24315;PQ340480.1:25521-24394;PQ340483.1:25505-24378;PQ340485.1:25491-24364;PQ340486.1:25506-24379;PQ340487.1:25531-24404;PQ340489.1:25506-24379;PQ340490.1:25457-24330;PQ340491.1:25515-24388;PQ340493.1:25491-24364;PQ340495.1:25521-24394;PQ340496.1:25554-24427;PQ368489.1:25477-24350;PQ451835.1:25565-24438;PQ451836.1:25497-24370;PQ451838.1:25548-24421;PQ451841.1:25508-24381;PQ451842.1:25460-24333 |
| OG0000155 | AY603973.1:25817-24963;AY741551.1:25678-24824;AY753185.1:26060-25206;DQ011154.1:26295-25441;DQ011155.1:26281-25427;EPI_ISL_13331598:24133-23279;EPI_ISL_13363142:24126-23272;EPI_ISL_13374487:24141-23287;EPI_ISL_13502582:24126-23272;EPI_ISL_13508471:24218-23364;EPI_ISL_13607904:24117-23263;EPI_ISL_13624509:24061-23207;EPI_ISL_13889442:24236-23382;EPI_ISL_13953610:24084-23230;EPI_ISL_14181948:24262-23408;EPI_ISL_14838587:26149-25295;EPI_ISL_15263355:24117-23263;EPI_ISL_15269699:24086-23232;EPI_ISL_15293002:24359-23505;EPI_ISL_15641561:24075-23221;EPI_ISL_15831211:24124-23270;EPI_ISL_15992098:24139-23285;EPI_ISL_16233784:24135-23281;EPI_ISL_16946400:24124-23270;EPI_ISL_17048206:23774-22920;EPI_ISL_17703567:24139-23285;EPI_ISL_17718458:24117-23263;EPI_ISL_18213374:24114-23260;EPI_ISL_18360394:23775-22921;EPI_ISL_18557816:24115-23261;EPI_ISL_18642365:24117-23263;EPI_ISL_18659846:24087-23233;EPI_ISL_18744049:24119-23265;EPI_ISL_19022858:24114-23260;EPI_ISL_19028778:24031-23177;EPI_ISL_19151800:24115-23261;EPI_ISL_19159116:24117-23263;EPI_ISL_19350788:25034-24180;EPI_ISL_19359677:24114-23260;EPI_ISL_19446306:24109-23255;EPI_ISL_19467692:26428-25574;JX878411.1:27143-26289;JX878425.1:26657-25803;JX878426.1:26097-25243;KC257460.1:26078-25224;KJ642612.1:24383-23529;KJ642613.1:25964-25110;KJ642616.1:25633-24779;KJ642618.1:23551-22697;KP849469.1:26465-25611;KP849471.1:26386-25532;LC831698.1:24115-23261;MN648051.1:24337-23483;MN702445.1:19810-18956;MN702446.1:25956-25102;MN702447.1:19321-18467;MN702449.1:19821-18967;MN702452.1:25994-25140;MT903338.1:24280-23426;MT903340.1:24117-23263;MT903341.1:15650-14796;MT903342.1:24132-23278;MT903347.1:26010-25156;ON568298.1:24263-23409;ON622713.1:24349-23495;ON622722.2:24120-23266;ON631963.1:24140-23286;ON649879.1:23950-23096;ON676707.1:24170-23316;ON755039.1:23780-22926;ON808413.1:24120-23266;ON880420.1:24347-23493;ON911481.2:24137-23283;ON918656.1:24152-23298;ON959133.1:24241-23387;ON959136.1:24242-23388;ON983168.1:22816-21962;OP019276.1:24114-23260;OP150924.1:24255-23401;OP160532.1:24080-23226;OP235282.1:24114-23260;OP289782.1:24117-23263;OP331335.1:24134-23280;OP382479.1:24121-23267;OP422341.1:24134-23280;OP434519.1:24113-23259;OP535324.1:24115-23261;OP535333.1:24224-23370;OP536770.1:24116-23262;OP597769.1:24138-23284;OP604566.1:24117-23263;OP612682.1:24043-23189;OP612684.1:24107-23253;OP612686.1:24107-23253;OP696841.1:21391-20537;OP879722.1:23340-22486;OQ419007.1:23350-22496;OQ419012.1:23342-22488;OQ419031.1:22939-22493;OQ419047.1:23350-22496;OQ451898.1:23202-22348;OQ451900.1:24115-23261;OQ581853.1:24141-23287;OQ581855.1:24222-23368;OQ721951.1:24155-23301;OQ721954.1:24112-23258;OR035652.1:24064-23210;OR035664.1:24064-23210;OR035702.1:24064-23210;OR146272.1:24080-23226;OR146330.1:24125-23271;OR264364.1:24135-23281;OR427332.1:24135-23281;OR459777.1:24119-23265;OR459797.1:24134-23280;OR943698.1:26125-25271;PP098589.2:24116-23262;PP496048.1:24120-23266;PP601206.1:26147-25293;PP776553.1:24065-23211;PP852971.1:24114-23260;PP852983.1:24119-23265;PP853000.1:24081-23227;PP853005.1:24089-23235;PP853007.1:24121-23267;PP853013.1:24095-23241;PP853027.1:24126-23272;PP853045.1:24107-23253;PP853048.1:24126-23272;PP860026.1:24121-23267;PP860027.1:24132-23278;PP860028.1:24088-23234;PP860031.1:24142-23288;PP860033.1:24131-23277;PQ153226.1:24114-23260;PQ178862.2:25011-24157;PQ221506.1:19967-19113;PQ221833.1:26139-25285;PQ340477.1:24097-23243;PQ340478.1:24082-23228;PQ340480.1:24161-23307;PQ340483.1:24145-23291;PQ340485.1:24131-23277;PQ340486.1:24146-23292;PQ340487.1:24171-23317;PQ340489.1:24146-23292;PQ340490.1:24097-23243;PQ340491.1:24155-23301;PQ340493.1:24131-23277;PQ340495.1:24161-23307;PQ340496.1:24194-23340;PQ368489.1:24117-23263;PQ451835.1:24205-23351;PQ451836.1:24137-23283;PQ451838.1:24188-23334;PQ451841.1:24148-23294;PQ451842.1:24100-23246 |
| OG0000156 | AY603973.1:22757-22224;AY741551.1:22619-22086;AY753185.1:23000-22467;DQ011154.1:23210-22677;DQ011155.1:23190-22657;EPI_ISL_13331598:21079-20546;EPI_ISL_13363142:21072-20539;EPI_ISL_13374487:21087-20554;EPI_ISL_13502582:21072-20539;EPI_ISL_13508471:21164-20631;EPI_ISL_13607904:21063-20530;EPI_ISL_13624509:21007-20474;EPI_ISL_13889442:21182-20649;EPI_ISL_13953610:21030-20497;EPI_ISL_14181948:21208-20675;EPI_ISL_14838587:23064-22531;EPI_ISL_15263355:21063-20530;EPI_ISL_15269699:21032-20499;EPI_ISL_15293002:21305-20772;EPI_ISL_15641561:21021-20488;EPI_ISL_15831211:21070-20537;EPI_ISL_15992098:21085-20552;EPI_ISL_16233784:21081-20548;EPI_ISL_16946400:21070-20537;EPI_ISL_17048206:20720-20187;EPI_ISL_17703567:21085-20552;EPI_ISL_17718458:21063-20530;EPI_ISL_18213374:21045-20527;EPI_ISL_18360394:20721-20188;EPI_ISL_18557816:21061-20528;EPI_ISL_18642365:21063-20530;EPI_ISL_18659846:21018-20500;EPI_ISL_18744049:21065-20532;EPI_ISL_19022858:21060-20527;EPI_ISL_19028778:20977-20444;EPI_ISL_19151800:21061-20528;EPI_ISL_19159116:21063-20530;EPI_ISL_19350788:21944-21411;EPI_ISL_19359677:21045-20527;EPI_ISL_19446306:21055-20522;EPI_ISL_19467692:23343-22810;JX878411.1:24058-23525;JX878425.1:23571-23038;JX878426.1:23013-22480;KC257460.1:22993-22460;KJ642612.1:21298-20765;KJ642613.1:22879-22346;KJ642616.1:22573-22040;KJ642618.1:20470-19937;KP849469.1:23380-22847;KP849471.1:23301-22768;LC831698.1:21061-20528;MN648051.1:21283-20750;MN702445.1:16726-16193;MN702446.1:22872-22339;MN702447.1:16232-15699;MN702449.1:16737-16204;MN702452.1:22909-22376;MT903338.1:21226-20693;MT903340.1:21063-20530;MT903341.1:12596-12063;MT903342.1:21078-20545;MT903347.1:22951-22418;ON568298.1:21209-20676;ON622713.1:21295-20762;ON622722.2:21066-20533;ON631963.1:21086-20553;ON649879.1:20896-20363;ON676707.1:21116-20583;ON755039.1:20726-20193;ON808413.1:21066-20533;ON880420.1:21293-20760;ON911481.2:21083-20550;ON918656.1:21098-20565;ON959133.1:21187-20654;ON959136.1:21188-20655;ON983168.1:19762-19229;OP019276.1:21060-20527;OP150924.1:21201-20668;OP160532.1:21026-20493;OP235282.1:21060-20527;OP289782.1:21063-20530;OP331335.1:21080-20547;OP382479.1:21067-20534;OP422341.1:21080-20547;OP434519.1:21059-20526;OP535324.1:21061-20528;OP535333.1:21170-20637;OP536770.1:21062-20529;OP597769.1:21084-20551;OP604566.1:21063-20530;OP612682.1:20989-20456;OP612684.1:21053-20520;OP612686.1:21053-20520;OP696841.1:18337-17804;OP879722.1:20286-19753;OQ419007.1:20296-19763;OQ419012.1:20288-19755;OQ419031.1:20293-19760;OQ419047.1:20296-19763;OQ451898.1:20148-19615;OQ451900.1:21061-20528;OQ581853.1:21087-20554;OQ581855.1:21168-20635;OQ721951.1:21101-20568;OQ721954.1:21058-20525;OR035652.1:21010-20477;OR035664.1:21010-20477;OR035702.1:21010-20477;OR146272.1:21026-20493;OR146330.1:21071-20538;OR264364.1:21081-20548;OR427332.1:21081-20548;OR459777.1:21065-20532;OR459797.1:21080-20547;OR943698.1:23047-22514;PP098589.2:21062-20529;PP496048.1:21066-20533;PP601206.1:23062-22529;PP776553.1:20996-20478;PP852971.1:21060-20527;PP852983.1:21065-20532;PP853000.1:21027-20494;PP853005.1:21035-20502;PP853007.1:21067-20534;PP853013.1:21041-20508;PP853027.1:21072-20539;PP853045.1:21053-20520;PP853048.1:21072-20539;PP860026.1:21067-20534;PP860027.1:21078-20545;PP860028.1:21034-20501;PP860031.1:21088-20555;PP860033.1:21077-20544;PQ153226.1:21060-20527;PQ178862.2:21921-21388;PQ221506.1:16883-16350;PQ221833.1:23054-22521;PQ340477.1:21043-20510;PQ340478.1:21028-20495;PQ340480.1:21107-20574;PQ340483.1:21091-20558;PQ340485.1:21077-20544;PQ340486.1:21092-20559;PQ340487.1:21117-20584;PQ340489.1:21092-20559;PQ340490.1:21043-20510;PQ340491.1:21101-20568;PQ340493.1:21077-20544;PQ340495.1:21107-20574;PQ340496.1:21140-20607;PQ368489.1:21063-20530;PQ451835.1:21151-20618;PQ451836.1:21083-20550;PQ451838.1:21134-20601;PQ451841.1:21094-20561;PQ451842.1:21046-20513 |
| OG0000157 | AY603973.1:22097-21744;AY741551.1:21959-21606;AY753185.1:22340-21987;DQ011154.1:22550-22197;DQ011155.1:22530-22177;EPI_ISL_13331598:20419-20066;EPI_ISL_13363142:20412-20059;EPI_ISL_13374487:20427-20074;EPI_ISL_13502582:20412-20059;EPI_ISL_13508471:20504-20151;EPI_ISL_13607904:20403-20050;EPI_ISL_13624509:20347-19994;EPI_ISL_13889442:20522-20169;EPI_ISL_13953610:20370-20017;EPI_ISL_14181948:20548-20195;EPI_ISL_14838587:22404-22051;EPI_ISL_15263355:20403-20050;EPI_ISL_15269699:20372-20019;EPI_ISL_15293002:20645-20292;EPI_ISL_15641561:20361-20008;EPI_ISL_15831211:20410-20057;EPI_ISL_15992098:20425-20072;EPI_ISL_16233784:20421-20068;EPI_ISL_16946400:20410-20057;EPI_ISL_17048206:20060-19707;EPI_ISL_17703567:20425-20072;EPI_ISL_17718458:20403-20050;EPI_ISL_18213374:20400-20047;EPI_ISL_18360394:20061-19708;EPI_ISL_18557816:20401-20048;EPI_ISL_18642365:20403-20050;EPI_ISL_18659846:20373-20020;EPI_ISL_18744049:20405-20052;EPI_ISL_19022858:20400-20047;EPI_ISL_19028778:20317-19964;EPI_ISL_19151800:20401-20048;EPI_ISL_19159116:20403-20050;EPI_ISL_19350788:21284-20931;EPI_ISL_19359677:20400-20047;EPI_ISL_19446306:20395-20042;EPI_ISL_19467692:22683-22330;JX878411.1:23398-23045;JX878425.1:22910-22557;JX878426.1:22353-22000;KC257460.1:22333-21980;KJ642612.1:20638-20285;KJ642613.1:22219-21866;KJ642616.1:21913-21560;KJ642618.1:19810-19457;KP849469.1:22720-22367;KP849471.1:22641-22288;LC831698.1:20401-20048;MN648051.1:20623-20270;MN702445.1:16066-15713;MN702446.1:22212-21859;MN702447.1:15572-15219;MN702449.1:16077-15724;MN702452.1:22249-21896;MT903338.1:20566-20213;MT903340.1:20403-20050;MT903341.1:11936-11583;MT903342.1:20418-20065;MT903347.1:22291-21938;ON568298.1:20549-20196;ON622713.1:20635-20282;ON622722.2:20406-20053;ON631963.1:20426-20073;ON649879.1:20236-19883;ON676707.1:20456-20103;ON755039.1:20066-19713;ON808413.1:20406-20053;ON880420.1:20633-20280;ON911481.2:20423-20070;ON918656.1:20438-20085;ON959133.1:20527-20174;ON959136.1:20528-20175;ON983168.1:19102-18749;OP019276.1:20400-20047;OP150924.1:20541-20188;OP160532.1:20366-20013;OP235282.1:20400-20047;OP289782.1:20403-20050;OP331335.1:20420-20067;OP382479.1:20407-20054;OP422341.1:20420-20067;OP434519.1:20399-20046;OP535324.1:20401-20048;OP535333.1:20510-20157;OP536770.1:20402-20049;OP597769.1:20424-20071;OP604566.1:20403-20050;OP612682.1:20329-19976;OP612684.1:20393-20040;OP612686.1:20393-20040;OP696841.1:17677-17324;OP879722.1:19626-19273;OQ419007.1:19636-19283;OQ419012.1:19628-19275;OQ419031.1:19633-19280;OQ419047.1:19636-19283;OQ451898.1:19488-19135;OQ451900.1:20401-20048;OQ581853.1:20427-20074;OQ581855.1:20508-20155;OQ721951.1:20441-20088;OQ721954.1:20398-20045;OR035652.1:20350-19997;OR035664.1:20350-19997;OR035702.1:20350-19997;OR146272.1:20366-20013;OR146330.1:20411-20058;OR264364.1:20421-20068;OR427332.1:20421-20068;OR459777.1:20405-20052;OR459797.1:20420-20067;OR943698.1:22387-22034;PP098589.2:20402-20049;PP496048.1:20406-20053;PP601206.1:22402-22049;PP776553.1:20351-19998;PP852971.1:20400-20047;PP852983.1:20405-20052;PP853000.1:20367-20014;PP853005.1:20375-20022;PP853007.1:20407-20054;PP853013.1:20381-20028;PP853027.1:20412-20059;PP853045.1:20393-20040;PP853048.1:20412-20059;PP860026.1:20407-20054;PP860027.1:20418-20065;PP860028.1:20374-20021;PP860031.1:20428-20075;PP860033.1:20417-20064;PQ153226.1:20400-20047;PQ178862.2:21261-20908;PQ221506.1:16223-15870;PQ221833.1:22394-22041;PQ340477.1:20383-20030;PQ340478.1:20368-20015;PQ340480.1:20447-20094;PQ340483.1:20431-20078;PQ340485.1:20417-20064;PQ340486.1:20432-20079;PQ340487.1:20457-20104;PQ340489.1:20432-20079;PQ340490.1:20383-20030;PQ340491.1:20441-20088;PQ340493.1:20417-20064;PQ340495.1:20447-20094;PQ340496.1:20480-20127;PQ368489.1:20403-20050;PQ451835.1:20491-20138;PQ451836.1:20423-20070;PQ451838.1:20474-20121;PQ451841.1:20434-20081;PQ451842.1:20386-20033 |
| OG0000158 | AY603973.1:21698-21054;AY741551.1:21560-20916;AY753185.1:21941-21297;DQ011154.1:22151-21507;DQ011155.1:22131-21487;EPI_ISL_13331598:20020-19376;EPI_ISL_13363142:20013-19369;EPI_ISL_13374487:20028-19384;EPI_ISL_13502582:20013-19369;EPI_ISL_13508471:20105-19461;EPI_ISL_13607904:20004-19360;EPI_ISL_13624509:19948-19304;EPI_ISL_13889442:20123-19479;EPI_ISL_13953610:19971-19330;EPI_ISL_14181948:20149-19505;EPI_ISL_14838587:22005-21361;EPI_ISL_15263355:20004-19360;EPI_ISL_15269699:19973-19329;EPI_ISL_15293002:20246-19602;EPI_ISL_15641561:19962-19318;EPI_ISL_15831211:20011-19367;EPI_ISL_15992098:20026-19382;EPI_ISL_16233784:20022-19378;EPI_ISL_16946400:20011-19367;EPI_ISL_17048206:19661-19017;EPI_ISL_17703567:20026-19382;EPI_ISL_17718458:20004-19360;EPI_ISL_18213374:20001-19357;EPI_ISL_18360394:19662-19018;EPI_ISL_18557816:20002-19358;EPI_ISL_18642365:20004-19360;EPI_ISL_18659846:19974-19330;EPI_ISL_18744049:20006-19362;EPI_ISL_19022858:20001-19357;EPI_ISL_19028778:19918-19274;EPI_ISL_19151800:20002-19358;EPI_ISL_19159116:20004-19360;EPI_ISL_19350788:20885-20241;EPI_ISL_19359677:20001-19357;EPI_ISL_19446306:19996-19352;EPI_ISL_19467692:22284-21640;JX878411.1:22999-22355;JX878425.1:22511-21867;JX878426.1:21954-21310;KC257460.1:21934-21290;KJ642612.1:20239-19595;KJ642613.1:21820-21176;KJ642616.1:21514-20870;KJ642618.1:19411-18767;KP849469.1:22321-21677;KP849471.1:22242-21598;LC831698.1:20002-19358;MN648051.1:20224-19580;MN702445.1:15664-15020;MN702446.1:21810-21166;MN702447.1:15173-14529;MN702449.1:15675-15031;MN702452.1:21850-21206;MT903338.1:20167-19523;MT903340.1:20004-19360;MT903341.1:11537-10893;MT903342.1:20019-19375;MT903347.1:21892-21248;ON568298.1:20150-19506;ON622713.1:20236-19592;ON622722.2:20007-19363;ON631963.1:20027-19383;ON649879.1:19837-19193;ON676707.1:20057-19413;ON755039.1:19667-19023;ON808413.1:20007-19363;ON880420.1:20234-19590;ON911481.2:20024-19380;ON918656.1:20039-19395;ON959133.1:20128-19484;ON959136.1:20129-19485;ON983168.1:18703-18059;OP019276.1:20001-19357;OP150924.1:20142-19498;OP160532.1:19967-19323;OP235282.1:20001-19357;OP289782.1:20004-19360;OP331335.1:20021-19377;OP382479.1:20008-19364;OP422341.1:20021-19377;OP434519.1:20000-19356;OP535324.1:20002-19358;OP535333.1:20111-19467;OP536770.1:20003-19359;OP597769.1:20025-19381;OP604566.1:20004-19360;OP612682.1:19930-19286;OP612684.1:19994-19350;OP612686.1:19994-19350;OP696841.1:17278-16634;OP879722.1:19227-18583;OQ419007.1:19237-18593;OQ419012.1:19229-18585;OQ419031.1:19234-18590;OQ419047.1:19237-18593;OQ451898.1:19089-18445;OQ451900.1:20002-19358;OQ581853.1:20028-19384;OQ581855.1:20109-19465;OQ721951.1:20042-19398;OQ721954.1:19999-19355;OR035652.1:19951-19307;OR035664.1:19951-19307;OR035702.1:19951-19307;OR146272.1:19967-19323;OR146330.1:20012-19368;OR264364.1:20022-19378;OR427332.1:20022-19378;OR459777.1:20006-19362;OR459797.1:20021-19377;OR943698.1:21988-21344;PP098589.2:20003-19359;PP496048.1:20007-19363;PP601206.1:22003-21359;PP776553.1:19952-19308;PP852971.1:20001-19357;PP852983.1:20006-19362;PP853000.1:19968-19324;PP853005.1:19976-19332;PP853007.1:20008-19364;PP853013.1:19982-19338;PP853027.1:20013-19369;PP853045.1:19994-19368;PP853048.1:20013-19369;PP860026.1:20008-19364;PP860027.1:20019-19375;PP860028.1:19975-19331;PP860031.1:20029-19385;PP860033.1:20018-19374;PQ153226.1:20001-19357;PQ178862.2:20862-20218;PQ221506.1:15824-15180;PQ221833.1:21995-21351;PQ340477.1:19984-19340;PQ340478.1:19969-19325;PQ340480.1:20048-19404;PQ340483.1:20032-19388;PQ340485.1:20018-19374;PQ340486.1:20033-19389;PQ340487.1:20058-19414;PQ340489.1:20033-19389;PQ340490.1:19984-19340;PQ340491.1:20042-19398;PQ340493.1:20018-19374;PQ340495.1:20048-19404;PQ340496.1:20081-19437;PQ368489.1:20004-19360;PQ451835.1:20092-19448;PQ451836.1:20024-19380;PQ451838.1:20075-19431;PQ451841.1:20035-19391;PQ451842.1:19987-19343 |
| OG0000159 | AY603973.1:20777-20439;AY741551.1:20644-20306;AY753185.1:21020-20682;DQ011154.1:21284-20961;DQ011155.1:21264-20941;EPI_ISL_13331598:19154-18774;EPI_ISL_13363142:19147-18767;EPI_ISL_13374487:19162-18782;EPI_ISL_13502582:19147-18767;EPI_ISL_13508471:19239-18859;EPI_ISL_13607904:19138-18758;EPI_ISL_13624509:19082-18702;EPI_ISL_13889442:19257-18877;EPI_ISL_13953610:19138-18758;EPI_ISL_14181948:19283-18903;EPI_ISL_14838587:21138-20815;EPI_ISL_15263355:19138-18758;EPI_ISL_15269699:19107-18727;EPI_ISL_15293002:19380-19000;EPI_ISL_15641561:19096-18716;EPI_ISL_15831211:19145-18765;EPI_ISL_15992098:19160-18780;EPI_ISL_16233784:19156-18776;EPI_ISL_16946400:19145-18765;EPI_ISL_17048206:18795-18415;EPI_ISL_17703567:19160-18780;EPI_ISL_17718458:19138-18758;EPI_ISL_18213374:19135-18755;EPI_ISL_18360394:18796-18416;EPI_ISL_18557816:19136-18756;EPI_ISL_18642365:19138-18758;EPI_ISL_18659846:19108-18728;EPI_ISL_18744049:19140-18760;EPI_ISL_19022858:19135-18755;EPI_ISL_19028778:19052-18672;EPI_ISL_19151800:19136-18756;EPI_ISL_19159116:19138-18758;EPI_ISL_19350788:20018-19695;EPI_ISL_19359677:19135-18755;EPI_ISL_19446306:19130-18750;EPI_ISL_19467692:21416-21093;JX878411.1:22131-21808;JX878425.1:21644-21321;JX878426.1:21087-20764;KC257460.1:21067-20744;KJ642612.1:19372-19049;KJ642613.1:20953-20630;KJ642616.1:20594-20256;KJ642618.1:18543-18220;KP849469.1:21454-21131;KP849471.1:21374-21051;LC831698.1:19136-18756;MN648051.1:19358-18978;MN702445.1:14797-14474;MN702446.1:20943-20620;MN702447.1:14306-13983;MN702449.1:14807-14484;MN702452.1:20983-20660;MT903338.1:19301-18921;MT903340.1:19138-18758;MT903341.1:10671-10291;MT903342.1:19153-18773;MT903347.1:20977-20639;ON568298.1:19284-18904;ON622713.1:19370-18990;ON622722.2:19141-18761;ON631963.1:19161-18781;ON649879.1:18971-18591;ON676707.1:19191-18811;ON755039.1:18801-18421;ON808413.1:19141-18761;ON880420.1:19368-18988;ON911481.2:19158-18778;ON918656.1:19173-18793;ON959133.1:19262-18882;ON959136.1:19263-18883;ON983168.1:17837-17457;OP019276.1:19135-18755;OP150924.1:19276-18896;OP160532.1:19101-18721;OP235282.1:19135-18755;OP289782.1:19138-18758;OP331335.1:19155-18775;OP382479.1:19142-18762;OP422341.1:19155-18775;OP434519.1:19134-18754;OP535324.1:19136-18756;OP535333.1:19245-18865;OP536770.1:19137-18757;OP597769.1:19159-18779;OP604566.1:19138-18758;OP612682.1:19064-18684;OP612684.1:19128-18748;OP612686.1:19128-18748;OP696841.1:16412-16032;OP879722.1:18361-17981;OQ419007.1:18371-17991;OQ419012.1:18363-17983;OQ419031.1:18368-17988;OQ419047.1:18371-17991;OQ451898.1:18223-17843;OQ451900.1:19136-18756;OQ581853.1:19162-18782;OQ581855.1:19243-18863;OQ721951.1:19176-18796;OQ721954.1:19133-18753;OR035652.1:19085-18705;OR035664.1:19085-18705;OR035702.1:19085-18705;OR146272.1:19101-18721;OR146330.1:19146-18766;OR264364.1:19156-18776;OR427332.1:19156-18776;OR459777.1:19140-18760;OR459797.1:19155-18775;OR943698.1:21121-20798;PP098589.2:19137-18757;PP496048.1:19141-18761;PP601206.1:21136-20813;PP776553.1:19086-18706;PP852971.1:19135-18755;PP852983.1:19140-18760;PP853000.1:19102-18722;PP853005.1:19110-18730;PP853007.1:19142-18762;PP853013.1:19116-18736;PP853027.1:19147-18767;PP853045.1:19146-18766;PP853048.1:19147-18767;PP860026.1:19142-18762;PP860027.1:19153-18773;PP860028.1:19109-18729;PP860031.1:19163-18783;PP860033.1:19152-18772;PQ153226.1:19135-18755;PQ178862.2:19995-19672;PQ221506.1:14958-14635;PQ221833.1:21128-20805;PQ340477.1:19118-18738;PQ340478.1:19103-18723;PQ340480.1:19182-18802;PQ340483.1:19166-18786;PQ340485.1:19152-18772;PQ340486.1:19167-18787;PQ340487.1:19192-18812;PQ340489.1:19167-18787;PQ340490.1:19118-18738;PQ340491.1:19176-18796;PQ340493.1:19152-18772;PQ340495.1:19182-18802;PQ340496.1:19215-18835;PQ368489.1:19138-18758;PQ451835.1:19226-18846;PQ451836.1:19158-18778;PQ451838.1:19209-18829;PQ451841.1:19169-18789;PQ451842.1:19121-18741 |
| OG0000160 | AY603973.1:18749-18279;AY741551.1:18616-18137;AY753185.1:18992-18525;DQ011154.1:17264-16803;DQ011155.1:17245-16784;EPI_ISL_13331598:17084-16617;EPI_ISL_13363142:17077-16610;EPI_ISL_13374487:17092-16625;EPI_ISL_13502582:17077-16610;EPI_ISL_13508471:17169-16702;EPI_ISL_13607904:17068-16601;EPI_ISL_13624509:17012-16545;EPI_ISL_13889442:17187-16720;EPI_ISL_13953610:17068-16601;EPI_ISL_14181948:17213-16746;EPI_ISL_14838587:17118-16657;EPI_ISL_15263355:17068-16598;EPI_ISL_15269699:17037-16570;EPI_ISL_15293002:17310-16843;EPI_ISL_15641561:17026-16559;EPI_ISL_15831211:17075-16608;EPI_ISL_15992098:17090-16623;EPI_ISL_16233784:17086-16619;EPI_ISL_16946400:17075-16608;EPI_ISL_17048206:16725-16258;EPI_ISL_17703567:17090-16623;EPI_ISL_17718458:17068-16601;EPI_ISL_18213374:17065-16598;EPI_ISL_18360394:16726-16259;EPI_ISL_18557816:17066-16599;EPI_ISL_18642365:17068-16601;EPI_ISL_18659846:17038-16571;EPI_ISL_18744049:17070-16603;EPI_ISL_19022858:17065-16598;EPI_ISL_19028778:16982-16515;EPI_ISL_19151800:17066-16599;EPI_ISL_19159116:17068-16601;EPI_ISL_19350788:17137-16676;EPI_ISL_19359677:17065-16598;EPI_ISL_19446306:17060-16593;EPI_ISL_19467692:17396-16935;JX878411.1:18110-17649;JX878425.1:17624-17163;JX878426.1:17067-16606;KC257460.1:17044-16583;KJ642612.1:15352-14891;KJ642613.1:16933-16472;KJ642616.1:18566-18096;KJ642618.1:14523-14062;KP849469.1:17434-16973;KP849471.1:17355-16894;LC831698.1:17066-16599;MN648051.1:17288-16821;MN702445.1:10775-10314;MN702446.1:16921-16460;MN702447.1:10286-9825;MN702449.1:10783-10322;MN702452.1:16963-16502;MT903338.1:17231-16764;MT903340.1:17068-16601;MT903341.1:8601-8134;MT903342.1:17083-16616;MT903347.1:18949-18473;ON568298.1:17214-16747;ON622713.1:17300-16833;ON622722.2:17071-16604;ON631963.1:17091-16624;ON649879.1:16901-16434;ON676707.1:17121-16654;ON755039.1:16731-16264;ON808413.1:17071-16604;ON880420.1:17298-16831;ON911481.2:17088-16621;ON918656.1:17103-16636;ON959133.1:17192-16725;ON959136.1:17193-16726;ON983168.1:15767-15300;OP019276.1:17065-16598;OP150924.1:17206-16739;OP160532.1:17031-16564;OP235282.1:17065-16598;OP289782.1:17068-16601;OP331335.1:17085-16618;OP382479.1:17072-16605;OP422341.1:17085-16618;OP434519.1:17064-16597;OP535324.1:17066-16599;OP535333.1:17175-16708;OP536770.1:17067-16600;OP597769.1:17089-16622;OP604566.1:17068-16601;OP612682.1:16994-16527;OP612684.1:17058-16591;OP612686.1:17058-16591;OP696841.1:14342-13875;OP879722.1:16291-15824;OQ419007.1:16301-15834;OQ419012.1:16293-15826;OQ419031.1:16298-15831;OQ419047.1:16301-15834;OQ451898.1:16153-15686;OQ451900.1:17066-16599;OQ581853.1:17092-16625;OQ581855.1:17173-16706;OQ721951.1:17106-16639;OQ721954.1:17063-16596;OR035652.1:17015-16548;OR035664.1:17015-16548;OR035702.1:17015-16548;OR146272.1:17031-16564;OR146330.1:17076-16609;OR264364.1:17086-16619;OR427332.1:17086-16619;OR459777.1:17070-16603;OR459797.1:17085-16618;OR943698.1:17101-16640;PP098589.2:17067-16600;PP496048.1:17071-16604;PP601206.1:17116-16655;PP776553.1:17016-16549;PP852971.1:17065-16598;PP852983.1:17070-16603;PP853000.1:17032-16565;PP853005.1:17040-16573;PP853007.1:17072-16605;PP853013.1:17046-16579;PP853027.1:17077-16610;PP853045.1:17076-16609;PP853048.1:17077-16610;PP860026.1:17072-16605;PP860027.1:17083-16604;PP860028.1:17039-16572;PP860031.1:17093-16611;PP860033.1:17082-16603;PQ153226.1:17065-16598;PQ178862.2:17114-16653;PQ221506.1:10938-10477;PQ221833.1:17108-16647;PQ340477.1:17048-16581;PQ340478.1:17033-16566;PQ340480.1:17112-16645;PQ340483.1:17096-16629;PQ340485.1:17082-16615;PQ340486.1:17097-16630;PQ340487.1:17122-16655;PQ340489.1:17097-16630;PQ340490.1:17048-16581;PQ340491.1:17106-16639;PQ340493.1:17082-16615;PQ340495.1:17112-16645;PQ340496.1:17145-16678;PQ368489.1:17068-16601;PQ451835.1:17156-16689;PQ451836.1:17088-16621;PQ451838.1:17139-16672;PQ451841.1:17099-16632;PQ451842.1:17051-16584 |
| OG0000161 | AY603973.1:153457-153275;AY741551.1:153531-153349;AY753185.1:153712-153530;DQ011154.1:153617-153435;DQ011155.1:153559-153377;EPI_ISL_13331598:151576-151394;EPI_ISL_13363142:151564-151382;EPI_ISL_13374487:151597-151415;EPI_ISL_13502582:151592-151410;EPI_ISL_13508471:151658-151476;EPI_ISL_13607904:151563-151381;EPI_ISL_13624509:151498-151316;EPI_ISL_13889442:151691-151509;EPI_ISL_13953610:151491-151309;EPI_ISL_14181948:151721-151539;EPI_ISL_14838587:153556-153374;EPI_ISL_15263355:151557-151375;EPI_ISL_15269699:151526-151344;EPI_ISL_15293002:151795-151613;EPI_ISL_15641561:151512-151330;EPI_ISL_15831211:151566-151384;EPI_ISL_15992098:151606-151424;EPI_ISL_16233784:151578-151396;EPI_ISL_16946400:151568-151386;EPI_ISL_17048206:151232-151050;EPI_ISL_17703567:151579-151397;EPI_ISL_17718458:151559-151377;EPI_ISL_18213374:151570-151388;EPI_ISL_18360394:150877-150695;EPI_ISL_18557816:151573-151391;EPI_ISL_18642365:151568-151386;EPI_ISL_18659846:151527-151345;EPI_ISL_18744049:151576-151394;EPI_ISL_19022858:151533-151351;EPI_ISL_19028778:151485-151303;EPI_ISL_19151800:151569-151387;EPI_ISL_19159116:151554-151372;EPI_ISL_19350788:152216-152034;EPI_ISL_19359677:151569-151387;EPI_ISL_19446306:151556-151374;EPI_ISL_19467692:153757-153575;JX878411.1:154458-154276;JX878425.1:153946-153764;JX878426.1:153486-153304;KC257460.1:153342-153160;KJ642612.1:151676-151494;KJ642613.1:153225-153043;KJ642616.1:153179-152997;KJ642618.1:151141-150959;KP849469.1:153774-153592;KP849471.1:153707-153525;LC831698.1:151552-151370;MN648051.1:151795-151613;MN702445.1:147093-146911;MN702446.1:153257-153075;MN702447.1:146620-146438;MN702449.1:147131-146949;MN702452.1:153308-153126;MT903338.1:151728-151546;MT903340.1:151581-151399;MT903341.1:143122-142940;MT903342.1:151618-151436;MT903347.1:153524-153342;ON568298.1:151722-151540;ON622713.1:151786-151604;ON622722.2:151574-151392;ON631963.1:151583-151401;ON649879.1:151389-151207;ON676707.1:151620-151438;ON755039.1:151211-151029;ON808413.1:151548-151366;ON880420.1:151784-151602;ON911481.2:151569-151387;ON918656.1:151591-151409;ON959133.1:151680-151498;ON959136.1:151681-151499;ON983168.1:150257-150075;OP019276.1:151568-151386;OP150924.1:151694-151512;OP160532.1:151517-151335;OP235282.1:151568-151386;OP289782.1:151572-151390;OP331335.1:151577-151395;OP382479.1:151561-151379;OP422341.1:151574-151392;OP434519.1:151550-151368;OP535324.1:151581-151399;OP535333.1:151688-151506;OP536770.1:151571-151389;OP597769.1:151605-151423;OP604566.1:151571-151389;OP612682.1:151506-151324;OP612684.1:151557-151375;OP612686.1:151559-151377;OP696841.1:148847-148665;OP879722.1:150794-150612;OQ419007.1:150788-150606;OQ419012.1:150762-150580;OQ419031.1:150766-150584;OQ419047.1:150768-150586;OQ451898.1:150658-150476;OQ451900.1:151572-151390;OQ581853.1:151580-151398;OQ581855.1:151679-151497;OQ721951.1:151598-151416;OQ721954.1:151571-151389;OR035652.1:151500-151318;OR035664.1:151504-151322;OR035702.1:151501-151319;OR146272.1:151520-151338;OR146330.1:151581-151399;OR264364.1:151575-151393;OR427332.1:151575-151393;OR459777.1:151564-151382;OR459797.1:151573-151391;OR943698.1:153462-153280;PP098589.2:151570-151388;PP496048.1:151574-151392;PP601206.1:153517-153335;PP776553.1:151494-151312;PP852971.1:151562-151380;PP852983.1:151579-151397;PP853000.1:151530-151348;PP853005.1:151538-151356;PP853007.1:151581-151399;PP853013.1:151553-151371;PP853027.1:151595-151413;PP853045.1:151546-151364;PP853048.1:151571-151389;PP860026.1:151566-151384;PP860027.1:151603-151421;PP860028.1:151529-151347;PP860031.1:151600-151418;PP860033.1:151586-151404;PQ153226.1:151551-151369;PQ178862.2:152195-152013;PQ221506.1:147306-147124;PQ221833.1:153493-153311;PQ340477.1:151538-151356;PQ340478.1:151520-151338;PQ340480.1:151598-151416;PQ340483.1:151584-151402;PQ340485.1:151567-151385;PQ340486.1:151585-151403;PQ340487.1:151609-151427;PQ340489.1:151584-151402;PQ340490.1:151536-151354;PQ340491.1:151591-151409;PQ340493.1:151575-151393;PQ340495.1:151601-151419;PQ340496.1:151636-151454;PQ368489.1:151584-151402;PQ451835.1:151641-151459;PQ451836.1:151579-151397;PQ451838.1:151629-151447;PQ451841.1:151592-151410;PQ451842.1:151538-151356 |
| OG0000162 | AY603973.1:142978-142076;AY741551.1:143023-142121;AY753185.1:143221-142319;DQ011154.1:143047-142145;DQ011155.1:143016-142114;EPI_ISL_13331598:141085-140183;EPI_ISL_13363142:141072-140170;EPI_ISL_13374487:141089-140187;EPI_ISL_13502582:141084-140182;EPI_ISL_13508471:141167-140265;EPI_ISL_13607904:141063-140161;EPI_ISL_13624509:141007-140105;EPI_ISL_13889442:141184-140282;EPI_ISL_13953610:140990-140088;EPI_ISL_14181948:141213-140311;EPI_ISL_14838587:142913-142011;EPI_ISL_15263355:141066-140164;EPI_ISL_15269699:141035-140133;EPI_ISL_15293002:141304-140402;EPI_ISL_15641561:141021-140119;EPI_ISL_15831211:141074-140172;EPI_ISL_15992098:141098-140196;EPI_ISL_16233784:141087-140185;EPI_ISL_16946400:141076-140174;EPI_ISL_17048206:140723-139821;EPI_ISL_17703567:141088-140186;EPI_ISL_17718458:141058-140156;EPI_ISL_18213374:141062-140160;EPI_ISL_18360394:140721-139819;EPI_ISL_18557816:141063-140161;EPI_ISL_18642365:141060-140158;EPI_ISL_18659846:141036-140134;EPI_ISL_18744049:141075-140173;EPI_ISL_19022858:141042-140140;EPI_ISL_19028778:140977-140075;EPI_ISL_19151800:141060-140158;EPI_ISL_19159116:141063-140161;EPI_ISL_19350788:141739-140837;EPI_ISL_19359677:141060-140158;EPI_ISL_19446306:141065-140163;EPI_ISL_19467692:143177-142275;JX878411.1:143892-142990;JX878425.1:143400-142498;JX878426.1:142853-141951;KC257460.1:142793-141891;KJ642612.1:141122-140220;KJ642613.1:142682-141780;KJ642616.1:142703-141801;KJ642618.1:140309-139407;KP849469.1:143214-142312;KP849471.1:143141-142239;LC831698.1:141061-140159;MN648051.1:141287-140385;MN702445.1:136539-135637;MN702446.1:142702-141800;MN702447.1:136076-135174;MN702449.1:136565-135663;MN702452.1:142749-141847;MT903338.1:141239-140337;MT903340.1:141073-140171;MT903341.1:132607-131705;MT903342.1:141091-140189;MT903347.1:142954-142142;ON568298.1:141214-140312;ON622713.1:141295-140393;ON622722.2:141066-140164;ON631963.1:141092-140190;ON649879.1:140898-139996;ON676707.1:141119-140217;ON755039.1:140720-139818;ON808413.1:141057-140155;ON880420.1:141293-140391;ON911481.2:141078-140176;ON918656.1:141100-140198;ON959133.1:141189-140287;ON959136.1:141190-140288;ON983168.1:139765-138863;OP019276.1:141060-140158;OP150924.1:141203-140301;OP160532.1:141026-140124;OP235282.1:141060-140158;OP289782.1:141063-140161;OP331335.1:141093-140191;OP382479.1:141070-140168;OP422341.1:141083-140181;OP434519.1:141059-140157;OP535324.1:141073-140171;OP535333.1:141180-140278;OP536770.1:141063-140161;OP597769.1:141097-140195;OP604566.1:141063-140161;OP612682.1:140998-140096;OP612684.1:141049-140147;OP612686.1:141051-140149;OP696841.1:138339-137437;OP879722.1:140286-139384;OQ419007.1:140289-139387;OQ419012.1:140263-139361;OQ419031.1:140267-139365;OQ419047.1:140269-139367;OQ451898.1:140150-139248;OQ451900.1:141063-140161;OQ581853.1:141089-140187;OQ581855.1:141170-140268;OQ721951.1:141090-140188;OQ721954.1:141063-140161;OR035652.1:141009-140107;OR035664.1:141013-140111;OR035702.1:141010-140108;OR146272.1:141029-140127;OR146330.1:141073-140171;OR264364.1:141084-140182;OR427332.1:141084-140182;OR459777.1:141063-140161;OR459797.1:141082-140180;OR943698.1:142923-142021;PP098589.2:141062-140160;PP496048.1:141066-140164;PP601206.1:142883-141981;PP776553.1:140986-140084;PP852971.1:141061-140159;PP852983.1:141078-140176;PP853000.1:141029-140127;PP853005.1:141037-140135;PP853007.1:141080-140178;PP853013.1:141052-140150;PP853027.1:141085-140183;PP853045.1:141045-140143;PP853048.1:141070-140168;PP860026.1:141075-140173;PP860027.1:141129-140227;PP860028.1:141038-140136;PP860031.1:141111-140209;PP860033.1:141112-140210;PQ153226.1:141060-140158;PQ178862.2:141719-140817;PQ221506.1:136711-135809;PQ221833.1:142886-141984;PQ340477.1:141047-140145;PQ340478.1:141029-140127;PQ340480.1:141107-140205;PQ340483.1:141093-140191;PQ340485.1:141076-140174;PQ340486.1:141094-140192;PQ340487.1:141118-140216;PQ340489.1:141093-140191;PQ340490.1:141045-140143;PQ340491.1:141100-140198;PQ340493.1:141084-140182;PQ340495.1:141110-140208;PQ340496.1:141145-140243;PQ368489.1:141076-140174;PQ451835.1:141150-140248;PQ451836.1:141088-140186;PQ451838.1:141138-140236;PQ451841.1:141101-140199;PQ451842.1:141047-140145 |
| OG0000163 | AY603973.1:141322-140405;AY741551.1:141367-140450;AY753185.1:141565-140648;DQ011154.1:141385-140468;DQ011155.1:141354-140437;EPI_ISL_13331598:139420-138503;EPI_ISL_13363142:139407-138490;EPI_ISL_13374487:139424-138507;EPI_ISL_13502582:139419-138502;EPI_ISL_13508471:139502-138585;EPI_ISL_13607904:139398-138481;EPI_ISL_13624509:139342-138425;EPI_ISL_13889442:139519-138602;EPI_ISL_13953610:139325-138408;EPI_ISL_14181948:139548-138631;EPI_ISL_14838587:141251-140334;EPI_ISL_15263355:139401-138484;EPI_ISL_15269699:139370-138453;EPI_ISL_15293002:139639-138722;EPI_ISL_15641561:139356-138439;EPI_ISL_15831211:139409-138492;EPI_ISL_15992098:139433-138516;EPI_ISL_16233784:139422-138505;EPI_ISL_16946400:139411-138494;EPI_ISL_17048206:139058-138141;EPI_ISL_17703567:139423-138506;EPI_ISL_17718458:139393-138476;EPI_ISL_18213374:139397-138480;EPI_ISL_18360394:139056-138139;EPI_ISL_18557816:139398-138481;EPI_ISL_18642365:139395-138478;EPI_ISL_18659846:139371-138454;EPI_ISL_18744049:139410-138493;EPI_ISL_19022858:139395-138478;EPI_ISL_19028778:139312-138395;EPI_ISL_19151800:139395-138478;EPI_ISL_19159116:139398-138481;EPI_ISL_19350788:140083-139166;EPI_ISL_19359677:139395-138478;EPI_ISL_19446306:139400-138483;EPI_ISL_19467692:141524-140607;JX878411.1:142230-141313;JX878425.1:141738-140821;JX878426.1:141200-140283;KC257460.1:141131-140214;KJ642612.1:139469-138552;KJ642613.1:141020-140103;KJ642616.1:141047-140130;KJ642618.1:138656-137739;KP849469.1:141552-140635;KP849471.1:141479-140562;LC831698.1:139396-138479;MN648051.1:139622-138705;MN702445.1:134886-133969;MN702446.1:141049-140132;MN702447.1:134405-133488;MN702449.1:134912-133995;MN702452.1:141087-140170;MT903338.1:139574-138657;MT903340.1:139408-138491;MT903341.1:130942-130025;MT903342.1:139426-138509;MT903347.1:141388-140471;ON568298.1:139549-138632;ON622713.1:139630-138713;ON622722.2:139401-138484;ON631963.1:139427-138510;ON649879.1:139233-138316;ON676707.1:139454-138537;ON755039.1:139055-138138;ON808413.1:139392-138475;ON880420.1:139628-138711;ON911481.2:139413-138496;ON918656.1:139435-138518;ON959133.1:139524-138607;ON959136.1:139525-138608;ON983168.1:138100-137183;OP019276.1:139395-138478;OP150924.1:139538-138621;OP160532.1:139361-138444;OP235282.1:139395-138478;OP289782.1:139398-138481;OP331335.1:139428-138511;OP382479.1:139405-138488;OP422341.1:139418-138501;OP434519.1:139394-138477;OP535324.1:139408-138491;OP535333.1:139515-138598;OP536770.1:139398-138481;OP597769.1:139432-138515;OP604566.1:139398-138481;OP612682.1:139333-138416;OP612684.1:139384-138467;OP612686.1:139386-138469;OP696841.1:136674-135757;OP879722.1:138621-137704;OQ419007.1:138624-137707;OQ419012.1:138598-137681;OQ419031.1:138602-137685;OQ419047.1:138604-137687;OQ451898.1:138485-137568;OQ451900.1:139398-138481;OQ581853.1:139424-138507;OQ581855.1:139505-138588;OQ721951.1:139425-138508;OQ721954.1:139398-138481;OR035652.1:139344-138427;OR035664.1:139348-138431;OR035702.1:139345-138428;OR146272.1:139364-138447;OR146330.1:139408-138491;OR264364.1:139419-138502;OR427332.1:139419-138502;OR459777.1:139398-138481;OR459797.1:139417-138500;OR943698.1:141270-140353;PP098589.2:139397-138480;PP496048.1:139401-138484;PP601206.1:141230-140313;PP776553.1:139321-138404;PP852971.1:139396-138479;PP852983.1:139413-138496;PP853000.1:139364-138447;PP853005.1:139372-138455;PP853007.1:139415-138498;PP853013.1:139387-138470;PP853027.1:139420-138503;PP853045.1:139380-138463;PP853048.1:139405-138488;PP860026.1:139419-138502;PP860027.1:139473-138556;PP860028.1:139382-138465;PP860031.1:139455-138538;PP860033.1:139456-138539;PQ153226.1:139395-138478;PQ178862.2:140063-139146;PQ221506.1:135058-134141;PQ221833.1:141233-140316;PQ340477.1:139382-138465;PQ340478.1:139364-138447;PQ340480.1:139442-138525;PQ340483.1:139428-138511;PQ340485.1:139411-138494;PQ340486.1:139429-138512;PQ340487.1:139453-138536;PQ340489.1:139428-138511;PQ340490.1:139380-138463;PQ340491.1:139435-138518;PQ340493.1:139419-138502;PQ340495.1:139445-138528;PQ340496.1:139480-138563;PQ368489.1:139411-138494;PQ451835.1:139485-138568;PQ451836.1:139423-138506;PQ451838.1:139473-138556;PQ451841.1:139436-138519;PQ451842.1:139382-138465 |
| OG0000164 | AY603973.1:140404-139964;AY741551.1:140449-140009;AY753185.1:140647-140207;DQ011154.1:140467-140027;DQ011155.1:140436-139996;EPI_ISL_13331598:138502-138062;EPI_ISL_13363142:138489-138049;EPI_ISL_13374487:138506-138066;EPI_ISL_13502582:138501-138061;EPI_ISL_13508471:138584-138144;EPI_ISL_13607904:138480-138040;EPI_ISL_13624509:138424-137984;EPI_ISL_13889442:138601-138161;EPI_ISL_13953610:138407-137967;EPI_ISL_14181948:138630-138190;EPI_ISL_14838587:140333-139893;EPI_ISL_15263355:138483-138043;EPI_ISL_15269699:138452-138012;EPI_ISL_15293002:138721-138281;EPI_ISL_15641561:138438-137998;EPI_ISL_15831211:138491-138051;EPI_ISL_15992098:138515-138075;EPI_ISL_16233784:138504-138064;EPI_ISL_16946400:138493-138053;EPI_ISL_17048206:138140-137700;EPI_ISL_17703567:138505-138065;EPI_ISL_17718458:138475-138035;EPI_ISL_18213374:138479-138039;EPI_ISL_18360394:138138-137698;EPI_ISL_18557816:138480-138040;EPI_ISL_18642365:138477-138037;EPI_ISL_18659846:138453-138013;EPI_ISL_18744049:138492-138052;EPI_ISL_19022858:138477-138037;EPI_ISL_19028778:138394-137954;EPI_ISL_19151800:138477-138037;EPI_ISL_19159116:138480-138040;EPI_ISL_19350788:139165-138725;EPI_ISL_19359677:138477-138037;EPI_ISL_19446306:138482-138042;EPI_ISL_19467692:140606-140166;JX878411.1:141312-140872;JX878425.1:140820-140380;JX878426.1:140282-139842;KC257460.1:140213-139773;KJ642612.1:138551-138111;KJ642613.1:140102-139662;KJ642616.1:140129-139689;KJ642618.1:137738-137298;KP849469.1:140634-140194;KP849471.1:140561-140121;LC831698.1:138478-138038;MN648051.1:138704-138264;MN702445.1:133968-133528;MN702446.1:140131-139691;MN702447.1:133487-133047;MN702449.1:133994-133554;MN702452.1:140169-139729;MT903338.1:138656-138216;MT903340.1:138490-138050;MT903341.1:130024-129584;MT903342.1:138508-138068;MT903347.1:140470-140030;ON568298.1:138631-138191;ON622713.1:138712-138272;ON622722.2:138483-138043;ON631963.1:138509-138069;ON649879.1:138315-137875;ON676707.1:138536-138096;ON755039.1:138137-137697;ON808413.1:138474-138034;ON880420.1:138710-138270;ON911481.2:138495-138055;ON918656.1:138517-138077;ON959133.1:138606-138166;ON959136.1:138607-138167;ON983168.1:137182-136742;OP019276.1:138477-138037;OP150924.1:138620-138180;OP160532.1:138443-138003;OP235282.1:138477-138037;OP289782.1:138480-138040;OP331335.1:138510-138070;OP382479.1:138487-138047;OP422341.1:138500-138060;OP434519.1:138476-138036;OP535324.1:138490-138050;OP535333.1:138597-138157;OP536770.1:138480-138040;OP597769.1:138514-138074;OP604566.1:138480-138040;OP612682.1:138415-137975;OP612684.1:138466-138026;OP612686.1:138468-138028;OP696841.1:135756-135316;OP879722.1:137703-137263;OQ419007.1:137706-137266;OQ419012.1:137680-137240;OQ419031.1:137684-137244;OQ419047.1:137686-137246;OQ451898.1:137567-137127;OQ451900.1:138480-138040;OQ581853.1:138506-138066;OQ581855.1:138587-138147;OQ721951.1:138507-138067;OQ721954.1:138480-138040;OR035652.1:138426-137986;OR035664.1:138430-137990;OR035702.1:138427-137987;OR146272.1:138446-138006;OR146330.1:138490-138050;OR264364.1:138501-138061;OR427332.1:138501-138061;OR459777.1:138480-138040;OR459797.1:138499-138059;OR943698.1:140352-139912;PP098589.2:138479-138039;PP496048.1:138483-138043;PP601206.1:140312-139872;PP776553.1:138403-137963;PP852971.1:138478-138038;PP852983.1:138495-138055;PP853000.1:138446-138006;PP853005.1:138454-138014;PP853007.1:138497-138057;PP853013.1:138469-138029;PP853027.1:138502-138062;PP853045.1:138462-138022;PP853048.1:138487-138047;PP860026.1:138501-138061;PP860027.1:138555-138115;PP860028.1:138464-138024;PP860031.1:138537-138097;PP860033.1:138538-138098;PQ153226.1:138477-138037;PQ178862.2:139145-138705;PQ221506.1:134140-133700;PQ221833.1:140315-139875;PQ340477.1:138464-138024;PQ340478.1:138446-138006;PQ340480.1:138524-138084;PQ340483.1:138510-138070;PQ340485.1:138493-138053;PQ340486.1:138511-138071;PQ340487.1:138535-138095;PQ340489.1:138510-138070;PQ340490.1:138462-138022;PQ340491.1:138517-138077;PQ340493.1:138501-138061;PQ340495.1:138527-138087;PQ340496.1:138562-138122;PQ368489.1:138493-138053;PQ451835.1:138567-138127;PQ451836.1:138505-138065;PQ451838.1:138555-138115;PQ451841.1:138518-138078;PQ451842.1:138464-138024 |
| OG0000165 | AY603973.1:139963-139631;AY741551.1:140008-139676;AY753185.1:140206-139874;DQ011154.1:140026-139694;DQ011155.1:139995-139663;EPI_ISL_13331598:138061-137729;EPI_ISL_13363142:138048-137716;EPI_ISL_13374487:138065-137733;EPI_ISL_13502582:138060-137728;EPI_ISL_13508471:138143-137811;EPI_ISL_13607904:138039-137707;EPI_ISL_13624509:137983-137651;EPI_ISL_13889442:138160-137828;EPI_ISL_13953610:137966-137634;EPI_ISL_14181948:138189-137857;EPI_ISL_14838587:139892-139560;EPI_ISL_15263355:138042-137710;EPI_ISL_15269699:138011-137679;EPI_ISL_15293002:138280-137948;EPI_ISL_15641561:137997-137665;EPI_ISL_15831211:138050-137718;EPI_ISL_15992098:138074-137742;EPI_ISL_16233784:138063-137731;EPI_ISL_16946400:138052-137720;EPI_ISL_17048206:137699-137367;EPI_ISL_17703567:138064-137732;EPI_ISL_17718458:138034-137702;EPI_ISL_18213374:138038-137706;EPI_ISL_18360394:137697-137365;EPI_ISL_18557816:138039-137707;EPI_ISL_18642365:138036-137704;EPI_ISL_18659846:138012-137680;EPI_ISL_18744049:138051-137719;EPI_ISL_19022858:138036-137704;EPI_ISL_19028778:137953-137621;EPI_ISL_19151800:138036-137704;EPI_ISL_19159116:138039-137707;EPI_ISL_19350788:138724-138392;EPI_ISL_19359677:138036-137704;EPI_ISL_19446306:138041-137709;EPI_ISL_19467692:140165-139833;JX878411.1:140871-140539;JX878425.1:140379-140047;JX878426.1:139841-139509;KC257460.1:139772-139440;KJ642612.1:138110-137778;KJ642613.1:139661-139329;KJ642616.1:139688-139356;KJ642618.1:137297-136965;KP849469.1:140193-139861;KP849471.1:140120-139788;LC831698.1:138037-137705;MN648051.1:138263-137931;MN702445.1:133527-133195;MN702446.1:139690-139358;MN702447.1:133046-132714;MN702449.1:133553-133221;MN702452.1:139728-139396;MT903338.1:138215-137883;MT903340.1:138049-137717;MT903341.1:129583-129251;MT903342.1:138067-137735;MT903347.1:140029-139697;ON568298.1:138190-137858;ON622713.1:138271-137939;ON622722.2:138042-137710;ON631963.1:138068-137736;ON649879.1:137874-137542;ON676707.1:138095-137763;ON755039.1:137696-137364;ON808413.1:138033-137701;ON880420.1:138269-137937;ON911481.2:138054-137722;ON918656.1:138076-137744;ON959133.1:138165-137833;ON959136.1:138166-137834;ON983168.1:136741-136409;OP019276.1:138036-137704;OP150924.1:138179-137847;OP160532.1:138002-137670;OP235282.1:138036-137704;OP289782.1:138039-137707;OP331335.1:138069-137737;OP382479.1:138046-137714;OP422341.1:138059-137727;OP434519.1:138035-137703;OP535324.1:138049-137717;OP535333.1:138156-137824;OP536770.1:138039-137707;OP597769.1:138073-137741;OP604566.1:138039-137707;OP612682.1:137974-137642;OP612684.1:138025-137693;OP612686.1:138027-137695;OP696841.1:135315-134983;OP879722.1:137262-136930;OQ419007.1:137265-136933;OQ419012.1:137239-136907;OQ419031.1:137243-136911;OQ419047.1:137245-136913;OQ451898.1:137126-136794;OQ451900.1:138039-137707;OQ581853.1:138065-137733;OQ581855.1:138146-137814;OQ721951.1:138066-137734;OQ721954.1:138039-137707;OR035652.1:137985-137653;OR035664.1:137989-137657;OR035702.1:137986-137654;OR146272.1:138005-137673;OR146330.1:138049-137717;OR264364.1:138060-137728;OR427332.1:138060-137728;OR459777.1:138039-137707;OR459797.1:138058-137726;OR943698.1:139911-139579;PP098589.2:138038-137706;PP496048.1:138042-137710;PP601206.1:139871-139539;PP776553.1:137962-137630;PP852971.1:138037-137705;PP852983.1:138054-137722;PP853000.1:138005-137673;PP853005.1:138013-137681;PP853007.1:138056-137724;PP853013.1:138028-137696;PP853027.1:138061-137729;PP853045.1:138021-137689;PP853048.1:138046-137714;PP860026.1:138060-137728;PP860027.1:138114-137782;PP860028.1:138023-137691;PP860031.1:138096-137764;PP860033.1:138097-137765;PQ153226.1:138036-137704;PQ178862.2:138704-138372;PQ221506.1:133699-133367;PQ221833.1:139874-139542;PQ340477.1:138023-137691;PQ340478.1:138005-137673;PQ340480.1:138083-137751;PQ340483.1:138069-137737;PQ340485.1:138052-137720;PQ340486.1:138070-137738;PQ340487.1:138094-137762;PQ340489.1:138069-137737;PQ340490.1:138021-137689;PQ340491.1:138076-137744;PQ340493.1:138060-137728;PQ340495.1:138086-137754;PQ340496.1:138121-137789;PQ368489.1:138052-137720;PQ451835.1:138126-137794;PQ451836.1:138064-137732;PQ451838.1:138114-137782;PQ451841.1:138077-137745;PQ451842.1:138023-137691 |
| OG0000166 | AY603973.1:134587-134417;AY741551.1:134609-134439;AY753185.1:134830-134660;DQ011154.1:135044-134874;DQ011155.1:135025-134855;EPI_ISL_13331598:132634-132464;EPI_ISL_13363142:132627-132457;EPI_ISL_13374487:132642-132472;EPI_ISL_13502582:132627-132457;EPI_ISL_13508471:132719-132549;EPI_ISL_13607904:132618-132448;EPI_ISL_13624509:132562-132392;EPI_ISL_13889442:132737-132567;EPI_ISL_13953610:132535-132365;EPI_ISL_14181948:132763-132593;EPI_ISL_14838587:134898-134728;EPI_ISL_15263355:132618-132448;EPI_ISL_15269699:132587-132417;EPI_ISL_15293002:132860-132690;EPI_ISL_15641561:132576-132406;EPI_ISL_15831211:132625-132455;EPI_ISL_15992098:132640-132470;EPI_ISL_16233784:132639-132469;EPI_ISL_16946400:132625-132455;EPI_ISL_17048206:132275-132105;EPI_ISL_17703567:132640-132470;EPI_ISL_17718458:132618-132448;EPI_ISL_18213374:132615-132445;EPI_ISL_18360394:132276-132106;EPI_ISL_18557816:132616-132446;EPI_ISL_18642365:132618-132448;EPI_ISL_18659846:132588-132418;EPI_ISL_18744049:132620-132450;EPI_ISL_19022858:132615-132445;EPI_ISL_19028778:132532-132362;EPI_ISL_19151800:132616-132446;EPI_ISL_19159116:132618-132448;EPI_ISL_19350788:133769-133599;EPI_ISL_19359677:132615-132445;EPI_ISL_19446306:132610-132440;EPI_ISL_19467692:135180-135010;JX878411.1:135889-135719;JX878425.1:135406-135236;JX878426.1:134856-134686;KC257460.1:134823-134653;KJ642612.1:133140-132970;KJ642613.1:134700-134530;KJ642616.1:134287-134117;KJ642618.1:132371-132201;KP849469.1:135214-135044;KP849471.1:135140-134970;LC831698.1:132616-132446;MN648051.1:132838-132668;MN702445.1:128557-128387;MN702446.1:134702-134532;MN702447.1:128070-127900;MN702449.1:128568-128398;MN702452.1:134743-134573;MT903338.1:132781-132611;MT903340.1:132618-132448;MT903341.1:124151-123981;MT903342.1:132633-132463;MT903347.1:134616-134446;ON568298.1:132764-132594;ON622713.1:132850-132680;ON622722.2:132621-132451;ON631963.1:132641-132471;ON649879.1:132451-132281;ON676707.1:132671-132501;ON755039.1:132281-132111;ON808413.1:132621-132451;ON880420.1:132848-132678;ON911481.2:132638-132468;ON918656.1:132653-132483;ON959133.1:132742-132572;ON959136.1:132743-132573;ON983168.1:131316-131146;OP019276.1:132615-132445;OP150924.1:132756-132586;OP160532.1:132581-132411;OP235282.1:132615-132445;OP289782.1:132618-132448;OP331335.1:132635-132465;OP382479.1:132622-132452;OP422341.1:132635-132465;OP434519.1:132614-132444;OP535324.1:132616-132446;OP535333.1:132725-132555;OP536770.1:132617-132447;OP597769.1:132639-132469;OP604566.1:132618-132448;OP612682.1:132543-132373;OP612684.1:132605-132435;OP612686.1:132607-132437;OP696841.1:129892-129722;OP879722.1:131841-131671;OQ419007.1:131851-131681;OQ419012.1:131818-131648;OQ419031.1:131822-131652;OQ419047.1:131824-131654;OQ451898.1:131703-131533;OQ451900.1:132616-132446;OQ581853.1:132642-132472;OQ581855.1:132723-132553;OQ721951.1:132656-132486;OQ721954.1:132613-132443;OR035652.1:132565-132395;OR035664.1:132565-132395;OR035702.1:132565-132395;OR146272.1:132581-132411;OR146330.1:132626-132456;OR264364.1:132636-132466;OR427332.1:132636-132466;OR459777.1:132620-132450;OR459797.1:132634-132464;OR943698.1:134956-134786;PP098589.2:132617-132447;PP496048.1:132621-132451;PP601206.1:134886-134716;PP776553.1:132542-132372;PP852971.1:132615-132445;PP852983.1:132621-132451;PP853000.1:132582-132412;PP853005.1:132590-132420;PP853007.1:132623-132453;PP853013.1:132596-132426;PP853027.1:132627-132457;PP853045.1:132608-132438;PP853048.1:132627-132457;PP860026.1:132625-132455;PP860027.1:132651-132481;PP860028.1:132590-132420;PP860031.1:132644-132474;PP860033.1:132650-132480;PQ153226.1:132615-132445;PQ178862.2:133746-133576;PQ221506.1:128723-128553;PQ221833.1:134889-134719;PQ340477.1:132598-132428;PQ340478.1:132583-132413;PQ340480.1:132662-132492;PQ340483.1:132646-132476;PQ340485.1:132632-132462;PQ340486.1:132647-132477;PQ340487.1:132672-132502;PQ340489.1:132647-132477;PQ340490.1:132598-132428;PQ340491.1:132653-132483;PQ340493.1:132632-132462;PQ340495.1:132662-132492;PQ340496.1:132695-132525;PQ368489.1:132618-132448;PQ451835.1:132703-132533;PQ451836.1:132635-132465;PQ451838.1:132686-132516;PQ451841.1:132646-132476;PQ451842.1:132598-132428 |
| OG0000167 | AY603973.1:123883-123722;AY741551.1:123905-123744;AY753185.1:124126-123965;DQ011154.1:124340-124179;DQ011155.1:124321-124160;EPI_ISL_13331598:121939-121778;EPI_ISL_13363142:121932-121771;EPI_ISL_13374487:121947-121786;EPI_ISL_13502582:121932-121771;EPI_ISL_13508471:122024-121863;EPI_ISL_13607904:121923-121762;EPI_ISL_13624509:121867-121706;EPI_ISL_13889442:122042-121881;EPI_ISL_13953610:121840-121679;EPI_ISL_14181948:122068-121907;EPI_ISL_14838587:124194-124033;EPI_ISL_15263355:121923-121762;EPI_ISL_15269699:121892-121731;EPI_ISL_15293002:122165-122004;EPI_ISL_15641561:121881-121720;EPI_ISL_15831211:121930-121769;EPI_ISL_15992098:121945-121784;EPI_ISL_16233784:121941-121780;EPI_ISL_16946400:121930-121769;EPI_ISL_17048206:121580-121419;EPI_ISL_17703567:121945-121784;EPI_ISL_17718458:121923-121762;EPI_ISL_18213374:121920-121759;EPI_ISL_18360394:121581-121420;EPI_ISL_18557816:121921-121760;EPI_ISL_18642365:121923-121762;EPI_ISL_18659846:121893-121732;EPI_ISL_18744049:121925-121764;EPI_ISL_19022858:121920-121759;EPI_ISL_19028778:121837-121676;EPI_ISL_19151800:121921-121760;EPI_ISL_19159116:121923-121762;EPI_ISL_19350788:123065-122904;EPI_ISL_19359677:121920-121759;EPI_ISL_19446306:121915-121754;EPI_ISL_19467692:124476-124315;JX878411.1:125185-125024;JX878425.1:124702-124541;JX878426.1:124152-123991;KC257460.1:124119-123958;KJ642612.1:122436-122275;KJ642613.1:123996-123835;KJ642616.1:123583-123422;KJ642618.1:121667-121506;KP849469.1:124510-124349;KP849471.1:124436-124275;LC831698.1:121921-121760;MN648051.1:122143-121982;MN702445.1:117853-117692;MN702446.1:123998-123837;MN702447.1:117366-117205;MN702449.1:117864-117703;MN702452.1:124039-123878;MT903338.1:122086-121925;MT903340.1:121923-121762;MT903341.1:113456-113295;MT903342.1:121938-121777;MT903347.1:123912-123751;ON568298.1:122069-121908;ON622713.1:122155-121994;ON622722.2:121926-121765;ON631963.1:121946-121785;ON649879.1:121756-121595;ON676707.1:121976-121815;ON755039.1:121586-121425;ON808413.1:121926-121765;ON880420.1:122153-121992;ON911481.2:121943-121782;ON918656.1:121958-121797;ON959133.1:122047-121886;ON959136.1:122048-121887;ON983168.1:120621-120460;OP019276.1:121920-121759;OP150924.1:122061-121900;OP160532.1:121886-121725;OP235282.1:121920-121759;OP289782.1:121923-121762;OP331335.1:121940-121779;OP382479.1:121927-121766;OP422341.1:121940-121779;OP434519.1:121919-121758;OP535324.1:121921-121760;OP535333.1:122030-121869;OP536770.1:121922-121761;OP597769.1:121944-121783;OP604566.1:121923-121762;OP612682.1:121848-121687;OP612684.1:121910-121749;OP612686.1:121912-121751;OP696841.1:119197-119036;OP879722.1:121146-120985;OQ419007.1:121156-120995;OQ419012.1:121123-120962;OQ419031.1:121127-120966;OQ419047.1:121129-120968;OQ451898.1:121008-120847;OQ451900.1:121921-121760;OQ581853.1:121947-121786;OQ581855.1:122028-121867;OQ721951.1:121961-121800;OQ721954.1:121918-121757;OR035652.1:121870-121709;OR035664.1:121870-121709;OR035702.1:121870-121709;OR146272.1:121886-121725;OR146330.1:121931-121770;OR264364.1:121941-121780;OR427332.1:121941-121780;OR459777.1:121925-121764;OR459797.1:121939-121778;OR943698.1:124252-124091;PP098589.2:121922-121761;PP496048.1:121926-121765;PP601206.1:124182-124021;PP776553.1:121847-121686;PP852971.1:121920-121759;PP852983.1:121926-121765;PP853000.1:121887-121726;PP853005.1:121895-121734;PP853007.1:121928-121767;PP853013.1:121901-121740;PP853027.1:121932-121771;PP853045.1:121913-121752;PP853048.1:121932-121771;PP860026.1:121930-121769;PP860027.1:121956-121795;PP860028.1:121895-121734;PP860031.1:121949-121788;PP860033.1:121955-121794;PQ153226.1:121920-121759;PQ178862.2:123042-122881;PQ221506.1:118019-117858;PQ221833.1:124185-124024;PQ340477.1:121903-121742;PQ340478.1:121888-121727;PQ340480.1:121967-121806;PQ340483.1:121951-121790;PQ340485.1:121937-121776;PQ340486.1:121952-121791;PQ340487.1:121977-121816;PQ340489.1:121952-121791;PQ340490.1:121903-121742;PQ340491.1:121958-121797;PQ340493.1:121937-121776;PQ340495.1:121967-121806;PQ340496.1:122000-121839;PQ368489.1:121923-121762;PQ451835.1:122008-121847;PQ451836.1:121940-121779;PQ451838.1:121991-121830;PQ451841.1:121951-121790;PQ451842.1:121903-121742 |
| OG0000168 | AY603973.1:123091-122519;AY741551.1:123113-122541;AY753185.1:123334-122762;DQ011154.1:123548-122976;DQ011155.1:123529-122957;EPI_ISL_13331598:121147-120575;EPI_ISL_13363142:121140-120568;EPI_ISL_13374487:121155-120583;EPI_ISL_13502582:121140-120568;EPI_ISL_13508471:121232-120660;EPI_ISL_13607904:121131-120559;EPI_ISL_13624509:121075-120503;EPI_ISL_13889442:121250-120678;EPI_ISL_13953610:121048-120476;EPI_ISL_14181948:121276-120704;EPI_ISL_14838587:123402-122830;EPI_ISL_15263355:121131-120559;EPI_ISL_15269699:121100-120528;EPI_ISL_15293002:121373-120801;EPI_ISL_15641561:121089-120517;EPI_ISL_15831211:121138-120566;EPI_ISL_15992098:121153-120581;EPI_ISL_16233784:121149-120577;EPI_ISL_16946400:121138-120566;EPI_ISL_17048206:120788-120216;EPI_ISL_17703567:121153-120581;EPI_ISL_17718458:121131-120559;EPI_ISL_18213374:121128-120556;EPI_ISL_18360394:120789-120217;EPI_ISL_18557816:121129-120557;EPI_ISL_18642365:121131-120559;EPI_ISL_18659846:121101-120529;EPI_ISL_18744049:121133-120561;EPI_ISL_19022858:121128-120556;EPI_ISL_19028778:121045-120473;EPI_ISL_19151800:121129-120557;EPI_ISL_19159116:121131-120559;EPI_ISL_19350788:122273-121701;EPI_ISL_19359677:121128-120556;EPI_ISL_19446306:121123-120551;EPI_ISL_19467692:123684-123112;JX878411.1:124393-123821;JX878425.1:123910-123338;JX878426.1:123360-122788;KC257460.1:123327-122755;KJ642612.1:121644-121072;KJ642613.1:123204-122632;KJ642616.1:122791-122219;KJ642618.1:120875-120303;KP849469.1:123718-123146;KP849471.1:123644-123072;LC831698.1:121129-120557;MN648051.1:121351-120779;MN702445.1:117061-116489;MN702446.1:123206-122634;MN702447.1:116574-116002;MN702449.1:117072-116500;MN702452.1:123247-122675;MT903338.1:121294-120722;MT903340.1:121131-120559;MT903341.1:112664-112092;MT903342.1:121146-120574;MT903347.1:123120-122548;ON568298.1:121277-120705;ON622713.1:121363-120791;ON622722.2:121134-120562;ON631963.1:121154-120582;ON649879.1:120964-120392;ON676707.1:121184-120612;ON755039.1:120794-120222;ON808413.1:121134-120562;ON880420.1:121361-120789;ON911481.2:121151-120579;ON918656.1:121166-120594;ON959133.1:121255-120683;ON959136.1:121256-120684;ON983168.1:119829-119257;OP019276.1:121128-120556;OP150924.1:121269-120697;OP160532.1:121094-120522;OP235282.1:121128-120556;OP289782.1:121131-120559;OP331335.1:121148-120576;OP382479.1:121135-120563;OP422341.1:121148-120576;OP434519.1:121127-120555;OP535324.1:121129-120557;OP535333.1:121238-120666;OP536770.1:121130-120558;OP597769.1:121152-120580;OP604566.1:121131-120559;OP612682.1:121056-120484;OP612684.1:120804-120547;OP612686.1:121120-120548;OP696841.1:118405-117833;OP879722.1:120354-119782;OQ419007.1:120364-119792;OQ419012.1:120331-119759;OQ419031.1:120335-119763;OQ419047.1:120337-119765;OQ451898.1:120216-119644;OQ451900.1:121129-120557;OQ581853.1:121155-120583;OQ581855.1:121236-120664;OQ721951.1:121169-120597;OQ721954.1:121126-120554;OR035652.1:121078-120506;OR035664.1:121078-120506;OR035702.1:121078-120506;OR146272.1:121094-120522;OR146330.1:121139-120567;OR264364.1:121149-120577;OR427332.1:121149-120577;OR459777.1:121133-120561;OR459797.1:121147-120575;OR943698.1:123460-122888;PP098589.2:121130-120558;PP496048.1:121134-120562;PP601206.1:123390-122818;PP776553.1:121055-120483;PP852971.1:121128-120556;PP852983.1:121134-120562;PP853000.1:121095-120523;PP853005.1:121103-120531;PP853007.1:121136-120564;PP853013.1:121109-120537;PP853027.1:121140-120568;PP853045.1:121121-120549;PP853048.1:121140-120568;PP860026.1:121138-120566;PP860027.1:121164-120592;PP860028.1:121103-120531;PP860031.1:121157-120585;PP860033.1:121163-120591;PQ153226.1:121128-120556;PQ178862.2:122250-121678;PQ221506.1:117227-116655;PQ221833.1:123393-122821;PQ340477.1:121111-120539;PQ340478.1:121096-120524;PQ340480.1:121175-120603;PQ340483.1:121159-120587;PQ340485.1:121145-120573;PQ340486.1:121160-120588;PQ340487.1:121185-120613;PQ340489.1:121160-120588;PQ340490.1:121111-120539;PQ340491.1:121166-120594;PQ340493.1:121145-120573;PQ340495.1:121175-120603;PQ340496.1:121208-120636;PQ368489.1:121131-120559;PQ451835.1:121216-120644;PQ451836.1:121148-120576;PQ451838.1:121199-120627;PQ451841.1:121159-120587;PQ451842.1:121111-120539 |
| OG0000169 | AY603973.1:121546-118871;AY741551.1:121568-118893;AY753185.1:121789-119114;DQ011154.1:122003-119328;DQ011155.1:121984-119309;EPI_ISL_13331598:119602-116927;EPI_ISL_13363142:119595-116920;EPI_ISL_13374487:119610-116935;EPI_ISL_13502582:119595-116920;EPI_ISL_13508471:119687-117012;EPI_ISL_13607904:119586-116911;EPI_ISL_13624509:119530-116855;EPI_ISL_13889442:119705-117030;EPI_ISL_13953610:119503-116828;EPI_ISL_14181948:119731-117056;EPI_ISL_14838587:121857-119182;EPI_ISL_15263355:119586-116911;EPI_ISL_15269699:119555-116880;EPI_ISL_15293002:119828-117153;EPI_ISL_15641561:119544-116869;EPI_ISL_15831211:119593-116918;EPI_ISL_15992098:119608-116933;EPI_ISL_16233784:119604-116929;EPI_ISL_16946400:119593-116918;EPI_ISL_17048206:119243-116568;EPI_ISL_17703567:119608-116933;EPI_ISL_17718458:119586-116911;EPI_ISL_18213374:119583-116908;EPI_ISL_18360394:119244-116569;EPI_ISL_18557816:119584-116909;EPI_ISL_18642365:119586-116911;EPI_ISL_18659846:119556-116881;EPI_ISL_18744049:119588-116913;EPI_ISL_19022858:119583-116908;EPI_ISL_19028778:119500-116825;EPI_ISL_19151800:119584-116909;EPI_ISL_19159116:119586-116911;EPI_ISL_19350788:120728-118053;EPI_ISL_19359677:119583-116908;EPI_ISL_19446306:119578-116903;EPI_ISL_19467692:122139-119464;JX878411.1:122848-120173;JX878425.1:122365-119690;JX878426.1:121815-119140;KC257460.1:121782-119107;KJ642612.1:120099-117424;KJ642613.1:121659-118984;KJ642616.1:121246-118571;KJ642618.1:119330-116655;KP849469.1:122173-119498;KP849471.1:122099-119424;LC831698.1:119584-116909;MN648051.1:119806-117131;MN702445.1:115516-112841;MN702446.1:121661-118986;MN702447.1:115029-112354;MN702449.1:115527-112852;MN702452.1:121702-119027;MT903338.1:119749-117074;MT903340.1:119586-116911;MT903341.1:111119-108444;MT903342.1:119601-116926;MT903347.1:121575-118900;ON568298.1:119732-117057;ON622713.1:119818-117143;ON622722.2:119589-116914;ON631963.1:119609-116934;ON649879.1:119419-116744;ON676707.1:119639-116964;ON755039.1:119249-116574;ON808413.1:119589-116914;ON880420.1:119816-117141;ON911481.2:119606-116931;ON918656.1:119621-116946;ON959133.1:119710-117035;ON959136.1:119711-117036;ON983168.1:118284-115609;OP019276.1:119583-116908;OP150924.1:119724-117049;OP160532.1:119549-116874;OP235282.1:119583-116908;OP289782.1:119586-116911;OP331335.1:119603-116928;OP382479.1:119590-116915;OP422341.1:119603-116928;OP434519.1:119582-116907;OP535324.1:119584-116909;OP535333.1:119693-117018;OP536770.1:119585-116910;OP597769.1:119607-116932;OP604566.1:119586-116911;OP612682.1:119511-116836;OP612684.1:119574-116899;OP612686.1:119575-116900;OP696841.1:116860-114185;OP879722.1:118809-116134;OQ419007.1:118819-116144;OQ419012.1:118786-116111;OQ419031.1:118790-116115;OQ419047.1:118792-116117;OQ451898.1:118671-115996;OQ451900.1:119584-116909;OQ581853.1:119610-116935;OQ581855.1:119691-117016;OQ721951.1:119624-116949;OQ721954.1:119581-116906;OR035652.1:119533-116858;OR035664.1:119533-116858;OR035702.1:119533-116858;OR146272.1:119549-116874;OR146330.1:119594-116919;OR264364.1:119604-116929;OR427332.1:119604-116929;OR459777.1:119588-116913;OR459797.1:119602-116927;OR943698.1:121915-119240;PP098589.2:119585-116910;PP496048.1:119589-116914;PP601206.1:121845-119170;PP776553.1:119510-116835;PP852971.1:119583-116908;PP852983.1:119589-116914;PP853000.1:119550-116875;PP853005.1:119558-116883;PP853007.1:119591-116916;PP853013.1:119564-116889;PP853027.1:119595-116920;PP853045.1:119576-116901;PP853048.1:119595-116920;PP860026.1:119593-116918;PP860027.1:119619-116944;PP860028.1:119558-116883;PP860031.1:119612-116937;PP860033.1:119618-116943;PQ153226.1:119583-116908;PQ178862.2:120705-118030;PQ221506.1:115682-113007;PQ221833.1:121848-119173;PQ340477.1:119566-116891;PQ340478.1:119551-116876;PQ340480.1:119630-116955;PQ340483.1:119614-116939;PQ340485.1:119600-116925;PQ340486.1:119615-116940;PQ340487.1:119640-116965;PQ340489.1:119615-116940;PQ340490.1:119566-116891;PQ340491.1:119621-116946;PQ340493.1:119600-116925;PQ340495.1:119630-116955;PQ340496.1:119663-116988;PQ368489.1:119586-116911;PQ451835.1:119671-116996;PQ451836.1:119603-116928;PQ451838.1:119654-116979;PQ451841.1:119614-116939;PQ451842.1:119566-116891 |
| OG0000170 | AY603973.1:118870-118550;AY741551.1:118892-118554;AY753185.1:119113-118793;DQ011154.1:119327-119025;DQ011155.1:119308-119006;EPI_ISL_13331598:116926-116624;EPI_ISL_13363142:116919-116617;EPI_ISL_13374487:116934-116632;EPI_ISL_13502582:116919-116617;EPI_ISL_13508471:117011-116709;EPI_ISL_13607904:116910-116608;EPI_ISL_13624509:116854-116552;EPI_ISL_13889442:117029-116727;EPI_ISL_13953610:116827-116525;EPI_ISL_14181948:117055-116753;EPI_ISL_14838587:119181-118879;EPI_ISL_15263355:116910-116608;EPI_ISL_15269699:116879-116577;EPI_ISL_15293002:117152-116850;EPI_ISL_15641561:116868-116566;EPI_ISL_15831211:116917-116615;EPI_ISL_15992098:116932-116630;EPI_ISL_16233784:116928-116626;EPI_ISL_16946400:116917-116615;EPI_ISL_17048206:116567-116265;EPI_ISL_17703567:116932-116630;EPI_ISL_17718458:116910-116608;EPI_ISL_18213374:116907-116605;EPI_ISL_18360394:116568-116266;EPI_ISL_18557816:116908-116606;EPI_ISL_18642365:116910-116608;EPI_ISL_18659846:116880-116578;EPI_ISL_18744049:116912-116610;EPI_ISL_19022858:116907-116605;EPI_ISL_19028778:116824-116522;EPI_ISL_19151800:116908-116606;EPI_ISL_19159116:116910-116608;EPI_ISL_19350788:118052-117750;EPI_ISL_19359677:116907-116605;EPI_ISL_19446306:116902-116600;EPI_ISL_19467692:119463-119161;JX878411.1:120172-119870;JX878425.1:119689-119387;JX878426.1:119139-118837;KC257460.1:119106-118804;KJ642612.1:117423-117121;KJ642613.1:118983-118681;KJ642616.1:118570-118244;KJ642618.1:116654-116352;KP849469.1:119497-119195;KP849471.1:119423-119121;LC831698.1:116908-116606;MN648051.1:117130-116828;MN702445.1:112840-112538;MN702446.1:118985-118683;MN702447.1:112353-112051;MN702449.1:112851-112549;MN702452.1:119026-118724;MT903338.1:117073-116771;MT903340.1:116910-116608;MT903341.1:108443-108141;MT903342.1:116925-116623;MT903347.1:118899-118561;ON568298.1:117056-116754;ON622713.1:117142-116840;ON622722.2:116913-116611;ON631963.1:116933-116631;ON649879.1:116743-116441;ON676707.1:116963-116661;ON755039.1:116573-116271;ON808413.1:116913-116611;ON880420.1:117140-116838;ON911481.2:116930-116628;ON918656.1:116945-116643;ON959133.1:117034-116732;ON959136.1:117035-116733;ON983168.1:115608-115306;OP019276.1:116907-116605;OP150924.1:117048-116746;OP160532.1:116873-116571;OP235282.1:116907-116605;OP289782.1:116910-116608;OP331335.1:116927-116625;OP382479.1:116914-116612;OP422341.1:116927-116625;OP434519.1:116906-116604;OP535324.1:116908-116606;OP535333.1:117017-116715;OP536770.1:116909-116607;OP597769.1:116931-116629;OP604566.1:116910-116608;OP612682.1:116835-116533;OP612684.1:116898-116596;OP612686.1:116899-116597;OP696841.1:114184-113882;OP879722.1:116133-115831;OQ419007.1:116143-115841;OQ419012.1:116110-115808;OQ419031.1:116114-115812;OQ419047.1:116116-115814;OQ451898.1:115995-115693;OQ451900.1:116908-116606;OQ581853.1:116934-116632;OQ581855.1:117015-116713;OQ721951.1:116948-116646;OQ721954.1:116905-116603;OR035652.1:116857-116555;OR035664.1:116857-116555;OR035702.1:116857-116555;OR146272.1:116873-116571;OR146330.1:116918-116616;OR264364.1:116928-116626;OR427332.1:116928-116626;OR459777.1:116912-116610;OR459797.1:116926-116624;OR943698.1:119239-118937;PP098589.2:116909-116607;PP496048.1:116913-116611;PP601206.1:119169-118867;PP776553.1:116834-116532;PP852971.1:116907-116605;PP852983.1:116913-116611;PP853000.1:116874-116572;PP853005.1:116882-116580;PP853007.1:116915-116613;PP853013.1:116888-116586;PP853027.1:116919-116617;PP853045.1:116900-116598;PP853048.1:116919-116617;PP860026.1:116917-116615;PP860027.1:116943-116641;PP860028.1:116882-116580;PP860031.1:116936-116634;PP860033.1:116942-116640;PQ153226.1:116907-116605;PQ178862.2:118029-117727;PQ221506.1:113006-112704;PQ221833.1:119172-118870;PQ340477.1:116890-116588;PQ340478.1:116875-116573;PQ340480.1:116954-116652;PQ340483.1:116938-116636;PQ340485.1:116924-116622;PQ340486.1:116939-116637;PQ340487.1:116964-116662;PQ340489.1:116939-116637;PQ340490.1:116890-116588;PQ340491.1:116945-116643;PQ340493.1:116924-116622;PQ340495.1:116954-116652;PQ340496.1:116987-116685;PQ368489.1:116910-116608;PQ451835.1:116995-116693;PQ451836.1:116927-116625;PQ451838.1:116978-116676;PQ451841.1:116938-116636;PQ451842.1:116890-116588 |
| OG0000171 | AY603973.1:117637-115505;AY741551.1:117641-115509;AY753185.1:117880-115748;DQ011154.1:118112-115980;DQ011155.1:118093-115961;EPI_ISL_13331598:115711-113579;EPI_ISL_13363142:115704-113572;EPI_ISL_13374487:115719-113587;EPI_ISL_13502582:115704-113572;EPI_ISL_13508471:115796-113664;EPI_ISL_13607904:115695-113563;EPI_ISL_13624509:115639-113507;EPI_ISL_13889442:115814-113682;EPI_ISL_13953610:115612-113480;EPI_ISL_14181948:115840-113708;EPI_ISL_14838587:117966-115834;EPI_ISL_15263355:115695-113563;EPI_ISL_15269699:115664-113532;EPI_ISL_15293002:115937-113805;EPI_ISL_15641561:115653-113521;EPI_ISL_15831211:115702-113570;EPI_ISL_15992098:115717-113585;EPI_ISL_16233784:115713-113581;EPI_ISL_16946400:115702-113570;EPI_ISL_17048206:115352-113220;EPI_ISL_17703567:115717-113585;EPI_ISL_17718458:115695-113563;EPI_ISL_18213374:115692-113560;EPI_ISL_18360394:115353-113221;EPI_ISL_18557816:115693-113561;EPI_ISL_18642365:115695-113563;EPI_ISL_18659846:115665-113533;EPI_ISL_18744049:115697-113565;EPI_ISL_19022858:115692-113560;EPI_ISL_19028778:115609-113477;EPI_ISL_19151800:115693-113561;EPI_ISL_19159116:115695-113563;EPI_ISL_19350788:116837-114705;EPI_ISL_19359677:115692-113560;EPI_ISL_19446306:115687-113555;EPI_ISL_19467692:118248-116116;JX878411.1:118957-116825;JX878425.1:118474-116342;JX878426.1:117924-115792;KC257460.1:117891-115759;KJ642612.1:116208-114076;KJ642613.1:117768-115636;KJ642616.1:117331-115199;KJ642618.1:115439-113307;KP849469.1:118282-116150;KP849471.1:118208-116076;LC831698.1:115693-113561;MN648051.1:115915-113783;MN702445.1:111625-109493;MN702446.1:117770-115638;MN702447.1:111138-109006;MN702449.1:111636-109504;MN702452.1:117811-115679;MT903338.1:115858-113726;MT903340.1:115695-113563;MT903341.1:107228-105096;MT903342.1:115710-113578;MT903347.1:117648-115516;ON568298.1:115841-113709;ON622713.1:115927-113795;ON622722.2:115698-113566;ON631963.1:115718-113586;ON649879.1:115528-113396;ON676707.1:115748-113616;ON755039.1:115358-113226;ON808413.1:115698-113566;ON880420.1:115925-113793;ON911481.2:115715-113583;ON918656.1:115730-113598;ON959133.1:115819-113687;ON959136.1:115820-113688;ON983168.1:114393-112261;OP019276.1:115692-113560;OP150924.1:115833-113701;OP160532.1:115658-113526;OP235282.1:115692-113560;OP289782.1:115695-113563;OP331335.1:115712-113580;OP382479.1:115699-113567;OP422341.1:115712-113580;OP434519.1:115691-113559;OP535324.1:115693-113561;OP535333.1:115802-113670;OP536770.1:115694-113562;OP597769.1:115716-113584;OP604566.1:115695-113563;OP612682.1:115620-113488;OP612684.1:115683-113551;OP612686.1:115684-113552;OP696841.1:112969-110837;OP879722.1:114918-112786;OQ419007.1:114928-112796;OQ419012.1:114895-112763;OQ419031.1:114899-112767;OQ419047.1:114901-112769;OQ451898.1:114780-112648;OQ451900.1:115693-113561;OQ581853.1:115719-113587;OQ581855.1:115800-113668;OQ721951.1:115733-113601;OQ721954.1:115690-113558;OR035652.1:115642-113510;OR035664.1:115642-113510;OR035702.1:115642-113510;OR146272.1:115658-113526;OR146330.1:115703-113571;OR264364.1:115713-113581;OR427332.1:115713-113581;OR459777.1:115697-113565;OR459797.1:115711-113909;OR943698.1:118024-115892;PP098589.2:115694-113562;PP496048.1:115698-113566;PP601206.1:117954-115822;PP776553.1:115619-113487;PP852971.1:115692-113560;PP852983.1:115698-113566;PP853000.1:115659-113527;PP853005.1:115667-113535;PP853007.1:115700-113568;PP853013.1:115673-113541;PP853027.1:115704-113572;PP853045.1:115685-113553;PP853048.1:115704-113572;PP860026.1:115702-113570;PP860027.1:115728-113596;PP860028.1:115667-113535;PP860031.1:115721-113589;PP860033.1:115727-113595;PQ153226.1:115692-113560;PQ178862.2:116814-114682;PQ221506.1:111791-109659;PQ221833.1:117957-115825;PQ340477.1:115675-113543;PQ340478.1:115660-113528;PQ340480.1:115739-113607;PQ340483.1:115723-113591;PQ340485.1:115709-113577;PQ340486.1:115724-113592;PQ340487.1:115749-113617;PQ340489.1:115724-113592;PQ340490.1:115675-113543;PQ340491.1:115730-113598;PQ340493.1:115709-113577;PQ340495.1:115739-113607;PQ340496.1:115772-113640;PQ368489.1:115695-113563;PQ451835.1:115780-113648;PQ451836.1:115712-113580;PQ451838.1:115763-113631;PQ451841.1:115723-113591;PQ451842.1:115675-113543 |
| OG0000172 | AY603973.1:112945-111011;AY741551.1:112949-111015;AY753185.1:113188-111254;DQ011154.1:113420-111486;DQ011155.1:113401-111467;EPI_ISL_13331598:111019-109085;EPI_ISL_13363142:111012-109078;EPI_ISL_13374487:111027-109093;EPI_ISL_13502582:111012-109078;EPI_ISL_13508471:111104-109170;EPI_ISL_13607904:111003-109069;EPI_ISL_13624509:110947-109013;EPI_ISL_13889442:111122-109188;EPI_ISL_13953610:110920-108986;EPI_ISL_14181948:111148-109214;EPI_ISL_14838587:113274-111340;EPI_ISL_15263355:111003-109069;EPI_ISL_15269699:110972-109038;EPI_ISL_15293002:111245-109311;EPI_ISL_15641561:110961-109027;EPI_ISL_15831211:111010-109076;EPI_ISL_15992098:111025-109091;EPI_ISL_16233784:111021-109087;EPI_ISL_16946400:111010-109076;EPI_ISL_17048206:110660-108726;EPI_ISL_17703567:111025-109091;EPI_ISL_17718458:111003-109069;EPI_ISL_18213374:111000-109066;EPI_ISL_18360394:110661-108727;EPI_ISL_18557816:111001-109067;EPI_ISL_18642365:111003-109069;EPI_ISL_18659846:110973-109039;EPI_ISL_18744049:111005-109071;EPI_ISL_19022858:111000-109066;EPI_ISL_19028778:110917-108983;EPI_ISL_19151800:111001-109067;EPI_ISL_19159116:111003-109069;EPI_ISL_19350788:112145-110211;EPI_ISL_19359677:111000-109066;EPI_ISL_19446306:110995-109061;EPI_ISL_19467692:113556-111622;JX878411.1:114265-112331;JX878425.1:113782-111848;JX878426.1:113232-111298;KC257460.1:113199-111265;KJ642612.1:111516-109582;KJ642613.1:113076-111142;KJ642616.1:112639-110705;KJ642618.1:110747-108813;KP849469.1:113590-111656;KP849471.1:113516-111582;LC831698.1:111001-109067;MN648051.1:111223-109289;MN702445.1:106933-104999;MN702446.1:113078-111144;MN702447.1:106446-104512;MN702449.1:106944-105010;MN702452.1:113119-111185;MT903338.1:111166-109232;MT903340.1:111003-109069;MT903341.1:102536-100602;MT903342.1:111018-109084;MT903347.1:112956-111022;ON568298.1:111149-109215;ON622713.1:111235-109301;ON622722.2:111006-109072;ON631963.1:111026-109092;ON649879.1:110836-108902;ON676707.1:111056-109122;ON755039.1:110666-108732;ON808413.1:111006-109072;ON880420.1:111233-109299;ON911481.2:111023-109089;ON918656.1:111038-109104;ON959133.1:111127-109193;ON959136.1:111128-109194;ON983168.1:109701-107767;OP019276.1:111000-109066;OP150924.1:111141-109207;OP160532.1:110966-109032;OP235282.1:111000-109066;OP289782.1:111003-109069;OP331335.1:111020-109086;OP382479.1:111007-109073;OP422341.1:111020-109086;OP434519.1:110999-109065;OP535324.1:111001-109067;OP535333.1:111110-109176;OP536770.1:111002-109068;OP597769.1:111024-109090;OP604566.1:111003-109069;OP612682.1:110928-108994;OP612684.1:110991-109057;OP612686.1:110992-109058;OP696841.1:108277-106343;OP879722.1:110226-108292;OQ419007.1:110236-108302;OQ419012.1:110203-108269;OQ419031.1:110207-108273;OQ419047.1:110209-108275;OQ451898.1:110088-108154;OQ451900.1:111001-109067;OQ581853.1:111027-109093;OQ581855.1:111108-109174;OQ721951.1:111041-109107;OQ721954.1:110998-109064;OR035652.1:110950-109016;OR035664.1:110950-109016;OR035702.1:110950-109016;OR146272.1:110966-109032;OR146330.1:111011-109077;OR264364.1:111021-109087;OR427332.1:111021-109087;OR459777.1:111005-109071;OR459797.1:111020-109086;OR943698.1:113332-111398;PP098589.2:111002-109068;PP496048.1:111006-109072;PP601206.1:113262-111328;PP776553.1:110927-108993;PP852971.1:111000-109066;PP852983.1:111006-109072;PP853000.1:110967-109033;PP853005.1:110975-109041;PP853007.1:111008-109074;PP853013.1:110981-109047;PP853027.1:111012-109078;PP853045.1:110993-109059;PP853048.1:111012-109078;PP860026.1:111010-109076;PP860027.1:111036-109102;PP860028.1:110975-109041;PP860031.1:111029-109095;PP860033.1:111035-109101;PQ153226.1:111000-109066;PQ178862.2:112122-110188;PQ221506.1:107099-105165;PQ221833.1:113265-111331;PQ340477.1:110983-109049;PQ340478.1:110968-109034;PQ340480.1:111047-109113;PQ340483.1:111031-109097;PQ340485.1:111017-109083;PQ340486.1:111032-109098;PQ340487.1:111057-109123;PQ340489.1:111032-109098;PQ340490.1:110983-109049;PQ340491.1:111038-109104;PQ340493.1:111017-109083;PQ340495.1:111047-109113;PQ340496.1:111080-109146;PQ368489.1:111003-109069;PQ451835.1:111088-109154;PQ451836.1:111020-109086;PQ451838.1:111071-109137;PQ451841.1:111031-109097;PQ451842.1:110983-109049 |
| OG0000173 | AY603973.1:110071-109619;AY741551.1:110075-109623;AY753185.1:110314-109862;DQ011154.1:110546-110094;DQ011155.1:110527-110075;EPI_ISL_13331598:108145-107693;EPI_ISL_13363142:108138-107686;EPI_ISL_13374487:108153-107701;EPI_ISL_13502582:108138-107686;EPI_ISL_13508471:108230-107778;EPI_ISL_13607904:108129-107677;EPI_ISL_13624509:108073-107621;EPI_ISL_13889442:108248-107796;EPI_ISL_13953610:108046-107594;EPI_ISL_14181948:108274-107822;EPI_ISL_14838587:110400-109948;EPI_ISL_15263355:108129-107677;EPI_ISL_15269699:108098-107646;EPI_ISL_15293002:108371-107919;EPI_ISL_15641561:108087-107635;EPI_ISL_15831211:108136-107684;EPI_ISL_15992098:108151-107699;EPI_ISL_16233784:108147-107695;EPI_ISL_16946400:108136-107684;EPI_ISL_17048206:107786-107334;EPI_ISL_17703567:108151-107699;EPI_ISL_17718458:108129-107677;EPI_ISL_18213374:108126-107674;EPI_ISL_18360394:107787-107335;EPI_ISL_18557816:108127-107675;EPI_ISL_18642365:108129-107677;EPI_ISL_18659846:108099-107647;EPI_ISL_18744049:108131-107679;EPI_ISL_19022858:108126-107674;EPI_ISL_19028778:108043-107591;EPI_ISL_19151800:108127-107675;EPI_ISL_19159116:108129-107677;EPI_ISL_19350788:109271-108819;EPI_ISL_19359677:108126-107674;EPI_ISL_19446306:108121-107669;EPI_ISL_19467692:110682-110230;JX878411.1:111391-110939;JX878425.1:110908-110456;JX878426.1:110357-109905;KC257460.1:110325-109873;KJ642612.1:108642-108190;KJ642613.1:110202-109750;KJ642616.1:109765-109313;KJ642618.1:107873-107421;KP849469.1:110716-110264;KP849471.1:110642-110190;LC831698.1:108127-107675;MN648051.1:108349-107897;MN702445.1:104059-103607;MN702446.1:110204-109752;MN702447.1:103572-103120;MN702449.1:104070-103618;MN702452.1:110245-109793;MT903338.1:108292-107840;MT903340.1:108129-107677;MT903341.1:99662-99210;MT903342.1:108144-107692;MT903347.1:110082-109630;ON568298.1:108275-107823;ON622713.1:108361-107909;ON622722.2:108132-107680;ON631963.1:108152-107700;ON649879.1:107962-107510;ON676707.1:108182-107730;ON755039.1:107792-107340;ON808413.1:108132-107680;ON880420.1:108359-107907;ON911481.2:108149-107697;ON918656.1:108164-107712;ON959133.1:108253-107801;ON959136.1:108254-107802;ON983168.1:106827-106375;OP019276.1:108126-107674;OP150924.1:108267-107815;OP160532.1:108092-107640;OP235282.1:108126-107674;OP289782.1:108129-107677;OP331335.1:108146-107694;OP382479.1:108133-107681;OP422341.1:108146-107694;OP434519.1:108125-107673;OP535324.1:108127-107675;OP535333.1:108236-107784;OP536770.1:108128-107676;OP597769.1:108150-107698;OP604566.1:108129-107677;OP612682.1:108054-107602;OP612684.1:108117-107665;OP612686.1:108118-107666;OP696841.1:105403-104951;OP879722.1:107352-106900;OQ419007.1:107362-106910;OQ419012.1:107329-106877;OQ419031.1:107333-106881;OQ419047.1:107335-106883;OQ451898.1:107214-106762;OQ451900.1:108127-107675;OQ581853.1:108153-107701;OQ581855.1:108234-107782;OQ721951.1:108167-107715;OQ721954.1:108124-107672;OR035652.1:108076-107624;OR035664.1:108076-107624;OR035702.1:108076-107624;OR146272.1:108092-107640;OR146330.1:108137-107685;OR264364.1:108147-107695;OR427332.1:108147-107695;OR459777.1:108131-107679;OR459797.1:108146-107694;OR943698.1:110458-110006;PP098589.2:108128-107676;PP496048.1:108132-107680;PP601206.1:110388-109936;PP776553.1:108053-107601;PP852971.1:108126-107674;PP852983.1:108132-107680;PP853000.1:108093-107641;PP853005.1:108101-107649;PP853007.1:108134-107682;PP853013.1:108107-107655;PP853027.1:108138-107686;PP853045.1:108119-107667;PP853048.1:108138-107686;PP860026.1:108136-107684;PP860027.1:108161-107709;PP860028.1:108101-107649;PP860031.1:108155-107703;PP860033.1:108160-107708;PQ153226.1:108126-107674;PQ178862.2:109248-108796;PQ221506.1:104225-103773;PQ221833.1:110391-109939;PQ340477.1:108109-107657;PQ340478.1:108094-107642;PQ340480.1:108173-107721;PQ340483.1:108157-107705;PQ340485.1:108143-107691;PQ340486.1:108158-107706;PQ340487.1:108183-107731;PQ340489.1:108158-107706;PQ340490.1:108109-107657;PQ340491.1:108164-107712;PQ340493.1:108143-107691;PQ340495.1:108173-107721;PQ340496.1:108206-107754;PQ368489.1:108129-107677;PQ451835.1:108214-107762;PQ451836.1:108146-107694;PQ451838.1:108197-107745;PQ451841.1:108157-107705;PQ451842.1:108109-107657 |
| OG0000174 | AY603973.1:107011-105116;AY741551.1:107015-105120;AY753185.1:107254-105359;DQ011154.1:107485-105590;DQ011155.1:107466-105571;EPI_ISL_13331598:105085-103190;EPI_ISL_13363142:105078-103183;EPI_ISL_13374487:105093-103198;EPI_ISL_13502582:105078-103183;EPI_ISL_13508471:105170-103275;EPI_ISL_13607904:105069-103174;EPI_ISL_13624509:105013-103118;EPI_ISL_13889442:105188-103293;EPI_ISL_13953610:104986-103091;EPI_ISL_14181948:105214-103319;EPI_ISL_14838587:107339-105444;EPI_ISL_15263355:105069-103174;EPI_ISL_15269699:105038-103143;EPI_ISL_15293002:105311-103416;EPI_ISL_15641561:105027-103132;EPI_ISL_15831211:105076-103181;EPI_ISL_15992098:105091-103196;EPI_ISL_16233784:105087-103192;EPI_ISL_16946400:105076-103181;EPI_ISL_17048206:104726-102831;EPI_ISL_17703567:105091-103196;EPI_ISL_17718458:105069-103174;EPI_ISL_18213374:105066-103171;EPI_ISL_18360394:104727-102832;EPI_ISL_18557816:105067-103172;EPI_ISL_18642365:105069-103174;EPI_ISL_18659846:105039-103144;EPI_ISL_18744049:105071-103176;EPI_ISL_19022858:105066-103171;EPI_ISL_19028778:104983-103088;EPI_ISL_19151800:105067-103172;EPI_ISL_19159116:105069-103174;EPI_ISL_19350788:106211-104316;EPI_ISL_19359677:105066-103171;EPI_ISL_19446306:105061-103166;EPI_ISL_19467692:107622-105727;JX878411.1:108330-106435;JX878425.1:107847-105952;JX878426.1:107296-105401;KC257460.1:107264-105369;KJ642612.1:105582-103687;KJ642613.1:107141-105246;KJ642616.1:106705-104810;KJ642618.1:104812-102917;KP849469.1:107655-105760;KP849471.1:107581-105686;LC831698.1:105067-103172;MN648051.1:105289-103394;MN702445.1:100998-99103;MN702446.1:107143-105248;MN702447.1:100511-98616;MN702449.1:101009-99114;MN702452.1:107184-105289;MT903338.1:105232-103337;MT903340.1:105069-103174;MT903341.1:96602-94707;MT903342.1:105084-103189;MT903347.1:107022-105127;ON568298.1:105215-103320;ON622713.1:105301-103406;ON622722.2:105072-103177;ON631963.1:105092-103197;ON649879.1:104902-103007;ON676707.1:105122-103227;ON755039.1:104732-102837;ON808413.1:105072-103177;ON880420.1:105299-103404;ON911481.2:105089-103194;ON918656.1:105104-103209;ON959133.1:105193-103298;ON959136.1:105194-103299;ON983168.1:103767-101872;OP019276.1:105066-103171;OP150924.1:105207-103312;OP160532.1:105032-103137;OP235282.1:105066-103171;OP289782.1:105069-103174;OP331335.1:105086-103191;OP382479.1:105073-103178;OP422341.1:105086-103191;OP434519.1:105065-103170;OP535324.1:105067-103172;OP535333.1:105176-103281;OP536770.1:105068-103173;OP597769.1:105090-103195;OP604566.1:105069-103174;OP612682.1:104994-103099;OP612684.1:105057-103162;OP612686.1:105058-103163;OP696841.1:102343-100448;OP879722.1:104292-102397;OQ419007.1:104302-102407;OQ419012.1:104269-102374;OQ419031.1:104273-102378;OQ419047.1:104275-102380;OQ451898.1:104154-102259;OQ451900.1:105067-103172;OQ581853.1:105093-103198;OQ581855.1:105174-103279;OQ721951.1:105107-103212;OQ721954.1:105064-103169;OR035652.1:105016-103121;OR035664.1:105016-103121;OR035702.1:105016-103121;OR146272.1:105032-103137;OR146330.1:105077-103182;OR264364.1:105087-103192;OR427332.1:105087-103192;OR459777.1:105071-103176;OR459797.1:105086-103191;OR943698.1:107397-105502;PP098589.2:105068-103173;PP496048.1:105072-103177;PP601206.1:107328-105433;PP776553.1:104993-103098;PP852971.1:105066-103171;PP852983.1:105072-103177;PP853000.1:105033-103138;PP853005.1:105041-103146;PP853007.1:105074-103179;PP853013.1:105047-103152;PP853027.1:105078-103183;PP853045.1:105059-103164;PP853048.1:105078-103183;PP860026.1:105076-103181;PP860027.1:105101-103206;PP860028.1:105041-103146;PP860031.1:105095-103200;PP860033.1:105100-103205;PQ153226.1:105066-103171;PQ178862.2:106188-104293;PQ221506.1:101164-99269;PQ221833.1:107330-105435;PQ340477.1:105049-103154;PQ340478.1:105034-103139;PQ340480.1:105113-103218;PQ340483.1:105097-103202;PQ340485.1:105083-103188;PQ340486.1:105098-103203;PQ340487.1:105123-103228;PQ340489.1:105098-103203;PQ340490.1:105049-103154;PQ340491.1:105104-103209;PQ340493.1:105083-103188;PQ340495.1:105113-103218;PQ340496.1:105146-103251;PQ368489.1:105069-103174;PQ451835.1:105154-103259;PQ451836.1:105086-103191;PQ451838.1:105137-103242;PQ451841.1:105097-103202;PQ451842.1:105049-103154 |
| OG0000175 | AY603973.1:87445-86930;AY741551.1:87449-86934;AY753185.1:87688-87173;DQ011154.1:87904-87389;DQ011155.1:87885-87370;EPI_ISL_13331598:85513-84998;EPI_ISL_13363142:85506-84991;EPI_ISL_13374487:85521-85006;EPI_ISL_13502582:85506-84991;EPI_ISL_13508471:85598-85083;EPI_ISL_13607904:85497-84982;EPI_ISL_13624509:85441-84926;EPI_ISL_13889442:85616-85101;EPI_ISL_13953610:85433-84918;EPI_ISL_14181948:85642-85127;EPI_ISL_14838587:87758-87243;EPI_ISL_15263355:85497-84982;EPI_ISL_15269699:85466-84951;EPI_ISL_15293002:85739-85224;EPI_ISL_15641561:85455-84940;EPI_ISL_15831211:85504-84989;EPI_ISL_15992098:85519-85004;EPI_ISL_16233784:85515-85000;EPI_ISL_16946400:85504-84989;EPI_ISL_17048206:85154-84639;EPI_ISL_17703567:85519-85004;EPI_ISL_17718458:85497-84982;EPI_ISL_18213374:85494-84979;EPI_ISL_18360394:85155-84640;EPI_ISL_18557816:85495-84980;EPI_ISL_18642365:85497-84982;EPI_ISL_18659846:85467-84952;EPI_ISL_18744049:85499-84984;EPI_ISL_19022858:85494-84979;EPI_ISL_19028778:85411-84896;EPI_ISL_19151800:85495-84980;EPI_ISL_19159116:85497-84982;EPI_ISL_19350788:86630-86115;EPI_ISL_19359677:85494-84979;EPI_ISL_19446306:85489-84974;EPI_ISL_19467692:88041-87526;JX878411.1:88749-88234;JX878425.1:88266-87751;JX878426.1:87715-87200;KC257460.1:87683-87168;KJ642612.1:86001-85486;KJ642613.1:87560-87045;KJ642616.1:87139-86624;KJ642618.1:85231-84716;KP849469.1:88074-87559;KP849471.1:88000-87485;LC831698.1:85495-84980;MN648051.1:85717-85202;MN702445.1:81417-80902;MN702446.1:87562-87047;MN702447.1:80930-80415;MN702449.1:81428-80913;MN702452.1:87603-87088;MT903338.1:85660-85145;MT903340.1:85497-84982;MT903341.1:77030-76515;MT903342.1:85512-84997;MT903347.1:87456-86941;ON568298.1:85643-85128;ON622713.1:85729-85214;ON622722.2:85500-84985;ON631963.1:85520-85005;ON649879.1:85330-84815;ON676707.1:85550-85035;ON755039.1:85160-84645;ON808413.1:85500-84985;ON880420.1:85727-85212;ON911481.2:85517-85002;ON918656.1:85532-85017;ON959133.1:85621-85106;ON959136.1:85622-85107;ON983168.1:84195-83680;OP019276.1:85494-84979;OP150924.1:85635-85120;OP160532.1:85460-84945;OP235282.1:85494-84979;OP289782.1:85497-84982;OP331335.1:85514-84999;OP382479.1:85501-84986;OP422341.1:85514-84999;OP434519.1:85493-84978;OP535324.1:85495-84980;OP535333.1:85604-85089;OP536770.1:85496-84981;OP597769.1:85518-85003;OP604566.1:85497-84982;OP612682.1:85422-84907;OP612684.1:85486-84971;OP612686.1:85486-84971;OP696841.1:82771-82256;OP879722.1:84720-84205;OQ419007.1:84730-84215;OQ419012.1:84697-84182;OQ419031.1:84701-84186;OQ419047.1:84703-84188;OQ451898.1:84582-84067;OQ451900.1:85495-84980;OQ581853.1:85521-85006;OQ581855.1:85602-85087;OQ721951.1:85535-85020;OQ721954.1:85492-84977;OR035652.1:85444-84929;OR035664.1:85444-84929;OR035702.1:85444-84929;OR146272.1:85460-84945;OR146330.1:85505-84990;OR264364.1:85515-85000;OR427332.1:85515-85000;OR459777.1:85499-84984;OR459797.1:85514-84999;OR943698.1:87807-87292;PP098589.2:85496-84981;PP496048.1:85500-84985;PP601206.1:87756-87241;PP776553.1:85421-84906;PP852971.1:85494-84979;PP852983.1:85500-84985;PP853000.1:85461-84946;PP853005.1:85469-84954;PP853007.1:85502-84987;PP853013.1:85475-84960;PP853027.1:85506-84991;PP853045.1:85487-84972;PP853048.1:85506-84991;PP860026.1:85504-84989;PP860027.1:85529-85014;PP860028.1:85469-84954;PP860031.1:85523-85008;PP860033.1:85528-85013;PQ153226.1:85494-84979;PQ178862.2:86607-86092;PQ221506.1:81583-81068;PQ221833.1:87749-87234;PQ340477.1:85477-84962;PQ340478.1:85462-84947;PQ340480.1:85541-85026;PQ340483.1:85525-85010;PQ340485.1:85511-84996;PQ340486.1:85526-85011;PQ340487.1:85551-85036;PQ340489.1:85526-85011;PQ340490.1:85477-84962;PQ340491.1:85532-85017;PQ340493.1:85511-84996;PQ340495.1:85541-85026;PQ340496.1:85574-85059;PQ368489.1:85497-84982;PQ451835.1:85582-85067;PQ451836.1:85514-84999;PQ451838.1:85565-85050;PQ451841.1:85525-85010;PQ451842.1:85477-84962 |
| OG0000176 | AY603973.1:82966-82565;AY741551.1:82970-82569;AY753185.1:83209-82808;DQ011154.1:83425-83024;DQ011155.1:83406-83005;EPI_ISL_13331598:81034-80633;EPI_ISL_13363142:81027-80626;EPI_ISL_13374487:81042-80641;EPI_ISL_13502582:81027-80626;EPI_ISL_13508471:81119-80718;EPI_ISL_13607904:81018-80617;EPI_ISL_13624509:80962-80561;EPI_ISL_13889442:81137-80736;EPI_ISL_13953610:80954-80553;EPI_ISL_14181948:81163-80762;EPI_ISL_14838587:83279-82878;EPI_ISL_15263355:81018-80617;EPI_ISL_15269699:80987-80586;EPI_ISL_15293002:81260-80859;EPI_ISL_15641561:80976-80575;EPI_ISL_15831211:81025-80624;EPI_ISL_15992098:81040-80639;EPI_ISL_16233784:81036-80635;EPI_ISL_16946400:81025-80624;EPI_ISL_17048206:80675-80274;EPI_ISL_17703567:81040-80639;EPI_ISL_17718458:81018-80617;EPI_ISL_18213374:81015-80614;EPI_ISL_18360394:80676-80275;EPI_ISL_18557816:81016-80615;EPI_ISL_18642365:81018-80617;EPI_ISL_18659846:80988-80587;EPI_ISL_18744049:81020-80619;EPI_ISL_19022858:81015-80614;EPI_ISL_19028778:80932-80531;EPI_ISL_19151800:81016-80615;EPI_ISL_19159116:81018-80617;EPI_ISL_19350788:82151-81750;EPI_ISL_19359677:81015-80614;EPI_ISL_19446306:81010-80609;EPI_ISL_19467692:83562-83161;JX878411.1:84270-83869;JX878425.1:83787-83386;JX878426.1:83236-82835;KC257460.1:83204-82803;KJ642612.1:81522-81121;KJ642613.1:83081-82680;KJ642616.1:82660-82259;KJ642618.1:80752-80351;KP849469.1:83595-83194;KP849471.1:83521-83120;LC831698.1:81016-80615;MN648051.1:81238-80837;MN702445.1:76938-76537;MN702446.1:83083-82682;MN702447.1:76451-76050;MN702449.1:76949-76548;MN702452.1:83124-82723;MT903338.1:81181-80780;MT903340.1:81018-80617;MT903341.1:72551-72150;MT903342.1:81033-80632;MT903347.1:82977-82576;ON568298.1:81164-80763;ON622713.1:81250-80849;ON622722.2:81021-80620;ON631963.1:81041-80640;ON649879.1:80851-80450;ON676707.1:81071-80670;ON755039.1:80681-80280;ON808413.1:81021-80620;ON880420.1:81248-80847;ON911481.2:81038-80637;ON918656.1:81053-80652;ON959133.1:81142-80741;ON959136.1:81143-80742;ON983168.1:79716-79468;OP019276.1:81015-80614;OP150924.1:81156-80755;OP160532.1:80981-80580;OP235282.1:81015-80614;OP289782.1:81018-80617;OP331335.1:81035-80634;OP382479.1:81022-80621;OP422341.1:81035-80634;OP434519.1:81014-80613;OP535324.1:81016-80615;OP535333.1:81125-80724;OP536770.1:81017-80616;OP597769.1:81039-80638;OP604566.1:81018-80617;OP612682.1:80943-80542;OP612684.1:81007-80606;OP612686.1:81007-80606;OP696841.1:78292-77891;OP879722.1:80241-79840;OQ419007.1:80251-79850;OQ419012.1:80218-79817;OQ419031.1:80222-79821;OQ419047.1:80224-79823;OQ451898.1:80103-79702;OQ451900.1:81016-80615;OQ581853.1:81042-80641;OQ581855.1:81123-80722;OQ721951.1:81056-80655;OQ721954.1:81013-80612;OR035652.1:80965-80564;OR035664.1:80965-80564;OR035702.1:80965-80564;OR146272.1:80981-80580;OR146330.1:81026-80625;OR264364.1:81036-80635;OR427332.1:81036-80635;OR459777.1:81020-80619;OR459797.1:81035-80634;OR943698.1:83328-82927;PP098589.2:81017-80616;PP496048.1:81021-80620;PP601206.1:83277-82876;PP776553.1:80942-80541;PP852971.1:81015-80614;PP852983.1:81021-80620;PP853000.1:80982-80581;PP853005.1:80990-80589;PP853007.1:81023-80622;PP853013.1:80996-80595;PP853027.1:81027-80626;PP853045.1:81008-80607;PP853048.1:81027-80626;PP860026.1:81025-80624;PP860027.1:81050-80649;PP860028.1:80990-80589;PP860031.1:81044-80643;PP860033.1:81049-80648;PQ153226.1:81015-80614;PQ178862.2:82128-81727;PQ221506.1:77104-76703;PQ221833.1:83270-82869;PQ340477.1:80998-80597;PQ340478.1:80983-80582;PQ340480.1:81062-80661;PQ340483.1:81046-80645;PQ340485.1:81032-80631;PQ340486.1:81047-80646;PQ340487.1:81072-80671;PQ340489.1:81047-80646;PQ340490.1:80998-80597;PQ340491.1:81053-80652;PQ340493.1:81032-80631;PQ340495.1:81062-80661;PQ340496.1:81095-80694;PQ368489.1:81018-80617;PQ451835.1:81103-80702;PQ451836.1:81035-80634;PQ451838.1:81086-80685;PQ451841.1:81046-80645;PQ451842.1:80998-80597 |
| OG0000177 | AY603973.1:78820-77786;AY741551.1:78824-77790;AY753185.1:79063-78029;DQ011154.1:79281-78247;DQ011155.1:79262-78228;EPI_ISL_13331598:76890-75856;EPI_ISL_13363142:76883-75849;EPI_ISL_13374487:76898-75864;EPI_ISL_13502582:76883-75849;EPI_ISL_13508471:76975-75941;EPI_ISL_13607904:76874-75840;EPI_ISL_13624509:76818-75784;EPI_ISL_13889442:76993-75959;EPI_ISL_13953610:76810-75776;EPI_ISL_14181948:77019-75985;EPI_ISL_14838587:79135-78101;EPI_ISL_15263355:76874-75840;EPI_ISL_15269699:76843-75809;EPI_ISL_15293002:77116-76082;EPI_ISL_15641561:76832-75798;EPI_ISL_15831211:76881-75847;EPI_ISL_15992098:76896-75862;EPI_ISL_16233784:76892-75858;EPI_ISL_16946400:76881-75847;EPI_ISL_17048206:76531-75497;EPI_ISL_17703567:76896-75862;EPI_ISL_17718458:76874-75840;EPI_ISL_18213374:76871-75837;EPI_ISL_18360394:76532-75498;EPI_ISL_18557816:76872-75838;EPI_ISL_18642365:76874-75840;EPI_ISL_18659846:76844-75810;EPI_ISL_18744049:76876-75842;EPI_ISL_19022858:76871-75837;EPI_ISL_19028778:76788-75754;EPI_ISL_19151800:76872-75838;EPI_ISL_19159116:76874-75840;EPI_ISL_19350788:78007-76973;EPI_ISL_19359677:76871-75837;EPI_ISL_19446306:76866-75832;EPI_ISL_19467692:79418-78384;JX878411.1:80126-79092;JX878425.1:79643-78609;JX878426.1:79092-78058;KC257460.1:79060-78026;KJ642612.1:77378-76344;KJ642613.1:78937-77903;KJ642616.1:78514-77480;KJ642618.1:76608-75574;KP849469.1:79451-78417;KP849471.1:79377-78343;LC831698.1:76872-75838;MN648051.1:77094-76060;MN702445.1:72794-71760;MN702446.1:78939-77905;MN702447.1:72307-71273;MN702449.1:72805-71771;MN702452.1:78980-77946;MT903338.1:77037-76003;MT903340.1:76874-75840;MT903341.1:68407-67373;MT903342.1:76889-75855;MT903347.1:78831-77797;ON568298.1:77020-75986;ON622713.1:77106-76072;ON622722.2:76877-75843;ON631963.1:76897-75863;ON649879.1:76707-75673;ON676707.1:76927-75893;ON755039.1:76537-75503;ON808413.1:76877-75843;ON880420.1:77104-76070;ON911481.2:76894-75860;ON918656.1:76909-75875;ON959133.1:76998-75964;ON959136.1:76999-75965;ON983168.1:75573-74539;OP019276.1:76871-75837;OP150924.1:77012-75978;OP160532.1:76837-75803;OP235282.1:76871-75837;OP289782.1:76874-75840;OP331335.1:76891-75857;OP382479.1:76878-75844;OP422341.1:76891-75857;OP434519.1:76870-75836;OP535324.1:76872-75838;OP535333.1:76981-75947;OP536770.1:76873-75839;OP597769.1:76895-75861;OP604566.1:76874-75840;OP612682.1:76799-75981;OP612684.1:76863-76045;OP612686.1:76863-76045;OP696841.1:74148-73114;OP879722.1:76097-75063;OQ419007.1:76107-75073;OQ419012.1:76074-75040;OQ419031.1:76078-75044;OQ419047.1:76080-75046;OQ451898.1:75959-74925;OQ451900.1:76872-75838;OQ581853.1:76898-75864;OQ581855.1:76979-75945;OQ721951.1:76912-75878;OQ721954.1:76869-75835;OR035652.1:76821-75787;OR035664.1:76821-75787;OR035702.1:76821-75787;OR146272.1:76837-75803;OR146330.1:76882-75848;OR264364.1:76892-75858;OR427332.1:76892-75858;OR459777.1:76876-75842;OR459797.1:76891-75857;OR943698.1:79184-78150;PP098589.2:76873-75839;PP496048.1:76877-75843;PP601206.1:79133-78099;PP776553.1:76798-75764;PP852971.1:76871-75837;PP852983.1:76877-75843;PP853000.1:76838-75804;PP853005.1:76846-75812;PP853007.1:76879-75845;PP853013.1:76852-75818;PP853027.1:76883-75849;PP853045.1:76864-75830;PP853048.1:76883-75849;PP860026.1:76881-75847;PP860027.1:76906-75872;PP860028.1:76846-75812;PP860031.1:76900-75866;PP860033.1:76905-75871;PQ153226.1:76871-75837;PQ178862.2:77984-76950;PQ221506.1:72960-71926;PQ221833.1:79126-78092;PQ340477.1:76854-75820;PQ340478.1:76839-75805;PQ340480.1:76918-75884;PQ340483.1:76902-75868;PQ340485.1:76888-75854;PQ340486.1:76903-75869;PQ340487.1:76928-75894;PQ340489.1:76903-75869;PQ340490.1:76854-75820;PQ340491.1:76909-75875;PQ340493.1:76888-75854;PQ340495.1:76918-75884;PQ340496.1:76951-75917;PQ368489.1:76874-75840;PQ451835.1:76959-75925;PQ451836.1:76891-75857;PQ451838.1:76942-75908;PQ451841.1:76902-75868;PQ451842.1:76854-75820 |
| OG0000178 | AY603973.1:66661-65390;AY741551.1:66665-65394;AY753185.1:66904-65633;DQ011154.1:67122-65851;DQ011155.1:67100-65829;EPI_ISL_13331598:64731-63460;EPI_ISL_13363142:64724-63453;EPI_ISL_13374487:64739-63468;EPI_ISL_13502582:64724-63453;EPI_ISL_13508471:64816-63545;EPI_ISL_13607904:64715-63444;EPI_ISL_13624509:64659-63388;EPI_ISL_13889442:64834-63563;EPI_ISL_13953610:64651-63380;EPI_ISL_14181948:64860-63589;EPI_ISL_14838587:66976-65705;EPI_ISL_15263355:64715-63444;EPI_ISL_15269699:64684-63413;EPI_ISL_15293002:64957-63686;EPI_ISL_15641561:64673-63402;EPI_ISL_15831211:64722-63451;EPI_ISL_15992098:64737-63466;EPI_ISL_16233784:64733-63462;EPI_ISL_16946400:64722-63451;EPI_ISL_17048206:64372-63101;EPI_ISL_17703567:64737-63466;EPI_ISL_17718458:64715-63444;EPI_ISL_18213374:64712-63441;EPI_ISL_18360394:64373-63102;EPI_ISL_18557816:64713-63442;EPI_ISL_18642365:64715-63444;EPI_ISL_18659846:64685-63414;EPI_ISL_18744049:64717-63446;EPI_ISL_19022858:64712-63441;EPI_ISL_19028778:64629-63358;EPI_ISL_19151800:64713-63442;EPI_ISL_19159116:64715-63444;EPI_ISL_19350788:65848-64577;EPI_ISL_19359677:64712-63441;EPI_ISL_19446306:64707-63436;EPI_ISL_19467692:67259-65988;JX878411.1:67967-66696;JX878425.1:67484-66213;JX878426.1:66933-65662;KC257460.1:66898-65627;KJ642612.1:65219-63948;KJ642613.1:66778-65507;KJ642616.1:66355-65084;KJ642618.1:64386-63115;KP849469.1:67292-66021;KP849471.1:67217-65946;LC831698.1:64713-63442;MN648051.1:64935-63664;MN702445.1:60635-59364;MN702446.1:66780-65509;MN702447.1:60148-58877;MN702449.1:60646-59375;MN702452.1:66821-65550;MT903338.1:64878-63607;MT903340.1:64715-63444;MT903341.1:56248-54977;MT903342.1:64730-63459;MT903347.1:66672-65401;ON568298.1:64861-63590;ON622713.1:64947-63676;ON622722.2:64718-63447;ON631963.1:64738-63467;ON649879.1:64548-63277;ON676707.1:64768-63497;ON755039.1:64378-63107;ON808413.1:64718-63447;ON880420.1:64945-63674;ON911481.2:64735-63464;ON918656.1:64750-63479;ON959133.1:64839-63568;ON959136.1:64840-63569;ON983168.1:63414-62143;OP019276.1:64712-63441;OP150924.1:64853-63582;OP160532.1:64678-63407;OP235282.1:64712-63441;OP289782.1:64715-63444;OP331335.1:64732-63461;OP382479.1:64719-63448;OP422341.1:64732-63461;OP434519.1:64711-63440;OP535324.1:64713-63442;OP535333.1:64822-63551;OP536770.1:64714-63443;OP597769.1:64736-63465;OP604566.1:64715-63444;OP612682.1:64641-63370;OP612684.1:64705-63434;OP612686.1:64705-63434;OP696841.1:61989-60718;OP879722.1:63938-62667;OQ419007.1:63948-62677;OQ419012.1:63915-62644;OQ419031.1:63919-62648;OQ419047.1:63921-62650;OQ451898.1:63800-62529;OQ451900.1:64713-63442;OQ581853.1:64739-63468;OQ581855.1:64820-63549;OQ721951.1:64753-63482;OQ721954.1:64710-63439;OR035652.1:64662-63391;OR035664.1:64662-63391;OR035702.1:64662-63391;OR146272.1:64678-63407;OR146330.1:64723-63452;OR264364.1:64733-63462;OR427332.1:64733-63462;OR459777.1:64717-63446;OR459797.1:64732-63461;OR943698.1:66962-65691;PP098589.2:64714-63443;PP496048.1:64718-63447;PP601206.1:66974-65703;PP776553.1:64639-63368;PP852971.1:64712-63441;PP852983.1:64718-63447;PP853000.1:64679-63408;PP853005.1:64687-63416;PP853007.1:64720-63449;PP853013.1:64693-63422;PP853027.1:64724-63453;PP853045.1:64705-63434;PP853048.1:64724-63453;PP860026.1:64722-63451;PP860027.1:64747-63476;PP860028.1:64687-63416;PP860031.1:64741-63470;PP860033.1:64746-63475;PQ153226.1:64712-63441;PQ178862.2:65825-64554;PQ221506.1:60801-59530;PQ221833.1:66967-65696;PQ340477.1:64695-63424;PQ340478.1:64680-63409;PQ340480.1:64759-63488;PQ340483.1:64743-63472;PQ340485.1:64729-63458;PQ340486.1:64744-63473;PQ340487.1:64769-63498;PQ340489.1:64744-63473;PQ340490.1:64695-63424;PQ340491.1:64750-63479;PQ340493.1:64729-63458;PQ340495.1:64759-63488;PQ340496.1:64792-63521;PQ368489.1:64715-63444;PQ451835.1:64800-63529;PQ451836.1:64732-63461;PQ451838.1:64783-63512;PQ451841.1:64743-63472;PQ451842.1:64695-63424 |
| OG0000179 | AY603973.1:57085-56696;AY741551.1:57089-56700;AY753185.1:57328-56939;DQ011154.1:57546-57157;DQ011155.1:57524-57135;EPI_ISL_13331598:55156-54767;EPI_ISL_13363142:55149-54760;EPI_ISL_13374487:55164-54775;EPI_ISL_13502582:55149-54760;EPI_ISL_13508471:55241-54852;EPI_ISL_13607904:55140-54751;EPI_ISL_13624509:55084-54695;EPI_ISL_13889442:55259-54870;EPI_ISL_13953610:55076-54687;EPI_ISL_14181948:55285-54896;EPI_ISL_14838587:57400-57011;EPI_ISL_15263355:55140-54751;EPI_ISL_15269699:55109-54720;EPI_ISL_15293002:55382-54993;EPI_ISL_15641561:55098-54709;EPI_ISL_15831211:55147-54758;EPI_ISL_15992098:55162-54773;EPI_ISL_16233784:55158-54769;EPI_ISL_16946400:55147-54758;EPI_ISL_17048206:54797-54408;EPI_ISL_17703567:55162-54773;EPI_ISL_17718458:55140-54751;EPI_ISL_18213374:55137-54748;EPI_ISL_18360394:54798-54409;EPI_ISL_18557816:55138-54749;EPI_ISL_18642365:55140-54751;EPI_ISL_18659846:55110-54721;EPI_ISL_18744049:55142-54753;EPI_ISL_19022858:55137-54748;EPI_ISL_19028778:55054-54665;EPI_ISL_19151800:55138-54749;EPI_ISL_19159116:55140-54751;EPI_ISL_19350788:56272-55883;EPI_ISL_19359677:55137-54748;EPI_ISL_19446306:55132-54743;EPI_ISL_19467692:57683-57294;JX878411.1:58391-58002;JX878425.1:57908-57519;JX878426.1:57357-56968;KC257460.1:57322-56933;KJ642612.1:55643-55254;KJ642613.1:57202-56813;KJ642616.1:56779-56390;KJ642618.1:54810-54421;KP849469.1:57716-57327;KP849471.1:57641-57252;LC831698.1:55138-54749;MN648051.1:55360-54971;MN702445.1:51059-50670;MN702446.1:57204-56815;MN702447.1:50572-50183;MN702449.1:51070-50681;MN702452.1:57245-56856;MT903338.1:55303-54914;MT903340.1:55140-54751;MT903341.1:46673-46284;MT903342.1:55155-54766;MT903347.1:57096-56707;ON568298.1:55286-54897;ON622713.1:55372-54983;ON622722.2:55143-54754;ON631963.1:55163-54774;ON649879.1:54973-54584;ON676707.1:55193-54804;ON755039.1:54803-54414;ON808413.1:55143-54754;ON880420.1:55370-54981;ON911481.2:55160-54771;ON918656.1:55175-54786;ON959133.1:55264-54875;ON959136.1:55265-54876;ON983168.1:53839-53450;OP019276.1:55137-54748;OP150924.1:55278-54889;OP160532.1:55103-54714;OP235282.1:55137-54748;OP289782.1:55140-54751;OP331335.1:55157-54768;OP382479.1:55144-54755;OP422341.1:55157-54768;OP434519.1:55136-54747;OP535324.1:55138-54749;OP535333.1:55247-54858;OP536770.1:55139-54750;OP597769.1:55161-54772;OP604566.1:55140-54751;OP612682.1:55066-54677;OP612684.1:55130-54741;OP612686.1:55130-54741;OP696841.1:52414-52025;OP879722.1:54363-53974;OQ419007.1:54373-53984;OQ419012.1:54340-53951;OQ419031.1:54344-53955;OQ419047.1:54373-53984;OQ451898.1:54225-53836;OQ451900.1:55138-54749;OQ581853.1:55164-54775;OQ581855.1:55245-54856;OQ721951.1:55178-54789;OQ721954.1:55135-54746;OR035652.1:55087-54698;OR035664.1:55087-54698;OR035702.1:55087-54698;OR146272.1:55103-54714;OR146330.1:55148-54759;OR264364.1:55158-54769;OR427332.1:55158-54769;OR459777.1:55142-54753;OR459797.1:55157-54768;OR943698.1:57386-56997;PP098589.2:55139-54750;PP496048.1:55143-54754;PP601206.1:57398-57009;PP776553.1:55064-54675;PP852971.1:55137-54748;PP852983.1:55143-54754;PP853000.1:55104-54715;PP853005.1:55112-54723;PP853007.1:55145-54756;PP853013.1:55118-54729;PP853027.1:55149-54760;PP853045.1:55130-54741;PP853048.1:55149-54760;PP860026.1:55147-54758;PP860027.1:55172-54783;PP860028.1:55113-54724;PP860031.1:55166-54777;PP860033.1:55171-54782;PQ153226.1:55137-54748;PQ178862.2:56249-55860;PQ221506.1:51225-50836;PQ221833.1:57391-57002;PQ340477.1:55120-54731;PQ340478.1:55105-54716;PQ340480.1:55184-54795;PQ340483.1:55168-54779;PQ340485.1:55154-54765;PQ340486.1:55169-54780;PQ340487.1:55194-54805;PQ340489.1:55169-54780;PQ340490.1:55120-54731;PQ340491.1:55175-54786;PQ340493.1:55154-54765;PQ340495.1:55184-54795;PQ340496.1:55217-54828;PQ368489.1:55140-54751;PQ451835.1:55225-54836;PQ451836.1:55157-54768;PQ451838.1:55208-54819;PQ451841.1:55168-54779;PQ451842.1:55120-54731 |
| OG0000180 | AY603973.1:49372-48593;AY741551.1:49376-48597;AY753185.1:49615-48836;DQ011154.1:49586-48807;DQ011155.1:49566-48787;EPI_ISL_13331598:47443-46664;EPI_ISL_13363142:47436-46657;EPI_ISL_13374487:47451-46672;EPI_ISL_13502582:47436-46657;EPI_ISL_13508471:47528-46749;EPI_ISL_13607904:47427-46648;EPI_ISL_13624509:47371-46592;EPI_ISL_13889442:47546-46767;EPI_ISL_13953610:47387-46608;EPI_ISL_14181948:47572-46793;EPI_ISL_14838587:49440-48661;EPI_ISL_15263355:47427-46648;EPI_ISL_15269699:47396-46617;EPI_ISL_15293002:47669-46890;EPI_ISL_15641561:47385-46606;EPI_ISL_15831211:47434-46655;EPI_ISL_15992098:47449-46670;EPI_ISL_16233784:47445-46666;EPI_ISL_16946400:47434-46655;EPI_ISL_17048206:47084-46305;EPI_ISL_17703567:47449-46670;EPI_ISL_17718458:47427-46648;EPI_ISL_18213374:47424-46645;EPI_ISL_18360394:47085-46306;EPI_ISL_18557816:47425-46646;EPI_ISL_18642365:47427-46648;EPI_ISL_18659846:47397-46618;EPI_ISL_18744049:47429-46650;EPI_ISL_19022858:47424-46645;EPI_ISL_19028778:47341-46562;EPI_ISL_19151800:47425-46646;EPI_ISL_19159116:47427-46648;EPI_ISL_19350788:48314-47535;EPI_ISL_19359677:47424-46645;EPI_ISL_19446306:47419-46640;EPI_ISL_19467692:49719-48940;JX878411.1:50433-49654;JX878425.1:49948-49169;JX878426.1:49392-48613;KC257460.1:49364-48585;KJ642612.1:47678-46899;KJ642613.1:49256-48477;KJ642616.1:49066-48287;KJ642618.1:46846-46067;KP849469.1:49756-48977;KP849471.1:49681-48902;LC831698.1:47425-46646;MN648051.1:47647-46868;MN702445.1:43101-42322;MN702446.1:49246-48467;MN702447.1:42612-41833;MN702449.1:43112-42333;MN702452.1:49285-48506;MT903338.1:47590-46811;MT903340.1:47427-46648;MT903341.1:38960-38181;MT903342.1:47442-46663;MT903347.1:49382-48603;ON568298.1:47573-46794;ON622713.1:47659-46880;ON622722.2:47430-46651;ON631963.1:47450-46671;ON649879.1:47260-46481;ON676707.1:47480-46701;ON755039.1:47090-46311;ON808413.1:47430-46651;ON880420.1:47657-46878;ON911481.2:47447-46668;ON918656.1:47462-46683;ON959133.1:47551-46772;ON959136.1:47552-46773;ON983168.1:46126-45347;OP019276.1:47424-46645;OP150924.1:47565-46786;OP160532.1:47390-46611;OP235282.1:47424-46645;OP289782.1:47427-46648;OP331335.1:47444-46665;OP382479.1:47431-46652;OP422341.1:47444-46665;OP434519.1:47423-46644;OP535324.1:47425-46646;OP535333.1:47534-46755;OP536770.1:47426-46647;OP597769.1:47448-46669;OP604566.1:47427-46648;OP612682.1:47353-46574;OP612684.1:47417-46638;OP612686.1:47417-46638;OP696841.1:44701-43922;OP879722.1:46650-45871;OQ419007.1:46660-45881;OQ419012.1:46649-45870;OQ419031.1:46631-45852;OQ419047.1:46660-45881;OQ451898.1:46512-45733;OQ451900.1:47425-46646;OQ581853.1:47451-46672;OQ581855.1:47532-46753;OQ721951.1:47465-46686;OQ721954.1:47422-46643;OR035652.1:47374-46595;OR035664.1:47374-46595;OR035702.1:47374-46595;OR146272.1:47390-46611;OR146330.1:47435-46656;OR264364.1:47445-46666;OR427332.1:47445-46666;OR459777.1:47429-46650;OR459797.1:47444-46665;OR943698.1:49420-48641;PP098589.2:47426-46647;PP496048.1:47430-46651;PP601206.1:49438-48659;PP776553.1:47351-46572;PP852971.1:47424-46645;PP852983.1:47430-46651;PP853000.1:47391-46612;PP853005.1:47399-46620;PP853007.1:47432-46653;PP853013.1:47405-46626;PP853027.1:47436-46657;PP853045.1:47417-46638;PP853048.1:47436-46657;PP860026.1:47434-46655;PP860027.1:47459-46680;PP860028.1:47400-46621;PP860031.1:47453-46674;PP860033.1:47458-46679;PQ153226.1:47424-46645;PQ178862.2:48291-47512;PQ221506.1:43260-42481;PQ221833.1:49431-48652;PQ340477.1:47407-46628;PQ340478.1:47392-46613;PQ340480.1:47471-46692;PQ340483.1:47455-46676;PQ340485.1:47441-46662;PQ340486.1:47456-46677;PQ340487.1:47481-46702;PQ340489.1:47456-46677;PQ340490.1:47407-46628;PQ340491.1:47462-46683;PQ340493.1:47441-46662;PQ340495.1:47471-46692;PQ340496.1:47504-46725;PQ368489.1:47427-46648;PQ451835.1:47512-46733;PQ451836.1:47444-46665;PQ451838.1:47495-46716;PQ451841.1:47455-46676;PQ451842.1:47407-46628 |
| OG0000181 | AY603973.1:48427-47966;AY741551.1:48431-47970;AY753185.1:48670-48209;DQ011154.1:48645-48184;DQ011155.1:48626-48165;EPI_ISL_13331598:46498-46037;EPI_ISL_13363142:46491-46030;EPI_ISL_13374487:46506-46045;EPI_ISL_13502582:46491-46030;EPI_ISL_13508471:46583-46122;EPI_ISL_13607904:46482-46021;EPI_ISL_13624509:46426-45965;EPI_ISL_13889442:46601-46140;EPI_ISL_13953610:46442-45981;EPI_ISL_14181948:46627-46166;EPI_ISL_14838587:48499-48038;EPI_ISL_15263355:46482-46021;EPI_ISL_15269699:46451-45990;EPI_ISL_15293002:46724-46263;EPI_ISL_15641561:46440-45979;EPI_ISL_15831211:46489-46028;EPI_ISL_15992098:46504-46043;EPI_ISL_16233784:46500-46039;EPI_ISL_16946400:46489-46028;EPI_ISL_17048206:46139-45678;EPI_ISL_17703567:46504-46043;EPI_ISL_17718458:46482-46021;EPI_ISL_18213374:46479-46018;EPI_ISL_18360394:46140-45679;EPI_ISL_18557816:46480-46019;EPI_ISL_18642365:46482-46021;EPI_ISL_18659846:46452-45991;EPI_ISL_18744049:46484-46023;EPI_ISL_19022858:46479-46018;EPI_ISL_19028778:46396-45935;EPI_ISL_19151800:46480-46019;EPI_ISL_19159116:46482-46021;EPI_ISL_19350788:47374-46913;EPI_ISL_19359677:46479-46018;EPI_ISL_19446306:46474-46013;EPI_ISL_19467692:48778-48317;JX878411.1:49493-49032;JX878425.1:49007-48546;JX878426.1:48452-47991;KC257460.1:48423-47962;KJ642612.1:46738-46277;KJ642613.1:48315-47854;KJ642616.1:48121-47660;KJ642618.1:45906-45445;KP849469.1:48815-48354;KP849471.1:48741-48280;LC831698.1:46480-46019;MN648051.1:46702-46241;MN702445.1:42160-41699;MN702446.1:48305-47844;MN702447.1:41671-41210;MN702449.1:42171-41710;MN702452.1:48344-47883;MT903338.1:46645-46184;MT903340.1:46482-46021;MT903341.1:38015-37554;MT903342.1:46497-46036;MT903347.1:48437-47976;ON568298.1:46628-46167;ON622713.1:46714-46253;ON622722.2:46485-46024;ON631963.1:46505-46044;ON649879.1:46315-45854;ON676707.1:46535-46074;ON755039.1:46145-45684;ON808413.1:46485-46024;ON880420.1:46712-46251;ON911481.2:46502-46041;ON918656.1:46517-46056;ON959133.1:46606-46145;ON959136.1:46607-46146;ON983168.1:45181-44720;OP019276.1:46479-46018;OP150924.1:46620-46159;OP160532.1:46445-45984;OP235282.1:46479-46018;OP289782.1:46482-46021;OP331335.1:46499-46038;OP382479.1:46486-46025;OP422341.1:46499-46038;OP434519.1:46478-46017;OP535324.1:46480-46019;OP535333.1:46589-46128;OP536770.1:46481-46020;OP597769.1:46503-46042;OP604566.1:46482-46021;OP612682.1:46408-45947;OP612684.1:46472-46011;OP612686.1:46472-46011;OP696841.1:43756-43295;OP879722.1:45705-45244;OQ419007.1:45715-45254;OQ419012.1:45704-45243;OQ419031.1:45686-45225;OQ419047.1:45715-45254;OQ451898.1:45567-45106;OQ451900.1:46480-46019;OQ581853.1:46506-46045;OQ581855.1:46587-46126;OQ721951.1:46520-46059;OQ721954.1:46477-46016;OR035652.1:46429-45968;OR035664.1:46429-45968;OR035702.1:46429-45968;OR146272.1:46445-45984;OR146330.1:46490-46029;OR264364.1:46500-46039;OR427332.1:46500-46039;OR459777.1:46484-46023;OR459797.1:46499-46038;OR943698.1:48480-48019;PP098589.2:46481-46020;PP496048.1:46485-46024;PP601206.1:48497-48036;PP776553.1:46406-45945;PP852971.1:46479-46018;PP852983.1:46485-46024;PP853000.1:46446-45985;PP853005.1:46454-45993;PP853007.1:46487-46026;PP853013.1:46460-45999;PP853027.1:46491-46030;PP853045.1:46472-46011;PP853048.1:46491-46030;PP860026.1:46489-46028;PP860027.1:46514-46053;PP860028.1:46455-45994;PP860031.1:46508-46047;PP860033.1:46513-46052;PQ153226.1:46479-46018;PQ178862.2:47351-46890;PQ221506.1:42320-41859;PQ221833.1:48490-48029;PQ340477.1:46462-46001;PQ340478.1:46447-45986;PQ340480.1:46526-46065;PQ340483.1:46510-46049;PQ340485.1:46496-46035;PQ340486.1:46511-46050;PQ340487.1:46536-46075;PQ340489.1:46511-46050;PQ340490.1:46462-46001;PQ340491.1:46517-46056;PQ340493.1:46496-46035;PQ340495.1:46526-46065;PQ340496.1:46559-46098;PQ368489.1:46482-46021;PQ451835.1:46567-46106;PQ451836.1:46499-46038;PQ451838.1:46550-46089;PQ451841.1:46510-46049;PQ451842.1:46462-46001 |
| OG0000182 | AY603973.1:47842-45629;AY741551.1:47846-45633;AY753185.1:48085-45872;DQ011154.1:48060-45847;DQ011155.1:48041-45828;EPI_ISL_13331598:45913-43700;EPI_ISL_13363142:45906-43693;EPI_ISL_13374487:45921-43708;EPI_ISL_13502582:45906-43693;EPI_ISL_13508471:45998-43785;EPI_ISL_13607904:45897-43684;EPI_ISL_13624509:45841-43628;EPI_ISL_13889442:46016-43803;EPI_ISL_13953610:45857-43644;EPI_ISL_14181948:46042-43829;EPI_ISL_14838587:47914-45701;EPI_ISL_15263355:45897-43684;EPI_ISL_15269699:45866-43653;EPI_ISL_15293002:46139-43926;EPI_ISL_15641561:45855-43642;EPI_ISL_15831211:45904-43691;EPI_ISL_15992098:45919-43706;EPI_ISL_16233784:45915-43702;EPI_ISL_16946400:45904-43691;EPI_ISL_17048206:45554-43341;EPI_ISL_17703567:45919-43706;EPI_ISL_17718458:45897-43684;EPI_ISL_18213374:45894-43681;EPI_ISL_18360394:45555-43342;EPI_ISL_18557816:45895-43682;EPI_ISL_18642365:45897-43684;EPI_ISL_18659846:45867-43654;EPI_ISL_18744049:45899-43686;EPI_ISL_19022858:45894-43681;EPI_ISL_19028778:45811-43598;EPI_ISL_19151800:45895-43682;EPI_ISL_19159116:45897-43684;EPI_ISL_19350788:46788-44575;EPI_ISL_19359677:45894-43681;EPI_ISL_19446306:45889-43676;EPI_ISL_19467692:48193-45980;JX878411.1:48908-46695;JX878425.1:48422-46209;JX878426.1:47867-45654;KC257460.1:47838-45625;KJ642612.1:46153-43940;KJ642613.1:47730-45517;KJ642616.1:47536-45323;KJ642618.1:45321-43108;KP849469.1:48230-46017;KP849471.1:48156-45943;LC831698.1:45895-43682;MN648051.1:46117-43904;MN702445.1:41575-39362;MN702446.1:47720-45507;MN702447.1:41086-38873;MN702449.1:41586-39373;MN702452.1:47759-45546;MT903338.1:46060-43847;MT903340.1:45897-43684;MT903341.1:37430-35217;MT903342.1:45912-43699;MT903347.1:47855-45642;ON568298.1:46043-43830;ON622713.1:46129-43916;ON622722.2:45900-43687;ON631963.1:45920-43707;ON649879.1:45730-43517;ON676707.1:45950-43737;ON755039.1:45560-43347;ON808413.1:45900-43687;ON880420.1:46127-43914;ON911481.2:45917-43704;ON918656.1:45932-43719;ON959133.1:46021-43808;ON959136.1:46022-43809;ON983168.1:44596-42383;OP019276.1:45894-43681;OP150924.1:46035-43822;OP160532.1:45860-43647;OP235282.1:45894-43681;OP289782.1:45897-43684;OP331335.1:45914-43701;OP382479.1:45901-43688;OP422341.1:45914-43701;OP434519.1:45893-43680;OP535324.1:45895-43682;OP535333.1:46004-43791;OP536770.1:45896-43683;OP597769.1:45918-43705;OP604566.1:45897-43684;OP612682.1:45823-43610;OP612684.1:45887-43674;OP612686.1:45887-43674;OP696841.1:43171-40958;OP879722.1:45120-42907;OQ419007.1:45130-42917;OQ419012.1:45119-42906;OQ419031.1:45101-42888;OQ419047.1:45130-42917;OQ451898.1:44982-42769;OQ451900.1:45895-43682;OQ581853.1:45921-43708;OQ581855.1:46002-43789;OQ721951.1:45935-43722;OQ721954.1:45892-43679;OR035652.1:45844-43631;OR035664.1:45844-43631;OR035702.1:45844-43631;OR146272.1:45860-43647;OR146330.1:45905-43692;OR264364.1:45915-43702;OR427332.1:45915-43702;OR459777.1:45899-43686;OR459797.1:45914-43701;OR943698.1:47895-45682;PP098589.2:45896-43683;PP496048.1:45900-43687;PP601206.1:47912-45699;PP776553.1:45821-43608;PP852971.1:45894-43681;PP852983.1:45900-43687;PP853000.1:45861-43648;PP853005.1:45869-43656;PP853007.1:45902-43689;PP853013.1:45875-43662;PP853027.1:45906-43693;PP853045.1:45887-43674;PP853048.1:45906-43693;PP860026.1:45904-43691;PP860027.1:45929-43716;PP860028.1:45870-43657;PP860031.1:45923-43710;PP860033.1:45928-43715;PQ153226.1:45894-43681;PQ178862.2:46765-44552;PQ221506.1:41735-39522;PQ221833.1:47904-45691;PQ340477.1:45877-43664;PQ340478.1:45862-43649;PQ340480.1:45941-43728;PQ340483.1:45925-43712;PQ340485.1:45911-43698;PQ340486.1:45926-43713;PQ340487.1:45951-43738;PQ340489.1:45926-43713;PQ340490.1:45877-43664;PQ340491.1:45932-43719;PQ340493.1:45911-43698;PQ340495.1:45941-43728;PQ340496.1:45974-43761;PQ368489.1:45897-43684;PQ451835.1:45982-43769;PQ451836.1:45914-43701;PQ451838.1:45965-43752;PQ451841.1:45925-43712;PQ451842.1:45877-43664 |
| OG0000183 | AY603973.1:43828-43133;AY741551.1:43832-43137;AY753185.1:44071-43376;DQ011154.1:44047-43352;DQ011155.1:44028-43333;EPI_ISL_13331598:41899-41204;EPI_ISL_13363142:41892-41197;EPI_ISL_13374487:41907-41212;EPI_ISL_13502582:41892-41197;EPI_ISL_13508471:41984-41289;EPI_ISL_13607904:41883-41188;EPI_ISL_13624509:41827-41132;EPI_ISL_13889442:42002-41307;EPI_ISL_13953610:41850-41155;EPI_ISL_14181948:42028-41333;EPI_ISL_14838587:43901-43206;EPI_ISL_15263355:41883-41188;EPI_ISL_15269699:41852-41157;EPI_ISL_15293002:42125-41430;EPI_ISL_15641561:41841-41146;EPI_ISL_15831211:41890-41195;EPI_ISL_15992098:41905-41210;EPI_ISL_16233784:41901-41206;EPI_ISL_16946400:41890-41195;EPI_ISL_17048206:41540-40845;EPI_ISL_17703567:41905-41210;EPI_ISL_17718458:41883-41188;EPI_ISL_18213374:41880-41185;EPI_ISL_18360394:41541-40846;EPI_ISL_18557816:41881-41186;EPI_ISL_18642365:41883-41188;EPI_ISL_18659846:41853-41158;EPI_ISL_18744049:41885-41190;EPI_ISL_19022858:41880-41185;EPI_ISL_19028778:41797-41102;EPI_ISL_19151800:41881-41186;EPI_ISL_19159116:41883-41188;EPI_ISL_19350788:42775-42080;EPI_ISL_19359677:41880-41185;EPI_ISL_19446306:41875-41180;EPI_ISL_19467692:44180-43485;JX878411.1:44895-44200;JX878425.1:44409-43714;JX878426.1:43854-43159;KC257460.1:43825-43130;KJ642612.1:42140-41445;KJ642613.1:43716-43021;KJ642616.1:43522-42827;KJ642618.1:41308-40613;KP849469.1:44217-43522;KP849471.1:44143-43448;LC831698.1:41881-41186;MN648051.1:42103-41408;MN702445.1:37562-36867;MN702446.1:43707-43012;MN702447.1:37073-36378;MN702449.1:37573-36878;MN702452.1:43746-43051;MT903338.1:42046-41351;MT903340.1:41883-41188;MT903341.1:33416-32721;MT903342.1:41898-41203;MT903347.1:43841-43146;ON568298.1:42029-41334;ON622713.1:42115-41420;ON622722.2:41886-41191;ON631963.1:41906-41211;ON649879.1:41716-41021;ON676707.1:41936-41241;ON755039.1:41546-40851;ON808413.1:41886-41191;ON880420.1:42113-41418;ON911481.2:41903-41208;ON918656.1:41918-41223;ON959133.1:42007-41312;ON959136.1:42008-41313;ON983168.1:40582-39887;OP019276.1:41880-41185;OP150924.1:42021-41326;OP160532.1:41846-41151;OP235282.1:41880-41185;OP289782.1:41883-41188;OP331335.1:41900-41205;OP382479.1:41887-41192;OP422341.1:41900-41205;OP434519.1:41879-41184;OP535324.1:41881-41186;OP535333.1:41990-41295;OP536770.1:41882-41187;OP597769.1:41904-41209;OP604566.1:41883-41188;OP612682.1:41809-41114;OP612684.1:41873-41178;OP612686.1:41873-41178;OP696841.1:39157-38462;OP879722.1:41106-40411;OQ419007.1:41116-40421;OQ419012.1:41105-40410;OQ419031.1:41087-40392;OQ419047.1:41116-40421;OQ451898.1:40968-40273;OQ451900.1:41881-41186;OQ581853.1:41907-41212;OQ581855.1:41988-41293;OQ721951.1:41921-41226;OQ721954.1:41878-41183;OR035652.1:41830-41135;OR035664.1:41830-41135;OR035702.1:41830-41135;OR146272.1:41846-41151;OR146330.1:41891-41196;OR264364.1:41901-41206;OR427332.1:41901-41206;OR459777.1:41885-41190;OR459797.1:41900-41205;OR943698.1:43882-43187;PP098589.2:41882-41187;PP496048.1:41886-41191;PP601206.1:43899-43204;PP776553.1:41807-41112;PP852971.1:41880-41185;PP852983.1:41886-41191;PP853000.1:41847-41152;PP853005.1:41855-41160;PP853007.1:41888-41193;PP853013.1:41861-41166;PP853027.1:41892-41197;PP853045.1:41873-41178;PP853048.1:41892-41197;PP860026.1:41890-41195;PP860027.1:41915-41220;PP860028.1:41856-41161;PP860031.1:41909-41214;PP860033.1:41914-41219;PQ153226.1:41880-41185;PQ178862.2:42752-42057;PQ221506.1:37722-37027;PQ221833.1:43891-43196;PQ340477.1:41863-41168;PQ340478.1:41848-41153;PQ340480.1:41927-41232;PQ340483.1:41911-41216;PQ340485.1:41897-41202;PQ340486.1:41912-41217;PQ340487.1:41937-41242;PQ340489.1:41912-41217;PQ340490.1:41863-41168;PQ340491.1:41918-41223;PQ340493.1:41897-41202;PQ340495.1:41927-41232;PQ340496.1:41960-41265;PQ368489.1:41883-41188;PQ451835.1:41968-41273;PQ451836.1:41900-41205;PQ451838.1:41951-41256;PQ451841.1:41911-41216;PQ451842.1:41863-41168 |
| OG0000184 | AY603973.1:43126-42650;AY741551.1:43130-42654;AY753185.1:43369-42893;DQ011154.1:43345-42869;DQ011155.1:43326-42850;EPI_ISL_13331598:41197-40721;EPI_ISL_13363142:41190-40714;EPI_ISL_13374487:41205-40729;EPI_ISL_13502582:41190-40714;EPI_ISL_13508471:41282-40806;EPI_ISL_13607904:41181-40705;EPI_ISL_13624509:41125-40649;EPI_ISL_13889442:41300-40824;EPI_ISL_13953610:41148-40672;EPI_ISL_14181948:41326-40850;EPI_ISL_14838587:43199-42723;EPI_ISL_15263355:41181-40705;EPI_ISL_15269699:41150-40674;EPI_ISL_15293002:41423-40947;EPI_ISL_15641561:41139-40663;EPI_ISL_15831211:41188-40712;EPI_ISL_15992098:41203-40727;EPI_ISL_16233784:41199-40723;EPI_ISL_16946400:41188-40712;EPI_ISL_17048206:40838-40362;EPI_ISL_17703567:41203-40727;EPI_ISL_17718458:41181-40705;EPI_ISL_18213374:41178-40702;EPI_ISL_18360394:40839-40363;EPI_ISL_18557816:41179-40703;EPI_ISL_18642365:41181-40705;EPI_ISL_18659846:41151-40675;EPI_ISL_18744049:41183-40707;EPI_ISL_19022858:41178-40702;EPI_ISL_19028778:41095-40619;EPI_ISL_19151800:41179-40703;EPI_ISL_19159116:41181-40705;EPI_ISL_19350788:42073-41597;EPI_ISL_19359677:41178-40702;EPI_ISL_19446306:41173-40697;EPI_ISL_19467692:43478-43002;JX878411.1:44193-43717;JX878425.1:43707-43231;JX878426.1:43152-42676;KC257460.1:43123-42647;KJ642612.1:41438-40962;KJ642613.1:43014-42538;KJ642616.1:42820-42344;KJ642618.1:40606-40130;KP849469.1:43515-43039;KP849471.1:43441-42965;LC831698.1:41179-40703;MN648051.1:41401-40925;MN702445.1:36860-36384;MN702446.1:43005-42529;MN702447.1:36371-35895;MN702449.1:36871-36395;MN702452.1:43044-42568;MT903338.1:41344-40868;MT903340.1:41181-40705;MT903341.1:32714-32238;MT903342.1:41196-40720;MT903347.1:43139-42663;ON568298.1:41327-40851;ON622713.1:41413-40937;ON622722.2:41184-40708;ON631963.1:41204-40728;ON649879.1:41014-40538;ON676707.1:41234-40758;ON755039.1:40844-40368;ON808413.1:41184-40708;ON880420.1:41411-40935;ON911481.2:41201-40725;ON918656.1:41216-40740;ON959133.1:41305-40829;ON959136.1:41306-40830;ON983168.1:39880-39404;OP019276.1:41178-40702;OP150924.1:41319-40843;OP160532.1:41144-40668;OP235282.1:41178-40702;OP289782.1:41181-40705;OP331335.1:41198-40722;OP382479.1:41185-40709;OP422341.1:41198-40722;OP434519.1:41177-40701;OP535324.1:41179-40703;OP535333.1:41288-40812;OP536770.1:41180-40704;OP597769.1:41202-40726;OP604566.1:41181-40705;OP612682.1:41107-40631;OP612684.1:41171-40695;OP612686.1:41171-40695;OP696841.1:38455-37979;OP879722.1:40404-39928;OQ419007.1:40414-39938;OQ419012.1:40403-39927;OQ419031.1:40385-39909;OQ419047.1:40414-39938;OQ451898.1:40266-39790;OQ451900.1:41179-40703;OQ581853.1:41205-40729;OQ581855.1:41286-40810;OQ721951.1:41219-40743;OQ721954.1:41176-40700;OR035652.1:41128-40652;OR035664.1:41128-40652;OR035702.1:41128-40652;OR146272.1:41144-40668;OR146330.1:41189-40713;OR264364.1:41199-40723;OR427332.1:41199-40723;OR459777.1:41183-40707;OR459797.1:41198-40722;OR943698.1:43180-42704;PP098589.2:41180-40704;PP496048.1:41184-40708;PP601206.1:43197-42721;PP776553.1:41105-40629;PP852971.1:41178-40702;PP852983.1:41184-40708;PP853000.1:41145-40669;PP853005.1:41153-40677;PP853007.1:41186-40710;PP853013.1:41159-40683;PP853027.1:41190-40714;PP853045.1:41171-40695;PP853048.1:41190-40714;PP860026.1:41188-40712;PP860027.1:41213-40737;PP860028.1:41154-40678;PP860031.1:41207-40731;PP860033.1:41212-40736;PQ153226.1:41178-40702;PQ178862.2:42050-41574;PQ221506.1:37020-36544;PQ221833.1:43189-42713;PQ340477.1:41161-40685;PQ340478.1:41146-40670;PQ340480.1:41225-40749;PQ340483.1:41209-40733;PQ340485.1:41195-40719;PQ340486.1:41210-40734;PQ340487.1:41235-40759;PQ340489.1:41210-40734;PQ340490.1:41161-40685;PQ340491.1:41216-40740;PQ340493.1:41195-40719;PQ340495.1:41225-40749;PQ340496.1:41258-40782;PQ368489.1:41181-40705;PQ451835.1:41266-40790;PQ451836.1:41198-40722;PQ451838.1:41249-40773;PQ451841.1:41209-40733;PQ451842.1:41161-40685 |
| OG0000185 | AY603973.1:42139-41021;AY741551.1:42143-41025;AY753185.1:42382-41264;DQ011154.1:42358-41240;DQ011155.1:42339-41221;EPI_ISL_13331598:40210-39092;EPI_ISL_13363142:40203-39085;EPI_ISL_13374487:40218-39100;EPI_ISL_13502582:40203-39085;EPI_ISL_13508471:40295-39177;EPI_ISL_13607904:40194-39076;EPI_ISL_13624509:40138-39020;EPI_ISL_13889442:40313-39195;EPI_ISL_13953610:40161-39043;EPI_ISL_14181948:40339-39221;EPI_ISL_14838587:42212-41094;EPI_ISL_15263355:40194-39076;EPI_ISL_15269699:40163-39045;EPI_ISL_15293002:40436-39318;EPI_ISL_15641561:40152-39034;EPI_ISL_15831211:40201-39083;EPI_ISL_15992098:40216-39098;EPI_ISL_16233784:40212-39094;EPI_ISL_16946400:40201-39083;EPI_ISL_17048206:39851-38733;EPI_ISL_17703567:40216-39098;EPI_ISL_17718458:40194-39076;EPI_ISL_18213374:40191-39073;EPI_ISL_18360394:39852-38734;EPI_ISL_18557816:40192-39074;EPI_ISL_18642365:40194-39076;EPI_ISL_18659846:40164-39046;EPI_ISL_18744049:40196-39078;EPI_ISL_19022858:40191-39073;EPI_ISL_19028778:40108-38990;EPI_ISL_19151800:40192-39074;EPI_ISL_19159116:40194-39076;EPI_ISL_19350788:41086-39968;EPI_ISL_19359677:40191-39073;EPI_ISL_19446306:40186-39068;EPI_ISL_19467692:42491-41373;JX878411.1:43206-42088;JX878425.1:42720-41602;JX878426.1:42165-41047;KC257460.1:42136-41018;KJ642612.1:40451-39333;KJ642613.1:42027-40909;KJ642616.1:41833-40715;KJ642618.1:39619-38501;KP849469.1:42528-41410;KP849471.1:42454-41336;LC831698.1:40192-39074;MN648051.1:40414-39296;MN702445.1:35873-34755;MN702446.1:42018-40900;MN702447.1:35384-34266;MN702449.1:35884-34766;MN702452.1:42057-40939;MT903338.1:40357-39239;MT903340.1:40194-39076;MT903341.1:31727-30609;MT903342.1:40209-39091;MT903347.1:42152-41034;ON568298.1:40340-39222;ON622713.1:40426-39308;ON622722.2:40197-39079;ON631963.1:40217-39099;ON649879.1:40027-38909;ON676707.1:40247-39129;ON755039.1:39857-38739;ON808413.1:40197-39079;ON880420.1:40424-39306;ON911481.2:40214-39096;ON918656.1:40229-39111;ON959133.1:40318-39200;ON959136.1:40319-39201;ON983168.1:38893-37775;OP019276.1:40191-39073;OP150924.1:40332-39214;OP160532.1:40157-39039;OP235282.1:40191-39073;OP289782.1:40194-39076;OP331335.1:40211-39093;OP382479.1:40198-39080;OP422341.1:40211-39093;OP434519.1:40190-39072;OP535324.1:40192-39074;OP535333.1:40301-39183;OP536770.1:40193-39075;OP597769.1:40215-39097;OP604566.1:40194-39076;OP612682.1:40120-39002;OP612684.1:40184-39066;OP612686.1:40184-39066;OP696841.1:37468-36350;OP879722.1:39417-38299;OQ419007.1:39427-38309;OQ419012.1:39416-38301;OQ419031.1:39398-38280;OQ419047.1:39427-38309;OQ451898.1:39279-38161;OQ451900.1:40192-39074;OQ581853.1:40218-39100;OQ581855.1:40299-39181;OQ721951.1:40232-39114;OQ721954.1:40189-39071;OR035652.1:40141-39023;OR035664.1:40141-39023;OR035702.1:40141-39023;OR146272.1:40157-39039;OR146330.1:40202-39084;OR264364.1:40212-39094;OR427332.1:40212-39094;OR459777.1:40196-39078;OR459797.1:40211-39093;OR943698.1:42193-41075;PP098589.2:40193-39075;PP496048.1:40197-39079;PP601206.1:42210-41092;PP776553.1:40118-39000;PP852971.1:40191-39073;PP852983.1:40197-39079;PP853000.1:40158-39040;PP853005.1:40166-39048;PP853007.1:40199-39081;PP853013.1:40172-39054;PP853027.1:40203-39085;PP853045.1:40184-39066;PP853048.1:40203-39085;PP860026.1:40201-39083;PP860027.1:40226-39108;PP860028.1:40167-39049;PP860031.1:40220-39102;PP860033.1:40225-39107;PQ153226.1:40191-39073;PQ178862.2:41063-39945;PQ221506.1:36033-34915;PQ221833.1:42202-41084;PQ340477.1:40174-39056;PQ340478.1:40159-39041;PQ340480.1:40238-39120;PQ340483.1:40222-39104;PQ340485.1:40208-39090;PQ340486.1:40223-39105;PQ340487.1:40248-39130;PQ340489.1:40223-39105;PQ340490.1:40174-39056;PQ340491.1:40229-39114;PQ340493.1:40208-39090;PQ340495.1:40238-39120;PQ340496.1:40271-39153;PQ368489.1:40194-39076;PQ451835.1:40279-39164;PQ451836.1:40211-39096;PQ451838.1:40262-39147;PQ451841.1:40222-39107;PQ451842.1:40174-39059 |
| OG0000186 | AY603973.1:40978-39071;AY741551.1:40982-39075;AY753185.1:41221-39314;DQ011154.1:41198-39291;DQ011155.1:41179-39272;EPI_ISL_13331598:39049-37142;EPI_ISL_13363142:39042-37135;EPI_ISL_13374487:39057-37150;EPI_ISL_13502582:39042-37135;EPI_ISL_13508471:39134-37227;EPI_ISL_13607904:39033-37126;EPI_ISL_13624509:38977-37070;EPI_ISL_13889442:39152-37245;EPI_ISL_13953610:39000-37093;EPI_ISL_14181948:39178-37271;EPI_ISL_14838587:41052-39145;EPI_ISL_15263355:39033-37126;EPI_ISL_15269699:39002-37095;EPI_ISL_15293002:39275-37368;EPI_ISL_15641561:38991-37084;EPI_ISL_15831211:39040-37133;EPI_ISL_15992098:39055-37148;EPI_ISL_16233784:39051-37144;EPI_ISL_16946400:39040-37133;EPI_ISL_17048206:38690-36783;EPI_ISL_17703567:39055-37148;EPI_ISL_17718458:39033-37126;EPI_ISL_18213374:39030-37123;EPI_ISL_18360394:38691-36784;EPI_ISL_18557816:39031-37124;EPI_ISL_18642365:39033-37126;EPI_ISL_18659846:39003-37096;EPI_ISL_18744049:39035-37128;EPI_ISL_19022858:39030-37123;EPI_ISL_19028778:38947-37040;EPI_ISL_19151800:39031-37124;EPI_ISL_19159116:39033-37126;EPI_ISL_19350788:39926-38019;EPI_ISL_19359677:39030-37123;EPI_ISL_19446306:39025-37118;EPI_ISL_19467692:41331-39424;JX878411.1:42046-40139;JX878425.1:41560-39653;JX878426.1:41005-39098;KC257460.1:40976-39069;KJ642612.1:39291-37384;KJ642613.1:40867-38960;KJ642616.1:40672-38765;KJ642618.1:38459-36552;KP849469.1:41368-39461;KP849471.1:41294-39387;LC831698.1:39031-37124;MN648051.1:39253-37346;MN702445.1:34713-32806;MN702446.1:40858-38951;MN702447.1:34224-32317;MN702449.1:34724-32817;MN702452.1:40897-38990;MT903338.1:39196-37289;MT903340.1:39033-37126;MT903341.1:30566-28659;MT903342.1:39048-37141;MT903347.1:40991-39084;ON568298.1:39179-37272;ON622713.1:39265-37358;ON622722.2:39036-37129;ON631963.1:39056-37149;ON649879.1:38866-36959;ON676707.1:39086-37179;ON755039.1:38696-36789;ON808413.1:39036-37129;ON880420.1:39263-37356;ON911481.2:39053-37146;ON918656.1:39068-37161;ON959133.1:39157-37250;ON959136.1:39158-37251;ON983168.1:37732-35825;OP019276.1:39030-37123;OP150924.1:39171-37264;OP160532.1:38996-37089;OP235282.1:39030-37123;OP289782.1:39033-37126;OP331335.1:39050-37143;OP382479.1:39037-37130;OP422341.1:39050-37143;OP434519.1:39029-37122;OP535324.1:39031-37124;OP535333.1:39140-37233;OP536770.1:39032-37125;OP597769.1:39054-37147;OP604566.1:39033-37126;OP612682.1:38959-37052;OP612684.1:39023-37116;OP612686.1:39023-37116;OP696841.1:36307-34400;OP879722.1:38256-36349;OQ419007.1:38266-36359;OQ419012.1:38258-36351;OQ419031.1:38237-36330;OQ419047.1:38266-36359;OQ451898.1:38118-36211;OQ451900.1:39031-37124;OQ581853.1:39057-37150;OQ581855.1:39138-37231;OQ721951.1:39071-37164;OQ721954.1:39028-37121;OR035652.1:38980-37073;OR035664.1:38980-37073;OR035702.1:38980-37073;OR146272.1:38996-37089;OR146330.1:39041-37134;OR264364.1:39051-37144;OR427332.1:39051-37144;OR459777.1:39035-37128;OR459797.1:39050-37143;OR943698.1:41033-39126;PP098589.2:39032-37125;PP496048.1:39036-37129;PP601206.1:41050-39143;PP776553.1:38957-37050;PP852971.1:39030-37123;PP852983.1:39036-37129;PP853000.1:38997-37090;PP853005.1:39005-37098;PP853007.1:39038-37131;PP853013.1:39011-37104;PP853027.1:39042-37135;PP853045.1:39023-37116;PP853048.1:39042-37135;PP860026.1:39040-37133;PP860027.1:39065-37158;PP860028.1:39006-37099;PP860031.1:39059-37152;PP860033.1:39064-37157;PQ153226.1:39030-37123;PQ178862.2:39903-37996;PQ221506.1:34873-32966;PQ221833.1:41042-39135;PQ340477.1:39013-37106;PQ340478.1:38998-37091;PQ340480.1:39077-37170;PQ340483.1:39061-37154;PQ340485.1:39047-37140;PQ340486.1:39062-37155;PQ340487.1:39087-37180;PQ340489.1:39062-37155;PQ340490.1:39013-37106;PQ340491.1:39071-37164;PQ340493.1:39047-37140;PQ340495.1:39077-37170;PQ340496.1:39110-37203;PQ368489.1:39033-37126;PQ451835.1:39121-37214;PQ451836.1:39053-37146;PQ451838.1:39104-37197;PQ451841.1:39064-37157;PQ451842.1:39016-37109 |
| OG0000187 | AY603973.1:36634-35996;AY741551.1:36638-36000;AY753185.1:36877-36239;DQ011154.1:36854-36216;DQ011155.1:36835-36197;EPI_ISL_13331598:34705-34067;EPI_ISL_13363142:34698-34060;EPI_ISL_13374487:34713-34075;EPI_ISL_13502582:34698-34060;EPI_ISL_13508471:34790-34152;EPI_ISL_13607904:34689-34051;EPI_ISL_13624509:34633-33995;EPI_ISL_13889442:34808-34170;EPI_ISL_13953610:34656-34018;EPI_ISL_14181948:34834-34196;EPI_ISL_14838587:36708-36070;EPI_ISL_15263355:34689-34051;EPI_ISL_15269699:34658-34020;EPI_ISL_15293002:34931-34293;EPI_ISL_15641561:34647-34009;EPI_ISL_15831211:34696-34058;EPI_ISL_15992098:34711-34073;EPI_ISL_16233784:34707-34069;EPI_ISL_16946400:34696-34058;EPI_ISL_17048206:34346-33708;EPI_ISL_17703567:34711-34073;EPI_ISL_17718458:34689-34051;EPI_ISL_18213374:34686-34048;EPI_ISL_18360394:34347-33709;EPI_ISL_18557816:34687-34049;EPI_ISL_18642365:34689-34051;EPI_ISL_18659846:34659-34021;EPI_ISL_18744049:34691-34053;EPI_ISL_19022858:34686-34048;EPI_ISL_19028778:34603-33965;EPI_ISL_19151800:34687-34049;EPI_ISL_19159116:34689-34051;EPI_ISL_19350788:35582-34944;EPI_ISL_19359677:34686-34048;EPI_ISL_19446306:34681-34043;EPI_ISL_19467692:36987-36349;JX878411.1:37702-37064;JX878425.1:37216-36578;JX878426.1:36661-36023;KC257460.1:36632-35994;KJ642612.1:34947-34309;KJ642613.1:36523-35885;KJ642616.1:36328-35690;KJ642618.1:34115-33477;KP849469.1:37024-36386;KP849471.1:36950-36312;LC831698.1:34687-34049;MN648051.1:34909-34271;MN702445.1:30369-29731;MN702446.1:36515-35877;MN702447.1:29880-29242;MN702449.1:30380-29742;MN702452.1:36553-35915;MT903338.1:34852-34214;MT903340.1:34689-34051;MT903341.1:26222-25584;MT903342.1:34704-34066;MT903347.1:36647-36009;ON568298.1:34835-34197;ON622713.1:34921-34283;ON622722.2:34692-34054;ON631963.1:34712-34074;ON649879.1:34522-33884;ON676707.1:34742-34104;ON755039.1:34352-33714;ON808413.1:34692-34054;ON880420.1:34919-34281;ON911481.2:34709-34071;ON918656.1:34724-34086;ON959133.1:34813-34175;ON959136.1:34814-34176;ON983168.1:33388-32750;OP019276.1:34686-34048;OP150924.1:34827-34189;OP160532.1:34652-34014;OP235282.1:34686-34048;OP289782.1:34689-34051;OP331335.1:34706-34068;OP382479.1:34693-34055;OP422341.1:34706-34068;OP434519.1:34685-34047;OP535324.1:34687-34049;OP535333.1:34796-34158;OP536770.1:34688-34050;OP597769.1:34710-34072;OP604566.1:34689-34051;OP612682.1:34615-33977;OP612684.1:34679-34041;OP612686.1:34679-34041;OP696841.1:31963-31325;OP879722.1:33912-33274;OQ419007.1:33922-33284;OQ419012.1:33914-33276;OQ419031.1:33893-33255;OQ419047.1:33922-33284;OQ451898.1:33774-33136;OQ451900.1:34687-34049;OQ581853.1:34713-34075;OQ581855.1:34794-34156;OQ721951.1:34727-34089;OQ721954.1:34684-34046;OR035652.1:34636-33998;OR035664.1:34636-33998;OR035702.1:34636-33998;OR146272.1:34652-34014;OR146330.1:34697-34059;OR264364.1:34707-34069;OR427332.1:34707-34069;OR459777.1:34691-34053;OR459797.1:34706-34068;OR943698.1:36689-36051;PP098589.2:34688-34050;PP496048.1:34692-34054;PP601206.1:36706-36068;PP776553.1:34613-33975;PP852971.1:34686-34048;PP852983.1:34692-34054;PP853000.1:34653-34015;PP853005.1:34661-34023;PP853007.1:34694-34056;PP853013.1:34667-34029;PP853027.1:34698-34060;PP853045.1:34679-34041;PP853048.1:34698-34060;PP860026.1:34696-34058;PP860027.1:34721-34083;PP860028.1:34662-34024;PP860031.1:34715-34077;PP860033.1:34720-34082;PQ153226.1:34686-34048;PQ178862.2:35559-34921;PQ221506.1:30529-29891;PQ221833.1:36698-36060;PQ340477.1:34669-34031;PQ340478.1:34654-34016;PQ340480.1:34733-34095;PQ340483.1:34717-34079;PQ340485.1:34703-34065;PQ340486.1:34718-34080;PQ340487.1:34743-34105;PQ340489.1:34718-34080;PQ340490.1:34669-34031;PQ340491.1:34727-34089;PQ340493.1:34703-34065;PQ340495.1:34733-34095;PQ340496.1:34766-34128;PQ368489.1:34689-34051;PQ451835.1:34777-34139;PQ451836.1:34709-34071;PQ451838.1:34760-34122;PQ451841.1:34720-34082;PQ451842.1:34672-34034 |
| OG0000188 | AY603973.1:35593-35369;AY741551.1:35597-35373;AY753185.1:35836-35612;DQ011154.1:35813-35589;DQ011155.1:35794-35570;EPI_ISL_13331598:33664-33440;EPI_ISL_13363142:33657-33433;EPI_ISL_13374487:33672-33448;EPI_ISL_13502582:33657-33433;EPI_ISL_13508471:33749-33525;EPI_ISL_13607904:33648-33424;EPI_ISL_13624509:33592-33368;EPI_ISL_13889442:33767-33543;EPI_ISL_13953610:33615-33391;EPI_ISL_14181948:33793-33569;EPI_ISL_14838587:35667-35443;EPI_ISL_15263355:33648-33424;EPI_ISL_15269699:33617-33393;EPI_ISL_15293002:33890-33666;EPI_ISL_15641561:33606-33382;EPI_ISL_15831211:33655-33431;EPI_ISL_15992098:33670-33446;EPI_ISL_16233784:33666-33442;EPI_ISL_16946400:33655-33431;EPI_ISL_17048206:33305-33081;EPI_ISL_17703567:33670-33446;EPI_ISL_17718458:33648-33424;EPI_ISL_18213374:33645-33421;EPI_ISL_18360394:33306-33082;EPI_ISL_18557816:33646-33422;EPI_ISL_18642365:33648-33424;EPI_ISL_18659846:33618-33394;EPI_ISL_18744049:33650-33426;EPI_ISL_19022858:33645-33421;EPI_ISL_19028778:33562-33338;EPI_ISL_19151800:33646-33422;EPI_ISL_19159116:33648-33424;EPI_ISL_19350788:34541-34317;EPI_ISL_19359677:33645-33421;EPI_ISL_19446306:33640-33416;EPI_ISL_19467692:35946-35722;JX878411.1:36661-36437;JX878425.1:36175-35951;JX878426.1:35620-35396;KC257460.1:35591-35367;KJ642612.1:33906-33682;KJ642613.1:35482-35258;KJ642616.1:35287-35063;KJ642618.1:33074-32850;KP849469.1:35983-35759;KP849471.1:35909-35685;LC831698.1:33646-33422;MN648051.1:33868-33644;MN702445.1:29328-29104;MN702446.1:35474-35250;MN702447.1:28839-28615;MN702449.1:29339-29115;MN702452.1:35512-35288;MT903338.1:33811-33587;MT903340.1:33648-33424;MT903341.1:25181-24957;MT903342.1:33663-33439;MT903347.1:35606-35382;ON568298.1:33794-33570;ON622713.1:33880-33656;ON622722.2:33651-33427;ON631963.1:33671-33447;ON649879.1:33481-33257;ON676707.1:33701-33477;ON755039.1:33311-33087;ON808413.1:33651-33427;ON880420.1:33878-33654;ON911481.2:33668-33444;ON918656.1:33683-33459;ON959133.1:33772-33548;ON959136.1:33773-33549;ON983168.1:32347-32123;OP019276.1:33645-33421;OP150924.1:33786-33562;OP160532.1:33611-33387;OP235282.1:33645-33421;OP289782.1:33648-33424;OP331335.1:33665-33441;OP382479.1:33652-33428;OP422341.1:33665-33441;OP434519.1:33644-33420;OP535324.1:33646-33422;OP535333.1:33755-33531;OP536770.1:33647-33423;OP597769.1:33669-33445;OP604566.1:33648-33424;OP612682.1:33574-33350;OP612684.1:33638-33414;OP612686.1:33638-33414;OP696841.1:30922-30698;OP879722.1:32871-32647;OQ419007.1:32881-32657;OQ419012.1:32873-32649;OQ419031.1:32852-32628;OQ419047.1:32881-32657;OQ451898.1:32733-32509;OQ451900.1:33646-33422;OQ581853.1:33672-33448;OQ581855.1:33753-33529;OQ721951.1:33686-33462;OQ721954.1:33643-33419;OR035652.1:33595-33371;OR035664.1:33595-33371;OR035702.1:33595-33371;OR146272.1:33611-33387;OR146330.1:33656-33432;OR264364.1:33666-33442;OR427332.1:33666-33442;OR459777.1:33650-33426;OR459797.1:33665-33441;OR943698.1:35648-35424;PP098589.2:33647-33423;PP496048.1:33651-33427;PP601206.1:35665-35441;PP776553.1:33572-33348;PP852971.1:33645-33421;PP852983.1:33651-33427;PP853000.1:33612-33388;PP853005.1:33620-33396;PP853007.1:33653-33429;PP853013.1:33626-33402;PP853027.1:33657-33433;PP853045.1:33638-33414;PP853048.1:33657-33433;PP860026.1:33655-33431;PP860027.1:33680-33456;PP860028.1:33621-33397;PP860031.1:33674-33450;PP860033.1:33679-33455;PQ153226.1:33645-33421;PQ178862.2:34518-34294;PQ221506.1:29488-29264;PQ221833.1:35657-35433;PQ340477.1:33628-33404;PQ340478.1:33613-33389;PQ340480.1:33692-33468;PQ340483.1:33676-33452;PQ340485.1:33662-33438;PQ340486.1:33677-33453;PQ340487.1:33702-33478;PQ340489.1:33677-33453;PQ340490.1:33628-33404;PQ340491.1:33686-33462;PQ340493.1:33662-33438;PQ340495.1:33692-33468;PQ340496.1:33725-33501;PQ368489.1:33648-33424;PQ451835.1:33736-33512;PQ451836.1:33668-33444;PQ451838.1:33719-33495;PQ451841.1:33679-33455;PQ451842.1:33631-33407 |
| OG0000189 | AY603973.1:35353-35132;AY741551.1:35357-35136;AY753185.1:35596-35375;DQ011154.1:35573-35352;DQ011155.1:35554-35333;EPI_ISL_13331598:33423-33196;EPI_ISL_13363142:33416-33189;EPI_ISL_13374487:33431-33204;EPI_ISL_13502582:33416-33189;EPI_ISL_13508471:33508-33281;EPI_ISL_13607904:33407-33180;EPI_ISL_13624509:33351-33124;EPI_ISL_13889442:33526-33299;EPI_ISL_13953610:33374-33147;EPI_ISL_14181948:33552-33325;EPI_ISL_14838587:35427-35206;EPI_ISL_15263355:33407-33180;EPI_ISL_15269699:33376-33149;EPI_ISL_15293002:33649-33422;EPI_ISL_15641561:33365-33138;EPI_ISL_15831211:33414-33187;EPI_ISL_15992098:33429-33202;EPI_ISL_16233784:33425-33198;EPI_ISL_16946400:33414-33187;EPI_ISL_17048206:33064-32837;EPI_ISL_17703567:33429-33202;EPI_ISL_17718458:33407-33180;EPI_ISL_18213374:33404-33177;EPI_ISL_18360394:33065-32838;EPI_ISL_18557816:33405-33178;EPI_ISL_18642365:33407-33180;EPI_ISL_18659846:33377-33150;EPI_ISL_18744049:33409-33182;EPI_ISL_19022858:33404-33177;EPI_ISL_19028778:33321-33094;EPI_ISL_19151800:33405-33178;EPI_ISL_19159116:33407-33180;EPI_ISL_19350788:34301-34080;EPI_ISL_19359677:33404-33177;EPI_ISL_19446306:33399-33172;EPI_ISL_19467692:35706-35485;JX878411.1:36421-36200;JX878425.1:35935-35714;JX878426.1:35380-35159;KC257460.1:35351-35130;KJ642612.1:33666-33445;KJ642613.1:35242-35021;KJ642616.1:35047-34826;KJ642618.1:32834-32613;KP849469.1:35743-35522;KP849471.1:35669-35448;LC831698.1:33405-33178;MN648051.1:33627-33400;MN702445.1:29088-28867;MN702446.1:35234-35013;MN702447.1:28599-28378;MN702449.1:29099-28878;MN702452.1:35272-35051;MT903338.1:33570-33343;MT903340.1:33407-33180;MT903341.1:24940-24713;MT903342.1:33422-33195;MT903347.1:35366-35145;ON568298.1:33553-33326;ON622713.1:33639-33412;ON622722.2:33410-33183;ON631963.1:33430-33203;ON649879.1:33240-33013;ON676707.1:33460-33233;ON755039.1:33070-32843;ON808413.1:33410-33183;ON880420.1:33637-33410;ON911481.2:33427-33200;ON918656.1:33442-33215;ON959133.1:33531-33304;ON959136.1:33532-33305;ON983168.1:32106-31879;OP019276.1:33404-33177;OP150924.1:33545-33318;OP160532.1:33370-33143;OP235282.1:33404-33177;OP289782.1:33407-33180;OP331335.1:33424-33197;OP382479.1:33411-33184;OP422341.1:33424-33197;OP434519.1:33403-33176;OP535324.1:33405-33178;OP535333.1:33514-33287;OP536770.1:33406-33179;OP597769.1:33428-33201;OP604566.1:33407-33180;OP612682.1:33333-33106;OP612684.1:33397-33170;OP612686.1:33397-33170;OP696841.1:30681-30454;OP879722.1:32630-32403;OQ419007.1:32640-32413;OQ419012.1:32632-32405;OQ419031.1:32611-32384;OQ419047.1:32640-32413;OQ451898.1:32492-32265;OQ451900.1:33405-33178;OQ581853.1:33431-33204;OQ581855.1:33512-33285;OQ721951.1:33445-33218;OQ721954.1:33402-33175;OR035652.1:33354-33127;OR035664.1:33354-33127;OR035702.1:33354-33127;OR146272.1:33370-33143;OR146330.1:33415-33188;OR264364.1:33425-33198;OR427332.1:33425-33198;OR459777.1:33409-33182;OR459797.1:33424-33197;OR943698.1:35408-35187;PP098589.2:33406-33179;PP496048.1:33410-33183;PP601206.1:35425-35204;PP776553.1:33331-33104;PP852971.1:33404-33177;PP852983.1:33409-33182;PP853000.1:33371-33144;PP853005.1:33379-33152;PP853007.1:33411-33184;PP853013.1:33385-33158;PP853027.1:33416-33189;PP853045.1:33397-33170;PP853048.1:33416-33189;PP860026.1:33414-33187;PP860027.1:33439-33212;PP860028.1:33380-33153;PP860031.1:33433-33212;PP860033.1:33438-33211;PQ153226.1:33404-33177;PQ178862.2:34278-34057;PQ221506.1:29248-29027;PQ221833.1:35417-35196;PQ340477.1:33387-33160;PQ340478.1:33372-33145;PQ340480.1:33451-33224;PQ340483.1:33435-33208;PQ340485.1:33421-33194;PQ340486.1:33436-33209;PQ340487.1:33461-33234;PQ340489.1:33436-33209;PQ340490.1:33387-33160;PQ340491.1:33445-33218;PQ340493.1:33421-33194;PQ340495.1:33451-33224;PQ340496.1:33484-33257;PQ368489.1:33407-33180;PQ451835.1:33495-33268;PQ451836.1:33427-33200;PQ451838.1:33478-33251;PQ451841.1:33438-33211;PQ451842.1:33390-33163 |
| OG0000190 | AY603973.1:29884-29054;AY741551.1:29898-29068;AY753185.1:30127-29297;DQ011154.1:30133-29303;DQ011155.1:30119-29289;EPI_ISL_13331598:27970-27140;EPI_ISL_13363142:27963-27133;EPI_ISL_13374487:27978-27148;EPI_ISL_13502582:27963-27133;EPI_ISL_13508471:28055-27225;EPI_ISL_13607904:27954-27124;EPI_ISL_13624509:27898-27068;EPI_ISL_13889442:28073-27243;EPI_ISL_13953610:27921-27091;EPI_ISL_14181948:28099-27269;EPI_ISL_14838587:29987-29157;EPI_ISL_15263355:27954-27124;EPI_ISL_15269699:27923-27093;EPI_ISL_15293002:28196-27366;EPI_ISL_15641561:27912-27082;EPI_ISL_15831211:27961-27131;EPI_ISL_15992098:27976-27146;EPI_ISL_16233784:27972-27142;EPI_ISL_16946400:27961-27131;EPI_ISL_17048206:27611-26781;EPI_ISL_17703567:27976-27146;EPI_ISL_17718458:27954-27124;EPI_ISL_18213374:27951-27121;EPI_ISL_18360394:27612-26782;EPI_ISL_18557816:27952-27122;EPI_ISL_18642365:27954-27124;EPI_ISL_18659846:27924-27094;EPI_ISL_18744049:27956-27126;EPI_ISL_19022858:27951-27121;EPI_ISL_19028778:27868-27038;EPI_ISL_19151800:27952-27122;EPI_ISL_19159116:27954-27124;EPI_ISL_19350788:28872-28042;EPI_ISL_19359677:27951-27121;EPI_ISL_19446306:27946-27116;EPI_ISL_19467692:30266-29436;JX878411.1:30981-30151;JX878425.1:30495-29665;JX878426.1:29935-29105;KC257460.1:29916-29086;KJ642612.1:28221-27391;KJ642613.1:29802-28972;KJ642616.1:29583-28753;KJ642618.1:27389-26559;KP849469.1:30303-29473;KP849471.1:30224-29394;LC831698.1:27952-27122;MN648051.1:28174-27344;MN702445.1:23648-22818;MN702446.1:29794-28964;MN702447.1:23159-22329;MN702449.1:23659-22829;MN702452.1:29832-29002;MT903338.1:28117-27287;MT903340.1:27954-27124;MT903341.1:19487-18657;MT903342.1:27969-27139;MT903347.1:29882-29052;ON568298.1:28100-27270;ON622713.1:28186-27356;ON622722.2:27957-27127;ON631963.1:27977-27147;ON649879.1:27787-26957;ON676707.1:28007-27177;ON755039.1:27617-26787;ON808413.1:27957-27127;ON880420.1:28184-27354;ON911481.2:27974-27144;ON918656.1:27989-27159;ON959133.1:28078-27248;ON959136.1:28079-27249;ON983168.1:26653-25823;OP019276.1:27951-27121;OP150924.1:28092-27262;OP160532.1:27917-27087;OP235282.1:27951-27121;OP289782.1:27954-27124;OP331335.1:27971-27141;OP382479.1:27958-27128;OP422341.1:27971-27141;OP434519.1:27950-27120;OP535324.1:27952-27122;OP535333.1:28061-27231;OP536770.1:27953-27123;OP597769.1:27975-27145;OP604566.1:27954-27124;OP612682.1:27880-27050;OP612684.1:27944-27114;OP612686.1:27944-27114;OP696841.1:25228-24398;OP879722.1:27177-26347;OQ419007.1:27187-26357;OQ419012.1:27179-26349;OQ419031.1:27158-26328;OQ419047.1:27187-26357;OQ451898.1:27039-26209;OQ451900.1:27952-27122;OQ581853.1:27978-27148;OQ581855.1:28059-27229;OQ721951.1:27992-27162;OQ721954.1:27949-27119;OR035652.1:27901-27071;OR035664.1:27901-27071;OR035702.1:27901-27071;OR146272.1:27917-27087;OR146330.1:27962-27132;OR264364.1:27972-27142;OR427332.1:27972-27142;OR459777.1:27956-27126;OR459797.1:27971-27141;OR943698.1:29963-29133;PP098589.2:27953-27123;PP496048.1:27957-27127;PP601206.1:29985-29155;PP776553.1:27878-27048;PP852971.1:27951-27121;PP852983.1:27956-27126;PP853000.1:27918-27088;PP853005.1:27926-27096;PP853007.1:27958-27128;PP853013.1:27932-27102;PP853027.1:27963-27133;PP853045.1:27944-27114;PP853048.1:27963-27133;PP860026.1:27958-27128;PP860027.1:27969-27139;PP860028.1:27925-27095;PP860031.1:27979-27149;PP860033.1:27968-27138;PQ153226.1:27951-27121;PQ178862.2:28849-28019;PQ221506.1:23805-22975;PQ221833.1:29977-29147;PQ340477.1:27934-27104;PQ340478.1:27919-27089;PQ340480.1:27998-27168;PQ340483.1:27982-27152;PQ340485.1:27968-27138;PQ340486.1:27983-27153;PQ340487.1:28008-27178;PQ340489.1:27983-27153;PQ340490.1:27934-27104;PQ340491.1:27992-27162;PQ340493.1:27968-27138;PQ340495.1:27998-27168;PQ340496.1:28031-27201;PQ368489.1:27954-27124;PQ451835.1:28042-27212;PQ451836.1:27974-27144;PQ451838.1:28025-27195;PQ451841.1:27985-27155;PQ451842.1:27937-27107 |
| OG0000191 | AY603973.1:29026-27752;AY741551.1:29040-27766;AY753185.1:29269-27995;DQ011154.1:29275-28001;DQ011155.1:29261-27987;EPI_ISL_13331598:27112-25838;EPI_ISL_13363142:27105-25831;EPI_ISL_13374487:27120-25846;EPI_ISL_13502582:27105-25831;EPI_ISL_13508471:27197-25923;EPI_ISL_13607904:27096-25822;EPI_ISL_13624509:27040-25766;EPI_ISL_13889442:27215-25941;EPI_ISL_13953610:27063-25789;EPI_ISL_14181948:27241-25967;EPI_ISL_14838587:29129-27855;EPI_ISL_15263355:27096-25822;EPI_ISL_15269699:27065-25791;EPI_ISL_15293002:27338-26064;EPI_ISL_15641561:27054-25780;EPI_ISL_15831211:27103-25829;EPI_ISL_15992098:27118-25844;EPI_ISL_16233784:27114-25840;EPI_ISL_16946400:27103-25829;EPI_ISL_17048206:26753-25479;EPI_ISL_17703567:27118-25844;EPI_ISL_17718458:27096-25822;EPI_ISL_18213374:27093-25819;EPI_ISL_18360394:26754-25480;EPI_ISL_18557816:27094-25820;EPI_ISL_18642365:27096-25822;EPI_ISL_18659846:27066-25792;EPI_ISL_18744049:27098-25824;EPI_ISL_19022858:27093-25819;EPI_ISL_19028778:27010-25736;EPI_ISL_19151800:27094-25820;EPI_ISL_19159116:27096-25822;EPI_ISL_19350788:28014-26740;EPI_ISL_19359677:27093-25819;EPI_ISL_19446306:27088-25814;EPI_ISL_19467692:29408-28134;JX878411.1:30123-28849;JX878425.1:29637-28363;JX878426.1:29077-27803;KC257460.1:29058-27784;KJ642612.1:27363-26089;KJ642613.1:28944-27670;KJ642616.1:28725-27451;KJ642618.1:26531-25257;KP849469.1:29445-28171;KP849471.1:29366-28092;LC831698.1:27094-25820;MN648051.1:27316-26042;MN702445.1:22790-21516;MN702446.1:28936-27662;MN702447.1:22301-21027;MN702449.1:22801-21527;MN702452.1:28974-27700;MT903338.1:27259-25985;MT903340.1:27096-25822;MT903341.1:18629-17355;MT903342.1:27111-25837;MT903347.1:29024-27750;ON568298.1:27242-25968;ON622713.1:27328-26054;ON622722.2:27099-25825;ON631963.1:27119-25845;ON649879.1:26929-25655;ON676707.1:27149-25875;ON755039.1:26759-25485;ON808413.1:27099-25825;ON880420.1:27326-26052;ON911481.2:27116-25842;ON918656.1:27131-25857;ON959133.1:27220-25946;ON959136.1:27221-25947;ON983168.1:25795-24521;OP019276.1:27093-25819;OP150924.1:27234-25960;OP160532.1:27059-25785;OP235282.1:27093-25819;OP289782.1:27096-25822;OP331335.1:27113-25839;OP382479.1:27100-25826;OP422341.1:27113-25839;OP434519.1:27092-25818;OP535324.1:27094-25820;OP535333.1:27203-25929;OP536770.1:27095-25821;OP597769.1:27117-25843;OP604566.1:27096-25822;OP612682.1:27022-25748;OP612684.1:27086-25812;OP612686.1:27086-25812;OP696841.1:24370-23096;OP879722.1:26319-25045;OQ419007.1:26329-25055;OQ419012.1:26321-25047;OQ419031.1:26300-25026;OQ419047.1:26329-25055;OQ451898.1:26181-24907;OQ451900.1:27094-25820;OQ581853.1:27120-25846;OQ581855.1:27201-25927;OQ721951.1:27134-25860;OQ721954.1:27091-25817;OR035652.1:27043-25769;OR035664.1:27043-25769;OR035702.1:27043-25769;OR146272.1:27059-25785;OR146330.1:27104-25830;OR264364.1:27114-25840;OR427332.1:27114-25840;OR459777.1:27098-25824;OR459797.1:27113-25839;OR943698.1:29105-27831;PP098589.2:27095-25821;PP496048.1:27099-25825;PP601206.1:29127-27853;PP776553.1:27020-25746;PP852971.1:27093-25819;PP852983.1:27098-25824;PP853000.1:27060-25786;PP853005.1:27068-25794;PP853007.1:27100-25826;PP853013.1:27074-25800;PP853027.1:27105-25831;PP853045.1:27086-25812;PP853048.1:27105-25831;PP860026.1:27100-25826;PP860027.1:27111-25837;PP860028.1:27067-25793;PP860031.1:27121-25847;PP860033.1:27110-25836;PQ153226.1:27093-25819;PQ178862.2:27991-26717;PQ221506.1:22947-21673;PQ221833.1:29119-27845;PQ340477.1:27076-25802;PQ340478.1:27061-25787;PQ340480.1:27140-25866;PQ340483.1:27124-25850;PQ340485.1:27110-25836;PQ340486.1:27125-25851;PQ340487.1:27150-25876;PQ340489.1:27125-25851;PQ340490.1:27076-25802;PQ340491.1:27134-25860;PQ340493.1:27110-25836;PQ340495.1:27140-25866;PQ340496.1:27173-25899;PQ368489.1:27096-25822;PQ451835.1:27184-25910;PQ451836.1:27116-25842;PQ451838.1:27167-25893;PQ451841.1:27127-25853;PQ451842.1:27079-25805 |
| OG0000192 | AY603973.1:19513-18893;AY741551.1:19380-18760;AY753185.1:19756-19136;DQ011154.1:18028-17408;DQ011155.1:18009-17389;EPI_ISL_13331598:17848-17228;EPI_ISL_13363142:17841-17221;EPI_ISL_13374487:17856-17236;EPI_ISL_13502582:17841-17221;EPI_ISL_13508471:17933-17313;EPI_ISL_13607904:17832-17212;EPI_ISL_13624509:17776-17156;EPI_ISL_13889442:17951-17331;EPI_ISL_13953610:17832-17212;EPI_ISL_14181948:17977-17357;EPI_ISL_14838587:17882-17262;EPI_ISL_15263355:17832-17212;EPI_ISL_15269699:17801-17181;EPI_ISL_15293002:18074-17454;EPI_ISL_15641561:17790-17170;EPI_ISL_15831211:17839-17219;EPI_ISL_15992098:17854-17234;EPI_ISL_16233784:17850-17230;EPI_ISL_16946400:17839-17219;EPI_ISL_17048206:17489-16869;EPI_ISL_17703567:17854-17234;EPI_ISL_17718458:17832-17212;EPI_ISL_18213374:17829-17209;EPI_ISL_18360394:17490-16870;EPI_ISL_18557816:17830-17210;EPI_ISL_18642365:17832-17212;EPI_ISL_18659846:17802-17182;EPI_ISL_18744049:17834-17214;EPI_ISL_19022858:17829-17209;EPI_ISL_19028778:17746-17126;EPI_ISL_19151800:17830-17210;EPI_ISL_19159116:17832-17212;EPI_ISL_19350788:17901-17281;EPI_ISL_19359677:17829-17209;EPI_ISL_19446306:17824-17204;EPI_ISL_19467692:18160-17540;JX878411.1:18874-18254;JX878425.1:18388-17768;JX878426.1:17831-17211;KC257460.1:17808-17188;KJ642612.1:16116-15496;KJ642613.1:17697-17077;KJ642616.1:19330-18710;KJ642618.1:15287-14667;KP849469.1:18198-17578;KP849471.1:18119-17499;LC831698.1:17830-17210;MN648051.1:18052-17432;MN702445.1:11539-10919;MN702446.1:17685-17065;MN702447.1:11050-10430;MN702449.1:11547-10927;MN702452.1:17727-17107;MT903338.1:17995-17375;MT903340.1:17832-17212;MT903341.1:9365-8745;MT903342.1:17847-17227;MT903347.1:19713-19093;ON568298.1:17978-17358;ON622713.1:18064-17444;ON622722.2:17835-17215;ON631963.1:17855-17235;ON649879.1:17665-17045;ON676707.1:17885-17265;ON755039.1:17495-16875;ON808413.1:17835-17215;ON880420.1:18062-17442;ON911481.2:17852-17232;ON918656.1:17867-17247;ON959133.1:17956-17336;ON959136.1:17957-17337;ON983168.1:16531-15911;OP019276.1:17829-17209;OP150924.1:17970-17350;OP160532.1:17795-17175;OP235282.1:17829-17209;OP289782.1:17832-17212;OP331335.1:17849-17229;OP382479.1:17836-17216;OP422341.1:17849-17229;OP434519.1:17828-17208;OP535324.1:17830-17210;OP535333.1:17939-17319;OP536770.1:17831-17211;OP597769.1:17853-17233;OP604566.1:17832-17212;OP612682.1:17758-17138;OP612684.1:17822-17202;OP612686.1:17822-17202;OP696841.1:15106-14486;OP879722.1:17055-16435;OQ419007.1:17065-16445;OQ419012.1:17057-16437;OQ419031.1:17062-16442;OQ419047.1:17065-16445;OQ451898.1:16917-16297;OQ451900.1:17830-17210;OQ581853.1:17856-17236;OQ581855.1:17937-17317;OQ721951.1:17870-17250;OQ721954.1:17827-17207;OR035652.1:17779-17159;OR035664.1:17779-17159;OR035702.1:17779-17159;OR146272.1:17795-17175;OR146330.1:17840-17220;OR264364.1:17850-17230;OR427332.1:17850-17230;OR459777.1:17834-17214;OR459797.1:17849-17229;OR943698.1:17865-17245;PP098589.2:17831-17211;PP496048.1:17835-17215;PP601206.1:17880-17260;PP776553.1:17780-17160;PP852971.1:17829-17209;PP852983.1:17834-17214;PP853000.1:17796-17176;PP853005.1:17804-17184;PP853007.1:17836-17216;PP853013.1:17810-17190;PP853027.1:17841-17221;PP853045.1:17840-17220;PP853048.1:17841-17221;PP860026.1:17836-17216;PP860027.1:17847-17227;PP860028.1:17803-17183;PP860031.1:17857-17237;PP860033.1:17846-17226;PQ153226.1:17829-17209;PQ178862.2:17878-17258;PQ221506.1:11702-11082;PQ221833.1:17872-17252;PQ340477.1:17812-17192;PQ340478.1:17797-17177;PQ340480.1:17876-17256;PQ340483.1:17860-17240;PQ340485.1:17846-17226;PQ340486.1:17861-17241;PQ340487.1:17886-17266;PQ340489.1:17861-17241;PQ340490.1:17812-17192;PQ340491.1:17870-17250;PQ340493.1:17846-17226;PQ340495.1:17876-17256;PQ340496.1:17909-17289;PQ368489.1:17832-17212;PQ451835.1:17920-17300;PQ451836.1:17852-17232;PQ451838.1:17903-17283;PQ451841.1:17863-17243;PQ451842.1:17815-17195 |
| OG0000193 | AY603973.1:152643-152810;AY741551.1:152717-152884;AY753185.1:152898-153065;DQ011154.1:152809-152976;DQ011155.1:152751-152918;EPI_ISL_13331598:150762-150929;EPI_ISL_13363142:150750-150917;EPI_ISL_13374487:150783-150950;EPI_ISL_13502582:150778-150945;EPI_ISL_13508471:150844-151011;EPI_ISL_13607904:150749-150916;EPI_ISL_13624509:150684-150851;EPI_ISL_13889442:150877-151044;EPI_ISL_13953610:150677-150844;EPI_ISL_14181948:150907-151074;EPI_ISL_15263355:150743-150910;EPI_ISL_15269699:150712-150879;EPI_ISL_15293002:150981-151148;EPI_ISL_15641561:150698-150865;EPI_ISL_15831211:150752-150919;EPI_ISL_15992098:150792-150959;EPI_ISL_16233784:150764-150931;EPI_ISL_16946400:150754-150921;EPI_ISL_17048206:150418-150585;EPI_ISL_17703567:150765-150932;EPI_ISL_17718458:150745-150912;EPI_ISL_18213374:150756-150923;EPI_ISL_18360394:150063-150230;EPI_ISL_18557816:150759-150926;EPI_ISL_18642365:150754-150921;EPI_ISL_18659846:150713-150880;EPI_ISL_18744049:150762-150929;EPI_ISL_19022858:150719-150886;EPI_ISL_19028778:150671-150838;EPI_ISL_19151800:150755-150922;EPI_ISL_19159116:150740-150907;EPI_ISL_19350788:151408-151575;EPI_ISL_19359677:150755-150922;EPI_ISL_19446306:150742-150909;EPI_ISL_19467692:152949-153116;JX878411.1:153650-153817;JX878425.1:153138-153305;JX878426.1:152678-152845;KC257460.1:152534-152701;KJ642612.1:150868-151035;KJ642613.1:152417-152584;KJ642616.1:152365-152532;KJ642618.1:150333-150500;KP849469.1:152966-153133;KP849471.1:152899-153066;LC831698.1:150738-150905;MN648051.1:150981-151148;MN702445.1:146285-146452;MN702446.1:152449-152616;MN702447.1:145812-145979;MN702449.1:146323-146490;MN702452.1:152500-152667;MT903338.1:150914-151081;MT903340.1:150767-150934;MT903341.1:142308-142475;MT903342.1:150804-150971;MT903347.1:152710-152877;ON568298.1:150908-151075;ON622713.1:150972-151139;ON622722.2:150760-150927;ON631963.1:150769-150936;ON649879.1:150575-150742;ON676707.1:150806-150973;ON755039.1:150397-150564;ON808413.1:150734-150901;ON880420.1:150970-151137;ON911481.2:150755-150922;ON918656.1:150777-150944;ON959133.1:150866-151033;ON959136.1:150867-151034;ON983168.1:149443-149610;OP019276.1:150754-150921;OP150924.1:150880-151047;OP160532.1:150703-150870;OP235282.1:150754-150921;OP289782.1:150758-150925;OP331335.1:150763-150930;OP382479.1:150747-150914;OP422341.1:150760-150927;OP434519.1:150736-150903;OP535324.1:150767-150934;OP535333.1:150874-151041;OP536770.1:150757-150924;OP597769.1:150791-150958;OP604566.1:150757-150924;OP612682.1:150692-150859;OP612684.1:150743-150910;OP612686.1:150745-150912;OP696841.1:148033-148200;OP879722.1:149980-150147;OQ419007.1:149974-150141;OQ419012.1:149948-150115;OQ419031.1:149952-150119;OQ419047.1:149954-150121;OQ451898.1:149844-150011;OQ451900.1:150758-150925;OQ581853.1:150766-150933;OQ581855.1:150865-151032;OQ721951.1:150784-150951;OQ721954.1:150757-150924;OR035652.1:150686-150853;OR035664.1:150690-150857;OR035702.1:150687-150854;OR146272.1:150706-150873;OR146330.1:150767-150934;OR264364.1:150761-150928;OR427332.1:150761-150928;OR459777.1:150750-150917;OR459797.1:150759-150926;OR943698.1:152654-152821;PP098589.2:150756-150923;PP496048.1:150760-150927;PP601206.1:152709-152876;PP776553.1:150680-150847;PP852971.1:150748-150915;PP852983.1:150765-150932;PP853000.1:150716-150883;PP853005.1:150724-150891;PP853007.1:150767-150934;PP853013.1:150739-150906;PP853027.1:150781-150948;PP853045.1:150732-150899;PP853048.1:150757-150924;PP860026.1:150752-150919;PP860027.1:150789-150956;PP860028.1:150715-150882;PP860031.1:150786-150953;PP860033.1:150772-150939;PQ153226.1:150737-150904;PQ178862.2:151387-151554;PQ221506.1:146498-146665;PQ221833.1:152685-152852;PQ340477.1:150724-150891;PQ340478.1:150706-150873;PQ340480.1:150784-150951;PQ340483.1:150770-150937;PQ340485.1:150753-150920;PQ340486.1:150771-150938;PQ340487.1:150795-150962;PQ340489.1:150770-150937;PQ340490.1:150722-150889;PQ340491.1:150777-150944;PQ340493.1:150761-150928;PQ340495.1:150787-150954;PQ340496.1:150822-150989;PQ368489.1:150770-150937;PQ451835.1:150827-150994;PQ451836.1:150765-150932;PQ451838.1:150815-150982;PQ451841.1:150778-150945;PQ451842.1:150724-150891 |
| OG0000194 | AY603973.1:11255-11983;AY741551.1:11080-11808;AY753185.1:11466-12194;DQ011154.1:9660-10388;DQ011155.1:9565-10293;EPI_ISL_13331598:9602-10330;EPI_ISL_13363142:9595-10323;EPI_ISL_13374487:9610-10338;EPI_ISL_13502582:9595-10323;EPI_ISL_13508471:9687-10415;EPI_ISL_13607904:9586-10314;EPI_ISL_13624509:9530-10258;EPI_ISL_13889442:9705-10433;EPI_ISL_13953610:9586-10314;EPI_ISL_14181948:9731-10459;EPI_ISL_14838587:9564-10292;EPI_ISL_15263355:9583-10311;EPI_ISL_15269699:9555-10283;EPI_ISL_15293002:9828-10556;EPI_ISL_15641561:9544-10272;EPI_ISL_15831211:9593-10321;EPI_ISL_15992098:9608-10336;EPI_ISL_16233784:9604-10332;EPI_ISL_16946400:9593-10321;EPI_ISL_17048206:9243-9971;EPI_ISL_17703567:9608-10336;EPI_ISL_17718458:9586-10314;EPI_ISL_18213374:9583-10311;EPI_ISL_18360394:9244-9972;EPI_ISL_18557816:9584-10312;EPI_ISL_18642365:9586-10314;EPI_ISL_18659846:9556-10284;EPI_ISL_18744049:9588-10316;EPI_ISL_19022858:9583-10311;EPI_ISL_19028778:9500-10228;EPI_ISL_19151800:9584-10312;EPI_ISL_19159116:9586-10314;EPI_ISL_19350788:9576-10304;EPI_ISL_19359677:9583-10311;EPI_ISL_19446306:9578-10306;EPI_ISL_19467692:9792-10520;JX878411.1:10505-11233;JX878425.1:9945-10673;JX878426.1:9512-10240;KC257460.1:9331-10059;KJ642612.1:7852-8580;KJ642613.1:9388-10116;KJ642616.1:11072-11800;KJ642618.1:6919-7647;KP849469.1:9767-10495;KP849471.1:9740-10468;LC831698.1:9584-10312;MN648051.1:9805-10533;MN702445.1:3230-3958;MN702446.1:9376-10104;MN702447.1:2737-3465;MN702449.1:3233-3961;MN702452.1:9414-10142;MT903338.1:9749-10477;MT903340.1:9586-10314;MT903342.1:9601-10329;MT903347.1:11346-12074;ON568298.1:9732-10460;ON622713.1:9817-10545;ON622722.2:9589-10317;ON631963.1:9609-10337;ON649879.1:9419-10147;ON676707.1:9639-10367;ON755039.1:9249-9977;ON808413.1:9589-10317;ON880420.1:9816-10544;ON911481.2:9606-10334;ON918656.1:9621-10349;ON959133.1:9710-10438;ON959136.1:9711-10439;ON983168.1:8285-9013;OP019276.1:9583-10311;OP150924.1:9724-10452;OP160532.1:9549-10277;OP235282.1:9583-10311;OP289782.1:9586-10314;OP331335.1:9603-10331;OP382479.1:9590-10318;OP422341.1:9603-10331;OP434519.1:9582-10310;OP535324.1:9584-10312;OP535333.1:9693-10421;OP536770.1:9585-10313;OP597769.1:9607-10335;OP604566.1:9586-10314;OP612682.1:9512-10240;OP612684.1:9576-10304;OP612686.1:9576-10304;OP696841.1:6887-7615;OP879722.1:8809-9537;OQ419007.1:8819-9547;OQ419012.1:8811-9539;OQ419031.1:8816-9544;OQ419047.1:8819-9547;OQ451898.1:9584-10312;OQ451900.1:9584-10312;OQ581853.1:9610-10338;OQ581855.1:9691-10419;OQ721951.1:9624-10352;OQ721954.1:9581-10309;OR035652.1:9533-10261;OR035664.1:9533-10261;OR035702.1:9533-10261;OR146272.1:9549-10277;OR146330.1:9594-10322;OR264364.1:9604-10332;OR427332.1:9604-10332;OR459777.1:9588-10316;OR459797.1:9603-10331;OR943698.1:9541-10269;PP098589.2:9585-10313;PP496048.1:9589-10317;PP601206.1:9562-10290;PP776553.1:9534-10262;PP852971.1:9583-10311;PP852983.1:9588-10316;PP853000.1:9550-10278;PP853005.1:9558-10286;PP853007.1:9590-10318;PP853013.1:9564-10292;PP853027.1:9595-10323;PP853045.1:9594-10322;PP853048.1:9595-10323;PP860026.1:9590-10318;PP860027.1:9589-10317;PP860028.1:9557-10285;PP860031.1:9601-10329;PP860033.1:9588-10316;PQ153226.1:9583-10311;PQ178862.2:9593-10321;PQ221506.1:3383-4111;PQ221833.1:9554-10282;PQ340477.1:9566-10294;PQ340478.1:9551-10279;PQ340480.1:9630-10358;PQ340483.1:9614-10342;PQ340485.1:9600-10328;PQ340486.1:9615-10343;PQ340487.1:9640-10368;PQ340489.1:9615-10343;PQ340490.1:9566-10294;PQ340491.1:9624-10352;PQ340493.1:9600-10328;PQ340495.1:9630-10358;PQ340496.1:9663-10391;PQ368489.1:9586-10314;PQ451835.1:9674-10402;PQ451836.1:9606-10334;PQ451838.1:9657-10385;PQ451841.1:9617-10345;PQ451842.1:9569-10297 |
| OG0000195 | AY603973.1:17018-17242;AY741551.1:16855-17100;AY753185.1:17264-17488;DQ011154.1:15521-15769;DQ011155.1:15501-15749;EPI_ISL_13331598:15335-15580;EPI_ISL_13363142:15328-15573;EPI_ISL_13374487:15343-15588;EPI_ISL_13502582:15328-15573;EPI_ISL_13508471:15420-15665;EPI_ISL_13607904:15319-15564;EPI_ISL_13624509:15263-15508;EPI_ISL_13889442:15438-15683;EPI_ISL_13953610:15319-15564;EPI_ISL_14181948:15464-15709;EPI_ISL_14838587:15375-15623;EPI_ISL_15263355:15316-15561;EPI_ISL_15269699:15288-15533;EPI_ISL_15293002:15561-15806;EPI_ISL_15641561:15277-15522;EPI_ISL_15831211:15326-15571;EPI_ISL_15992098:15341-15586;EPI_ISL_16233784:15337-15582;EPI_ISL_16946400:15326-15571;EPI_ISL_17048206:14976-15221;EPI_ISL_17703567:15341-15586;EPI_ISL_17718458:15319-15564;EPI_ISL_18213374:15316-15561;EPI_ISL_18360394:14977-15222;EPI_ISL_18557816:15317-15562;EPI_ISL_18642365:15319-15564;EPI_ISL_18659846:15289-15534;EPI_ISL_18744049:15321-15566;EPI_ISL_19022858:15316-15561;EPI_ISL_19028778:15233-15478;EPI_ISL_19151800:15317-15562;EPI_ISL_19159116:15319-15564;EPI_ISL_19350788:15387-15635;EPI_ISL_19359677:15316-15561;EPI_ISL_19446306:15311-15556;EPI_ISL_19467692:15653-15901;JX878411.1:16366-16614;JX878425.1:15881-16129;JX878426.1:15323-15571;KC257460.1:15300-15548;KJ642612.1:13608-13856;KJ642613.1:15189-15437;KJ642616.1:16835-17059;KJ642618.1:12779-13027;KP849469.1:15691-15939;KP849471.1:15611-15859;LC831698.1:15317-15562;MN648051.1:15539-15784;MN702445.1:9031-9279;MN702446.1:15177-15425;MN702447.1:8543-8791;MN702449.1:9039-9287;MN702452.1:15220-15468;MT903338.1:15482-15727;MT903340.1:15319-15564;MT903341.1:6852-7097;MT903342.1:15334-15579;MT903347.1:17191-17436;ON568298.1:15465-15710;ON622713.1:15551-15796;ON622722.2:15322-15567;ON631963.1:15342-15587;ON649879.1:15152-15397;ON676707.1:15372-15617;ON755039.1:14982-15227;ON808413.1:15322-15567;ON880420.1:15549-15794;ON911481.2:15339-15584;ON918656.1:15354-15599;ON959133.1:15443-15688;ON959136.1:15444-15689;ON983168.1:14018-14263;OP019276.1:15316-15561;OP150924.1:15457-15702;OP160532.1:15282-15527;OP235282.1:15316-15561;OP289782.1:15319-15564;OP331335.1:15336-15581;OP382479.1:15323-15568;OP422341.1:15336-15581;OP434519.1:15315-15560;OP535324.1:15317-15562;OP535333.1:15426-15671;OP536770.1:15318-15563;OP597769.1:15340-15585;OP604566.1:15319-15564;OP612682.1:15245-15490;OP612684.1:15309-15554;OP612686.1:15309-15554;OP696841.1:12593-12838;OP879722.1:14542-14787;OQ419007.1:14552-14797;OQ419012.1:14544-14789;OQ419031.1:14549-14794;OQ419047.1:14552-14797;OQ451898.1:14404-14649;OQ451900.1:15317-15562;OQ581853.1:15343-15588;OQ581855.1:15424-15669;OQ721951.1:15357-15602;OQ721954.1:15314-15559;OR035652.1:15266-15511;OR035664.1:15266-15511;OR035702.1:15266-15511;OR146272.1:15282-15527;OR146330.1:15327-15572;OR264364.1:15337-15582;OR427332.1:15337-15582;OR459777.1:15321-15566;OR459797.1:15336-15581;PP098589.2:15318-15563;PP496048.1:15322-15567;PP601206.1:15373-15621;PP776553.1:15267-15512;PP852971.1:15316-15561;PP852983.1:15321-15566;PP853000.1:15283-15528;PP853005.1:15291-15536;PP853007.1:15323-15568;PP853013.1:15297-15542;PP853027.1:15328-15573;PP853045.1:15327-15572;PP853048.1:15328-15573;PP860026.1:15323-15568;PP860027.1:15322-15567;PP860028.1:15290-15535;PP860031.1:15329-15574;PP860033.1:15321-15566;PQ153226.1:15316-15561;PQ178862.2:15364-15612;PQ221506.1:9194-9442;PQ221833.1:15365-15613;PQ340477.1:15299-15544;PQ340478.1:15284-15529;PQ340480.1:15363-15608;PQ340483.1:15347-15592;PQ340485.1:15333-15578;PQ340486.1:15348-15593;PQ340487.1:15373-15618;PQ340489.1:15348-15593;PQ340490.1:15299-15544;PQ340491.1:15357-15602;PQ340493.1:15333-15578;PQ340495.1:15363-15608;PQ340496.1:15396-15641;PQ368489.1:15319-15564;PQ451835.1:15407-15652;PQ451836.1:15339-15584;PQ451838.1:15390-15635;PQ451841.1:15350-15595;PQ451842.1:15302-15547 |
| OG0000196 | AY603973.1:180611-180775;AY741551.1:180618-180782;AY753185.1:180874-181038;DQ011154.1:178654-178818;DQ011155.1:178575-178739;EPI_ISL_13331598:178781-178945;EPI_ISL_13363142:178819-178983;EPI_ISL_13374487:178876-179040;EPI_ISL_13502582:178868-179032;EPI_ISL_13508471:178865-179029;EPI_ISL_13607904:178771-178935;EPI_ISL_13624509:178701-178865;EPI_ISL_13889442:178960-179124;EPI_ISL_13953610:178695-178859;EPI_ISL_14181948:178928-179092;EPI_ISL_14838587:178477-178641;EPI_ISL_15263355:178666-178830;EPI_ISL_15269699:178733-178897;EPI_ISL_15293002:179004-179168;EPI_ISL_15641561:178227-178391;EPI_ISL_15831211:178839-179003;EPI_ISL_15992098:178845-179009;EPI_ISL_16233784:178785-178949;EPI_ISL_16946400:178763-178927;EPI_ISL_17048206:178471-178635;EPI_ISL_17703567:178786-178950;EPI_ISL_17718458:178745-178909;EPI_ISL_18213374:178773-178937;EPI_ISL_18557816:178792-178956;EPI_ISL_18642365:178779-178943;EPI_ISL_18659846:178734-178898;EPI_ISL_18744049:178774-178938;EPI_ISL_19022858:178736-178900;EPI_ISL_19028778:178688-178852;EPI_ISL_19151800:177699-177863;EPI_ISL_19159116:178757-178921;EPI_ISL_19350788:177235-177399;EPI_ISL_19359677:178772-178936;EPI_ISL_19446306:178759-178923;EPI_ISL_19467692:178724-178888;JX878411.1:179498-179662;JX878425.1:178947-179111;JX878426.1:178423-178587;KC257460.1:178311-178475;KJ642612.1:176617-176781;KJ642613.1:178219-178383;KJ642616.1:180316-180480;KJ642618.1:176144-176308;KP849469.1:178841-179005;KP849471.1:178645-178809;LC831698.1:178755-178919;MN648051.1:178994-179158;MN702445.1:172015-172179;MN702446.1:178185-178349;MN702447.1:171548-171712;MN702449.1:172053-172217;MN702452.1:178239-178403;MT903338.1:178967-179131;MT903340.1:178784-178948;MT903341.1:170347-170511;MT903342.1:178887-179051;MT903347.1:180182-180346;ON568298.1:178929-179093;ON622713.1:179001-179165;ON622722.2:178789-178953;ON631963.1:178788-178952;ON649879.1:178594-178758;ON676707.1:178859-179023;ON755039.1:178410-178574;ON808413.1:178757-178921;ON880420.1:178991-179155;ON911481.2:178781-178945;ON918656.1:178798-178962;ON959133.1:178889-179053;ON959136.1:178884-179048;ON983168.1:177464-177628;OP019276.1:178771-178935;OP150924.1:178905-179069;OP160532.1:178720-178884;OP235282.1:178771-178935;OP289782.1:178775-178939;OP331335.1:178785-178949;OP382479.1:178766-178930;OP422341.1:178781-178945;OP434519.1:178753-178917;OP535324.1:178811-178975;OP535333.1:178893-179057;OP536770.1:178774-178938;OP597769.1:178854-179018;OP604566.1:178780-178944;OP612682.1:178681-178845;OP612684.1:178732-178896;OP612686.1:178734-178898;OP696841.1:176066-176230;OP879722.1:178033-178197;OQ419007.1:177991-178155;OQ419012.1:177965-178129;OQ419031.1:177969-178133;OQ419047.1:177971-178135;OQ451898.1:177877-178041;OQ451900.1:178791-178955;OQ581853.1:178789-178953;OQ581855.1:178898-179062;OQ721951.1:178777-178941;OQ721954.1:178810-178974;OR035652.1:178707-178871;OR035664.1:178711-178875;OR035702.1:178710-178874;OR146272.1:178727-178891;OR146330.1:178860-179024;OR264364.1:178782-178946;OR427332.1:178782-178946;OR459777.1:178750-178914;OR459797.1:178780-178944;OR943698.1:178242-178406;PP098589.2:178773-178937;PP496048.1:178789-178953;PP601206.1:178432-178596;PP776553.1:178715-178879;PP852971.1:178762-178926;PP852983.1:178786-178950;PP853000.1:178725-178889;PP853005.1:178745-178909;PP853007.1:178782-178946;PP853013.1:178779-178943;PP853027.1:178820-178984;PP853045.1:178585-178749;PP853048.1:178780-178944;PP860026.1:178783-178947;PP860027.1:178764-178928;PP860028.1:178719-178883;PP860031.1:178760-178924;PP860033.1:178757-178921;PQ153226.1:178754-178918;PQ178862.2:177097-177261;PQ221506.1:172290-172454;PQ221833.1:178407-178571;PQ340477.1:178745-178909;PQ340478.1:178735-178899;PQ340480.1:178803-178967;PQ340483.1:178795-178959;PQ340485.1:178780-178944;PQ340486.1:178794-178958;PQ340487.1:178820-178984;PQ340489.1:178797-178961;PQ340490.1:178745-178909;PQ340491.1:178808-178972;PQ340493.1:178780-178944;PQ340495.1:178814-178978;PQ340496.1:178849-179013;PQ368489.1:178787-178951;PQ451835.1:178858-179022;PQ451836.1:178798-178962;PQ451838.1:178850-179014;PQ451841.1:178815-178979;PQ451842.1:178751-178915 |
| OG0000197 | AY603973.1:183317-188959;AY741551.1:183054-188696;AY753185.1:183489-189131;DQ011154.1:181283-186922;DQ011155.1:181141-186780;EPI_ISL_13331598:181559-187201;EPI_ISL_13363142:181318-186960;EPI_ISL_13374487:181373-187015;EPI_ISL_13502582:181367-187009;EPI_ISL_13508471:181418-187060;EPI_ISL_13607904:181610-187252;EPI_ISL_13624509:181200-186842;EPI_ISL_13889442:181459-187101;EPI_ISL_13953610:181266-186908;EPI_ISL_14181948:181400-187042;EPI_ISL_14838587:181042-186681;EPI_ISL_15263355:181062-186704;EPI_ISL_15269699:181286-186928;EPI_ISL_15293002:181881-187523;EPI_ISL_15641561:180699-186341;EPI_ISL_15831211:181338-186980;EPI_ISL_15992098:181317-186959;EPI_ISL_16233784:181338-186980;EPI_ISL_16946400:181271-186913;EPI_ISL_17048206:180943-186585;EPI_ISL_17703567:181339-186981;EPI_ISL_17718458:181316-186958;EPI_ISL_18213374:181344-186986;EPI_ISL_18360394:180013-185655;EPI_ISL_18557816:181291-186933;EPI_ISL_18642365:181350-186992;EPI_ISL_18659846:181620-187262;EPI_ISL_18744049:181345-186987;EPI_ISL_19022858:181289-186931;EPI_ISL_19028778:181259-186901;EPI_ISL_19151800:180198-185840;EPI_ISL_19159116:181328-186970;EPI_ISL_19350788:179716-185355;EPI_ISL_19359677:181343-186985;EPI_ISL_19446306:181330-186972;EPI_ISL_19467692:181306-186945;JX878411.1:182126-187765;JX878425.1:181576-187215;JX878426.1:180989-186628;KC257460.1:180930-186536;KJ642612.1:179092-184731;KJ642613.1:180847-186486;KJ642616.1:183049-188691;KJ642618.1:178611-184250;KP849469.1:181433-187072;KP849471.1:181250-186889;LC831698.1:181326-186968;MN648051.1:181533-187175;MN702445.1:174489-180128;MN702446.1:180845-186484;MN702447.1:174051-179690;MN702449.1:174536-180175;MN702452.1:180732-186371;MT903338.1:181538-187180;MT903340.1:181355-186997;MT903341.1:172900-176838;MT903342.1:181440-187082;MT903347.1:182815-188457;ON568298.1:181401-187043;ON622713.1:181932-187574;ON622722.2:181263-186905;ON631963.1:181566-187208;ON649879.1:181066-186708;ON676707.1:181358-187000;ON755039.1:180949-186591;ON808413.1:181274-186916;ON880420.1:181886-187528;ON911481.2:181541-187183;ON918656.1:181351-186993;ON959133.1:181793-187435;ON959136.1:181815-187457;ON983168.1:180359-186001;OP019276.1:181342-186984;OP150924.1:181786-187428;OP160532.1:181192-186834;OP235282.1:181342-186984;OP289782.1:181346-186988;OP331335.1:181338-186980;OP382479.1:181319-186961;OP422341.1:181334-186976;OP434519.1:181324-185562;OP535324.1:181382-187024;OP535333.1:181437-187079;OP536770.1:181345-186987;OP597769.1:181327-186969;OP604566.1:181351-186993;OP612682.1:181252-186894;OP612684.1:181492-187134;OP612686.1:181305-186947;OP696841.1:178565-184207;OP879722.1:180505-186147;OQ419007.1:180562-186204;OQ419012.1:180536-186178;OQ419031.1:180540-186182;OQ419047.1:180542-186184;OQ451898.1:180376-186018;OQ451900.1:181290-186932;OQ581853.1:181288-186930;OQ581855.1:181397-187039;OQ721951.1:181366-187008;OQ721954.1:181298-186940;OR035652.1:181678-187320;OR035664.1:181624-187266;OR035702.1:181641-187283;OR146272.1:181280-186922;OR146330.1:181359-187001;OR264364.1:181335-186977;OR427332.1:181335-186977;OR459777.1:181321-186963;OR459797.1:181333-186975;OR943698.1:180954-186593;PP098589.2:181344-186986;PP496048.1:181262-186904;PP601206.1:180997-186636;PP776553.1:181214-186856;PP852971.1:181333-186975;PP852983.1:181339-186981;PP853000.1:181224-186866;PP853005.1:181298-186940;PP853007.1:181281-186923;PP853013.1:181278-186920;PP853027.1:181319-186961;PP853045.1:181084-186726;PP853048.1:181279-186921;PP860026.1:181282-186924;PP860027.1:181262-186904;PP860028.1:181218-186860;PP860031.1:181259-186901;PP860033.1:181255-186897;PQ153226.1:181325-186967;PQ178862.2:179481-185120;PQ221506.1:174855-180491;PQ221833.1:180974-186613;PQ340477.1:181415-187057;PQ340478.1:181324-186966;PQ340480.1:181446-187088;PQ340483.1:181402-187044;PQ340485.1:181441-187083;PQ340486.1:181500-187142;PQ340487.1:181544-187186;PQ340489.1:181476-187118;PQ340490.1:181334-186976;PQ340491.1:181577-187219;PQ340493.1:181396-187038;PQ340495.1:181457-187099;PQ340496.1:181555-187197;PQ368489.1:181358-187000;PQ451835.1:181519-187161;PQ451836.1:181297-186939;PQ451838.1:181502-187144;PQ451841.1:181413-187055 |
| OG0000198 | AY603973.1:93306-93139;AY741551.1:93310-93143;AY753185.1:93549-93382;DQ011154.1:93780-93607;DQ011155.1:93761-93588;EPI_ISL_13331598:91380-91207;EPI_ISL_13363142:91373-91200;EPI_ISL_13374487:91388-91215;EPI_ISL_13502582:91373-91200;EPI_ISL_13508471:91465-91292;EPI_ISL_13607904:91364-91191;EPI_ISL_13624509:91308-91135;EPI_ISL_13889442:91483-91310;EPI_ISL_13953610:91281-91108;EPI_ISL_14181948:91509-91336;EPI_ISL_14838587:93634-93461;EPI_ISL_15263355:91364-91191;EPI_ISL_15269699:91333-91160;EPI_ISL_15293002:91606-91433;EPI_ISL_15641561:91322-91149;EPI_ISL_15831211:91371-91198;EPI_ISL_15992098:91386-91213;EPI_ISL_16233784:91382-91209;EPI_ISL_16946400:91371-91198;EPI_ISL_17048206:91021-90848;EPI_ISL_17703567:91386-91213;EPI_ISL_17718458:91364-91191;EPI_ISL_18213374:91361-91188;EPI_ISL_18360394:91022-90849;EPI_ISL_18557816:91362-91189;EPI_ISL_18642365:91364-91191;EPI_ISL_18659846:91334-91161;EPI_ISL_18744049:91366-91193;EPI_ISL_19022858:91361-91188;EPI_ISL_19028778:91278-91105;EPI_ISL_19151800:91362-91189;EPI_ISL_19159116:91364-91191;EPI_ISL_19350788:92506-92333;EPI_ISL_19359677:91361-91188;EPI_ISL_19446306:91356-91183;EPI_ISL_19467692:93917-93744;JX878411.1:94625-94452;JX878425.1:94142-93969;JX878426.1:93591-93418;KC257460.1:93559-93386;KJ642612.1:91877-91704;KJ642613.1:93436-93263;KJ642616.1:93000-92833;KJ642618.1:91107-90934;KP849469.1:93950-93777;KP849471.1:93876-93703;LC831698.1:91362-91189;MN702445.1:87293-87120;MN702446.1:93438-93265;MN702447.1:86806-86633;MN702449.1:87304-87131;MN702452.1:93479-93306;MT903338.1:91527-91354;MT903340.1:91364-91191;MT903341.1:82897-82724;MT903342.1:91379-91206;MT903347.1:93317-93150;ON568298.1:91510-91337;ON622713.1:91596-91423;ON622722.2:91367-91194;ON631963.1:91387-91214;ON649879.1:91197-91024;ON676707.1:91417-91244;ON755039.1:91027-90854;ON808413.1:91367-91194;ON880420.1:91594-91421;ON911481.2:91384-91211;ON918656.1:91399-91226;ON959133.1:91488-91315;ON959136.1:91489-91316;ON983168.1:90062-89889;OP019276.1:91361-91188;OP150924.1:91502-91329;OP160532.1:91327-91154;OP235282.1:91361-91188;OP289782.1:91364-91191;OP331335.1:91381-91208;OP382479.1:91368-91195;OP422341.1:91381-91208;OP434519.1:91360-91187;OP535324.1:91362-91189;OP535333.1:91471-91298;OP536770.1:91363-91190;OP597769.1:91385-91212;OP604566.1:91364-91191;OP612682.1:91289-91116;OP612684.1:91352-91179;OP612686.1:91353-91180;OP696841.1:88638-88465;OP879722.1:90587-90414;OQ419007.1:90597-90424;OQ419012.1:90564-90391;OQ419031.1:90568-90395;OQ419047.1:90570-90397;OQ451898.1:90449-90276;OQ451900.1:91362-91189;OQ581853.1:91388-91215;OQ581855.1:91469-91296;OQ721951.1:91402-91229;OQ721954.1:91359-91186;OR035652.1:91311-91138;OR035664.1:91311-91138;OR035702.1:91311-91138;OR146272.1:91327-91154;OR146330.1:91372-91199;OR264364.1:91382-91209;OR427332.1:91382-91209;OR459777.1:91366-91193;OR459797.1:91381-91208;OR943698.1:93692-93519;PP098589.2:91363-91190;PP496048.1:91367-91194;PP601206.1:93623-93450;PP776553.1:91288-91115;PP852971.1:91361-91188;PP852983.1:91367-91194;PP853000.1:91328-91155;PP853005.1:91336-91163;PP853007.1:91369-91196;PP853013.1:91342-91169;PP853027.1:91373-91200;PP853045.1:91354-91181;PP853048.1:91373-91200;PP860026.1:91371-91198;PP860027.1:91396-91223;PP860028.1:91336-91163;PP860031.1:91390-91217;PP860033.1:91395-91222;PQ153226.1:91361-91188;PQ178862.2:92483-92310;PQ221506.1:87459-87286;PQ221833.1:93625-93452;PQ340477.1:91344-91171;PQ340478.1:91329-91156;PQ340480.1:91408-91235;PQ340483.1:91392-91219;PQ340485.1:91378-91205;PQ340486.1:91393-91220;PQ340487.1:91418-91245;PQ340489.1:91393-91220;PQ340490.1:91344-91171;PQ340491.1:91399-91226;PQ340493.1:91378-91205;PQ340495.1:91408-91235;PQ340496.1:91441-91268;PQ368489.1:91364-91191;PQ451835.1:91449-91276;PQ451836.1:91381-91208;PQ451838.1:91432-91259;PQ451841.1:91392-91219;PQ451842.1:91344-91171 |
| OG0000199 | AY603973.1:175964-175797;AY741551.1:175973-175806;AY753185.1:176227-176060;DQ011154.1:174003-173836;DQ011155.1:173925-173758;EPI_ISL_13331598:174127-173960;EPI_ISL_13363142:174165-173998;EPI_ISL_13374487:174222-174055;EPI_ISL_13502582:174214-174047;EPI_ISL_13508471:174211-174044;EPI_ISL_13607904:174117-173950;EPI_ISL_13624509:174047-173880;EPI_ISL_13889442:174306-174139;EPI_ISL_13953610:174041-173874;EPI_ISL_14181948:174274-174107;EPI_ISL_14838587:173831-173664;EPI_ISL_15263355:174012-173845;EPI_ISL_15269699:174079-173912;EPI_ISL_15293002:174350-174183;EPI_ISL_15641561:173573-173406;EPI_ISL_15831211:174191-174024;EPI_ISL_15992098:174191-174024;EPI_ISL_16233784:174131-173964;EPI_ISL_16946400:174115-173948;EPI_ISL_17048206:173817-173650;EPI_ISL_17703567:174132-173965;EPI_ISL_17718458:174097-173930;EPI_ISL_18213374:174119-173952;EPI_ISL_18360394:173395-173228;EPI_ISL_18557816:174138-173971;EPI_ISL_18642365:174125-173958;EPI_ISL_18659846:174080-173913;EPI_ISL_18744049:174126-173959;EPI_ISL_19022858:174082-173915;EPI_ISL_19028778:174034-173867;EPI_ISL_19151800:173045-172878;EPI_ISL_19159116:174103-173936;EPI_ISL_19350788:172472-172305;EPI_ISL_19359677:174118-173951;EPI_ISL_19446306:174105-173938;EPI_ISL_19467692:174072-173905;JX878411.1:174844-174677;JX878425.1:174296-174129;JX878426.1:173767-173600;KC257460.1:173661-173494;KJ642612.1:171957-171790;KJ642613.1:173564-173397;KJ642616.1:175671-175504;KJ642618.1:171487-171320;KP849469.1:174191-174024;KP849471.1:173994-173827;LC831698.1:174101-173934;MN648051.1:174340-174173;MN702445.1:167371-167204;MN702446.1:173541-173374;MN702449.1:167409-167242;MN702452.1:173593-173426;MT903338.1:174313-174146;MT903340.1:174130-173963;MT903341.1:165693-165526;MT903342.1:174233-174066;MT903347.1:175529-175362;ON568298.1:174275-174108;ON622713.1:174347-174180;ON622722.2:174135-173968;ON631963.1:174134-173967;ON649879.1:173940-173773;ON676707.1:174205-174038;ON755039.1:173756-173589;ON808413.1:174103-173936;ON880420.1:174337-174170;ON911481.2:174127-173960;ON918656.1:174144-173977;ON959133.1:174235-174068;ON959136.1:174230-174063;ON983168.1:172810-172643;OP019276.1:174117-173950;OP150924.1:174251-174084;OP160532.1:174066-173899;OP235282.1:174117-173950;OP289782.1:174121-173954;OP331335.1:174131-173964;OP382479.1:174112-173945;OP422341.1:174127-173960;OP434519.1:174099-173932;OP535324.1:174157-173990;OP535333.1:174239-174072;OP536770.1:174120-173953;OP597769.1:174200-174033;OP604566.1:174120-173953;OP612682.1:174027-173860;OP612684.1:174078-173911;OP612686.1:174080-173913;OP696841.1:171412-171245;OP879722.1:173379-173212;OQ419007.1:173337-173170;OQ419012.1:173311-173144;OQ419031.1:173315-173148;OQ419047.1:173317-173150;OQ451898.1:173223-173056;OQ451900.1:174137-173970;OQ581853.1:174135-173968;OQ581855.1:174244-174077;OQ721951.1:174123-173956;OQ721954.1:174156-173989;OR035652.1:174053-173886;OR035664.1:174057-173890;OR035702.1:174056-173889;OR146272.1:174073-173906;OR146330.1:174206-174039;OR264364.1:174128-173961;OR427332.1:174128-173961;OR459777.1:174102-173935;OR459797.1:174126-173959;OR943698.1:173582-173415;PP098589.2:174119-173952;PP496048.1:174135-173968;PP601206.1:173779-173612;PP776553.1:174061-173894;PP852971.1:174108-173941;PP852983.1:174132-173965;PP853000.1:174071-173904;PP853005.1:174091-173924;PP853007.1:174128-173961;PP853013.1:174125-173958;PP853027.1:174166-173999;PP853045.1:174106-173939;PP853048.1:174126-173959;PP860026.1:174133-173966;PP860027.1:174116-173949;PP860028.1:174070-173903;PP860031.1:174112-173945;PP860033.1:174109-173942;PQ153226.1:174100-173933;PQ178862.2:172448-172281;PQ221506.1:167631-167464;PQ221833.1:173759-173592;PQ340477.1:174091-173924;PQ340478.1:174081-173914;PQ340480.1:174149-173982;PQ340483.1:174141-173974;PQ340485.1:174126-173959;PQ340486.1:174140-173973;PQ340487.1:174160-173993;PQ340489.1:174143-173976;PQ340490.1:174091-173924;PQ340491.1:174154-173987;PQ340493.1:174126-173959;PQ340495.1:174160-173993;PQ340496.1:174195-174028;PQ368489.1:174133-173966;PQ451835.1:174204-174037;PQ451836.1:174144-173977;PQ451838.1:174196-174029;PQ451841.1:174161-173994;PQ451842.1:174097-173930 |
| OG0000200 | AY603973.1:152924-152670;AY741551.1:152998-152744;AY753185.1:153179-152925;DQ011154.1:153009-152836;DQ011155.1:152951-152778;EPI_ISL_13331598:151043-150789;EPI_ISL_13363142:151031-150777;EPI_ISL_13374487:151064-150810;EPI_ISL_13502582:151059-150805;EPI_ISL_13508471:151125-150871;EPI_ISL_13607904:151030-150776;EPI_ISL_13624509:150965-150711;EPI_ISL_13889442:151158-150904;EPI_ISL_13953610:150958-150704;EPI_ISL_14181948:151188-150934;EPI_ISL_15263355:151024-150770;EPI_ISL_15269699:150993-150739;EPI_ISL_15293002:151262-151008;EPI_ISL_15641561:150979-150725;EPI_ISL_15831211:151033-150779;EPI_ISL_15992098:151073-150819;EPI_ISL_16233784:151045-150791;EPI_ISL_16946400:151035-150781;EPI_ISL_17048206:150699-150445;EPI_ISL_17703567:151046-150792;EPI_ISL_17718458:151026-150772;EPI_ISL_18213374:151037-150783;EPI_ISL_18360394:150344-150090;EPI_ISL_18557816:151040-150786;EPI_ISL_18642365:151035-150781;EPI_ISL_18659846:150994-150740;EPI_ISL_18744049:151043-150789;EPI_ISL_19022858:151000-150746;EPI_ISL_19028778:150952-150698;EPI_ISL_19151800:151036-150782;EPI_ISL_19159116:151021-150767;EPI_ISL_19350788:151608-151435;EPI_ISL_19359677:151036-150782;EPI_ISL_19446306:151023-150769;EPI_ISL_19467692:153149-152976;JX878411.1:153850-153677;JX878425.1:153338-153165;JX878426.1:152878-152705;KC257460.1:152734-152561;KJ642612.1:151068-150895;KJ642613.1:152617-152444;KJ642616.1:152646-152392;KJ642618.1:150533-150360;KP849469.1:153166-152993;KP849471.1:153099-152926;LC831698.1:151019-150765;MN648051.1:151262-151008;MN702445.1:146560-146312;MN702446.1:152649-152476;MN702447.1:146012-145839;MN702449.1:146523-146350;MN702452.1:152700-152527;MT903338.1:151195-150941;MT903340.1:151048-150794;MT903341.1:142589-142335;MT903342.1:151085-150831;MT903347.1:152991-152737;ON568298.1:151189-150935;ON622713.1:151253-150999;ON622722.2:151041-150787;ON631963.1:151050-150796;ON649879.1:150856-150602;ON676707.1:151087-150836;ON755039.1:150678-150424;ON808413.1:151015-150761;ON880420.1:151251-150997;ON911481.2:151036-150782;ON918656.1:151058-150804;ON959133.1:151147-150893;ON959136.1:151148-150894;ON983168.1:149724-149470;OP019276.1:151035-150781;OP150924.1:151161-150907;OP160532.1:150984-150730;OP235282.1:151035-150781;OP289782.1:151039-150785;OP331335.1:151044-150790;OP382479.1:151028-150774;OP422341.1:151041-150787;OP434519.1:151017-150763;OP535324.1:151048-150794;OP535333.1:151155-150901;OP536770.1:151038-150784;OP597769.1:151072-150818;OP604566.1:151038-150784;OP612682.1:150973-150719;OP612684.1:151024-150770;OP612686.1:151026-150772;OP696841.1:148314-148060;OP879722.1:150261-150007;OQ419007.1:150255-150001;OQ419012.1:150229-149975;OQ419031.1:150233-149979;OQ419047.1:150235-149981;OQ451898.1:150125-149871;OQ451900.1:151039-150785;OQ581853.1:151047-150793;OQ581855.1:151146-150892;OQ721951.1:151065-150811;OQ721954.1:151038-150784;OR035652.1:150967-150713;OR035664.1:150971-150717;OR035702.1:150968-150714;OR146272.1:150987-150733;OR146330.1:151048-150794;OR264364.1:151042-150788;OR427332.1:151042-150788;OR459777.1:151031-150777;OR459797.1:151040-150786;OR943698.1:152854-152681;PP098589.2:151037-150783;PP496048.1:151041-150787;PP601206.1:152909-152736;PP776553.1:150961-150707;PP852971.1:151029-150775;PP852983.1:151046-150795;PP853000.1:150997-150746;PP853005.1:151005-150754;PP853007.1:151048-150797;PP853013.1:151020-150766;PP853027.1:151062-150811;PP853045.1:151013-150759;PP853048.1:151038-150787;PP860026.1:151033-150779;PP860027.1:151070-150816;PP860028.1:150996-150742;PP860031.1:151067-150813;PP860033.1:151053-150799;PQ153226.1:151018-150764;PQ178862.2:151587-151414;PQ221506.1:146698-146525;PQ221833.1:152885-152712;PQ340477.1:151005-150751;PQ340478.1:150987-150733;PQ340480.1:151065-150811;PQ340483.1:151051-150797;PQ340485.1:151034-150780;PQ340486.1:151052-150798;PQ340487.1:151076-150822;PQ340489.1:151051-150797;PQ340490.1:151003-150749;PQ340491.1:151058-150804;PQ340493.1:151042-150788;PQ340495.1:151068-150814;PQ340496.1:151103-150849;PQ368489.1:151051-150797;PQ451835.1:151108-150854;PQ451836.1:151046-150792;PQ451838.1:151096-150842;PQ451841.1:151059-150805;PQ451842.1:151005-150751 |
| OG0000201 | AY603973.1:12680-12501;AY741551.1:12515-12336;AY753185.1:12926-12747;DQ011154.1:11170-10991;DQ011155.1:11150-10971;EPI_ISL_13331598:10997-10818;EPI_ISL_13363142:10990-10811;EPI_ISL_13374487:11005-10826;EPI_ISL_13502582:10990-10811;EPI_ISL_13508471:11082-10903;EPI_ISL_13607904:10981-10802;EPI_ISL_13624509:10925-10746;EPI_ISL_13889442:11100-10921;EPI_ISL_13953610:10981-10802;EPI_ISL_14181948:11126-10947;EPI_ISL_14838587:11024-10845;EPI_ISL_15263355:10978-10799;EPI_ISL_15269699:10950-10771;EPI_ISL_15293002:11223-11044;EPI_ISL_15641561:10939-10760;EPI_ISL_15831211:10988-10809;EPI_ISL_15992098:11003-10824;EPI_ISL_16233784:10999-10820;EPI_ISL_16946400:10988-10809;EPI_ISL_17048206:10638-10459;EPI_ISL_17703567:11003-10824;EPI_ISL_17718458:10981-10802;EPI_ISL_18213374:10978-10799;EPI_ISL_18360394:10639-10460;EPI_ISL_18557816:10979-10800;EPI_ISL_18642365:10981-10802;EPI_ISL_18659846:10951-10772;EPI_ISL_18744049:10983-10804;EPI_ISL_19022858:10978-10799;EPI_ISL_19028778:10895-10716;EPI_ISL_19151800:10979-10800;EPI_ISL_19159116:10981-10802;EPI_ISL_19350788:11036-10857;EPI_ISL_19359677:10978-10799;EPI_ISL_19446306:10973-10794;EPI_ISL_19467692:11302-11123;JX878411.1:12015-11836;JX878425.1:11530-11351;JX878426.1:10972-10793;KC257460.1:10951-10772;KJ642612.1:9257-9078;KJ642613.1:10833-10654;KJ642616.1:12497-12318;KJ642618.1:8429-8250;KP849469.1:11340-11161;KP849471.1:11260-11081;LC831698.1:10979-10800;MN648051.1:11200-11021;MN702445.1:4680-4501;MN702446.1:10826-10647;MN702447.1:4192-4013;MN702449.1:4688-4509;MN702452.1:10869-10690;MT903338.1:11144-10965;MT903340.1:10981-10802;MT903342.1:10996-10817;MT903347.1:12851-12672;ON568298.1:11127-10948;ON622713.1:11212-11033;ON622722.2:10984-10805;ON631963.1:11004-10825;ON649879.1:10814-10635;ON676707.1:11034-10855;ON755039.1:10644-10465;ON808413.1:10984-10805;ON880420.1:11211-11032;ON911481.2:11001-10822;ON918656.1:11016-10837;ON959133.1:11105-10926;ON959136.1:11106-10927;ON983168.1:9680-9501;OP019276.1:10978-10799;OP150924.1:11119-10940;OP160532.1:10944-10765;OP235282.1:10978-10799;OP289782.1:10981-10802;OP331335.1:10998-10819;OP382479.1:10985-10806;OP422341.1:10998-10819;OP434519.1:10977-10798;OP535324.1:10979-10800;OP535333.1:11088-10909;OP536770.1:10980-10801;OP597769.1:11002-10823;OP604566.1:10981-10802;OP612682.1:10907-10728;OP612684.1:10971-10792;OP612686.1:10971-10792;OP696841.1:8282-8103;OP879722.1:10204-10025;OQ419007.1:10214-10035;OQ419012.1:10206-10027;OQ419031.1:10211-10032;OQ419047.1:10214-10035;OQ451898.1:10979-10800;OQ451900.1:10979-10800;OQ581853.1:11005-10826;OQ581855.1:11086-10907;OQ721951.1:11019-10840;OQ721954.1:10976-10797;OR035652.1:10928-10749;OR035664.1:10928-10749;OR035702.1:10928-10749;OR146272.1:10944-10765;OR146330.1:10989-10810;OR264364.1:10999-10820;OR427332.1:10999-10820;OR459777.1:10983-10804;OR459797.1:10998-10819;OR943698.1:11036-10857;PP098589.2:10980-10801;PP496048.1:10984-10805;PP601206.1:11022-10843;PP776553.1:10929-10750;PP852971.1:10978-10799;PP852983.1:10983-10804;PP853000.1:10945-10766;PP853005.1:10953-10774;PP853007.1:10985-10806;PP853013.1:10959-10780;PP853027.1:10990-10811;PP853045.1:10989-10810;PP853048.1:10990-10811;PP860026.1:10985-10806;PP860027.1:10984-10805;PP860028.1:10952-10773;PP860031.1:10991-10812;PP860033.1:10983-10804;PQ153226.1:10978-10799;PQ178862.2:11013-10834;PQ221506.1:4843-4664;PQ221833.1:11014-10835;PQ340477.1:10961-10782;PQ340478.1:10946-10767;PQ340480.1:11025-10846;PQ340483.1:11009-10830;PQ340485.1:10995-10816;PQ340486.1:11010-10831;PQ340487.1:11035-10856;PQ340489.1:11010-10831;PQ340490.1:10961-10782;PQ340491.1:11019-10840;PQ340493.1:10995-10816;PQ340495.1:11025-10846;PQ340496.1:11058-10879;PQ368489.1:10981-10802;PQ451835.1:11069-10890;PQ451836.1:11001-10822;PQ451838.1:11052-10873;PQ451841.1:11012-10833;PQ451842.1:10964-10785 |
| OG0000202 | AY603973.1:12556-12176;AY741551.1:12391-12011;AY753185.1:12802-12422;DQ011154.1:11046-10666;DQ011155.1:11026-10646;EPI_ISL_13331598:10873-10493;EPI_ISL_13363142:10866-10486;EPI_ISL_13374487:10881-10501;EPI_ISL_13502582:10866-10486;EPI_ISL_13508471:10958-10578;EPI_ISL_13607904:10857-10477;EPI_ISL_13624509:10801-10421;EPI_ISL_13889442:10976-10596;EPI_ISL_13953610:10857-10477;EPI_ISL_14181948:11002-10622;EPI_ISL_14838587:10900-10520;EPI_ISL_15263355:10854-10474;EPI_ISL_15269699:10826-10446;EPI_ISL_15293002:11099-10719;EPI_ISL_15641561:10815-10435;EPI_ISL_15831211:10864-10484;EPI_ISL_15992098:10879-10499;EPI_ISL_16233784:10875-10495;EPI_ISL_16946400:10864-10484;EPI_ISL_17048206:10514-10134;EPI_ISL_17703567:10879-10499;EPI_ISL_17718458:10857-10477;EPI_ISL_18213374:10854-10474;EPI_ISL_18360394:10515-10135;EPI_ISL_18557816:10855-10475;EPI_ISL_18642365:10857-10477;EPI_ISL_18659846:10827-10447;EPI_ISL_18744049:10859-10479;EPI_ISL_19022858:10854-10474;EPI_ISL_19028778:10771-10391;EPI_ISL_19151800:10855-10475;EPI_ISL_19159116:10857-10477;EPI_ISL_19350788:10912-10532;EPI_ISL_19359677:10854-10474;EPI_ISL_19446306:10849-10469;EPI_ISL_19467692:11178-10798;JX878411.1:11891-11511;JX878425.1:11406-11026;JX878426.1:10848-10468;KC257460.1:10827-10447;KJ642612.1:9133-8753;KJ642613.1:10709-10329;KJ642616.1:12373-11993;KJ642618.1:8305-7925;KP849469.1:11216-10836;KP849471.1:11136-10756;LC831698.1:10855-10475;MN648051.1:11076-10696;MN702445.1:4556-4176;MN702446.1:10702-10322;MN702447.1:4068-3688;MN702449.1:4564-4184;MN702452.1:10745-10365;MT903338.1:11020-10640;MT903340.1:10857-10477;MT903342.1:10872-10492;MT903347.1:12727-12347;ON568298.1:11003-10623;ON622713.1:11088-10708;ON622722.2:10860-10480;ON631963.1:10880-10500;ON649879.1:10690-10310;ON676707.1:10910-10530;ON755039.1:10520-10140;ON808413.1:10860-10480;ON880420.1:11087-10707;ON911481.2:10877-10497;ON918656.1:10892-10512;ON959133.1:10981-10601;ON959136.1:10982-10602;ON983168.1:9556-9176;OP019276.1:10854-10474;OP150924.1:10995-10615;OP160532.1:10820-10440;OP235282.1:10854-10474;OP289782.1:10857-10477;OP331335.1:10874-10494;OP382479.1:10861-10481;OP422341.1:10874-10494;OP434519.1:10853-10473;OP535324.1:10855-10475;OP535333.1:10964-10584;OP536770.1:10856-10476;OP597769.1:10878-10498;OP604566.1:10857-10477;OP612682.1:10783-10403;OP612684.1:10847-10467;OP612686.1:10847-10467;OP696841.1:8158-7778;OP879722.1:10080-9700;OQ419007.1:10090-9710;OQ419012.1:10082-9702;OQ419031.1:10087-9707;OQ419047.1:10090-9710;OQ451898.1:10855-10475;OQ451900.1:10855-10475;OQ581853.1:10881-10501;OQ581855.1:10962-10582;OQ721951.1:10895-10515;OQ721954.1:10852-10472;OR035652.1:10804-10424;OR035664.1:10804-10424;OR035702.1:10804-10424;OR146272.1:10820-10440;OR146330.1:10865-10485;OR264364.1:10875-10495;OR427332.1:10875-10495;OR459777.1:10859-10479;OR459797.1:10874-10494;OR943698.1:10912-10532;PP098589.2:10856-10476;PP496048.1:10860-10480;PP601206.1:10898-10518;PP776553.1:10805-10425;PP852971.1:10854-10474;PP852983.1:10859-10479;PP853000.1:10821-10441;PP853005.1:10829-10449;PP853007.1:10861-10481;PP853013.1:10835-10455;PP853027.1:10866-10486;PP853045.1:10865-10485;PP853048.1:10866-10486;PP860026.1:10861-10481;PP860027.1:10860-10480;PP860028.1:10828-10448;PP860031.1:10867-10487;PP860033.1:10859-10479;PQ153226.1:10854-10474;PQ178862.2:10889-10509;PQ221506.1:4719-4339;PQ221833.1:10890-10510;PQ340477.1:10837-10457;PQ340478.1:10822-10442;PQ340480.1:10901-10521;PQ340483.1:10885-10505;PQ340485.1:10871-10491;PQ340486.1:10886-10506;PQ340487.1:10911-10531;PQ340489.1:10886-10506;PQ340490.1:10837-10457;PQ340491.1:10895-10515;PQ340493.1:10871-10491;PQ340495.1:10901-10521;PQ340496.1:10934-10554;PQ368489.1:10857-10477;PQ451835.1:10945-10565;PQ451836.1:10877-10497;PQ451838.1:10928-10548;PQ451841.1:10888-10508;PQ451842.1:10840-10460 |
| OG0000203 | AY603973.1:10317-10487;AY741551.1:10142-10312;AY753185.1:10528-10698;DQ011154.1:8722-8892;DQ011155.1:8627-8797;EPI_ISL_13331598:8663-8833;EPI_ISL_13363142:8656-8826;EPI_ISL_13374487:8671-8841;EPI_ISL_13502582:8656-8826;EPI_ISL_13508471:8748-8918;EPI_ISL_13607904:8647-8817;EPI_ISL_13624509:8591-8761;EPI_ISL_13889442:8766-8936;EPI_ISL_13953610:8647-8817;EPI_ISL_14181948:8792-8962;EPI_ISL_14838587:8626-8796;EPI_ISL_15263355:8644-8814;EPI_ISL_15269699:8616-8786;EPI_ISL_15293002:8889-9059;EPI_ISL_15641561:8605-8775;EPI_ISL_15831211:8654-8824;EPI_ISL_15992098:8669-8839;EPI_ISL_16233784:8665-8835;EPI_ISL_16946400:8654-8824;EPI_ISL_17048206:8304-8474;EPI_ISL_17703567:8669-8839;EPI_ISL_17718458:8647-8817;EPI_ISL_18213374:8644-8814;EPI_ISL_18360394:8305-8475;EPI_ISL_18557816:8645-8815;EPI_ISL_18642365:8647-8817;EPI_ISL_18659846:8617-8787;EPI_ISL_18744049:8649-8819;EPI_ISL_19022858:8644-8814;EPI_ISL_19028778:8561-8731;EPI_ISL_19151800:8645-8815;EPI_ISL_19159116:8647-8817;EPI_ISL_19350788:8638-8808;EPI_ISL_19359677:8644-8814;EPI_ISL_19446306:8639-8809;EPI_ISL_19467692:8854-9024;JX878411.1:9567-9737;JX878425.1:9007-9177;JX878426.1:8574-8744;KC257460.1:8393-8563;KJ642612.1:6913-7083;KJ642613.1:8436-8606;KJ642616.1:10134-10304;KJ642618.1:5981-6151;KP849469.1:8829-8999;KP849471.1:8801-8989;LC831698.1:8645-8815;MN648051.1:8866-9036;MN702445.1:2292-2462;MN702446.1:8438-8608;MN702447.1:1799-1969;MN702449.1:2295-2465;MN702452.1:8476-8646;MT903338.1:8810-8980;MT903340.1:8647-8817;MT903342.1:8662-8832;MT903347.1:10403-10573;ON568298.1:8793-8963;ON622713.1:8878-9048;ON622722.2:8650-8820;ON631963.1:8670-8840;ON649879.1:8480-8650;ON676707.1:8700-8870;ON755039.1:8310-8480;ON808413.1:8650-8820;ON880420.1:8877-9047;ON911481.2:8667-8837;ON918656.1:8682-8852;ON959133.1:8771-8941;ON959136.1:8772-8942;ON983168.1:7346-7516;OP019276.1:8644-8814;OP150924.1:8785-8955;OP160532.1:8610-8780;OP235282.1:8644-8814;OP289782.1:8647-8817;OP331335.1:8664-8834;OP382479.1:8651-8821;OP422341.1:8664-8834;OP434519.1:8643-8813;OP535324.1:8645-8815;OP535333.1:8754-8924;OP536770.1:8646-8816;OP597769.1:8668-8838;OP604566.1:8647-8817;OP612682.1:8573-8743;OP612684.1:8637-8807;OP612686.1:8637-8807;OP879722.1:7870-8040;OQ419007.1:7880-8050;OQ419012.1:7872-8042;OQ419031.1:7877-8047;OQ419047.1:7880-8050;OQ451898.1:8645-8815;OQ451900.1:8645-8815;OQ581853.1:8671-8841;OQ581855.1:8752-8922;OQ721951.1:8685-8855;OQ721954.1:8642-8812;OR035652.1:8594-8764;OR035664.1:8594-8764;OR035702.1:8594-8764;OR146272.1:8610-8780;OR146330.1:8655-8825;OR264364.1:8665-8835;OR427332.1:8665-8835;OR459777.1:8649-8819;OR459797.1:8664-8834;OR943698.1:8603-8773;PP098589.2:8646-8816;PP496048.1:8650-8820;PP601206.1:8624-8794;PP776553.1:8595-8765;PP852971.1:8644-8814;PP852983.1:8649-8819;PP853000.1:8608-8907;PP853005.1:8619-8789;PP853007.1:8656-9021;PP853013.1:8625-8795;PP853027.1:8656-8826;PP853045.1:8660-9025;PP853048.1:8656-8826;PP860026.1:8650-8820;PP860027.1:8650-8820;PP860028.1:8618-8788;PP860031.1:8662-8832;PP860033.1:8649-8819;PQ153226.1:8644-8814;PQ178862.2:8655-8825;PQ221506.1:2445-2615;PQ221833.1:8616-8786;PQ340477.1:8627-8797;PQ340478.1:8612-8782;PQ340480.1:8691-8861;PQ340483.1:8675-8845;PQ340485.1:8661-8831;PQ340486.1:8676-8846;PQ340487.1:8701-8871;PQ340489.1:8676-8846;PQ340490.1:8627-8797;PQ340491.1:8685-8855;PQ340493.1:8661-8831;PQ340495.1:8691-8861;PQ340496.1:8724-8894;PQ368489.1:8647-8817;PQ451835.1:8735-8905;PQ451836.1:8667-8837;PQ451838.1:8718-8888;PQ451841.1:8678-8848;PQ451842.1:8630-8800 |
| OG0000204 | AY603973.1:22137-22556;AY741551.1:21999-22418;AY753185.1:22380-22799;DQ011154.1:22590-23009;DQ011155.1:22570-22989;EPI_ISL_13331598:20459-20878;EPI_ISL_13363142:20452-20871;EPI_ISL_13374487:20467-20886;EPI_ISL_13502582:20452-20871;EPI_ISL_13508471:20544-20963;EPI_ISL_13607904:20443-20862;EPI_ISL_13624509:20387-20806;EPI_ISL_13889442:20562-20981;EPI_ISL_13953610:20410-20829;EPI_ISL_14181948:20588-21007;EPI_ISL_14838587:22444-22863;EPI_ISL_15263355:20443-20862;EPI_ISL_15269699:20412-20831;EPI_ISL_15293002:20685-21104;EPI_ISL_15641561:20401-20820;EPI_ISL_15831211:20450-20869;EPI_ISL_15992098:20465-20884;EPI_ISL_16233784:20461-20880;EPI_ISL_16946400:20450-20869;EPI_ISL_17048206:20100-20519;EPI_ISL_17703567:20465-20884;EPI_ISL_17718458:20443-20862;EPI_ISL_18213374:20440-20859;EPI_ISL_18360394:20101-20520;EPI_ISL_18557816:20441-20860;EPI_ISL_18642365:20443-20862;EPI_ISL_18659846:20413-20832;EPI_ISL_18744049:20445-20864;EPI_ISL_19022858:20440-20859;EPI_ISL_19028778:20357-20776;EPI_ISL_19151800:20441-20860;EPI_ISL_19159116:20443-20862;EPI_ISL_19350788:21324-21743;EPI_ISL_19359677:20440-20859;EPI_ISL_19467692:22723-23142;JX878411.1:23438-23857;JX878425.1:22909-23370;JX878426.1:22393-22812;KC257460.1:22373-22792;KJ642612.1:20678-21097;KJ642613.1:22259-22678;KJ642616.1:21953-22372;KJ642618.1:19850-20269;KP849469.1:22760-23179;KP849471.1:22681-23100;LC831698.1:20441-20860;MN648051.1:20663-21082;MN702445.1:16106-16525;MN702446.1:22252-22671;MN702447.1:15612-16031;MN702449.1:16117-16536;MN702452.1:22289-22708;MT903338.1:20606-21025;MT903340.1:20443-20862;MT903341.1:11976-12395;MT903342.1:20458-20877;ON568298.1:20589-21008;ON622713.1:20675-21094;ON622722.2:20446-20865;ON631963.1:20466-20885;ON649879.1:20276-20695;ON676707.1:20496-20915;ON755039.1:20106-20525;ON808413.1:20446-20865;ON880420.1:20673-21092;ON911481.2:20463-20882;ON918656.1:20478-20897;ON959133.1:20567-20986;ON959136.1:20568-20987;ON983168.1:19142-19561;OP019276.1:20440-20859;OP150924.1:20581-21000;OP160532.1:20406-20825;OP235282.1:20440-20859;OP289782.1:20443-20862;OP331335.1:20460-20879;OP382479.1:20447-20866;OP422341.1:20460-20879;OP434519.1:20439-20858;OP535324.1:20441-20860;OP535333.1:20550-20969;OP536770.1:20442-20861;OP597769.1:20464-20883;OP604566.1:20443-20862;OP612682.1:20369-20788;OP612684.1:20433-20852;OP612686.1:20433-20852;OP696841.1:17717-18136;OP879722.1:19666-20085;OQ419007.1:19676-20095;OQ419012.1:19668-20087;OQ419031.1:19673-20092;OQ419047.1:19676-20095;OQ451898.1:19528-19947;OQ451900.1:20441-20860;OQ581853.1:20467-20886;OQ581855.1:20548-20967;OQ721951.1:20481-20900;OQ721954.1:20438-20857;OR035652.1:20390-20809;OR035664.1:20390-20809;OR035702.1:20390-20809;OR146272.1:20406-20825;OR146330.1:20451-20870;OR264364.1:20461-20880;OR427332.1:20461-20880;OR459777.1:20445-20864;OR459797.1:20460-20879;OR943698.1:22427-22846;PP098589.2:20442-20861;PP496048.1:20446-20865;PP601206.1:22442-22861;PP776553.1:20391-20810;PP852971.1:20440-20859;PP852983.1:20445-20864;PP853000.1:20407-20826;PP853005.1:20415-20834;PP853007.1:20447-20866;PP853013.1:20421-20840;PP853027.1:20452-20871;PP853045.1:20433-20852;PP853048.1:20452-20871;PP860026.1:20447-20866;PP860027.1:20458-20877;PP860028.1:20414-20833;PP860031.1:20468-20887;PP860033.1:20457-20876;PQ153226.1:20440-20859;PQ178862.2:21301-21720;PQ221506.1:16263-16682;PQ221833.1:22434-22853;PQ340477.1:20423-20842;PQ340478.1:20408-20827;PQ340480.1:20487-20906;PQ340483.1:20471-20890;PQ340485.1:20457-20876;PQ340486.1:20472-20891;PQ340487.1:20497-20916;PQ340489.1:20472-20891;PQ340490.1:20423-20842;PQ340491.1:20481-20900;PQ340493.1:20457-20876;PQ340495.1:20487-20906;PQ340496.1:20520-20939;PQ368489.1:20443-20862;PQ451835.1:20531-20950;PQ451836.1:20463-20882;PQ451838.1:20514-20933;PQ451841.1:20474-20893;PQ451842.1:20426-20845 |
| OG0000205 | AY603973.1:9251-9679;AY741551.1:9075-9503;AY753185.1:9462-9890;DQ011154.1:7654-8082;DQ011155.1:7566-7994;EPI_ISL_13331598:7595-8023;EPI_ISL_13363142:7588-8016;EPI_ISL_13374487:7603-8031;EPI_ISL_13502582:7588-8016;EPI_ISL_13508471:7680-8108;EPI_ISL_13607904:7579-8007;EPI_ISL_13624509:7523-7951;EPI_ISL_13889442:7698-8126;EPI_ISL_13953610:7579-8007;EPI_ISL_14181948:7724-8152;EPI_ISL_14838587:7551-7979;EPI_ISL_15263355:7576-8004;EPI_ISL_15269699:7548-7976;EPI_ISL_15293002:7821-8249;EPI_ISL_15641561:7537-7965;EPI_ISL_15831211:7586-8014;EPI_ISL_15992098:7601-8029;EPI_ISL_16233784:7597-8025;EPI_ISL_16946400:7586-8014;EPI_ISL_17048206:7236-7664;EPI_ISL_17703567:7601-8029;EPI_ISL_17718458:7579-8007;EPI_ISL_18213374:7576-8004;EPI_ISL_18360394:7237-7665;EPI_ISL_18557816:7577-8005;EPI_ISL_18642365:7579-8007;EPI_ISL_18659846:7549-7977;EPI_ISL_18744049:7581-8009;EPI_ISL_19022858:7576-8004;EPI_ISL_19028778:7493-7921;EPI_ISL_19151800:7577-8005;EPI_ISL_19159116:7579-8007;EPI_ISL_19350788:7562-7990;EPI_ISL_19359677:7576-8004;EPI_ISL_19446306:7571-7999;EPI_ISL_19467692:7790-8218;JX878411.1:8494-8922;JX878425.1:7934-8362;JX878426.1:7499-7927;KC257460.1:7332-7760;KJ642612.1:5838-6266;KJ642613.1:7337-7765;KJ642616.1:9068-9496;KJ642618.1:4914-5342;KP849469.1:7757-8185;KP849471.1:7740-8168;LC831698.1:7577-8005;MN648051.1:7798-8226;MN702445.1:1230-1658;MN702446.1:7364-7792;MN702447.1:731-1159;MN702449.1:1230-1658;MN702452.1:7406-7834;MT903338.1:7742-8170;MT903340.1:7579-8007;MT903342.1:7594-8022;MT903347.1:9338-9766;ON568298.1:7725-8153;ON622713.1:7810-8238;ON622722.2:7582-8010;ON631963.1:7602-8030;ON649879.1:7412-7840;ON676707.1:7632-8060;ON755039.1:7242-7670;ON808413.1:7582-8010;ON880420.1:7809-8237;ON911481.2:7599-8027;ON918656.1:7614-8042;ON959133.1:7703-8131;ON959136.1:7704-8132;ON983168.1:6278-6706;OP019276.1:7576-8004;OP150924.1:7717-8145;OP160532.1:7542-7970;OP235282.1:7576-8004;OP289782.1:7579-8007;OP331335.1:7596-8024;OP382479.1:7583-8011;OP422341.1:7596-8024;OP434519.1:7575-8003;OP535324.1:7577-8005;OP535333.1:7686-8114;OP536770.1:7578-8006;OP597769.1:7600-8028;OP604566.1:7579-8007;OP612682.1:7505-7933;OP612684.1:7569-7997;OP612686.1:7569-7997;OP879722.1:6802-7230;OQ419007.1:6812-7240;OQ419012.1:6804-7232;OQ419031.1:6809-7237;OQ419047.1:6812-7240;OQ451898.1:7577-8005;OQ451900.1:7577-8005;OQ581853.1:7603-8031;OQ581855.1:7684-8112;OQ721951.1:7617-8045;OQ721954.1:7574-8002;OR035652.1:7526-7954;OR035664.1:7526-7954;OR035702.1:7526-7954;OR146272.1:7542-7970;OR146330.1:7587-8015;OR264364.1:7597-8025;OR427332.1:7597-8025;OR459777.1:7581-8009;OR459797.1:7596-8024;OR943698.1:7516-7944;PP098589.2:7578-8006;PP496048.1:7582-8010;PP601206.1:7551-7979;PP776553.1:7527-7955;PP852971.1:7576-8004;PP852983.1:7581-8009;PP853000.1:7540-7968;PP853005.1:7551-7979;PP853007.1:7588-8016;PP853013.1:7557-7823;PP853027.1:7588-8016;PP853045.1:7592-8020;PP853048.1:7588-8016;PP860026.1:7581-8009;PP860027.1:7582-8010;PP860028.1:7550-7978;PP860031.1:7595-8023;PP860033.1:7581-8009;PQ153226.1:7576-8004;PQ178862.2:7583-8011;PQ221506.1:1376-1804;PQ221833.1:7550-7978;PQ340477.1:7559-7987;PQ340478.1:7544-7972;PQ340480.1:7623-8051;PQ340483.1:7607-8035;PQ340485.1:7593-8021;PQ340486.1:7608-8036;PQ340487.1:7633-8061;PQ340489.1:7608-8036;PQ340490.1:7559-7987;PQ340491.1:7617-8045;PQ340493.1:7593-8021;PQ340495.1:7623-8051;PQ340496.1:7656-8084;PQ368489.1:7579-8007;PQ451835.1:7667-8095;PQ451836.1:7599-8027;PQ451838.1:7650-8078;PQ451841.1:7610-8038;PQ451842.1:7562-7990 |
| OG0000206 | AY603973.1:49490-49891;AY741551.1:49494-49895;AY753185.1:49733-50134;DQ011154.1:49893-50096;DQ011155.1:49873-50076;EPI_ISL_13331598:47561-47962;EPI_ISL_13363142:47554-47955;EPI_ISL_13374487:47569-47970;EPI_ISL_13502582:47554-47955;EPI_ISL_13508471:47646-48047;EPI_ISL_13607904:47545-47946;EPI_ISL_13624509:47489-47890;EPI_ISL_13889442:47664-48065;EPI_ISL_13953610:47505-47906;EPI_ISL_14181948:47690-48091;EPI_ISL_14838587:49747-49950;EPI_ISL_15263355:47545-47946;EPI_ISL_15269699:47514-47915;EPI_ISL_15293002:47787-48188;EPI_ISL_15641561:47503-47904;EPI_ISL_15831211:47552-47953;EPI_ISL_15992098:47567-47968;EPI_ISL_16233784:47563-47964;EPI_ISL_16946400:47552-47953;EPI_ISL_17048206:47202-47603;EPI_ISL_17703567:47567-47968;EPI_ISL_17718458:47545-47946;EPI_ISL_18213374:47542-47943;EPI_ISL_18360394:47203-47604;EPI_ISL_18557816:47543-47944;EPI_ISL_18642365:47545-47946;EPI_ISL_18659846:47515-47916;EPI_ISL_18744049:47547-47948;EPI_ISL_19022858:47542-47943;EPI_ISL_19028778:47459-47860;EPI_ISL_19151800:47543-47944;EPI_ISL_19159116:47545-47946;EPI_ISL_19350788:48621-48824;EPI_ISL_19359677:47542-47943;EPI_ISL_19446306:47537-47938;EPI_ISL_19467692:50026-50229;JX878411.1:50740-50943;JX878425.1:50255-50458;KC257460.1:49671-49874;KJ642613.1:49563-49766;KJ642616.1:49184-49585;KJ642618.1:47153-47389;KP849469.1:50063-50266;KP849471.1:49988-50191;LC831698.1:47543-47944;MN648051.1:47765-48166;MN702445.1:43408-43611;MN702446.1:49553-49756;MN702447.1:42919-43122;MN702449.1:43419-43622;MN702452.1:49592-49795;MT903338.1:47708-48109;MT903340.1:47545-47946;MT903341.1:39078-39479;MT903342.1:47560-47961;MT903347.1:49500-49688;MT903347.1:49693-49902;ON568298.1:47691-48092;ON622713.1:47777-48178;ON622722.2:47548-47949;ON631963.1:47568-47969;ON649879.1:47378-47779;ON676707.1:47598-47999;ON755039.1:47208-47609;ON808413.1:47548-47949;ON880420.1:47775-48176;ON911481.2:47565-47966;ON918656.1:47580-47981;ON959133.1:47669-48070;ON959136.1:47670-48071;ON983168.1:46244-46645;OP019276.1:47542-47943;OP150924.1:47683-48084;OP160532.1:47508-47909;OP235282.1:47542-47943;OP289782.1:47545-47946;OP331335.1:47562-47963;OP382479.1:47549-47950;OP422341.1:47562-47963;OP434519.1:47541-47942;OP535324.1:47543-47944;OP535333.1:47652-48053;OP536770.1:47544-47945;OP597769.1:47566-47967;OP604566.1:47545-47946;OP612682.1:47471-47872;OP612684.1:47535-47936;OP612686.1:47535-47936;OP696841.1:44819-45220;OP879722.1:46768-47169;OQ419007.1:46778-47179;OQ419012.1:46767-47168;OQ419031.1:46749-47150;OQ419047.1:46778-47179;OQ451898.1:46630-47031;OQ451900.1:47543-47944;OQ581853.1:47569-47970;OQ581855.1:47650-48051;OQ721951.1:47583-47984;OQ721954.1:47540-47941;OR035652.1:47492-47893;OR035664.1:47492-47893;OR035702.1:47492-47893;OR146272.1:47508-47909;OR146330.1:47553-47954;OR264364.1:47563-47964;OR427332.1:47563-47964;OR459777.1:47547-47948;OR459797.1:47562-47963;OR943698.1:49727-49930;PP098589.2:47544-47945;PP496048.1:47548-47949;PP601206.1:49745-49948;PP776553.1:47469-47870;PP852971.1:47542-47943;PP852983.1:47548-47949;PP853000.1:47509-47910;PP853005.1:47517-47918;PP853007.1:47550-47951;PP853013.1:47523-47924;PP853027.1:47554-47955;PP853045.1:47535-47936;PP853048.1:47554-47955;PP860026.1:47552-47953;PP860027.1:47577-47978;PP860028.1:47518-47919;PP860031.1:47571-47972;PP860033.1:47576-47977;PQ153226.1:47542-47943;PQ178862.2:48598-48801;PQ221833.1:49738-49941;PQ340477.1:47525-47926;PQ340478.1:47510-47911;PQ340480.1:47589-47990;PQ340483.1:47573-47974;PQ340485.1:47559-47960;PQ340486.1:47574-47975;PQ340487.1:47599-48000;PQ340489.1:47574-47975;PQ340490.1:47525-47926;PQ340491.1:47580-47981;PQ340493.1:47559-47960;PQ340495.1:47589-47990;PQ340496.1:47622-48023;PQ368489.1:47545-47946;PQ451835.1:47630-48031;PQ451836.1:47562-47963;PQ451838.1:47613-48014;PQ451841.1:47573-47974;PQ451842.1:47525-47926 |
| OG0000207 | AY603973.1:17051-16893;AY741551.1:16888-16730;AY753185.1:17297-17139;DQ011154.1:15554-15396;DQ011155.1:15534-15376;EPI_ISL_13331598:15368-15210;EPI_ISL_13363142:15361-15203;EPI_ISL_13374487:15376-15218;EPI_ISL_13502582:15361-15203;EPI_ISL_13508471:15453-15295;EPI_ISL_13607904:15352-15194;EPI_ISL_13624509:15296-15138;EPI_ISL_13889442:15471-15313;EPI_ISL_13953610:15352-15194;EPI_ISL_14181948:15497-15339;EPI_ISL_14838587:15408-15250;EPI_ISL_15263355:15349-15191;EPI_ISL_15269699:15321-15163;EPI_ISL_15293002:15594-15436;EPI_ISL_15641561:15310-15152;EPI_ISL_15831211:15359-15201;EPI_ISL_15992098:15374-15216;EPI_ISL_16233784:15370-15212;EPI_ISL_16946400:15359-15201;EPI_ISL_17048206:15009-14851;EPI_ISL_17703567:15374-15216;EPI_ISL_17718458:15352-15194;EPI_ISL_18213374:15349-15191;EPI_ISL_18360394:15010-14852;EPI_ISL_18557816:15350-15192;EPI_ISL_18642365:15352-15194;EPI_ISL_18659846:15322-15164;EPI_ISL_18744049:15354-15196;EPI_ISL_19022858:15349-15191;EPI_ISL_19028778:15266-15108;EPI_ISL_19151800:15350-15192;EPI_ISL_19159116:15352-15194;EPI_ISL_19350788:15420-15262;EPI_ISL_19359677:15349-15191;EPI_ISL_19446306:15344-15186;EPI_ISL_19467692:15686-15528;JX878411.1:16399-16241;JX878425.1:15914-15756;JX878426.1:15356-15198;KC257460.1:15333-15175;KJ642612.1:13641-13483;KJ642613.1:15222-15064;KJ642616.1:16868-16710;KJ642618.1:12812-12654;KP849469.1:15724-15566;KP849471.1:15644-15486;LC831698.1:15350-15192;MN702445.1:9064-8906;MN702446.1:15210-15052;MN702447.1:8576-8418;MN702449.1:9072-8914;MN702452.1:15253-15095;MT903338.1:15515-15357;MT903340.1:15352-15194;MT903341.1:6885-6727;MT903342.1:15367-15209;MT903347.1:17224-17066;ON568298.1:15498-15340;ON622722.2:15355-15197;ON631963.1:15375-15217;ON649879.1:15185-15027;ON676707.1:15405-15247;ON755039.1:15015-14857;ON808413.1:15355-15197;ON880420.1:15582-15424;ON911481.2:15372-15214;ON918656.1:15387-15229;ON959133.1:15476-15318;ON959136.1:15477-15319;ON983168.1:14051-13893;OP019276.1:15349-15191;OP150924.1:15490-15332;OP160532.1:15315-15157;OP235282.1:15349-15191;OP289782.1:15352-15194;OP331335.1:15369-15211;OP382479.1:15356-15198;OP422341.1:15369-15211;OP434519.1:15348-15190;OP535324.1:15350-15192;OP535333.1:15459-15301;OP536770.1:15351-15193;OP597769.1:15373-15215;OP604566.1:15352-15194;OP612682.1:15278-15120;OP612684.1:15342-15184;OP612686.1:15342-15184;OP696841.1:12626-12468;OP879722.1:14575-14417;OQ419007.1:14585-14427;OQ419012.1:14577-14419;OQ419031.1:14582-14424;OQ419047.1:14585-14427;OQ451898.1:14437-14279;OQ451900.1:15350-15192;OQ581853.1:15376-15218;OQ581855.1:15457-15299;OQ721951.1:15390-15232;OQ721954.1:15347-15189;OR035652.1:15299-15141;OR035664.1:15299-15141;OR035702.1:15299-15141;OR146272.1:15315-15157;OR146330.1:15360-15202;OR264364.1:15370-15212;OR427332.1:15370-15212;OR459777.1:15354-15196;OR459797.1:15369-15211;OR943698.1:15391-15233;PP098589.2:15351-15193;PP496048.1:15355-15197;PP601206.1:15406-15248;PP776553.1:15300-15142;PP852971.1:15349-15191;PP852983.1:15354-15196;PP853000.1:15316-15158;PP853005.1:15324-15166;PP853007.1:15356-15198;PP853013.1:15330-15172;PP853027.1:15361-15203;PP853045.1:15360-15202;PP853048.1:15361-15203;PP860026.1:15356-15198;PP860027.1:15355-15197;PP860028.1:15323-15165;PP860031.1:15362-15204;PP860033.1:15354-15196;PQ153226.1:15349-15191;PQ178862.2:15397-15239;PQ221506.1:9227-9069;PQ221833.1:15398-15240;PQ340477.1:15332-15174;PQ340478.1:15317-15159;PQ340480.1:15396-15238;PQ340483.1:15380-15222;PQ340485.1:15366-15208;PQ340486.1:15381-15223;PQ340487.1:15406-15248;PQ340489.1:15381-15223;PQ340490.1:15332-15174;PQ340491.1:15390-15232;PQ340493.1:15366-15208;PQ340495.1:15396-15238;PQ340496.1:15429-15271;PQ368489.1:15352-15194;PQ451835.1:15440-15282;PQ451836.1:15372-15214;PQ451838.1:15423-15265;PQ451841.1:15383-15225;PQ451842.1:15335-15177 |
| OG0000208 | AY603973.1:190097-190255;AY741551.1:189834-189992;AY753185.1:190269-190427;DQ011154.1:188064-188222;DQ011155.1:187923-188081;EPI_ISL_13331598:188343-188501;EPI_ISL_13363142:188102-188260;EPI_ISL_13374487:188157-188315;EPI_ISL_13502582:188151-188309;EPI_ISL_13508471:188202-188360;EPI_ISL_13607904:188394-188552;EPI_ISL_13624509:187984-188142;EPI_ISL_13889442:188243-188401;EPI_ISL_13953610:188050-188208;EPI_ISL_14181948:188184-188342;EPI_ISL_14838587:187823-187981;EPI_ISL_15263355:187846-188004;EPI_ISL_15269699:188070-188228;EPI_ISL_15293002:188665-188823;EPI_ISL_15641561:187483-187641;EPI_ISL_15831211:188122-188280;EPI_ISL_15992098:188101-188259;EPI_ISL_16233784:188122-188280;EPI_ISL_16946400:188055-188213;EPI_ISL_17048206:187727-187885;EPI_ISL_17703567:188123-188281;EPI_ISL_17718458:188100-188258;EPI_ISL_18213374:188128-188286;EPI_ISL_18360394:186797-186955;EPI_ISL_18557816:188075-188233;EPI_ISL_18642365:188134-188292;EPI_ISL_18659846:188404-188562;EPI_ISL_18744049:188129-188287;EPI_ISL_19022858:188073-188231;EPI_ISL_19028778:188043-188201;EPI_ISL_19151800:186982-187140;EPI_ISL_19159116:188112-188270;EPI_ISL_19350788:186497-186655;EPI_ISL_19359677:188127-188285;EPI_ISL_19446306:188114-188272;EPI_ISL_19467692:188087-188245;JX878411.1:188907-189065;JX878425.1:188357-188515;JX878426.1:187771-187929;KC257460.1:187678-187836;KJ642612.1:185873-186031;KJ642613.1:187628-187786;KJ642616.1:189829-189987;KJ642618.1:185391-185549;KP849469.1:188214-188372;KP849471.1:188031-188189;LC831698.1:188110-188268;MN648051.1:188317-188475;MN702445.1:181270-181428;MN702446.1:187626-187784;MN702447.1:180832-180990;MN702449.1:181317-181475;MN702452.1:187513-187671;MT903338.1:188322-188480;MT903340.1:188139-188297;MT903342.1:188224-188382;MT903347.1:189595-189753;ON568298.1:188185-188343;ON622713.1:188716-188874;ON622722.2:188047-188205;ON631963.1:188350-188508;ON649879.1:187850-188008;ON676707.1:188142-188300;ON755039.1:187733-187891;ON808413.1:188058-188216;ON880420.1:188670-188828;ON911481.2:188325-188483;ON918656.1:188135-188293;ON959133.1:188577-188735;ON959136.1:188599-188757;ON983168.1:187143-187301;OP019276.1:188126-188284;OP150924.1:188570-188728;OP160532.1:187976-188134;OP235282.1:188126-188284;OP289782.1:188130-188288;OP331335.1:188122-188280;OP382479.1:188103-188261;OP422341.1:188118-188276;OP535324.1:188166-188324;OP535333.1:188221-188379;OP536770.1:188129-188287;OP597769.1:188111-188269;OP604566.1:188135-188293;OP612682.1:188036-188194;OP612684.1:188276-188434;OP612686.1:188089-188247;OP696841.1:185349-185507;OP879722.1:187289-187447;OQ419007.1:187346-187504;OQ419012.1:187320-187478;OQ419031.1:187324-187482;OQ419047.1:187326-187484;OQ451898.1:187160-187318;OQ451900.1:188074-188232;OQ581853.1:188072-188230;OQ581855.1:188181-188339;OQ721951.1:188150-188308;OQ721954.1:188082-188240;OR035652.1:188462-188620;OR035664.1:188408-188566;OR035702.1:188425-188583;OR146272.1:188064-188222;OR146330.1:188143-188301;OR264364.1:188119-188277;OR427332.1:188119-188277;OR459777.1:188105-188263;OR459797.1:188117-188275;OR943698.1:187734-187892;PP098589.2:188128-188286;PP496048.1:188046-188204;PP601206.1:187778-187936;PP776553.1:187998-188156;PP852971.1:188117-188275;PP852983.1:188123-188281;PP853000.1:188008-188166;PP853005.1:188082-188240;PP853007.1:188065-188223;PP853013.1:188062-188220;PP853027.1:188103-188261;PP853045.1:187868-188026;PP853048.1:188063-188221;PP860026.1:188066-188224;PP860027.1:188046-188204;PP860028.1:188003-188161;PP860031.1:188044-188202;PP860033.1:188039-188197;PQ153226.1:188109-188267;PQ178862.2:186262-186420;PQ221506.1:181634-181792;PQ221833.1:187755-187913;PQ340477.1:188199-188357;PQ340478.1:188108-188266;PQ340480.1:188230-188388;PQ340483.1:188186-188344;PQ340485.1:188225-188383;PQ340486.1:188284-188442;PQ340487.1:188328-188486;PQ340489.1:188260-188418;PQ340490.1:188118-188276;PQ340491.1:188361-188519;PQ340493.1:188180-188338;PQ340495.1:188241-188399;PQ340496.1:188339-188497;PQ368489.1:188142-188300;PQ451835.1:188303-188461;PQ451836.1:188081-188239;PQ451838.1:188286-188444;PQ451841.1:188197-188355 |
| OG0000209 | AY603973.1:189838-190185;AY741551.1:189575-189922;AY753185.1:190010-190357;DQ011154.1:187804-188016;DQ011155.1:187663-187875;EPI_ISL_13331598:188084-188431;EPI_ISL_13363142:187843-188190;EPI_ISL_13374487:187898-188245;EPI_ISL_13502582:187892-188239;EPI_ISL_13508471:187943-188290;EPI_ISL_13607904:188135-188482;EPI_ISL_13624509:187725-188072;EPI_ISL_13889442:187984-188331;EPI_ISL_13953610:187791-188138;EPI_ISL_14181948:187925-188272;EPI_ISL_14838587:187563-187775;EPI_ISL_15263355:187587-187934;EPI_ISL_15269699:187811-188158;EPI_ISL_15293002:188406-188753;EPI_ISL_15641561:187224-187571;EPI_ISL_15831211:187863-188210;EPI_ISL_15992098:187842-188189;EPI_ISL_16233784:187863-188210;EPI_ISL_16946400:187796-188143;EPI_ISL_17048206:187468-187815;EPI_ISL_17703567:187864-188211;EPI_ISL_17718458:187841-188188;EPI_ISL_18213374:187869-188216;EPI_ISL_18360394:186538-186885;EPI_ISL_18557816:187816-188163;EPI_ISL_18642365:187875-188222;EPI_ISL_18659846:188145-188492;EPI_ISL_18744049:187870-188217;EPI_ISL_19022858:187814-188161;EPI_ISL_19028778:187784-188131;EPI_ISL_19151800:186723-187070;EPI_ISL_19159116:187853-188200;EPI_ISL_19350788:186237-186449;EPI_ISL_19359677:187868-188215;EPI_ISL_19446306:187855-188202;EPI_ISL_19467692:187827-188039;JX878411.1:188647-188859;JX878425.1:188097-188309;JX878426.1:187511-187723;KC257460.1:187418-187630;KJ642613.1:187368-187580;KJ642616.1:189570-189917;KJ642618.1:185131-185343;KP849469.1:187954-188166;KP849471.1:187771-187983;LC831698.1:187851-188198;MN648051.1:188058-188405;MN702445.1:181010-181222;MN702446.1:187366-187578;MN702447.1:180572-180784;MN702449.1:181057-181269;MN702452.1:187253-187465;MT903338.1:188063-188410;MT903340.1:187880-188227;MT903342.1:187965-188312;MT903347.1:189336-189683;ON568298.1:187926-188273;ON622713.1:188457-188804;ON622722.2:187788-188135;ON631963.1:188091-188438;ON649879.1:187591-187938;ON676707.1:187883-188230;ON755039.1:187474-187821;ON808413.1:187799-188146;ON880420.1:188411-188758;ON911481.2:188066-188413;ON918656.1:187876-188223;ON959133.1:188318-188665;ON959136.1:188340-188687;ON983168.1:186884-187231;OP019276.1:187867-188214;OP150924.1:188311-188658;OP160532.1:187717-188064;OP235282.1:187867-188214;OP289782.1:187871-188218;OP331335.1:187863-188210;OP382479.1:187844-188191;OP422341.1:187859-188206;OP535324.1:187907-188254;OP535333.1:187962-188309;OP536770.1:187870-188217;OP597769.1:187852-188199;OP604566.1:187876-188223;OP612682.1:187777-188124;OP612684.1:188017-188364;OP612686.1:187830-188177;OP696841.1:185090-185437;OP879722.1:187030-187377;OQ419007.1:187087-187434;OQ419012.1:187061-187408;OQ419031.1:187065-187412;OQ419047.1:187067-187414;OQ451898.1:186901-187248;OQ451900.1:187815-188162;OQ581853.1:187813-188160;OQ581855.1:187922-188269;OQ721951.1:187891-188238;OQ721954.1:187823-188170;OR035652.1:188203-188550;OR035664.1:188149-188496;OR035702.1:188166-188513;OR146272.1:187805-188152;OR146330.1:187884-188231;OR264364.1:187860-188207;OR427332.1:187860-188207;OR459777.1:187846-188193;OR459797.1:187858-188205;OR943698.1:187474-187686;PP098589.2:187869-188216;PP496048.1:187787-188134;PP601206.1:187518-187730;PP776553.1:187739-188086;PP852971.1:187858-188205;PP852983.1:187864-188211;PP853000.1:187749-188096;PP853005.1:187823-188170;PP853007.1:187806-188153;PP853013.1:187803-188150;PP853027.1:187844-188191;PP853045.1:187609-187956;PP853048.1:187804-188151;PP860026.1:187807-188154;PP860027.1:187787-188134;PP860028.1:187744-188091;PP860031.1:187785-188132;PP860033.1:187780-188127;PQ153226.1:187850-188197;PQ178862.2:186002-186214;PQ221506.1:181374-181586;PQ221833.1:187495-187707;PQ340477.1:187940-188287;PQ340478.1:187849-188196;PQ340480.1:187971-188318;PQ340483.1:187927-188274;PQ340485.1:187966-188313;PQ340486.1:188025-188372;PQ340487.1:188069-188416;PQ340489.1:188001-188348;PQ340490.1:187859-188206;PQ340491.1:188102-188449;PQ340493.1:187921-188268;PQ340495.1:187982-188329;PQ340496.1:188080-188427;PQ368489.1:187883-188230;PQ451835.1:188044-188391;PQ451836.1:187822-188169;PQ451838.1:188027-188374;PQ451841.1:187938-188285 |
| OG0000210 | AY603973.1:174487-174206;AY741551.1:174496-174215;AY753185.1:174750-174469;DQ011154.1:172522-172241;DQ011155.1:172428-172147;EPI_ISL_13331598:172611-172330;EPI_ISL_13363142:172599-172318;EPI_ISL_13374487:172632-172351;EPI_ISL_13502582:172627-172346;EPI_ISL_13508471:172693-172412;EPI_ISL_13607904:172598-172317;EPI_ISL_13624509:172533-172252;EPI_ISL_13889442:172726-172445;EPI_ISL_13953610:172527-172246;EPI_ISL_14181948:172756-172475;EPI_ISL_14838587:172350-172069;EPI_ISL_15263355:172592-172311;EPI_ISL_15269699:172561-172280;EPI_ISL_15293002:172830-172549;EPI_ISL_15641561:172059-171778;EPI_ISL_15831211:172601-172320;EPI_ISL_15992098:172641-172360;EPI_ISL_16233784:172613-172332;EPI_ISL_16946400:172603-172322;EPI_ISL_17048206:172267-171986;EPI_ISL_17703567:172614-172333;EPI_ISL_17718458:172583-172302;EPI_ISL_18213374:172605-172324;EPI_ISL_18360394:171912-171631;EPI_ISL_18557816:172608-172327;EPI_ISL_18642365:172603-172322;EPI_ISL_18659846:172562-172281;EPI_ISL_18744049:172612-172331;EPI_ISL_19022858:172568-172287;EPI_ISL_19028778:172520-172239;EPI_ISL_19151800:171515-171234;EPI_ISL_19159116:172589-172308;EPI_ISL_19350788:170979-170698;EPI_ISL_19359677:172604-172323;EPI_ISL_19446306:172591-172310;EPI_ISL_19467692:172591-172310;JX878411.1:173363-173082;JX878425.1:172815-172534;JX878426.1:172286-172005;KC257460.1:172178-171897;KJ642612.1:170476-170195;KJ642613.1:172083-171802;KJ642616.1:174194-173913;KJ642618.1:169996-169715;KP849469.1:172710-172429;KP849471.1:172513-172232;LC831698.1:172587-172306;MN648051.1:172830-172549;MN702445.1:165890-165609;MN702446.1:172060-171779;MN702447.1:165406-165125;MN702449.1:165928-165647;MN702452.1:172112-171831;MT903338.1:172763-172482;MT903340.1:172616-172335;MT903341.1:164157-163876;MT903342.1:172653-172372;MT903347.1:174052-173771;ON568298.1:172757-172476;ON622713.1:172821-172540;ON622722.2:172609-172328;ON631963.1:172618-172337;ON649879.1:172424-172143;ON755039.1:172246-171965;ON808413.1:172583-172302;ON880420.1:172819-172538;ON911481.2:172604-172323;ON918656.1:172626-172345;ON959133.1:172715-172434;ON959136.1:172716-172435;ON983168.1:171292-171011;OP019276.1:172603-172322;OP150924.1:172729-172448;OP160532.1:172552-172271;OP235282.1:172603-172322;OP289782.1:172607-172326;OP331335.1:172613-172332;OP382479.1:172596-172315;OP422341.1:172609-172328;OP434519.1:172585-172304;OP535324.1:172617-172336;OP535333.1:172723-172442;OP536770.1:172606-172325;OP597769.1:172640-172359;OP604566.1:172606-172325;OP612682.1:172541-172260;OP612684.1:172592-172311;OP612686.1:172594-172313;OP696841.1:169882-169601;OP879722.1:171829-171548;OQ419007.1:171823-171542;OQ419012.1:171797-171516;OQ419031.1:171801-171520;OQ419047.1:171803-171522;OQ451898.1:171693-171412;OQ451900.1:172607-172326;OQ581853.1:172615-172334;OQ581855.1:172714-172433;OQ721951.1:172633-172352;OQ721954.1:172606-172325;OR035652.1:172535-172254;OR035664.1:172539-172258;OR035702.1:172536-172255;OR146272.1:172555-172274;OR146330.1:172616-172335;OR264364.1:172610-172329;OR427332.1:172610-172329;OR459777.1:172588-172307;OR459797.1:172608-172327;OR943698.1:172121-171840;PP098589.2:172605-172324;PP496048.1:172609-172328;PP601206.1:172298-172017;PP776553.1:172529-172248;PP852971.1:172594-172313;PP853013.1:172589-172308;PP853045.1:172570-172289;PP860026.1:172603-172322;PP860027.1:172622-172341;PP860028.1:172552-172271;PP860031.1:172594-172313;PP860033.1:172605-172324;PQ153226.1:172586-172305;PQ178862.2:170949-170668;PQ221506.1:166150-165869;PQ221833.1:172278-171997;PQ340477.1:172573-172292;PQ340478.1:172555-172274;PQ340480.1:172633-172352;PQ340483.1:172619-172338;PQ340485.1:172602-172321;PQ340486.1:172620-172339;PQ340487.1:172644-172363;PQ340489.1:172619-172338;PQ340490.1:172571-172290;PQ340491.1:172626-172345;PQ340493.1:172610-172329;PQ340495.1:172636-172355;PQ340496.1:172671-172390;PQ368489.1:172619-172338;PQ451835.1:172676-172395;PQ451836.1:172614-172333;PQ451838.1:172664-172383;PQ451841.1:172627-172346;PQ451842.1:172573-172292 |
| OG0000211 | DQ011154.1:189940-190161;DQ011155.1:189799-190020;EPI_ISL_13331598:190300-190521;EPI_ISL_13363142:190059-190280;EPI_ISL_13374487:190114-190335;EPI_ISL_13502582:190108-190329;EPI_ISL_13508471:190159-190380;EPI_ISL_13607904:190351-190572;EPI_ISL_13624509:189941-190162;EPI_ISL_13889442:190200-190421;EPI_ISL_13953610:190007-190228;EPI_ISL_14181948:190141-190362;EPI_ISL_14838587:189699-189920;EPI_ISL_15263355:189803-190024;EPI_ISL_15269699:190027-190248;EPI_ISL_15293002:190622-190843;EPI_ISL_15641561:189440-189661;EPI_ISL_15831211:190079-190300;EPI_ISL_15992098:190058-190279;EPI_ISL_16233784:190079-190300;EPI_ISL_16946400:190012-190233;EPI_ISL_17048206:189684-189905;EPI_ISL_17703567:190080-190301;EPI_ISL_17718458:190057-190278;EPI_ISL_18213374:190085-190306;EPI_ISL_18360394:188754-188975;EPI_ISL_18557816:190032-190253;EPI_ISL_18642365:190091-190312;EPI_ISL_18659846:190361-190582;EPI_ISL_18744049:190086-190307;EPI_ISL_19022858:190030-190251;EPI_ISL_19028778:190000-190221;EPI_ISL_19151800:188939-189160;EPI_ISL_19159116:190069-190290;EPI_ISL_19350788:188373-188594;EPI_ISL_19359677:190084-190305;EPI_ISL_19446306:190071-190292;EPI_ISL_19467692:189963-190184;JX878411.1:190782-191003;JX878426.1:189647-189868;KC257460.1:189554-189775;KJ642612.1:187749-187970;KJ642613.1:189504-189725;KJ642618.1:187262-187483;KP849469.1:190089-190310;KP849471.1:189907-190128;LC831698.1:190067-190288;MN648051.1:190274-190495;MN702445.1:183146-183367;MN702446.1:189502-189723;MN702447.1:182708-182929;MN702449.1:183193-183414;MN702452.1:189389-189610;MT903338.1:190279-190500;MT903340.1:190096-190317;MT903341.1:177965-178186;MT903342.1:190181-190402;ON568298.1:190142-190363;ON622713.1:190673-190894;ON622722.2:190004-190225;ON631963.1:190307-190528;ON649879.1:189807-190028;ON676707.1:190099-190320;ON755039.1:189690-189911;ON808413.1:190015-190236;ON880420.1:190627-190848;ON911481.2:190282-190503;ON918656.1:190092-190313;ON959133.1:190534-190755;ON959136.1:190556-190777;ON983168.1:189100-189321;OP019276.1:190083-190304;OP150924.1:190527-190748;OP160532.1:189933-190154;OP235282.1:190083-190304;OP289782.1:190087-190308;OP331335.1:190079-190300;OP382479.1:190060-190281;OP422341.1:190075-190296;OP535324.1:190123-190344;OP535333.1:190178-190399;OP536770.1:190086-190307;OP597769.1:190068-190289;OP604566.1:190092-190313;OP612682.1:189993-190214;OP612684.1:190233-190454;OP612686.1:190046-190267;OP696841.1:187306-187527;OP879722.1:189246-189467;OQ419007.1:189303-189524;OQ419012.1:189277-189498;OQ419031.1:189281-189502;OQ419047.1:189283-189504;OQ451898.1:189117-189338;OQ451900.1:190031-190252;OQ581853.1:190029-190250;OQ581855.1:190138-190359;OQ721951.1:190107-190328;OQ721954.1:190039-190260;OR035652.1:190419-190640;OR035664.1:190365-190586;OR035702.1:190382-190603;OR146272.1:190021-190242;OR146330.1:190099-190320;OR264364.1:190076-190297;OR427332.1:190076-190297;OR459777.1:190062-190283;OR459797.1:190074-190295;OR943698.1:189610-189831;PP098589.2:190085-190306;PP496048.1:190003-190224;PP601206.1:189654-189875;PP776553.1:189955-190176;PP852971.1:190074-190295;PP852983.1:190080-190301;PP853000.1:189965-190186;PP853005.1:190039-190260;PP853007.1:190022-190243;PP853013.1:190019-190240;PP853027.1:190060-190281;PP853045.1:189825-190046;PP853048.1:190020-190241;PP860026.1:190023-190244;PP860027.1:190003-190224;PP860028.1:189960-190181;PP860031.1:190001-190222;PP860033.1:189996-190217;PQ153226.1:190066-190287;PQ178862.2:188138-188359;PQ221506.1:183510-183731;PQ221833.1:189631-189852;PQ340477.1:190156-190377;PQ340478.1:190065-190286;PQ340480.1:190187-190408;PQ340483.1:190143-190364;PQ340485.1:190182-190403;PQ340486.1:190241-190462;PQ340487.1:190285-190506;PQ340489.1:190217-190438;PQ340490.1:190075-190296;PQ340491.1:190318-190539;PQ340493.1:190137-190358;PQ340495.1:190198-190419;PQ340496.1:190296-190517;PQ368489.1:190099-190320;PQ451835.1:190260-190481;PQ451836.1:190038-190259;PQ451838.1:190243-190464;PQ451841.1:190154-190375 |
| OG0000212 | DQ011154.1:7219-7025;DQ011155.1:7131-6937;EPI_ISL_13331598:7158-6940;EPI_ISL_13363142:7151-6933;EPI_ISL_13374487:7166-6948;EPI_ISL_13502582:7151-6933;EPI_ISL_13508471:7243-7025;EPI_ISL_13607904:7142-6924;EPI_ISL_13624509:7086-6868;EPI_ISL_13889442:7261-7043;EPI_ISL_13953610:7142-6924;EPI_ISL_14181948:7287-7069;EPI_ISL_14838587:7116-6922;EPI_ISL_15263355:7139-6921;EPI_ISL_15269699:7111-6893;EPI_ISL_15293002:7384-7166;EPI_ISL_15641561:7100-6882;EPI_ISL_15831211:7149-6931;EPI_ISL_15992098:7164-6946;EPI_ISL_16233784:7160-6942;EPI_ISL_16946400:7149-6931;EPI_ISL_17048206:6799-6581;EPI_ISL_17703567:7164-6946;EPI_ISL_17718458:7142-6924;EPI_ISL_18213374:7139-6921;EPI_ISL_18360394:6800-6582;EPI_ISL_18557816:7140-6922;EPI_ISL_18642365:7142-6924;EPI_ISL_18659846:7112-6894;EPI_ISL_18744049:7144-6926;EPI_ISL_19022858:7139-6921;EPI_ISL_19028778:7056-6838;EPI_ISL_19151800:7140-6922;EPI_ISL_19159116:7142-6924;EPI_ISL_19350788:7127-6933;EPI_ISL_19359677:7139-6921;EPI_ISL_19446306:7134-6916;EPI_ISL_19467692:7355-7161;JX878411.1:8059-7865;JX878425.1:7499-7305;JX878426.1:7064-6870;KC257460.1:6897-6703;KJ642612.1:5403-5209;KJ642613.1:6902-6708;KP849469.1:7322-7128;KP849471.1:7305-7111;LC831698.1:7140-6922;MN648051.1:7361-7143;MN702445.1:795-625;MN702446.1:6929-6759;MN702447.1:296-102;MN702449.1:795-625;MN702452.1:6971-6777;MT903338.1:7305-7087;MT903340.1:7142-6924;MT903342.1:7157-6939;ON568298.1:7288-7070;ON622713.1:7373-7155;ON622722.2:7145-6927;ON631963.1:7165-6947;ON649879.1:6975-6757;ON676707.1:7195-6977;ON755039.1:6805-6587;ON808413.1:7145-6927;ON880420.1:7372-7154;ON911481.2:7162-6944;ON918656.1:7177-6959;ON959133.1:7266-7048;ON959136.1:7267-7049;ON983168.1:5841-5623;OP019276.1:7139-6921;OP150924.1:7280-7062;OP160532.1:7105-6887;OP235282.1:7139-6921;OP289782.1:7142-6924;OP331335.1:7159-6941;OP382479.1:7146-6928;OP422341.1:7159-6941;OP434519.1:7138-6920;OP535324.1:7140-6922;OP535333.1:7249-7031;OP536770.1:7141-6923;OP597769.1:7163-6945;OP604566.1:7142-6924;OP612682.1:7068-6850;OP612684.1:7132-6914;OP612686.1:7132-6914;OP879722.1:6365-6147;OQ419007.1:6375-6157;OQ419012.1:6367-6149;OQ419031.1:6372-6154;OQ419047.1:6375-6157;OQ451898.1:7140-6922;OQ451900.1:7140-6922;OQ581853.1:7166-6948;OQ581855.1:7247-7029;OQ721951.1:7180-6962;OQ721954.1:7137-6919;OR035652.1:7089-6871;OR035664.1:7089-6871;OR035702.1:7089-6871;OR146272.1:7105-6887;OR146330.1:7150-6932;OR264364.1:7160-6942;OR427332.1:7160-6942;OR459777.1:7144-6926;OR459797.1:7159-6941;OR943698.1:7081-6887;PP098589.2:7141-6923;PP496048.1:7145-6927;PP601206.1:7116-6922;PP776553.1:7090-6872;PP852971.1:7139-6921;PP852983.1:7144-6926;PP853000.1:7103-6885;PP853005.1:7114-6896;PP853007.1:7151-6933;PP853013.1:7120-6902;PP853027.1:7151-6933;PP853045.1:7155-6937;PP853048.1:7151-6933;PP860026.1:7144-6926;PP860027.1:7145-6927;PP860028.1:7113-6895;PP860031.1:7158-6940;PP860033.1:7144-6926;PQ153226.1:7139-6921;PQ178862.2:7148-6954;PQ221506.1:941-747;PQ221833.1:7115-6921;PQ340477.1:7122-6904;PQ340478.1:7107-6889;PQ340480.1:7186-6968;PQ340483.1:7170-6952;PQ340485.1:7156-6938;PQ340486.1:7171-6953;PQ340487.1:7196-6978;PQ340489.1:7171-6953;PQ340490.1:7122-6904;PQ340491.1:7180-6962;PQ340493.1:7156-6938;PQ340495.1:7186-6968;PQ340496.1:7219-7001;PQ368489.1:7142-6924;PQ451835.1:7230-7012;PQ451836.1:7162-6944;PQ451838.1:7213-6995;PQ451841.1:7173-6955;PQ451842.1:7125-6907 |
| OG0000213 | DQ011154.1:6974-6807;DQ011155.1:6886-6719;EPI_ISL_13331598:6936-6769;EPI_ISL_13363142:6929-6762;EPI_ISL_13374487:6944-6777;EPI_ISL_13502582:6929-6762;EPI_ISL_13508471:7021-6854;EPI_ISL_13607904:6920-6753;EPI_ISL_13624509:6864-6697;EPI_ISL_13889442:7039-6872;EPI_ISL_13953610:6920-6753;EPI_ISL_14181948:7065-6898;EPI_ISL_14838587:6871-6704;EPI_ISL_15263355:6917-6750;EPI_ISL_15269699:6889-6722;EPI_ISL_15293002:7162-6995;EPI_ISL_15641561:6878-6711;EPI_ISL_15831211:6927-6760;EPI_ISL_15992098:6942-6775;EPI_ISL_16233784:6938-6771;EPI_ISL_16946400:6927-6760;EPI_ISL_17048206:6577-6410;EPI_ISL_17703567:6942-6775;EPI_ISL_17718458:6920-6753;EPI_ISL_18213374:6917-6750;EPI_ISL_18360394:6578-6411;EPI_ISL_18557816:6918-6751;EPI_ISL_18642365:6920-6753;EPI_ISL_18659846:6890-6723;EPI_ISL_18744049:6922-6755;EPI_ISL_19022858:6917-6750;EPI_ISL_19028778:6834-6667;EPI_ISL_19151800:6918-6751;EPI_ISL_19159116:6920-6753;EPI_ISL_19350788:6882-6715;EPI_ISL_19359677:6917-6750;EPI_ISL_19446306:6912-6745;EPI_ISL_19467692:7110-6943;JX878411.1:7815-7648;JX878425.1:7254-7087;JX878426.1:6819-6652;KC257460.1:6652-6485;KJ642612.1:5158-4991;KJ642613.1:6657-6490;KP849469.1:7077-6910;KP849471.1:7060-6893;LC831698.1:6918-6751;MN648051.1:7139-6972;MN702445.1:574-407;MN702446.1:6708-6541;MN702449.1:574-407;MN702452.1:6726-6559;MT903338.1:7083-6916;MT903340.1:6920-6753;MT903342.1:6935-6768;ON568298.1:7066-6899;ON622713.1:7151-6984;ON622722.2:6923-6756;ON631963.1:6943-6776;ON649879.1:6753-6586;ON676707.1:6973-6806;ON755039.1:6583-6416;ON808413.1:6923-6756;ON880420.1:7150-6983;ON911481.2:6940-6773;ON918656.1:6955-6788;ON959133.1:7044-6877;ON959136.1:7045-6878;ON983168.1:5619-5452;OP019276.1:6917-6750;OP150924.1:7058-6891;OP160532.1:6883-6716;OP235282.1:6917-6750;OP289782.1:6920-6753;OP331335.1:6937-6770;OP382479.1:6924-6757;OP422341.1:6937-6770;OP434519.1:6916-6749;OP535324.1:6918-6751;OP535333.1:7027-6860;OP536770.1:6919-6752;OP597769.1:6941-6774;OP604566.1:6920-6753;OP612682.1:6846-6679;OP612684.1:6910-6743;OP612686.1:6910-6743;OP879722.1:6143-5976;OQ419007.1:6153-5986;OQ419012.1:6145-5978;OQ419031.1:6150-5983;OQ419047.1:6153-5986;OQ451898.1:6918-6751;OQ451900.1:6918-6751;OQ581853.1:6944-6777;OQ581855.1:7025-6858;OQ721951.1:6958-6791;OQ721954.1:6915-6748;OR035652.1:6867-6700;OR035664.1:6867-6700;OR035702.1:6867-6700;OR146272.1:6883-6716;OR146330.1:6928-6761;OR264364.1:6938-6771;OR427332.1:6938-6771;OR459777.1:6922-6755;OR459797.1:6937-6770;OR943698.1:6837-6670;PP098589.2:6919-6752;PP496048.1:6923-6756;PP601206.1:6871-6704;PP776553.1:6868-6701;PP852971.1:6917-6750;PP852983.1:6922-6755;PP853000.1:6881-6714;PP853005.1:6892-6725;PP853007.1:6929-6762;PP853013.1:6898-6731;PP853027.1:6929-6762;PP853045.1:6933-6766;PP853048.1:6929-6762;PP860026.1:6922-6755;PP860027.1:6923-6756;PP860028.1:6891-6724;PP860031.1:6936-6769;PP860033.1:6922-6755;PQ153226.1:6917-6750;PQ178862.2:6903-6736;PQ221506.1:696-529;PQ221833.1:6870-6703;PQ340477.1:6900-6733;PQ340478.1:6885-6718;PQ340480.1:6964-6797;PQ340483.1:6948-6781;PQ340485.1:6934-6767;PQ340486.1:6949-6782;PQ340487.1:6974-6807;PQ340489.1:6949-6782;PQ340490.1:6900-6733;PQ340491.1:6958-6791;PQ340493.1:6934-6767;PQ340495.1:6964-6797;PQ340496.1:6997-6830;PQ368489.1:6920-6753;PQ451835.1:7008-6841;PQ451836.1:6940-6773;PQ451838.1:6991-6824;PQ451841.1:6951-6784;PQ451842.1:6903-6736 |
| OG0000214 | AY603973.1:175133-175531;AY741551.1:175142-175540;AY753185.1:175396-175794;DQ011155.1:173112-173492;EPI_ISL_13331598:173497-173724;EPI_ISL_13363142:173535-173762;EPI_ISL_13374487:173592-173819;EPI_ISL_13502582:173584-173811;EPI_ISL_13508471:173581-173808;EPI_ISL_13607904:173487-173714;EPI_ISL_13624509:173417-173644;EPI_ISL_13889442:173676-173903;EPI_ISL_13953610:173411-173638;EPI_ISL_14181948:173644-173871;EPI_ISL_15263355:173382-173609;EPI_ISL_15269699:173449-173676;EPI_ISL_15293002:173720-173947;EPI_ISL_15641561:172943-173170;EPI_ISL_15831211:173561-173788;EPI_ISL_15992098:173561-173788;EPI_ISL_16233784:173501-173728;EPI_ISL_16946400:173485-173712;EPI_ISL_17048206:173187-173414;EPI_ISL_17703567:173502-173729;EPI_ISL_17718458:173467-173694;EPI_ISL_18213374:173489-173716;EPI_ISL_18360394:172765-172992;EPI_ISL_18557816:173508-173735;EPI_ISL_18642365:173495-173722;EPI_ISL_18659846:173450-173677;EPI_ISL_18744049:173496-173723;EPI_ISL_19022858:173452-173679;EPI_ISL_19028778:173404-173631;EPI_ISL_19151800:172415-172642;EPI_ISL_19159116:173473-173700;EPI_ISL_19359677:173488-173715;EPI_ISL_19446306:173475-173702;KC257460.1:172824-173228;KJ642616.1:174840-175238;KJ642618.1:170674-171054;LC831698.1:173471-173698;MN648051.1:173710-173937;MN702447.1:166078-166458;MT903338.1:173683-173910;MT903340.1:173500-173727;MT903341.1:165063-165290;MT903342.1:173603-173830;MT903347.1:174698-175096;ON568298.1:173645-173872;ON622713.1:173717-173944;ON622722.2:173505-173732;ON631963.1:173504-173731;ON649879.1:173310-173537;ON676707.1:173575-173802;ON755039.1:173126-173353;ON808413.1:173473-173700;ON880420.1:173707-173934;ON911481.2:173497-173724;ON918656.1:173514-173741;ON959133.1:173605-173832;ON959136.1:173600-173827;ON983168.1:172180-172407;OP019276.1:173487-173714;OP150924.1:173621-173848;OP160532.1:173436-173663;OP235282.1:173487-173714;OP289782.1:173491-173718;OP331335.1:173501-173728;OP382479.1:173482-173709;OP422341.1:173497-173724;OP434519.1:173469-173696;OP535324.1:173527-173754;OP535333.1:173609-173836;OP536770.1:173490-173717;OP597769.1:173570-173797;OP604566.1:173490-173717;OP612682.1:173397-173624;OP612684.1:173448-173675;OP612686.1:173450-173677;OP696841.1:170782-171009;OP879722.1:172749-172976;OQ419007.1:172707-172934;OQ419012.1:172681-172908;OQ419031.1:172685-172912;OQ419047.1:172687-172914;OQ451898.1:172593-172820;OQ451900.1:173507-173734;OQ581853.1:173505-173732;OQ581855.1:173614-173841;OQ721951.1:173493-173720;OQ721954.1:173526-173753;OR035652.1:173423-173650;OR035664.1:173427-173654;OR035702.1:173426-173653;OR146272.1:173443-173670;OR146330.1:173576-173803;OR264364.1:173498-173725;OR427332.1:173498-173725;OR459777.1:173472-173699;OR459797.1:173496-173723;PP098589.2:173489-173716;PP496048.1:173505-173732;PP776553.1:173431-173658;PP852971.1:173478-173705;PP852983.1:173502-173729;PP853000.1:173441-173668;PP853005.1:173461-173688;PP853007.1:173498-173725;PP853013.1:173495-173722;PP853027.1:173536-173763;PP853045.1:173476-173703;PP853048.1:173496-173723;PP860026.1:173503-173730;PP860027.1:173488-173721;PP860028.1:173440-173667;PP860031.1:173482-173709;PP860033.1:173481-173714;PQ153226.1:173470-173697;PQ340477.1:173461-173688;PQ340478.1:173451-173678;PQ340480.1:173519-173746;PQ340483.1:173511-173738;PQ340485.1:173496-173723;PQ340486.1:173510-173737;PQ340487.1:173530-173757;PQ340489.1:173513-173740;PQ340490.1:173461-173688;PQ340491.1:173524-173751;PQ340493.1:173496-173723;PQ340495.1:173530-173757;PQ340496.1:173565-173792;PQ368489.1:173503-173730;PQ451835.1:173574-173801;PQ451836.1:173514-173741;PQ451838.1:173566-173793;PQ451841.1:173531-173758;PQ451842.1:173467-173694 |
| OG0000215 | AY603973.1:157363-157554;AY741551.1:157434-157625;AY753185.1:157618-157809;EPI_ISL_13331598:155479-155670;EPI_ISL_13363142:155467-155658;EPI_ISL_13374487:155500-155691;EPI_ISL_13502582:155495-155686;EPI_ISL_13508471:155561-155752;EPI_ISL_13607904:155466-155657;EPI_ISL_13624509:155401-155592;EPI_ISL_13889442:155594-155785;EPI_ISL_13953610:155394-155585;EPI_ISL_14181948:155624-155815;EPI_ISL_15263355:155460-155651;EPI_ISL_15269699:155429-155620;EPI_ISL_15293002:155698-155889;EPI_ISL_15641561:155415-155606;EPI_ISL_15831211:155469-155660;EPI_ISL_15992098:155509-155700;EPI_ISL_16233784:155481-155672;EPI_ISL_16946400:155471-155662;EPI_ISL_17048206:155135-155326;EPI_ISL_17703567:155482-155673;EPI_ISL_17718458:155462-155653;EPI_ISL_18213374:155473-155664;EPI_ISL_18360394:154780-154971;EPI_ISL_18557816:155476-155667;EPI_ISL_18642365:155471-155662;EPI_ISL_18659846:155430-155621;EPI_ISL_18744049:155479-155670;EPI_ISL_19022858:155436-155627;EPI_ISL_19028778:155388-155579;EPI_ISL_19151800:155472-155663;EPI_ISL_19159116:155457-155648;EPI_ISL_19350788:156116-156310;EPI_ISL_19359677:155472-155663;EPI_ISL_19446306:155459-155650;KJ642616.1:157084-157275;LC831698.1:155455-155646;MN648051.1:155698-155889;MT903338.1:155631-155822;MT903340.1:155484-155675;MT903341.1:147025-147216;MT903342.1:155521-155712;MT903347.1:157427-157618;ON568298.1:155625-155816;ON622713.1:155689-155880;ON622722.2:155477-155668;ON631963.1:155486-155677;ON649879.1:155292-155483;ON676707.1:155523-155714;ON755039.1:155114-155305;ON808413.1:155451-155642;ON880420.1:155687-155878;ON911481.2:155472-155663;ON918656.1:155494-155685;ON959133.1:155583-155774;ON959136.1:155584-155775;ON983168.1:154160-154351;OP019276.1:155471-155662;OP150924.1:155597-155788;OP160532.1:155420-155611;OP235282.1:155471-155662;OP289782.1:155475-155666;OP331335.1:155480-155671;OP382479.1:155464-155655;OP422341.1:155477-155668;OP434519.1:155453-155644;OP535324.1:155484-155675;OP535333.1:155591-155782;OP536770.1:155474-155665;OP597769.1:155508-155699;OP604566.1:155474-155665;OP612682.1:155409-155600;OP612684.1:155460-155651;OP612686.1:155462-155653;OP696841.1:152750-152941;OP879722.1:154697-154888;OQ419007.1:154691-154882;OQ419012.1:154665-154856;OQ419031.1:154669-154860;OQ419047.1:154671-154862;OQ451898.1:154561-154752;OQ451900.1:155475-155666;OQ581853.1:155483-155674;OQ581855.1:155582-155773;OQ721951.1:155501-155692;OQ721954.1:155474-155665;OR035652.1:155403-155594;OR035664.1:155407-155598;OR035702.1:155404-155595;OR146272.1:155423-155614;OR146330.1:155484-155675;OR264364.1:155478-155669;OR427332.1:155478-155669;OR459777.1:155467-155658;OR459797.1:155476-155667;PP098589.2:155473-155664;PP496048.1:155477-155668;PP776553.1:155397-155588;PP852971.1:155465-155656;PP852983.1:155482-155673;PP853000.1:155433-155624;PP853005.1:155441-155632;PP853007.1:155484-155675;PP853013.1:155456-155647;PP853027.1:155498-155689;PP853045.1:155449-155640;PP853048.1:155474-155665;PP860026.1:155470-155661;PP860027.1:155506-155697;PP860028.1:155433-155624;PP860031.1:155503-155694;PP860033.1:155489-155680;PQ153226.1:155454-155645;PQ178862.2:156095-156289;PQ340477.1:155441-155632;PQ340478.1:155423-155614;PQ340480.1:155501-155692;PQ340483.1:155487-155678;PQ340485.1:155470-155661;PQ340486.1:155488-155679;PQ340487.1:155512-155703;PQ340489.1:155487-155678;PQ340490.1:155439-155630;PQ340491.1:155494-155685;PQ340493.1:155478-155669;PQ340495.1:155504-155695;PQ340496.1:155539-155730;PQ368489.1:155487-155678;PQ451835.1:155544-155735;PQ451836.1:155482-155673;PQ451838.1:155532-155723;PQ451841.1:155495-155686;PQ451842.1:155441-155632 |
| OG0000216 | AY603973.1:8489-8671;AY603973.1:190705-190523;AY741551.1:8313-8495;AY741551.1:190442-190260;AY753185.1:8591-8773;AY753185.1:190877-190695;EPI_ISL_13331598:188951-188769;EPI_ISL_13363142:188710-188528;EPI_ISL_13374487:188765-188583;EPI_ISL_13502582:188759-188577;EPI_ISL_13508471:188810-188628;EPI_ISL_13607904:189002-188820;EPI_ISL_13624509:188592-188410;EPI_ISL_13889442:188851-188669;EPI_ISL_13953610:188658-188476;EPI_ISL_14181948:188792-188610;EPI_ISL_15263355:188454-188272;EPI_ISL_15269699:188678-188496;EPI_ISL_15293002:189273-189091;EPI_ISL_15641561:188091-187909;EPI_ISL_15831211:188730-188548;EPI_ISL_15992098:188709-188527;EPI_ISL_16233784:188730-188548;EPI_ISL_16946400:188663-188481;EPI_ISL_17048206:188335-188153;EPI_ISL_17703567:188731-188549;EPI_ISL_17718458:188708-188526;EPI_ISL_18213374:188736-188554;EPI_ISL_18360394:187405-187223;EPI_ISL_18557816:188683-188501;EPI_ISL_18642365:188742-188560;EPI_ISL_18659846:189012-188830;EPI_ISL_18744049:188737-188555;EPI_ISL_19022858:188681-188499;EPI_ISL_19028778:188651-188469;EPI_ISL_19151800:187590-187408;EPI_ISL_19159116:188720-188538;EPI_ISL_19359677:188735-188553;EPI_ISL_19446306:188722-188540;KJ642616.1:8306-8488;KJ642616.1:190437-190255;LC831698.1:188718-188536;MN648051.1:188925-188743;MT903338.1:188930-188748;MT903340.1:188747-188565;MT903342.1:188832-188650;MT903347.1:8576-8758;MT903347.1:190203-190021;ON568298.1:188793-188611;ON622713.1:189324-189142;ON622722.2:188655-188473;ON631963.1:188958-188776;ON649879.1:188458-188276;ON676707.1:188750-188568;ON755039.1:188341-188159;ON808413.1:188666-188484;ON880420.1:189278-189096;ON911481.2:188933-188751;ON918656.1:188743-188561;ON959133.1:189185-189003;ON959136.1:189207-189025;ON983168.1:187751-187569;OP019276.1:188734-188552;OP150924.1:189178-188996;OP160532.1:188584-188402;OP235282.1:188734-188552;OP289782.1:188738-188556;OP331335.1:188730-188548;OP382479.1:188711-188529;OP422341.1:188726-188544;OP535324.1:188774-188592;OP535333.1:188829-188647;OP536770.1:188737-188555;OP597769.1:188719-188537;OP604566.1:188743-188561;OP612682.1:188644-188462;OP612684.1:188884-188702;OP612686.1:188697-188515;OP696841.1:185957-185775;OP879722.1:187897-187715;OQ419007.1:187954-187772;OQ419012.1:187928-187746;OQ419031.1:187932-187750;OQ419047.1:187934-187752;OQ451898.1:187768-187586;OQ451900.1:188682-188500;OQ581853.1:188680-188498;OQ581855.1:188789-188607;OQ721951.1:188758-188576;OQ721954.1:188690-188508;OR035652.1:189070-188888;OR035664.1:189016-188834;OR035702.1:189033-188851;OR146272.1:188672-188490;OR146330.1:188750-188568;OR264364.1:188727-188545;OR427332.1:188727-188545;OR459777.1:188713-188531;OR459797.1:188725-188543;PP098589.2:188736-188554;PP496048.1:188654-188472;PP776553.1:188606-188424;PP852971.1:188725-188543;PP852983.1:188731-188549;PP853000.1:188616-188434;PP853005.1:188690-188508;PP853007.1:188673-188491;PP853013.1:188670-188488;PP853027.1:188711-188529;PP853045.1:188476-188294;PP853048.1:188671-188489;PP860026.1:188674-188492;PP860027.1:188654-188472;PP860028.1:188611-188429;PP860031.1:188652-188470;PP860033.1:188647-188465;PQ153226.1:188717-188535;PQ340477.1:188807-188625;PQ340478.1:188716-188534;PQ340480.1:188838-188656;PQ340483.1:188794-188612;PQ340485.1:188833-188651;PQ340486.1:188892-188710;PQ340487.1:188936-188754;PQ340489.1:188868-188686;PQ340490.1:188726-188544;PQ340491.1:188969-188787;PQ340493.1:188788-188606;PQ340495.1:188849-188667;PQ340496.1:188947-188765;PQ368489.1:188750-188568;PQ451835.1:188911-188729;PQ451836.1:188689-188507;PQ451838.1:188894-188712;PQ451841.1:188805-188623 |
| OG0000217 | AY603973.1:135522-135686;AY741551.1:135546-135710;AY753185.1:135765-135929;EPI_ISL_13331598:133590-133754;EPI_ISL_13363142:133577-133741;EPI_ISL_13374487:133594-133758;EPI_ISL_13502582:133589-133753;EPI_ISL_13508471:133672-133836;EPI_ISL_13607904:133568-133732;EPI_ISL_13624509:133512-133676;EPI_ISL_13889442:133689-133853;EPI_ISL_13953610:133495-133659;EPI_ISL_14181948:133718-133882;EPI_ISL_15263355:133571-133735;EPI_ISL_15269699:133540-133704;EPI_ISL_15293002:133809-133973;EPI_ISL_15641561:133526-133690;EPI_ISL_15831211:133579-133743;EPI_ISL_15992098:133603-133767;EPI_ISL_16233784:133592-133756;EPI_ISL_16946400:133581-133745;EPI_ISL_17048206:133228-133392;EPI_ISL_17703567:133593-133757;EPI_ISL_17718458:133578-133742;EPI_ISL_18213374:133567-133731;EPI_ISL_18360394:133226-133390;EPI_ISL_18557816:133568-133732;EPI_ISL_18642365:133568-133732;EPI_ISL_18659846:133541-133705;EPI_ISL_18744049:133580-133744;EPI_ISL_19022858:133565-133729;EPI_ISL_19028778:133482-133646;EPI_ISL_19151800:133568-133732;EPI_ISL_19159116:133568-133732;EPI_ISL_19359677:133565-133729;EPI_ISL_19446306:133570-133734;KJ642616.1:135223-135387;LC831698.1:133566-133730;MN648051.1:133792-133956;MT903338.1:133744-133908;MT903340.1:133578-133742;MT903341.1:125112-125276;MT903342.1:133596-133760;MT903347.1:135552-135716;ON568298.1:133719-133883;ON622713.1:133800-133964;ON622722.2:133571-133735;ON631963.1:133597-133761;ON649879.1:133403-133567;ON676707.1:133633-133797;ON755039.1:133225-133389;ON808413.1:133565-133729;ON880420.1:133798-133962;ON911481.2:133583-133747;ON918656.1:133605-133769;ON959133.1:133694-133858;ON959136.1:133695-133859;ON983168.1:132270-132434;OP019276.1:133565-133729;OP150924.1:133708-133872;OP160532.1:133531-133695;OP235282.1:133565-133729;OP289782.1:133568-133732;OP331335.1:133598-133762;OP382479.1:133575-133739;OP422341.1:133588-133752;OP434519.1:133564-133728;OP535324.1:133578-133742;OP535333.1:133685-133849;OP536770.1:133568-133732;OP597769.1:133602-133766;OP604566.1:133568-133732;OP612682.1:133503-133667;OP612684.1:133566-133730;OP612686.1:133556-133720;OP696841.1:130844-131008;OP879722.1:132794-132958;OQ419007.1:132797-132961;OQ419012.1:132768-132932;OQ419031.1:132772-132936;OQ419047.1:132774-132938;OQ451898.1:132655-132819;OQ451900.1:133568-133732;OQ581853.1:133594-133758;OQ581855.1:133675-133839;OQ721951.1:133595-133759;OQ721954.1:133565-133729;OR035652.1:133514-133678;OR035664.1:133518-133682;OR035702.1:133515-133679;OR146272.1:133534-133698;OR146330.1:133578-133742;OR264364.1:133589-133753;OR427332.1:133589-133753;OR459777.1:133580-133744;OR459797.1:133587-133751;PP098589.2:133567-133731;PP496048.1:133571-133735;PP776553.1:133491-133655;PP852971.1:133575-133739;PP852983.1:133583-133747;PP853000.1:133540-133704;PP853005.1:133548-133712;PP853007.1:133585-133749;PP853013.1:133566-133730;PP853027.1:133596-133760;PP853045.1:133568-133732;PP853048.1:133584-133748;PP860026.1:133583-133747;PP860027.1:133637-133801;PP860028.1:133540-133704;PP860031.1:133631-133795;PP860033.1:133626-133790;PQ153226.1:133565-133729;PQ340477.1:133552-133716;PQ340478.1:133534-133698;PQ340480.1:133615-133779;PQ340483.1:133598-133762;PQ340485.1:133581-133745;PQ340486.1:133599-133763;PQ340487.1:133623-133787;PQ340489.1:133598-133762;PQ340490.1:133550-133714;PQ340491.1:133605-133769;PQ340493.1:133589-133753;PQ340495.1:133615-133779;PQ340496.1:133650-133814;PQ368489.1:133581-133745;PQ451835.1:133655-133819;PQ451836.1:133593-133757;PQ451838.1:133646-133810;PQ451841.1:133606-133770;PQ451842.1:133552-133716 |
| OG0000218 | AY603973.1:154201-154410;AY741551.1:154275-154484;AY753185.1:154456-154665;EPI_ISL_13331598:152320-152529;EPI_ISL_13363142:152308-152517;EPI_ISL_13374487:152341-152550;EPI_ISL_13502582:152336-152545;EPI_ISL_13508471:152402-152611;EPI_ISL_13607904:152307-152516;EPI_ISL_13624509:152242-152451;EPI_ISL_13889442:152435-152644;EPI_ISL_13953610:152235-152444;EPI_ISL_14181948:152465-152674;EPI_ISL_15263355:152301-152510;EPI_ISL_15269699:152270-152479;EPI_ISL_15293002:152539-152748;EPI_ISL_15641561:152256-152465;EPI_ISL_15831211:152310-152519;EPI_ISL_15992098:152350-152559;EPI_ISL_16233784:152322-152531;EPI_ISL_16946400:152312-152521;EPI_ISL_17048206:151976-152185;EPI_ISL_17703567:152323-152532;EPI_ISL_17718458:152303-152512;EPI_ISL_18213374:152314-152523;EPI_ISL_18360394:151621-151830;EPI_ISL_18557816:152317-152526;EPI_ISL_18642365:152312-152521;EPI_ISL_18659846:152271-152480;EPI_ISL_18744049:152320-152529;EPI_ISL_19022858:152277-152486;EPI_ISL_19028778:152229-152438;EPI_ISL_19151800:152313-152522;EPI_ISL_19159116:152298-152507;EPI_ISL_19359677:152313-152522;EPI_ISL_19446306:152300-152509;KJ642616.1:153923-154132;LC831698.1:152296-152505;MN648051.1:152539-152748;MT903338.1:152472-152681;MT903340.1:152325-152534;MT903341.1:143866-144075;MT903342.1:152362-152571;MT903347.1:154268-154477;ON568298.1:152466-152675;ON622713.1:152530-152739;ON622722.2:152318-152527;ON631963.1:152327-152536;ON649879.1:152133-152342;ON676707.1:152364-152573;ON755039.1:151955-152164;ON808413.1:152292-152501;ON880420.1:152528-152737;ON911481.2:152313-152522;ON918656.1:152335-152544;ON959133.1:152424-152633;ON959136.1:152425-152634;ON983168.1:151001-151210;OP019276.1:152312-152521;OP150924.1:152438-152647;OP160532.1:152261-152470;OP235282.1:152312-152521;OP289782.1:152316-152525;OP331335.1:152321-152530;OP382479.1:152305-152514;OP422341.1:152318-152527;OP434519.1:152294-152503;OP535324.1:152325-152534;OP535333.1:152432-152641;OP536770.1:152315-152524;OP597769.1:152349-152558;OP604566.1:152315-152524;OP612682.1:152250-152459;OP612684.1:152301-152510;OP612686.1:152303-152512;OP696841.1:149591-149800;OP879722.1:151538-151747;OQ419007.1:151532-151741;OQ419012.1:151506-151715;OQ419031.1:151510-151719;OQ419047.1:151512-151721;OQ451898.1:151402-151611;OQ451900.1:152316-152525;OQ581853.1:152324-152533;OQ581855.1:152423-152632;OQ721951.1:152342-152551;OQ721954.1:152315-152524;OR035652.1:152244-152453;OR035664.1:152248-152457;OR035702.1:152245-152454;OR146272.1:152264-152473;OR146330.1:152325-152534;OR264364.1:152319-152528;OR427332.1:152319-152528;OR459777.1:152308-152517;OR459797.1:152317-152526;PP098589.2:152314-152523;PP496048.1:152318-152527;PP776553.1:152238-152447;PP852971.1:152306-152515;PP852983.1:152323-152532;PP853000.1:152274-152483;PP853005.1:152282-152491;PP853007.1:152325-152534;PP853013.1:152297-152506;PP853027.1:152339-152548;PP853045.1:152290-152499;PP853048.1:152315-152524;PP860026.1:152310-152519;PP860027.1:152347-152556;PP860028.1:152273-152482;PP860031.1:152344-152553;PP860033.1:152330-152539;PQ153226.1:152295-152504;PQ340477.1:152282-152491;PQ340478.1:152264-152473;PQ340480.1:152342-152551;PQ340483.1:152328-152537;PQ340485.1:152311-152520;PQ340486.1:152329-152538;PQ340487.1:152353-152562;PQ340489.1:152328-152537;PQ340490.1:152280-152489;PQ340491.1:152335-152544;PQ340493.1:152319-152528;PQ340495.1:152345-152554;PQ340496.1:152380-152589;PQ368489.1:152328-152537;PQ451835.1:152385-152594;PQ451836.1:152323-152532;PQ451838.1:152373-152582;PQ451841.1:152336-152545;PQ451842.1:152282-152491 |
| OG0000219 | AY603973.1:135900-135568;AY741551.1:135924-135592;AY753185.1:136143-135811;EPI_ISL_13331598:133968-133636;EPI_ISL_13363142:133955-133623;EPI_ISL_13374487:133972-133640;EPI_ISL_13502582:133967-133635;EPI_ISL_13508471:134050-133718;EPI_ISL_13607904:133946-133614;EPI_ISL_13624509:133890-133558;EPI_ISL_13889442:134067-133735;EPI_ISL_13953610:133873-133541;EPI_ISL_14181948:134096-133764;EPI_ISL_15263355:133949-133617;EPI_ISL_15269699:133918-133586;EPI_ISL_15293002:134187-133855;EPI_ISL_15641561:133904-133572;EPI_ISL_15831211:133957-133625;EPI_ISL_15992098:133981-133649;EPI_ISL_16233784:133970-133638;EPI_ISL_16946400:133959-133627;EPI_ISL_17048206:133606-133274;EPI_ISL_17703567:133971-133639;EPI_ISL_17718458:133956-133624;EPI_ISL_18213374:133945-133613;EPI_ISL_18360394:133604-133272;EPI_ISL_18557816:133946-133614;EPI_ISL_18642365:133946-133614;EPI_ISL_18659846:133919-133587;EPI_ISL_18744049:133958-133626;EPI_ISL_19022858:133943-133611;EPI_ISL_19028778:133860-133528;EPI_ISL_19151800:133946-133614;EPI_ISL_19159116:133946-133614;EPI_ISL_19359677:133943-133611;EPI_ISL_19446306:133948-133616;KJ642616.1:135601-135269;LC831698.1:133944-133612;MN648051.1:134170-133838;MT903338.1:134122-133790;MT903340.1:133956-133624;MT903341.1:125490-125158;MT903342.1:133974-133642;MT903347.1:135930-135598;ON568298.1:134097-133765;ON622713.1:134178-133846;ON622722.2:133949-133617;ON631963.1:133975-133643;ON649879.1:133781-133449;ON676707.1:134011-133679;ON755039.1:133603-133271;ON808413.1:133943-133611;ON880420.1:134176-133844;ON911481.2:133961-133629;ON918656.1:133983-133651;ON959133.1:134072-133740;ON959136.1:134073-133741;ON983168.1:132648-132316;OP019276.1:133943-133611;OP150924.1:134086-133754;OP160532.1:133909-133577;OP235282.1:133943-133611;OP289782.1:133946-133614;OP331335.1:133976-133644;OP382479.1:133953-133621;OP422341.1:133966-133634;OP434519.1:133942-133610;OP535324.1:133956-133624;OP535333.1:134063-133731;OP536770.1:133946-133614;OP597769.1:133980-133648;OP604566.1:133946-133614;OP612682.1:133881-133549;OP612684.1:133944-133612;OP612686.1:133934-133602;OP696841.1:131222-130890;OP879722.1:133172-132840;OQ419007.1:133175-132843;OQ419012.1:133146-132814;OQ419031.1:133150-132818;OQ419047.1:133152-132820;OQ451898.1:133033-132701;OQ451900.1:133946-133614;OQ581853.1:133972-133640;OQ581855.1:134053-133721;OQ721951.1:133973-133641;OQ721954.1:133943-133611;OR035652.1:133892-133560;OR035664.1:133896-133564;OR035702.1:133893-133561;OR146272.1:133912-133580;OR146330.1:133956-133624;OR264364.1:133967-133635;OR427332.1:133967-133635;OR459777.1:133958-133626;OR459797.1:133965-133633;PP098589.2:133945-133613;PP496048.1:133949-133617;PP776553.1:133869-133537;PP852971.1:133953-133621;PP852983.1:133961-133629;PP853000.1:133918-133586;PP853005.1:133926-133594;PP853007.1:133963-133631;PP853013.1:133944-133612;PP853027.1:133974-133642;PP853045.1:133946-133614;PP853048.1:133962-133630;PP860026.1:133961-133629;PP860027.1:134015-133683;PP860028.1:133918-133586;PP860031.1:134009-133677;PP860033.1:134004-133672;PQ153226.1:133943-133611;PQ340477.1:133930-133598;PQ340478.1:133912-133580;PQ340480.1:133993-133661;PQ340483.1:133976-133644;PQ340485.1:133959-133627;PQ340486.1:133977-133645;PQ340487.1:134001-133669;PQ340489.1:133976-133644;PQ340490.1:133928-133596;PQ340491.1:133983-133651;PQ340493.1:133967-133635;PQ340495.1:133993-133661;PQ340496.1:134028-133696;PQ368489.1:133959-133627;PQ451835.1:134033-133701;PQ451836.1:133971-133639;PQ451838.1:134024-133692;PQ451841.1:133984-133652;PQ451842.1:133930-133598 |
| OG0000220 | AY603973.1:49905-49741;AY741551.1:49909-49745;AY753185.1:50148-49984;EPI_ISL_13331598:47976-47812;EPI_ISL_13363142:47969-47805;EPI_ISL_13374487:47984-47820;EPI_ISL_13502582:47969-47805;EPI_ISL_13508471:48061-47897;EPI_ISL_13607904:47960-47796;EPI_ISL_13624509:47904-47740;EPI_ISL_13889442:48079-47915;EPI_ISL_13953610:47920-47756;EPI_ISL_14181948:48105-47941;EPI_ISL_15263355:47960-47796;EPI_ISL_15269699:47929-47765;EPI_ISL_15293002:48202-48038;EPI_ISL_15641561:47918-47754;EPI_ISL_15831211:47967-47803;EPI_ISL_15992098:47982-47818;EPI_ISL_16233784:47978-47814;EPI_ISL_16946400:47967-47803;EPI_ISL_17048206:47617-47453;EPI_ISL_17703567:47982-47818;EPI_ISL_17718458:47960-47796;EPI_ISL_18213374:47957-47793;EPI_ISL_18360394:47618-47454;EPI_ISL_18557816:47958-47794;EPI_ISL_18642365:47960-47796;EPI_ISL_18659846:47930-47766;EPI_ISL_18744049:47962-47798;EPI_ISL_19022858:47957-47793;EPI_ISL_19028778:47874-47710;EPI_ISL_19151800:47958-47794;EPI_ISL_19159116:47960-47796;EPI_ISL_19359677:47957-47793;EPI_ISL_19446306:47952-47788;KJ642616.1:49599-49435;LC831698.1:47958-47794;MN648051.1:48180-48016;MT903338.1:48123-47959;MT903340.1:47960-47796;MT903341.1:39493-39329;MT903342.1:47975-47811;MT903347.1:49916-49752;ON568298.1:48106-47942;ON622713.1:48192-48028;ON622722.2:47963-47799;ON631963.1:47983-47819;ON649879.1:47793-47629;ON676707.1:48013-47849;ON755039.1:47623-47459;ON808413.1:47963-47799;ON880420.1:48190-48026;ON911481.2:47980-47816;ON918656.1:47995-47831;ON959133.1:48084-47920;ON959136.1:48085-47921;ON983168.1:46659-46495;OP019276.1:47957-47793;OP150924.1:48098-47934;OP160532.1:47923-47759;OP235282.1:47957-47793;OP289782.1:47960-47796;OP331335.1:47977-47813;OP382479.1:47964-47800;OP422341.1:47977-47813;OP434519.1:47956-47792;OP535324.1:47958-47794;OP535333.1:48067-47903;OP536770.1:47959-47795;OP597769.1:47981-47817;OP604566.1:47960-47796;OP612682.1:47886-47722;OP612684.1:47950-47786;OP612686.1:47950-47786;OP696841.1:45234-45070;OP879722.1:47183-47019;OQ419007.1:47193-47029;OQ419012.1:47182-47018;OQ419031.1:47164-47000;OQ419047.1:47193-47029;OQ451898.1:47045-46881;OQ451900.1:47958-47794;OQ581853.1:47984-47820;OQ581855.1:48065-47901;OQ721951.1:47998-47834;OQ721954.1:47955-47791;OR035652.1:47907-47743;OR035664.1:47907-47743;OR035702.1:47907-47743;OR146272.1:47923-47759;OR146330.1:47968-47804;OR264364.1:47978-47814;OR427332.1:47978-47814;OR459777.1:47962-47798;OR459797.1:47977-47813;PP098589.2:47959-47795;PP496048.1:47963-47799;PP776553.1:47884-47720;PP852971.1:47957-47793;PP852983.1:47963-47799;PP853000.1:47924-47760;PP853005.1:47932-47768;PP853007.1:47965-47801;PP853013.1:47938-47774;PP853027.1:47969-47805;PP853045.1:47950-47786;PP853048.1:47969-47805;PP860026.1:47967-47803;PP860027.1:47992-47828;PP860028.1:47933-47769;PP860031.1:47986-47822;PP860033.1:47991-47827;PQ153226.1:47957-47793;PQ340477.1:47940-47776;PQ340478.1:47925-47761;PQ340480.1:48004-47840;PQ340483.1:47988-47824;PQ340485.1:47974-47810;PQ340486.1:47989-47825;PQ340487.1:48014-47850;PQ340489.1:47989-47825;PQ340490.1:47940-47776;PQ340491.1:47995-47831;PQ340493.1:47974-47810;PQ340495.1:48004-47840;PQ340496.1:48037-47873;PQ368489.1:47960-47796;PQ451835.1:48045-47881;PQ451836.1:47977-47813;PQ451838.1:48028-47864;PQ451841.1:47988-47824;PQ451842.1:47940-47776 |
| OG0000221 | AY603973.1:159869-160207;AY741551.1:159942-160280;AY753185.1:160124-160462;EPI_ISL_13331598:158002-158178;EPI_ISL_13363142:157990-158166;EPI_ISL_13374487:158023-158199;EPI_ISL_13502582:158018-158194;EPI_ISL_13508471:158084-158260;EPI_ISL_13607904:157989-158165;EPI_ISL_13624509:157924-158100;EPI_ISL_13889442:158117-158293;EPI_ISL_13953610:157917-158093;EPI_ISL_14181948:158147-158323;EPI_ISL_15263355:157983-158159;EPI_ISL_15269699:157952-158128;EPI_ISL_15293002:158221-158397;EPI_ISL_15641561:157938-158114;EPI_ISL_15831211:157992-158168;EPI_ISL_15992098:158032-158208;EPI_ISL_16233784:158004-158180;EPI_ISL_16946400:157994-158170;EPI_ISL_17048206:157658-157834;EPI_ISL_17703567:158005-158181;EPI_ISL_17718458:157985-158161;EPI_ISL_18213374:157996-158172;EPI_ISL_18360394:157303-157479;EPI_ISL_18557816:157999-158175;EPI_ISL_18642365:157994-158170;EPI_ISL_18659846:157953-158129;EPI_ISL_18744049:158002-158178;EPI_ISL_19022858:157959-158135;EPI_ISL_19028778:157911-158087;EPI_ISL_19151800:157995-158171;EPI_ISL_19159116:157980-158156;EPI_ISL_19359677:157995-158171;EPI_ISL_19446306:157982-158158;KJ642616.1:159590-159928;LC831698.1:157978-158154;MN648051.1:158221-158397;MT903338.1:158154-158330;MT903340.1:158007-158183;MT903341.1:149548-149724;MT903342.1:158044-158220;MT903347.1:159938-160276;ON568298.1:158148-158324;ON622713.1:158212-158388;ON622722.2:158000-158176;ON631963.1:158009-158185;ON649879.1:157815-157991;ON676707.1:158046-158222;ON755039.1:157637-157813;ON808413.1:157974-158150;ON880420.1:158210-158386;ON911481.2:157995-158171;ON918656.1:158017-158193;ON959133.1:158106-158282;ON959136.1:158107-158283;ON983168.1:156683-156859;OP019276.1:157994-158170;OP150924.1:158120-158296;OP160532.1:157943-158119;OP235282.1:157994-158170;OP289782.1:157998-158174;OP331335.1:158003-158179;OP382479.1:157987-158163;OP422341.1:158000-158176;OP434519.1:157976-158152;OP535324.1:158007-158183;OP535333.1:158114-158290;OP536770.1:157997-158173;OP597769.1:158031-158207;OP604566.1:157997-158173;OP612682.1:157932-158108;OP612684.1:157983-158159;OP612686.1:157985-158161;OP696841.1:155273-155449;OP879722.1:157220-157396;OQ419007.1:157214-157390;OQ419012.1:157188-157364;OQ419031.1:157192-157368;OQ419047.1:157194-157370;OQ451898.1:157084-157260;OQ451900.1:157998-158174;OQ581853.1:158006-158182;OQ581855.1:158105-158281;OQ721951.1:158024-158200;OQ721954.1:157997-158173;OR035652.1:157926-158102;OR035664.1:157930-158106;OR035702.1:157927-158103;OR146272.1:157946-158122;OR146330.1:158007-158183;OR264364.1:158001-158177;OR427332.1:158001-158177;OR459777.1:157990-158166;OR459797.1:157999-158175;PP098589.2:157996-158172;PP496048.1:158000-158176;PP776553.1:157920-158096;PP852971.1:157988-158164;PP852983.1:158005-158181;PP853000.1:157956-158132;PP853005.1:157964-158140;PP853007.1:158007-158183;PP853013.1:157979-158155;PP853027.1:158021-158197;PP853045.1:157972-158148;PP853048.1:157997-158173;PP860026.1:157993-158334;PP860027.1:158029-158370;PP860028.1:157956-158297;PP860033.1:158012-158353;PQ153226.1:157977-158153;PQ340477.1:157964-158140;PQ340478.1:157946-158122;PQ340480.1:158024-158200;PQ340483.1:158010-158186;PQ340485.1:157993-158169;PQ340486.1:158011-158187;PQ340487.1:158035-158211;PQ340489.1:158010-158186;PQ340490.1:157962-158138;PQ340491.1:158017-158193;PQ340493.1:158001-158177;PQ340495.1:158027-158203;PQ340496.1:158062-158238;PQ368489.1:158010-158186;PQ451835.1:158067-158243;PQ451836.1:158005-158181;PQ451838.1:158055-158231;PQ451841.1:158018-158194;PQ451842.1:157964-158140 |
| OG0000222 | AY741551.1:135391-135573;EPI_ISL_13331598:133435-133617;EPI_ISL_13363142:133422-133604;EPI_ISL_13374487:133439-133621;EPI_ISL_13502582:133434-133616;EPI_ISL_13508471:133517-133699;EPI_ISL_13607904:133413-133595;EPI_ISL_13624509:133357-133539;EPI_ISL_13889442:133534-133716;EPI_ISL_13953610:133340-133522;EPI_ISL_14181948:133563-133745;EPI_ISL_15263355:133416-133598;EPI_ISL_15269699:133385-133567;EPI_ISL_15293002:133654-133836;EPI_ISL_15641561:133371-133553;EPI_ISL_15831211:133424-133606;EPI_ISL_15992098:133448-133630;EPI_ISL_16233784:133437-133619;EPI_ISL_16946400:133426-133608;EPI_ISL_17048206:133073-133255;EPI_ISL_17703567:133438-133620;EPI_ISL_17718458:133423-133605;EPI_ISL_18213374:133412-133594;EPI_ISL_18360394:133071-133253;EPI_ISL_18557816:133413-133595;EPI_ISL_18642365:133413-133595;EPI_ISL_18659846:133386-133568;EPI_ISL_18744049:133425-133607;EPI_ISL_19022858:133410-133592;EPI_ISL_19028778:133327-133509;EPI_ISL_19151800:133413-133595;EPI_ISL_19159116:133413-133595;EPI_ISL_19359677:133410-133592;EPI_ISL_19446306:133415-133597;KJ642616.1:135068-135250;LC831698.1:133411-133593;MN648051.1:133637-133819;MT903338.1:133589-133771;MT903340.1:133423-133605;MT903341.1:124957-125139;MT903342.1:133441-133623;MT903347.1:135397-135579;ON568298.1:133564-133746;ON622713.1:133645-133827;ON622722.2:133416-133598;ON631963.1:133442-133624;ON649879.1:133248-133430;ON676707.1:133478-133660;ON755039.1:133070-133252;ON808413.1:133410-133592;ON880420.1:133643-133825;ON911481.2:133428-133610;ON918656.1:133450-133632;ON959133.1:133539-133721;ON959136.1:133540-133722;ON983168.1:132115-132297;OP019276.1:133410-133592;OP150924.1:133553-133735;OP160532.1:133376-133558;OP235282.1:133410-133592;OP289782.1:133413-133595;OP331335.1:133443-133625;OP382479.1:133420-133602;OP422341.1:133433-133615;OP434519.1:133409-133591;OP535324.1:133423-133605;OP535333.1:133530-133712;OP536770.1:133413-133595;OP597769.1:133447-133629;OP604566.1:133413-133595;OP612682.1:133348-133530;OP612684.1:133411-133593;OP612686.1:133401-133583;OP696841.1:130689-130871;OP879722.1:132639-132821;OQ419007.1:132642-132824;OQ419012.1:132613-132795;OQ419031.1:132617-132799;OQ419047.1:132619-132801;OQ451898.1:132500-132682;OQ451900.1:133413-133595;OQ581853.1:133439-133621;OQ581855.1:133520-133702;OQ721951.1:133440-133622;OQ721954.1:133410-133592;OR035652.1:133359-133541;OR035664.1:133363-133545;OR035702.1:133360-133542;OR146272.1:133379-133561;OR146330.1:133423-133605;OR264364.1:133434-133616;OR427332.1:133434-133616;OR459777.1:133425-133607;OR459797.1:133432-133614;PP098589.2:133412-133594;PP496048.1:133416-133598;PP776553.1:133336-133518;PP852971.1:133420-133602;PP852983.1:133428-133610;PP853000.1:133385-133567;PP853005.1:133393-133575;PP853007.1:133430-133612;PP853013.1:133411-133593;PP853027.1:133441-133623;PP853045.1:133413-133595;PP853048.1:133429-133611;PP860026.1:133428-133610;PP860027.1:133482-133664;PP860028.1:133385-133567;PP860031.1:133476-133658;PP860033.1:133471-133653;PQ153226.1:133410-133592;PQ340477.1:133397-133579;PQ340478.1:133379-133561;PQ340480.1:133460-133642;PQ340483.1:133443-133625;PQ340485.1:133426-133608;PQ340486.1:133444-133626;PQ340487.1:133468-133650;PQ340489.1:133443-133625;PQ340490.1:133395-133577;PQ340491.1:133450-133632;PQ340493.1:133434-133616;PQ340495.1:133460-133642;PQ340496.1:133495-133677;PQ368489.1:133426-133608;PQ451835.1:133500-133682;PQ451836.1:133438-133620;PQ451838.1:133491-133673;PQ451841.1:133451-133633;PQ451842.1:133397-133579 |
| OG0000223 | EPI_ISL_13331598:30998-31237;EPI_ISL_13363142:30991-31230;EPI_ISL_13374487:31006-31245;EPI_ISL_13502582:30991-31230;EPI_ISL_13508471:31083-31322;EPI_ISL_13607904:30982-31221;EPI_ISL_13624509:30926-31165;EPI_ISL_13889442:31101-31340;EPI_ISL_13953610:30949-31188;EPI_ISL_14181948:31127-31366;EPI_ISL_15263355:30982-31221;EPI_ISL_15269699:30951-31190;EPI_ISL_15293002:31224-31463;EPI_ISL_15641561:30940-31179;EPI_ISL_15831211:30989-31228;EPI_ISL_15992098:31004-31243;EPI_ISL_16233784:31000-31239;EPI_ISL_16946400:30989-31228;EPI_ISL_17048206:30639-30878;EPI_ISL_17703567:31004-31243;EPI_ISL_17718458:30982-31221;EPI_ISL_18213374:30979-31218;EPI_ISL_18360394:30640-30879;EPI_ISL_18557816:30980-31219;EPI_ISL_18642365:30982-31221;EPI_ISL_18659846:30952-31191;EPI_ISL_18744049:30984-31223;EPI_ISL_19022858:30979-31218;EPI_ISL_19028778:30896-31135;EPI_ISL_19151800:30980-31219;EPI_ISL_19159116:30982-31221;EPI_ISL_19359677:30979-31218;EPI_ISL_19446306:30974-31213;LC831698.1:30980-31219;MN648051.1:31202-31441;MT903338.1:31145-31384;MT903340.1:30982-31221;MT903341.1:22515-22754;MT903342.1:30997-31236;ON568298.1:31128-31367;ON622713.1:31214-31453;ON622722.2:30985-31224;ON631963.1:31005-31244;ON649879.1:30815-31054;ON676707.1:31035-31274;ON755039.1:30645-30884;ON808413.1:30985-31224;ON880420.1:31212-31451;ON911481.2:31002-31241;ON918656.1:31017-31256;ON959133.1:31106-31345;ON959136.1:31107-31346;ON983168.1:29681-29920;OP019276.1:30979-31218;OP150924.1:31120-31359;OP160532.1:30945-31184;OP235282.1:30979-31218;OP289782.1:30982-31221;OP331335.1:30999-31238;OP382479.1:30986-31225;OP422341.1:30999-31238;OP434519.1:30978-31217;OP535324.1:30980-31219;OP535333.1:31089-31328;OP536770.1:30981-31220;OP597769.1:31003-31242;OP604566.1:30982-31221;OP612682.1:30908-31147;OP612684.1:30972-31211;OP612686.1:30972-31211;OP696841.1:28256-28495;OP879722.1:30205-30444;OQ419007.1:30215-30454;OQ419012.1:30207-30446;OQ419031.1:30186-30425;OQ419047.1:30215-30454;OQ451898.1:30067-30306;OQ451900.1:30980-31219;OQ581853.1:31006-31245;OQ581855.1:31087-31326;OQ721951.1:31020-31259;OQ721954.1:30977-31216;OR035652.1:30929-31168;OR035664.1:30929-31168;OR035702.1:30929-31168;OR146272.1:30945-31184;OR146330.1:30990-31229;OR264364.1:31000-31239;OR427332.1:31000-31239;OR459777.1:30984-31223;OR459797.1:30999-31238;PP098589.2:30981-31220;PP496048.1:30985-31224;PP776553.1:30906-31145;PP852971.1:30979-31218;PP852983.1:30984-31223;PP853000.1:30946-31185;PP853005.1:30954-31193;PP853007.1:30986-31225;PP853013.1:30960-31199;PP853027.1:30991-31230;PP853045.1:30972-31211;PP853048.1:30991-31230;PP860026.1:30992-31231;PP860027.1:31017-31256;PP860028.1:30958-31197;PP860031.1:31012-31251;PP860033.1:31016-31255;PQ153226.1:30979-31218;PQ340477.1:30962-31201;PQ340478.1:30947-31186;PQ340480.1:31026-31265;PQ340483.1:31010-31249;PQ340485.1:30996-31235;PQ340486.1:31011-31250;PQ340487.1:31036-31275;PQ340489.1:31011-31250;PQ340490.1:30962-31201;PQ340491.1:31020-31259;PQ340493.1:30996-31235;PQ340495.1:31026-31265;PQ340496.1:31059-31298;PQ368489.1:30982-31221;PQ451835.1:31070-31309;PQ451836.1:31002-31241;PQ451838.1:31053-31292;PQ451841.1:31013-31252;PQ451842.1:30965-31204 |
| OG0000224 | EPI_ISL_13331598:173312-173539;EPI_ISL_13363142:173350-173577;EPI_ISL_13374487:173407-173634;EPI_ISL_13502582:173399-173626;EPI_ISL_13508471:173396-173623;EPI_ISL_13607904:173302-173529;EPI_ISL_13624509:173178-173459;EPI_ISL_13889442:173371-173718;EPI_ISL_13953610:173172-173453;EPI_ISL_14181948:173459-173686;EPI_ISL_15263355:173206-173424;EPI_ISL_15269699:173264-173491;EPI_ISL_15293002:173475-173762;EPI_ISL_15641561:172704-172985;EPI_ISL_15831211:173376-173603;EPI_ISL_15992098:173286-173603;EPI_ISL_16233784:173316-173543;EPI_ISL_16946400:173300-173527;EPI_ISL_17048206:172912-173229;EPI_ISL_17703567:173317-173544;EPI_ISL_17718458:173228-173509;EPI_ISL_18213374:173250-173531;EPI_ISL_18360394:172580-172807;EPI_ISL_18557816:173323-173550;EPI_ISL_18642365:173310-173537;EPI_ISL_18659846:173265-173492;EPI_ISL_18744049:173257-173538;EPI_ISL_19022858:173213-173494;EPI_ISL_19028778:173165-173446;EPI_ISL_19151800:172230-172457;EPI_ISL_19159116:173234-173515;EPI_ISL_19359677:173249-173530;EPI_ISL_19446306:173236-173517;LC831698.1:173232-173513;MN648051.1:173525-173752;MT903338.1:173408-173725;MT903340.1:173261-173542;MT903341.1:164878-165105;MT903342.1:173298-173645;ON568298.1:173460-173687;ON622713.1:173466-173759;ON622722.2:173254-173547;ON631963.1:173319-173546;ON649879.1:173125-173352;ON676707.1:173300-173617;ON755039.1:172941-173168;ON808413.1:173228-173515;ON880420.1:173522-173749;ON911481.2:173249-173539;ON918656.1:173329-173556;ON959133.1:173360-173647;ON959136.1:173361-173642;ON983168.1:171995-172222;OP019276.1:173248-173529;OP150924.1:173436-173663;OP160532.1:173197-173478;OP235282.1:173248-173529;OP289782.1:173252-173533;OP331335.1:173316-173543;OP382479.1:173297-173524;OP422341.1:173312-173539;OP434519.1:173230-173511;OP535324.1:173342-173569;OP535333.1:173424-173651;OP536770.1:173251-173532;OP597769.1:173385-173612;OP604566.1:173251-173532;OP612682.1:173212-173439;OP612684.1:173263-173490;OP612686.1:173265-173492;OP696841.1:170597-170824;OP879722.1:172474-172791;OQ419007.1:172468-172749;OQ419012.1:172442-172723;OQ419031.1:172446-172727;OQ419047.1:172448-172729;OQ451898.1:172408-172635;OQ451900.1:173322-173549;OQ581853.1:173260-173547;OQ581855.1:173429-173656;OQ721951.1:173278-173535;OQ721954.1:173251-173568;OR035652.1:173238-173465;OR035664.1:173242-173469;OR035702.1:173181-173468;OR146272.1:173258-173485;OR146330.1:173391-173618;OR264364.1:173313-173540;OR427332.1:173313-173540;OR459777.1:173233-173514;OR459797.1:173311-173538;PP098589.2:173250-173531;PP496048.1:173254-173547;PP776553.1:173174-173473;PP852971.1:173239-173520;PP852983.1:173317-173544;PP853000.1:173256-173483;PP853005.1:173276-173503;PP853007.1:173313-173540;PP853013.1:173310-173537;PP853027.1:173351-173578;PP853045.1:173291-173518;PP853048.1:173251-173538;PP860026.1:173318-173545;PP860027.1:173267-173530;PP860028.1:173324-173482;PP860031.1:173366-173524;PP860033.1:173296-173523;PQ153226.1:173231-173512;PQ340477.1:173276-173503;PQ340478.1:173200-173493;PQ340480.1:173334-173561;PQ340483.1:173326-173553;PQ340485.1:173311-173538;PQ340486.1:173265-173552;PQ340487.1:173345-173572;PQ340489.1:173328-173555;PQ340490.1:173216-173503;PQ340491.1:173339-173566;PQ340493.1:173311-173538;PQ340495.1:173345-173572;PQ340496.1:173380-173607;PQ368489.1:173264-173545;PQ451835.1:173389-173616;PQ451836.1:173329-173556;PQ451838.1:173309-173608;PQ451841.1:173346-173573;PQ451842.1:173282-173509 |
| OG0000225 | EPI_ISL_13331598:178392-178153;EPI_ISL_13363142:178430-178191;EPI_ISL_13374487:178487-178248;EPI_ISL_13502582:178479-178240;EPI_ISL_13508471:178476-178237;EPI_ISL_13607904:178382-178143;EPI_ISL_13624509:178312-178073;EPI_ISL_13889442:178571-178332;EPI_ISL_13953610:178306-178067;EPI_ISL_14181948:178539-178300;EPI_ISL_15263355:178277-178038;EPI_ISL_15269699:178344-178105;EPI_ISL_15293002:178615-178376;EPI_ISL_15641561:177838-177599;EPI_ISL_15831211:178450-178211;EPI_ISL_15992098:178456-178217;EPI_ISL_16233784:178396-178157;EPI_ISL_16946400:178374-178135;EPI_ISL_17048206:178082-177843;EPI_ISL_17703567:178397-178158;EPI_ISL_17718458:178356-178117;EPI_ISL_18213374:178384-178145;EPI_ISL_18360394:177660-177421;EPI_ISL_18557816:178403-178164;EPI_ISL_18642365:178390-178151;EPI_ISL_18659846:178345-178106;EPI_ISL_18744049:178385-178146;EPI_ISL_19022858:178347-178108;EPI_ISL_19028778:178299-178060;EPI_ISL_19151800:177310-177071;EPI_ISL_19159116:178368-178129;EPI_ISL_19359677:178383-178144;EPI_ISL_19446306:178370-178131;LC831698.1:178366-178127;MN648051.1:178605-178366;MT903338.1:178578-178339;MT903340.1:178395-178156;MT903341.1:169958-169719;MT903342.1:178498-178259;ON568298.1:178540-178301;ON622713.1:178612-178373;ON622722.2:178400-178161;ON631963.1:178399-178160;ON649879.1:178205-177966;ON676707.1:178470-178231;ON755039.1:178021-177782;ON808413.1:178368-178129;ON880420.1:178602-178363;ON911481.2:178392-178153;ON918656.1:178409-178170;ON959133.1:178500-178261;ON959136.1:178495-178256;ON983168.1:177075-176836;OP019276.1:178382-178143;OP150924.1:178516-178277;OP160532.1:178331-178092;OP235282.1:178382-178143;OP289782.1:178386-178147;OP331335.1:178396-178157;OP382479.1:178377-178138;OP422341.1:178392-178153;OP434519.1:178364-178125;OP535324.1:178422-178183;OP535333.1:178504-178265;OP536770.1:178385-178146;OP597769.1:178465-178226;OP604566.1:178391-178152;OP612682.1:178292-178053;OP612684.1:178343-178104;OP612686.1:178345-178106;OP696841.1:175677-175438;OP879722.1:177644-177405;OQ419007.1:177602-177363;OQ419012.1:177576-177337;OQ419031.1:177580-177341;OQ419047.1:177582-177343;OQ451898.1:177488-177249;OQ451900.1:178402-178163;OQ581853.1:178400-178161;OQ581855.1:178509-178270;OQ721951.1:178388-178149;OQ721954.1:178421-178182;OR035652.1:178318-178079;OR035664.1:178322-178083;OR035702.1:178321-178082;OR146272.1:178338-178099;OR146330.1:178471-178232;OR264364.1:178393-178154;OR427332.1:178393-178154;OR459777.1:178361-178122;OR459797.1:178391-178152;PP098589.2:178384-178145;PP496048.1:178400-178161;PP776553.1:178326-178087;PP852971.1:178373-178134;PP852983.1:178397-178158;PP853000.1:178336-178097;PP853005.1:178356-178117;PP853007.1:178393-178154;PP853013.1:178390-178151;PP853027.1:178431-178192;PP853045.1:178196-177957;PP853048.1:178391-178152;PP860026.1:178394-178155;PP860027.1:178375-178136;PP860028.1:178330-178091;PP860031.1:178371-178132;PP860033.1:178368-178129;PQ153226.1:178365-178126;PQ340477.1:178356-178117;PQ340478.1:178346-178107;PQ340480.1:178414-178175;PQ340483.1:178406-178167;PQ340485.1:178391-178152;PQ340486.1:178405-178166;PQ340487.1:178431-178192;PQ340489.1:178408-178169;PQ340490.1:178356-178117;PQ340491.1:178419-178180;PQ340493.1:178391-178152;PQ340495.1:178425-178186;PQ340496.1:178460-178221;PQ368489.1:178398-178159;PQ451835.1:178469-178230;PQ451836.1:178409-178170;PQ451838.1:178461-178222;PQ451841.1:178426-178187;PQ451842.1:178362-178123 |
| OG0000226 | EPI_ISL_13331598:157760-157545;EPI_ISL_13363142:157748-157533;EPI_ISL_13374487:157781-157566;EPI_ISL_13502582:157776-157561;EPI_ISL_13508471:157842-157627;EPI_ISL_13607904:157747-157532;EPI_ISL_13624509:157682-157467;EPI_ISL_13889442:157875-157660;EPI_ISL_13953610:157675-157460;EPI_ISL_14181948:157905-157690;EPI_ISL_15263355:157741-157526;EPI_ISL_15269699:157710-157495;EPI_ISL_15293002:157979-157764;EPI_ISL_15641561:157696-157481;EPI_ISL_15831211:157750-157535;EPI_ISL_15992098:157790-157575;EPI_ISL_16233784:157762-157547;EPI_ISL_16946400:157752-157537;EPI_ISL_17048206:157416-157201;EPI_ISL_17703567:157763-157548;EPI_ISL_17718458:157743-157528;EPI_ISL_18213374:157754-157539;EPI_ISL_18360394:157061-156846;EPI_ISL_18557816:157757-157542;EPI_ISL_18642365:157752-157537;EPI_ISL_18659846:157711-157496;EPI_ISL_18744049:157760-157545;EPI_ISL_19022858:157717-157502;EPI_ISL_19028778:157669-157454;EPI_ISL_19151800:157753-157538;EPI_ISL_19159116:157738-157523;EPI_ISL_19359677:157753-157538;EPI_ISL_19446306:157740-157525;LC831698.1:157736-157521;MN648051.1:157979-157764;MT903338.1:157912-157697;MT903340.1:157765-157550;MT903341.1:149306-149091;MT903342.1:157802-157587;ON568298.1:157906-157691;ON622713.1:157970-157755;ON622722.2:157758-157543;ON631963.1:157767-157552;ON649879.1:157573-157358;ON676707.1:157804-157589;ON755039.1:157395-157180;ON808413.1:157732-157517;ON880420.1:157968-157753;ON911481.2:157753-157538;ON918656.1:157775-157560;ON959133.1:157864-157649;ON959136.1:157865-157650;ON983168.1:156441-156226;OP019276.1:157752-157537;OP150924.1:157878-157663;OP160532.1:157701-157486;OP235282.1:157752-157537;OP289782.1:157756-157541;OP331335.1:157761-157546;OP382479.1:157745-157530;OP422341.1:157758-157543;OP434519.1:157734-157519;OP535324.1:157765-157550;OP535333.1:157872-157657;OP536770.1:157755-157540;OP597769.1:157789-157574;OP604566.1:157755-157540;OP612682.1:157690-157475;OP612684.1:157741-157526;OP612686.1:157743-157528;OP696841.1:155031-154816;OP879722.1:156978-156763;OQ419007.1:156972-156757;OQ419012.1:156946-156731;OQ419031.1:156950-156735;OQ419047.1:156952-156737;OQ451898.1:156842-156627;OQ451900.1:157756-157541;OQ581853.1:157764-157549;OQ581855.1:157863-157648;OQ721951.1:157782-157567;OQ721954.1:157755-157540;OR035652.1:157684-157469;OR035664.1:157688-157473;OR035702.1:157685-157470;OR146272.1:157704-157489;OR146330.1:157765-157550;OR264364.1:157759-157544;OR427332.1:157759-157544;OR459777.1:157748-157533;OR459797.1:157757-157542;PP098589.2:157754-157539;PP496048.1:157758-157543;PP776553.1:157678-157463;PP852971.1:157746-157531;PP852983.1:157763-157548;PP853000.1:157714-157499;PP853005.1:157722-157507;PP853007.1:157765-157550;PP853013.1:157737-157522;PP853027.1:157779-157564;PP853045.1:157730-157515;PP853048.1:157755-157540;PP860026.1:157751-157536;PP860027.1:157787-157572;PP860028.1:157714-157499;PP860031.1:157784-157569;PP860033.1:157770-157555;PQ153226.1:157735-157520;PQ340477.1:157722-157507;PQ340478.1:157704-157489;PQ340480.1:157782-157567;PQ340483.1:157768-157553;PQ340485.1:157751-157536;PQ340486.1:157769-157554;PQ340487.1:157793-157578;PQ340489.1:157768-157553;PQ340490.1:157720-157505;PQ340491.1:157775-157560;PQ340493.1:157759-157544;PQ340495.1:157785-157570;PQ340496.1:157820-157605;PQ368489.1:157768-157553;PQ451835.1:157825-157610;PQ451836.1:157763-157548;PQ451838.1:157813-157598;PQ451841.1:157776-157561;PQ451842.1:157722-157507 |
| OG0000227 | EPI_ISL_13331598:156623-156417;EPI_ISL_13363142:156611-156405;EPI_ISL_13374487:156644-156438;EPI_ISL_13502582:156639-156433;EPI_ISL_13508471:156705-156499;EPI_ISL_13607904:156610-156404;EPI_ISL_13624509:156545-156339;EPI_ISL_13889442:156738-156532;EPI_ISL_13953610:156538-156332;EPI_ISL_14181948:156768-156562;EPI_ISL_15263355:156604-156398;EPI_ISL_15269699:156573-156367;EPI_ISL_15293002:156842-156636;EPI_ISL_15641561:156559-156353;EPI_ISL_15831211:156613-156407;EPI_ISL_15992098:156653-156447;EPI_ISL_16233784:156625-156419;EPI_ISL_16946400:156615-156409;EPI_ISL_17048206:156279-156073;EPI_ISL_17703567:156626-156420;EPI_ISL_17718458:156606-156400;EPI_ISL_18213374:156617-156411;EPI_ISL_18360394:155924-155718;EPI_ISL_18557816:156620-156414;EPI_ISL_18642365:156615-156409;EPI_ISL_18659846:156574-156368;EPI_ISL_18744049:156623-156417;EPI_ISL_19022858:156580-156374;EPI_ISL_19028778:156532-156326;EPI_ISL_19151800:156616-156410;EPI_ISL_19159116:156601-156395;EPI_ISL_19359677:156616-156410;EPI_ISL_19446306:156603-156397;LC831698.1:156599-156393;MN648051.1:156842-156636;MT903338.1:156775-156569;MT903340.1:156628-156422;MT903341.1:148169-147963;MT903342.1:156665-156459;ON568298.1:156769-156563;ON622713.1:156833-156627;ON622722.2:156621-156415;ON631963.1:156630-156424;ON649879.1:156436-156230;ON676707.1:156667-156461;ON755039.1:156258-156052;ON808413.1:156595-156389;ON880420.1:156831-156625;ON911481.2:156616-156410;ON918656.1:156638-156432;ON959133.1:156727-156521;ON959136.1:156728-156522;ON983168.1:155304-155098;OP019276.1:156615-156409;OP150924.1:156741-156535;OP160532.1:156564-156358;OP235282.1:156615-156409;OP289782.1:156619-156413;OP331335.1:156624-156418;OP382479.1:156608-156402;OP422341.1:156621-156415;OP434519.1:156597-156391;OP535324.1:156628-156422;OP535333.1:156735-156529;OP536770.1:156618-156412;OP597769.1:156652-156446;OP604566.1:156618-156412;OP612682.1:156553-156347;OP612684.1:156604-156398;OP612686.1:156606-156400;OP696841.1:153894-153688;OP879722.1:155841-155635;OQ419007.1:155835-155629;OQ419012.1:155809-155603;OQ419031.1:155813-155607;OQ419047.1:155815-155609;OQ451898.1:155705-155499;OQ451900.1:156619-156413;OQ581853.1:156627-156421;OQ581855.1:156726-156520;OQ721951.1:156645-156439;OQ721954.1:156618-156412;OR035652.1:156547-156341;OR035664.1:156551-156345;OR035702.1:156548-156342;OR146272.1:156567-156361;OR146330.1:156628-156422;OR264364.1:156622-156416;OR427332.1:156622-156416;OR459777.1:156611-156405;OR459797.1:156620-156414;PP098589.2:156617-156411;PP496048.1:156621-156415;PP776553.1:156541-156335;PP852971.1:156609-156403;PP852983.1:156626-156420;PP853000.1:156577-156371;PP853005.1:156585-156379;PP853007.1:156628-156422;PP853013.1:156600-156394;PP853027.1:156642-156436;PP853045.1:156593-156387;PP853048.1:156618-156412;PP860026.1:156614-156408;PP860027.1:156650-156444;PP860028.1:156577-156371;PP860031.1:156647-156441;PP860033.1:156633-156427;PQ153226.1:156598-156392;PQ340477.1:156585-156379;PQ340478.1:156567-156361;PQ340480.1:156645-156439;PQ340483.1:156631-156425;PQ340485.1:156614-156408;PQ340486.1:156632-156426;PQ340487.1:156656-156450;PQ340489.1:156631-156425;PQ340490.1:156583-156377;PQ340491.1:156638-156432;PQ340493.1:156622-156416;PQ340495.1:156648-156442;PQ340496.1:156683-156477;PQ368489.1:156631-156425;PQ451835.1:156688-156482;PQ451836.1:156626-156420;PQ451838.1:156676-156470;PQ451841.1:156639-156433;PQ451842.1:156585-156379 |
| OG0000228 | EPI_ISL_13331598:33057-33209;EPI_ISL_13363142:33050-33202;EPI_ISL_13374487:33065-33217;EPI_ISL_13502582:33050-33202;EPI_ISL_13508471:33142-33294;EPI_ISL_13607904:33041-33193;EPI_ISL_13624509:32985-33137;EPI_ISL_13889442:33160-33312;EPI_ISL_13953610:33008-33160;EPI_ISL_14181948:33186-33338;EPI_ISL_15263355:33041-33193;EPI_ISL_15269699:33010-33162;EPI_ISL_15293002:33283-33435;EPI_ISL_15641561:32999-33151;EPI_ISL_15831211:33048-33200;EPI_ISL_15992098:33063-33215;EPI_ISL_16233784:33059-33211;EPI_ISL_16946400:33048-33200;EPI_ISL_17048206:32698-32850;EPI_ISL_17703567:33063-33215;EPI_ISL_17718458:33041-33193;EPI_ISL_18213374:33038-33190;EPI_ISL_18360394:32699-32851;EPI_ISL_18557816:33039-33191;EPI_ISL_18642365:33041-33193;EPI_ISL_18659846:33011-33163;EPI_ISL_18744049:33043-33195;EPI_ISL_19022858:33038-33190;EPI_ISL_19028778:32955-33107;EPI_ISL_19151800:33039-33191;EPI_ISL_19159116:33041-33193;EPI_ISL_19359677:33038-33190;EPI_ISL_19446306:33033-33185;LC831698.1:33039-33191;MN648051.1:33261-33413;MT903338.1:33204-33356;MT903340.1:33041-33193;MT903341.1:24574-24726;MT903342.1:33056-33208;ON568298.1:33187-33339;ON622713.1:33273-33425;ON622722.2:33044-33196;ON631963.1:33064-33216;ON649879.1:32874-33026;ON676707.1:33094-33246;ON755039.1:32704-32856;ON808413.1:33044-33196;ON880420.1:33271-33423;ON911481.2:33061-33213;ON918656.1:33076-33228;ON959133.1:33165-33317;ON959136.1:33166-33318;ON983168.1:31740-31892;OP019276.1:33038-33190;OP150924.1:33179-33331;OP160532.1:33004-33156;OP235282.1:33038-33190;OP289782.1:33041-33193;OP331335.1:33058-33210;OP382479.1:33045-33197;OP422341.1:33058-33210;OP434519.1:33037-33189;OP535324.1:33039-33191;OP535333.1:33148-33300;OP536770.1:33040-33192;OP597769.1:33062-33214;OP604566.1:33041-33193;OP612682.1:32967-33119;OP612684.1:33031-33183;OP612686.1:33031-33183;OP696841.1:30315-30467;OP879722.1:32264-32416;OQ419007.1:32274-32426;OQ419012.1:32266-32418;OQ419031.1:32245-32397;OQ419047.1:32274-32426;OQ451898.1:32126-32278;OQ451900.1:33039-33191;OQ581853.1:33065-33217;OQ581855.1:33146-33298;OQ721951.1:33079-33231;OQ721954.1:33036-33188;OR035652.1:32988-33140;OR035664.1:32988-33140;OR035702.1:32988-33140;OR146272.1:33004-33156;OR146330.1:33049-33201;OR264364.1:33059-33211;OR427332.1:33059-33211;OR459777.1:33043-33195;OR459797.1:33058-33210;PP098589.2:33040-33192;PP496048.1:33044-33196;PP776553.1:32965-33117;PP852971.1:33038-33190;PP852983.1:33043-33195;PP853000.1:33005-33157;PP853005.1:33013-33165;PP853007.1:33045-33197;PP853013.1:33019-33171;PP853027.1:33050-33202;PP853045.1:33031-33183;PP853048.1:33050-33202;PP860026.1:33048-33200;PP860027.1:33073-33225;PP860028.1:33014-33166;PP860033.1:33072-33224;PQ153226.1:33038-33190;PQ340477.1:33021-33173;PQ340478.1:33006-33158;PQ340480.1:33085-33237;PQ340483.1:33069-33221;PQ340485.1:33055-33207;PQ340486.1:33070-33222;PQ340487.1:33095-33247;PQ340489.1:33070-33222;PQ340490.1:33021-33173;PQ340491.1:33079-33231;PQ340493.1:33055-33207;PQ340495.1:33085-33237;PQ340496.1:33118-33270;PQ368489.1:33041-33193;PQ451835.1:33129-33281;PQ451836.1:33061-33213;PQ451838.1:33112-33264;PQ451841.1:33072-33224;PQ451842.1:33024-33176 |
| OG0000229 | EPI_ISL_13331598:173621-173785;EPI_ISL_13363142:173659-173823;EPI_ISL_13374487:173716-173880;EPI_ISL_13502582:173708-173872;EPI_ISL_13508471:173705-173869;EPI_ISL_13607904:173611-173775;EPI_ISL_13624509:173541-173705;EPI_ISL_13889442:173800-173964;EPI_ISL_13953610:173535-173699;EPI_ISL_14181948:173768-173932;EPI_ISL_15263355:173506-173670;EPI_ISL_15269699:173573-173737;EPI_ISL_15293002:173844-174008;EPI_ISL_15641561:173067-173231;EPI_ISL_15831211:173685-173849;EPI_ISL_15992098:173685-173849;EPI_ISL_16233784:173625-173789;EPI_ISL_16946400:173609-173773;EPI_ISL_17048206:173311-173475;EPI_ISL_17703567:173626-173790;EPI_ISL_17718458:173591-173755;EPI_ISL_18213374:173613-173777;EPI_ISL_18360394:172889-173053;EPI_ISL_18557816:173632-173796;EPI_ISL_18642365:173619-173783;EPI_ISL_18659846:173574-173738;EPI_ISL_18744049:173620-173784;EPI_ISL_19022858:173576-173740;EPI_ISL_19028778:173528-173692;EPI_ISL_19151800:172539-172703;EPI_ISL_19159116:173597-173761;EPI_ISL_19359677:173612-173776;EPI_ISL_19446306:173599-173763;LC831698.1:173595-173759;MN648051.1:173834-173998;MT903338.1:173807-173971;MT903340.1:173624-173788;MT903341.1:165187-165351;MT903342.1:173727-173891;ON568298.1:173769-173933;ON622713.1:173841-174005;ON622722.2:173629-173793;ON631963.1:173628-173792;ON649879.1:173434-173598;ON676707.1:173699-173863;ON755039.1:173250-173414;ON808413.1:173597-173761;ON880420.1:173831-173995;ON911481.2:173621-173785;ON918656.1:173638-173802;ON959133.1:173729-173893;ON959136.1:173724-173888;ON983168.1:172304-172468;OP019276.1:173611-173775;OP150924.1:173745-173909;OP160532.1:173560-173724;OP235282.1:173611-173775;OP289782.1:173615-173779;OP331335.1:173625-173789;OP382479.1:173606-173770;OP422341.1:173621-173785;OP434519.1:173593-173757;OP535324.1:173651-173815;OP535333.1:173733-173897;OP536770.1:173614-173778;OP597769.1:173694-173858;OP604566.1:173614-173778;OP612682.1:173521-173685;OP612684.1:173572-173736;OP612686.1:173574-173738;OP696841.1:170906-171070;OP879722.1:172873-173037;OQ419007.1:172831-172995;OQ419012.1:172805-172969;OQ419031.1:172809-172973;OQ419047.1:172811-172975;OQ451898.1:172717-172881;OQ451900.1:173631-173795;OQ581853.1:173629-173793;OQ581855.1:173738-173902;OQ721951.1:173617-173781;OQ721954.1:173650-173814;OR035652.1:173547-173711;OR035664.1:173551-173715;OR035702.1:173550-173714;OR146272.1:173567-173731;OR146330.1:173700-173864;OR264364.1:173622-173786;OR427332.1:173622-173786;OR459777.1:173596-173760;OR459797.1:173620-173784;PP098589.2:173613-173777;PP496048.1:173629-173793;PP776553.1:173555-173719;PP852971.1:173602-173766;PP852983.1:173626-173790;PP853000.1:173565-173729;PP853005.1:173585-173749;PP853007.1:173622-173786;PP853013.1:173619-173783;PP853027.1:173660-173824;PP853045.1:173600-173764;PP853048.1:173620-173784;PP860026.1:173627-173791;PP860028.1:173564-173728;PP860031.1:173606-173770;PQ153226.1:173594-173758;PQ340477.1:173585-173749;PQ340478.1:173575-173739;PQ340480.1:173643-173807;PQ340483.1:173635-173799;PQ340485.1:173620-173784;PQ340486.1:173634-173798;PQ340487.1:173654-173818;PQ340489.1:173637-173801;PQ340490.1:173585-173749;PQ340491.1:173648-173812;PQ340493.1:173620-173784;PQ340495.1:173654-173818;PQ340496.1:173689-173853;PQ368489.1:173627-173791;PQ451835.1:173698-173862;PQ451836.1:173638-173802;PQ451838.1:173690-173854;PQ451841.1:173655-173819;PQ451842.1:173591-173755 |
| OG0000230 | EPI_ISL_13363142:150641-150489;EPI_ISL_13374487:150674-150483;EPI_ISL_13502582:150669-150478;EPI_ISL_13889442:150768-150601;EPI_ISL_13953610:150568-150377;EPI_ISL_14181948:150798-150607;EPI_ISL_15831211:150643-150491;EPI_ISL_15992098:150683-150492;EPI_ISL_16946400:150645-150493;EPI_ISL_17718458:150636-150445;EPI_ISL_18213374:150647-150456;EPI_ISL_18360394:149954-149763;EPI_ISL_18557816:150650-150480;EPI_ISL_18642365:150645-150454;EPI_ISL_18744049:150653-150462;EPI_ISL_19028778:150562-150371;MN648051.1:150872-150681;MT903338.1:150805-150614;MT903340.1:150658-150467;MT903341.1:142199-142008;MT903342.1:150695-150504;ON568298.1:150799-150608;ON622722.2:150651-150460;ON676707.1:150697-150506;OP019276.1:150645-150454;OP235282.1:150645-150454;OP535324.1:150658-150467;OP535333.1:150765-150574;OP536770.1:150648-150457;OP597769.1:150682-150491;OP604566.1:150648-150457;OP612682.1:150583-150392;OP612684.1:150634-150443;OP612686.1:150636-150445;OP696841.1:147924-147733;OP879722.1:149871-149680;OQ419007.1:149865-149683;OQ419012.1:149839-149657;OQ419031.1:149843-149661;OQ419047.1:149845-149663;OQ451898.1:149735-149544;OQ721951.1:150675-150484;OQ721954.1:150648-150457;OR146330.1:150658-150467;OR459777.1:150641-150450;PP098589.2:150647-150456;PP496048.1:150651-150460;PP776553.1:150571-150380;PP852971.1:150639-150448;PP852983.1:150656-150465;PP853000.1:150607-150416;PP853005.1:150615-150424;PP853007.1:150658-150467;PP853013.1:150630-150439;PP853027.1:150672-150472;PP853045.1:150623-150432;PP853048.1:150648-150457;PP860027.1:150680-150504;PP860031.1:150677-150486;PP860033.1:150663-150487;PQ221833.1:152576-152316;PQ368489.1:150661-150470 |
| OG0000231 | AY603973.1:135143-135298;AY741551.1:135166-135321;AY753185.1:135386-135541;DQ011154.1:135598-135753;DQ011155.1:135579-135734;EPI_ISL_14838587:135452-135607;EPI_ISL_19350788:134323-134478;EPI_ISL_19467692:135734-135889;JX878411.1:136443-136598;JX878425.1:135960-136115;JX878426.1:135410-135565;KC257460.1:135377-135532;KJ642612.1:133694-133849;KJ642613.1:135254-135409;KJ642616.1:134843-134998;KJ642618.1:132926-133081;KP849469.1:135768-135923;KP849471.1:135694-135849;MN702445.1:129111-129266;MN702446.1:135256-135411;MN702447.1:128624-128779;MN702449.1:129122-129277;MN702452.1:135297-135452;MT903347.1:135172-135327;OR943698.1:135510-135665;PP601206.1:135440-135595;PQ178862.2:134300-134455;PQ221506.1:129277-129432;PQ221833.1:135443-135598 |
| OG0000232 | AY603973.1:10770-10567;AY741551.1:10595-10392;AY753185.1:10981-10778;DQ011154.1:9174-8923;DQ011155.1:9079-8828;EPI_ISL_14838587:9078-8827;EPI_ISL_19350788:9090-8839;EPI_ISL_19467692:9306-9055;JX878411.1:10019-9768;JX878425.1:9459-9208;JX878426.1:9026-8775;KC257460.1:8845-8594;KJ642612.1:7366-7190;KJ642613.1:8888-8637;KJ642616.1:10587-10384;KJ642618.1:6433-6182;KP849469.1:9281-9030;KP849471.1:9254-9003;MN702445.1:2744-2493;MN702446.1:8890-8639;MN702447.1:2251-2000;MN702449.1:2747-2496;MN702452.1:8928-8677;MT903347.1:10856-10653;OR943698.1:9055-8804;PP601206.1:9076-8825;PQ178862.2:9107-8856;PQ221506.1:2897-2646;PQ221833.1:9068-8817 |
| OG0000233 | DQ011154.1:8442-8612;DQ011155.1:8354-8524;EPI_ISL_14838587:8341-8511;EPI_ISL_19350788:8350-8553;EPI_ISL_19467692:8578-8748;JX878411.1:9282-9452;JX878425.1:8722-8892;JX878426.1:8287-8457;KC257460.1:8120-8290;KJ642612.1:6626-6796;KJ642613.1:8125-8295;KJ642618.1:5672-5872;KP849469.1:8545-8715;KP849471.1:8528-8698;MN702445.1:2017-2187;MN702446.1:8151-8321;MN702447.1:1519-1689;MN702449.1:2018-2188;MN702452.1:8194-8364;OR943698.1:8304-8474;PP601206.1:8339-8509;PQ178862.2:8371-8574;PQ221506.1:2164-2334;PQ221833.1:8338-8508 |
| OG0000234 | DQ011154.1:48687-48860;DQ011155.1:48668-49051;EPI_ISL_14838587:48541-48714;EPI_ISL_19350788:47416-47799;EPI_ISL_19467692:48820-48993;JX878411.1:49535-49918;JX878425.1:49049-49222;JX878426.1:48494-48877;KC257460.1:48465-48638;KJ642612.1:46780-47163;KJ642613.1:48357-48530;KJ642618.1:45948-46331;KP849469.1:48857-49030;KP849471.1:48783-49166;MN702445.1:42202-42375;MN702446.1:48347-48520;MN702447.1:41713-41886;MN702449.1:42213-42386;MN702452.1:48386-48559;OR943698.1:48522-48905;PP601206.1:48539-48712;PQ178862.2:47393-47776;PQ221506.1:42362-42745;PQ221833.1:48532-48705 |
| OG0000235 | DQ011154.1:13360-13560;DQ011155.1:13340-13540;EPI_ISL_14838587:13214-13414;EPI_ISL_19350788:13226-13426;EPI_ISL_19467692:13492-13692;JX878411.1:14205-14405;JX878425.1:13720-13920;JX878426.1:13162-13362;KC257460.1:13139-13339;KJ642612.1:11447-11647;KJ642613.1:13028-13228;KJ642618.1:10619-10819;KP849469.1:13530-13730;KP849471.1:13450-13650;MN702445.1:6870-7070;MN702446.1:13016-13216;MN702447.1:6382-6582;MN702449.1:6878-7078;MN702452.1:13059-13259;OR943698.1:13197-13397;PP601206.1:13212-13412;PQ178862.2:13203-13403;PQ221506.1:7033-7233;PQ221833.1:13204-13404 |
| OG0000236 | DQ011154.1:20756-20971;DQ011155.1:20736-20951;EPI_ISL_14838587:20610-20825;EPI_ISL_19350788:19490-19705;EPI_ISL_19467692:20888-21103;JX878411.1:21603-21818;JX878425.1:21116-21331;JX878426.1:20559-20774;KC257460.1:20539-20754;KJ642612.1:18844-19059;KJ642613.1:20425-20640;KJ642618.1:18015-18230;KP849469.1:20926-21141;KP849471.1:20846-21061;MN702445.1:14269-14484;MN702446.1:20415-20630;MN702447.1:13778-13993;MN702449.1:14279-14494;MN702452.1:20455-20670;OR943698.1:20593-20808;PP601206.1:20608-20823;PQ178862.2:19467-19682;PQ221506.1:14430-14645;PQ221833.1:20600-20815 |
| OG0000237 | DQ011154.1:152906-153097;DQ011155.1:152848-153039;EPI_ISL_14838587:152749-153036;EPI_ISL_19350788:151505-151696;EPI_ISL_19467692:153046-153237;JX878411.1:153747-153938;JX878425.1:153235-153426;JX878426.1:152775-152966;KC257460.1:152631-152822;KJ642612.1:150965-151156;KJ642613.1:152514-152705;KJ642618.1:150430-150621;KP849469.1:153063-153254;KP849471.1:152996-153187;MN702445.1:146382-146573;MN702446.1:152546-152737;MN702447.1:145909-146100;MN702449.1:146420-146611;MN702452.1:152597-152788;OR943698.1:152751-152942;PP601206.1:152806-152997;PQ178862.2:151484-151675;PQ221506.1:146595-146786;PQ221833.1:152782-152973 |
| OG0000238 | DQ011154.1:189259-189104;DQ011155.1:189118-188963;EPI_ISL_14838587:189018-188863;EPI_ISL_19350788:187692-187537;EPI_ISL_19467692:189282-189127;JX878411.1:190102-189947;JX878425.1:189552-189397;JX878426.1:188966-188811;KC257460.1:188873-188718;KJ642612.1:187068-186913;KJ642613.1:188823-188668;KJ642618.1:186581-186426;KP849469.1:189408-189253;KP849471.1:189226-189071;MN702445.1:182465-182310;MN702446.1:188821-188666;MN702447.1:182027-181872;MN702449.1:182512-182357;MN702452.1:188708-188553;OR943698.1:188929-188774;PP601206.1:188973-188818;PQ178862.2:187457-187302;PQ221506.1:182829-182674;PQ221833.1:188950-188795 |
| OG0000239 | DQ011154.1:188146-187943;DQ011155.1:188005-187802;EPI_ISL_14838587:187905-187702;EPI_ISL_19350788:186579-186376;EPI_ISL_19467692:188169-187966;JX878411.1:188989-188786;JX878425.1:188439-188236;JX878426.1:187853-187650;KC257460.1:187760-187557;KJ642612.1:185955-185752;KJ642613.1:187710-187507;KJ642618.1:185473-185270;KP849469.1:188296-188093;KP849471.1:188113-187910;MN702445.1:181352-181149;MN702446.1:187708-187505;MN702447.1:180914-180711;MN702449.1:181399-181196;MN702452.1:187595-187392;OR943698.1:187816-187613;PP601206.1:187860-187657;PQ178862.2:186344-186141;PQ221506.1:181716-181513;PQ221833.1:187837-187634 |
| OG0000240 | DQ011154.1:167962-167393;DQ011155.1:167904-167335;EPI_ISL_14838587:167916-167347;EPI_ISL_19350788:166548-165979;EPI_ISL_19467692:168103-167534;JX878411.1:168804-168235;JX878425.1:168291-167722;JX878426.1:167864-167295;KC257460.1:167687-167118;KJ642612.1:166035-165466;KJ642613.1:167570-167001;KJ642618.1:165501-164932;KP849469.1:168120-167551;KP849471.1:168055-167486;MN702445.1:161438-160869;MN702446.1:167602-167033;MN702447.1:160972-160403;MN702449.1:161476-160907;MN702452.1:167660-167091;OR943698.1:167810-167241;PP601206.1:167863-167294;PQ178862.2:166527-165958;PQ221506.1:161697-161128;PQ221833.1:167844-167275 |
| OG0000241 | DQ011154.1:20878-20582;DQ011155.1:20858-20328;EPI_ISL_14838587:20732-20436;EPI_ISL_19350788:19612-19313;EPI_ISL_19467692:21010-20714;JX878411.1:21725-21429;JX878425.1:21238-20942;JX878426.1:20681-20385;KC257460.1:20661-20362;KJ642612.1:18966-18670;KJ642613.1:20547-20251;KJ642618.1:18137-17841;KP849469.1:21048-20752;KP849471.1:20968-20672;MN702445.1:14391-13858;MN702446.1:20537-20004;MN702447.1:13900-13604;MN702449.1:14401-14087;MN702452.1:20577-20281;OR943698.1:20715-20419;PP601206.1:20730-20434;PQ178862.2:19589-19290;PQ221506.1:14552-14256;PQ221833.1:20722-20426 |
| OG0000242 | DQ011154.1:53880-53716;DQ011155.1:53858-53694;EPI_ISL_14838587:53734-53570;EPI_ISL_19350788:52606-52442;EPI_ISL_19467692:54017-53853;JX878411.1:54725-54561;JX878425.1:54242-54078;JX878426.1:53691-53527;KC257460.1:53656-53492;KJ642612.1:51977-51813;KJ642613.1:53536-53372;KJ642618.1:51144-50980;KP849469.1:54050-53886;KP849471.1:53975-53811;MN702445.1:47393-47229;MN702446.1:53538-53374;MN702447.1:46906-46742;MN702449.1:47404-47240;MN702452.1:53579-53415;OR943698.1:53720-53556;PP601206.1:53732-53568;PQ178862.2:52583-52419;PQ221506.1:47559-47395;PQ221833.1:53725-53561 |
| OG0000243 | DQ011154.1:173852-173616;DQ011155.1:173774-173538;EPI_ISL_14838587:173680-173444;EPI_ISL_19350788:172321-172085;EPI_ISL_19467692:173921-173685;JX878411.1:174693-174457;JX878425.1:174145-173909;JX878426.1:173616-173380;KC257460.1:173510-173274;KJ642612.1:171806-171570;KJ642613.1:173413-173177;KJ642618.1:171336-171100;KP849469.1:174040-173804;KP849471.1:173843-173607;MN702445.1:167220-166984;MN702446.1:173390-173154;MN702447.1:166740-166504;MN702449.1:167258-167022;MN702452.1:173442-173206;OR943698.1:173431-173195;PP601206.1:173628-173392;PQ178862.2:172297-172061;PQ221506.1:167480-167244;PQ221833.1:173608-173372 |
| OG0000244 | DQ011154.1:19103-18951;DQ011155.1:19084-18932;EPI_ISL_14838587:18957-18805;EPI_ISL_19350788:18976-18824;EPI_ISL_19467692:19235-19083;JX878411.1:19949-19797;JX878425.1:19463-19311;JX878426.1:18906-18754;KC257460.1:18883-18731;KJ642612.1:17191-17039;KJ642613.1:18772-18620;KJ642618.1:16362-16210;KP849469.1:19273-19121;KP849471.1:19194-19042;MN702445.1:12614-12462;MN702446.1:18760-18608;MN702447.1:12125-11973;MN702449.1:12622-12470;MN702452.1:18802-18650;OR943698.1:18940-18788;PP601206.1:18955-18803;PQ178862.2:18953-18801;PQ221506.1:12777-12625;PQ221833.1:18947-18795 |
| OG0000245 | DQ011154.1:189540-189692;DQ011155.1:189399-189551;EPI_ISL_14838587:189299-189451;EPI_ISL_19350788:187973-188125;EPI_ISL_19467692:189563-189715;JX878425.1:189833-189985;JX878426.1:189247-189399;KC257460.1:189154-189306;KJ642612.1:187349-187501;KJ642613.1:189104-189256;KJ642618.1:186862-187014;KP849469.1:189689-189841;KP849471.1:189507-189659;MN702445.1:182746-182898;MN702446.1:189102-189254;MN702447.1:182308-182460;MN702449.1:182793-182945;MN702452.1:188989-189141;OR943698.1:189210-189362;PP601206.1:189254-189406;PQ178862.2:187738-187890;PQ221506.1:183110-183262;PQ221833.1:189231-189383 |
| OG0000246 | DQ011154.1:15694-15849;DQ011155.1:15674-15829;EPI_ISL_14838587:15548-15703;EPI_ISL_19350788:15560-15715;EPI_ISL_19467692:15826-15981;JX878411.1:16539-16694;JX878425.1:16054-16209;JX878426.1:15496-15651;KC257460.1:15473-15628;KJ642612.1:13781-13936;KJ642613.1:15362-15517;KJ642618.1:12952-13107;KP849469.1:15864-16019;KP849471.1:15784-15939;MN702445.1:9204-9359;MN702446.1:15350-15505;MN702447.1:8716-8871;MN702449.1:9212-9367;MN702452.1:15393-15548;PP601206.1:15546-15701;PQ178862.2:15537-15692;PQ221506.1:9367-9522;PQ221833.1:15538-15693 |
| OG0000247 | DQ011154.1:168912-169070;DQ011155.1:168854-169012;EPI_ISL_14838587:168866-169024;EPI_ISL_19350788:167498-167656;EPI_ISL_19467692:169053-169211;JX878425.1:169241-169399;JX878426.1:168814-168972;KC257460.1:168636-168794;KJ642612.1:166985-167143;KJ642613.1:168520-168678;KJ642618.1:166451-166609;KP849469.1:169070-169228;MN702445.1:162388-162546;MN702446.1:168552-168710;MN702447.1:161922-162080;MN702449.1:162426-162584;MN702452.1:168610-168768;OR943698.1:168760-168918;PP601206.1:168813-168971;PQ178862.2:167477-167635;PQ221506.1:162647-162805;PQ221833.1:168794-168952 |
| OG0000248 | DQ011154.1:20293-19976;DQ011155.1:20274-19957;EPI_ISL_14838587:20147-19830;EPI_ISL_19467692:20425-20108;JX878411.1:21140-20823;JX878425.1:20653-20336;JX878426.1:20096-19779;KC257460.1:20073-19756;KJ642612.1:18381-18064;KJ642613.1:19962-19645;KJ642618.1:17552-17235;KP849469.1:20463-20146;KP849471.1:20384-20067;MN702445.1:13804-13487;MN702446.1:19950-19633;MN702447.1:13315-12998;MN702449.1:13812-13495;MN702452.1:19992-19675;OR943698.1:20130-19813;PP601206.1:20145-19828;PQ221506.1:13967-13650;PQ221833.1:20137-19820 |
| OG0000249 | DQ011154.1:19852-19202;DQ011155.1:19833-19183;EPI_ISL_14838587:19706-19056;EPI_ISL_19467692:19984-19334;JX878411.1:20699-20049;JX878425.1:20212-19562;JX878426.1:19655-19005;KC257460.1:19632-18982;KJ642612.1:17940-17290;KJ642613.1:19521-18871;KJ642618.1:17111-16461;KP849469.1:20022-19372;KP849471.1:19943-19293;MN702445.1:13363-12713;MN702446.1:19509-18859;MN702447.1:12874-12224;MN702449.1:13371-12721;MN702452.1:19551-18901;OR943698.1:19689-19039;PP601206.1:19704-19054;PQ221506.1:13526-12876;PQ221833.1:19696-19046 |
| OG0000250 | DQ011154.1:6607-6437;DQ011155.1:6519-6349;EPI_ISL_14838587:6504-6334;EPI_ISL_19350788:6514-6344;EPI_ISL_19467692:6743-6573;JX878411.1:7448-7278;JX878425.1:6887-6717;JX878426.1:6452-6282;KC257460.1:6285-6115;KJ642612.1:4791-4621;KJ642613.1:6290-6120;KP849469.1:6710-6540;KP849471.1:6693-6523;MN702445.1:207-37;MN702446.1:6341-6171;MN702449.1:207-37;MN702452.1:6359-6189;OR943698.1:6470-6300;PP601206.1:6504-6334;PQ178862.2:6535-6365;PQ221506.1:329-159;PQ221833.1:6503-6333 |
| OG0000251 | DQ011154.1:7083-6874;DQ011155.1:6995-6786;EPI_ISL_14838587:6980-6771;EPI_ISL_19350788:6991-6782;EPI_ISL_19467692:7219-7010;JX878425.1:7363-7154;JX878426.1:6928-6719;KC257460.1:6761-6552;KJ642612.1:5267-5058;KJ642613.1:6766-6557;KP849469.1:7186-6977;KP849471.1:7169-6960;MN702445.1:683-474;MN702446.1:6817-6608;MN702447.1:160-2;MN702449.1:683-474;MN702452.1:6835-6626;PP601206.1:6980-6771;PQ178862.2:7012-6803;PQ221506.1:805-596;PQ221833.1:6979-6770 |
| OG0000252 | DQ011154.1:169754-169464;DQ011155.1:169660-169406;EPI_ISL_14838587:169582-169418;EPI_ISL_19467692:169823-169605;JX878411.1:170596-170306;JX878425.1:170047-169793;KC257460.1:169409-169188;KJ642612.1:167707-167537;KJ642613.1:169314-169072;KJ642618.1:167227-167003;KP849469.1:169942-169622;KP849471.1:169745-169557;MN702445.1:163122-162940;MN702446.1:169292-169104;MN702447.1:162638-162474;MN702449.1:163160-162978;MN702452.1:169344-169162;OR943698.1:169353-169078;PP601206.1:169529-169365;PQ221506.1:163375-163199;PQ221833.1:169510-169346 |
| OG0000253 | DQ011154.1:20580-20347;EPI_ISL_14838587:20434-20201;EPI_ISL_19350788:19329-19075;EPI_ISL_19467692:20712-20479;JX878411.1:21427-21194;JX878425.1:20940-20707;JX878426.1:20383-20150;KC257460.1:20360-20127;KJ642612.1:18668-18435;KJ642613.1:20249-20016;KJ642618.1:17839-17606;KP849469.1:20750-20517;MN702447.1:13602-13369;MN702449.1:14099-13866;MN702452.1:20279-20046;OR943698.1:20417-20184;PP601206.1:20432-20199;PQ178862.2:19306-19052;PQ221506.1:14254-14021;PQ221833.1:20424-20191 |
| OG0000254 | DQ011154.1:8960-9232;EPI_ISL_14838587:8864-9136;EPI_ISL_19350788:8876-9148;EPI_ISL_19467692:9092-9364;JX878411.1:9805-10077;JX878425.1:9245-9517;JX878426.1:8812-9084;KJ642613.1:8674-8946;KJ642618.1:6219-6491;KP849469.1:9067-9339;KP849471.1:9040-9312;MN702447.1:2037-2309;MN702452.1:8714-8986;OR943698.1:8841-9113;PP601206.1:8862-9134;PQ178862.2:8893-9165;PQ221506.1:2683-2955;PQ221833.1:8854-9126 |
| OG0000255 | EPI_ISL_13953610:148474-148271;EPI_ISL_17718458:148542-148339;EPI_ISL_18744049:148559-148356;ON676707.1:148603-148400;OP331335.1:148577-148374;OR459777.1:148547-148344;PP852971.1:148545-148342;PP852983.1:148562-148359;PP853000.1:148513-148310;PP853005.1:148521-148318;PP853007.1:148564-148361;PP853013.1:148536-148333;PP853027.1:148569-148366;PP853045.1:148529-148326;PP853048.1:148554-148351 |
| OG0000256 | DQ011154.1:48817-48656;EPI_ISL_14838587:48671-48510;EPI_ISL_19467692:48950-48789;JX878425.1:49179-49018;KC257460.1:48595-48434;KJ642613.1:48487-48326;KP849469.1:48987-48826;MN702445.1:42332-42171;MN702446.1:48477-48316;MN702447.1:41843-41682;MN702449.1:42343-42182;MN702452.1:48516-48355;PP601206.1:48669-48508;PQ221833.1:48662-48501 |
| OG0000257 | DQ011154.1:178943-179221;DQ011155.1:178864-179079;EPI_ISL_14838587:178765-178980;JX878411.1:179787-180065;JX878425.1:179236-179514;JX878426.1:178712-178927;MN702447.1:171837-171989;ON622722.2:179031-179186;OP535324.1:179053-179313 |
| OG0000258 | DQ011155.1:50230-50424;EPI_ISL_19350788:48978-49172;EPI_ISL_19467692:50383-50583;JX878411.1:51097-51291;KC257460.1:50028-50222;MN702445.1:43765-43959;MN702446.1:49910-50104;MN702449.1:43776-43970;PQ178862.2:48955-49149 |
| OG0000259 | DQ011155.1:50327-50163;EPI_ISL_19350788:49075-48911;EPI_ISL_19467692:50486-50316;JX878411.1:51194-51030;KC257460.1:50125-49961;MN702445.1:43862-43698;MN702446.1:50007-49843;MN702449.1:43873-43709;PQ178862.2:49052-48888 |
| OG0000260 | EPI_ISL_19467692:152719-152874;JX878426.1:152349-152603;KJ642612.1:150608-150793;KJ642618.1:149935-150258;KP849471.1:152657-152824;OR943698.1:152415-152579;PP601206.1:152401-152634;PQ221506.1:146196-146423 |
| OG0000261 | DQ011154.1:152700-152449;EPI_ISL_19467692:152840-152580;JX878411.1:153541-153290;KP849469.1:152857-152615;MN702447.1:145703-145479;MN702452.1:152391-152149;PP601206.1:152600-152262 |
| OG0000262 | AY603973.1:196416-196604;AY603973.1:2778-2590;AY753185.1:196684-196872;AY753185.1:2784-2596;KJ642616.1:196100-196288;KJ642616.1:2640-2452 |
| OG0000263 | AY603973.1:158259-158423;AY741551.1:158330-158494;AY753185.1:158514-158678;KJ642616.1:157980-158144;MT903347.1:158323-158487 |
| OG0000264 | AY603973.1:158762-158914;AY741551.1:158834-158986;AY753185.1:159017-159169;KJ642616.1:158483-158635;MT903347.1:158825-158977 |
| OG0000265 | AY603973.1:181214-180783;AY741551.1:180951-180790;AY753185.1:181387-181046;KJ642616.1:180946-180488;MT903347.1:180713-180354 |
| OG0000266 | AY603973.1:158468-158307;AY741551.1:158539-158378;AY753185.1:158723-158562;KJ642616.1:158189-158028;MT903347.1:158532-158371 |
| OG0000267 | KC257460.1:178600-178860;KJ642613.1:178508-178777;KP849469.1:179130-179363;PP601206.1:178721-178927;PQ221506.1:172579-172785 |
| OG0000268 | EPI_ISL_13374487:173419-173249;EPI_ISL_13502582:173411-173244;EPI_ISL_15831211:173388-173218;OR146330.1:173403-173233 |
| OG0000269 | KP849471.1:8961-8797;PP853000.1:8759-8604;PP853007.1:8807-8652;PP853045.1:8811-8656 |
| OG0000270 | DQ011155.1:24658-24506;EPI_ISL_19350788:23411-23259;PQ178862.2:23388-23236 |
| OG0000271 | PQ340480.1:191894-192457;PQ340480.1:5449-4886;PQ451842.1:180651-181025 |
| OG0000272 | AY603973.1:150291-150130;AY753185.1:150546-150385 |
| OG0000273 | AY741551.1:162107-162280;JX878426.1:159909-160070 |
| OG0000274 | DQ011155.1:166154-165942;KJ642618.1:163751-163539 |
| OG0000275 | DQ011155.1:135959-135756;KC257460.1:135757-135554 |
| OG0000276 | JX878411.1:7795-7956;OR943698.1:6817-6978 |
| OG0000277 | PP860026.1:145958-146116;PP860028.1:145921-146079 |
| OG0000278 | AY741551.1:159676-159945 |
| OG0000279 | DQ011154.1:179151-178933 |
| OG0000280 | DQ011155.1:179009-178854 |
| OG0000281 | EPI_ISL_13331598:179440-179060 |
| OG0000282 | EPI_ISL_13374487:179264-179082 |
| OG0000283 | EPI_ISL_13508471:179299-179144 |
| OG0000284 | EPI_ISL_13607904:179491-179306 |
| OG0000285 | EPI_ISL_13953610:89021-89173 |
| OG0000286 | EPI_ISL_13953610:42802-42215 |
| OG0000287 | EPI_ISL_13953610:179147-178974 |
| OG0000288 | EPI_ISL_14838587:178910-178755 |
| OG0000289 | EPI_ISL_14838587:152640-152416 |
| OG0000290 | EPI_ISL_15269699:179167-179012 |
| OG0000291 | EPI_ISL_15293002:179762-179283 |
| OG0000292 | EPI_ISL_16233784:179219-179064 |
| OG0000293 | EPI_ISL_17048206:156342-156566 |
| OG0000294 | EPI_ISL_17703567:179220-179065 |
| OG0000295 | EPI_ISL_17718458:179197-179024 |
| OG0000296 | EPI_ISL_18213374:179225-179052 |
| OG0000297 | EPI_ISL_18360394:146536-146381 |
| OG0000298 | EPI_ISL_18360394:141468-141262 |
| OG0000299 | EPI_ISL_18557816:150513-150668 |
| OG0000300 | EPI_ISL_18642365:179231-179058 |
| OG0000301 | EPI_ISL_18659846:179501-179013 |
| OG0000302 | EPI_ISL_18744049:179226-179053 |
| OG0000303 | EPI_ISL_19022858:179170-179015 |
| OG0000304 | EPI_ISL_19028778:179140-178967 |
| OG0000305 | EPI_ISL_19159116:179209-179036 |
| OG0000306 | EPI_ISL_19350788:177047-177259 |
| OG0000307 | EPI_ISL_19350788:177186-176962 |
| OG0000308 | EPI_ISL_19350788:168204-168049 |
| OG0000309 | EPI_ISL_19359677:179224-179051 |
| OG0000310 | EPI_ISL_19446306:179211-179038 |
| OG0000311 | EPI_ISL_19446306:192364-192191 |
| OG0000312 | EPI_ISL_19467692:179194-179003 |
| OG0000313 | JX878411.1:190513-190358 |
| OG0000314 | JX878411.1:179995-179777 |
| OG0000315 | JX878425.1:179444-179226 |
| OG0000316 | JX878426.1:178857-178702 |
| OG0000317 | KC257460.1:178808-178590 |
| OG0000318 | KJ642613.1:178725-178498 |
| OG0000319 | KJ642618.1:12294-12533 |
| OG0000320 | KJ642618.1:149856-150080 |
| OG0000321 | KJ642618.1:163477-163662 |
| OG0000322 | KJ642618.1:145139-145333 |
| OG0000323 | KJ642618.1:145260-145051 |
| OG0000324 | KJ642618.1:12726-12556 |
| OG0000325 | KP849469.1:179311-179120 |
| OG0000326 | KP849471.1:179106-178924 |
| OG0000327 | KP849471.1:138917-138180 |
| OG0000328 | LC831698.1:179207-179034 |
| OG0000329 | MN648051.1:179283-179456 |
| OG0000330 | MT903338.1:179419-179246 |
| OG0000331 | MT903340.1:179236-179063 |
| OG0000332 | MT903341.1:170781-170626 |
| OG0000333 | MT903341.1:3276-3112 |
| OG0000334 | MT903342.1:179321-179166 |
| OG0000335 | MT903347.1:182035-181841 |
| OG0000336 | ON622713.1:179813-179280 |
| OG0000337 | ON631963.1:179447-179067 |
| OG0000338 | ON755039.1:178699-178872 |
| OG0000339 | ON880420.1:179767-179270 |
| OG0000340 | ON918656.1:179232-179077 |
| OG0000341 | ON959133.1:179674-179168 |
| OG0000342 | ON959136.1:179696-179163 |
| OG0000343 | ON983168.1:178240-177743 |
| OG0000344 | OP019276.1:179223-179050 |
| OG0000345 | OP150924.1:179194-179709 |
| OG0000346 | OP150924.1:179667-179188 |
| OG0000347 | OP150924.1:179447-179184 |
| OG0000348 | OP235282.1:179223-179050 |
| OG0000349 | OP289782.1:179227-179054 |
| OG0000350 | OP331335.1:179219-179064 |
| OG0000351 | OP382479.1:179200-179045 |
| OG0000352 | OP422341.1:179215-179060 |
| OG0000353 | OP434519.1:179205-179032 |
| OG0000354 | OP536770.1:179226-179053 |
| OG0000355 | OP604566.1:179232-179059 |
| OG0000356 | OP612682.1:179133-178960 |
| OG0000357 | OP612684.1:89434-89078 |
| OG0000358 | OP612684.1:179373-179011 |
| OG0000359 | OP612686.1:179186-179013 |
| OG0000360 | OQ419007.1:178443-178270 |
| OG0000361 | OQ419012.1:178417-178244 |
| OG0000362 | OQ419031.1:178421-178248 |
| OG0000363 | OQ419031.1:23321-22932 |
| OG0000364 | OQ419047.1:178423-178250 |
| OG0000365 | OQ721951.1:179247-179056 |
| OG0000366 | OR035652.1:178996-179601 |
| OG0000367 | OR035652.1:179559-178990 |
| OG0000368 | OR035664.1:179505-178990 |
| OG0000369 | OR035702.1:179522-178989 |
| OG0000370 | OR146272.1:179161-179006 |
| OG0000371 | OR264364.1:179216-179061 |
| OG0000372 | OR427332.1:179216-179061 |
| OG0000373 | OR459777.1:179202-179029 |
| OG0000374 | OR459797.1:113855-113580 |
| OG0000375 | OR459797.1:179214-179059 |
| OG0000376 | OR943698.1:178838-178521 |
| OG0000377 | OR943698.1:15749-15348 |
| OG0000378 | PP098589.2:179225-179052 |
| OG0000379 | PP601206.1:178875-178711 |
| OG0000380 | PP852971.1:179214-179041 |
| OG0000381 | PP852983.1:179220-179065 |
| OG0000382 | PP853005.1:169994-170392 |
| OG0000383 | PP853005.1:179179-179024 |
| OG0000384 | PP853013.1:7793-7945 |
| OG0000385 | PP860026.1:28569-28375 |
| OG0000386 | PQ153226.1:179206-179033 |
| OG0000387 | PQ221506.1:172733-172569 |
| OG0000388 | PQ340477.1:179296-179024 |
| OG0000389 | PQ340478.1:179205-179014 |
| OG0000390 | PQ340480.1:179327-179082 |
| OG0000391 | PQ340483.1:179283-179074 |
| OG0000392 | PQ340485.1:179322-179059 |
| OG0000393 | PQ340486.1:179381-179073 |
| OG0000394 | PQ340487.1:179425-179099 |
| OG0000395 | PQ340489.1:179357-179076 |
| OG0000396 | PQ340490.1:179215-179024 |
| OG0000397 | PQ340491.1:179458-179087 |
| OG0000398 | PQ340493.1:179277-179059 |
| OG0000399 | PQ340495.1:179338-179093 |
| OG0000400 | PQ340496.1:179436-179128 |
| OG0000401 | PQ368489.1:179239-179066 |
| OG0000402 | PQ451835.1:179400-179137 |
| OG0000403 | PQ451838.1:179383-179129 |
| OG0000404 | PQ451841.1:179294-179094 |

**Table S3**. Annotation of MPXV protein across groups.

| **Group** | **Functional category** | **Description** |
| --- | --- | --- |
| OG0000010 | Immune Evasion or Adaptation Proteins | Serpin [Monkeypox virus] |
| OG0000190 | Enzymes and Catalytic Proteins | Putative monoglyceride lipase [Monkeypox virus] |
| OG0000118 | Immune Evasion or Adaptation Proteins | Host range protein [Monkeypox virus] |
| OG0000178 | Enzymes and Catalytic Proteins | Viral Core cysteine proteinase [Monkeypox virus] |
| OG0000327 | Structural Proteins | Orthopoxvirus A26L/A30L protein [Monkeypox virus] |
| OG0000114 | Enzymes and Catalytic Proteins | Ribonucleotide reductase small subunit [Monkeypox virus] |
| OG0000172 | Structural Proteins | Virion Core protein P4b [Monkeypox virus] |
| OG0000278 | Immune Evasion or Adaptation Proteins | Kelch-like protein [Camelpox virus] |
| OG0000087 | Structural Proteins | Type-I membrane glycoprotein [Monkeypox virus] |
| OG0000157 | Immune Evasion or Adaptation Proteins | Bcl-2-like protein [Monkeypox virus] |
| OG0000094 | Immune Evasion or Adaptation Proteins | IFN-alpha/beta-receptor-like secreted glycoprotein [Monkeypox virus] |
| OG0000363 | Immune Evasion or Adaptation Proteins | Ankyrin-like protein [Monkeypox virus] |
| OG0000191 | Enzymes and Catalytic Proteins | Phospholipase-D-like protein [Monkeypox virus] |
| OG0000374 | Replication and Transcription Regulatory Proteins | Early transcription factor 82 kDa subunit [Monkeypox virus] |
| OG0000113 | Replication and Transcription Regulatory Proteins | Telomere-binding protein I6 [Monkeypox virus] |
| OG0000251 | Immune Evasion or Adaptation Proteins | ankyrin-like protein [Taterapox virus] |
| OG0000162 | Enzymes and Catalytic Proteins | ATPase A32 [Monkeypox virus] |
| OG0000322 | Immune Evasion or Adaptation Proteins | C-type lectin-like type-II membrane protein [Vaccinia virus] |
| OG0000265 | Immune Evasion or Adaptation Proteins | CPXV216 protein [Cowpox virus] |
| OG0000085 | Replication and Transcription Regulatory Proteins | DNA-dependent RNA polymerase subunit rpo132 [Monkeypox virus] |
| OG0000111 | Immune Evasion or Adaptation Proteins | Protein F14 [Monkeypox virus] |
| OG0000321 | Immune Evasion or Adaptation Proteins | Ankyrin-like protein [Monkeypox virus] |
| OG0000177 | Structural Proteins | Internal virion L3/FP4 protein [Monkeypox virus] |
| OG0000046 | Structural Proteins | Membrane protein E8 [Monkeypox virus] |
| OG0000007 | Replication and Transcription Regulatory Proteins | Intracellular viral protein [Monkeypox virus] |
| OG0000286 | Enzymes and Catalytic Proteins | Poly(A) polymerase catalytic subunit [Monkeypox virus] |
| OG0000001 | Immune Evasion or Adaptation Proteins | Crm-B secreted TNF-alpha-receptor-like protein [Monkeypox virus] |
| OG0000139 | Structural Proteins | Virion protein D2 [Monkeypox virus] |
| OG0000179 | Structural Proteins | Virion Core protein E11 [Monkeypox virus] |
| OG0000141 | Structural Proteins | IMV heparin binding surface protein [Monkeypox virus] |
| OG0000194 | Replication and Transcription Regulatory Proteins | Zinc finger-like protein [Monkeypox virus] |
| OG0000101 | Replication and Transcription Regulatory Proteins | Late transcription factor VLTF-3 [Monkeypox virus] |
| OG0000184 | Immune Evasion or Adaptation Proteins | Protein F15 [Monkeypox virus] |
| OG0000036 | Enzymes and Catalytic Proteins | DNA ligase [Monkeypox virus] |
| OG0000166 | Immune Evasion or Adaptation Proteins | Ati or CPXV158 protein [Cowpox virus] |
| OG0000134 | Enzymes and Catalytic Proteins | S-S bond formation pathway protein [Monkeypox virus] |
| OG0000253 | Immune Evasion or Adaptation Proteins | D16L [Monkeypox virus Zaire-96-I-16] |
| OG0000173 | Replication and Transcription Regulatory Proteins | late transcription factor VLTF-2 [Variola virus] |
| OG0000186 | Structural Proteins | EEV maturation protein [Monkeypox virus] |
| OG0000019 | Enzymes and Catalytic Proteins | FEN1-like nuclease [Monkeypox virus] |
| OG0000181 | Replication and Transcription Regulatory Proteins | Double-stranded RNA binding protein [Monkeypox virus] |
| OG0000102 | Enzymes and Catalytic Proteins | Carbonic anhydrase [Monkeypox virus] |
| OG0000133 | Immune Evasion or Adaptation Proteins | A5L protein-like [Monkeypox virus] |
| OG0000079 | Replication and Transcription Regulatory Proteins | Nucleic acid binding protein VP8/L4R [Monkeypox virus] |
| OG0000096 | Structural Proteins | A26L [Monkeypox virus Zaire-96-I-16] |
| OG0000119 | Immune Evasion or Adaptation Proteins | Ankyrin repeat protein [Monkeypox virus] |
| OG0000274 | Unknown | P68475\|YVBD_VACCC Uncharacterized 7.8 kDa protein |
| OG0000058 | Replication and Transcription Regulatory Proteins | Intermediate transcription factor VITF-3 [Monkeypox virus] |
| OG0000028 | Enzymes and Catalytic Proteins | NUDIX domain protein [Monkeypox virus] |
| OG0000054 | Structural Proteins | Virion Core protein D3 [Monkeypox virus] |
| OG0000069 | Enzymes and Catalytic Proteins | Ser/thr kinase [Monkeypox virus] |
| OG0000147 | Replication and Transcription Regulatory Proteins | Telomere-binding protein I1 [Monkeypox virus] |
| OG0000206 | Unknown | A0A7H0DN40\|PG067_MONPV Protein OPG067 |
| OG0000195 | Unknown | P21121\|YVCC_VACCC Uncharacterized 9.2 kDa protein |
| OG0000248 | Immune Evasion or Adaptation Proteins | D15L [Monkeypox virus Zaire-96-I-16] |
| OG0000196 | Unknown | hypothetical protein KM543_gp198 [Orthopoxvirus Abatino] |
| OG0000095 | Immune Evasion or Adaptation Proteins | CMLV203 [Camelpox virus] |
| OG0000164 | Structural Proteins | Envelope protein A28 homolog [Monkeypox virus] |
| OG0000093 | Immune Evasion or Adaptation Proteins | Bcl-2-like protein [Monkeypox virus] |
| OG0000175 | Enzymes and Catalytic Proteins | Tyr/ser protein phosphatase [Monkeypox virus] |
| OG0000158 | Immune Evasion or Adaptation Proteins | Bcl-2-like protein [Monkeypox virus] |
| OG0000105 | Enzymes and Catalytic Proteins | Metalloendopeptidase [Monkeypox virus] |
| OG0000053 | Enzymes and Catalytic Proteins | DNA topoisomerase type I [Monkeypox virus] |
| OG0000185 | Structural Proteins | Palmytilated EEV membrane protein [Monkeypox virus] |
| OG0000022 | Replication and Transcription Regulatory Proteins | Cap-specific mRNA [Monkeypox virus] |
| OG0000151 | Unknown | Cytoplasmic protein [Monkeypox virus] |
| OG0000012 | Immune Evasion or Adaptation Proteins | Ankyrin repeat protein [Monkeypox virus] |
| OG0000125 | Structural Proteins | IMV membrane protein A30 [Monkeypox virus] |
| OG0000033 | Structural Proteins | EEV glycoprotein [Monkeypox virus] |
| OG0000026 | Enzymes and Catalytic Proteins | Uracil DNA glycosylase superfamily [Monkeypox virus] |
| OG0000035 | Enzymes and Catalytic Proteins | Thymidylate kinase [Monkeypox virus] |
| OG0000048 | Enzymes and Catalytic Proteins | Nlpc/p60 superfamily protein [Monkeypox virus] |
| OG0000122 | Immune Evasion or Adaptation Proteins | Chemokine binding protein [Monkeypox virus] |
| OG0000049 | Replication and Transcription Regulatory Proteins | Late transcription factor VLTF-1 [Monkeypox virus] |
| OG0000032 | Immune Evasion or Adaptation Proteins | CPXV166 protein [Monkeypox virus] |
| OG0000016 | Immune Evasion or Adaptation Proteins | CPXV063 protein [Cowpox virus] |
| OG0000067 | Immune Evasion or Adaptation Proteins | M137R [Monkeypox virus] |
| OG0000136 | Enzymes and Catalytic Proteins | mRNA capping enzyme small subunit [Monkeypox virus] |
| OG0000013 | Immune Evasion or Adaptation Proteins | C4L/C10L-like family protein [Monkeypox virus] |
| OG0000121 | Enzymes and Catalytic Proteins | Hydroxysteroid dehydrogenase [Monkeypox virus] |
| OG0000215 | Immune Evasion or Adaptation Proteins | P21070\|A52_VACCC Protein A52 |
| OG0000137 | Unknown | P68481\|YVDE_VACCC Uncharacterized 9.3 kDa protein |
| OG0000176 | Unknown | hypothetical protein VARVgp082 [Variola virus] |
| OG0000129 | Replication and Transcription Regulatory Proteins | Zinc finger-like protein [Monkeypox virus] |
| OG0000098 | Structural Proteins | Myristylated protein [Monkeypox virus] |
| OG0000183 | Structural Proteins | Protein F16 [Monkeypox virus] |
| OG0000250 | Immune Evasion or Adaptation Proteins | ankyrin-like protein [Taterapox virus] |
| OG0000249 | Immune Evasion or Adaptation Proteins | Complement control protein (CCP) [Monkeypox virus] |
| OG0000199 | Immune Evasion or Adaptation Proteins | P21075\|B17_VACCC Protein B17 |
| OG0000059 | Structural Proteins | Viral membrane formation protein [Monkeypox virus] |
| OG0000024 | Structural Proteins | putative viral membrane protein [Variola virus] |
| OG0000148 | Structural Proteins | Iev morphogenesis protein [Monkeypox virus] |
| OG0000182 | Structural Proteins | Iev morphogenesis protein [Monkeypox virus] |
| OG0000009 | Immune Evasion or Adaptation Proteins | N-methyl D-aspartate receptor homolog [Monkeypox virus] |
| OG0000366 | Unknown | low complexity protein [Salmon gill poxvirus] |
| OG0000042 | Immune Evasion or Adaptation Proteins | IL-1 receptor homolog [Monkeypox virus] |
| OG0000159 | Immune Evasion or Adaptation Proteins | D18L [Monkeypox virus Zaire-96-I-16] |
| OG0000106 | Immune Evasion or Adaptation Proteins | Telomere-binding protein [Monkeypox virus] |
| OG0000169 | Structural Proteins | Virion Core protein P4a [Monkeypox virus] |
| OG0000254 | Unknown | P21123\|YVCE_VACCC Uncharacterized 7.2 kDa protein |
| OG0000209 | Unknown | putative gCPXV0003 protein [Orthopoxvirus Abatino] |
| OG0000345 | Unknown | low complexity protein [Salmon gill poxvirus] |
| OG0000132 | Structural Proteins | Virion morphogenesis protein [Monkeypox virus] |
| OG0000168 | Immune Evasion or Adaptation Proteins | A12 protein [Monkeypox virus] |
| OG0000082 | Replication and Transcription Regulatory Proteins | MutT motif protein [Monkeypox virus] |
| OG0000324 | Immune Evasion or Adaptation Proteins | ankyrin-like protein [Vaccinia virus] |
| OG0000145 | Replication and Transcription Regulatory Proteins | DNA-binding phosphoprotein [Monkeypox virus] |
| OG0000131 | Structural Proteins | Core protein A15 [Monkeypox virus] |
| OG0000051 | Replication and Transcription Regulatory Proteins | DNA-directed RNA polymerase subunit [Monkeypox virus] |
| OG0000083 | Replication and Transcription Regulatory Proteins | DNA helicase [Monkeypox virus] |
| OG0000115 | Immune Evasion or Adaptation Proteins | Caspase-9 inhibitor [Monkeypox virus] |
| OG0000089 | Immune Evasion or Adaptation Proteins | Schlafen [Monkeypox virus] |
| OG0000120 | Immune Evasion or Adaptation Proteins | retroviral pseudoprotease-like protein [Monkeypox virus] |
| OG0000211 | Immune Evasion or Adaptation Proteins | N2R [Monkeypox virus Zaire-96-I-16] |
| OG0000040 | Immune Evasion or Adaptation Proteins | Virulence protein [Monkeypox virus] |
| OG0000130 | Structural Proteins | IMV membrane protein P21 [Monkeypox virus] |
| OG0000055 | Enzymes and Catalytic Proteins | NTPase [Monkeypox virus] |
| OG0000149 | Enzymes and Catalytic Proteins | Poly(A) polymerase catalytic subunit [Monkeypox virus] |
| OG0000246 | Unknown | P21120\|YVCB_VACCC Uncharacterized 8.4 kDa protein |
| OG0000156 | Immune Evasion or Adaptation Proteins | Bcl-2-like protein [Monkeypox virus] |
| OG0000219 | Structural Proteins | cowpox A-type inclusion protein [Taterapox virus] |
| OG0000146 | Structural Proteins | IMV membrane protein I2 [Monkeypox virus] |
| OG0000319 | Unknown | P21122\|YVCD_VACCC Uncharacterized 7.9 kDa protein |
| OG0000091 | Immune Evasion or Adaptation Proteins | Ankyrin-like protein [Monkeypox virus] |
| OG0000084 | Enzymes and Catalytic Proteins | Holliday junction resolvase [Monkeypox virus] |
| OG0000062 | Immune Evasion or Adaptation Proteins | IEV transmembrane phosphoprotein [Monkeypox virus] |
| OG0000123 | Immune Evasion or Adaptation Proteins | CD47-like protein [Monkeypox virus] |
| OG0000214 | Immune Evasion or Adaptation Proteins | IL-1 receptor homolog [Monkeypox virus] |
| OG0000047 | Replication and Transcription Regulatory Proteins | RNA helicase NPH-II [Monkeypox virus] |
| OG0000126 | Structural Proteins | Orthopoxvirus A26L/A30L protein [Monkeypox virus] |
| OG0000110 | Enzymes and Catalytic Proteins | DNA polymerase [Monkeypox virus] |
| OG0000155 | Immune Evasion or Adaptation Proteins | Ankyrin-like protein [Monkeypox virus] |
| OG0000180 | Replication and Transcription Regulatory Proteins | DNA-directed RNA polymerase 30 kDa polypeptide [Monkeypox virus] |
| OG0000135 | Immune Evasion or Adaptation Proteins | Rifampicin resistance protein [Monkeypox virus] |
| OG0000061 | Immune Evasion or Adaptation Proteins | MHC class II antigen presentation inhibitor [Monkeypox virus] |
| OG0000034 | Structural Proteins | MPXV_gp150 [Monkeypox virus] |
| OG0000066 | Enzymes and Catalytic Proteins | Copper/zinc superoxide dismutase [Monkeypox virus] |
| OG0000078 | Structural Proteins | IMV membrane protein L1R [Monkeypox virus] |
| OG0000163 | Replication and Transcription Regulatory Proteins | DNA-directed RNA polymerase 35 kDa subunit [Monkeypox virus] |
| OG0000160 | Immune Evasion or Adaptation Proteins | Bcl-2-like protein [Monkeypox virus] |
| OG0000077 | Structural Proteins | Myristylated protein [Monkeypox virus] |
| OG0000202 | Immune Evasion or Adaptation Proteins | Interleukin-18-binding protein [Monkeypox virus] |
| OG0000104 | Enzymes and Catalytic Proteins | Glutaredoxin-2 [Monkeypox virus] |
| OG0000263 | Immune Evasion or Adaptation Proteins | putative A53R protein [Orthopoxvirus Abatino] |
| OG0000140 | Replication and Transcription Regulatory Proteins | RNA polymerase-associated transcription-specificity factor RAP94 [Monkeypox virus] |
| OG0000017 | Structural Proteins | IMV membrane protein E6 [Monkeypox virus] |
| OG0000060 | Replication and Transcription Regulatory Proteins | DNA polymerase processivity factor [Monkeypox virus] |
| OG0000377 | Unknown | hypothetical protein VACWR020 [Vaccinia virus] |
| OG0000221 | Replication and Transcription Regulatory Proteins | CPXV193 protein [Cowpox virus] |
| OG0000143 | Immune Evasion or Adaptation Proteins | Entry/fusion complex component [Monkeypox virus] |
| OG0000039 | Structural Proteins | EEV type-I membrane glycoprotein [Monkeypox virus] |
| OG0000116 | Immune Evasion or Adaptation Proteins | NFkB inhibitor [Monkeypox virus] |
| OG0000167 | Unknown | DUF1029 domain protein [Monkeypox virus] |
| OG0000002 | Immune Evasion or Adaptation Proteins | Chemokine binding protein [Monkeypox virus] |
| OG0000198 | Unknown | hypothetical protein KM542_gp113 [Akhmeta virus] |
| OG0000117 | Immune Evasion or Adaptation Proteins | Ankyrin-like protein [Monkeypox virus] |
| OG0000269 | Unknown | CMLV012 [Camelpox virus] |
| OG0000128 | Structural Proteins | IMV membrane protein A21 [Monkeypox virus] |
| OG0000070 | Immune Evasion or Adaptation Proteins | Serpin [Monkeypox virus] |
| OG0000224 | Immune Evasion or Adaptation Proteins | IL-1 receptor homolog [Monkeypox virus] |
| OG0000045 | Replication and Transcription Regulatory Proteins | DNA-binding phosphoprotein [Monkeypox virus] |
| OG0000188 | Unknown | CPXV054 protein [Monkeypox virus] |
| OG0000099 | Structural Proteins | IMV membrane protein A14 [Monkeypox virus] |
| OG0000074 | Enzymes and Catalytic Proteins | Sulfhydryl oxidase [Monkeypox virus] |
| OG0000138 | Unknown | YVDC_VACCW Uncharacterized 7.3 kDa protein |
| OG0000056 | Replication and Transcription Regulatory Proteins | Early transcription factor 70 kDa subunit [Monkeypox virus] |
| OG0000218 | Immune Evasion or Adaptation Proteins | A49_VACCA Protein A49 |
| OG0000107 | Structural Proteins | IMV membrane protein I5 [Monkeypox virus] |
| OG0000075 | Replication and Transcription Regulatory Proteins | Late transcription elongation factor [Monkeypox virus] |
| OG0000100 | Structural Proteins | IMV membrane protein A13L [Monkeypox virus] |
| OG0000008 | Immune Evasion or Adaptation Proteins | Bcl-2-like protein [Monkeypox virus] |
| OG0000382 | Replication and Transcription Regulatory Proteins | Ser/thr kinase [Monkeypox virus] |
| OG0000073 | Unknown | P20562\|YVFD_VACCC Uncharacterized 9.9 kDa protein |
| OG0000232 | Immune Evasion or Adaptation Proteins | D4L [Monkeypox virus Zaire-96-I-16] |
| OG0000092 | Unknown | hypothetical protein ECTVgp160 [Ectromelia virus] |
| OG0000080 | Structural Proteins | Membrane protein CL5 [Monkeypox virus] |
| OG0000153 | Enzymes and Catalytic Proteins | dUTPase [Monkeypox virus] |
| OG0000271 | Immune Evasion or Adaptation Proteins | Ankyrin repeat protein [Monkeypox virus] |
| OG0000065 | Immune Evasion or Adaptation Proteins | Profilin domain protein [Monkeypox virus] |
| OG0000108 | Enzymes and Catalytic Proteins | Glutaredoxin-1 [Monkeypox virus] |
| OG0000213 | Immune Evasion or Adaptation Proteins | CMLV008 [Camelpox virus] |
| OG0000192 | Immune Evasion or Adaptation Proteins | Kelch-like protein [Monkeypox virus] |
| OG0000057 | Enzymes and Catalytic Proteins | DNA-directed RNA polymerase 19 kDa subunit [Monkeypox virus] |
| OG0000050 | Enzymes and Catalytic Proteins | Thymidine kinase [Monkeypox virus] |
| OG0000170 | Structural Proteins | IMV membrane protein A9 [Monkeypox virus] |
| OG0000018 | Immune Evasion or Adaptation Proteins | Myristoylated protein E7 [Monkeypox virus] |
| OG0000208 | Unknown | putative gCPXV0003 protein [Orthopoxvirus Abatino] |
| OG0000023 | Enzymes and Catalytic Proteins | DNA-dependent RNA polymerase subunit rpo147 [Monkeypox virus] |
| OG0000197 | Immune Evasion or Adaptation Proteins | B22R family protein [Monkeypox virus] |
| OG0000112 | Structural Proteins | Protein F11 [Monkeypox virus] |
| OG0000273 | Enzymes and Catalytic Proteins | guanylate kinase [Akhmeta virus] |
| OG0000052 | Replication and Transcription Regulatory Proteins | Late transcription factor VLTF-4 [Monkeypox virus] |
| OG0000031 | Unknown | P20520\|YVAK_VACCC Uncharacterized 7.5 kDa protein |
| OG0000165 | Structural Proteins | IMV surface fusion protein [Monkeypox virus] |
| OG0000174 | Enzymes and Catalytic Proteins | Nucleoside triphosphatase I [Monkeypox virus] |
| OG0000029 | Immune Evasion or Adaptation Proteins | CPXV130 protein [Cowpox virus] |
| OG0000071 | Immune Evasion or Adaptation Proteins | Ankyrin repeat protein [Monkeypox virus] |
| OG0000154 | Immune Evasion or Adaptation Proteins | Serpin [Monkeypox virus] |
| OG0000124 | Unknown | P68627\|YVAM_VACCW Uncharacterized 8.6 kDa protein |
| OG0000005 | Immune Evasion or Adaptation Proteins | Virulence protein [Monkeypox virus] |
| OG0000086 | Structural Proteins | EEV glycoprotein [Monkeypox virus] |
| OG0000258 | Unknown | Hypothetical protein ECTVgp045 [Ectromelia virus] |
| OG0000152 | Immune Evasion or Adaptation Proteins | Kelch-like protein [Monkeypox virus] |
| OG0000097 | Immune Evasion or Adaptation Proteins | HSPV146b [Horsepox virus] |
| OG0000189 | Immune Evasion or Adaptation Proteins | CPXV053 protein [Monkeypox virus] |
| OG0000038 | Structural Proteins | Hemagglutinin [Monkeypox virus] |
| OG0000063 | Immune Evasion or Adaptation Proteins | CPXV173 protein [Monkeypox virus] |
| OG0000006 | Unknown | Brix domain protein [Monkeypox virus] |
| OG0000247 | Immune Evasion or Adaptation Proteins | kelch-like protein [Taterapox virus] |
| OG0000021 | Structural Proteins | IMV membrane protein J1 [Monkeypox virus] |
| OG0000275 | Unknown | putative Ati protein [Orthopoxvirus Abatino] |
| OG0000187 | Structural Proteins | IMV membrane protein L1R [Monkeypox virus] |
| OG0000270 | Unknown | HSPV033c [Horsepox virus] |
| OG0000020 | Structural Proteins | Crescent membrane and immature virion formation protein [Monkeypox virus] |
| OG0000171 | Replication and Transcription Regulatory Proteins | Early transcription factor 82 kDa subunit [Monkeypox virus] |
| OG0000068 | Enzymes and Catalytic Proteins | Ser/thr kinase [Monkeypox virus] |
| OG0000064 | Unknown | hypothetical protein NBT03_gp144 [Monkeypox virus] |
| OG0000259 | Unknown | P20554\|YVEB_VACCC Uncharacterized 11.9 kDa protein |
| OG0000210 | Unknown | P68477\|YVBE_VACCC Uncharacterized 10.5 kDa protein |
| OG0000150 | Enzymes and Catalytic Proteins | Serine/threonine-protein kinase [Monkeypox virus] |
| OG0000072 | Immune Evasion or Adaptation Proteins | Bcl-2-like protein [Monkeypox virus] |
| OG0000090 | Immune Evasion or Adaptation Proteins | Ankyrin repeat protein [Monkeypox virus] |
| OG0000144 | Enzymes and Catalytic Proteins | Ribonucleoside-diphosphate reductase [Monkeypox virus] |
| OG0000041 | Immune Evasion or Adaptation Proteins | Soluble interferon-gamma receptor-like protein [Monkeypox virus] |
| OG0000088 | Immune Evasion or Adaptation Proteins | Bcl-2-like protein [Monkeypox virus] |
| OG0000212 | Unknown | hypothetical protein TATV_DAH68_009 [Taterapox virus] |
| OG0000127 | Structural Proteins | A27L [Monkeypox virus Zaire-96-I-16] |
| OG0000081 | Structural Proteins | Late protein H7 [Monkeypox virus] |
| OG0000357 | Replication and Transcription Regulatory Proteins | RNA polymerase-associated transcription-specificity factor RAP94 [Monkeypox virus] |
| OG0000103 | Structural Proteins | Assembly protein G7 [Monkeypox virus] |
| OG0000255 | Enzymes and Catalytic Proteins | Hydroxysteroid dehydrogenase [Monkeypox virus] |
| OG0000025 | Enzymes and Catalytic Proteins | mRNA capping enzyme large subunit [Monkeypox virus] |
| OG0000030 | Replication and Transcription Regulatory Proteins | Intermediate transcription factor VITF-3 [Monkeypox virus] |
| OG0000003 | Immune Evasion or Adaptation Proteins | Ankyrin repeat protein [Monkeypox virus] |
| OG0000241 | Immune Evasion or Adaptation Proteins | P87617\|C2_CWPXG Kelch repeat protein C2 |
| OG0000243 | Immune Evasion or Adaptation Proteins | B15L [Monkeypox virus Zaire-96-I-16] |
| OG0000027 | Replication and Transcription Regulatory Proteins | RNA polymerase subunit RPO18 [Monkeypox virus] |
| OG0000142 | Unknown | hypothetical protein KM542_gp093 [Akhmeta virus] |
| OG0000037 | Immune Evasion or Adaptation Proteins | CMLV175 [Camelpox virus] |
| OG0000293 | Immune Evasion or Adaptation Proteins | CPXV192 protein [Cowpox virus] |
| OG0000076 | Replication and Transcription Regulatory Proteins | DNA-directed RNA polymerase 7 kDa subunit [Monkeypox virus] |
| OG0000000 | Immune Evasion or Adaptation Proteins | Ankyrin repeat protein [Monkeypox virus] |
| OG0000203 | Unknown | hypothetical protein VARVgp003 [Variola virus] |
| OG0000205 | Immune Evasion or Adaptation Proteins | EGF-like domain protein [Monkeypox virus] |
| OG0000200 | Immune Evasion or Adaptation Proteins | putative A47L protein [Orthopoxvirus Abatino] |
| OG0000393 | Unknown |  |
| OG0000330 | Unknown |  |
| OG0000392 | Unknown |  |
| OG0000302 | Unknown |  |
| OG0000354 | Unknown |  |
| OG0000300 | Unknown |  |
| OG0000331 | Unknown |  |
| OG0000340 | Unknown |  |
| OG0000315 | Unknown |  |
| OG0000306 | Unknown |  |
| OG0000015 | Unknown |  |
| OG0000320 | Unknown |  |
| OG0000252 | Unknown |  |
| OG0000225 | Unknown |  |
| OG0000298 | Unknown |  |
| OG0000355 | Unknown |  |
| OG0000336 | Unknown |  |
| OG0000344 | Unknown |  |
| OG0000323 | Unknown |  |
| OG0000227 | Unknown |  |
| OG0000353 | Unknown |  |
| OG0000360 | Unknown |  |
| OG0000260 | Unknown |  |
| OG0000394 | Unknown |  |
| OG0000312 | Unknown |  |
| OG0000228 | Unknown |  |
| OG0000367 | Unknown |  |
| OG0000217 | Unknown |  |
| OG0000285 | Unknown |  |
| OG0000339 | Unknown |  |
| OG0000220 | Unknown |  |
| OG0000362 | Unknown |  |
| OG0000395 | Unknown |  |
| OG0000375 | Unknown |  |
| OG0000346 | Unknown |  |
| OG0000328 | Unknown |  |
| OG0000043 | Unknown |  |
| OG0000318 | Unknown |  |
| OG0000294 | Unknown |  |
| OG0000359 | Unknown |  |
| OG0000369 | Unknown |  |
| OG0000342 | Unknown |  |
| OG0000284 | Unknown |  |
| OG0000364 | Unknown |  |
| OG0000365 | Unknown |  |
| OG0000381 | Unknown |  |
| OG0000308 | Unknown |  |
| OG0000201 | Unknown |  |
| OG0000256 | Unknown |  |
| OG0000304 | Unknown |  |
| OG0000004 | Unknown |  |
| OG0000268 | Unknown |  |
| OG0000267 | Unknown |  |
| OG0000397 | Unknown |  |
| OG0000337 | Unknown |  |
| OG0000296 | Unknown |  |
| OG0000403 | Unknown |  |
| OG0000044 | Unknown |  |
| OG0000239 | Unknown |  |
| OG0000390 | Unknown |  |
| OG0000356 | Unknown |  |
| OG0000240 | Unknown |  |
| OG0000398 | Unknown |  |
| OG0000368 | Unknown |  |
| OG0000400 | Unknown |  |
| OG0000234 | Unknown |  |
| OG0000014 | Unknown |  |
| OG0000387 | Unknown |  |
| OG0000376 | Unknown |  |
| OG0000289 | Unknown |  |
| OG0000231 | Unknown |  |
| OG0000309 | Unknown |  |
| OG0000386 | Unknown |  |
| OG0000329 | Unknown |  |
| OG0000316 | Unknown |  |
| OG0000348 | Unknown |  |
| OG0000358 | Unknown |  |
| OG0000244 | Unknown |  |
| OG0000272 | Unknown |  |
| OG0000277 | Unknown |  |
| OG0000222 | Unknown |  |
| OG0000216 | Unknown |  |
| OG0000282 | Unknown |  |
| OG0000301 | Unknown |  |
| OG0000262 | Unknown |  |
| OG0000238 | Unknown |  |
| OG0000193 | Unknown |  |
| OG0000399 | Unknown |  |
| OG0000235 | Unknown |  |
| OG0000299 | Unknown |  |
| OG0000307 | Unknown |  |
| OG0000109 | Unknown |  |
| OG0000283 | Unknown |  |
| OG0000290 | Unknown |  |
| OG0000388 | Unknown |  |
| OG0000233 | Unknown |  |
| OG0000257 | Unknown |  |
| OG0000297 | Unknown |  |
| OG0000391 | Unknown |  |
| OG0000295 | Unknown |  |
| OG0000310 | Unknown |  |
| OG0000404 | Unknown |  |
| OG0000280 | Unknown |  |
| OG0000266 | Unknown |  |
| OG0000230 | Unknown |  |
| OG0000223 | Unknown |  |
| OG0000207 | Unknown |  |
| OG0000338 | Unknown |  |
| OG0000372 | Unknown |  |
| OG0000335 | Unknown |  |
| OG0000236 | Unknown |  |
| OG0000349 | Unknown |  |
| OG0000378 | Unknown |  |
| OG0000314 | Unknown |  |
| OG0000237 | Unknown |  |
| OG0000380 | Unknown |  |
| OG0000351 | Unknown |  |
| OG0000279 | Unknown |  |
| OG0000313 | Unknown |  |
| OG0000373 | Unknown |  |
| OG0000402 | Unknown |  |
| OG0000261 | Unknown |  |
| OG0000292 | Unknown |  |
| OG0000311 | Unknown |  |
| OG0000242 | Unknown |  |
| OG0000383 | Unknown |  |
| OG0000264 | Unknown |  |
| OG0000334 | Unknown |  |
| OG0000361 | Unknown |  |
| OG0000291 | Unknown |  |
| OG0000370 | Unknown |  |
| OG0000288 | Unknown |  |
| OG0000343 | Unknown |  |
| OG0000161 | Unknown |  |
| OG0000371 | Unknown |  |
| OG0000281 | Unknown |  |
| OG0000379 | Unknown |  |
| OG0000350 | Unknown |  |
| OG0000317 | Unknown |  |
| OG0000389 | Unknown |  |
| OG0000276 | Unknown |  |
| OG0000326 | Unknown |  |
| OG0000011 | Unknown |  |
| OG0000401 | Unknown |  |
| OG0000245 | Unknown |  |
| OG0000384 | Unknown |  |
| OG0000229 | Unknown |  |
| OG0000396 | Unknown |  |
| OG0000332 | Unknown |  |
| OG0000305 | Unknown |  |
| OG0000347 | Unknown |  |
| OG0000287 | Unknown |  |
| OG0000352 | Unknown |  |
| OG0000325 | Unknown |  |
| OG0000341 | Unknown |  |
| OG0000226 | Unknown |  |
| OG0000303 | Unknown |  |
| OG0000204 | Unknown |  |
| OG0000385 | Unknown |  |
| OG0000333 | Unknown |  |

Table S4. Recombination events among the 157 strains (only those detected by three or more methods in RDP5 were included in the table).

| Number In .RDP File | Begin | End | Recombinant Sequence(s) | Minor Parental Sequence(s) | Major Parental Sequence(s) |
| --- | --- | --- | --- | --- | --- |
| 4~ | 83938 | 84195 | ^PP853007.1 | Unknown (PQ178862.2) | PP853000.1 |
|  |  |  |  |  |  |
| 8~ | 40559 | 75258 | ^KJ642616.1 | PP852971.1 | Unknown (JX878411.1) |
|  |  |  | AY741551.1 | EPI_ISL_13374487 | Unknown(PQ178862.2) |
|  |  |  | MT903347.1 | PP496048.1 | Unknown(KJ642612.1) |
|  |  |  | AY753185.1 | EPI_ISL_19359677 | Unknown(KJ642613.1) |
|  |  |  | AY603973.1 | EPI_ISL_19446306 | Unknown(DQ011155.1) |
|  |  |  |  | EPI_ISL_17048206 | Unknown(MN702445.1) |
|  |  |  |  | EPI_ISL_13508471 | Unknown(MN702446.1) |
|  |  |  |  | OP160532.1 | Unknown(MN702447.1) |
|  |  |  |  | EPI_ISL_15641561 | Unknown(MN702449.1) |
|  |  |  |  | EPI_ISL_15269699 | Unknown(MN702452.1) |
|  |  |  |  | OR146272.1 | Unknown(PQ221506.1) |
|  |  |  |  | OR146330.1 | Unknown(PQ221833.1) |
|  |  |  |  | OP382479.1 | Unknown(PP601206.1) |
|  |  |  |  | ON755039.1 | Unknown(KP849469.1) |
|  |  |  |  | ON622722.2 | Unknown(KP849471.1) |
|  |  |  |  | OQ721951.1 | Unknown(JX878425.1) |
|  |  |  |  | OQ721954.1 | Unknown(JX878426.1) |
|  |  |  |  | OQ581853.1 | Unknown(KC257460.1) |
|  |  |  |  | OQ581855.1 | Unknown(EPI_ISL_19467692) |
|  |  |  |  | ON918656.1 | Unknown(EPI_ISL_14838587) |
|  |  |  |  | EPI_ISL_18213374 | Unknown(EPI_ISL_19350788) |
|  |  |  |  | PQ451835.1 | Unknown(DQ011154.1) |
|  |  |  |  | PQ451836.1 | Unknown(OR943698.1) |
|  |  |  |  | PQ451838.1 | Unknown(KJ642618.1) |
|  |  |  |  | PQ451841.1 |  |
|  |  |  |  | PQ451842.1 |  |
|  |  |  |  | PQ340477.1 |  |
|  |  |  |  | PQ340478.1 |  |
|  |  |  |  | PQ340480.1 |  |
|  |  |  |  | PQ340483.1 |  |
|  |  |  |  | PQ340485.1 |  |
|  |  |  |  | PQ340486.1 |  |
|  |  |  |  | PQ340487.1 |  |
|  |  |  |  | PQ340489.1 |  |
|  |  |  |  | PQ340490.1 |  |
|  |  |  |  | PQ340491.1 |  |
|  |  |  |  | PQ340493.1 |  |
|  |  |  |  | PQ340495.1 |  |
|  |  |  |  | PQ340496.1 |  |
|  |  |  |  | OR427332.1 |  |
|  |  |  |  | OQ419007.1 |  |
|  |  |  |  | OQ419012.1 |  |
|  |  |  |  | OQ419031.1 |  |
|  |  |  |  | OQ419047.1 |  |
|  |  |  |  | OP879722.1 |  |
|  |  |  |  | OP604566.1 |  |
|  |  |  |  | OP434519.1 |  |
|  |  |  |  | OP150924.1 |  |
|  |  |  |  | ON959133.1 |  |
|  |  |  |  | ON959136.1 |  |
|  |  |  |  | ON676707.1 |  |
|  |  |  |  | LC831698.1 |  |
|  |  |  |  | EPI_ISL_19151800 |  |
|  |  |  |  | EPI_ISL_18557816 |  |
|  |  |  |  | EPI_ISL_19022858 |  |
|  |  |  |  | MT903342.1 |  |
|  |  |  |  | EPI_ISL_16233784 |  |
|  |  |  |  | EPI_ISL_14181948 |  |
|  |  |  |  | EPI_ISL_13363142 |  |
|  |  |  |  | EPI_ISL_13331598 |  |
|  |  |  |  | EPI_ISL_15831211 |  |
|  |  |  |  | EPI_ISL_16946400 |  |
|  |  |  |  | EPI_ISL_19028778 |  |
|  |  |  |  | EPI_ISL_13953610 |  |
|  |  |  |  | ON911481.2 |  |
|  |  |  |  | EPI_ISL_15992098 |  |
|  |  |  |  | EPI_ISL_13624509 |  |
|  |  |  |  | EPI_ISL_13607904 |  |
|  |  |  |  | OP289782.1 |  |
|  |  |  |  | ON649879.1 |  |
|  |  |  |  | MN648051.1 |  |
|  |  |  |  | OR035652.1 |  |
|  |  |  |  | OR035664.1 |  |
|  |  |  |  | OR035702.1 |  |
|  |  |  |  | ON880420.1 |  |
|  |  |  |  | ON622713.1 |  |
|  |  |  |  | PP852983.1 |  |
|  |  |  |  | PP853000.1 |  |
|  |  |  |  | PP853005.1 |  |
|  |  |  |  | PP853007.1 |  |
|  |  |  |  | PP853013.1 |  |
|  |  |  |  | PP853027.1 |  |
|  |  |  |  | PP853045.1 |  |
|  |  |  |  | PP853048.1 |  |
|  |  |  |  | OP535333.1 |  |
|  |  |  |  | OP612682.1 |  |
|  |  |  |  | OP612684.1 |  |
|  |  |  |  | OP612686.1 |  |
|  |  |  |  | MT903338.1 |  |
|  |  |  |  | MT903340.1 |  |
|  |  |  |  | MT903341.1 |  |
|  |  |  |  | PP776553.1 |  |
|  |  |  |  | OQ451898.1 |  |
|  |  |  |  | OQ451900.1 |  |
|  |  |  |  | OP696841.1 |  |
|  |  |  |  | ON568298.1 |  |
|  |  |  |  | EPI_ISL_13889442 |  |
|  |  |  |  | EPI_ISL_17718458 |  |
|  |  |  |  | PQ368489.1 |  |
|  |  |  |  | PQ153226.1 |  |
|  |  |  |  | OP235282.1 |  |
|  |  |  |  | OR264364.1 |  |
|  |  |  |  | ON631963.1 |  |
|  |  |  |  | ON983168.1 |  |
|  |  |  |  | PP098589.2 |  |
|  |  |  |  | EPI_ISL_15263355 |  |
|  |  |  |  | EPI_ISL_13502582 |  |
|  |  |  |  | EPI_ISL_18642365 |  |
|  |  |  |  | EPI_ISL_19159116 |  |
|  |  |  |  | OP422341.1 |  |
|  |  |  |  | OP019276.1 |  |
|  |  |  |  | PP860026.1 |  |
|  |  |  |  | PP860027.1 |  |
|  |  |  |  | PP860028.1 |  |
|  |  |  |  | PP860031.1 |  |
|  |  |  |  | PP860033.1 |  |
|  |  |  |  | OR459797.1 |  |
|  |  |  |  | OP536770.1 |  |
|  |  |  |  | OP331335.1 |  |
|  |  |  |  | ON808413.1 |  |

**Table S5**. The number of dynamic changes of each MPXV gene along the phylogenetic tree of MPXV.

| **Group** | **Gains** | **Losses** | **Copy Number Increases** | **Copy Number Decreases** | **Total Changes** |
| --- | --- | --- | --- | --- | --- |
| OG0000000 | 0 | 1 | 0 | 5 | 6 |
| OG0000001 | 0 | 1 | 0 | 5 | 6 |
| OG0000002 | 0 | 1 | 1 | 8 | 10 |
| OG0000003 | 0 | 1 | 0 | 9 | 10 |
| OG0000004 | 2 | 1 | 1 | 7 | 11 |
| OG0000005 | 0 | 0 | 1 | 1 | 2 |
| OG0000006 | 0 | 2 | 1 | 1 | 4 |
| OG0000007 | 0 | 1 | 0 | 1 | 2 |
| OG0000008 | 0 | 2 | 0 | 1 | 3 |
| OG0000009 | 0 | 3 | 0 | 1 | 4 |
| OG0000010 | 0 | 0 | 2 | 0 | 2 |
| OG0000011 | 0 | 3 | 0 | 1 | 4 |
| OG0000012 | 0 | 0 | 2 | 0 | 2 |
| OG0000013 | 0 | 0 | 1 | 0 | 1 |
| OG0000193 | 0 | 1 | 0 | 0 | 1 |
| OG0000194 | 0 | 1 | 0 | 0 | 1 |
| OG0000195 | 0 | 1 | 0 | 0 | 1 |
| OG0000196 | 0 | 1 | 0 | 0 | 1 |
| OG0000197 | 0 | 1 | 0 | 0 | 1 |
| OG0000198 | 0 | 1 | 0 | 0 | 1 |
| OG0000199 | 0 | 1 | 0 | 0 | 1 |
| OG0000200 | 0 | 1 | 0 | 0 | 1 |
| OG0000201 | 0 | 1 | 0 | 0 | 1 |
| OG0000202 | 0 | 1 | 0 | 0 | 1 |
| OG0000203 | 0 | 2 | 0 | 0 | 2 |
| OG0000204 | 0 | 2 | 0 | 0 | 2 |
| OG0000205 | 0 | 2 | 0 | 0 | 2 |
| OG0000206 | 0 | 1 | 1 | 0 | 2 |
| OG0000207 | 0 | 2 | 0 | 0 | 2 |
| OG0000208 | 0 | 3 | 0 | 0 | 3 |
| OG0000209 | 0 | 4 | 0 | 0 | 4 |
| OG0000210 | 1 | 1 | 0 | 0 | 2 |
| OG0000211 | 1 | 3 | 0 | 0 | 4 |
| OG0000212 | 1 | 3 | 0 | 0 | 4 |
| OG0000213 | 1 | 4 | 0 | 0 | 5 |
| OG0000214 | 3 | 1 | 0 | 0 | 4 |
| OG0000215 | 0 | 1 | 0 | 0 | 1 |
| OG0000216 | 0 | 4 | 0 | 1 | 5 |
| OG0000217 | 0 | 1 | 0 | 0 | 1 |
| OG0000218 | 0 | 1 | 0 | 0 | 1 |
| OG0000219 | 0 | 1 | 0 | 0 | 1 |
| OG0000220 | 0 | 1 | 0 | 0 | 1 |
| OG0000221 | 0 | 2 | 0 | 0 | 2 |
| OG0000222 | 0 | 2 | 0 | 0 | 2 |
| OG0000223 | 1 | 0 | 0 | 0 | 1 |
| OG0000224 | 1 | 0 | 0 | 0 | 1 |
| OG0000225 | 1 | 0 | 0 | 0 | 1 |
| OG0000226 | 1 | 0 | 0 | 0 | 1 |
| OG0000227 | 1 | 0 | 0 | 0 | 1 |
| OG0000228 | 1 | 1 | 0 | 0 | 2 |
| OG0000229 | 1 | 1 | 0 | 0 | 2 |
| OG0000230 | 17 | 12 | 0 | 0 | 29 |
| OG0000231 | 0 | 1 | 0 | 0 | 1 |
| OG0000232 | 0 | 1 | 0 | 0 | 1 |
| OG0000233 | 1 | 0 | 0 | 0 | 1 |
| OG0000234 | 1 | 0 | 0 | 0 | 1 |
| OG0000235 | 1 | 0 | 0 | 0 | 1 |
| OG0000236 | 1 | 0 | 0 | 0 | 1 |
| OG0000237 | 1 | 0 | 0 | 0 | 1 |
| OG0000238 | 1 | 0 | 0 | 0 | 1 |
| OG0000239 | 1 | 0 | 0 | 0 | 1 |
| OG0000240 | 1 | 0 | 0 | 0 | 1 |
| OG0000241 | 1 | 0 | 0 | 0 | 1 |
| OG0000242 | 1 | 0 | 0 | 0 | 1 |
| OG0000243 | 1 | 0 | 0 | 0 | 1 |
| OG0000244 | 1 | 0 | 0 | 0 | 1 |
| OG0000245 | 1 | 1 | 0 | 0 | 2 |
| OG0000246 | 1 | 1 | 0 | 0 | 2 |
| OG0000247 | 1 | 1 | 0 | 0 | 2 |
| OG0000248 | 1 | 0 | 0 | 0 | 1 |
| OG0000249 | 1 | 0 | 0 | 0 | 1 |
| OG0000250 | 1 | 2 | 0 | 0 | 3 |
| OG0000251 | 1 | 2 | 0 | 0 | 3 |
| OG0000252 | 1 | 1 | 0 | 0 | 2 |
| OG0000253 | 1 | 3 | 0 | 0 | 4 |
| OG0000254 | 1 | 2 | 0 | 0 | 3 |
| OG0000255 | 1 | 1 | 0 | 0 | 2 |
| OG0000256 | 1 | 1 | 0 | 0 | 2 |
| OG0000257 | 8 | 0 | 0 | 0 | 8 |
| OG0000258 | 4 | 0 | 0 | 0 | 4 |
| OG0000259 | 4 | 0 | 0 | 0 | 4 |
| OG0000260 | 2 | 2 | 0 | 0 | 4 |
| OG0000261 | 2 | 3 | 0 | 0 | 5 |
| OG0000262 | 0 | 1 | 0 | 1 | 2 |
| OG0000263 | 0 | 1 | 0 | 0 | 1 |
| OG0000264 | 0 | 1 | 0 | 0 | 1 |
| OG0000265 | 0 | 1 | 0 | 0 | 1 |
| OG0000266 | 0 | 1 | 0 | 0 | 1 |
| OG0000267 | 5 | 0 | 0 | 0 | 5 |
| OG0000268 | 4 | 0 | 0 | 0 | 4 |
| OG0000269 | 2 | 0 | 0 | 0 | 2 |
| OG0000270 | 2 | 0 | 0 | 0 | 2 |
| OG0000271 | 2 | 0 | 1 | 0 | 3 |
| OG0000272 | 1 | 0 | 0 | 0 | 1 |
| OG0000273 | 2 | 0 | 0 | 0 | 2 |
| OG0000274 | 2 | 0 | 0 | 0 | 2 |
| OG0000275 | 1 | 0 | 0 | 0 | 1 |
| OG0000276 | 2 | 0 | 0 | 0 | 2 |
| OG0000277 | 1 | 0 | 0 | 0 | 1 |
| OG0000278 | 1 | 0 | 0 | 0 | 1 |
| OG0000279 | 1 | 0 | 0 | 0 | 1 |
| OG0000280 | 1 | 0 | 0 | 0 | 1 |
| OG0000281 | 1 | 0 | 0 | 0 | 1 |
| OG0000282 | 1 | 0 | 0 | 0 | 1 |
| OG0000283 | 1 | 0 | 0 | 0 | 1 |
| OG0000284 | 1 | 0 | 0 | 0 | 1 |
| OG0000285 | 1 | 0 | 0 | 0 | 1 |
| OG0000286 | 1 | 0 | 0 | 0 | 1 |
| OG0000287 | 1 | 0 | 0 | 0 | 1 |
| OG0000288 | 1 | 0 | 0 | 0 | 1 |
| OG0000289 | 1 | 0 | 0 | 0 | 1 |
| OG0000290 | 1 | 0 | 0 | 0 | 1 |
| OG0000291 | 1 | 0 | 0 | 0 | 1 |
| OG0000292 | 1 | 0 | 0 | 0 | 1 |
| OG0000293 | 1 | 0 | 0 | 0 | 1 |
| OG0000294 | 1 | 0 | 0 | 0 | 1 |
| OG0000295 | 1 | 0 | 0 | 0 | 1 |
| OG0000296 | 1 | 0 | 0 | 0 | 1 |
| OG0000297 | 1 | 0 | 0 | 0 | 1 |
| OG0000298 | 1 | 0 | 0 | 0 | 1 |
| OG0000299 | 1 | 0 | 0 | 0 | 1 |
| OG0000300 | 1 | 0 | 0 | 0 | 1 |
| OG0000301 | 1 | 0 | 0 | 0 | 1 |
| OG0000302 | 1 | 0 | 0 | 0 | 1 |
| OG0000303 | 1 | 0 | 0 | 0 | 1 |
| OG0000304 | 1 | 0 | 0 | 0 | 1 |
| OG0000305 | 1 | 0 | 0 | 0 | 1 |
| OG0000306 | 1 | 0 | 0 | 0 | 1 |
| OG0000307 | 1 | 0 | 0 | 0 | 1 |
| OG0000308 | 1 | 0 | 0 | 0 | 1 |
| OG0000309 | 1 | 0 | 0 | 0 | 1 |
| OG0000310 | 1 | 0 | 0 | 0 | 1 |
| OG0000311 | 1 | 0 | 0 | 0 | 1 |
| OG0000312 | 1 | 0 | 0 | 0 | 1 |
| OG0000313 | 1 | 0 | 0 | 0 | 1 |
| OG0000314 | 1 | 0 | 0 | 0 | 1 |
| OG0000315 | 1 | 0 | 0 | 0 | 1 |
| OG0000316 | 1 | 0 | 0 | 0 | 1 |
| OG0000317 | 1 | 0 | 0 | 0 | 1 |
| OG0000318 | 1 | 0 | 0 | 0 | 1 |
| OG0000319 | 1 | 0 | 0 | 0 | 1 |
| OG0000320 | 1 | 0 | 0 | 0 | 1 |
| OG0000321 | 1 | 0 | 0 | 0 | 1 |
| OG0000322 | 1 | 0 | 0 | 0 | 1 |
| OG0000323 | 1 | 0 | 0 | 0 | 1 |
| OG0000324 | 1 | 0 | 0 | 0 | 1 |
| OG0000325 | 1 | 0 | 0 | 0 | 1 |
| OG0000326 | 1 | 0 | 0 | 0 | 1 |
| OG0000327 | 1 | 0 | 0 | 0 | 1 |
| OG0000328 | 1 | 0 | 0 | 0 | 1 |
| OG0000329 | 1 | 0 | 0 | 0 | 1 |
| OG0000330 | 1 | 0 | 0 | 0 | 1 |
| OG0000331 | 1 | 0 | 0 | 0 | 1 |
| OG0000332 | 1 | 0 | 0 | 0 | 1 |
| OG0000333 | 1 | 0 | 0 | 0 | 1 |
| OG0000334 | 1 | 0 | 0 | 0 | 1 |
| OG0000335 | 1 | 0 | 0 | 0 | 1 |
| OG0000336 | 1 | 0 | 0 | 0 | 1 |
| OG0000337 | 1 | 0 | 0 | 0 | 1 |
| OG0000338 | 1 | 0 | 0 | 0 | 1 |
| OG0000339 | 1 | 0 | 0 | 0 | 1 |
| OG0000340 | 1 | 0 | 0 | 0 | 1 |
| OG0000341 | 1 | 0 | 0 | 0 | 1 |
| OG0000342 | 1 | 0 | 0 | 0 | 1 |
| OG0000343 | 1 | 0 | 0 | 0 | 1 |
| OG0000344 | 1 | 0 | 0 | 0 | 1 |
| OG0000345 | 1 | 0 | 0 | 0 | 1 |
| OG0000346 | 1 | 0 | 0 | 0 | 1 |
| OG0000347 | 1 | 0 | 0 | 0 | 1 |
| OG0000348 | 1 | 0 | 0 | 0 | 1 |
| OG0000349 | 1 | 0 | 0 | 0 | 1 |
| OG0000350 | 1 | 0 | 0 | 0 | 1 |
| OG0000351 | 1 | 0 | 0 | 0 | 1 |
| OG0000352 | 1 | 0 | 0 | 0 | 1 |
| OG0000353 | 1 | 0 | 0 | 0 | 1 |
| OG0000354 | 1 | 0 | 0 | 0 | 1 |
| OG0000355 | 1 | 0 | 0 | 0 | 1 |
| OG0000356 | 1 | 0 | 0 | 0 | 1 |
| OG0000357 | 1 | 0 | 0 | 0 | 1 |
| OG0000358 | 1 | 0 | 0 | 0 | 1 |
| OG0000359 | 1 | 0 | 0 | 0 | 1 |
| OG0000360 | 1 | 0 | 0 | 0 | 1 |
| OG0000361 | 1 | 0 | 0 | 0 | 1 |
| OG0000362 | 1 | 0 | 0 | 0 | 1 |
| OG0000363 | 1 | 0 | 0 | 0 | 1 |
| OG0000364 | 1 | 0 | 0 | 0 | 1 |
| OG0000365 | 1 | 0 | 0 | 0 | 1 |
| OG0000366 | 1 | 0 | 0 | 0 | 1 |
| OG0000367 | 1 | 0 | 0 | 0 | 1 |
| OG0000368 | 1 | 0 | 0 | 0 | 1 |
| OG0000369 | 1 | 0 | 0 | 0 | 1 |
| OG0000370 | 1 | 0 | 0 | 0 | 1 |
| OG0000371 | 1 | 0 | 0 | 0 | 1 |
| OG0000372 | 1 | 0 | 0 | 0 | 1 |
| OG0000373 | 1 | 0 | 0 | 0 | 1 |
| OG0000374 | 1 | 0 | 0 | 0 | 1 |
| OG0000375 | 1 | 0 | 0 | 0 | 1 |
| OG0000376 | 1 | 0 | 0 | 0 | 1 |
| OG0000377 | 1 | 0 | 0 | 0 | 1 |
| OG0000378 | 1 | 0 | 0 | 0 | 1 |
| OG0000379 | 1 | 0 | 0 | 0 | 1 |
| OG0000380 | 1 | 0 | 0 | 0 | 1 |
| OG0000381 | 1 | 0 | 0 | 0 | 1 |
| OG0000382 | 1 | 0 | 0 | 0 | 1 |
| OG0000383 | 1 | 0 | 0 | 0 | 1 |
| OG0000384 | 1 | 0 | 0 | 0 | 1 |
| OG0000385 | 1 | 0 | 0 | 0 | 1 |
| OG0000386 | 1 | 0 | 0 | 0 | 1 |
| OG0000387 | 1 | 0 | 0 | 0 | 1 |
| OG0000388 | 1 | 0 | 0 | 0 | 1 |
| OG0000389 | 1 | 0 | 0 | 0 | 1 |
| OG0000390 | 1 | 0 | 0 | 0 | 1 |
| OG0000391 | 1 | 0 | 0 | 0 | 1 |
| OG0000392 | 1 | 0 | 0 | 0 | 1 |
| OG0000393 | 1 | 0 | 0 | 0 | 1 |
| OG0000394 | 1 | 0 | 0 | 0 | 1 |
| OG0000395 | 1 | 0 | 0 | 0 | 1 |
| OG0000396 | 1 | 0 | 0 | 0 | 1 |
| OG0000397 | 1 | 0 | 0 | 0 | 1 |
| OG0000398 | 1 | 0 | 0 | 0 | 1 |
| OG0000399 | 1 | 0 | 0 | 0 | 1 |
| OG0000400 | 1 | 0 | 0 | 0 | 1 |
| OG0000401 | 1 | 0 | 0 | 0 | 1 |
| OG0000402 | 1 | 0 | 0 | 0 | 1 |
| OG0000403 | 1 | 0 | 0 | 0 | 1 |
| OG0000404 | 1 | 0 | 0 | 0 | 1 |
